# Substantial Neotropical bat diversity likely remains undescribed and vulnerable to extinction

**DOI:** 10.1038/s41467-026-78393-0

**Published:** 2026-11-03

**Authors:** Diego A. Esquivel, Angie Penagos, Anderson Feijó, Maria João Ramos Pereira

**Affiliations:** 1 Programa de Pós-graduação em Biologia Animal, Universidade Federal do Rio Grande do Sul, Porto Alegre, Brazil; 2 Negaunee Integrative Research Center, Field Museum of Natural History, Chicago, IL USA; 3 Centre for Environmental and Marine Studies, Universidade de Aveiro, Aveiro, Portugal

**Keywords:** Conservation biology, Biodiversity

## Abstract

Numerous undescribed species face extinction before formal scientific recognition. Despite research advances, the exceptional bat diversity of the Neotropics remains overlooked, with accelerating habitat loss threatening unrecognized lineages with silent extinction, requiring urgent conservation prioritization. Here, we integrate molecular species delimitation, phylogenetic inference, ecological niche modelling, and spatial conservation analyses to identify 95 Neotropical bat lineages that may represent undescribed species within 45 complexes, revealing that at least one-fifth of the recognized taxa may harbor hidden diversity spanning the entire evolutionary spectrum. This overlooked diversity faces a countdown to extinction: 62% of complexes face imminent habitat loss, and most are projected to lose over 50% of their suitable habitat by 2070. At current description rates, formally recognizing this diversity would take decades, a timeframe these putative species cannot afford. We develop a Hidden Species Richness (HSR) Index that pinpoints ten hidden diversity hotspots where undescribed richness coincides with significant human threats and extreme climate vulnerability. These hotspots highlight regions where research efforts are most needed, transforming crises into priorities. This race against extinction requires immediate taxonomic attention to document this biodiversity before it vanishes.

## Abstract (web summary)

Many Neotropical bat species may be disappearing before scientists can even describe them. The study identified 95 candidate lineages across 45 complexes and found many overlap with areas of habitat loss, indicating urgent conservation needs.

## Introduction

Scientists race against time to discover and describe new species as global biodiversity faces increasingly severe threats, including habitat destruction, climate change, invasive species, and anthropogenic disturbance. Although taxonomists describe hundreds of new species every year, the true extent of global biodiversity and the number of undescribed species remain elusive^1,2^. Conservative estimates suggest our planet harbors between 5 and 9 million species^3,4^, yet scientists have formally named only about 1.9 million^5,6^, with much of this discrepancy attributed to cryptic and unrecognized diversity. This knowledge gap, known as the Linnean shortfall^7,8^, is particularly concerning because individual species are the primary currency of biodiversity research, as well as the foundation in ecology, evolution, and conservation^9^. So, understanding real species diversity is critical not only for exploring patterns and processes shaping biodiversity but also for developing effective conservation initiatives^10^.

Current extinction rates, while debated in scale, are exceeding natural levels by several orders of magnitude^11–16^, underscoring the urgency of addressing the Linnean shortfall. According to the IUCN, thousands of species are categorized as critically endangered (CR), endangered (EN), or vulnerable (VU), but these figures likely underestimate the true scale of biodiversity at risk, even more so if we consider data deficient (DD) and not-assessed taxa as well as many undescribed or hidden species that may already be threatened due to their occurrence in areas under intense anthropogenic pressure. Among these species are the so-called “cryptic species,” distinct evolutionary lineages that have diverged genetically but remain morphologically indistinguishable^17^. Hidden diversity, whether cryptic or simply unrecognized, represents a significant and overlooked portion of global biodiversity^18^. This diversity often lies outside conservation priorities, because unrecognized species are not included in conservation planning or policy frameworks, exacerbating the Linnean shortfall and leaving many species at risk of extinction before they are even documented^19^.

Several examples show us the implications of hidden diversity and the importance of addressing it quickly. The Tapanuli orangutan (*Pongo tapanuliensis*) was described in 2017 and immediately classified as critically endangered, with fewer than 800 individuals remaining in the wild^20^. The Sardinian long-eared bat (*Plecotus sardus*) was described in 2002 but has suffered a dramatic decline from ~1000 individuals to ~350 individuals in 2020, leading to reclassification from vulnerable to critically endangered^21^. Like these, hidden species represent an essential problem for the advancement of biodiversity conservation: how many undescribed species are there, and how many will disappear in the coming decades if taxonomic efforts and subsequent conservation initiatives do not accelerate?

Bats illuminate the urgency of this challenge. They comprise the second most diverse mammalian order after rodents, with more than 1500 described species playing crucial ecological roles in seed dispersal, pollination, and insect control across ecosystems worldwide^22,23^. Nevertheless, due to their nocturnal habits, elusive behaviors, the ability to fly, and the challenges of assessing them in the wild, current estimates of bat species richness are likely underestimated^23^. This group has seen a significant increase in new species descriptions in recent years, driven primarily by advances in molecular techniques that can readily detect cryptic diversity despite morphological similarity. Over the past two decades, taxonomists have described over 300 bat species^24^, many of which were previously hidden within morphologically similar complexes. However, although bats are considered one of the better-studied mammalian orders, numerous studies continue to uncover hidden diversity within many bat taxa, highlighting the need for a comprehensive revision^25–29^.

Neotropical bats represent an excellent model system to study the conservation implications of hidden diversity, at scale. Its exceptional diversity (harboring ~30% of recognized bat species) and rapid habitat loss provide a setting that allows us to evaluate how undescribed species experience extinction risk even prior to formal taxonomic recognition. Here, we aim to investigate the extent and magnitude of bat hidden diversity in the Neotropics, one of Earth’s most biodiverse yet threatened regions. In particular, we address four key questions: (1) How many hidden bat species remain undescribed in the Neotropics? (2) Where are they? (3) Which areas, considering human-induced landscape modification and climate change, are critical for prioritizing time, funding, research, and conservation efforts focused on describing and protecting hidden species? And (4) How many hidden species could lose significant areas of suitable habitat in the next decades due to climate change? We expect that: (1) substantial hidden diversity exists in Neotropical bats, particularly in morphologically conservative groups; (2) hidden diversity is concentrated in climatically and topographically complex regions with high habitat heterogeneity; and (3) unrecognized species face disproportionate extinction risk due to climate change and uneven human-induced landscape modification.

To address these goals, we employ phylogenetic analyses, molecular species delimitation methods, modern spatial analyses, and ecological niche modelling. By leveraging extensive mitochondrial DNA datasets from public repositories, we identify and map hidden bat species across the Neotropics. Our study highlights regions with elevated hidden species richness (HSR) and identifies hotspots requiring immediate attention. It aims to document hidden diversity before it vanishes and to provide scientific foundations for conservation strategies addressing both current and future threats to undescribed biodiversity. These findings can guide environmental policymakers, institutions, and conservation funding toward urgent taxonomic and geographic priorities.

## Results

**Fig. 1:**
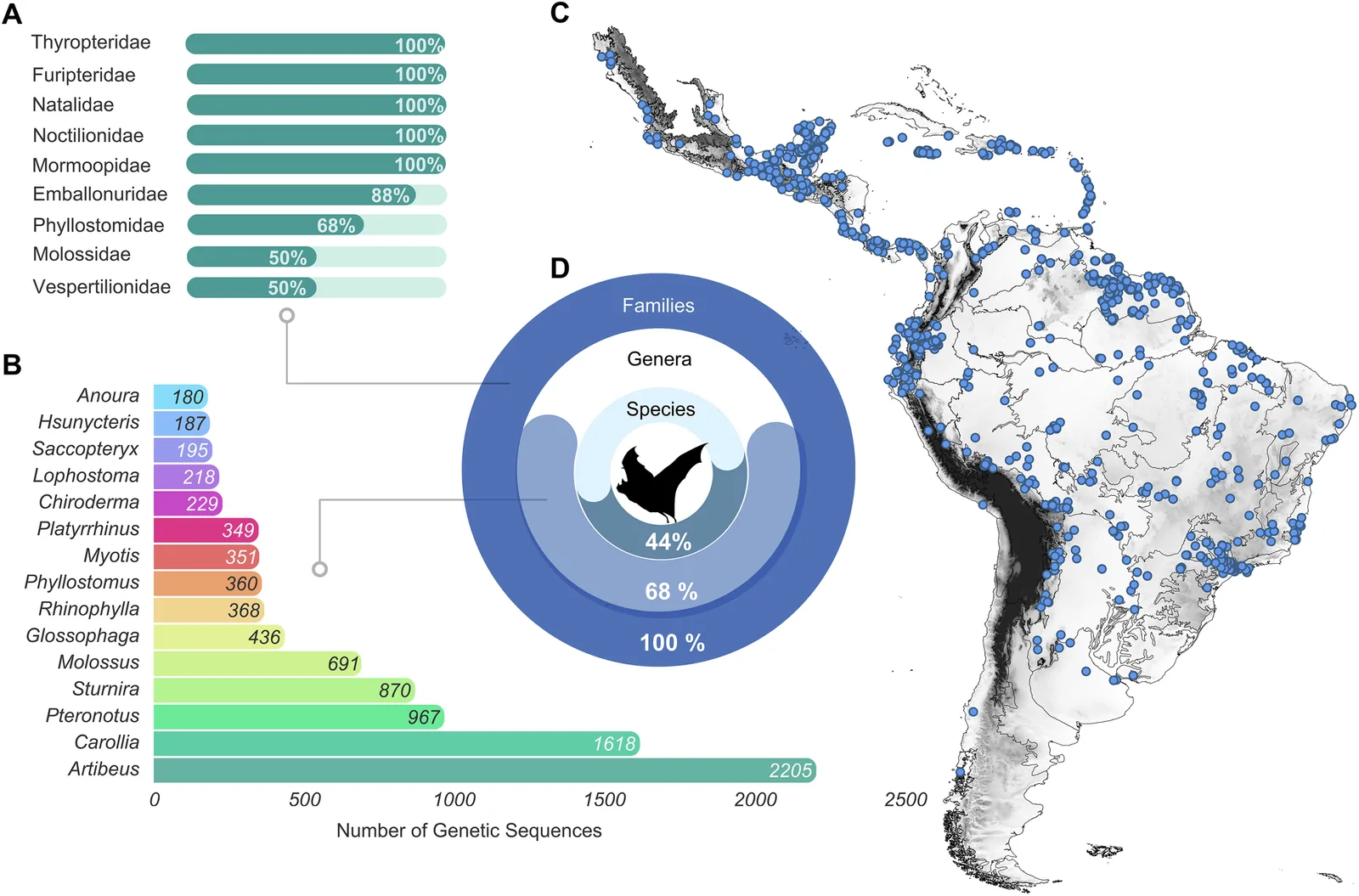
Taxonomic sampling and geographic coverage of COI sequences in Neotropical bats. **A** Proportion of genera with available COI sequences by family. Five families have complete coverage: Thyropteridae, Furipteridae, Natalidae, Noctilionidae, and Mormoopidae, while Emballonuridae (88%), Phyllostomidae (68%), Molossidae, and Vespertilionidae (50%) have partial representation. **B** Top 15 most sequenced genera with the number of sequences on the x-axis. **C** Spatial distribution of curated and validated COI sequences across the Neotropical realm, overlaid on Morrone´s biogeographic ﻿provinces^58^. **D** Hierarchical representation of taxonomic coverage showing complete family-level sampling (outer ring, 100%), high genus-level representation (middle ring, 68%), and intermediate species-level coverage (inner ring, 44%).

We analyzed a molecular dataset of 12,813 sequences of Neotropical bats and constructed 63 phylogenies covering 65 genera to evaluate the hidden diversity patterns in this group. Our analysis achieved complete coverage of all nine bat families present in the region, encompassing ~68% of known genera (65 of 95) and ~44% of currently recognized species (191 of 435). Taxonomically and ecologically diverse families like Phyllostomidae showed extensive representation, while less diverse families like Furipteridae and Natalidae had fewer records. The frugivorous genera *Artibeus* and *Carollia* emerged as the most extensively sequenced taxa (2204 and 1619 sequences, respectively), followed by the insectivorous *Pteronotus* (967) and the frugivorous *Sturnira* (925) (Fig. 1).

The geographic distribution of COI sequences revealed distinct clusters across the Neotropics, with four densely sampled provinces: the Guianan Lowlands (6151 sequences), Guianan (1174 sequences), Napo (997 sequences), and Veracruzan provinces (516 sequences), but also uncovered substantial sampling gaps across vast regions of South America. Large regions in southern South America, the Amazon, the Andes, the west coast of South America, and some central areas of Mesoamerican forests lack genetic sequences, indicating a significant gap in available data for these areas.

### Hidden species identification

**Fig. 2:**
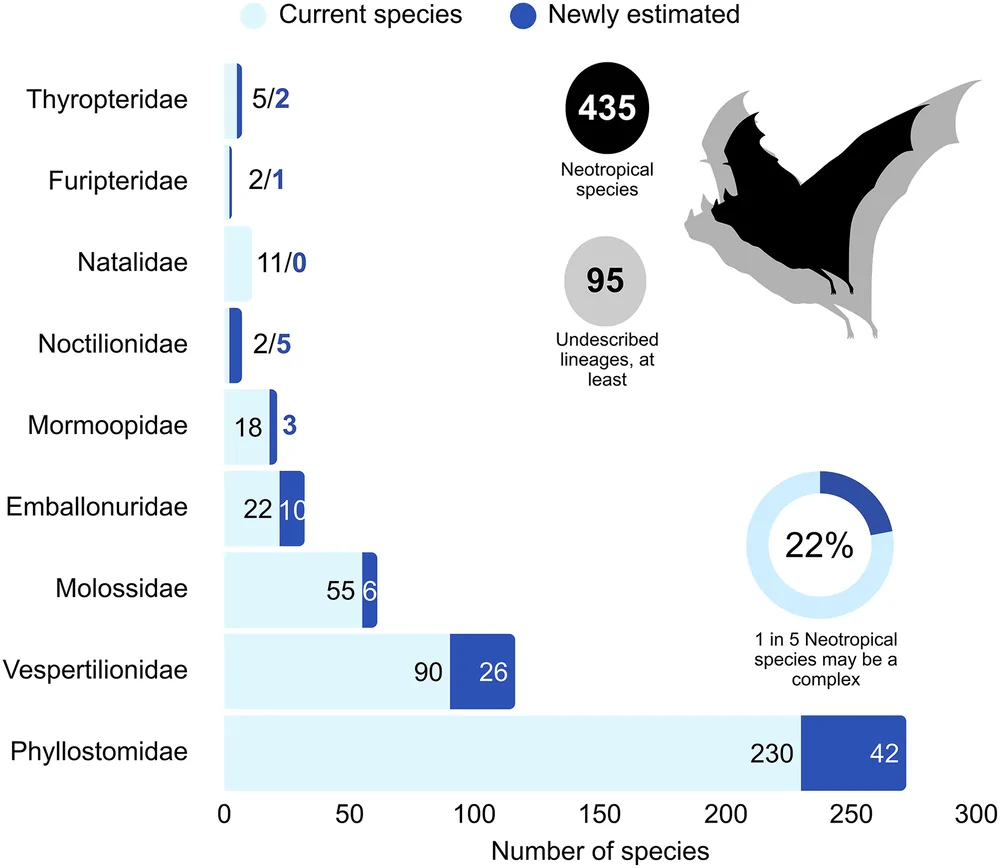
Hidden diversity in Neotropical bats. Bar chart compares current recognized species (light blue with black numbers) with newly estimated species (dark blue with white numbers) across nine bat families. The Phyllostomidae family shows the highest absolute diversity with 230 described species and an additional 42 estimated undescribed lineages. Silhouette depicts a bat, with its shadow representing the ratio between predicted potentially undescribed species and currently recognized species in Neotropical bat families.

Our species delimitation analyses identified 45 species complexes harboring 95 hidden lineages across the Neotropical realm, which may represent potentially undescribed species pending formal taxonomic validation (Supplementary Table 1). Hidden diversity is distributed across all nine families, with Phyllostomidae (42 newly estimated species) and Vespertilionidae^26^ showing the highest hidden richness. These unrecognized evolutionary units span diverse ecological and geographic scales, from complexes widely distributed across multiple biomes like *Noctilio leporinus, Lophostoma silvicola*, and *Phyllostomus hastatus*, to geographically restricted complexes like *Monophyllus redmani*. Notably, these hidden complexes occur across the entire phylogenetic spectrum of Neotropical bats (see Supplementary Figs. 1–45 for phylogenies, maps, intra- and interspecific distances, and hidden lineages to genus-level), transcending ecological guilds and habitat preferences (Fig. 2).

### Hidden bat diversity hotspots

**Fig. 3:**
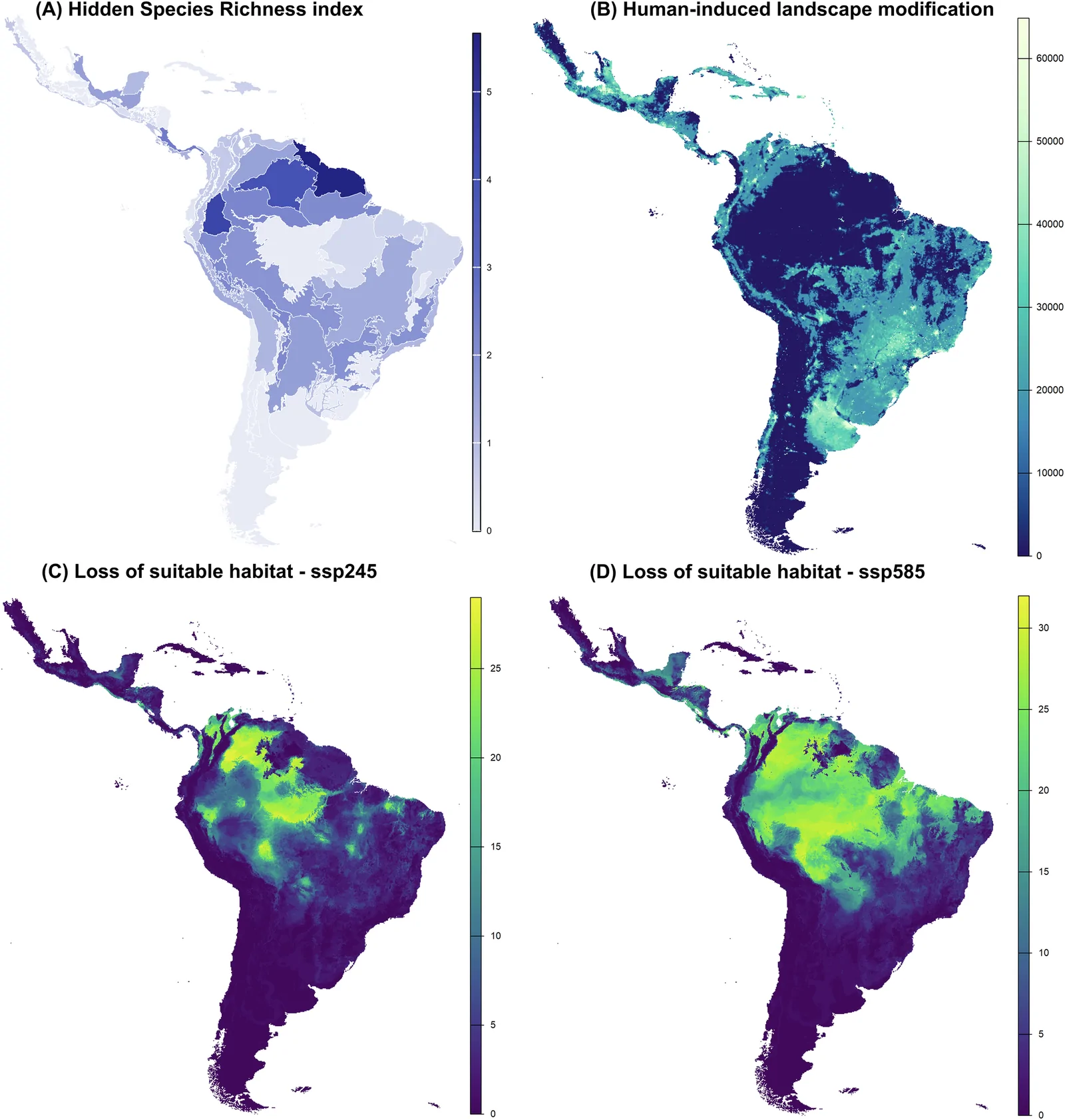
Three different layers for modeling hidden diversity hotspots. **A** Hidden species richness (HSR) index showing areas of potential undescribed bat diversity. **B** Human-induced landscape modification intensity adapted from Theobald et al.^39^. **C** Projected loss of suitable habitat for bat species complexes under SSP245 climate scenario (moderate emissions). **D** Projected loss of suitable habitat under SSP585 climate scenario (high emissions).

Our HSR index, which estimates hidden richness while accounting for spatial biases in genetic sampling, reveals that the northern and northeastern regions of South America, the eastern Ecuadorian and Peruvian Amazon, and the central-southern region of Brazil harbor the highest levels of hidden bat richness in the Neotropics, ranging from 5 to 31 potential undescribed lineages (Fig. 3A). Most of these regions coincide with high human modification, especially in the lowlands Central-America, Andes, northern South America, and southeastern Brazil (Fig. 3B). We also identified that the greatest potential loss of species complexes due to reductions in suitable habitat resulting from climatic changes will occur in areas with high HSR, such as the Amazon region in Guianas, Brazil, Venezuela, Colombia and Ecuador, and the eastern plains of Colombia and Venezuela. Similarly, northern Colombia stands out as one of the regions that will lose more areas of suitable habitats for complexes with hidden diversity in the next 45 years (Fig. 3C, D, and see Supplementary Figs. 46–87 for current and future distribution models for each species complex).

**Fig. 4:**
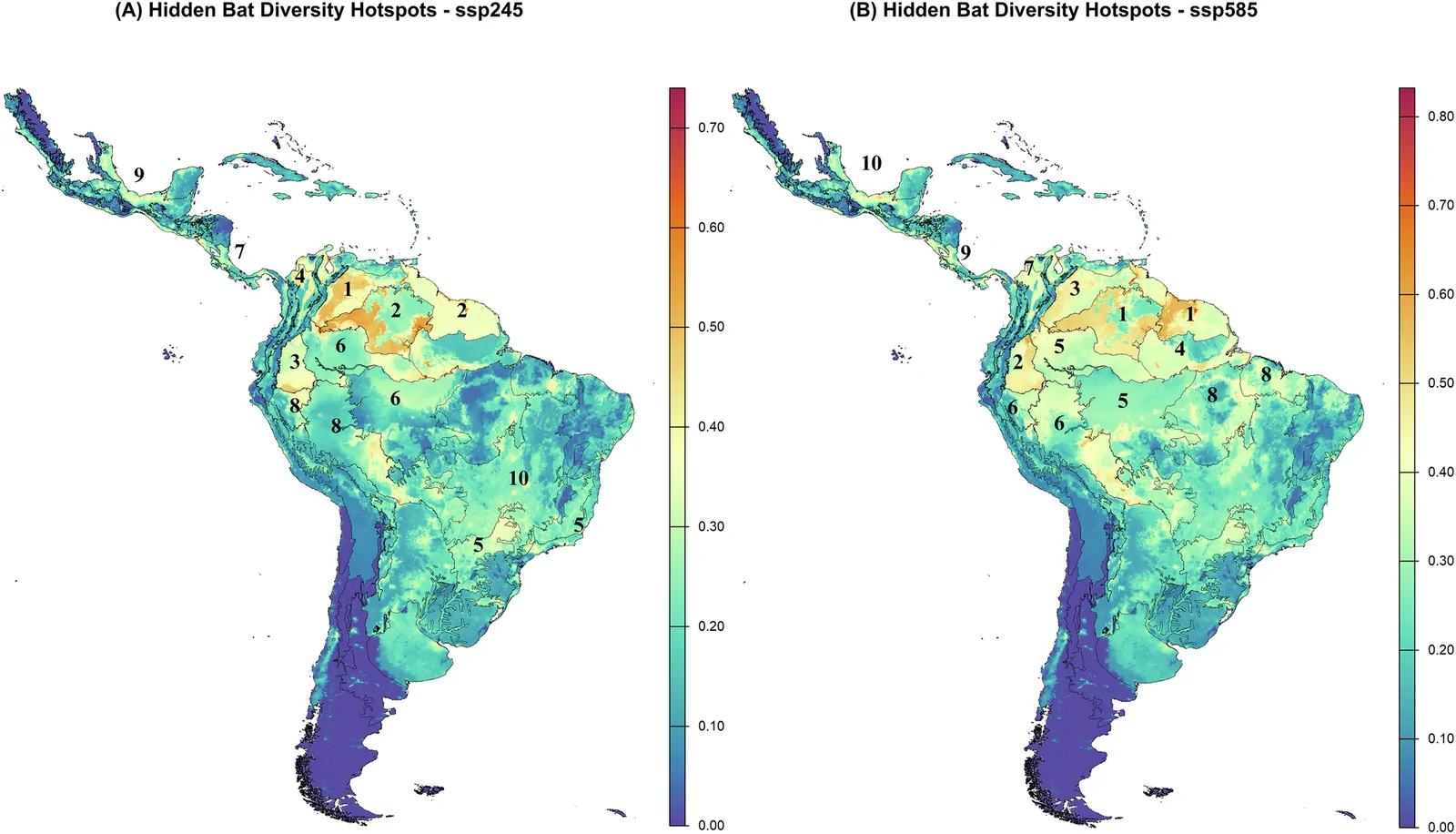
Hidden diversity hotspots for Neotropical bats. **A** Hotspots under SSP245, where values from 0.00 (blue) to 0.70 (red) represent a composite value integrating hidden species richness (HSR) index, human impact, and projected habitat suitability loss by 2070. **B** Hotspots under SSP585, where values from 0.00 (blue) to 0.80 (red) show increased vulnerability in northeastern South America and parts of Central America under more extreme climate projections. Higher values (warmer colors) indicate areas of maximum conservation urgency. Numbers represent the 10 main hotspots for hidden bat diversity in the Neotropics.

By integrating HSR with metrics of anthropogenic change and projected losses in habitat suitability, we recognized 10 major hotspots of hidden diversity, each one showing its minimum number of undescribed lineages: (1) Amazonian forests of northeastern South America (Guianan and Guianan Lowlands provinces)—31, (2) Ecuadorian Amazon (Napo province)—23, (3) Eastern plains of Colombia and Venezuela (Sabana province)—7, (4) North Amazon in Brazil (Roraima province)—6, (5) Southern Colombian Amazon and northwestern Brazilian Amazon (Imeri and Madeira provinces)—2, (6) Eastern Amazon and plains of Peru and Bolivia (Uyucali and Rondonia provinces)—9, (7) Caribbean and Inter-Andean region in Colombia (Guajira and Magdalena forests)—8, (8) Eastern Brazil (Para and Xingu-Tapajos provinces)—4, (9) Central forests in Panama and Costa Rica (Guatuso-Talamanca and Puntarenas-Chiriquí provinces)—22, and (10) Eastern coast of Mexico (Veracruzan province)—11 (Fig. 4). See complete list of provinces in Supplementary Tables 2 and 3.

### Priority species complex

In addition to revealing the hotspots of hidden diversity, we identified seven species complexes categorized as “Very High” conservation priority, representing evolutionary lineages facing imminent extinction risk before formal description. Additionally, 11 complexes were categorized as “High” priority, 16 as “Medium,” two as “Low,” and nine as “Very Low” priority. While these complexes face lower immediate threats, they still need research attention to unveil their hidden diversity before potential future declines (Table 1).

**Table 1:** Priority hidden species complexes

Species complex	IUCN status	Area loss (%) ssp245	Area loss (%) ssp585	Terrestrial EOO (km^2^)	Human-induced landscape modification (%)	Unified score	Priority level
*Monophyllus redmani*	LC	NA	NA	186,742	82.74	3.00	Very high
*Anoura luismanueli*	LC	32.47	44.93	48,136	65.25	3.00	Very high
*Hylonycteris underwoodi*	LC	49.17	70.54	740,184	54.34	2.75	Very high
*Histiotus velatus*	DD	36.56	57.26	5,236,129	49.52	2.75	Very high
*Macrophyllum macrophyllum*	LC	65.66	92.98	12,181,080	28.13	2.75	Very high
*Thyroptera tricolor*	LC	45.22	80.97	11,277,030	28.08	2.75	Very high
*Eumops hansae*	LC	62.17	96.50	8,093,269	22.84	2.75	Very high
*Pteronotus pusillus*	NE	NA	NA	57,103	81.68	2.50	High
*Glyphonycteris sylvestris*	LC	31.59	61.83	12,487,242	29.08	2.50	High
*Myotis riparius*	LC	55.52	75.56	14,824,227	28.85	2.50	High
*Anoura caudifer*	LC	67.95	76.49	11,833,214	28.04	2.50	High
*Trachops cirrhosus*	LC	33.05	66.88	12,594,206	27.11	2.50	High
*Rhynchonycteris naso*	LC	21.62	65.58	12,372,620	25.23	2.50	High
*Diclidurus albus*	LC	33.00	67.40	11,115,323	23.60	2.50	High
*Trinycteris nicefori*	LC	22.35	67.44	10,466,135	21.15	2.50	High
*Lophostoma silvicola*	LC	44.29	77.27	10,024,190	20.34	2.50	High
*Choeroniscus minor*	LC	67.18	84.67	8,991,000	19.70	2.50	High
*Neoeptesicus chiriquinus*	LC	77.62	83.58	4,905,640	10.26	2.50	High
*Histiotus montanus*	LC	35.11	46.45	10,758,888	31.21	2.25	Medium
*Lasiurus ega*	LC	16.92	42.33	17,276,247	30.82	2.25	Medium
*Neoeptesicus furinalis*	LC	27.46	56.82	17,925,685	30.59	2.25	Medium
*Nyctinomops laticaudatus*	LC	20.97	50.54	15,876,917	30.06	2.25	Medium
*Noctilio leporinus*	LC	19.67	51.89	13,437,403	29.74	2.25	Medium
*Neoeptesicus brasiliensis*	LC	24.97	57.82	16,701,492	29.73	2.25	Medium
*Myotis nigricans*	LC	20.45	51.79	15,215,525	28.83	2.25	Medium
*Desmodus rotundus*	LC	15.92	47.60	15,337,934	27.87	2.25	Medium
*Chrotopterus auritus*	LC	25.39	51.92	14,044,374	27.84	2.25	Medium
*Noctilio albiventris*	LC	16.13	57.95	16,143,161	27.17	2.25	Medium
*Nyctinomops macrotis*	LC	19.79	42.35	15,159,902	26.92	2.25	Medium
*Saccopteryx bilineata*	LC	12.67	45.52	12,684,356	26.07	2.25	Medium
*Carollia castanea*	LC	53.22	77.89	4,622,473	19.14	2.25	Medium
*Pteronotus personatus*	LC	20.97	62.37	7,296,081	16.89	2.25	Medium
*Molossops neglectus*	DD	NA	NA	5,887,517	13.92	2.25	Medium
*Cormura brevirostris*	LC	32.86	64.57	6,880,194	12.84	2.25	Medium
*Dermanura watsoni*	LC	4.69	15.85	1,084,939	43.25	2.00	Low
*Anoura cultrata*	LC	39.25	52.38	3,724,873	14.90	2.00	Low
*Peropteryx macrotis*	LC	1.24	1.25	14,591,584	27.56	1.75	Very low
*Furipterus horrens*	LC	9.12	13.33	11,738,988	26.91	1.75	Very low
*Phyllostomus hastatus*	LC	5.48	7.92	12,604,866	26.87	1.75	Very low
*Phylloderma stenops*	LC	11.40	12.83	10,070,844	23.46	1.75	Very low
*Tonatia maresi*	NE	34.47	60.88	8,350,575	14.16	1.75	Very low
*Anoura peruana*	LC	27.03	37.61	2,454,375	13.46	1.75	Very low
*Dermanura anderseni*	LC	17.70	19.43	3,297,591	13.45	1.50	Very low
*Hsunycteris thomasi*	LC	9.71	16.88	6,637,908	10.41	1.50	Very low
*Cyttarops alecto*	LC	8.71	18.37	3,842,522	10.36	1.50	Very low

Ranking of 45 bat species complexes with potential undescribed diversity, based on Unified Score (see “Materials and Methods” for details). Priority levels integrate IUCN status of entities currently recognized as species but which, in our analysis, present hidden diversity, projected habitat loss under moderate (ssp245) and severe (ssp585) climate scenarios, geographic range size (Terrestrial EOO km²), and human-induced landscape modification intensity (%). Higher scores (scale 1.5–3) indicate greater conservation urgency. NA represents data that could not be obtained.

**Fig. 5:**
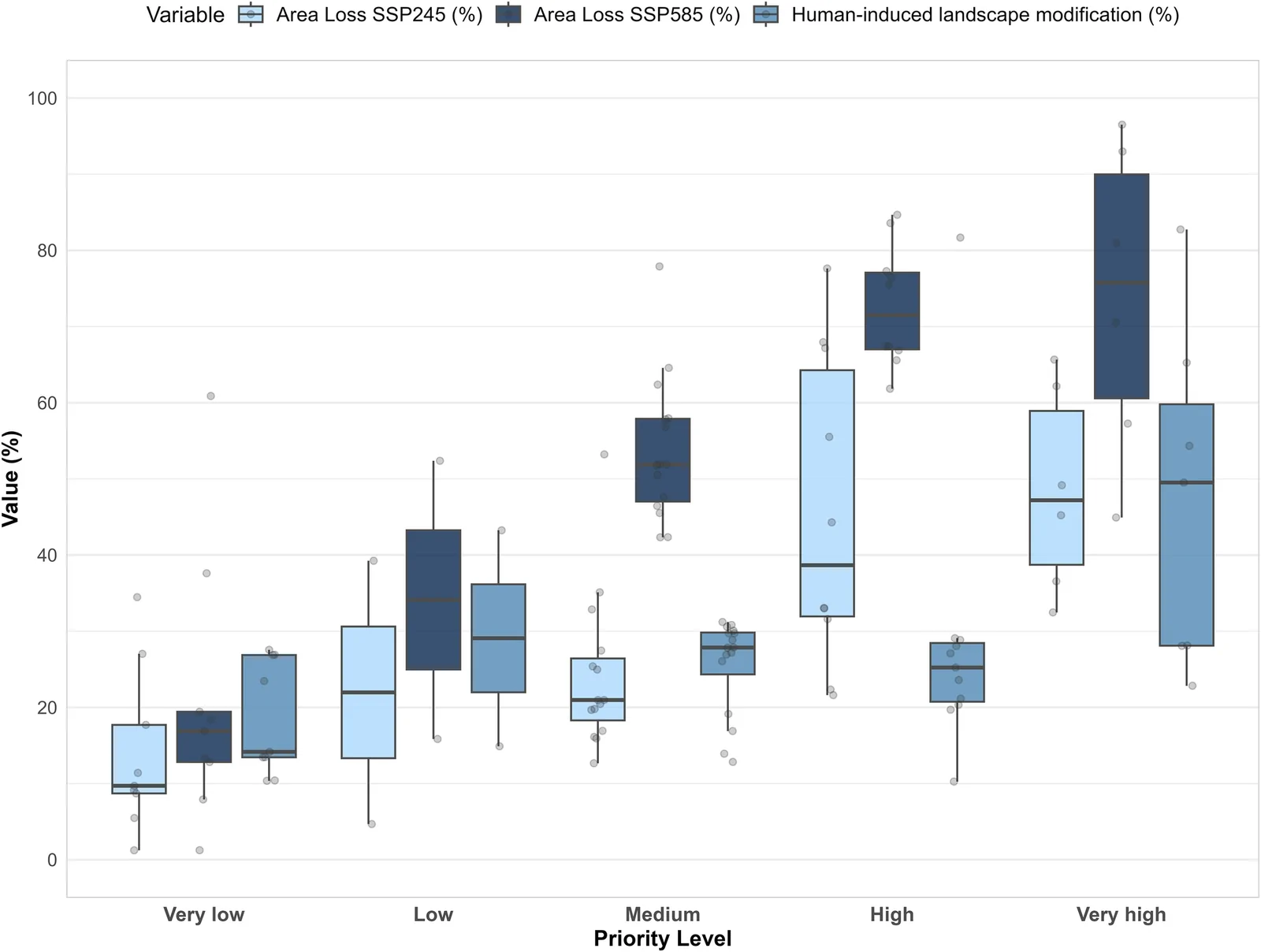
Threat intensity across conservation priority levels for bat species complexes. Boxplots representing three vulnerability metrics across five priority levels: Very low (*n* = 9), Low (*n* = 2), Medium (*n* = 16), High (*n* = 11), and Very high (*n* = 7), for a total of 45 species complexes. Area Loss SSP245 and SSP585 show the projected percentage of suitable habitat lost by 2070 under moderate and severe climate change scenarios, respectively. Human-induced landscape modification represents the percentage of each species complex’s range currently affected by anthropogenic habitat transformation. Boxes represent the first and third quartiles, the centre line the mean, and whiskers the minimum and maximum values. Each point represents one species complex and constitutes an independent observational unit; no technical replicates were used. Higher priority levels generally correspond to greater threats across all metrics.

Our results point to differences in how species complexes are affected by climate change and human-induced landscape modification (Fig. 5). Under the most severe climate scenario (SSP585), very-high priority species complexes are projected to lose on average 73.8% of their suitable habitats (range 44.9–96.5%). Even under moderate scenarios, projected losses remain alarming (range 32.4–65.6%). Very-high priority complexes also face potential threats from human-induced landscape modification, with ~50% of their ranges already modified by human activity, compared to 28.6% for high-priority and 25.6% for medium-priority complexes. Lower-priority species complexes, while less vulnerable overall, show varied responses to climate change, with potential losses of suitable habitat ranging from 1.2 to 60.8%. These findings highlight the urgent need for targeted conservation efforts, especially for very-high and high-priority species, which are most exposed to climate intensification and human-induced landscape modification (Fig. 5).

**Fig. 6:**
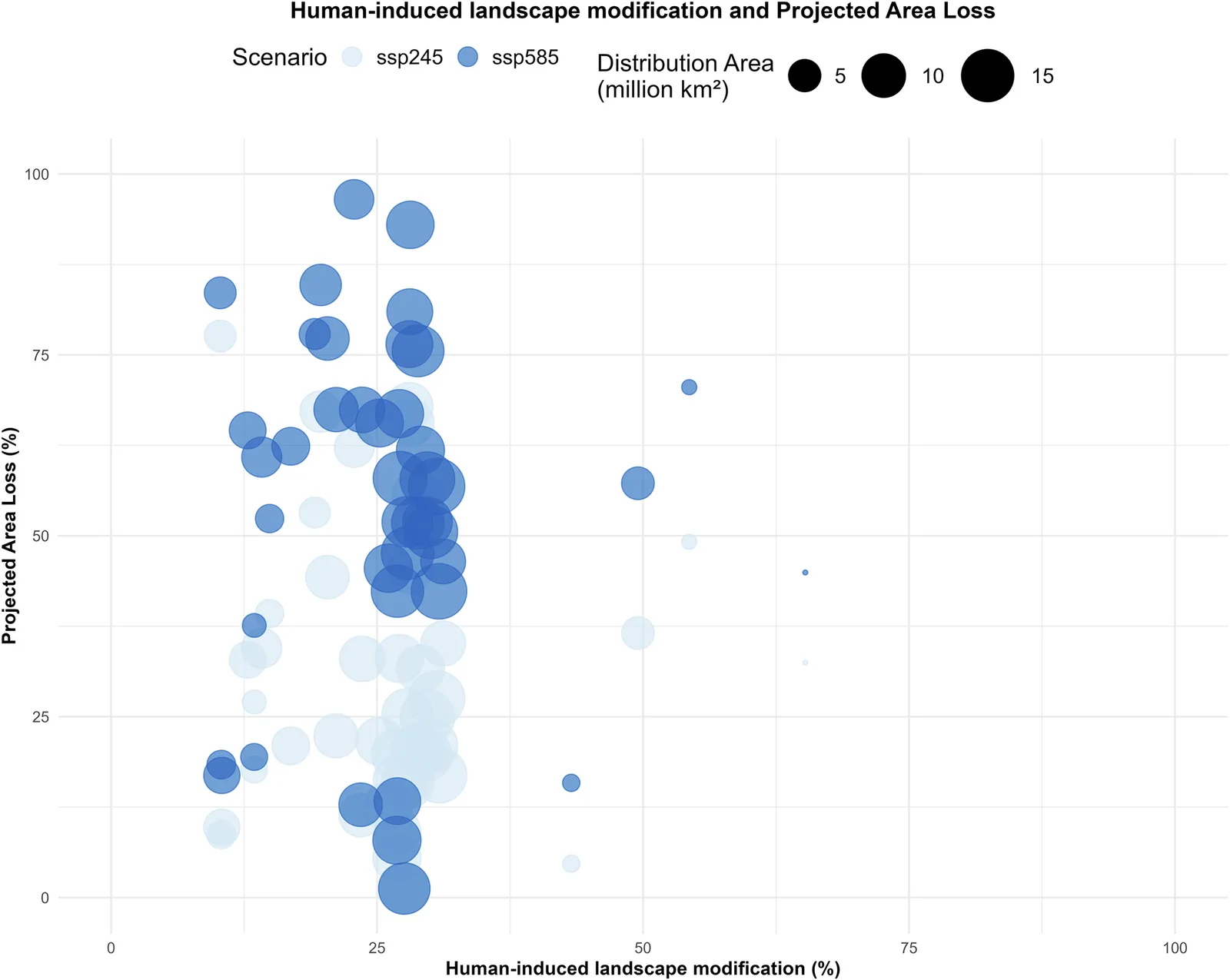
Human-induced landscape modification, projected habitat loss, and distribution range of Neotropical bat species complexes. Scatterplot showing the relationship between human-induced landscape modification (x-axis, %) and projected area loss (y-axis, %) under two climate scenarios (SSP245 in light blue and SSP585 in dark blue). Circle size represents the geographic distribution area (million km^2^) of each species complex. Larger circles indicate species complexes with wider distribution ranges, while smaller circles represent more geographically restricted taxa.

Geographic range sizes varied considerably among priority complexes, from comparatively restricted distributions (e.g., *Monophyllus redmani*: 186,742 km^2^) to widespread distributions (e.g., *Neoeptesicus furinalis*: 17,925.685 km^2^). Despite this variation, species vulnerability resulting from loss of suitable habitat due to climate change or from impacts resulting from human-driven landscape modifications does not strictly correlate with range size (Fig. 6). Under the severe SSP585 climate scenario, species complexes with smaller ranges, such as *Anoura luismanueli* and *Hylonycteris underwoodi*, are expected to experience substantial reduction of suitable habitat (44.93% and 70.54%, respectively). Nevertheless, widespread complexes, like *Myotis riparius*, will also potentially face significant losses in suitable habitat (75.5%). Some complexes with narrow ranges are further subjected to human-induced landscape modifications, such as *Monophyllus redmani* (82.74%), exacerbating their vulnerability.

## Discussion

Here, we present a comprehensive prioritization for the description, research, and conservation of the hidden diversity in one of the most threatened^30^ and biodiverse^31–33^ regions of the world, the Neotropical realm. By analyzing a large taxonomic coverage of bat species and an extensive genetic dataset through a multi-disciplinary approach, we found marked hidden diversity among Neotropical bats, with at least 95 candidate species within 45 complexes, which means that approximately a one-fifth of currently recognized bat species may contain new taxa. Importantly, we showed that hidden diversity is not restricted to specific taxonomic groups but spreads throughout the phylogeny, encompassing taxa with distinct ecological and habitat preferences. From specialized nectarivores, insect-hunters, sanguivores, to generalist frugivores, hidden diversity permeates the entire evolutionary landscape of Neotropical bats.

In addition to identifying species complexes, we also uncovered 10 critical hotspots of hidden diversity. We recommend that universities, academic institutions, researchers, and funding bodies direct their efforts, time, and resources to these regions in order to address the Linnaean shortfall in this important mammalian group^7,8^. The identified hotspots are regions characterized by complex topography, historical climate stability, and high habitat heterogeneity. These features align with established biogeographic theory, which predicts higher speciation rates in areas with high environmental heterogeneity, including complex topography, diverse habitat types, and variable climatic conditions^34^, yet challenge previous mammalian studies that found no clear geographic pattern in hidden diversity^29^.

Our province-level analysis reveals that hidden bat diversity exhibits distinct local spatial signatures. We found extensive knowledge gaps across the Neotropics (Supplementary Fig. 88). Particularly, the southern cone of South America, including the Uruguayan Savanna, Andean highlands, desert areas of the western coast of South America, and portions of southern Mexico remain completely data-deficient. Based on patterns observed in adjacent well-sampled regions, these areas may harbor undocumented diversity, but the absence of genetic data currently prevents their inclusion in conservation planning. Limited sampling caused by insufficient funding, difficult access, social instability, and lack of institutional support perpetuates these knowledge gaps. Without genetic data to identify hidden species and evaluate their distributions and threats, effective conservation strategies cannot be formulated. In addition, several regions (Supplementary Fig. 88) have minimal genetic sampling, sufficient only to confirm the presence of described species but inadequate to detect hidden diversity. Vast areas of Paraguay (Chaco), Brazil (Cerrado, Caatinga, and northeastern Amazon), western Ecuador and Colombia (Choco), and Mesoamerican forest in Honduras and Guatemala remain genetically undersampled and underrepresented. These “genetic deserts,” particularly the Cerrado, Caatinga, and portions of the Amazon basin, closely mirror the areas highlighted by other authors using environmental complementarity analysis, as sites where potential new species may appear^35^. These undersampled regions emphasize the need for targeted field expeditions to complete the map of Neotropical bat diversity before human-driven landscape changes, including those resulting from climate change, render these knowledge gaps permanent. They also highlight the role of natural history collections in preserving the genetic resources awaiting to be studied.

We provide a list of species complexes harboring significant hidden diversity. Although regional conservation frameworks for Neotropical bats exist through national action plans or priority area analyses^35–38^, these initiatives are still limited by the Linnean shortfall and thus focus only on formally described species. In contrast, our study brings attention to hidden taxa and prioritizes undescribed lineages before they go extinct. Our integration of hidden diversity patterns with human-driven landscape changes and climate vulnerability metrics reveals that many of these undescribed lineages face immediate threats. Forty percent of the identified species complexes were classified as “Very High” or “High” priority. Our results show that several of these taxa, despite currently being listed as Least Concern under IUCN, harbor hidden diversity in regions projected to lose between 25 and 60% of their suitable habitat under both moderate (SSP245) and severe (SSP585) climate change scenarios, and only a few species complexes showed net range gains (~10% under SSP245, ~5% under SSP585; Supplementary Fig. 89). This is particularly concerning in the Amazon, Eastern plains, and Central forests in Mesoamerica, areas that face rapid landscape changes resulting from agricultural expansion, livestock production, mining, urban development, and transportation infrastructure^39^. These factors are recognized as major threats to global bat conservation^40,41^. In addition, climate change will not only influence the availability of suitable habitats but shifts in temperature and precipitation patterns may make the synchronization between bat reproductive cycles and food availability increasingly precarious, potentially affecting energy expenditure and survival rates^42^.

Notably, we found that potentially undescribed species are not restricted to pristine, remote habitats with little human intervention. Contrary to popular conception^43^, many undescribed lineages persist in anthropogenically modified landscapes, as evidenced by the significant overlap between high HSR values and elevated Human Modification Index scores. These patterns may reflect the ecological plasticity of some bat taxa, such as generalists relying on anthropogenic resources, such as crops, insects associated with livestock, or roosts in human-created habitat. Moderate habitat disturbance may also create edge habitat and resource heterogeneity that can benefit some bat guilds. Together, these findings emphasize that species discovery efforts should target both pristine wilderness and modified habitats, expanding our search for hidden diversity beyond traditional boundaries^44^.

Despite the continued effort to document bat diversity (approximately 300 new bat species were described in the last 20 years globally^23,24^), our study identified the presence of at least 95 molecularly delimited candidate lineages in the Neotropics alone. At the current rate of description for the Neotropics, formally recognizing this diversity would take decades, a time these putative species likely do not have due to accelerating habitat loss and climate change. Taxonomic expertise itself is a limited resource, with a critical imbalance between the vast number of species yet to be described and the number of trained taxonomists available to describe them^45,46^. We urge the taxonomic community to focus on those species’ complexes assigned to very high and high priority levels (Table 1) given their elevated risk of extinction. Targeted taxonomic efforts on these 18 complexes, using integrative approaches that include evidence from molecular, morphological, acoustic, and ecological data, would permit the formal recognition of dozens of species in a 10-year timeframe, potentially allowing incorporation into a conservation framework before local extinction occurs. Addressing the Linnaean shortfall requires dedicating time, logistics, and resources to the species complex and hotspots identified here, as well as employing integrative taxonomy to properly delimit and describe this hidden diversity. We explicitly caution readers that conservation actions stemming from our list should be accompanied by targeted, integrative taxonomic studies to confirm species boundaries.

Our findings also underscore the complex interplay between range size, climate change, and other human-driven environmental changes, emphasizing the need for conservation strategies that address both restricted and widespread species complexes. For instance, strategies for habitat protection must include both small critical areas for endemic lineages as well as landscape-scale connections for widespread species. Climate change mitigation needs to include both the preservation of local microrefugia protection for range-restricted taxa and the creation and maintenance of regional connectivity for mobile widespread complexes. Our study challenges the common assumption that widespread species are inherently more resilient to global change, as many complexes with extensive distributions exhibited significant vulnerability to habitat loss despite their breadth of occurrence^47^. This is even more evident when we consider that we are modeling species complexes that contain undescribed lineages with even smaller ranges. In general, this pattern aligns with emerging evidence that range size alone may be an inadequate predictor of extinction risk when species face rapid environmental shifts^47^, particularly for taxa with specific ecological requirements or limited adaptive capacity^48^.

Our approach, particularly our HSR index and integration of multiple threat layers, offers a template for similar assessments across other taxonomic groups and geographic regions. By integrating molecular data with spatial analyses and niche modelling, we provide a transferable roadmap for uncovering potential hidden biodiversity in diverse ecosystems worldwide. Nevertheless, our study faced some limitations. First, while our accessibility index captures geographic constraints to sampling (distance to roads, cities, and protected areas), we acknowledge that other factors, such as political instability, funding limitations, or safety concerns, may prevent sampling in otherwise accessible areas. However, our approach provides a standardized, large-scale approximation of sampling likelihood that can be consistently applied across the entire Neotropical realm. Second, although our HSR index shows moderate correlation with sampling intensity (Spearman *ρ* = 0.584, *p* < 0.01), this relationship is expected: taxonomists have historically concentrated fieldwork in regions with high described species richness, where research funding is more readily justified. Nevertheless, the HSR framework successfully identifies critical deviations from sampling patterns, demonstrating its potential for revealing overlooked biodiversity hotspots. Third, our analyses were performed using a single mitochondrial marker (COI), which is abundant in public repositories but inevitably introduces some uncertainty into species delimitation^49–51^. While our conservative approach relied on agreement among multiple delimitation algorithms, using nuclear markers or whole-genome data would offer greater resolution. However, this would restrict our continental assessment due to limited available data. Nevertheless, when comparing our phylogenies with published papers using multiple genes, we obtained similar results in most cases, suggesting that our single-gene-dependent phylogenies did not significantly affect our main findings. Fourth, the geographic coverage of available COI sequences is highly heterogeneous across the Neotropical realm. Well-sampled provinces are more likely to yield detectable candidate lineages, while poorly sampled regions yield no detectable signal due to insufficient data rather than true biological absence.

Finally, it is also important to acknowledge that we modeled species complexes rather than individual hidden lineages, as the distributional limits of each hidden species cannot yet be definitively determined. This approach potentially masks differential habitat requirements and climate responses among the undescribed lineages within each complex. Despite these constraints, our ecological niche modeling approach remains methodologically robust for investigating hidden diversity patterns. The conservative parameterization of our models, rigorous algorithm selection, and thorough cross-validation significantly minimize potential overfitting issues. Furthermore, the consistent patterns we observed across multiple species complexes and climate scenarios suggest that our findings represent genuine biological signals rather than methodological artifacts. Most critically, our conservation prioritization framework was explicitly designed to be robust to uncertainties in any single component. Climate projections contribute only 25% to the Unified Score, with the remaining 75% derived from current data (IUCN status, human modification, and range size) that involve no future extrapolation.

The race against extinction to document Earth’s biodiversity has never been more urgent. Our study reveals that, for Neotropical bats, one of the most studied groups of mammals^23,24^, the number of currently recognized species (435 species^52^) considerably underestimates the true bat diversity, and that numerous lineages may be facing extinction before being formally recognized. Beyond its taxonomic implications, this work highlights that the diversity of undescribed Neotropical bats is concentrated in threatened regions that require urgent scientific attention and conservation action.

While our study focuses on bats, the trends and issues we identify have broad implications for biodiversity conservation globally. Hidden diversity is not unique to bats, or the Neotropics; undescribed species complexes are increasingly identified across a multitude of taxonomic groups and biogeographic regions, from Southeast Asian amphibians to small mammals in Africa, and invertebrates across the globe^53^. The taxonomic challenges that we report for Neotropical bats are consistent with patterns that emerge from other hotspots of bat diversity in the tropics. Recent phylogeographic studies with Asian chiropterans have identified comparable levels of hidden diversity, especially in morphologically conservative groups, such as Hipposideridae and Rhinolophidae, where 20–40% of currently recognized species contained lineages yet to be formally described^54,55^. Similarly, Afrotropical bat assemblages show extensive hidden diversity^56,57^ in areas with rapid land-use conversions. These convergent patterns across bat faunas demonstrate that the race to document hidden diversity before extinction is a global imperative for its conservation. Our results highlight the urgent need for international collaboration to accelerate taxonomic research, prioritizing biodiversity hotspots where institutional capacity and funding are limited. Addressing hidden diversity is not only an academic exercise in the field of systematic biology, but it is also essential to understanding the true scale of the current extinction crisis and to implementing conservation strategies that consider the undocumented biodiversity worldwide.

## Methods

### Hidden species identification

#### Genetic data acquisition and processing

**Fig. 7:**
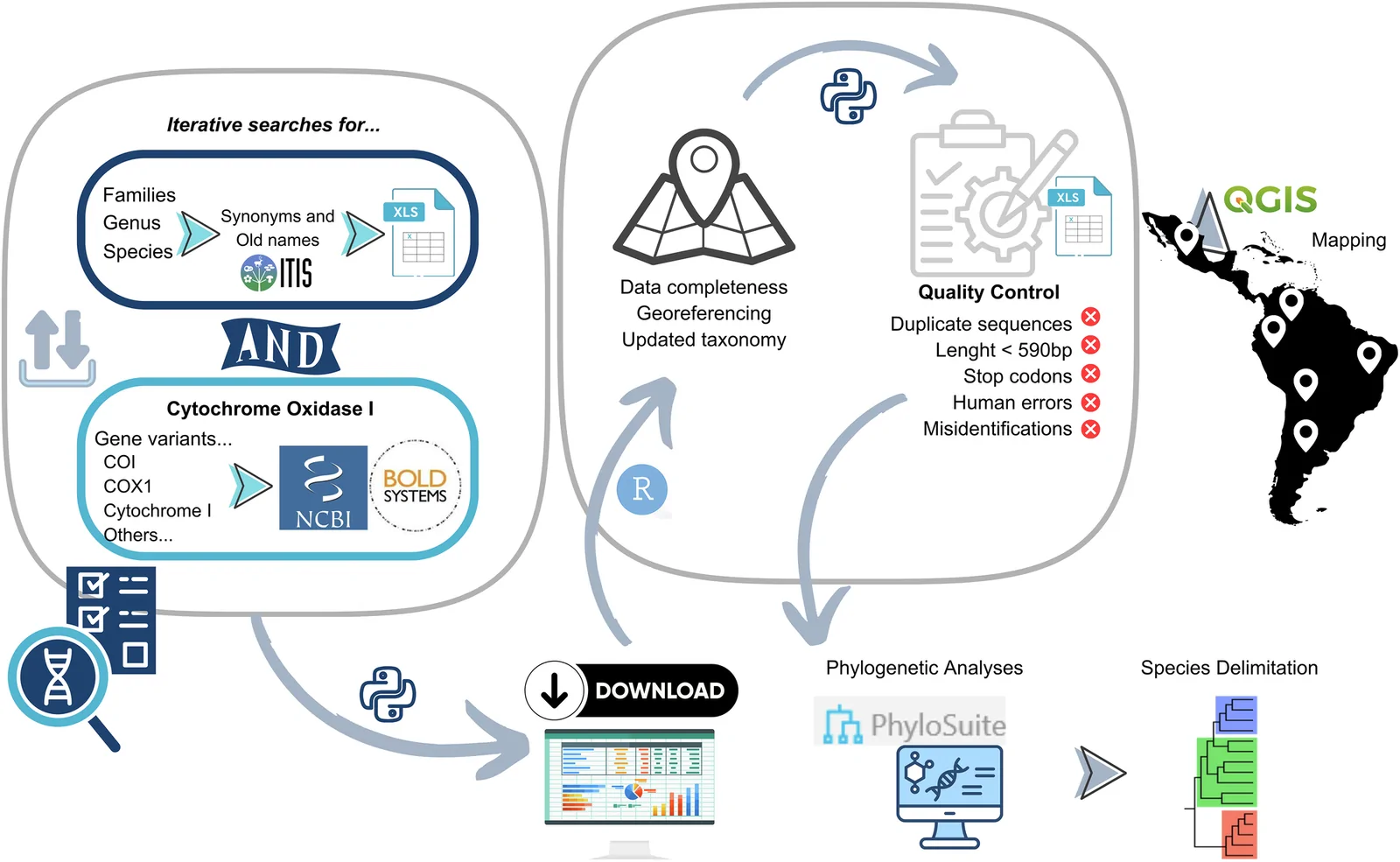
Pipeline used to obtain and clean genetic data. The workflow includes: (1) data acquisition from GenBank and BOLD using iterative searches; (2) data processing including data completeness, georeferencing, and taxonomic updates; (3) quality control filtering to remove artifacts; and (4) mapping and data preparation for phylogenetic analyses.

We compiled a comprehensive dataset of mitochondrial DNA sequences for Neotropical bats by extracting data from GenBank and BoldSystems using custom Python scripts. For bat genera with a Panamerican continental distribution extending beyond the Neotropics, we only considered species whose distribution covers at least 70% of the Neotropics, considering their occurrence within Morrone´s biogeographic provinces^58^. We retrieved sequences of the mitochondrial Cytochrome Oxidase I (COI) gene, along with associated metadata, such as accession numbers, taxonomy, geographic coordinates, and specimen voucher information. We selected the COI gene as our molecular marker because of its established role as a universal DNA barcode for animal species identification and its useful evolutionary rate for species-level discrimination in bats^25,26,51^. In some cases, COI alone may not provide sufficient resolution to distinguish closely related taxa, requiring additional molecular markers, such as nuclear genes or whole-genome sequencing. However, in these instances, our approach is more likely to result in an underestimation rather than an overestimation of cryptic taxa. Additionally, COI’s extensive representation in public databases makes it particularly valuable for broad-scale taxonomic studies, enabling comprehensive coverage across Neotropical bat families. Our data acquisition workflow involved (1) *Iterative database searches*. Using Boolean operators, we conducted searches at the family, genus, and species levels. This approach ensured exhaustive coverage by querying both current and outdated nomenclature, referencing the ITIS database for synonyms; (2) *Data completion*. Missing information (e.g., coordinates or voucher details) was retrieved from related publications or by contacting authors; (3) *Taxonomic updates*. We updated nomenclature following the Mammal Diversity Database^52^ and the batnames database^22^. (4) *Quality control*. We removed duplicate sequences, as well as those shorter than 90% of the typical gene length (COI < 590 bp). We also conducted a preliminary alignment for each dataset using MEGA X^59^ with default parameters. During this process, we identified and excluded sequences with nonsense mutations, stop codons, insertions, and deletions, as these anomalies were likely results of low-quality sequencing, erroneous amplification, laboratory contamination, or other errors. We tested for substitution saturation using DAMBE, following Xia^60^. In all cases, Iss was significantly lower than Iss.c (*p* < 0.05), indicating that the data are not saturated and are appropriate for phylogenetic inference. We also corroborated the taxonomic identification of each sequence through phylogenetic trees and using BLAST. These quality control filters resulted in the exclusion of approximately 12% of initially retrieved sequences, primarily due to length requirements and the removal of sequences with stop codons or indels. While this filtering reduced our overall dataset size, it ensured high-quality phylogenetic analyses and did not inflate the geographic gaps. All Python scripts are available at https://github.com/diegodaem/HSR-Index.git, and our pipeline is described in Fig. 7. The filtered and cleaned dataset used in this study is presented in the Supplementary Data 1. Filtered sequences used for each species complex are presented in Supplementary Data 2.

#### Genus-level phylogenies

Phylogenies were constructed for each genus separately to ensure analytical stability and the robustness of species delimitation algorithms. Our pilot analyses revealed that broader taxonomic scales (e.g., tribes or families) often lead to algorithmic instability and nonsensical results. Genus-level analyses increase sample density and focus by analyzing species that are more similar in terms of evolutionary rates, which is better for monolocus species delimitation methods, improving precision and efficiency. After downloading and cleaning the genetic sequences, we aligned them using MAFFT v.7.505^61^ with the “–auto” strategy, “–normal” alignment mode, and “–adjustdirectionaccurately” parameter. Gap sites were removed with trimAl v.1.2^62^ using “–automated” command. We used ModelFinder v.2.2^63^ to select the best partitioning schemes and evolutionary models for partitions identified for each genus using the BIC criterion. We constructed maximum-likelihood phylogenies using IQ-TREE v.2.4^64^ under predefined models for each genus. We conducted ML analyses with 10,000 ultrafast bootstraps tests^65^ and 5000 replications of the Shimodaira–Hasegawa-like approximate likelihood ratio test. For the Generalized Mixed Yule Coalescent analysis (see below), we built an ultrametric tree using BEAUti2.7/BEAST v2.7.6^66^ with the same parameters and substitution models of the previous analyses. We assumed a strict clock model and a coalescent tree prior with constant population size. The strict clock model was selected because it assumes a constant substitution rate across lineages, aligning with the relatively short evolutionary timescales and shared histories within genera. This approach reduces overparameterization risks, improves convergence in Bayesian analyses, and is supported by empirical studies showing its reliability for closely related taxa with minimal rate heterogeneity^67^. We performed two independent runs for each dataset with 50 million generations, sampling the parameters every 10,000 generations. We used Tracer v.1.7.2^68^ to assess the statistical convergence of MCMC chains (stationary) by ensuring that the average standard deviation of split frequencies (ASDSF) was <0.01, the estimated sample size (ESS) was > 200, and the potential scale reduction factor (PSRF) approached 1. When these parameters were not met, we ran our analysis again. The resulting trees were combined using LogCombiner, and the Maximum Clade Credibility tree was generated in TreeAnnotator v.2.6.3^66^ after removing 25% as burn-in. We visualized and edited the trees using FigTree v1.4.4 (http://tree.bio.ed.ac.uk/software/figtree/).

#### Species complex delimitation

To identify and delineate candidate hidden complex species, we employed a combination of five DNA-based single-locus species delimitation methods, encompassing both distance and phylogenetic tree approaches. The methods included: (a) Automatic Barcode Gap Discovery (ABGD)^69^, (b) Assemble Species by Automatic Partitioning (ASAP)^70^, (c) the Generalized Mixed Yule Coalescent (GMYC)^71^, (d) the Bayesian Poisson Tree Processes model (bPTP)^72^, and (e) the Multi-rate Poisson Tree Processes model (mPTP)^73^. We conducted these analyses via respective web servers: the ABGD platform (https://bioinfo.mnhn.fr/abi/public/abgd/abgdweb.html) with settings for Simple Distance (p-distances) and a relative gap width of 1.5; the ASAP tool (https://bioinfo.mnhn.fr/abi/public/asap/) using Simple Distance; and the Exelixis Lab’s servers for both bPTP (http://species.h-its.org/ptp/) with an unrooted tree, 500,000 MCMC generations, and a 10% burn-in, and GMYC set to the “single threshold” method. For mPTP, we performed two independent MCMC runs of 1,000,000 generations each, sampling every 1000 generations with 10% burn-in in the Web Server (https://mcmc-mptp.h-its.org/). For the distance-based methods, uncorrected p-distances have been found to provide results that are as accurate, if not more so, than those derived from other models of nucleotide evolution, such as the K2P model^74^. We used a conservative approach where hidden species complexes were defined when three or more algorithms identified the same lineages as a hidden species.

### Hotspots for hidden species discovery and conservation

#### Mapping hidden species complex

Once the hidden complex species were identified, we mapped and overlaid their occurrences to pinpoint regions with a high concentration of hidden species. We plotted and analyzed them using the biogeographic provinces according to Morrone et al.^58^ for the Neotropical realm. These provinces are defined based on biotic and abiotic factors, including climate, topography, geological history, and endemic taxa distributions, incorporating distinct ecological and evolutionary processes that define species distributions. The provinces are widely recognized for their ability to capture the complexity of biogeographic patterns, making them particularly suitable for our study. To account for sampling biases inherent to historical, geographical, and social processes in which genetic sequences have been generated, we implemented a statistical approach. First, we plotted all available COI genetic sequences for Neotropical bats by biogeographic province. This provided a comprehensive visualization of the geographic coverage of genetic data and highlighted areas with potential under-sampling. Second, to mitigate the influence of uneven sampling effort, and considering the size of the biogeographic province, as well as its accessibility may influence this sampling bias, we develop the HSR index for each biogeographic province using the Eq. 1:

1 $${HSR}=\frac{{S}_{{hidden}}}{{{\mathrm{log}}}\left({N}_{{sequences}}+1\right)}x\,(1+{w}_{1}{Z}_{{accessibility}}+{w}_{2}{Z}_{{size}})$$

where *HSR* is the hidden species richness (HSR) index, ${S}_{{hidden}}$ is the estimated number of hidden species, ${N}_{{sequences}}$ is the total number of sequences, ${Z}_{{accessibility}}$ is the Z-score for accessibility (provinces with lower accessibility get higher weights), ${Z}_{{size}}$ is the Z-score for size (largest provinces get higher weights), and ${w}_{1}$ and ${w}_{2}$ are weights that determine the relative contribution of accessibility and size. These values can be empirically adjusted based on sensitivity analyses or calibration with real data.

To calculate the Z-scores for accessibility, we measured the distance from each genetic sampling coordinate (genetic sequences) to three key features: (i) access routes (main roads), based on the dataset of Meijer et al.^75^, (ii) major urban centers (population >10,000; based on the dataset of geonames.org), and (iii) protected areas (based on the World Database on Protected Areas^76^). From each sampling point *j* in province *i*, the minimum geographic distance to a reference feature was calculated using the Haversine formula, which accounts for the Earth’s curvature, following the Eq. 2:

2 $${d}_{{ij}}^{min }=\min \left\{2{R\; X} \arcsin \left(\sqrt{{sin }^{2}}\,\left(\frac{{{\varnothing }}2-\,{{\varnothing }}1}{2}\right) \right. \right.\\ \left. \left.+\cos \left({{{\varnothing }}}_{1}\right)\cos \left({{{\varnothing }}}_{2}\right){sin }^{2}\left(\frac{\gamma 2-\gamma 1}{2}\right)\right)\right\}$$

where *R* is the Earth´s radius (~6371 km), $(\varnothing 1,\,\gamma 1)$ are the coordinates of sampling point *j*, and $(\varnothing 2,\,\gamma 2)$ are the coordinates of each reference feature (road, city, or protected area).

For each province *i* with *n* points, we calculated the median minimum distance across all genetic sampling points to each reference feature, following the Eq. 3:

3 $$\widetilde{d}i,{road}={median}\,\left({d}_{{ij},{road}}^{\min }\right);\widetilde{d}i,{city}={median}\left({d}_{{ij},{city}}^{\min }\right);\\ \ \ \ \widetilde{d}i,{pa}={median}({d}_{{ij},{pa}}^{\min })$$

where ${d}_{i,{road}}$ is the median minimum distance to roads, ${d}_{i,{city}}$ is the median minimum distance to cities, ${d}_{i,{pa}}$ is the median minimum distance to protected areas.

An accessibility index (A_*i*_) was then calculated for each province as the average of these median distances, following the Eq. 4:

4 $${A}_{i}=\frac{\widetilde{{di}}\,{road}+\,\widetilde{{di}}\,{city}+\,\widetilde{{di}}\,{pa}}{3}$$

This metric represents the overall accessibility of genetic sampling locations within each province, where higher values indicate lower accessibility (i.e., greater distances to roads, cities, and protected areas), while lower values indicate higher accessibility (i.e., closer proximity to these features). Since accessibility and province size also vary significantly between provinces, we standardized them using median-based normalization with the median absolute deviation (MAD) method.

Then, for all *k* provinces, we computed:

The global median of accessibility following the Eq. 5:

5 $$\widetilde{A}{global}={median}({A}_{1},{A}_{2},\ldots,{A}_{k})$$

The MAD, following the Eq. 6:

6 $${{MAD}}_{{Accessibility}}={median}(|{A}_{i}-\tilde{A}{global})$$

And, the Z-score for accessibility as Eq. 7:

7 $${Z}_{{accessibility}}=\frac{{A}_{i}-\tilde{A}{global}}{{{MAD}}_{{accesibility}}}$$

To calculate the Z-scores for size, we considered the total area of each biogeographic province in square kilometers. It followed the same standardization process as the Z-score for accessibility:

To ensure comparability across provinces, we also computed:

The global median of province size, as Eq. 8:

8 $$\widetilde{S}{global}={median}({S}_{1},{S}_{2},\ldots,{S}_{k})$$

The MAD for province size, as Eq. 9:

9 $${{MAD}}_{Size}=median(|{S}_{i}-\tilde{S}global)$$

Z-score for province size, as Eq. 10:

10 $${Z}_{{size}}=\frac{{S}_{i}-\tilde{S}{global}}{{{MAD}}_{{size}}}$$

Finally, we determined the optimal parameter values for the HSR index, performing a sensitivity analysis where we tested over 80 different combinations of weights (*w*₁ and *w*₂) ranging from 0.05 to 5. We consider that this metric enables a more equitable comparison of HSR across regions by controlling for three major sources of sampling bias: uneven sampling effort (through logarithmic normalization), geographic accessibility (through accessibility weighting), and province size effects (through area normalization). HSR values were mapped at the province level to visualize patterns of HSR. A more detailed explanation of the formula is presented in Supplementary Text 1. All scripts are available at https://github.com/diegodaem/HSR-Index.git.

#### Current anthropogenic threats

To assess anthropogenic pressure in areas with hidden species, we used the Global Human Modification dataset (gHM)^39^, which provides a comprehensive measurement of human-induced landscape modification other than that potentially resulting directly from climate change. This dataset primarily captures landscape-scale habitat modifications integrating multiple anthropogenic pressures, such as urban development, agricultural expansion, livestock production, resource extraction, transportation infrastructure, and other human activities known to alter natural habitats or to otherwise lead to changes in survival, natality, and mortality rates of bat populations. The gHM values range from 0 (no modification) to 1 (complete modification). We overlaid the gHM layer with our HSR map to identify critical areas where high concentrations of hidden species coincide with severe habitat modification.

#### Climate change vulnerability

Using ecological niche models (ENMs), we predicted the potential distribution of identified hidden complex species under both current and future climate scenarios, applying the following workflow: (1) *Data preparation and spatial thinning*: We collected occurrence data representing the total distribution of each hidden species complex from databases, such as Arctos, GBIF (Global Biodiversity Information Facility), SpeciesLink (Brazil), SIB Colombia, VertNet, iNaturalist, and different museum collections including the Field Museum of Natural History (Chicago, USA), American Museum of Natural History (New York, USA), and National Museum of Natural History (Washington D.C., USA). We used data from each “complex” and not from each “hidden species” due to the undefined geographical boundaries of each hidden lineage. By modeling these complexes, we assumed shared climatic requirements among hidden and recognized species. Even when using generic variables for these complexes, we observed a significant reduction in suitable habitat. To ensure data quality, we removed inaccurate records (inconsistent species names, missing dates, missing coordinate values, and highly uncertain coordinates) and applied spatial thinning using a minimum distance of 5 km to reduce autocorrelation and minimize overrepresentation of well-sampled areas. Spatial thinning was conducted using the *spThin* v.0.2 package in R with 100 iterations. (2) *Environmental variables selection and calibration area*: We obtained bioclimatic variables from the WorldClim database for current conditions and future climate projections under ssp245 and ssp585 for the year 2070 at a resolution of 2.5 min^77^. We also considered IPCC6 climate projections for three general circulation models (GCMs): BCC-CSM2-MR, MRI-ESM2-0, and UKESM1-0-LL. We selected these models using the R package *chooseGCM* v.1.3^78^, which allows for choosing the best GCMs based on their performance across different climate projections for the study area (Supplementary Figs. 90 and 91). After that, we established a calibration area representing the hypothesized accessible area [M] for each species complex, defined as a 200 km buffer around occurrence points^79^. We then masked bioclimatic variables for this calibration area before conducting multicollinearity tests. We selected non-correlated variables using Spearman’s correlation coefficients, retaining only those with values <0.8 (Supplementary Table 4). We chose this threshold to achieve an optimal balance between multicollinearity and biological signal, allowing us to control extreme multicollinearity while preserving variables that can capture different aspects of the ecological requirements of hidden species. Likewise, we chose variables with high ecological relevance for each modeled species complex, based on their biological importance, considering critical factors, such as thermal limits, precipitation patterns, and seasonality that directly influence the physiology, phenology, and distribution of the species complex. (3) *Model calibration and validation*: We generated ecological niche models using the R package *kuenm*^80^ and the Maxent software v.3.4.4^81^. We selected MaxEnt given its robust performance with presence-only data and its proven reliability across diverse taxa and regions. A single well-tuned algorithm ensures consistent and comparable predictions across our 45 species complexes, whereas other methods can introduce variability that complicates cross-taxa comparisons at continental scales. To optimize model performance, we explored multiple combinations of regularization multipliers, feature classes, and variable subsets. We evaluated all candidate models and selected the best models based on statistical significance (partial ROC), performance (omission rate), and model complexity (AICc). We used omission rate as our performance metric because it minimizes false negatives, which is critical when modeling potentially threatened species complexes and more reliable when working with presence-only data^82^. (4) *Habitat suitability mapping*: We generated logistic output maps to display continuous habitat suitability for each hidden species complex. Using threshold values derived from omission rates at 5% (*E* = 5%), we converted these maps into binary outputs (suitable and unsuitable areas). For each scenario, we identified areas of loss of habitat suitability for species. Finally, we summarize these outputs across all hidden species complexes to highlight geographic regions experiencing significant changes in habitat suitability.

#### Integration of threat layers

To identify critical areas for hidden bat species (hereinafter hotspots), we integrated three key analytical layers: (1) the HSR index, representing the spatial patterns of undescribed bat diversity; (2) the Global Human Modification index (gHM), indicating current anthropogenic pressures; and (3) climate change vulnerability derived from ecological niche models, showing projected habitat loss in 2070. We overlaid these layers using a weighted approach where each cell was classified based on the combined intensity of these three components. Areas were categorized as hotspots when they exhibited concurrent high values across all three dimensions. This integration highlighted Neotropical regions where high concentrations of undescribed bat diversity coincide with both immediate anthropogenic threats and future climate vulnerabilities, identifying priority areas that demand urgent attention for both species’ discovery efforts and conservation planning.

#### Priority species complex identification

In addition to describing hotspots for hidden bat species, we also identified and listed species complexes to draw the attention of researchers and stakeholders, focusing on groups that urgently demand research efforts, economic resources, and conservation action. To achieve this, we employed an evaluation based on four criteria that consider current threats and future vulnerabilities. Each criterion was selected to capture different aspects of conservation urgency: (1) Conservation status of the entities currently recognized as species but which in our analysis present hidden diversity by IUCN, reflecting current extinction risk using IUCN criteria^21^; where approximately 17% are listed as DD or Not Evaluated; (2) Future Projected Suitable Habitat Loss in percentage, as indicated by ecological niche models, reflecting future climatic vulnerability; (3) Extent of Occurrence (EOO) as proxy of geographic range size, showing narrow or wide geographic ranges; and (4) Human Impact Exposure, overlapping hidden complexes’ distribution with high human-impact areas, emphasizing regions where taxa are exposed to anthropogenic pressures as described by Theobald et al.^39^ (Supplementary Text 2 describes how we calculate the EOO and the percentage of human-induced landscape modification in it for each complex; see Supplementary Figs. 92–136 for EOO maps to each species complex). These criteria were integrated into a weighted scoring matrix, with weights assigned: IUCN status (25%), Future Projected Suitable Habitat Loss (25%), Human Impact Exposure (25%), and Geographic Range Size (25%). The weighting scheme was inspired by methodologies in conservation prioritization that emphasize the differential impacts of these factors on species persistence^83–85^. Each criterion was evaluated using a standardized scoring system, where higher scores reflect greater conservation concern. For instance, IUCN categories ranged from critically endangered (CR, 7 points) to Not Evaluated (NE, 1 point), while other criteria used a 5-point scale based on criteria established by the IUCN and ecologically meaningful thresholds (e.g., >80% habitat loss = 5 points; see Supplementary Figs. 137–141 for details about the scoring system). Similar approaches have been successfully applied in spatial conservation assessments to identify priority species and areas^86,87^. The weighted scores were combined to calculate a final priority index (Unified Score), categorizing species complexes into five categories: Very High, High, Medium, Low and Very Low priority. Ties in Unified Score were resolved considering the Human Impact Exposure (descending) and then by Extent of Occurrence (ascending), prioritizing taxa under immediate anthropogenic pressure and with more restricted ranges. All scripts are available at https://github.com/diegodaem/HSR-Index.git.

### Reporting summary

Further information on research design is available in the Nature Portfolio Reporting Summary linked to this article.

## Supplementary information

Supplementary Information

Description of Additional Supplementary Information

Supplementary Data 1

Supplementary Data 2

Reporting Summary

Transparent Peer Review File

## Data Availability

All data generated and analysed in this study are provided in the Supplementary Information and are available in the GitHub repository [https://github.com/diegodaem/] and archived on Zenodo^88^. Data sources and accession information for all genetic sequences analysed in this study are provided in Supplementary Data 1.

## Code availability

Data and code supporting the findings of this study are provided on the public GitHub repository (https://github.com/diegodaem/) and are archived on Zenodo^88^. It is also possible to request them from the corresponding author.
