# Supplementary material for: Substantial Neotropical bat diversity likely remains undescribed and vulnerable to extinction
Source: Nat Commun. 2026 Nov 3;17:11368. doi: 10.1038/s41467-026-78393-0 (PMC13731659; doi:10.1038/s41467-026-78393-0)
Supplement: Supplementary file 1 [file 41467_2026_78393_MOESM1_ESM.pdf]

Supplementary Information for

Substantial Neotropical bat diversity likely remains undescribed and vulnerable to extinction

Diego A. Esquivel*, Angie P. Penagos, Anderson Feijó, Maria João Ramos Pereira

*Corresponding author. Email: diegodaem@gmail.com

This PDF file includes:

Supplementary Text 1 to 2
Supplementary Figs. 1 to 141
Supplementary Tables 1 to 4

Supplementary Text 1: Rationale and explanation of the Hidden Species Richness (HSR) index

Overview

The Hidden Species Richness (HSR) index was developed to address a fundamental challenge in biodiversity assessment: how to estimate the true richness of undescribed lineages while accounting for the inherent biases in genetic sampling efforts. This index provides a standardized metric that allows comparisons of hidden diversity across biogeographic provinces with different sampling intensities, accessibility, and spatial extents.

The HSR

$$HSR = \frac{S_{hidden}}{\log(N_{sequences} + 1)} \, x \, (1 + w_1 Z_{accessibility} + w_2 Z_{size})$$

1. Numerator: S_{hidden} (Estimated number of hidden species)

This represents the raw count of potential undescribed lineages identified through molecular delimitation analyses for a given region.

2. Denominator: $\log(N_{sequences} + 1)$

Genetic sampling effort varies dramatically across regions due to multiple factors, as for example proximity to research institutions, cities or universities, historical collecting expeditions, logistical accessibility, and funding availability. So, the logarithmic transformation serves to control the bias produced by unequal number of sequences between regions.

3. Correction Factor: $(1 + w_1 Z_{accessibility} + w_2 Z_{size})$

This multiplicative correction addresses two major sources of sampling bias:

A) Accessibility ($w_1 Z_{accessibility}$), where remote, inaccessible areas are systematically under-sampled in biodiversity studies, generating a "accessibility bias" where hidden diversity in these regions appears artificially low. Our accessibility metric considers distances to roads, cities and protected areas. Provinces with poor accessibility (high positive Z-scores) receive an upward correction, acknowledging that their low sequence counts likely reflect logistical challenges rather than low diversity. This is biologically justified because many hidden species may be endemic to isolated regions, and also by historical sampling has favored accessible locations.

B) Size ($w_2 Z_{size}$), where we assume that larger provinces have more species based in the species-area relationship as fundamental ecological principle, larger areas typically support more species. This explains why larger areas generally have greater habitat heterogeneity, more ecological niches, higher probability of geographic barriers promoting speciation and greater potential for sampling gaps. So, our size correction ensures that extensive provinces (e.g., Cerrado or Amazonian regions) are not penalized for having proportionally fewer sequences per unit area.

4. Weight Calibration: (w_1 and w_2)
The weights are empirically determined through sensitivity analysis to balance conservation priority (ensuring remote and threatened areas are not overlooked), biological realism (reflecting known patterns of bat diversity) and statistical robustness (maintaining index stability across extreme values). These values must be calculated for each distinct dataset and are not universal.
Example application

Please note that the values of $\boldsymbol{w_1}$ *and* $\boldsymbol{w_2}$ *must be calculated from your own dataset using sensitivity analyses (scripts are available). For illustration, we use the same values here, assuming they were estimated for the following examples:*

Example 1

Province A (accessible areas with cities, protected areas, museums and universities nearby)
Hidden species: 5
Sequences: 3,000
Highly accessible (Z_accessibility = -1.5)
Medium size (Z_size = 0)
w_1= 0.15
w_2 = 0.10

Calculation:
Basic ratio: 5/log (3,001) = 5/8.01 = 0.62
Correction: (1 + 0.15*(-1.5) + 0.10*(0)) = 0.775
HSR = 0.62 × 0.775 = **0.48**

Province B (Remote areas, far from cities, museums, protected areas and universities)
Hidden species: 5
Sequences: 30
Poorly accessible (Z_accessibility = +2.0)
Medium size (Z_size = 0)

Calculation:
Basic ratio: 5/log (31) = 5/3.43 = 1.46
Correction: (1 + 0.15*(2.0) + 0.10*(0)) = 1.30
HSR = 1.46 × 1.30 = **1.90**

Impact: Same hidden lineages count (5) and same province size, differences in accessibility. Province B scores **4× higher**, revealing it as the true priority for exploration. Its low sequence count reflects logistical barriers, not low diversity.

Example 2: The "Size Matters"

Province A – Small province, moderate accessibility
Hidden species: 10
Sequences: 500
Moderate access (Z_accessibility = 0)
Small size (Z_size = -2.0)

Calculation:
Basic ratio: 10/log (501) = 10/6.22 = 1.61
Correction: (1 + 0.15*(0) + 0.10*(-2.0)) = 0.80
HSR = 1.61 × 0.80 = **1.29**

Province B Large province, moderate accessibility
Hidden species: 10
Sequences: 500
Moderate access (Z_accessibility = 0)
Large size (Z_size = +2.0)

Calculation:
Basic ratio: 10/log (501) = 10/6.22 = 1.61
Correction: (1 + 0.15*(0) + 0.10*(2.0)) = 1.20
HSR = 1.61 × 1.20 = **1.93**

Impact: Identical hidden lineages count (10), identical sequences (500), and identical accessibility, unique differences in size. Province B scores **1.5× higher**, demonstrating how the size correction prevents large provinces from being penalized for having proportionally fewer sequences per unit area.

Validation and limitations

The HSR index assumes that:

- Sampling effort shows some degree of correlation with sequence availability.
- Accessibility and size are primary drivers of sampling bias.
- Hidden diversity patterns follow similar biogeographic rules as described diversity.

Users should note that HSR provides relative, not absolute, estimates of hidden diversity and works best for comparative analyses across regions rather than precise species counts.

Implementation Guidelines

When applying HSR to other taxa or regions:

1. Ensure comprehensive sequence databases.
2. Use ecologically meaningful biogeographic units.
3. Adjust weights based on taxon-specific sampling patterns.
4. Consider additional bias sources relevant to focal organisms.

This index represents a transferable framework for identifying priority areas for taxonomic research and conservation planning in the face of incomplete biodiversity knowledge.

A script running is provided at https://github.com/diegodaem/HSR-Index. Inside, there are notes that guide the user through the entire process.

Supplementary Text 2: EOO Calculation and percentage of human footprint

To calculate species' EOO polygons, we downloaded all occurrence records from multiple databases including GBIF, Vertnet, SIB Colombia, and SpeciesLink. For species with reliable taxonomic identification, we also included records from iNaturalist, and we complement these with searches from literature (articles, notes). After removing duplicate records and cleaning the data, we generated the EOO polygons following IUCN guidelines. To assess the proportion of each species' range under high human pressure, we first obtained the terrestrial Extent of Occurrence (EOO) by intersecting the original EOO polygon with a Neotropical landmass shapefile, effectively removing marine areas. We then analyzed the overlap between this terrestrial EOO and areas of high human modification using the Human Footprint Index (HFI) from Theobald et al. (2020). The HFI raster was first reclassified to identify areas above the 70th percentile, representing regions under high human pressure. Due to computational limitations with large raster files, we processed the analysis in blocks. The raster was divided into 5x5 blocks (25 total), and for each block, we: (1) cropped both the HFI raster and the EOO polygon to the block extent, (2) masked the HFI raster with the EOO polygon, (3) calculated the area of high human pressure within each block using cell-by-cell analysis, and (4) accumulated these areas across all blocks. The final proportion was calculated by dividing the total area under high human pressure by the total terrestrial EOO area. All spatial analyses were conducted using R version 4.0.0 with the “terra” and “sf” packages, maintaining the original projection (EPSG:4326) throughout the process to ensure accurate area calculations. Maps for each species showing the Terrestrial EOO, and Terrestrial EOO + High Human Pressure in km^2 with its proportion in percentage are displayed in **figs. 92-136**. **Note**: Green color represents the terrestrial Extent of Occurrence (EOO) of the species, while red color indicates areas within the EOO that are under high human pressure (above the 70th percentile of the Human Footprint Index). Scripts for all analyses are also available in main text.

Thyroptera tricolor

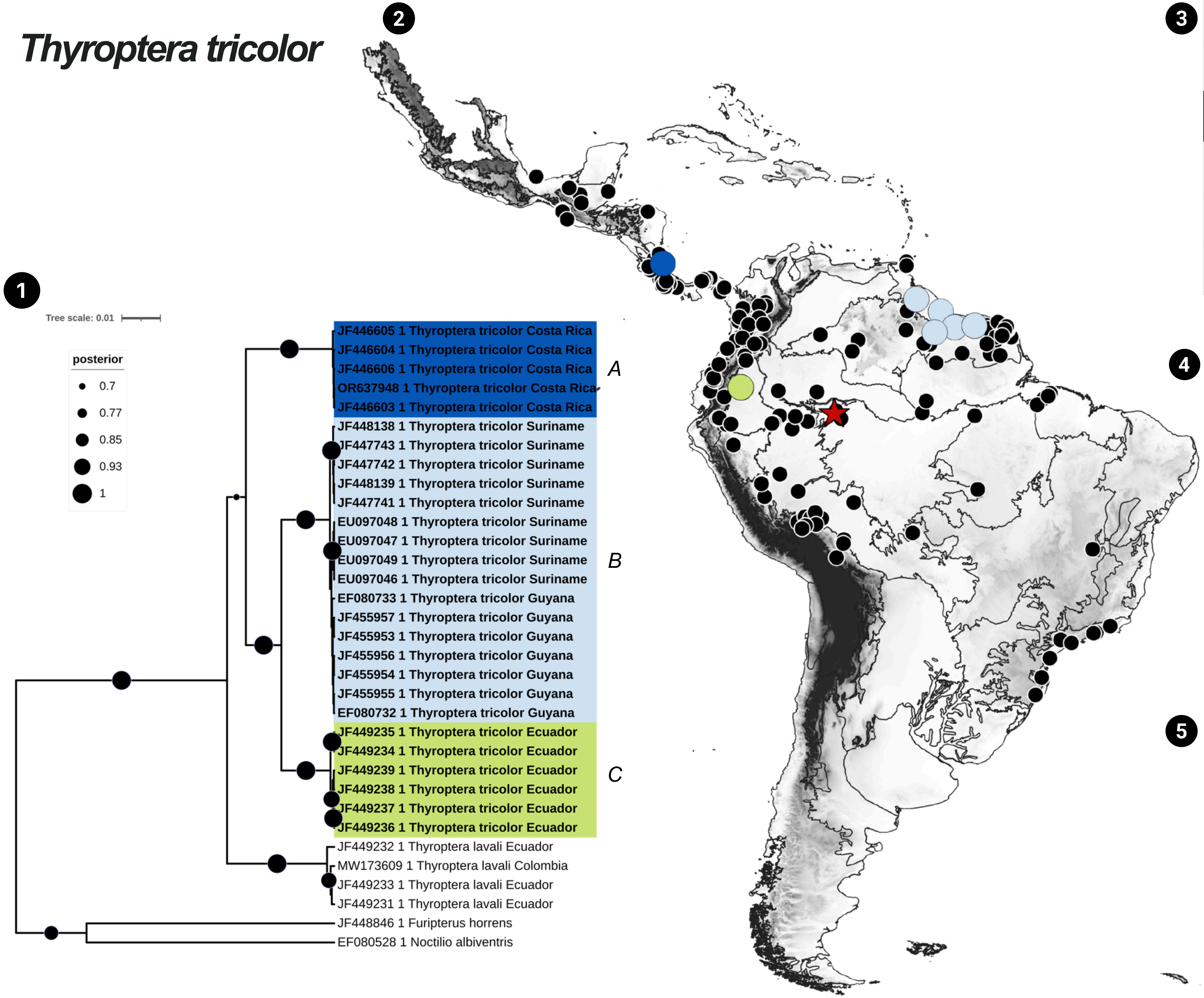

3

Mean genetic distances (Simple Distance)

Diagonal (red) = mean intraspecific distance | Off-diagonal (black) = mean interspecific distance | n = number of unique pairs

Especie	*T. tricolor* A	*T. tricolor* B	*T. tricolor* C	*T. lavali*
T. tricolor A	0.0000 (n=10)	0.1272 (n=80)	0.1277 (n=30)	0.1211 (n=20)
T. tricolor B	0.1272 (n=80)	0.0030 (n=120)	0.0861 (n=96)	0.1330 (n=64)
T. tricolor C	0.1277 (n=30)	0.0861 (n=96)	0.0033 (n=15)	0.1244 (n=24)

4

Max intraspecific: 0.0171 | Min interspecific: 0.0826 | Gap size: 0.0654

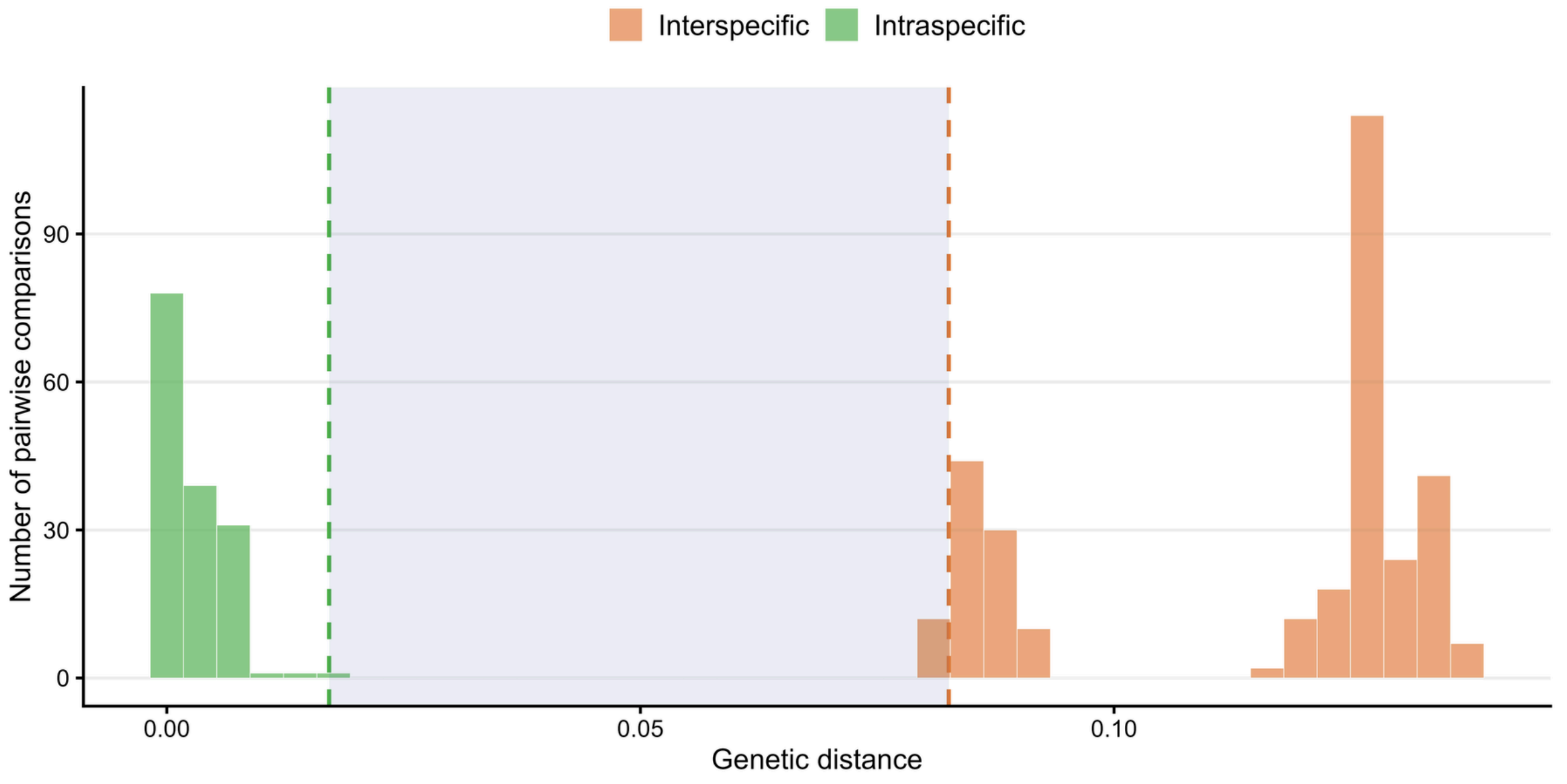

5 COI

Clare et al., (2011) PloS One

Figure 1

Fig. 1. Phylogenetic, geographic, and supporting evidence for candidate lineages in *Thyroptera tricolor.* (1) Phylogenetic hypothesis based on COI sequences, with colored clades representing candidate lineages identified through molecular species delimitation. (2) Geographic distribution map, where colored points correspond to the lineages recovered in the phylogeny and black points represent the overall known distribution of the species complex. (3) Table of genetic distances (when sufficient data are available), reporting intra- and inter-lineage divergence among delimited groups. (4) Histogram of genetic distances illustrating the barcode gap. (5) Phylogenetic hypotheses from previous studies (when available), provided for comparison with our proposed delimitations. (6) Additional lines of evidence (occasionally available), such as geometric morphometrics or other independent datasets supporting lineage differentiation. Red star indicates holotype.

Note: *When this symbol (⚠) appears next to the phylogeny, it indicates that the delimitation should be interpreted with caution, as additional sampling is needed. When it appears next to the table, it means that genetic distances are low; therefore, we recommend that these results also be considered with caution. In both cases, we have retained the delimitations as inferred by our methods.*

Furipterus horrens

1

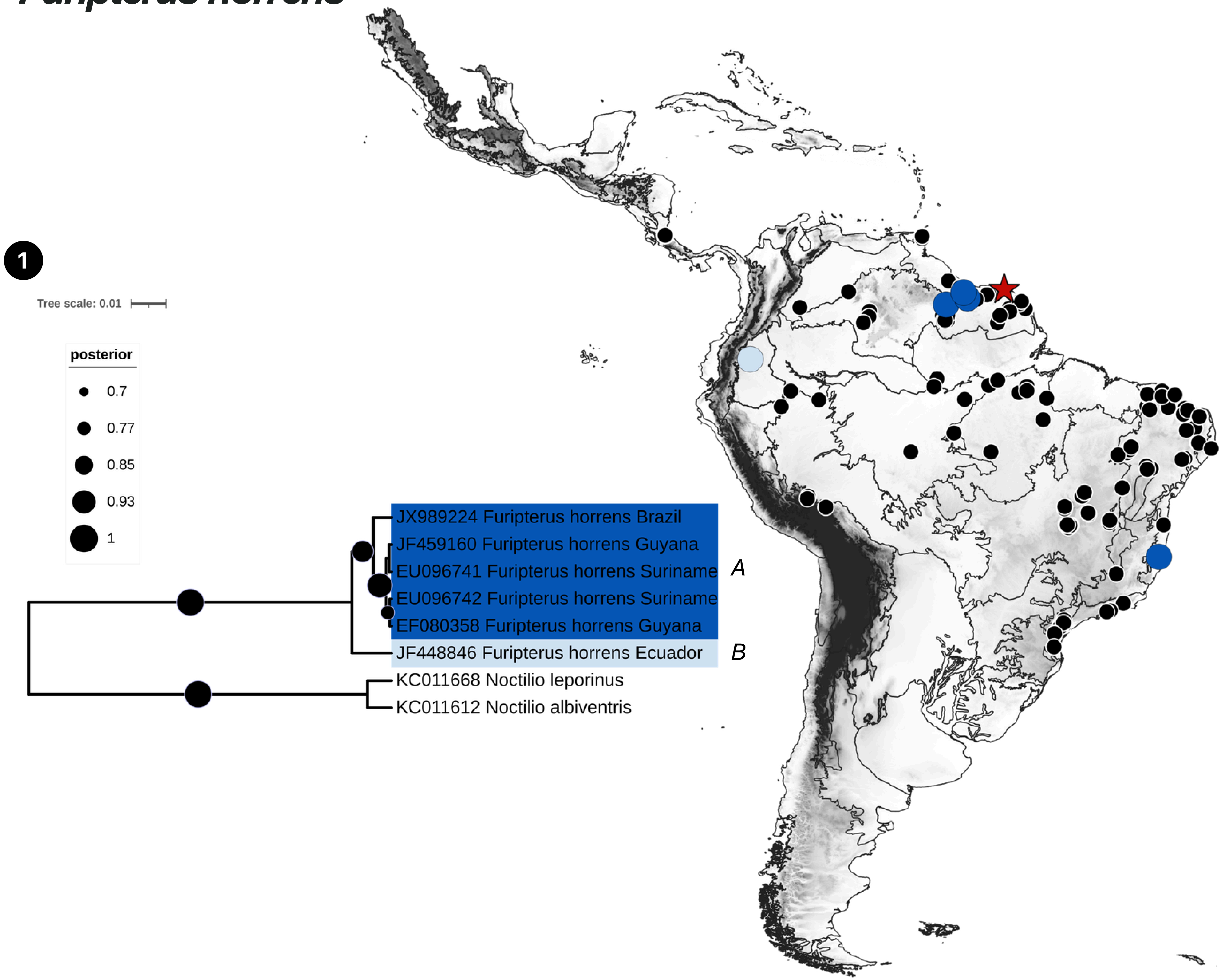

2

3

Mean genetic distances (Simple Distance)

Diagonal (red) = mean intraspecific distance | Off-diagonal (black) = mean interspecific distance | n = number of unique pairs

Especie	F. *horrens* A	F. *horrens* B
**F. *horrens* A**	**0.0131 (n=10)**	**0.0487 (n=5)**
**F. *horrens* B**	**0.0487 (n=5)**	--

4

Max intraspecific: 0.0268 | Min interspecific: 0.0453 | Gap size: 0.0185

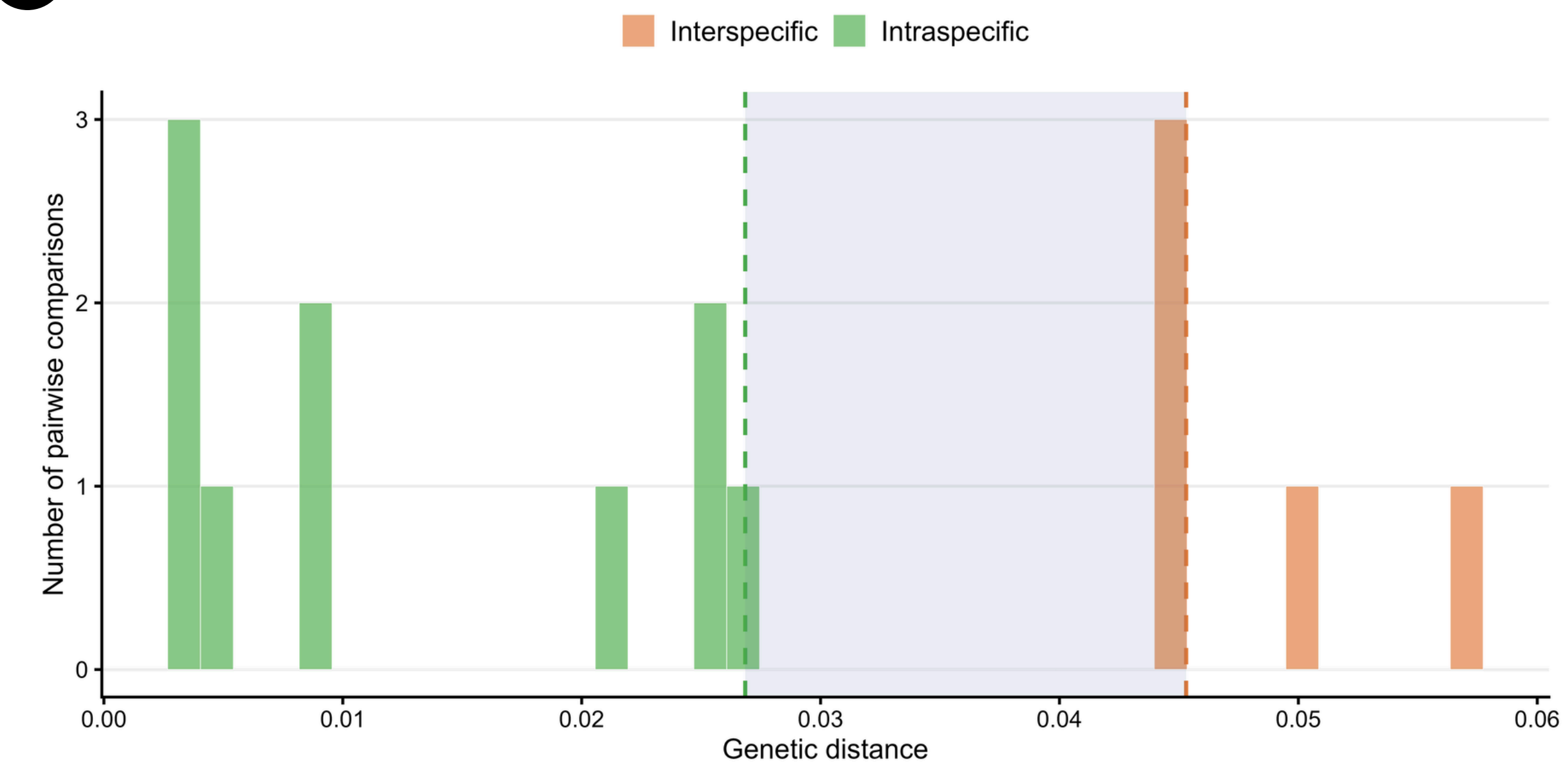

5 COI + CYTB

Moras et al. (2025) Mamm. Biol.

Figure 2

Fig. 2. Phylogenetic, geographic, and supporting evidence for candidate lineages in *Furipterus horrens*. (1) Phylogenetic hypothesis based on COI sequences, with colored clades representing candidate lineages identified through molecular species delimitation. (2) Geographic distribution map, where colored points correspond to the lineages recovered in the phylogeny and black points represent the overall known distribution of the species complex. (3) Table of genetic distances (when sufficient data are available), reporting intra- and inter-lineage divergence among delimited groups. (4) Histogram of genetic distances illustrating the barcode gap. (5) Phylogenetic hypotheses from previous studies (when available), provided for comparison with our proposed delimitations. (6) Additional lines of evidence (occasionally available), such as geometric morphometrics or other independent datasets supporting lineage differentiation. Red star indicates holotype.

Note: *When this symbol (⚠) appears next to the phylogeny, it indicates that the delimitation should be interpreted with caution, as additional sampling is needed. When it appears next to the table, it means that genetic distances are low; therefore, we recommend that these results also be considered with caution. In both cases, we have retained the delimitations as inferred by our methods.*

Noctilio albiventris

1

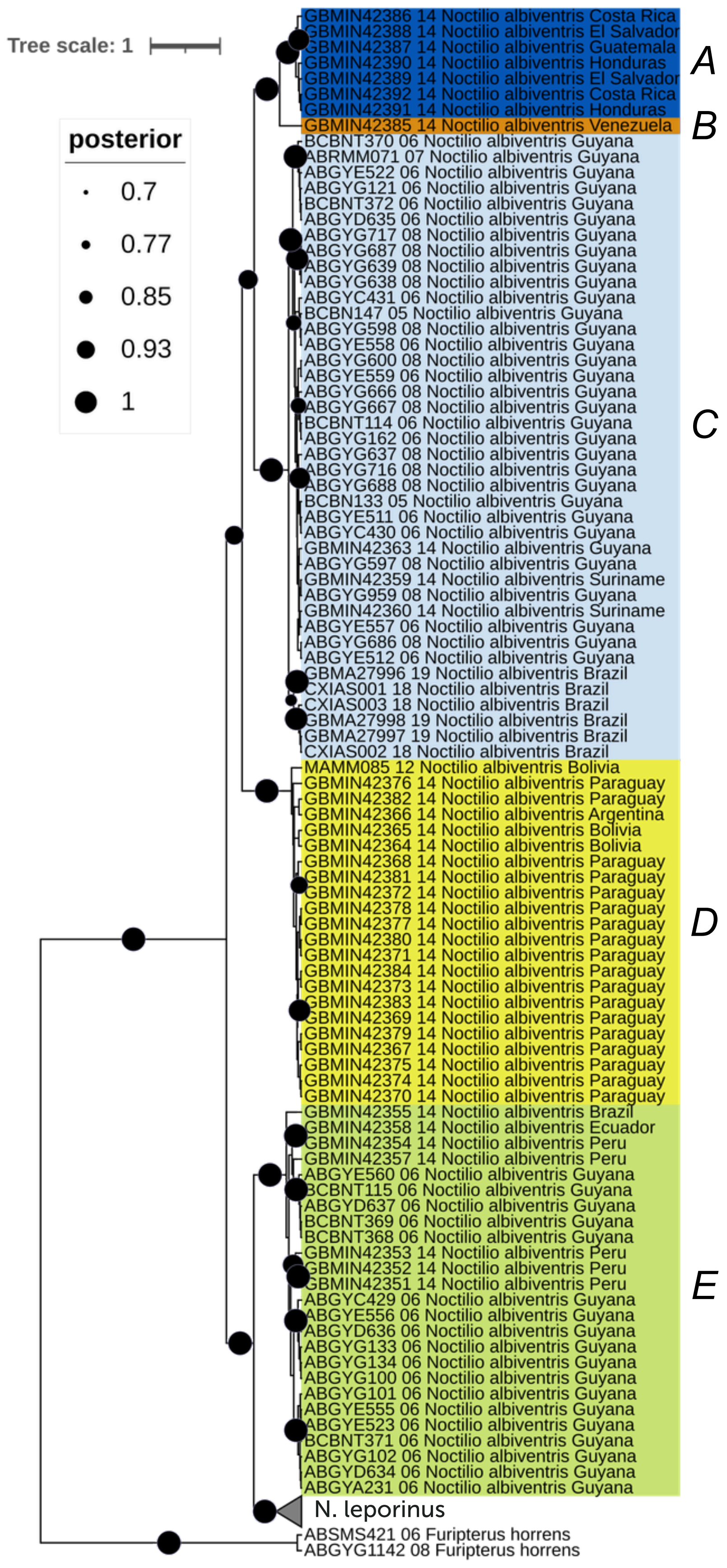

2

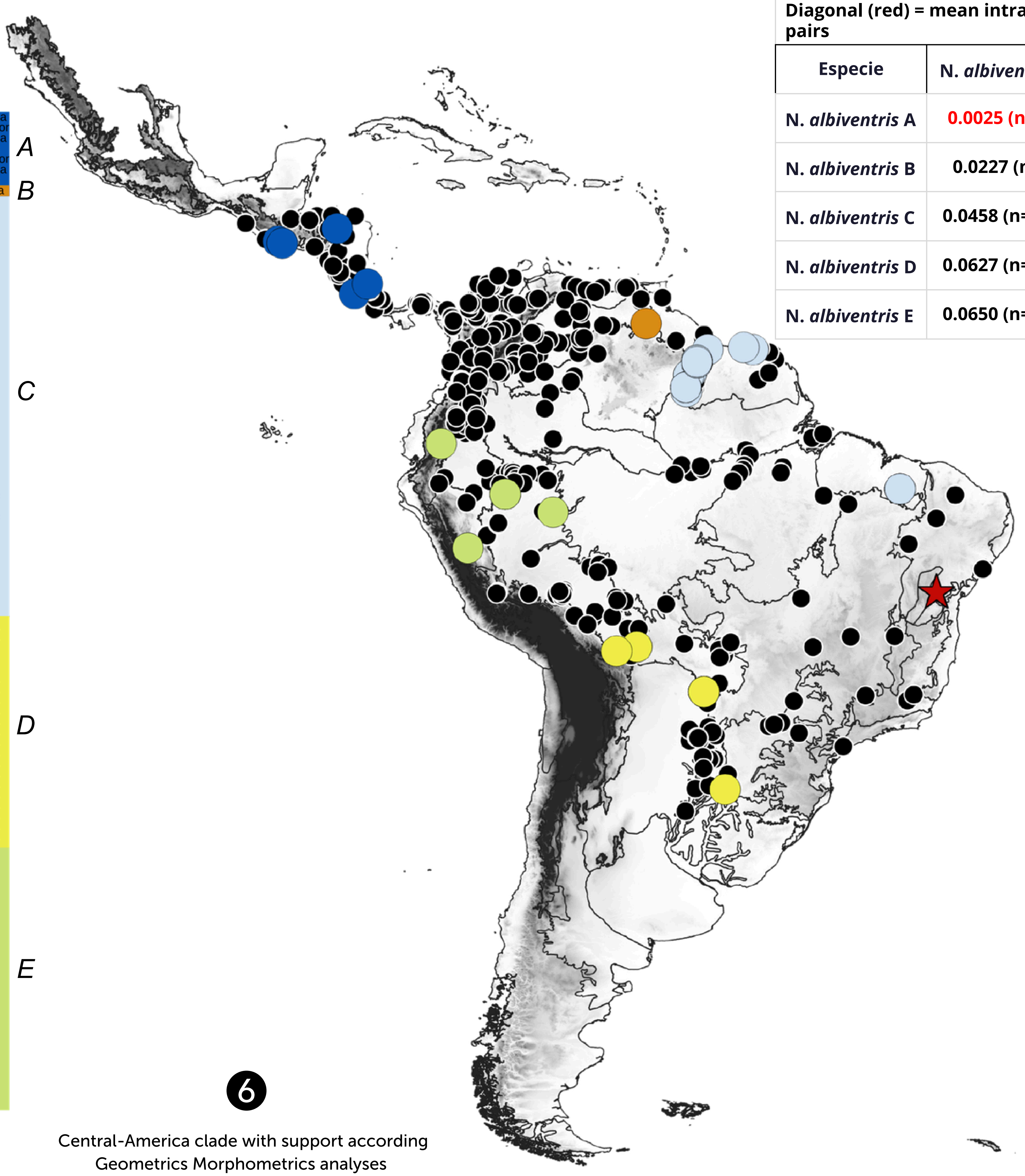

3

Mean genetic distances (Simple Distance)

Diagonal (red) = mean intraspecific distance | Off-diagonal (black) = mean interspecific distance | n = number of unique pairs

Especie	*N. albiventris* A	*N. albiventris* B	*N. albiventris* C	*N. albiventris* D	*N. albiventris* E	*N. leporinus*
N. albiventris A	0.0025 (n=21)	0.0227 (n=7)	0.0458 (n=280)	0.0627 (n=154)	0.0650 (n=175)	0.0686 (n=378)
N. albiventris B	0.0227 (n=7)		0.0487 (n=40)	0.0559 (n=22)	0.0634 (n=25)	0.0638 (n=54)
N. albiventris C	0.0458 (n=280)	0.0487 (n=40)	0.0035 (n=780)	0.0597 (n=880)	0.0598 (n=1000)	0.0714 (n=2160)
N. albiventris D	0.0627 (n=154)	0.0559 (n=22)	0.0597 (n=880)	0.0027 (n=231)	0.0705 (n=550)	0.0760 (n=1188)
N. albiventris E	0.0650 (n=175)	0.0634 (n=25)	0.0598 (n=1000)	0.0705 (n=550)	0.0073 (n=300)	0.0392 (n=1350)

4

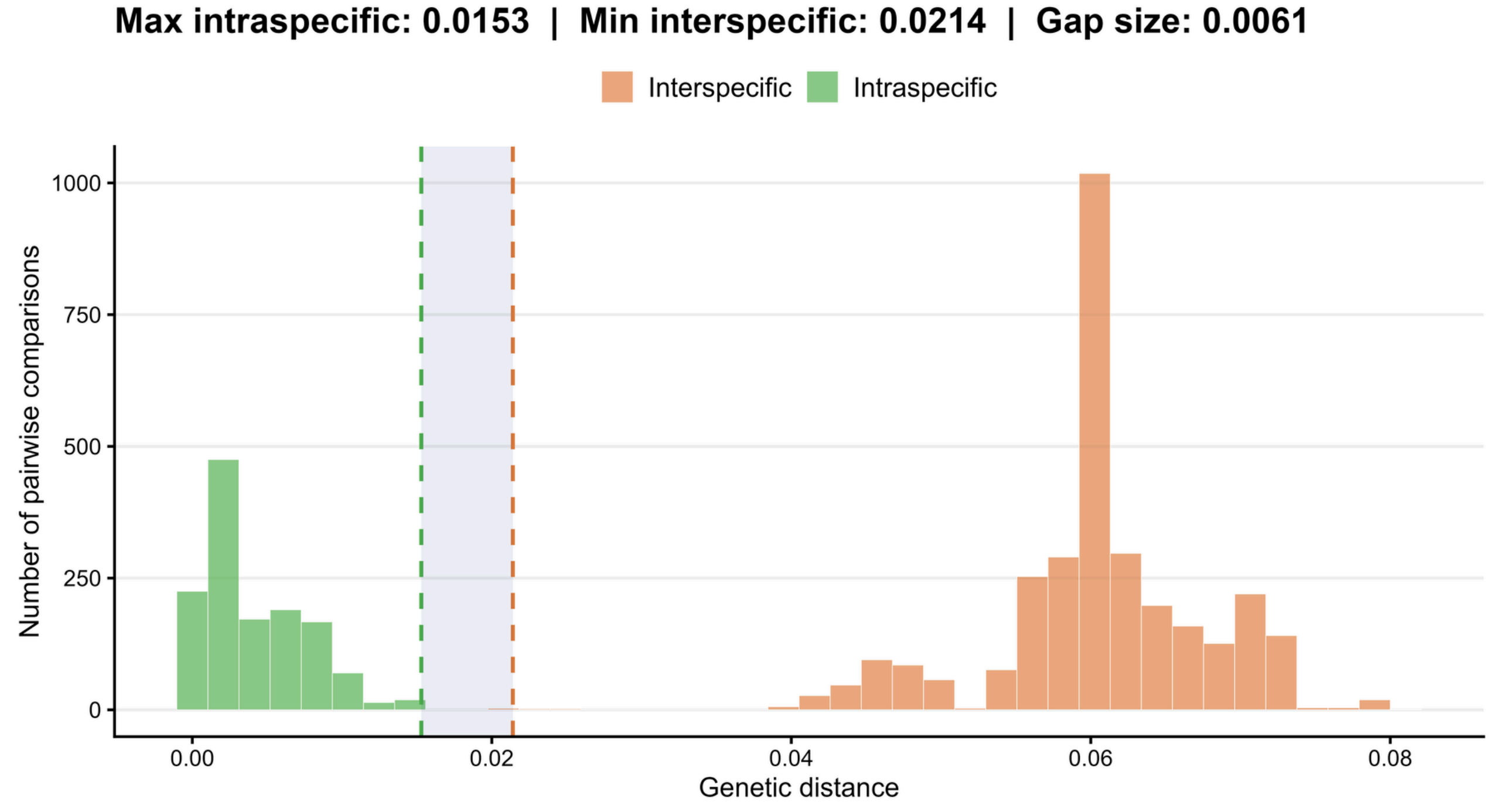

5 COI+CYTB

Pavan et al., (2012) Biol. J. Linn. Soc

Khan et al., (2013) Syst. Biol.

6

Central-America clade with support according Geometrics Morphometrics analyses

Ospina-Garcés & León-Paniagua (2022) Org. Divers. Evol.

Figure 3

Fig. 3. Phylogenetic, geographic, and supporting evidence for candidate lineages in *Noctilio albiventris*. (1) Phylogenetic hypothesis based on COI sequences, with colored clades representing candidate lineages identified through molecular species delimitation. (2) Geographic distribution map, where colored points correspond to the lineages recovered in the phylogeny and black points represent the overall known distribution of the species complex. (3) Table of genetic distances (when sufficient data are available), reporting intra- and inter-lineage divergence among delimited groups. (4) Histogram of genetic distances illustrating the barcode gap. (5) Phylogenetic hypotheses from previous studies (when available), provided for comparison with our proposed delimitations. (6) Additional lines of evidence (occasionally available), such as geometric morphometrics or other independent datasets supporting lineage differentiation. Red star indicates holotype.

Note: *When this symbol (⚠) appears next to the phylogeny, it indicates that the delimitation should be interpreted with caution, as additional sampling is needed. When it appears next to the table, it means that genetic distances are low; therefore, we recommend that these results also be considered with caution. In both cases, we have retained the delimitations as inferred by our methods.*

Noctilio leporinus

1

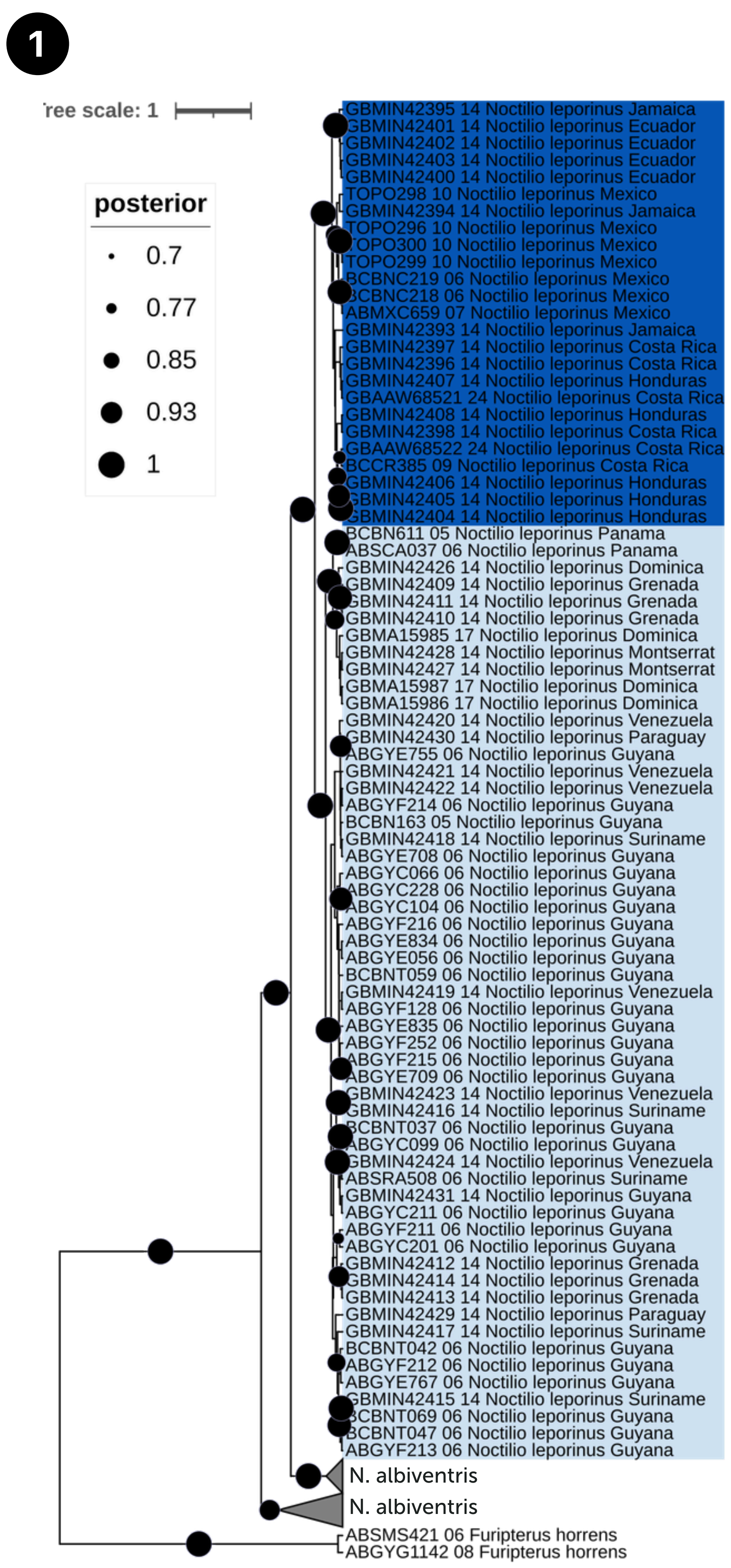

2

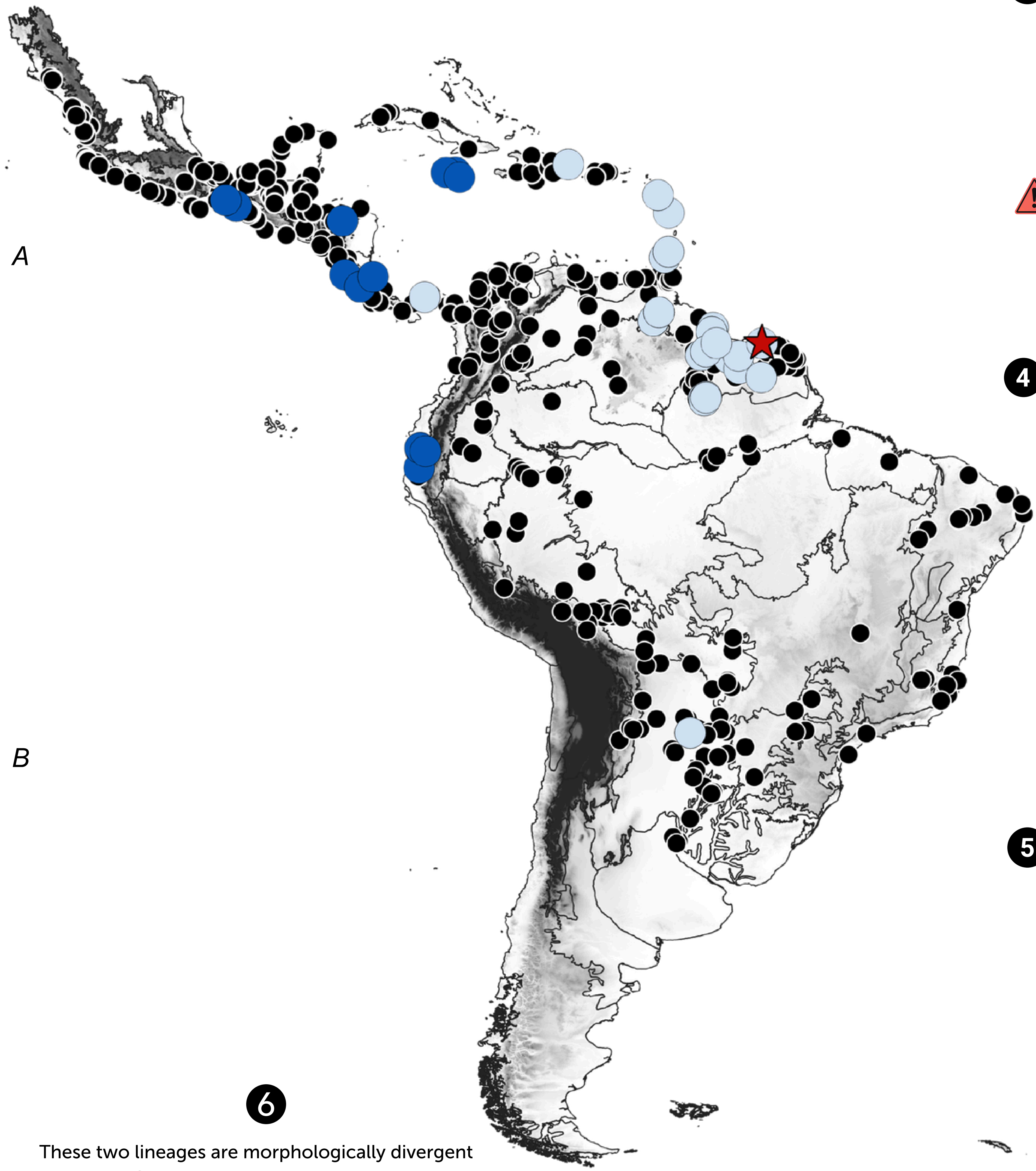

3

Mean genetic distances (Simple Distance)		
Diagonal (red) = mean intraspecific distance | Off-diagonal (black) = mean interspecific distance | n = number of unique pairs		
**Especie**	***N. leporinus A***	***N. leporinus B***
⚠ ***N. leporinus A***	**0.0042 (n=300)**	**0.0233 (n=1375)**
N. leporinus B	**0.0233 (n=1375)**	**0.0050 (n=1485)**

4

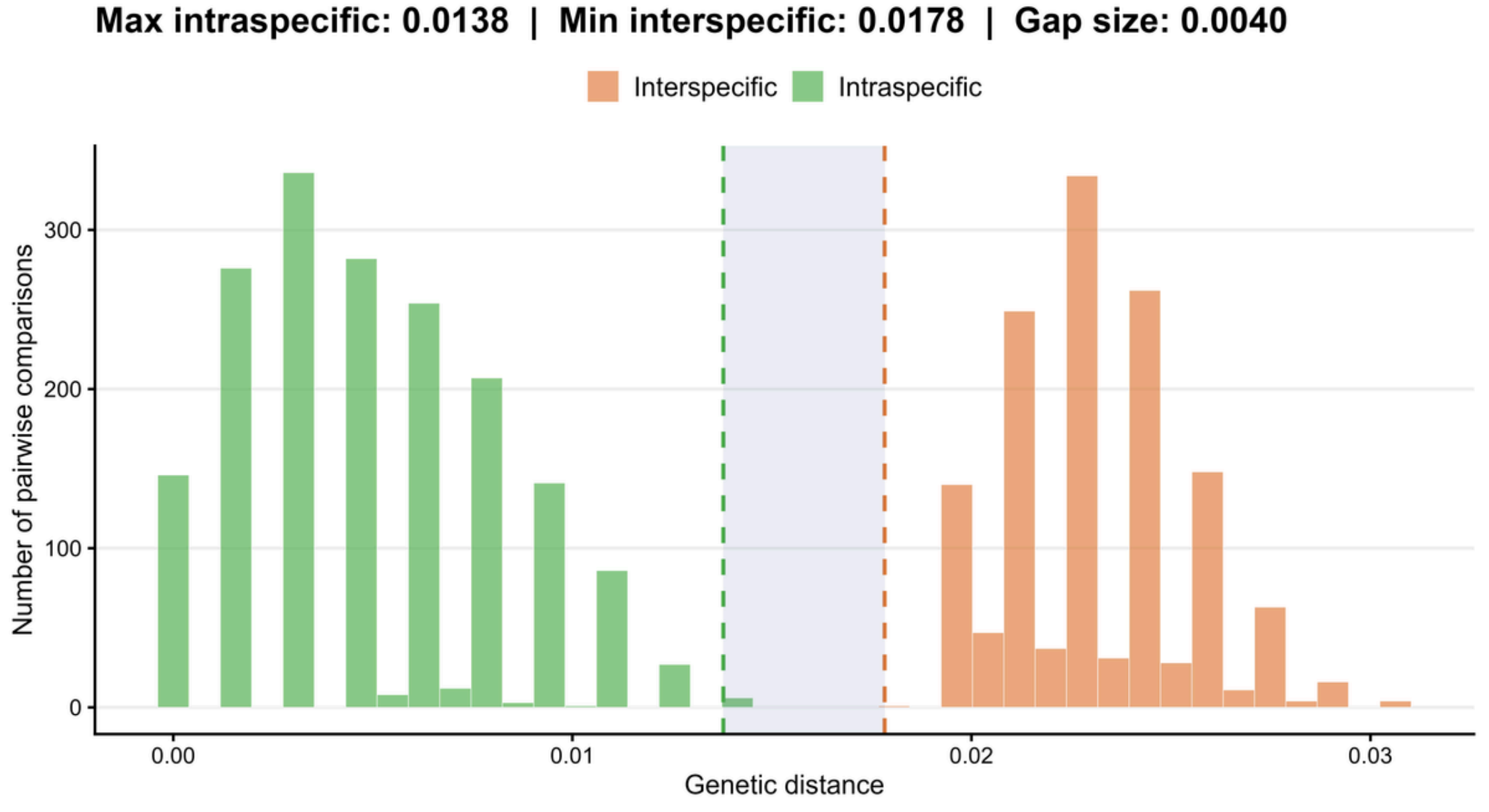

5 COI+CYTB

Khan et al., (2013) Syst. Biol.

6

These two lineages are morphologically divergent according Geometrics Morphometrics analyses

Ospina-Garcés & León-Paniagua (2022) Org. Divers. Evol.

Figure 4

Fig. 4. Phylogenetic, geographic, and supporting evidence for candidate lineages in *Noctilio leporinus*. (1) Phylogenetic hypothesis based on COI sequences, with colored clades representing candidate lineages identified through molecular species delimitation. (2) Geographic distribution map, where colored points correspond to the lineages recovered in the phylogeny and black points represent the overall known distribution of the species complex. (3) Table of genetic distances (when sufficient data are available), reporting intra- and inter-lineage divergence among delimited groups. (4) Histogram of genetic distances illustrating the barcode gap. (5) Phylogenetic hypotheses from previous studies (when available), provided for comparison with our proposed delimitations. (6) Additional lines of evidence (occasionally available), such as geometric morphometrics or other independent datasets supporting lineage differentiation. Red star indicates holotype.

Note: *When this symbol (⚠) appears next to the phylogeny, it indicates that the delimitation should be interpreted with caution, as additional sampling is needed. When it appears next to the table, it means that genetic distances are low; therefore, we recommend that these results also be considered with caution. In both cases, we have retained the delimitations as inferred by our methods.*

Cormura brevirostris

1

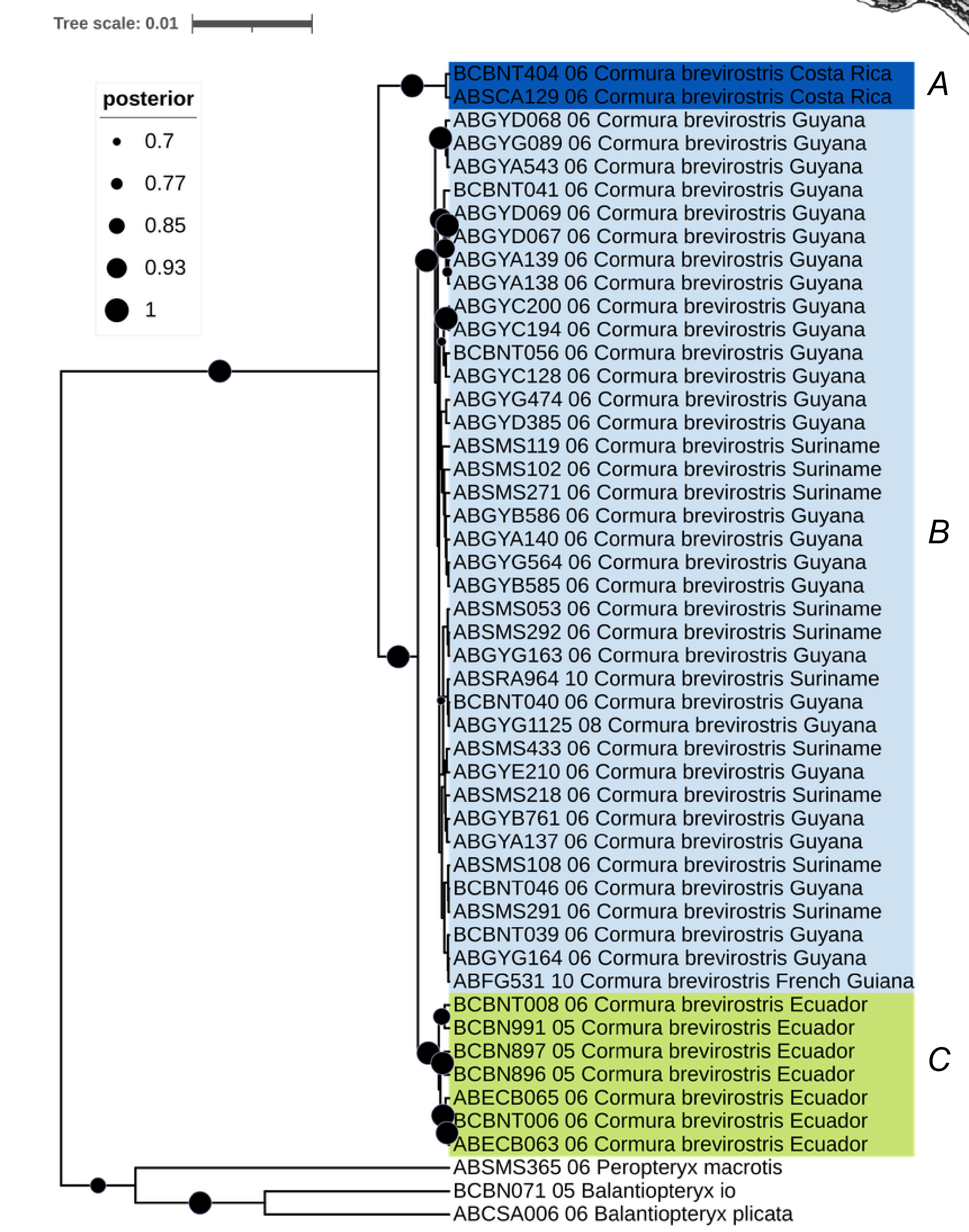

2

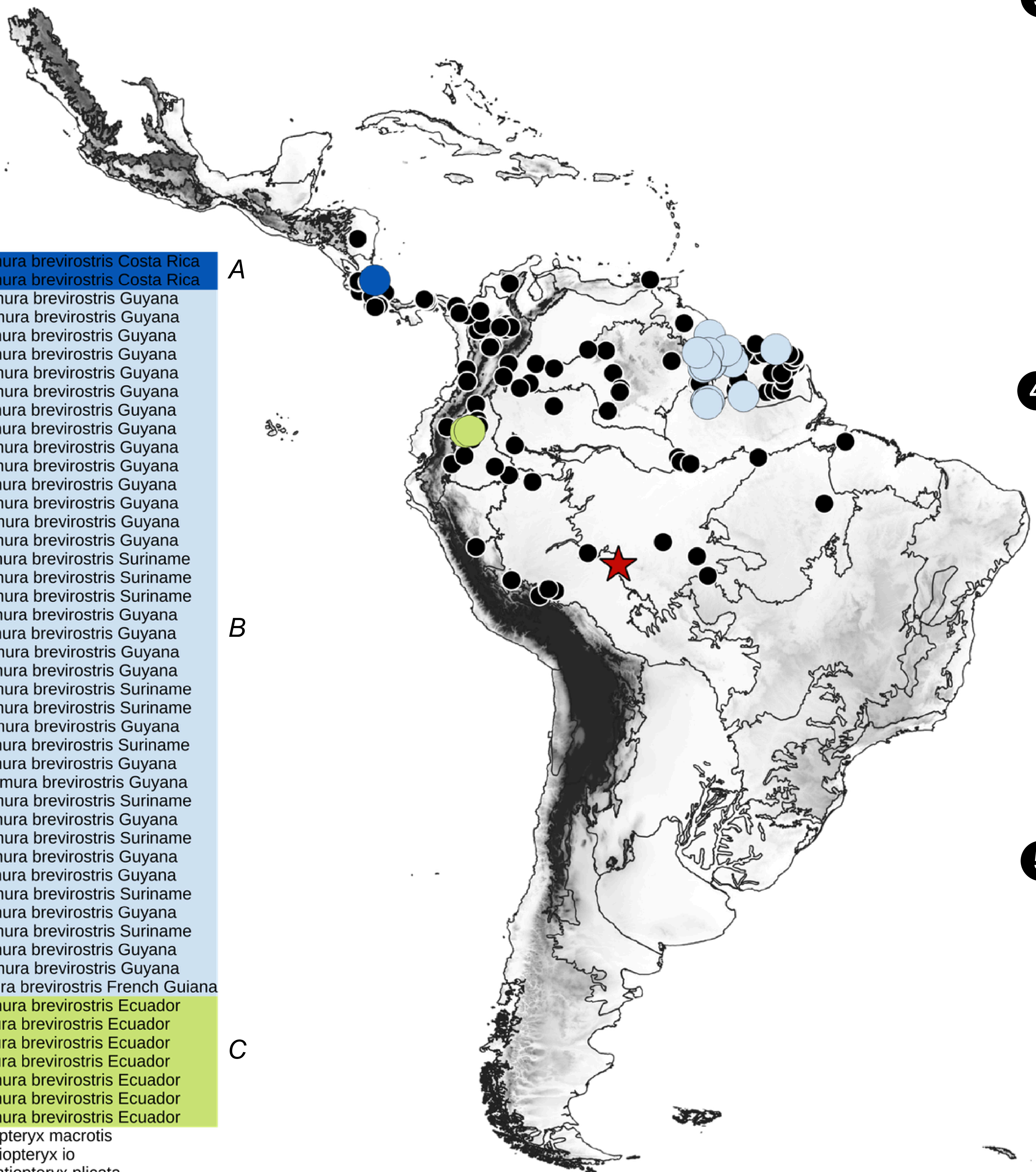

3

Mean genetic distances (Simple distance)

Diagonal (red) = mean intraspecific distance | Off-diagonal (black) = mean interspecific distance | n = number of unique pairs

Especie	*C. brevirostris A*	*C. brevirostris B*	*C. brevirostris C*
C. brevirostris A	0.0032 (n=1)	0.0663 (n=76)	0.0749 (n=14)
C. brevirostris B	0.0663 (n=76)	0.0053 (n=703)	0.0296 (n=266)
C. brevirostris C	0.0749 (n=14)	0.0296 (n=266)	0.0082 (n=21)

4

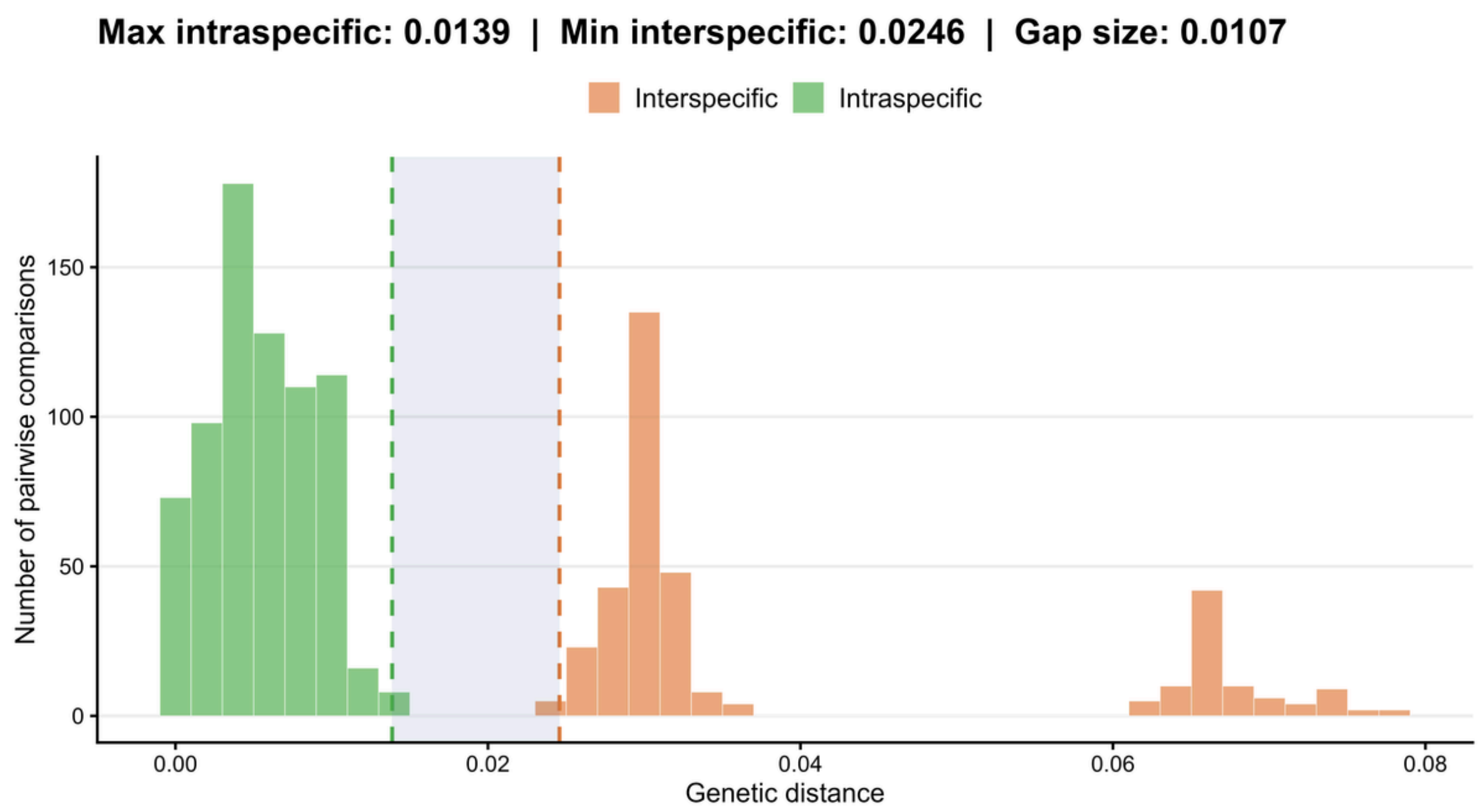

5 COI+CYTB+DBY

Correa et al., (2017) J. Mammal.

Figure 5

Fig. 5. Phylogenetic, geographic, and supporting evidence for candidate lineages in *Cormura brevirostris.* (1) Phylogenetic hypothesis based on COI sequences, with colored clades representing candidate lineages identified through molecular species delimitation. (2) Geographic distribution map, where colored points correspond to the lineages recovered in the phylogeny and black points represent the overall known distribution of the species complex. (3) Table of genetic distances (when sufficient data are available), reporting intra- and inter-lineage divergence among delimited groups. (4) Histogram of genetic distances illustrating the barcode gap. (5) Phylogenetic hypotheses from previous studies (when available), provided for comparison with our proposed delimitations. (6) Additional lines of evidence (occasionally available), such as geometric morphometrics or other independent datasets supporting lineage differentiation. Red star indicates holotype.

Note: *When this symbol (⚠) appears next to the phylogeny, it indicates that the delimitation should be interpreted with caution, as additional sampling is needed. When it appears next to the table, it means that genetic distances are low; therefore, we recommend that these results also be considered with caution. In both cases, we have retained the delimitations as inferred by our methods.*

Cyttarops alecto

1

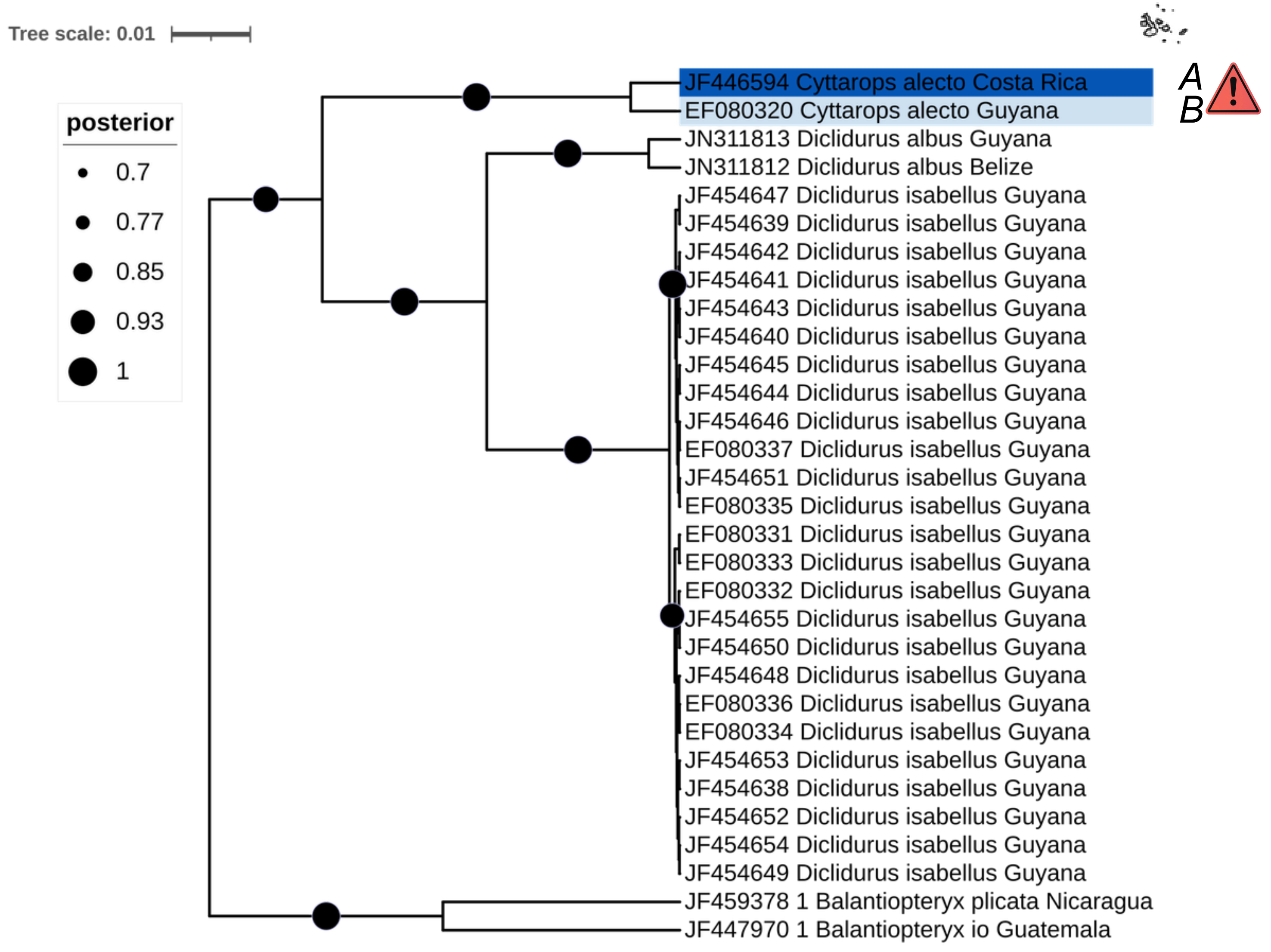

2

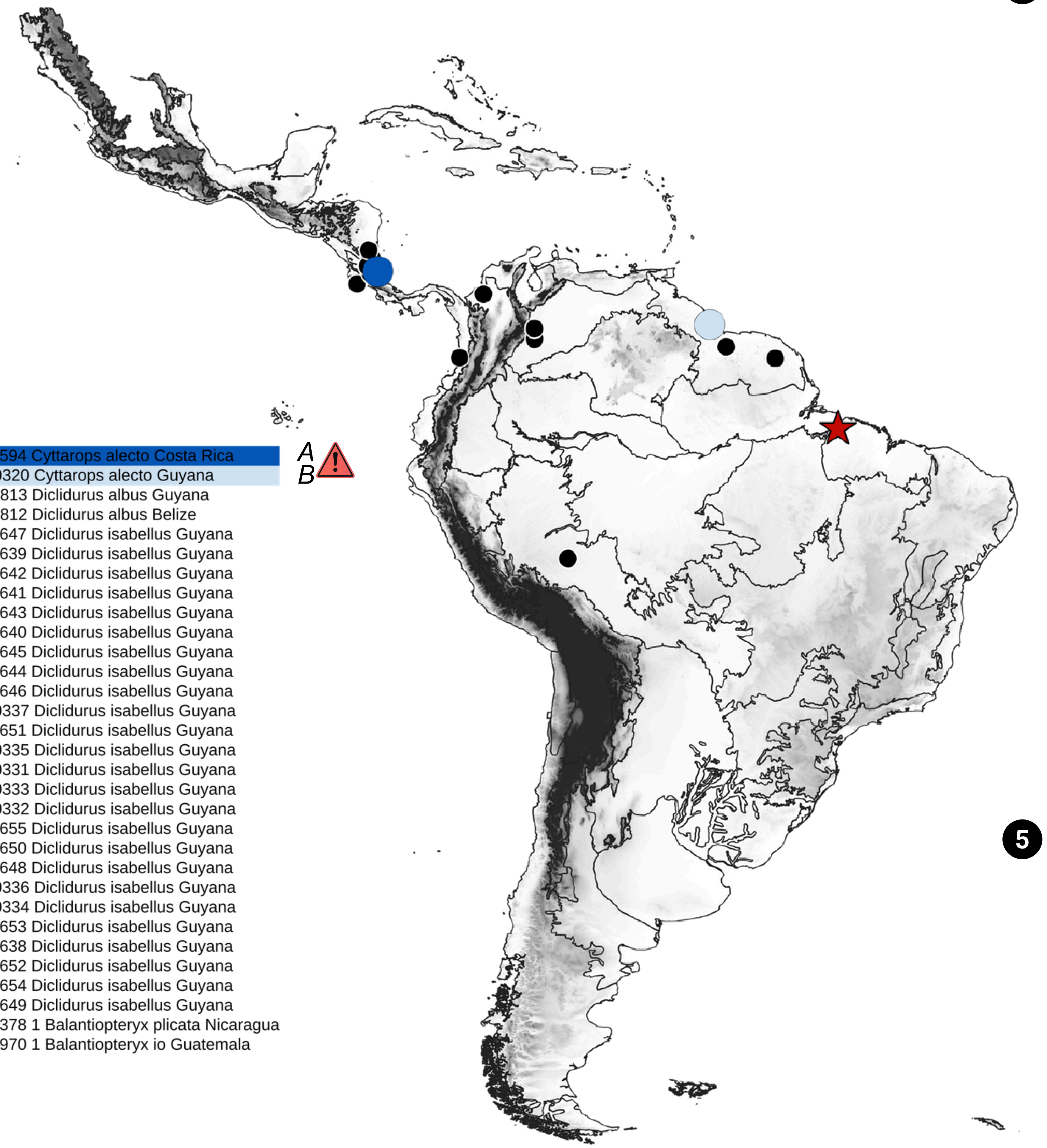

3

Mean genetic distances (Simple Distance)

Diagonal (red) = mean intraspecific distance | Off-diagonal (black) = mean interspecific distance | n = number of unique pairs

Especie	*C. alecto* Guyana	*C. alecto* Costa Rica
C. alecto Guyana	--	0.0525 (n=1)
C. alecto Costa Rica	0.0525 (n=1)	--

5 COI

Clare et al., (2011) PloS One

Figure 6

Fig. 6. Phylogenetic, geographic, and supporting evidence for candidate lineages in *Cyttarops alecto*. (1) Phylogenetic hypothesis based on COI sequences, with colored clades representing candidate lineages identified through molecular species delimitation. (2) Geographic distribution map, where colored points correspond to the lineages recovered in the phylogeny and black points represent the overall known distribution of the species complex. (3) Table of genetic distances (when sufficient data are available), reporting intra- and inter-lineage divergence among delimited groups. (4) Histogram of genetic distances illustrating the barcode gap. (5) Phylogenetic hypotheses from previous studies (when available), provided for comparison with our proposed delimitations. (6) Additional lines of evidence (occasionally available), such as geometric morphometrics or other independent datasets supporting lineage differentiation. Red star indicates holotype.

Note: *When this symbol (⚠) appears next to the phylogeny, it indicates that the delimitation should be interpreted with caution, as additional sampling is needed. When it appears next to the table, it means that genetic distances are low; therefore, we recommend that these results also be considered with caution. In both cases, we have retained the delimitations as inferred by our methods.*

Diclidurus albus

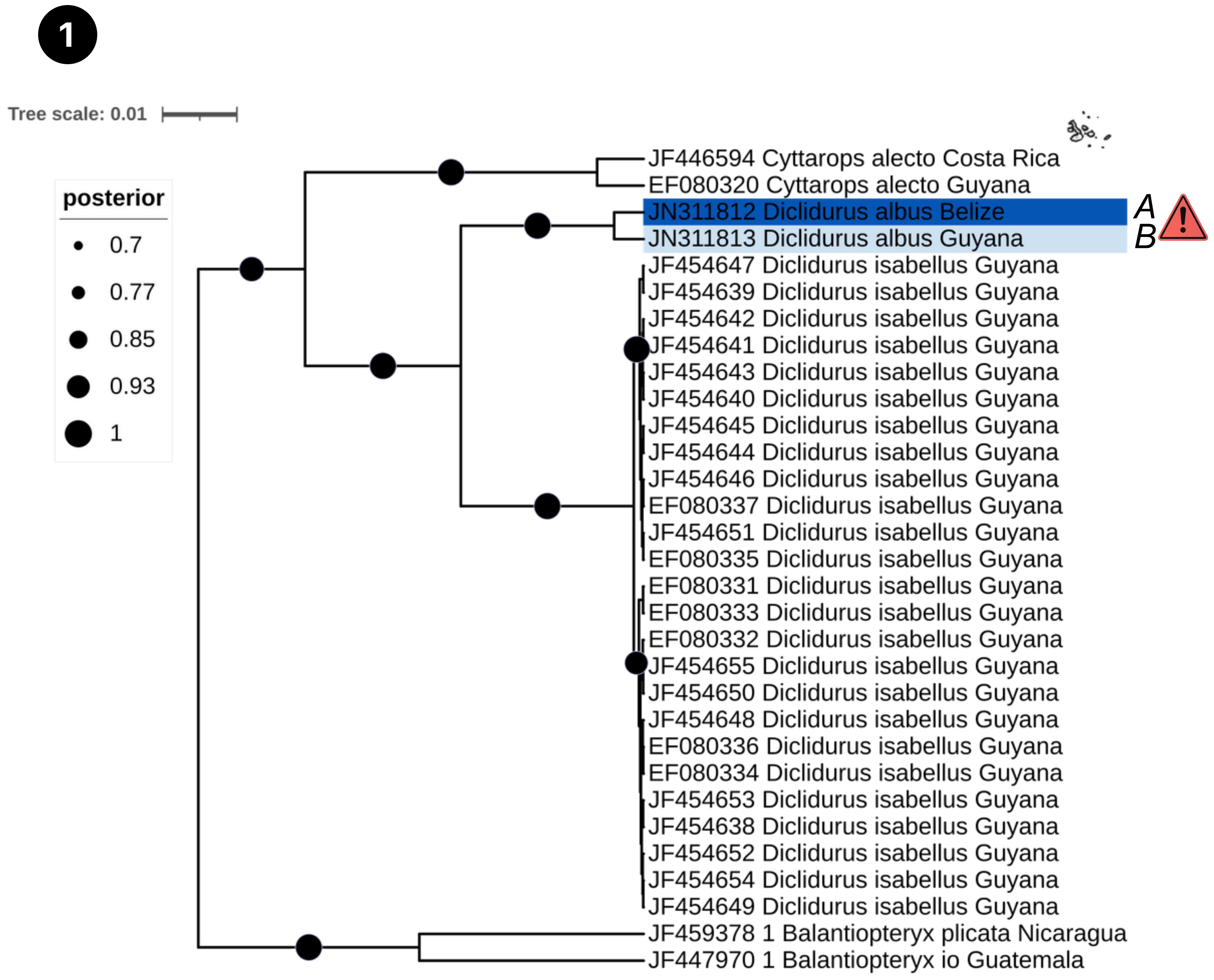

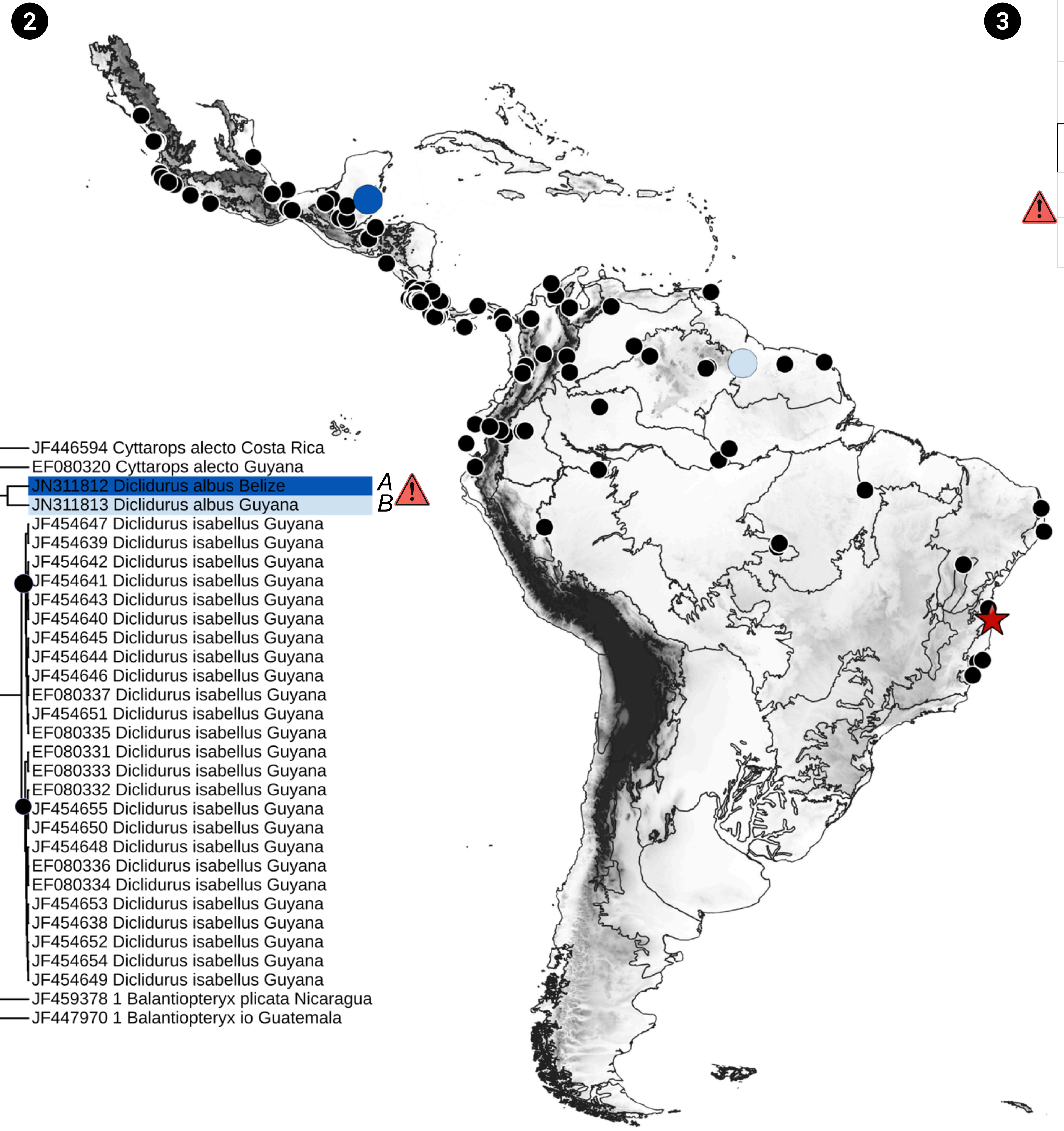

3

Mean genetic distances (Simple Distance)

Diagonal (red) = mean intraspecific distance | Off-diagonal (black) = mean interspecific distance | n = number of unique pairs

Especie	*D. albus* Belize	*D. albus* Guyana
⚠ *D. albus* Belize	--	0.0386 (n=1)
D. albus Guyana	0.0386 (n=1)	--

Figure 7

Fig. 7. Phylogenetic, geographic, and supporting evidence for candidate lineages in *Diclidurus albus*. (1) Phylogenetic hypothesis based on COI sequences, with colored clades representing candidate lineages identified through molecular species delimitation. (2) Geographic distribution map, where colored points correspond to the lineages recovered in the phylogeny and black points represent the overall known distribution of the species complex. (3) Table of genetic distances (when sufficient data are available), reporting intra- and inter-lineage divergence among delimited groups. (4) Histogram of genetic distances illustrating the barcode gap. (5) Phylogenetic hypotheses from previous studies (when available), provided for comparison with our proposed delimitations. (6) Additional lines of evidence (occasionally available), such as geometric morphometrics or other independent datasets supporting lineage differentiation. Red star indicates holotype.

Note: *When this symbol (⚠) appears next to the phylogeny, it indicates that the delimitation should be interpreted with caution, as additional sampling is needed. When it appears next to the table, it means that genetic distances are low; therefore, we recommend that these results also be considered with caution. In both cases, we have retained the delimitations as inferred by our methods.*

Peropteryx macrotis

1

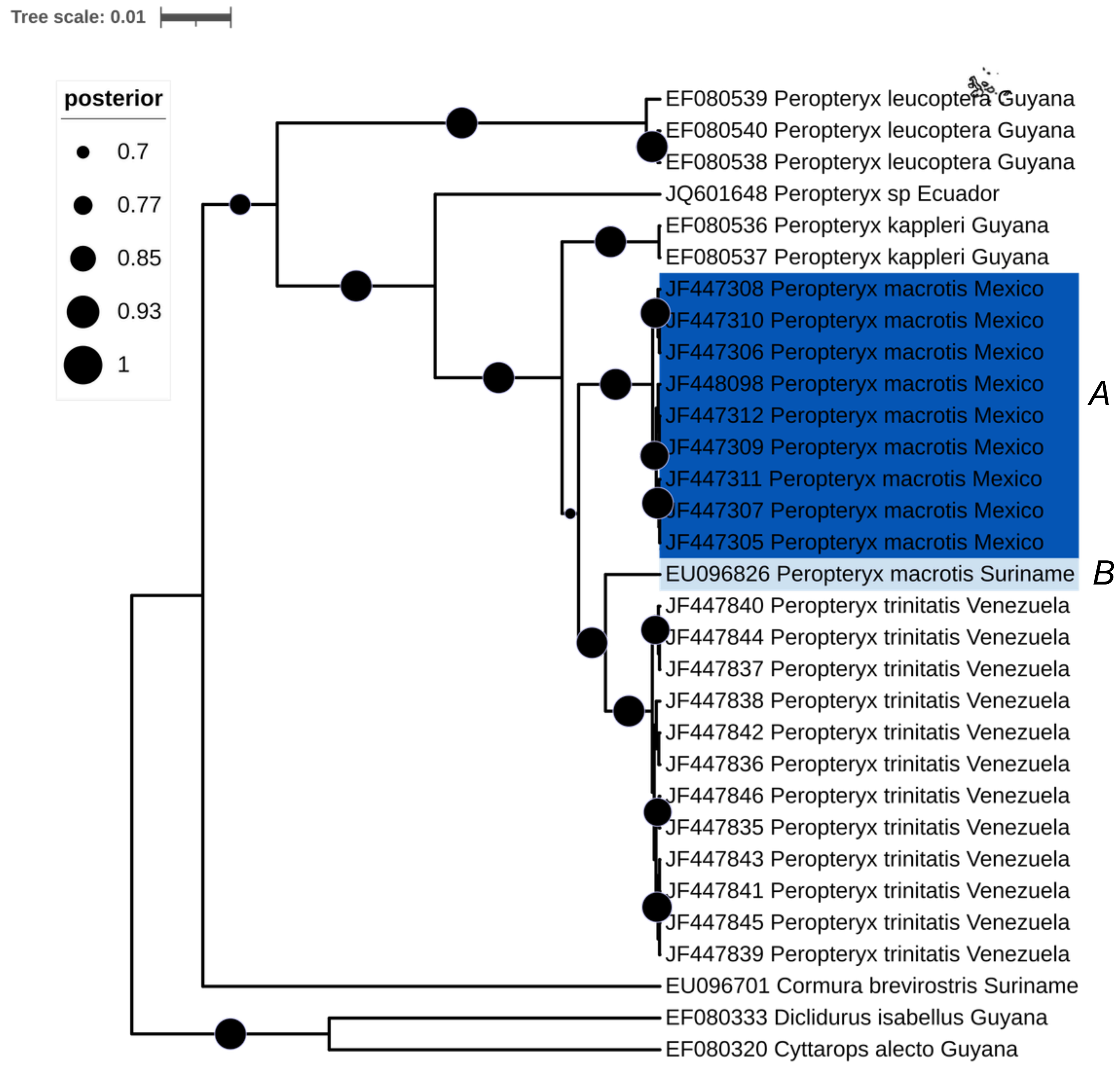

2

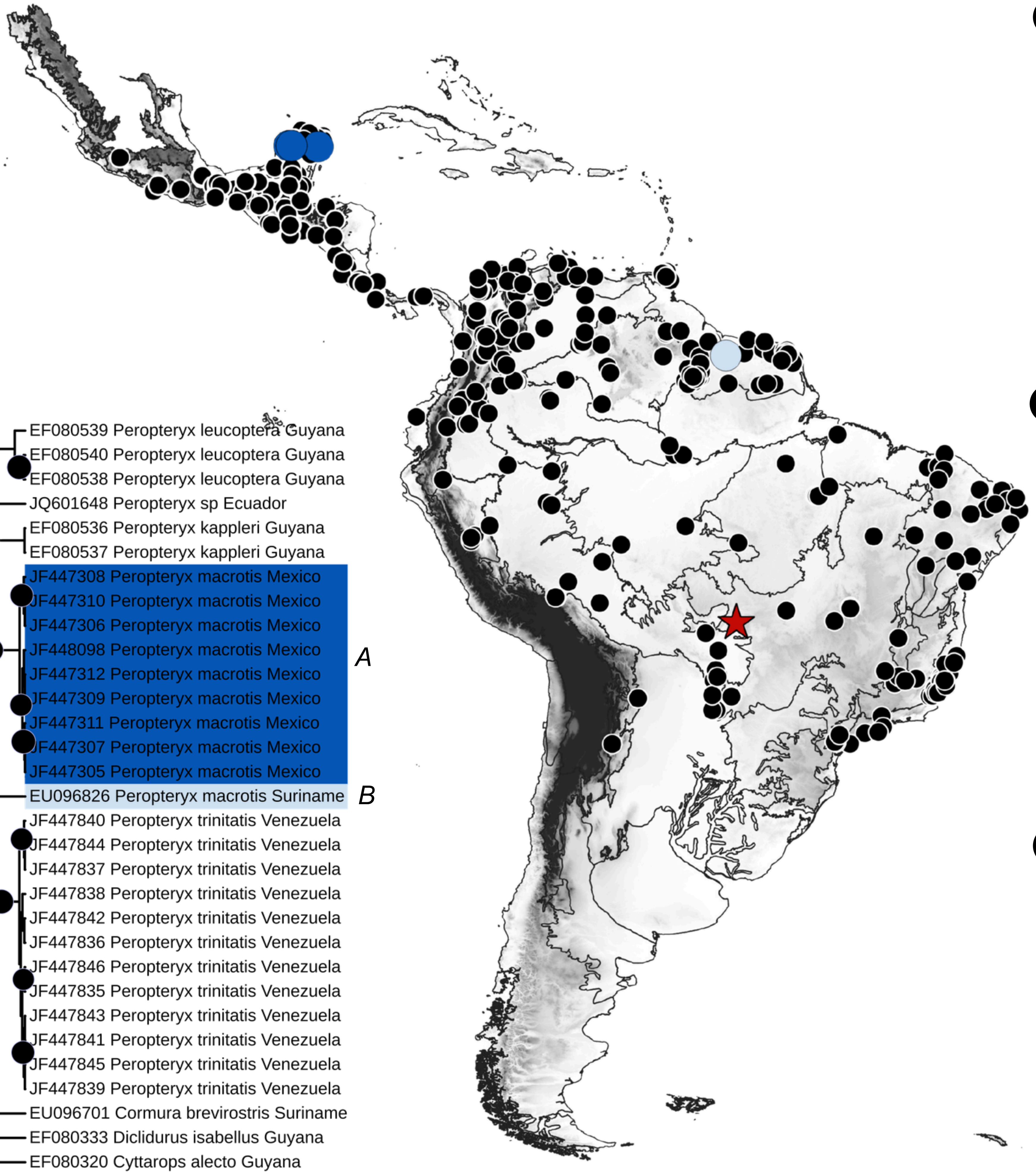

3

Mean genetic distances (Simple Distance)

Diagonal (red) = mean intraspecific distance | Off-diagonal (black) = mean interspecific distance | n = number of unique pairs

Especie	*P. macrotis* A	*P. macrotis* B
P. macrotis A	0.0023 (n=36)	0.0759 (n=9)
P. macrotis B	0.0759 (n=9)	--

4 **Max intraspecific: 0.0046 | Min interspecific: 0.0748 | Gap size: 0.0701**

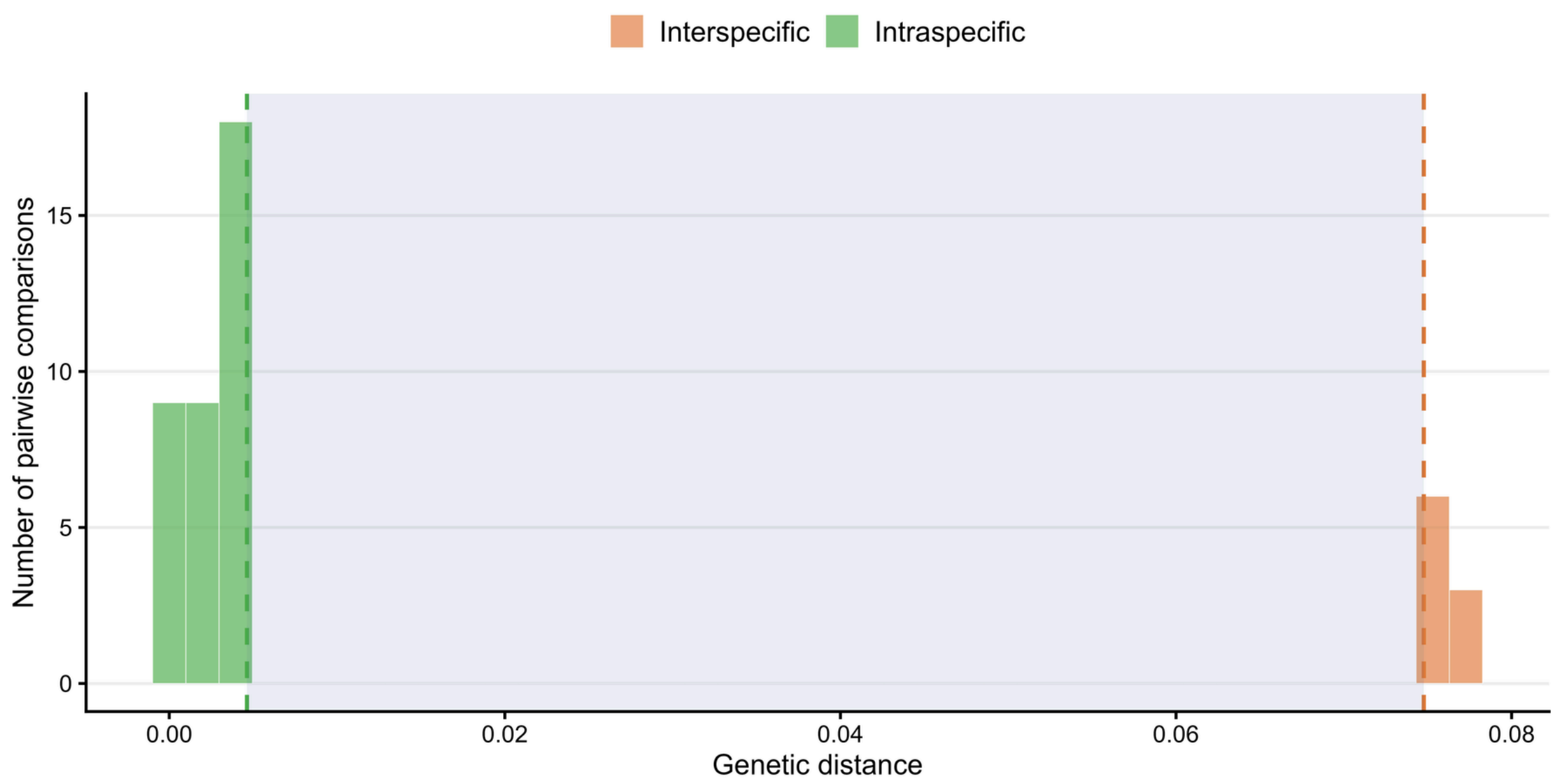

5 Cytb+Chd1+Dby+Usp9x

Lim et al., (2008) Biol. J. Linn. Soc.

Figure 8

Fig. 8. Phylogenetic, geographic, and supporting evidence for candidate lineages in *Peropteryx macrotis.* (1) Phylogenetic hypothesis based on COI sequences, with colored clades representing candidate lineages identified through molecular species delimitation. (2) Geographic distribution map, where colored points correspond to the lineages recovered in the phylogeny and black points represent the overall known distribution of the species complex. (3) Table of genetic distances (when sufficient data are available), reporting intra- and inter-lineage divergence among delimited groups. (4) Histogram of genetic distances illustrating the barcode gap. (5) Phylogenetic hypotheses from previous studies (when available), provided for comparison with our proposed delimitations. (6) Additional lines of evidence (occasionally available), such as geometric morphometrics or other independent datasets supporting lineage differentiation. Red star indicates holotype.

Note: *When this symbol (⚠) appears next to the phylogeny, it indicates that the delimitation should be interpreted with caution, as additional sampling is needed. When it appears next to the table, it means that genetic distances are low; therefore, we recommend that these results also be considered with caution. In both cases, we have retained the delimitations as inferred by our methods.*

Rhynchonycteris naso

1

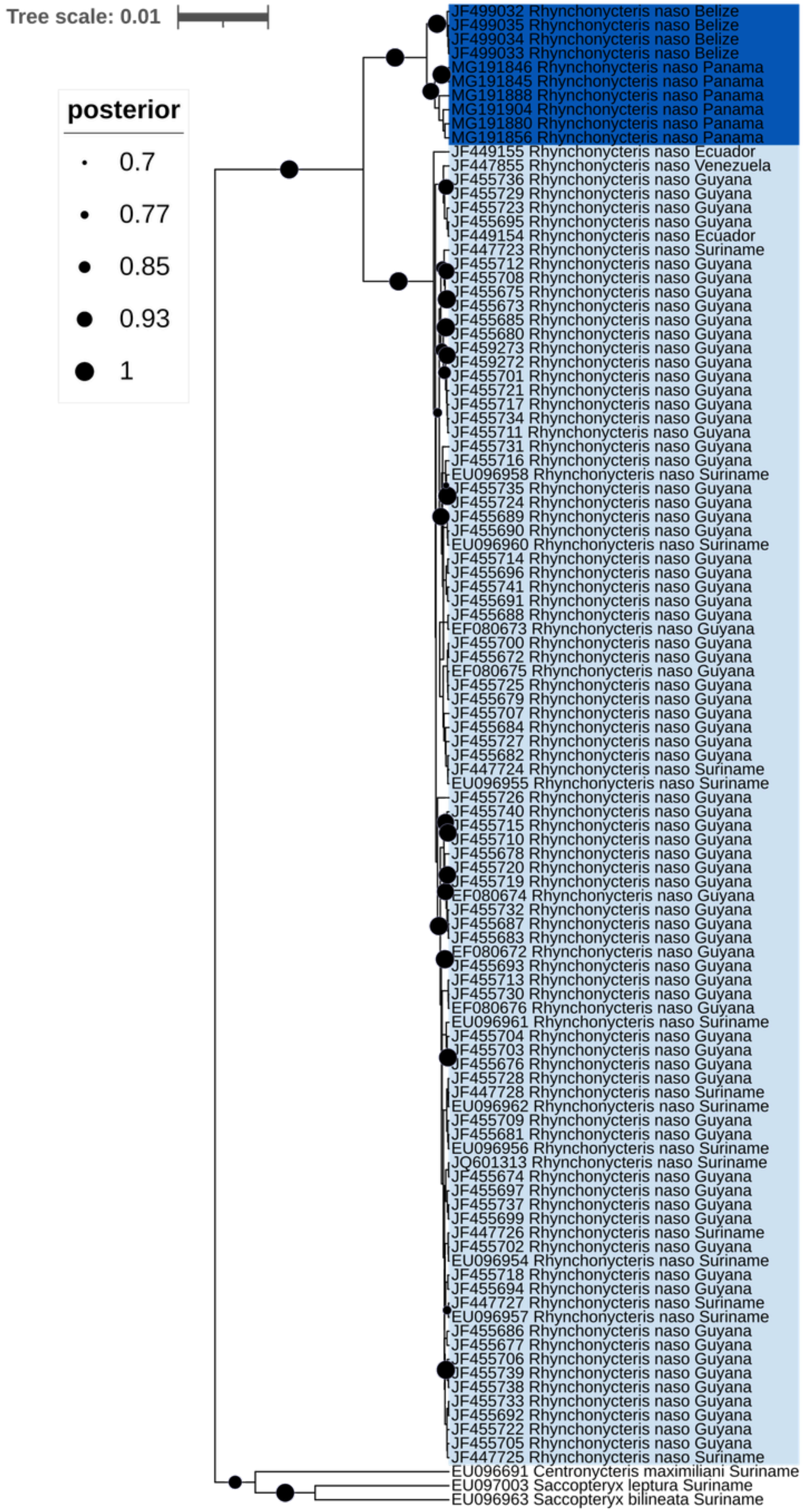

2

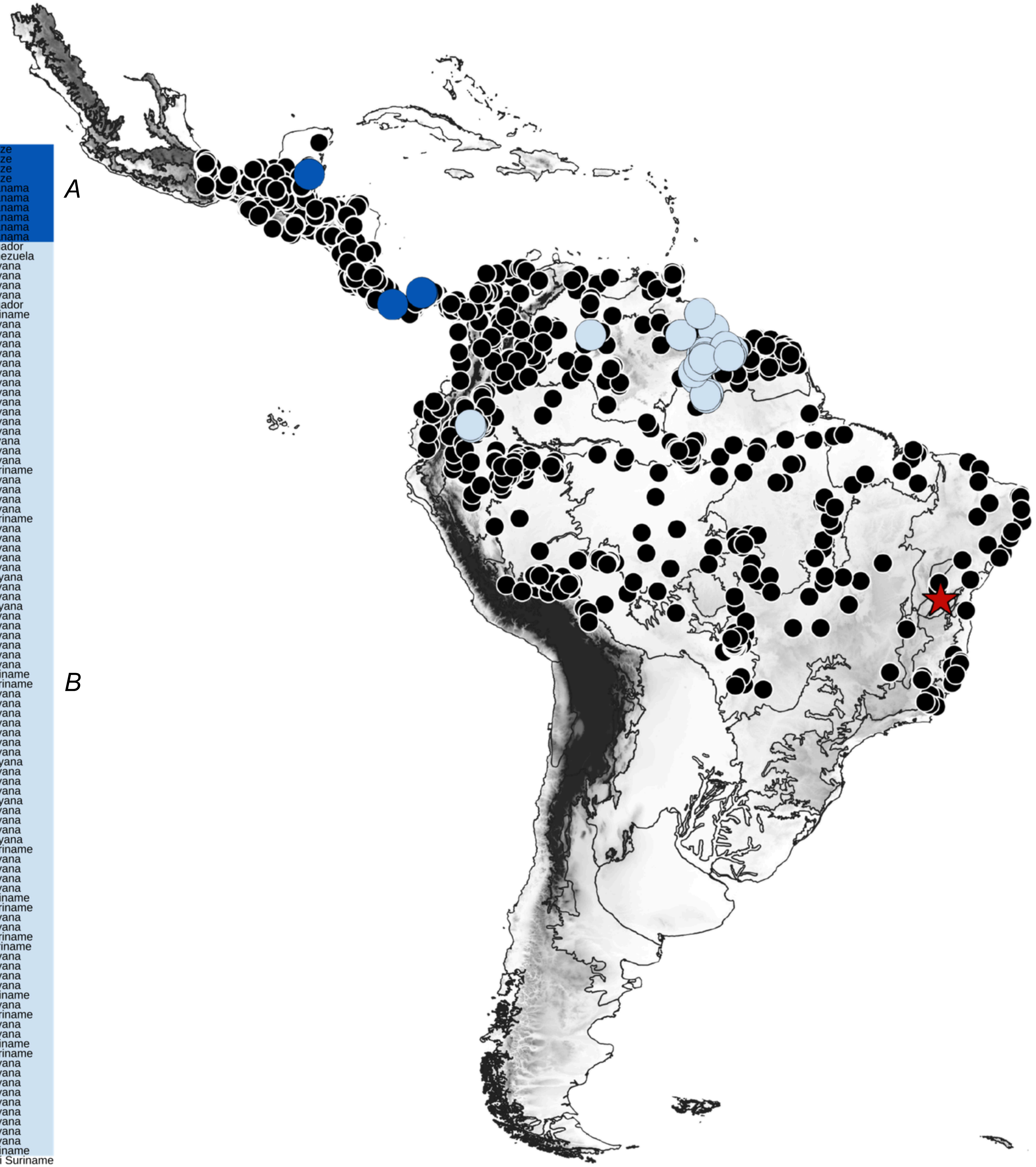

3

Mean genetic distances (Simple Distance)

Diagonal (red) = mean intraspecific distance | Off-diagonal (black) = mean interspecific distance | n = number of unique pairs

Especie	*R. naso* A	*R. naso* B
R. naso A	0.0190 (n=45)	0.0999 (n=940)
R. naso B	0.0999 (n=940)	0.0088 (n=4371)

4

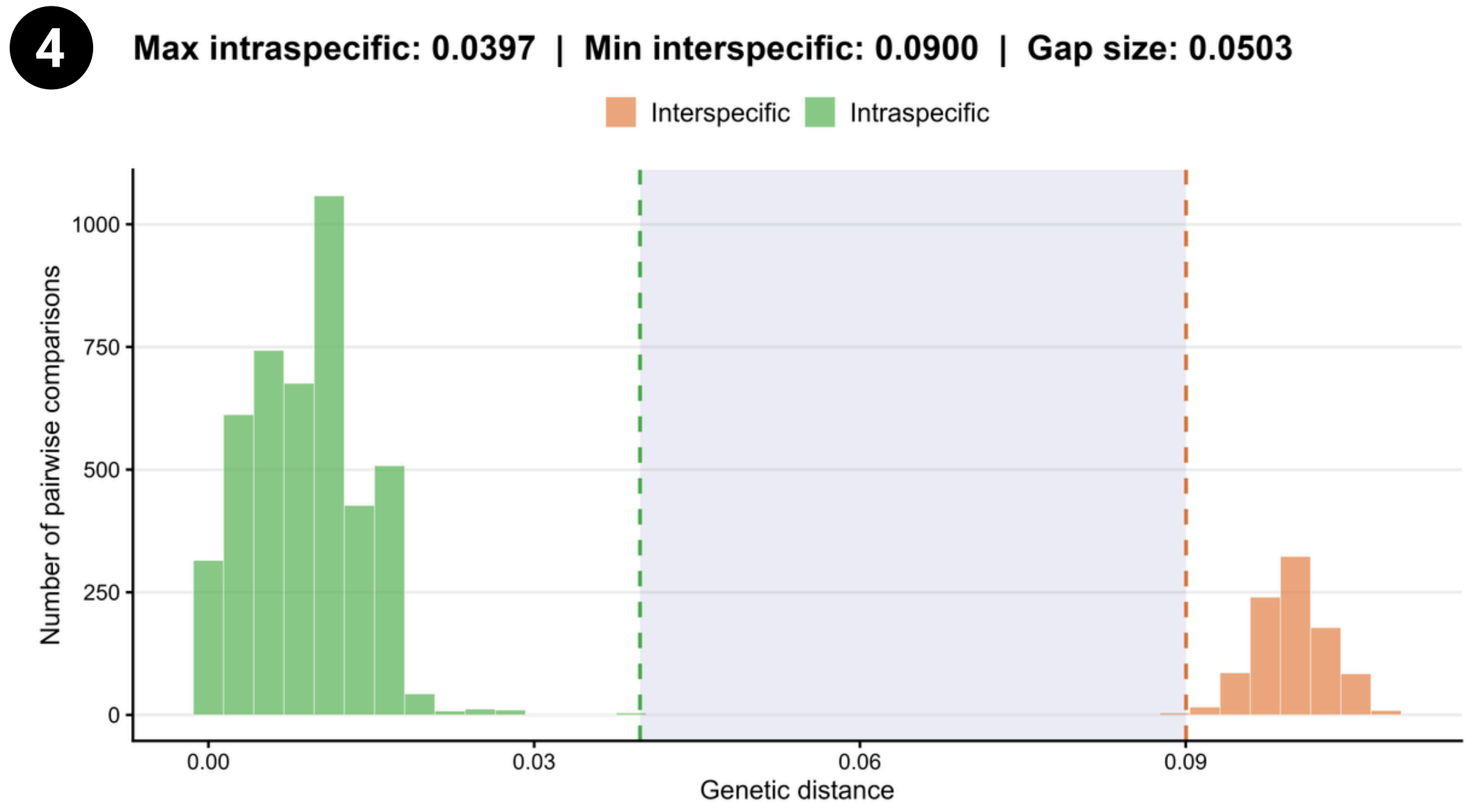

5 COI+CYTB+DBY+Chd1

Biganzoli-Rangel et al., (2023) PloS One

Figure 9

Fig. 9. Phylogenetic, geographic, and supporting evidence for candidate lineages in *Rhynchonycteris naso*. (1) Phylogenetic hypothesis based on COI sequences, with colored clades representing candidate lineages identified through molecular species delimitation. (2) Geographic distribution map, where colored points correspond to the lineages recovered in the phylogeny and black points represent the overall known distribution of the species complex. (3) Table of genetic distances (when sufficient data are available), reporting intra- and inter-lineage divergence among delimited groups. (4) Histogram of genetic distances illustrating the barcode gap. (5) Phylogenetic hypotheses from previous studies (when available), provided for comparison with our proposed delimitations. (6) Additional lines of evidence (occasionally available), such as geometric morphometrics or other independent datasets supporting lineage differentiation. Red star indicates holotype.

Note: *When this symbol (⚠) appears next to the phylogeny, it indicates that the delimitation should be interpreted with caution, as additional sampling is needed. When it appears next to the table, it means that genetic distances are low; therefore, we recommend that these results also be considered with caution. In both cases, we have retained the delimitations as inferred by our methods.*

Saccopteryx bilineata

1

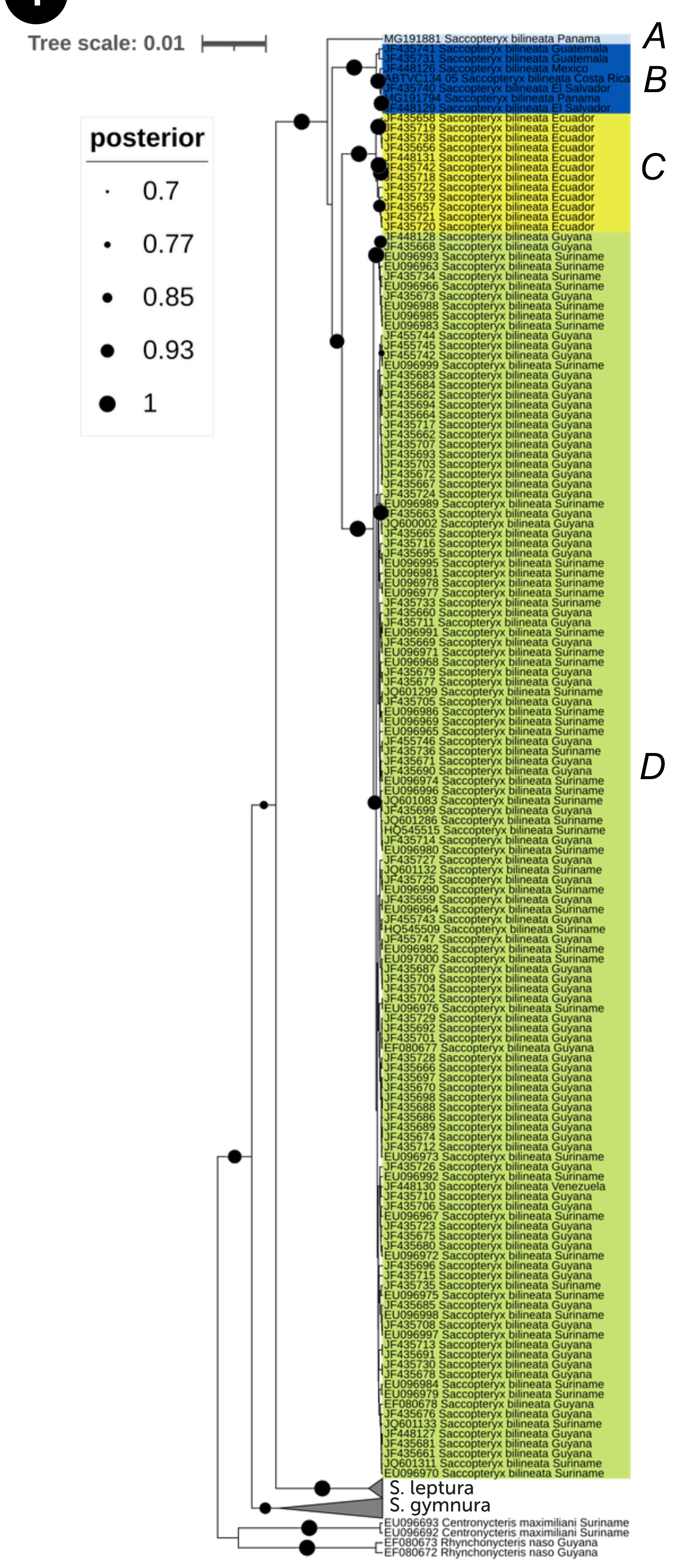

2

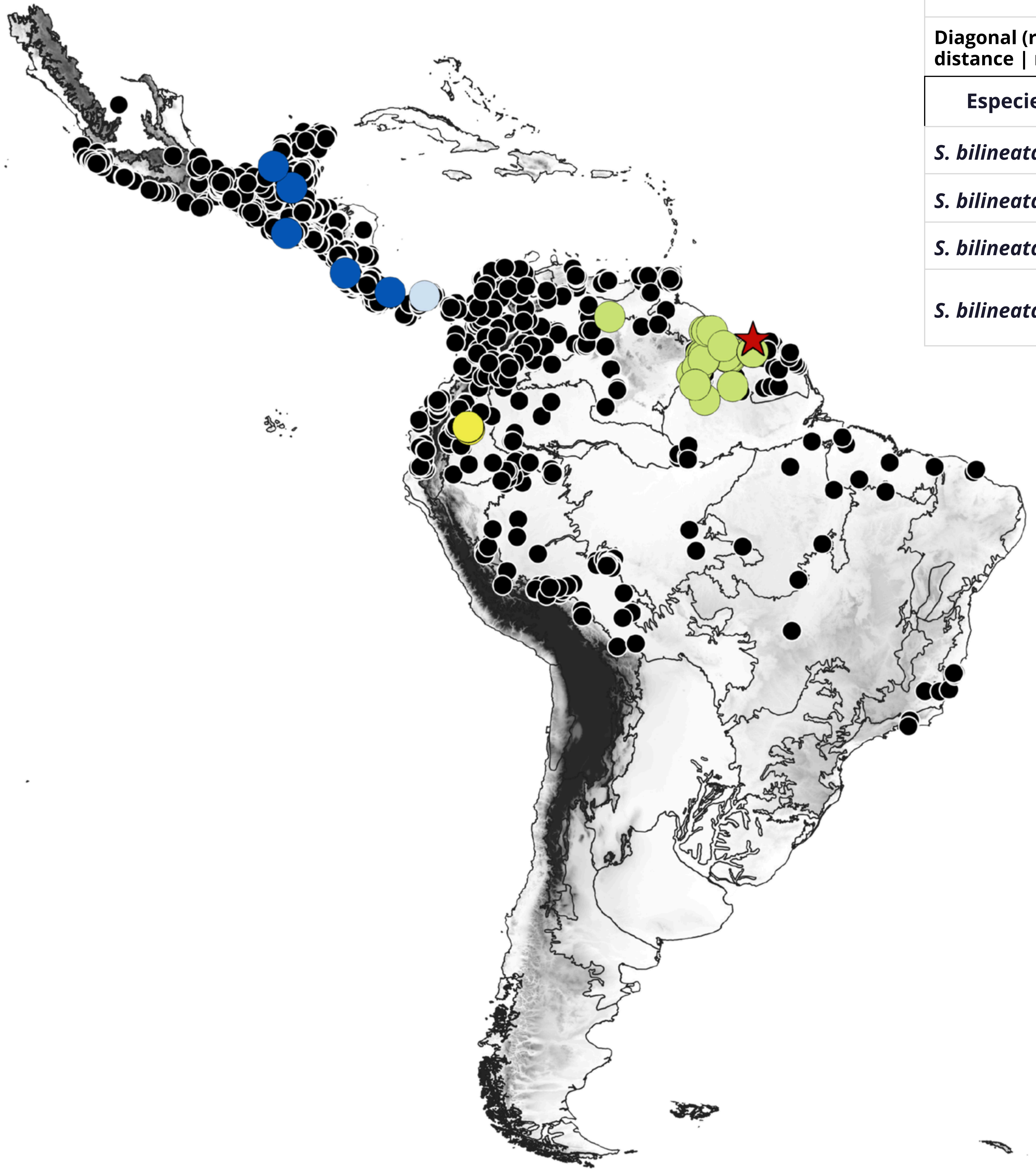

3

Mean genetic distances (Simple Distance)				
Diagonal (red) = mean intraspecific distance | Off-diagonal (black) = mean interspecific distance | n = number of unique pairs				
Especie	*S. bilineata* A	*S. bilineata* B	*S. bilineata* C	*S. bilineata* D
S. bilineata A	--	0.0770 (n=7)	0.0873 (n=12)	0.0980 (n=125)
S. bilineata B	0.0770 (n=7)	0.0055 (n=21)	0.0712 (n=84)	0.0813 (n=875)
S. bilineata C	0.0873 (n=12)	0.0712 (n=84)	0.0072 (n=66)	0.0694 (n=1500)
S. bilineata D	0.0980 (n=125)	0.0813 (n=875)	0.0694 (n=1500)	0.0034 (n=7750)

4 **Max intraspecific: 0.0155 | Min interspecific: 0.0614 | Gap size: 0.0459**

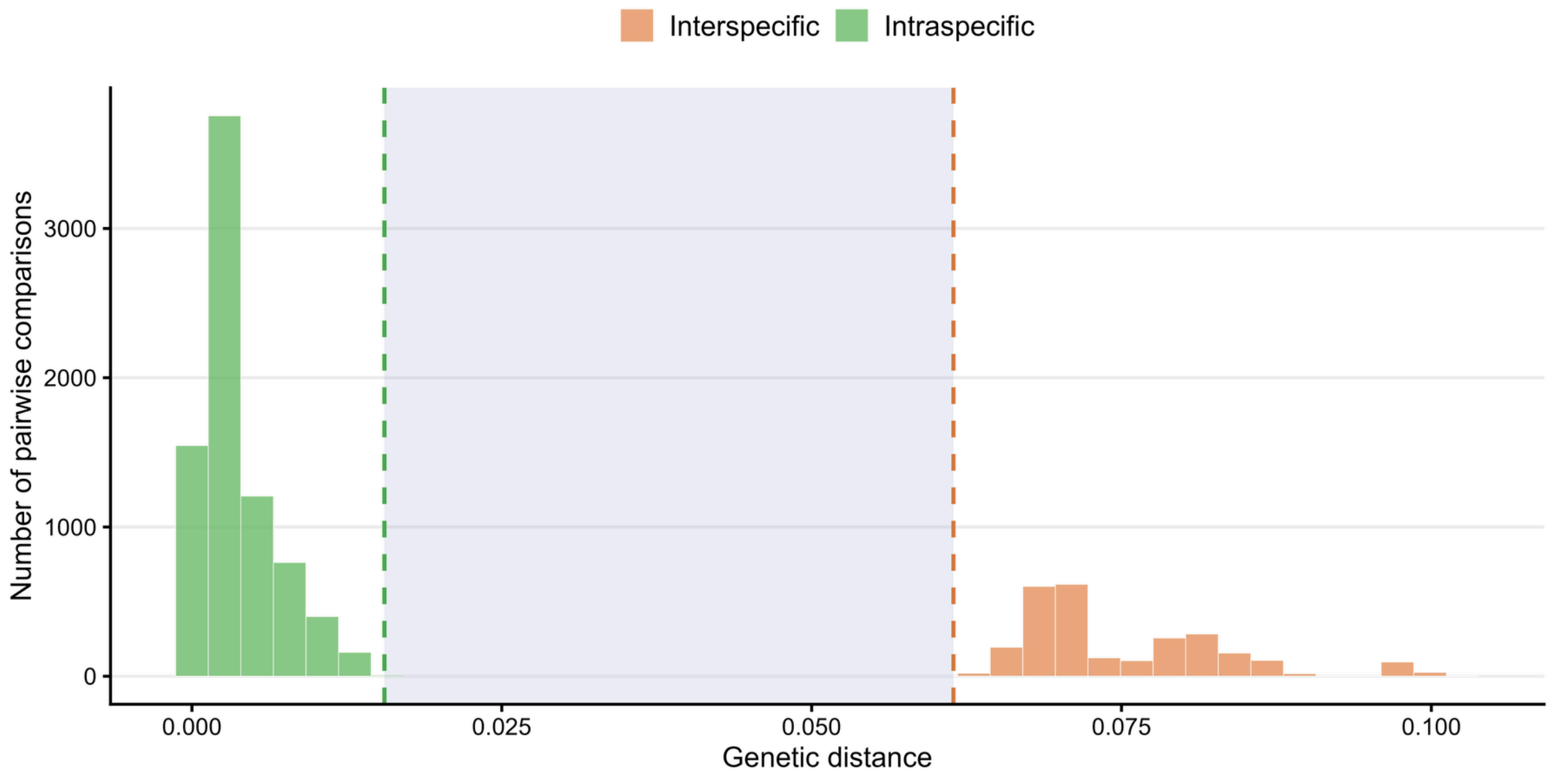

5 COI+DBY

Clare et al., (2011) PloS One

Figure 10

Fig. 10. Phylogenetic, geographic, and supporting evidence for candidate lineages in *Saccopteryx bilineata*. (1) Phylogenetic hypothesis based on COI sequences, with colored clades representing candidate lineages identified through molecular species delimitation. (2) Geographic distribution map, where colored points correspond to the lineages recovered in the phylogeny and black points represent the overall known distribution of the species complex. (3) Table of genetic distances (when sufficient data are available), reporting intra- and inter-lineage divergence among delimited groups. (4) Histogram of genetic distances illustrating the barcode gap. (5) Phylogenetic hypotheses from previous studies (when available), provided for comparison with our proposed delimitations. (6) Additional lines of evidence (occasionally available), such as geometric morphometrics or other independent datasets supporting lineage differentiation. Red star indicates holotype.

Note: *When this symbol (⚠) appears next to the phylogeny, it indicates that the delimitation should be interpreted with caution, as additional sampling is needed. When it appears next to the table, it means that genetic distances are low; therefore, we recommend that these results also be considered with caution. In both cases, we have retained the delimitations as inferred by our methods.*

Anoura caudifer

1

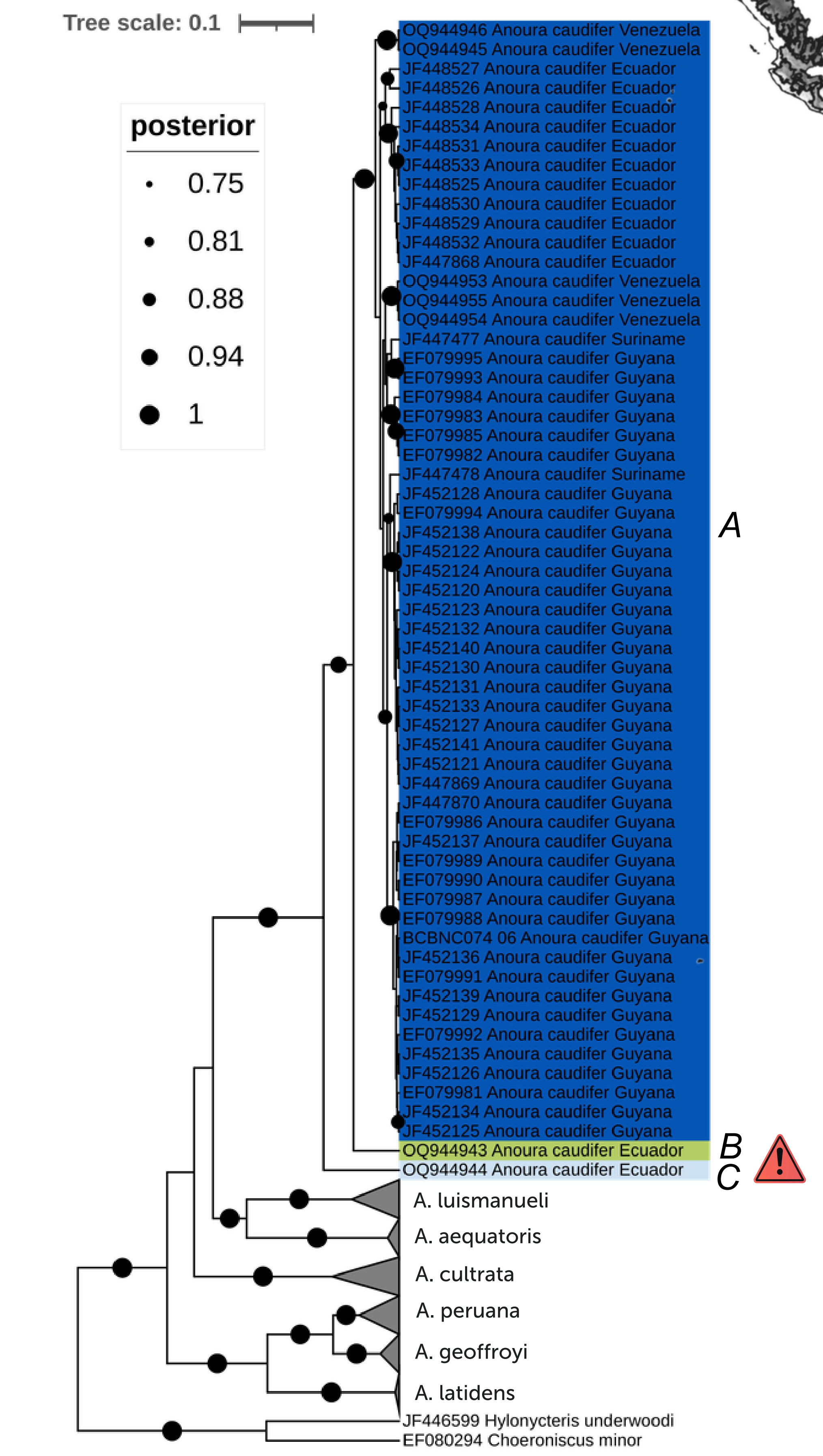

2

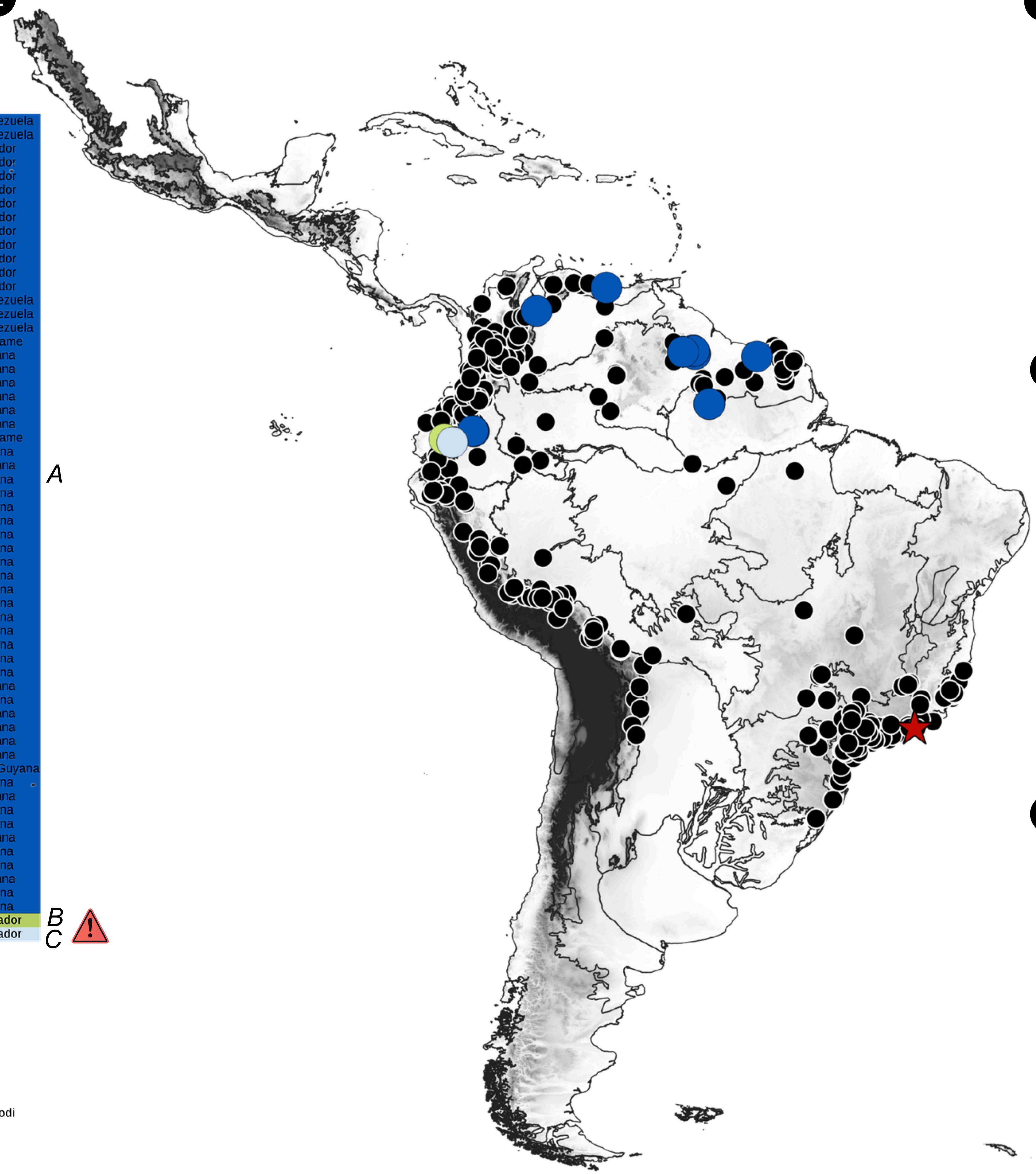

3

Mean genetic distances (Simple Distance)

Diagonal (red) = mean intraspecific distance | Off-diagonal (black) = mean interspecific distance | n = number of unique pairs

Especie	*A. caudifer* A	*A. caudifer* B	*A. caudifer* C
A. caudifer A	0.0115 (n=1653)	0.0437 (n=58)	0.0600 (n=58)
A. caudifer B	0.0437 (n=58)	--	0.0750 (n=1)
A. caudifer C	0.0600 (n=58)	0.0750 (n=1)	--

4

Max intraspecific: 0.0255 | Min interspecific: 0.0319 | Gap size: 0.0064

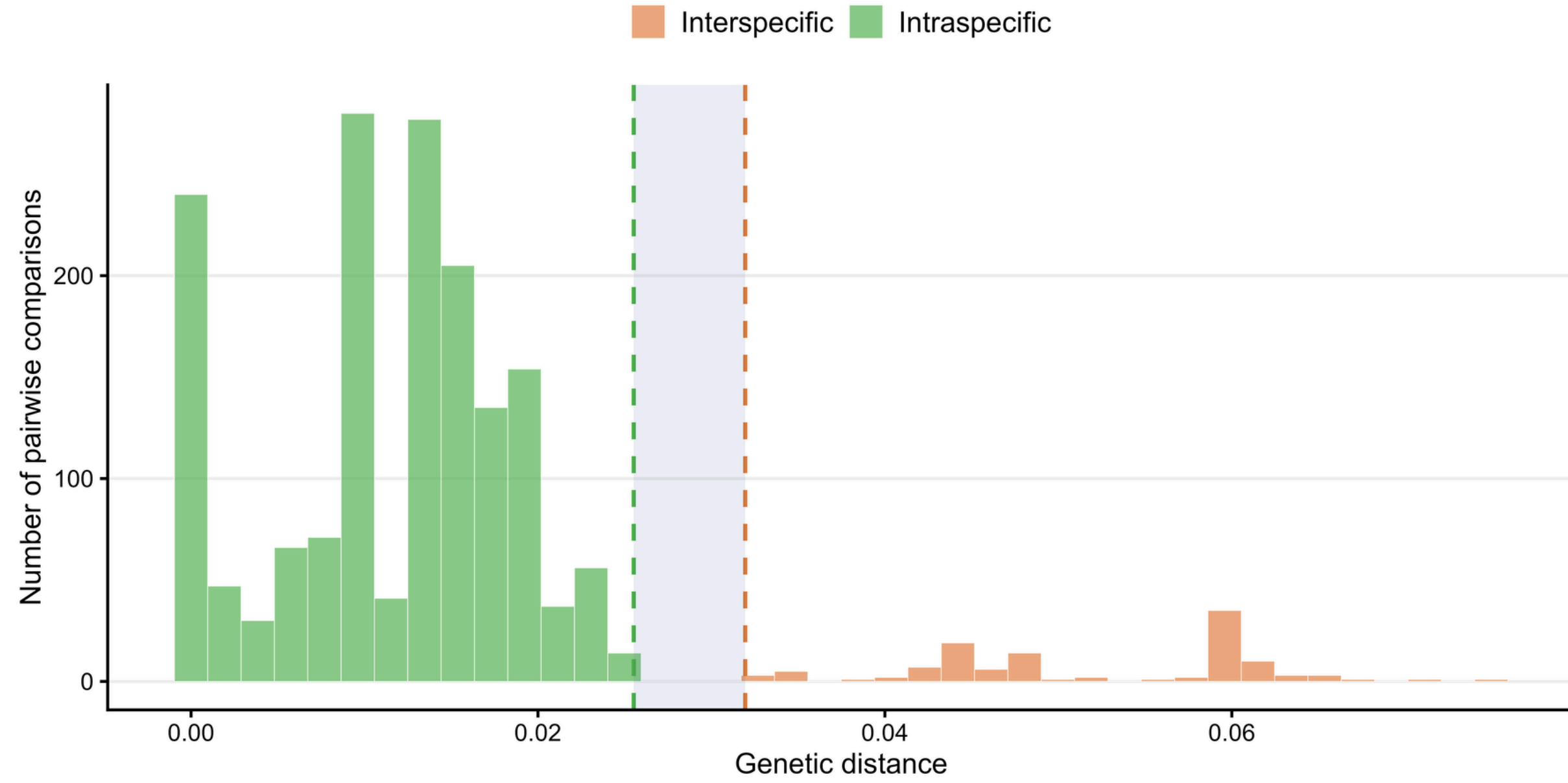

5 COI

Molinari et al., (2023) Zootaxa

Figure 11

Fig. 11. Phylogenetic, geographic, and supporting evidence for candidate lineages in *Anoura caudifer*. (1) Phylogenetic hypothesis based on COI sequences, with colored clades representing candidate lineages identified through molecular species delimitation. (2) Geographic distribution map, where colored points correspond to the lineages recovered in the phylogeny and black points represent the overall known distribution of the species complex. (3) Table of genetic distances (when sufficient data are available), reporting intra- and inter-lineage divergence among delimited groups. (4) Histogram of genetic distances illustrating the barcode gap. (5) Phylogenetic hypotheses from previous studies (when available), provided for comparison with our proposed delimitations. (6) Additional lines of evidence (occasionally available), such as geometric morphometrics or other independent datasets supporting lineage differentiation. Red star indicates holotype.

Note: *When this symbol (⚠) appears next to the phylogeny, it indicates that the delimitation should be interpreted with caution, as additional sampling is needed. When it appears next to the table, it means that genetic distances are low; therefore, we recommend that these results also be considered with caution. In both cases, we have retained the delimitations as inferred by our methods.*

Anoura cultrata

1

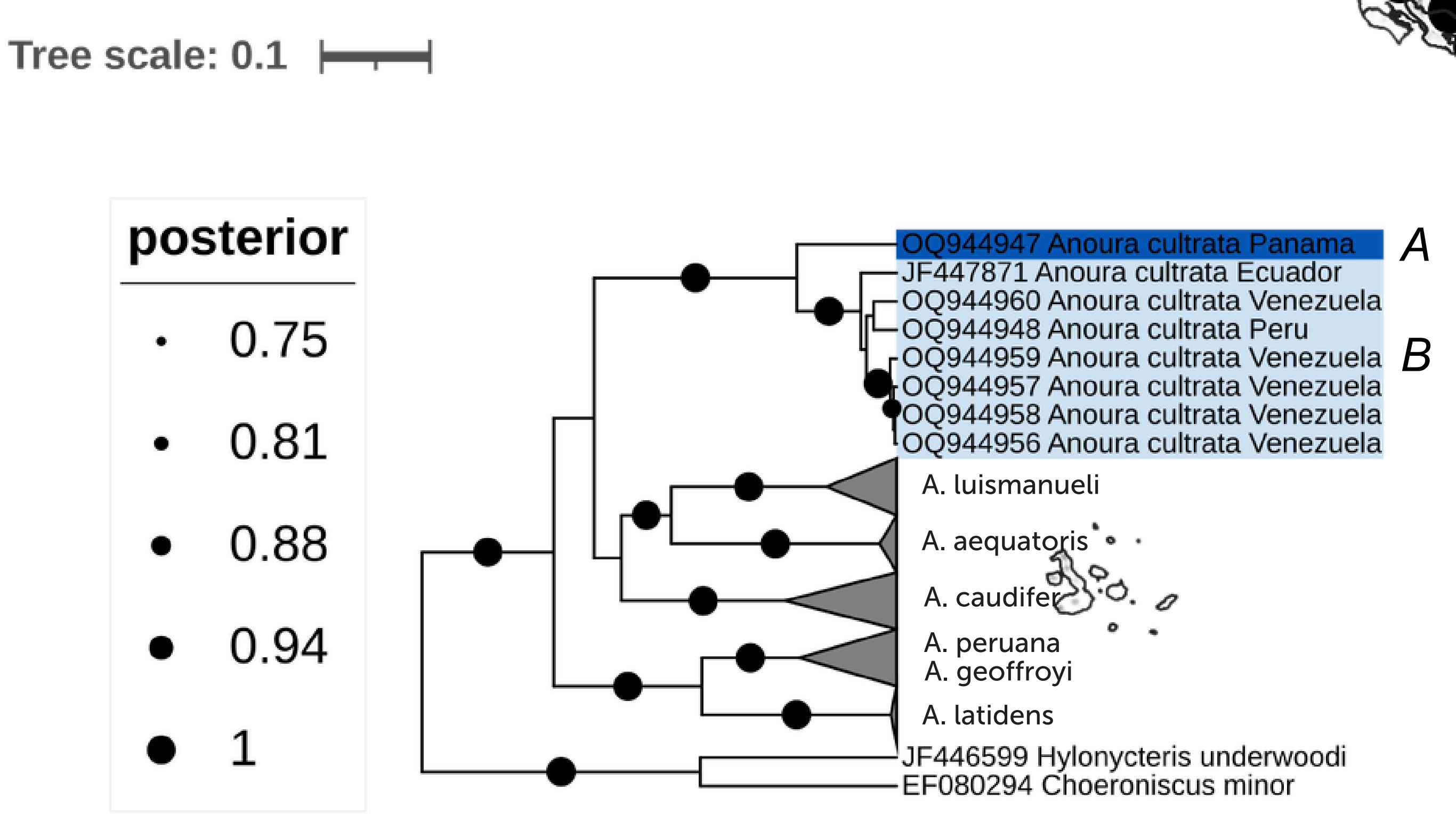

2

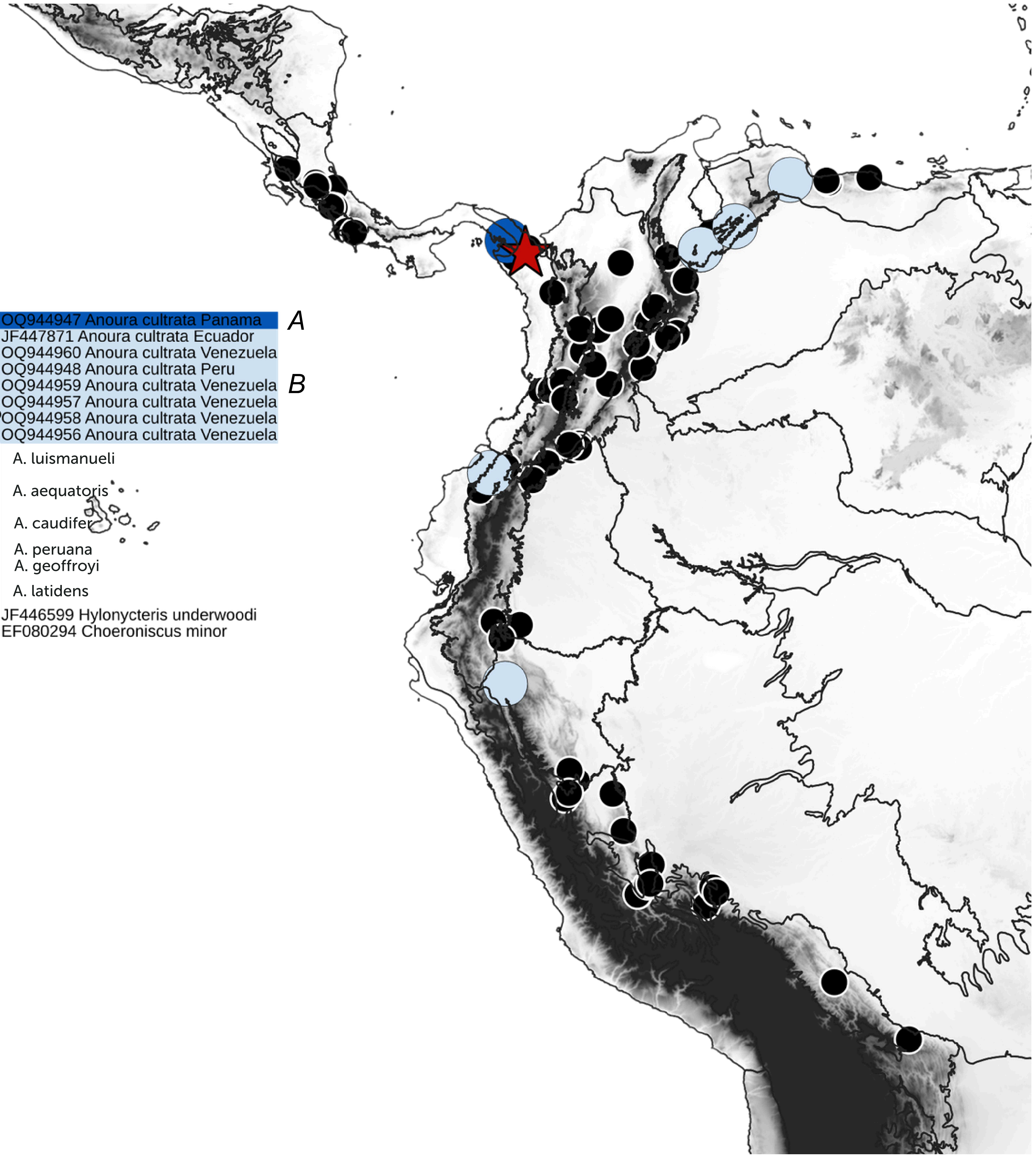

3

Mean genetic distances (Simple Distance)

Diagonal (red) = mean intraspecific distance | Off-diagonal (black) = mean interspecific distance | n = number of unique pairs

Especie	*A. cultrata* A	*A. cultrata* B
A. cultrata A	--	0.0622 (n=7)
A. cultrata B	0.0622 (n=7)	0.0165 (n=21)

4

Max intraspecific: 0.0287 | Min interspecific: 0.0542 | Gap size: 0.0255

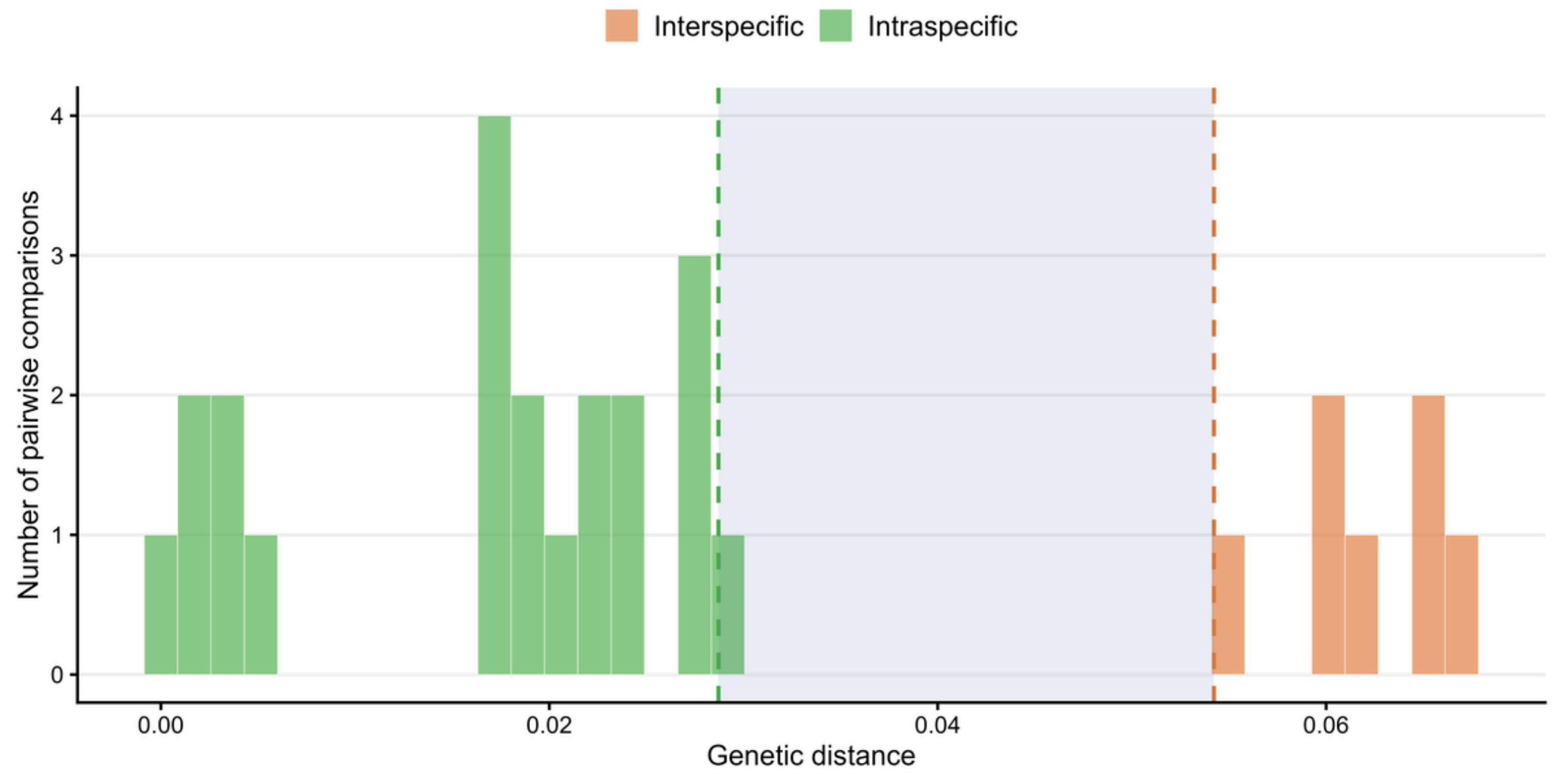

5 COI

Molinari et al., (2023) Zootaxa

Figure 12

Fig. 12. Phylogenetic, geographic, and supporting evidence for candidate lineages in *Anoura cultrata*. (1) Phylogenetic hypothesis based on COI sequences, with colored clades representing candidate lineages identified through molecular species delimitation. (2) Geographic distribution map, where colored points correspond to the lineages recovered in the phylogeny and black points represent the overall known distribution of the species complex. (3) Table of genetic distances (when sufficient data are available), reporting intra- and inter-lineage divergence among delimited groups. (4) Histogram of genetic distances illustrating the barcode gap. (5) Phylogenetic hypotheses from previous studies (when available), provided for comparison with our proposed delimitations. (6) Additional lines of evidence (occasionally available), such as geometric morphometrics or other independent datasets supporting lineage differentiation. Red star indicates holotype.

Note: *When this symbol (⚠) appears next to the phylogeny, it indicates that the delimitation should be interpreted with caution, as additional sampling is needed. When it appears next to the table, it means that genetic distances are low; therefore, we recommend that these results also be considered with caution. In both cases, we have retained the delimitations as inferred by our methods.*

Anoura luismanueli

1

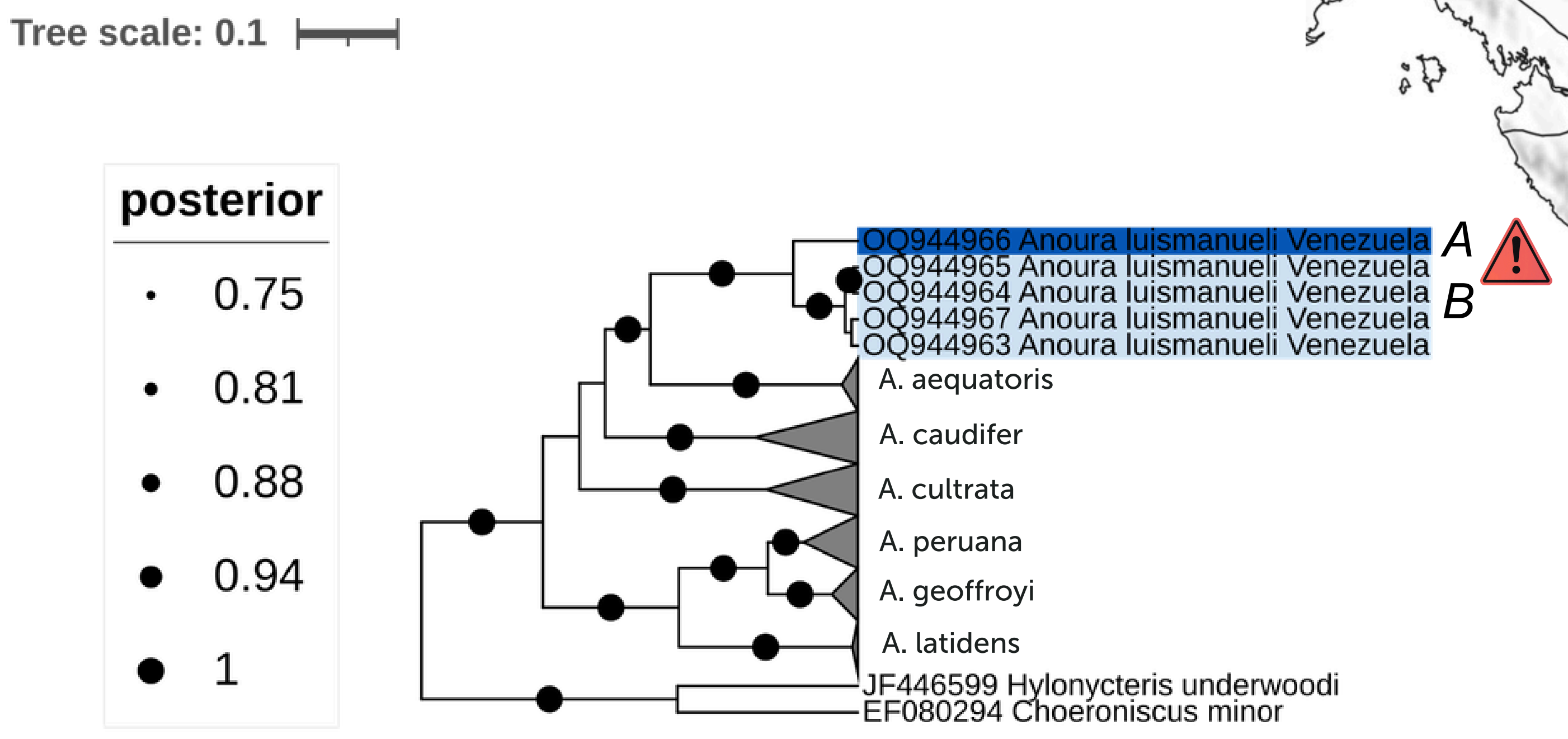

2

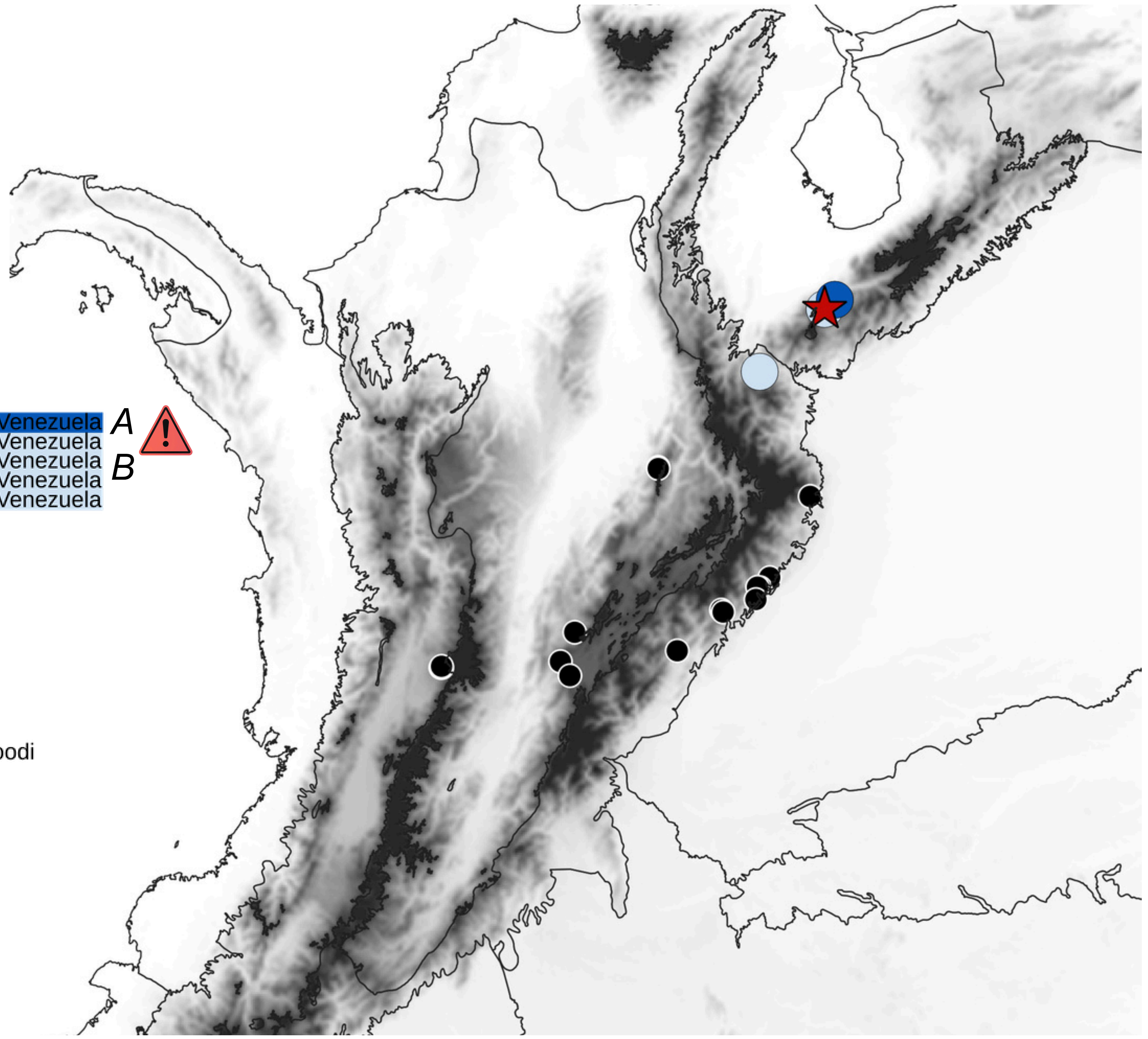

3

Mean genetic distances (Simple Distance)

Diagonal (red) = mean intraspecific distance | Off-diagonal (black) = mean interspecific distance | n = number of unique pairs

Especie	*A. luismanueli* A	*A. luismanueli* B
A. luismanueli A	--	0.0504 (n=4)
A. luismanueli B	0.0504 (n=4)	0.0072 (n=6)

4 **Max intraspecific: 0.0128 | Min interspecific: 0.0468 | Gap size: 0.0340**

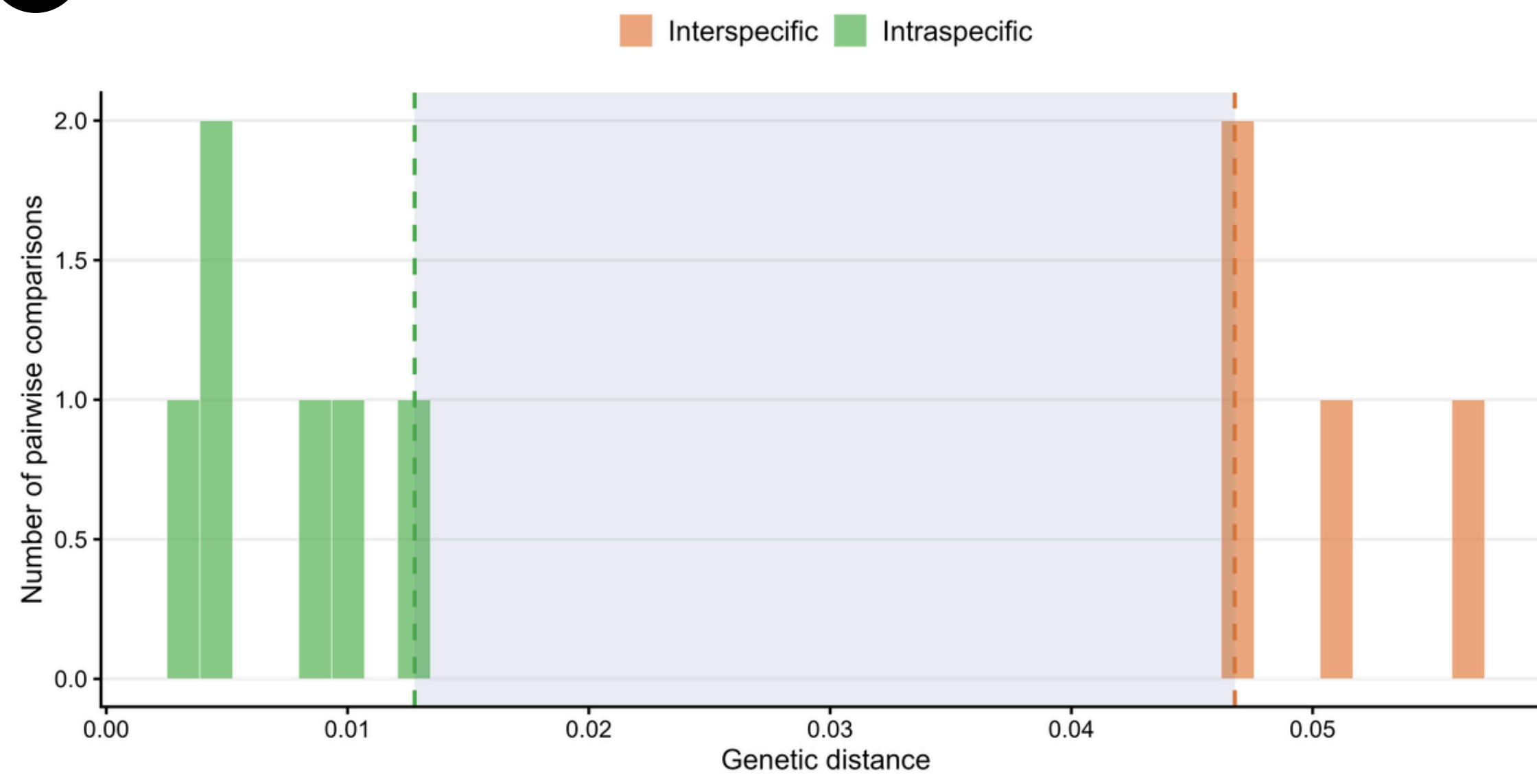

5 COI

Molinari et al., (2023) Zootaxa

Figure 13

Fig. 13. Phylogenetic, geographic, and supporting evidence for candidate lineages in *Anoura luismanueli.* (1) Phylogenetic hypothesis based on COI sequences, with colored clades representing candidate lineages identified through molecular species delimitation. (2) Geographic distribution map, where colored points correspond to the lineages recovered in the phylogeny and black points represent the overall known distribution of the species complex. (3) Table of genetic distances (when sufficient data are available), reporting intra- and inter-lineage divergence among delimited groups. (4) Histogram of genetic distances illustrating the barcode gap. (5) Phylogenetic hypotheses from previous studies (when available), provided for comparison with our proposed delimitations. (6) Additional lines of evidence (occasionally available), such as geometric morphometrics or other independent datasets supporting lineage differentiation. Red star indicates holotype.

Note: *When this symbol (⚠) appears next to the phylogeny, it indicates that the delimitation should be interpreted with caution, as additional sampling is needed. When it appears next to the table, it means that genetic distances are low; therefore, we recommend that these results also be considered with caution. In both cases, we have retained the delimitations as inferred by our methods.*

Anoura peruana

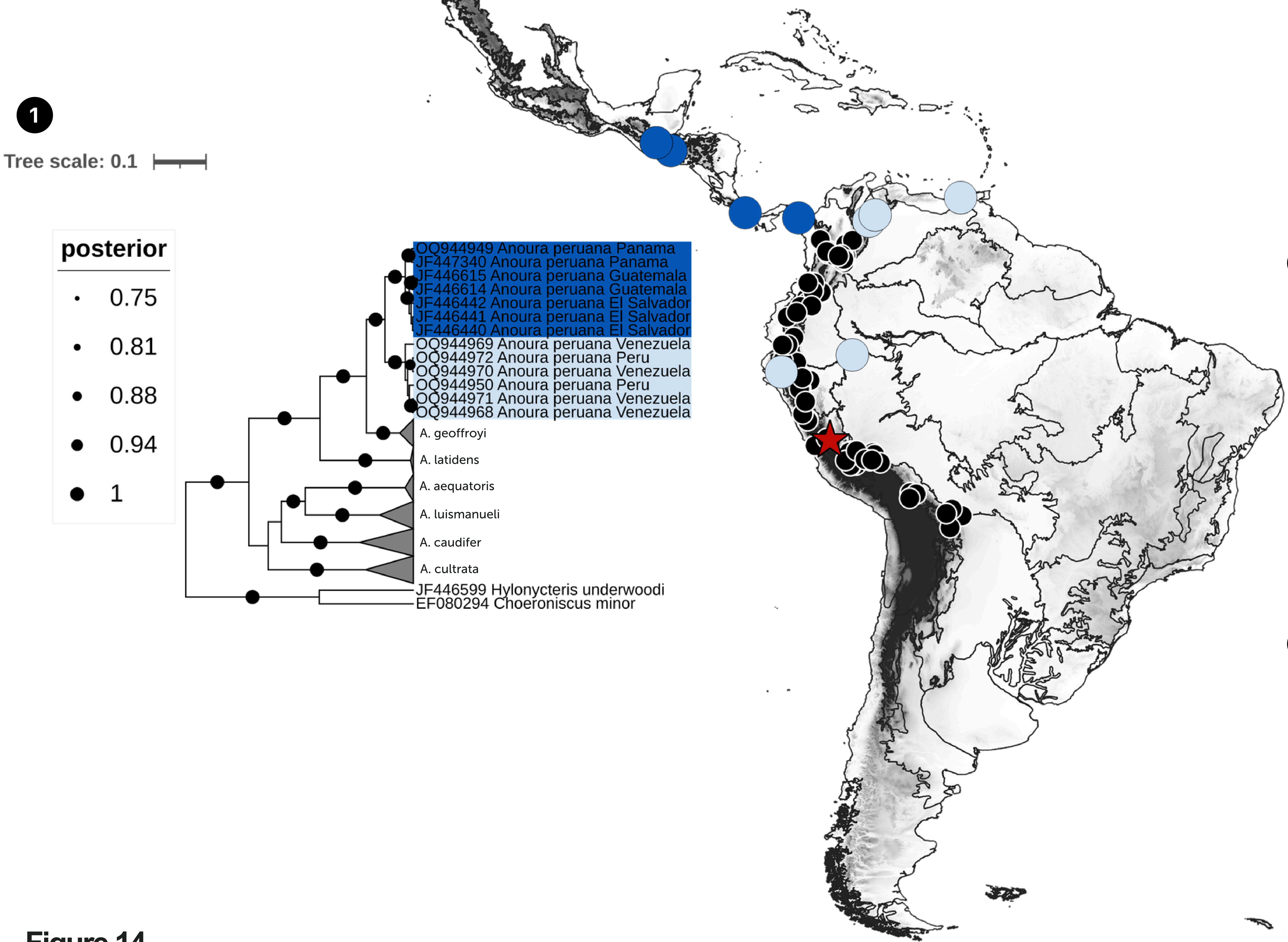

3

Mean genetic distances (Simple Distance)

Diagonal (red) = mean intraspecific distance | Off-diagonal (black) = mean interspecific distance | n = number of unique pairs

Especie	*A. peruana* A	*A. peruana* B
A. peruana A	0.0074 (n=21)	0.0389 (n=42)
A. peruana B	0.0389 (n=42)	0.0060 (n=15)

4

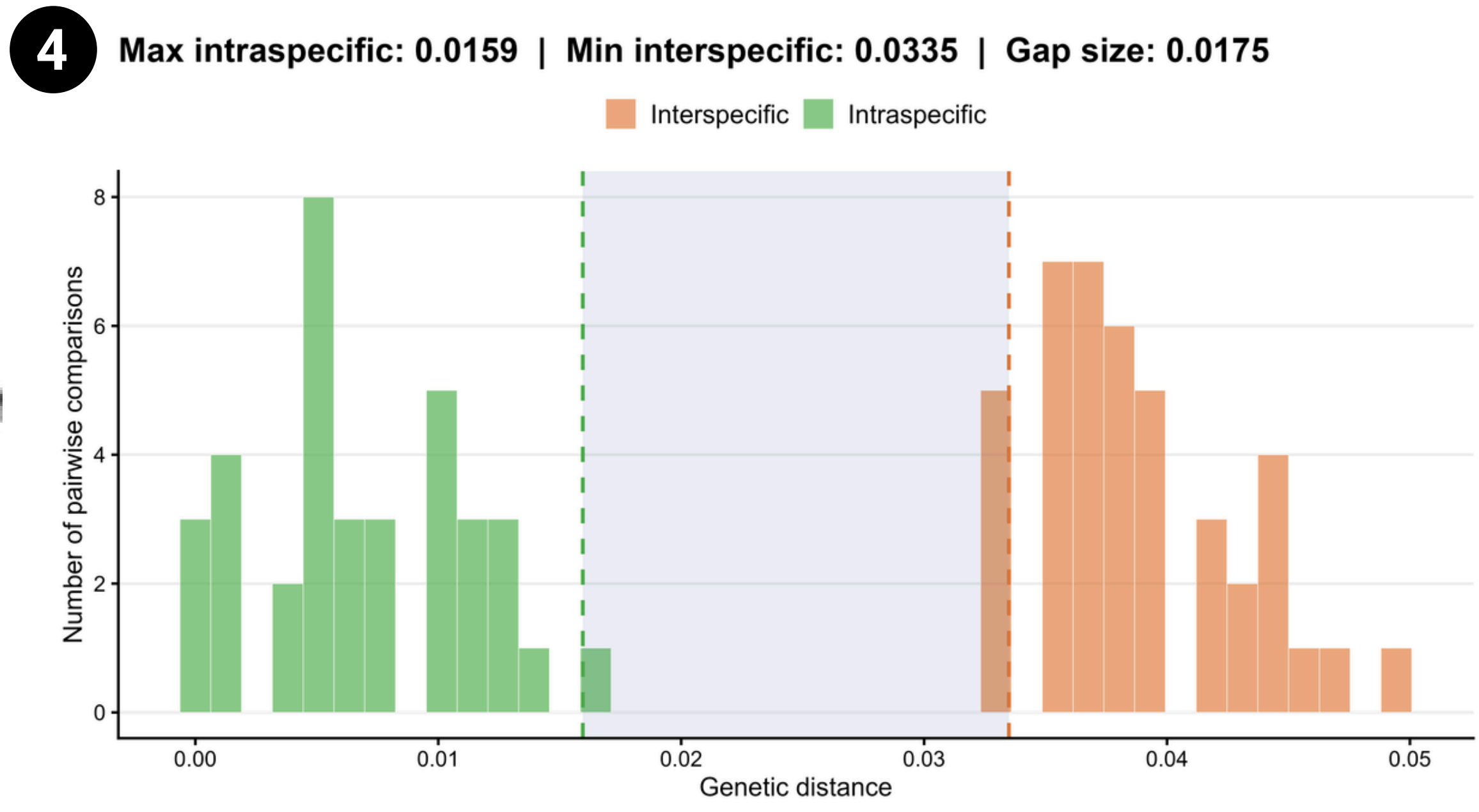

5 COI

Molinari et al., (2023) Zootaxa

Figure 14

Fig. 14. Phylogenetic, geographic, and supporting evidence for candidate lineages in *Anoura peruana*. (1) Phylogenetic hypothesis based on COI sequences, with colored clades representing candidate lineages identified through molecular species delimitation. (2) Geographic distribution map, where colored points correspond to the lineages recovered in the phylogeny and black points represent the overall known distribution of the species complex. (3) Table of genetic distances (when sufficient data are available), reporting intra- and inter-lineage divergence among delimited groups. (4) Histogram of genetic distances illustrating the barcode gap. (5) Phylogenetic hypotheses from previous studies (when available), provided for comparison with our proposed delimitations. (6) Additional lines of evidence (occasionally available), such as geometric morphometrics or other independent datasets supporting lineage differentiation. Red star indicates holotype.

Note: *When this symbol (⚠) appears next to the phylogeny, it indicates that the delimitation should be interpreted with caution, as additional sampling is needed. When it appears next to the table, it means that genetic distances are low; therefore, we recommend that these results also be considered with caution. In both cases, we have retained the delimitations as inferred by our methods.*

Carollia castanea

1

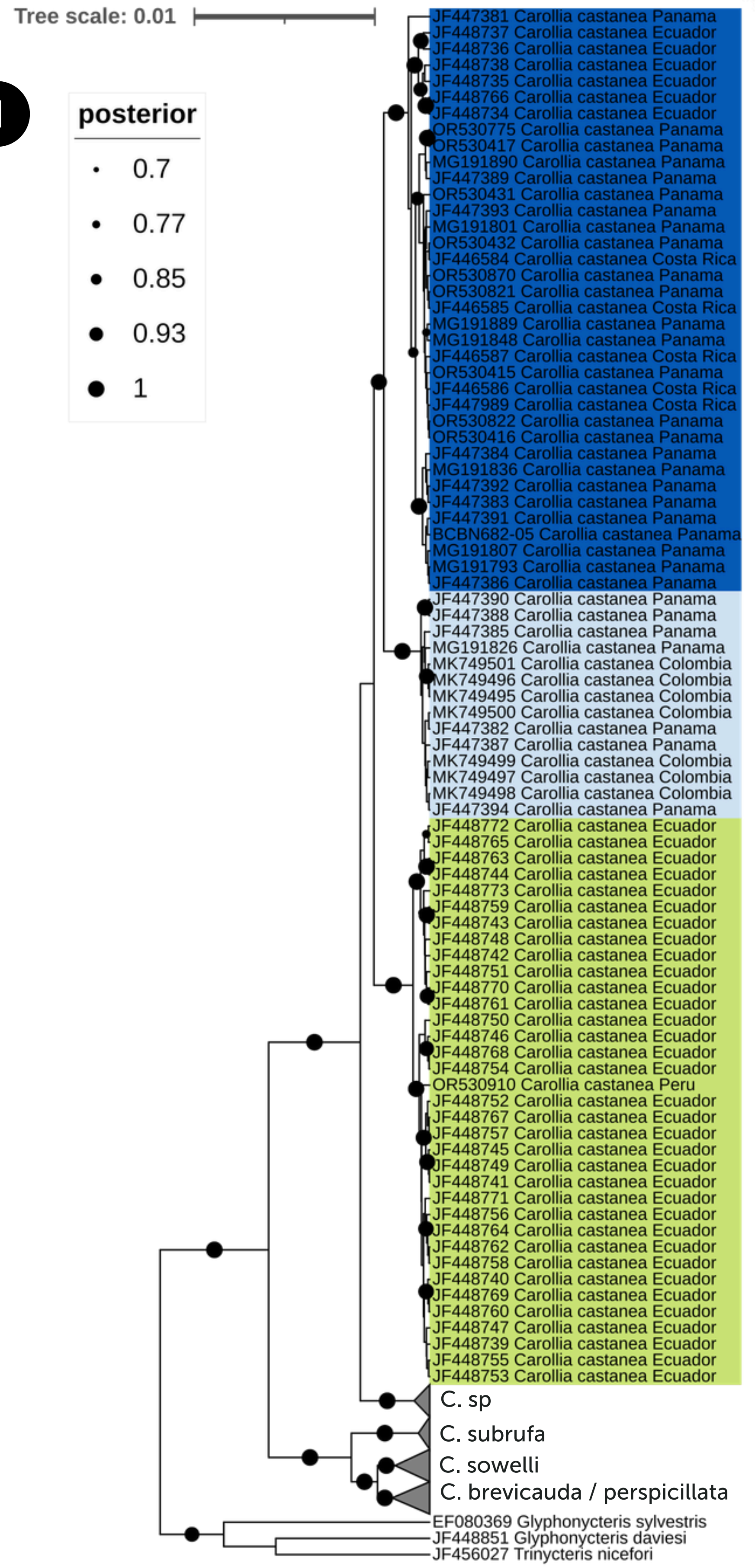

2

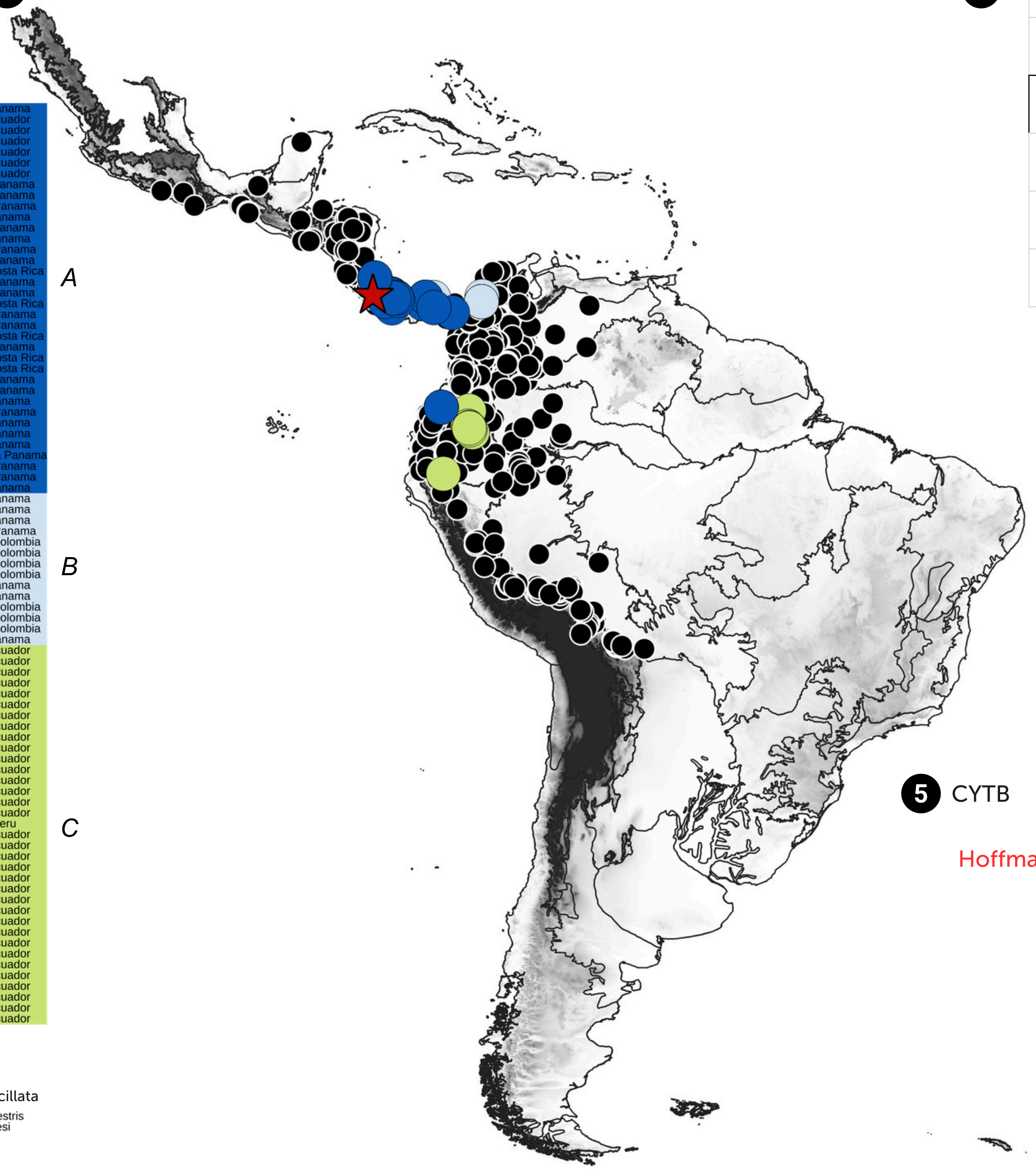

3

Mean genetic distances (Simple Distance)

Diagonal (red) = mean intraspecific distance | Off-diagonal (black) = mean interspecific distance | n = number of unique pairs

Especie	*C. castanea* A	*C. castanea* B	*C. castanea* C
C. castanea A	0.0083 (n=630)	0.0456 (n=504)	0.0535 (n=1260)
C. castanea B	0.0456 (n=504)	0.0021 (n=91)	0.0539 (n=490)
C. castanea C	0.0535 (n=1260)	0.0539 (n=490)	0.0065 (n=595)

4

Max intraspecific: 0.0229 | Min interspecific: 0.0382 | Gap size: 0.0153

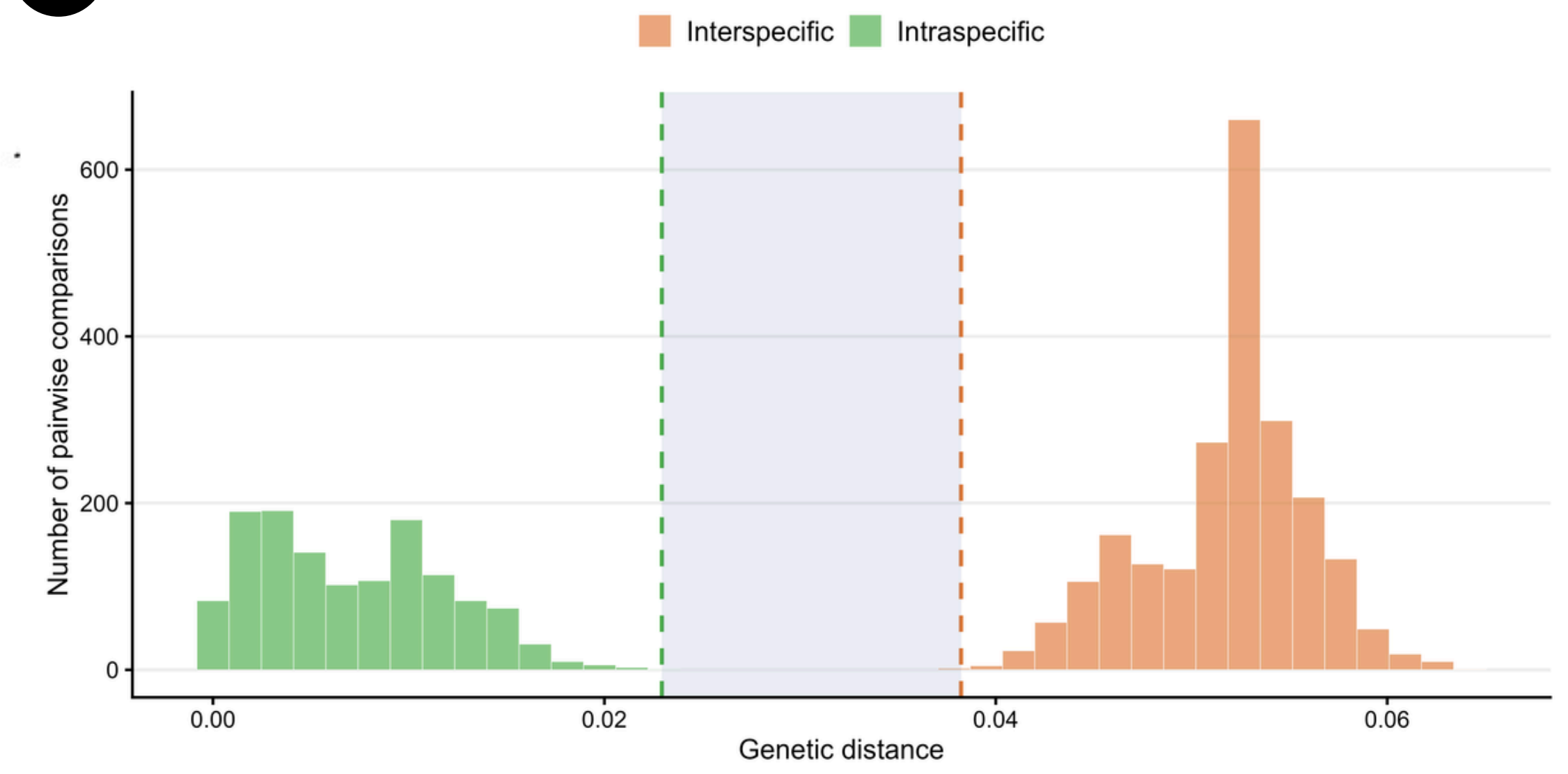

5 CYTB

COI

Hoffman & Baker (2003) Mol. Ecol.

Clare et al., (2011) PloS One

Figure 15

Fig. 15. Phylogenetic, geographic, and supporting evidence for candidate lineages in *Carollia castanea*. (1) Phylogenetic hypothesis based on COI sequences, with colored clades representing candidate lineages identified through molecular species delimitation. (2) Geographic distribution map, where colored points correspond to the lineages recovered in the phylogeny and black points represent the overall known distribution of the species complex. (3) Table of genetic distances (when sufficient data are available), reporting intra- and inter-lineage divergence among delimited groups. (4) Histogram of genetic distances illustrating the barcode gap. (5) Phylogenetic hypotheses from previous studies (when available), provided for comparison with our proposed delimitations. (6) Additional lines of evidence (occasionally available), such as geometric morphometrics or other independent datasets supporting lineage differentiation. Red star indicates holotype.

Note: *When this symbol (⚠) appears next to the phylogeny, it indicates that the delimitation should be interpreted with caution, as additional sampling is needed. When it appears next to the table, it means that genetic distances are low; therefore, we recommend that these results also be considered with caution. In both cases, we have retained the delimitations as inferred by our methods.*

Choeroniscus minor

1

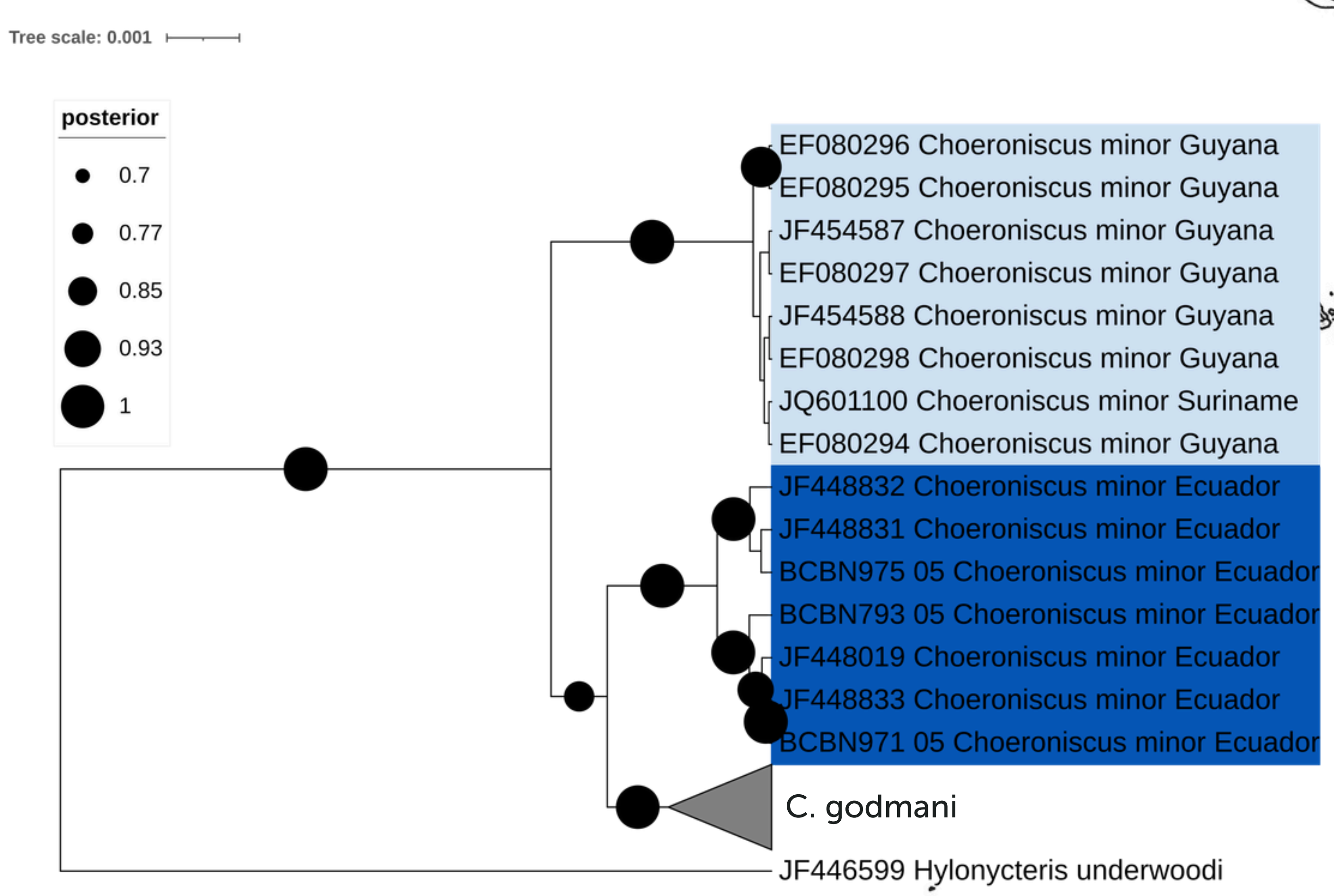

2

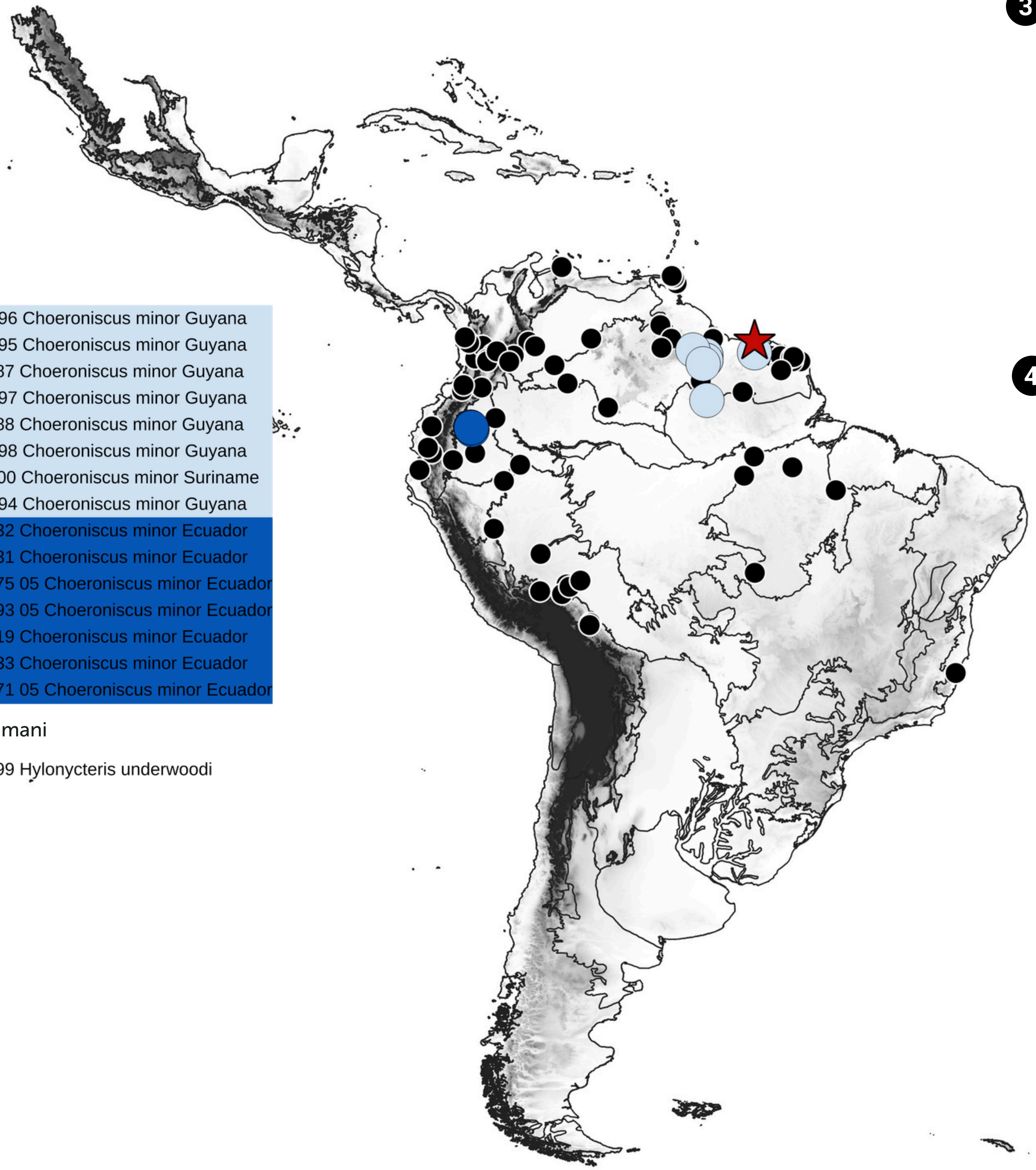

3

Mean genetic distances (Simple Distance)

Diagonal (red) = mean intraspecific distance | Off-diagonal (black) = mean interspecific distance | n = number of unique pairs

Especie	*C. minor* A	*C. minor* B
C. minor A	0.0007 (n=28)	0.0445 (n=56)
C. minor B	0.0445 (n=56)	0.0083 (n=21)

4

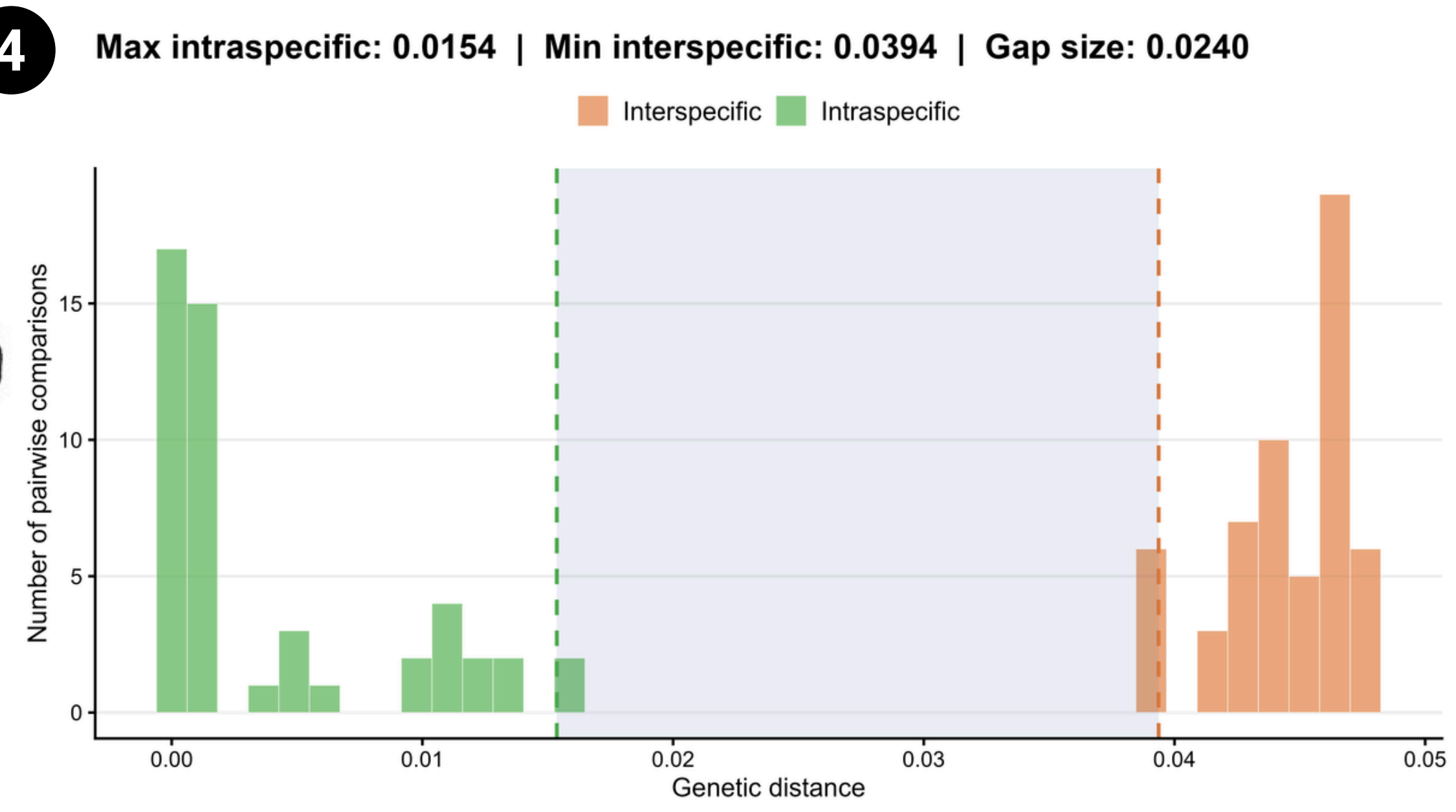

Figure 16

Fig. 16. Phylogenetic, geographic, and supporting evidence for candidate lineages in *Choeroniscus minor*. (1) Phylogenetic hypothesis based on COI sequences, with colored clades representing candidate lineages identified through molecular species delimitation. (2) Geographic distribution map, where colored points correspond to the lineages recovered in the phylogeny and black points represent the overall known distribution of the species complex. (3) Table of genetic distances (when sufficient data are available), reporting intra- and inter-lineage divergence among delimited groups. (4) Histogram of genetic distances illustrating the barcode gap. (5) Phylogenetic hypotheses from previous studies (when available), provided for comparison with our proposed delimitations. (6) Additional lines of evidence (occasionally available), such as geometric morphometrics or other independent datasets supporting lineage differentiation. Red star indicates holotype.

Note: *When this symbol ( ) appears next to the phylogeny, it indicates that the delimitation should be interpreted with caution, as additional sampling is needed. When it appears next to the table, it means that genetic distances are low; therefore, we recommend that these results also be considered with caution. In both cases, we have retained the delimitations as inferred by our methods.*

Chrotopterus auritus

1

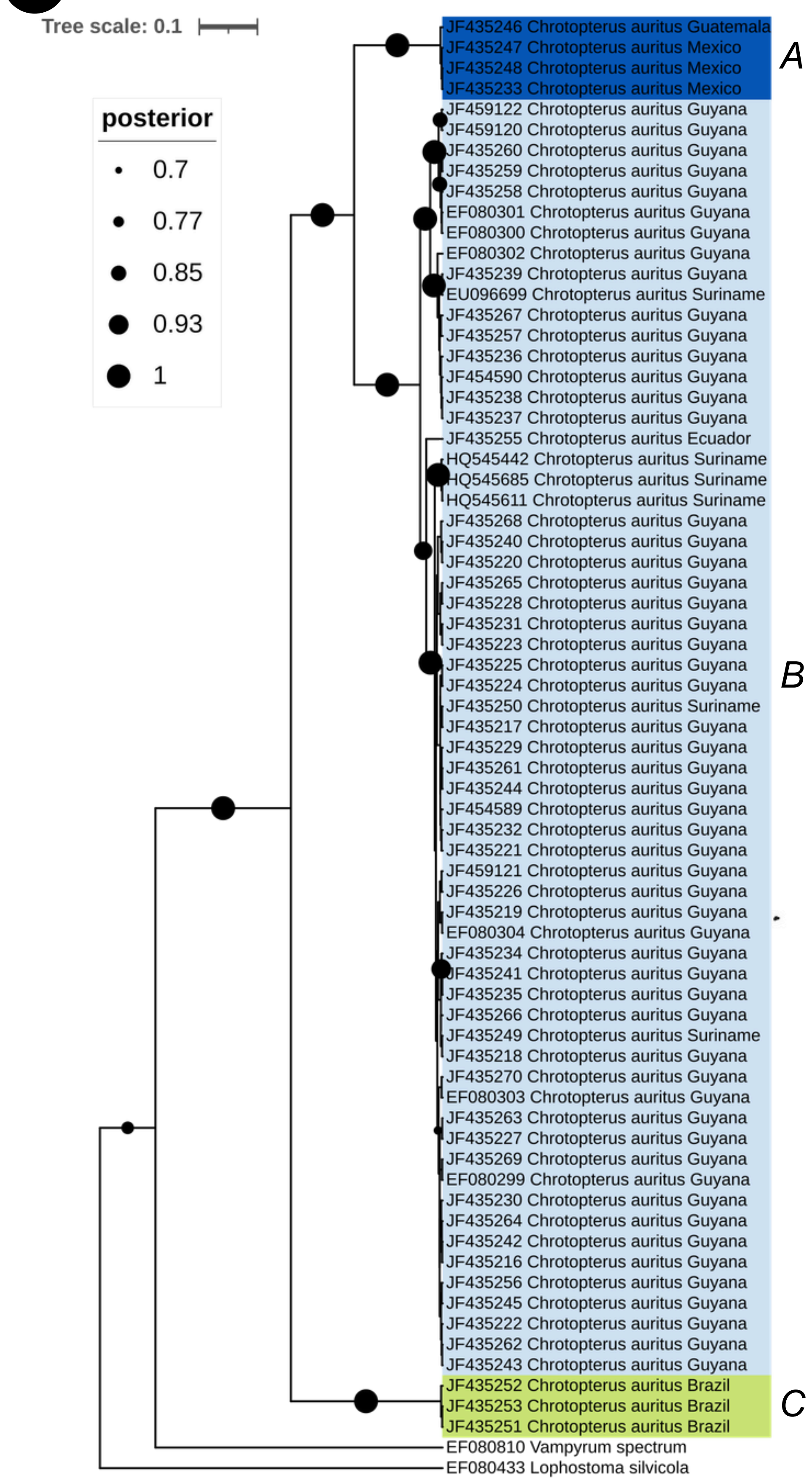

2

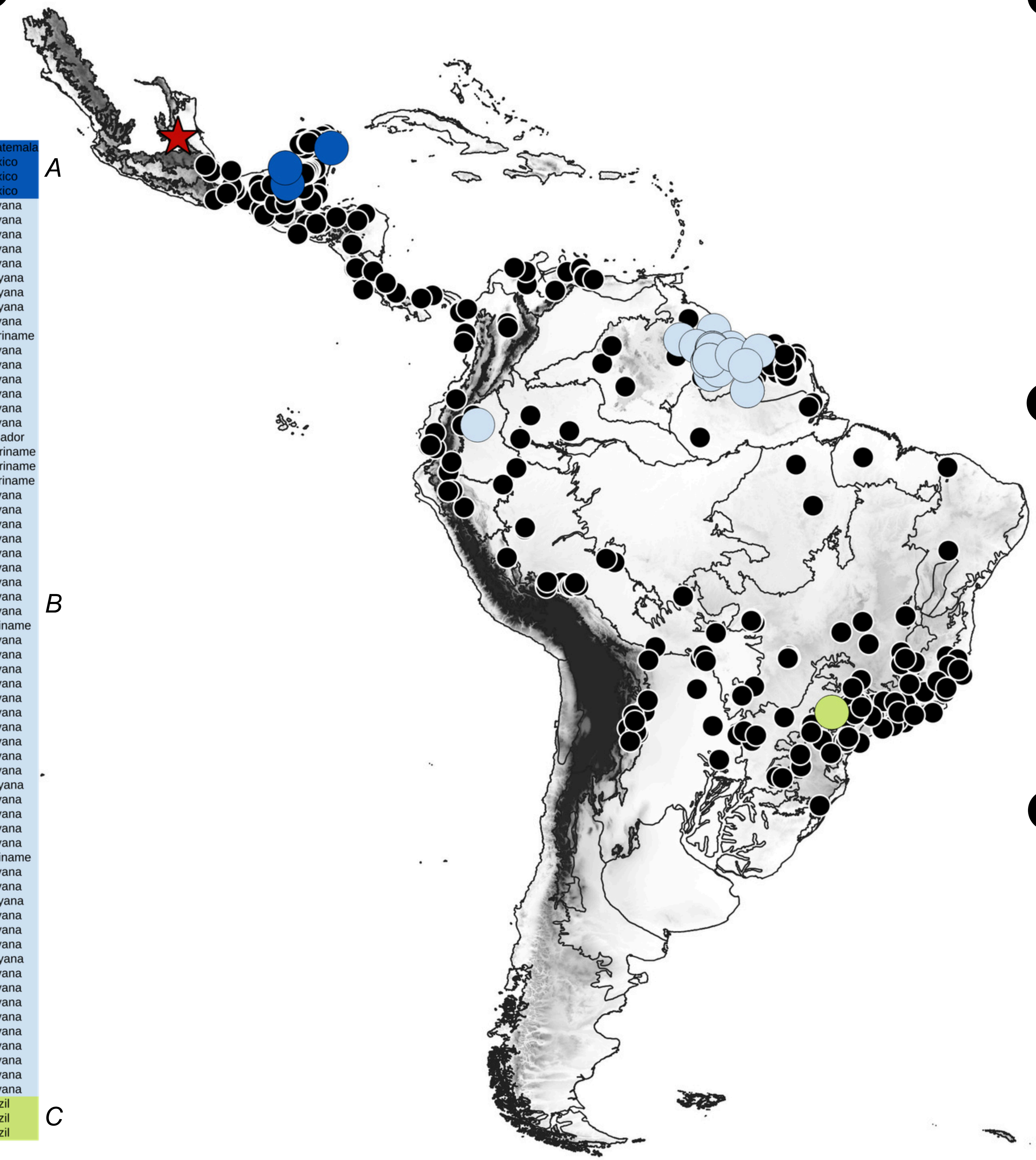

3

Mean genetic distances (Simple Distance)			
Diagonal (red) = mean intraspecific distance | Off-diagonal (black) = mean interspecific distance | n = number of unique pairs			
**Especie**	**C. *auritus* A**	**C. *auritus* B**	**C. *auritus* C**
**C. *auritus* A**	0.0010 (n=6)	0.0922 (n=248)	0.1147 (n=12)
**C. *auritus* B**	0.0922 (n=248)	0.0111 (n=1891)	0.1335 (n=186)
**C. *auritus* C**	0.1147 (n=12)	0.1335 (n=186)	0.0000 (n=3)

4

Max intraspecific: 0.0299 | Min interspecific: 0.0841 | Gap size: 0.0542

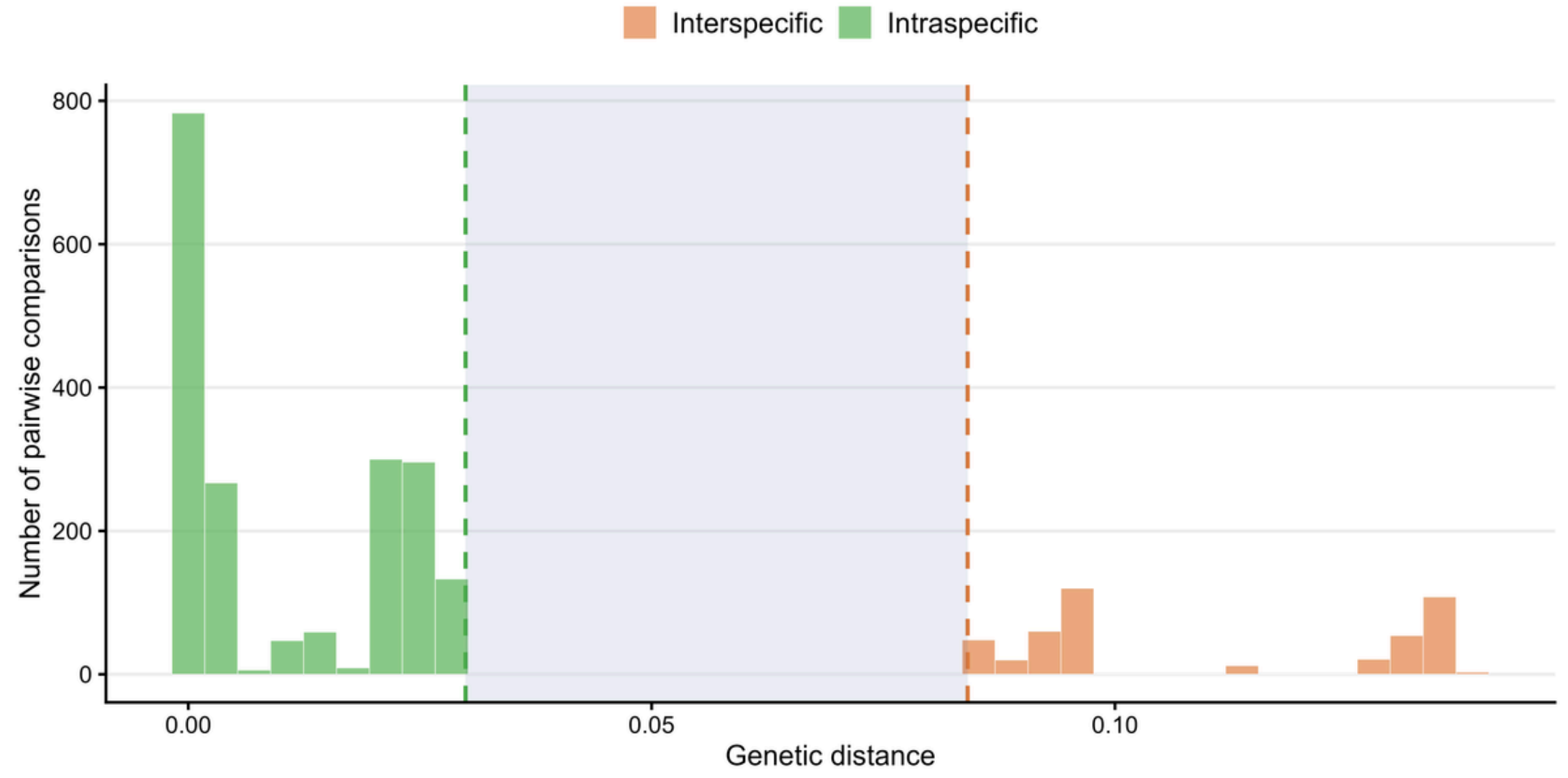

5 COI+DBY

Clare et al., (2011) PloS One

Figure 17

Fig. 17. Phylogenetic, geographic, and supporting evidence for candidate lineages in *Chrotopterus auritus.* (1) Phylogenetic hypothesis based on COI sequences, with colored clades representing candidate lineages identified through molecular species delimitation. (2) Geographic distribution map, where colored points correspond to the lineages recovered in the phylogeny and black points represent the overall known distribution of the species complex. (3) Table of genetic distances (when sufficient data are available), reporting intra- and inter-lineage divergence among delimited groups. (4) Histogram of genetic distances illustrating the barcode gap. (5) Phylogenetic hypotheses from previous studies (when available), provided for comparison with our proposed delimitations. (6) Additional lines of evidence (occasionally available), such as geometric morphometrics or other independent datasets supporting lineage differentiation. Red star indicates holotype.

Note: *When this symbol (⚠) appears next to the phylogeny, it indicates that the delimitation should be interpreted with caution, as additional sampling is needed. When it appears next to the table, it means that genetic distances are low; therefore, we recommend that these results also be considered with caution. In both cases, we have retained the delimitations as inferred by our methods.*

Dermanura anderseni

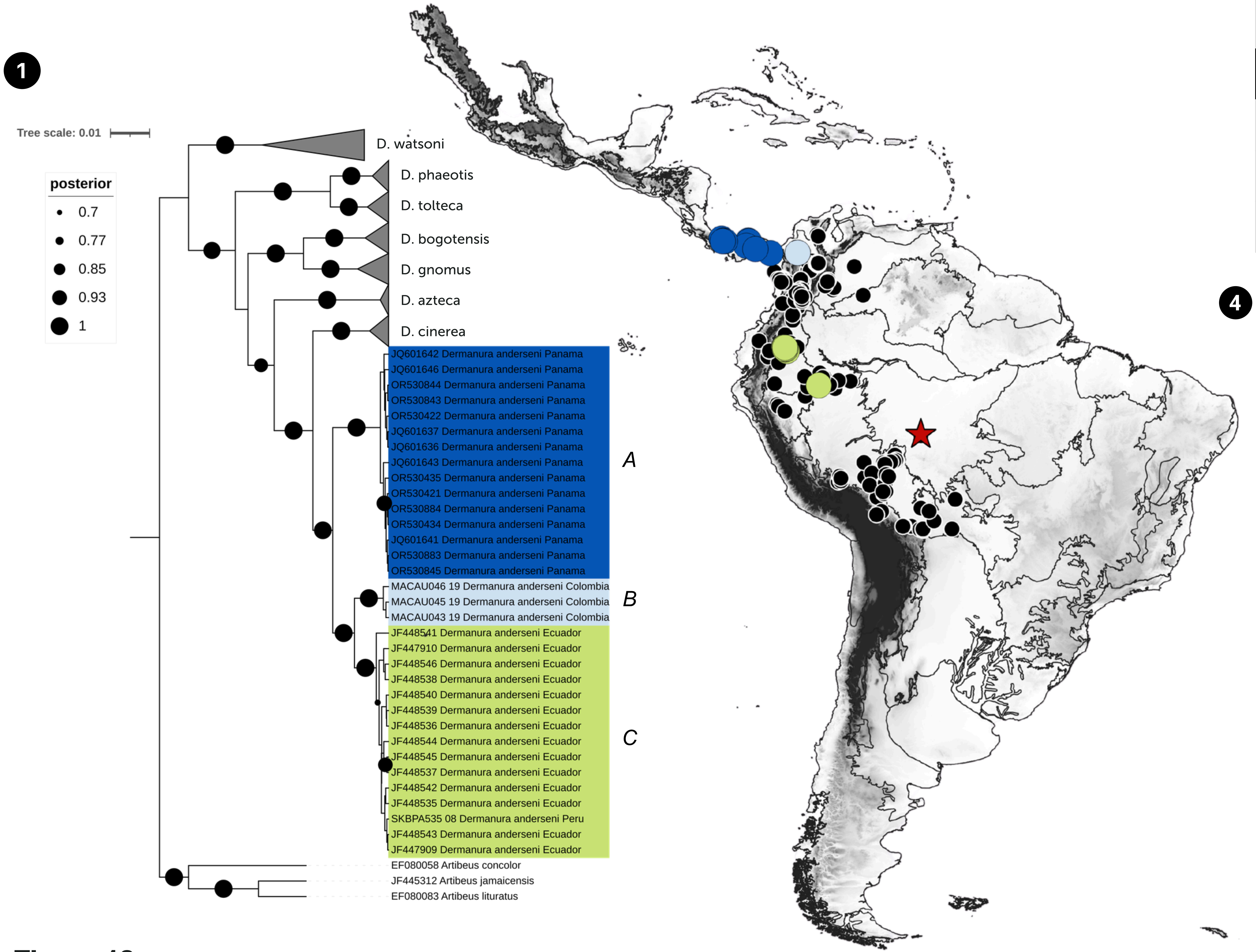

3

Mean genetic distances (Simple Distance)

Diagonal (red) = mean intraspecific distance | Off-diagonal (black) = mean interspecific distance | n = number of unique pairs

Especie	*D. anderseni* A	*D. anderseni* B	*D. anderseni* C
D. anderseni A	0.0013 (n=105)	0.0449 (n=45)	0.0368 (n=225)
D. anderseni B	0.0449 (n=45)	0.0031 (n=3)	0.0249 (n=45)
D. anderseni C	0.0368 (n=225)	0.0249 (n=45)	0.0029 (n=105)

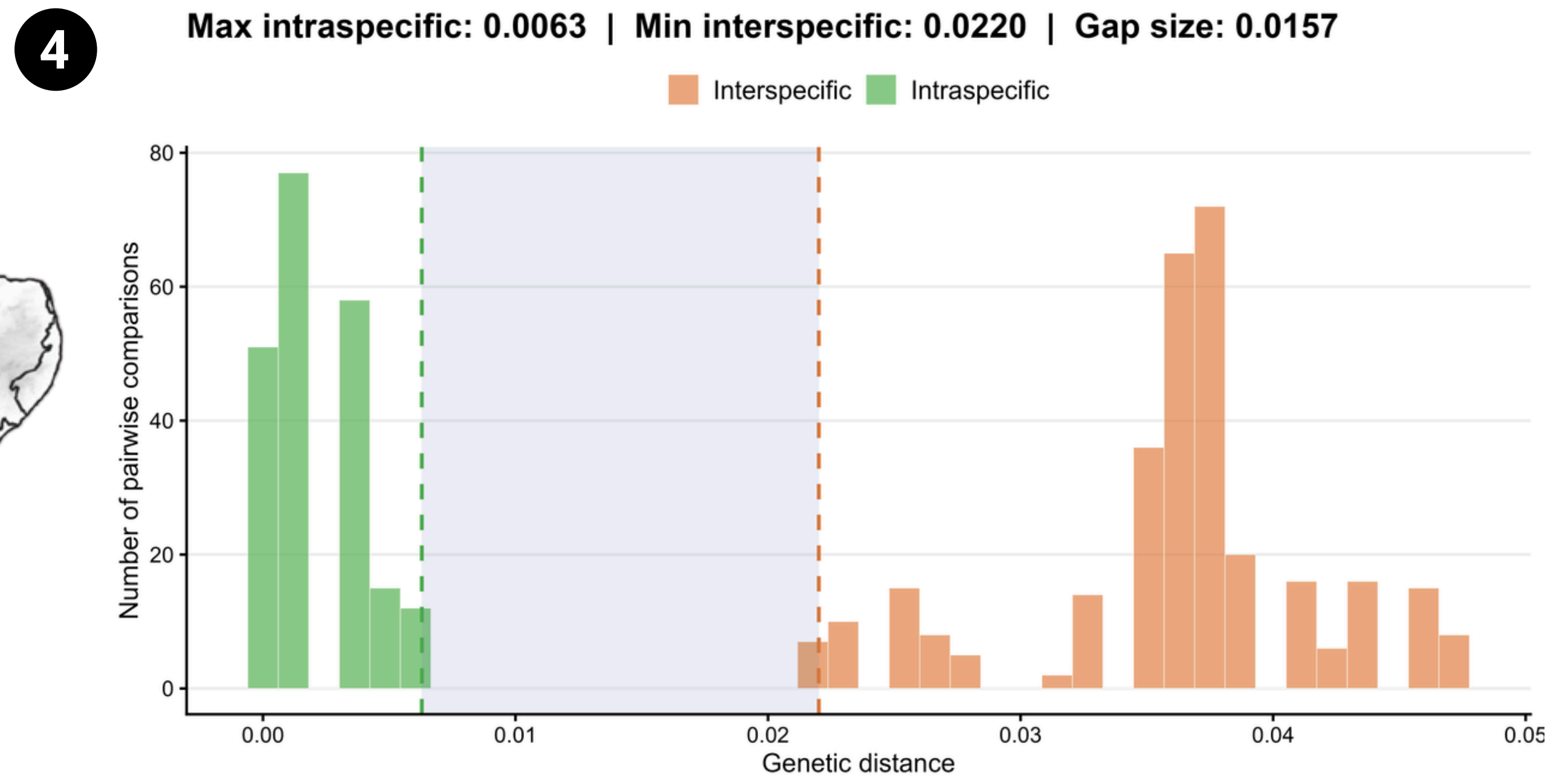

Figure 18

Fig. 18. Phylogenetic, geographic, and supporting evidence for candidate lineages in *Dermanura anderseni*. (1) Phylogenetic hypothesis based on COI sequences, with colored clades representing candidate lineages identified through molecular species delimitation. (2) Geographic distribution map, where colored points correspond to the lineages recovered in the phylogeny and black points represent the overall known distribution of the species complex. (3) Table of genetic distances (when sufficient data are available), reporting intra- and inter-lineage divergence among delimited groups. (4) Histogram of genetic distances illustrating the barcode gap. (5) Phylogenetic hypotheses from previous studies (when available), provided for comparison with our proposed delimitations. (6) Additional lines of evidence (occasionally available), such as geometric morphometrics or other independent datasets supporting lineage differentiation. Red star indicates holotype.

Note: *When this symbol (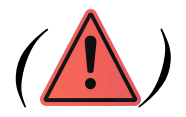) appears next to the phylogeny, it indicates that the delimitation should be interpreted with caution, as additional sampling is needed. When it appears next to the table, it means that genetic distances are low; therefore, we recommend that these results also be considered with caution. In both cases, we have retained the delimitations as inferred by our methods.*

Dermanura watsoni

1

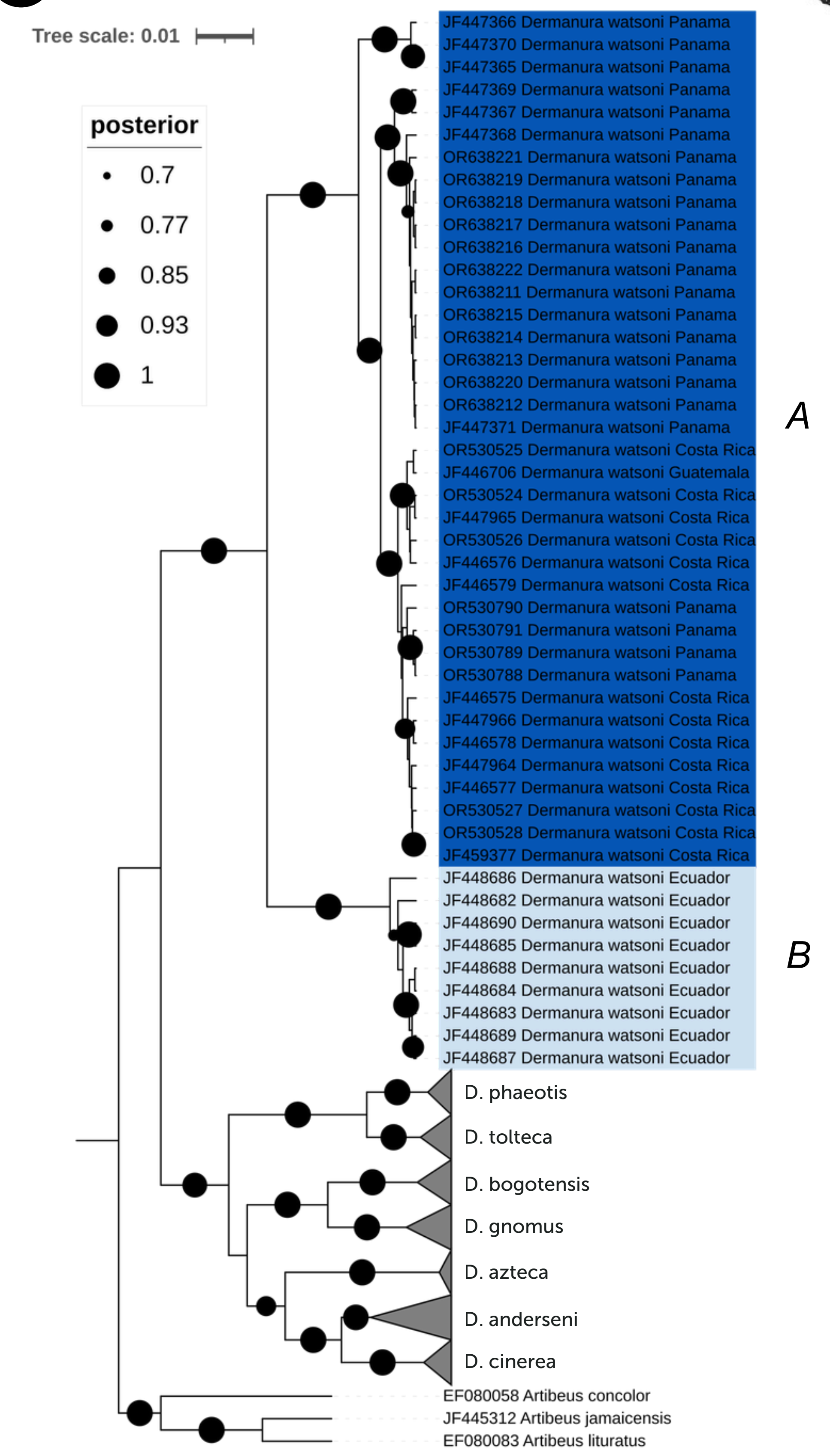

2

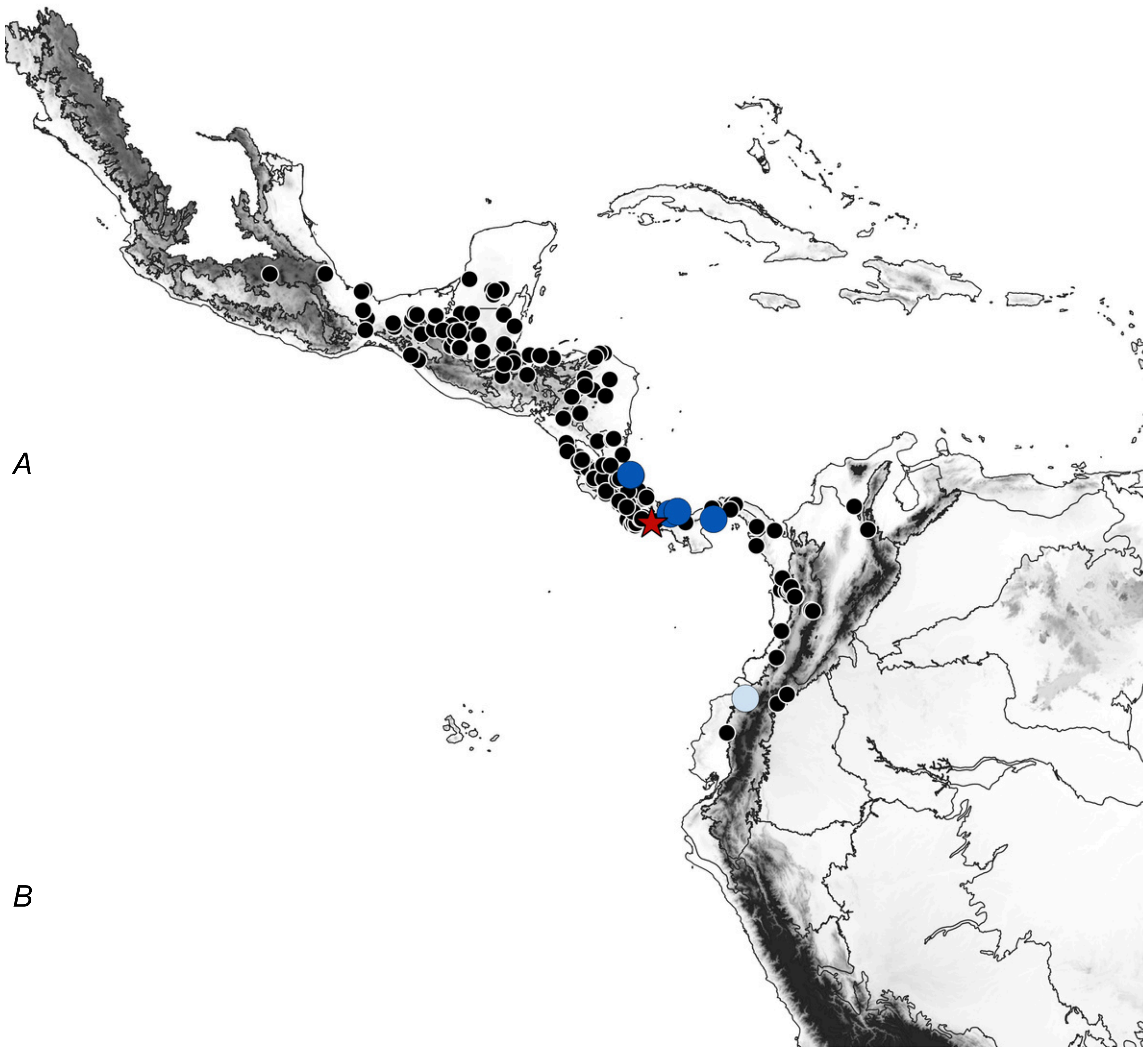

3

Mean genetic distances (Simple Distance)

Diagonal (red) = mean intraspecific distance | Off-diagonal (black) = mean interspecific distance | n = number of unique pairs

Especie	D. *watsoni* A	D. *watsoni* B
D. *watsoni* A	0.0139 (n=703)	0.0847 (n=342)
D. *watsoni* B	0.0847 (n=342)	0.0066 (n=36)

4

Max intraspecific: 0.0330 | Min interspecific: 0.0750 | Gap size: 0.0419

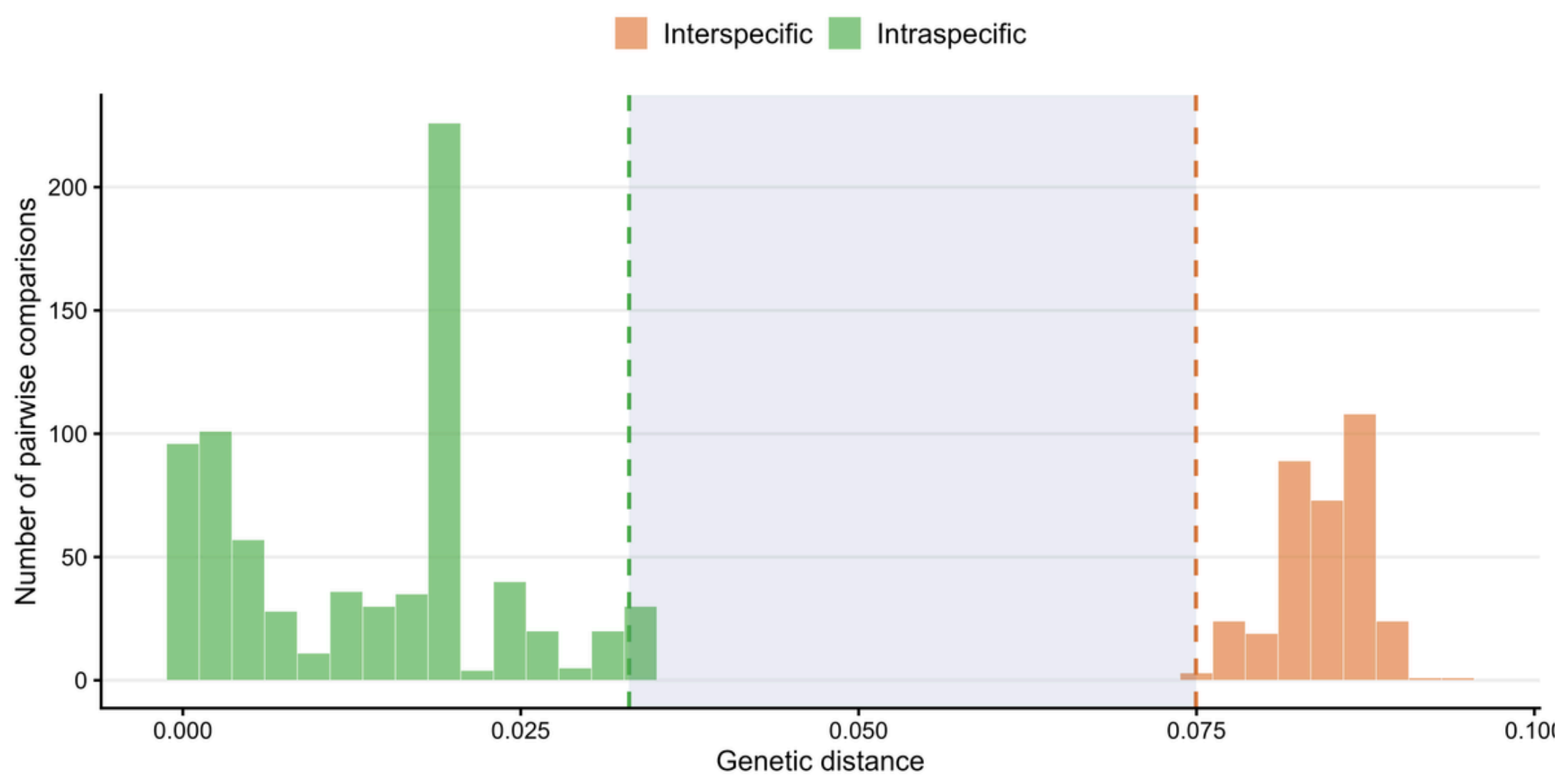

5 COI

Clare et al., (2011) PloS One

Figure 19

Fig. 19. Phylogenetic, geographic, and supporting evidence for candidate lineages in *Dermanura watsoni*. (1) Phylogenetic hypothesis based on COI sequences, with colored clades representing candidate lineages identified through molecular species delimitation. (2) Geographic distribution map, where colored points correspond to the lineages recovered in the phylogeny and black points represent the overall known distribution of the species complex. (3) Table of genetic distances (when sufficient data are available), reporting intra- and inter-lineage divergence among delimited groups. (4) Histogram of genetic distances illustrating the barcode gap. (5) Phylogenetic hypotheses from previous studies (when available), provided for comparison with our proposed delimitations. (6) Additional lines of evidence (occasionally available), such as geometric morphometrics or other independent datasets supporting lineage differentiation. Red star indicates holotype.

Note: *When this symbol (⚠) appears next to the phylogeny, it indicates that the delimitation should be interpreted with caution, as additional sampling is needed. When it appears next to the table, it means that genetic distances are low; therefore, we recommend that these results also be considered with caution. In both cases, we have retained the delimitations as inferred by our methods.*

Desmodus rotundus

1

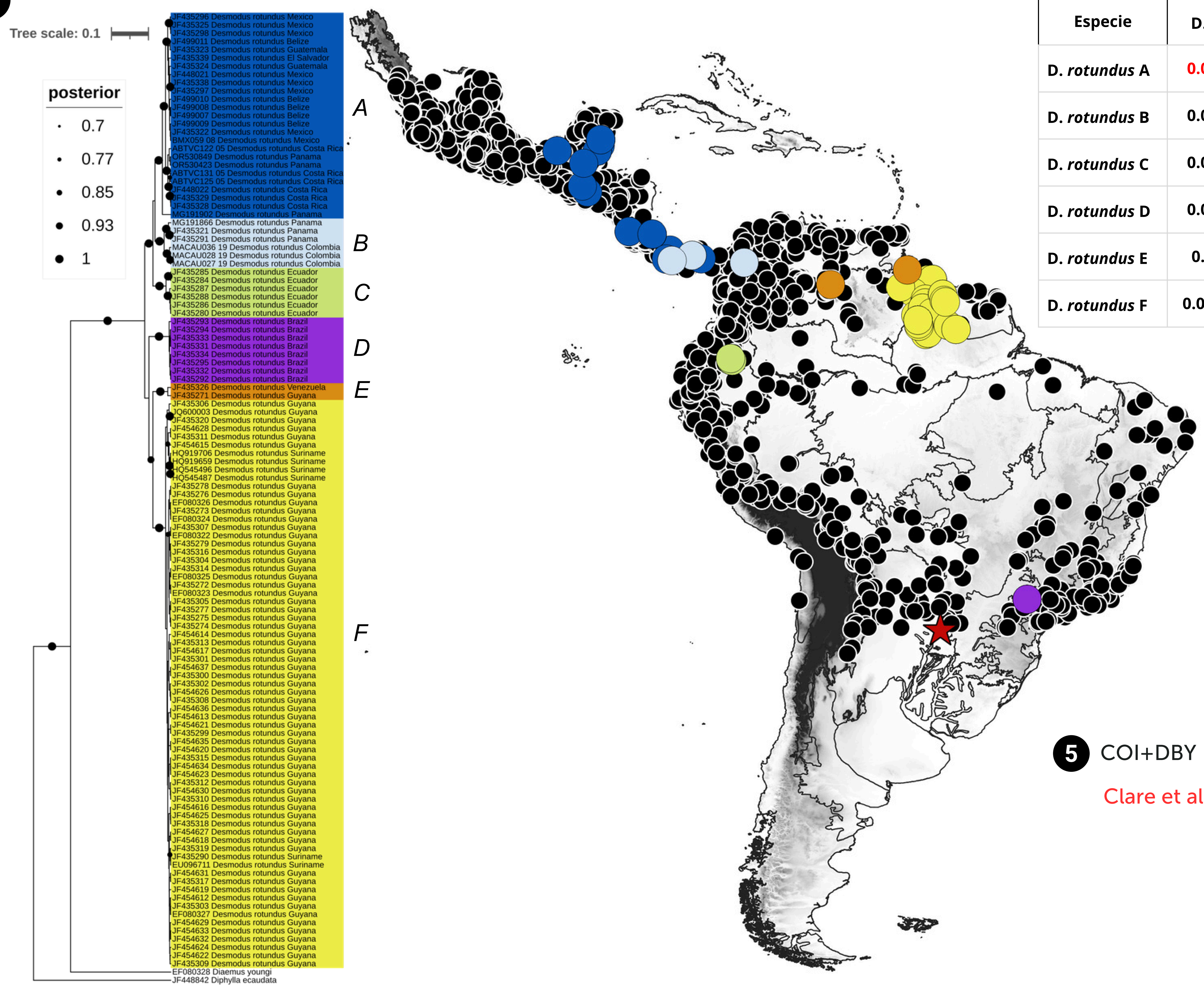

3

Mean genetic distances (Simple Distance)						
Diagonal (red) = mean intraspecific distance | Off-diagonal (black) = mean interspecific distance | n = number of unique pairs						
**Especie**	**D. *rotundus* A**	**D. *rotundus* B**	**D. *rotundus* C**	**D. *rotundus* D**	**D. *rotundus* E**	**D. *rotundus* F**
**D. *rotundus* A**	0.0064 (n=300)	0.0398 (n=150)	0.0391 (n=150)	0.0512 (n=200)	0.0495 (n=50)	0.0476 (n=1725)
**D. *rotundus* B**	0.0398 (n=150)	0.0103 (n=15)	0.0401 (n=36)	0.0543 (n=48)	0.0517 (n=12)	0.0498 (n=414)
**D. *rotundus* C**	0.0391 (n=150)	0.0401 (n=36)	0.0057 (n=15)	0.0541 (n=48)	0.0567 (n=12)	0.0466 (n=414)
**D. *rotundus* D**	0.0512 (n=200)	0.0543 (n=48)	0.0541 (n=48)	0.0000 (n=28)	0.0522 (n=16)	0.0476 (n=552)
**D. *rotundus* E**	0.0495 (n=50)	0.0517 (n=12)	0.0567 (n=12)	0.0522 (n=16)	0.0077 (n=1)	0.0398 (n=138)
**D. *rotundus* F**	0.0476 (n=1725)	0.0498 (n=414)	0.0466 (n=414)	0.0476 (n=552)	0.0398 (n=138)	0.0020 (n=2346)

4

Max intraspecific: 0.0181 | Min interspecific: 0.0300 | Gap size: 0.0119

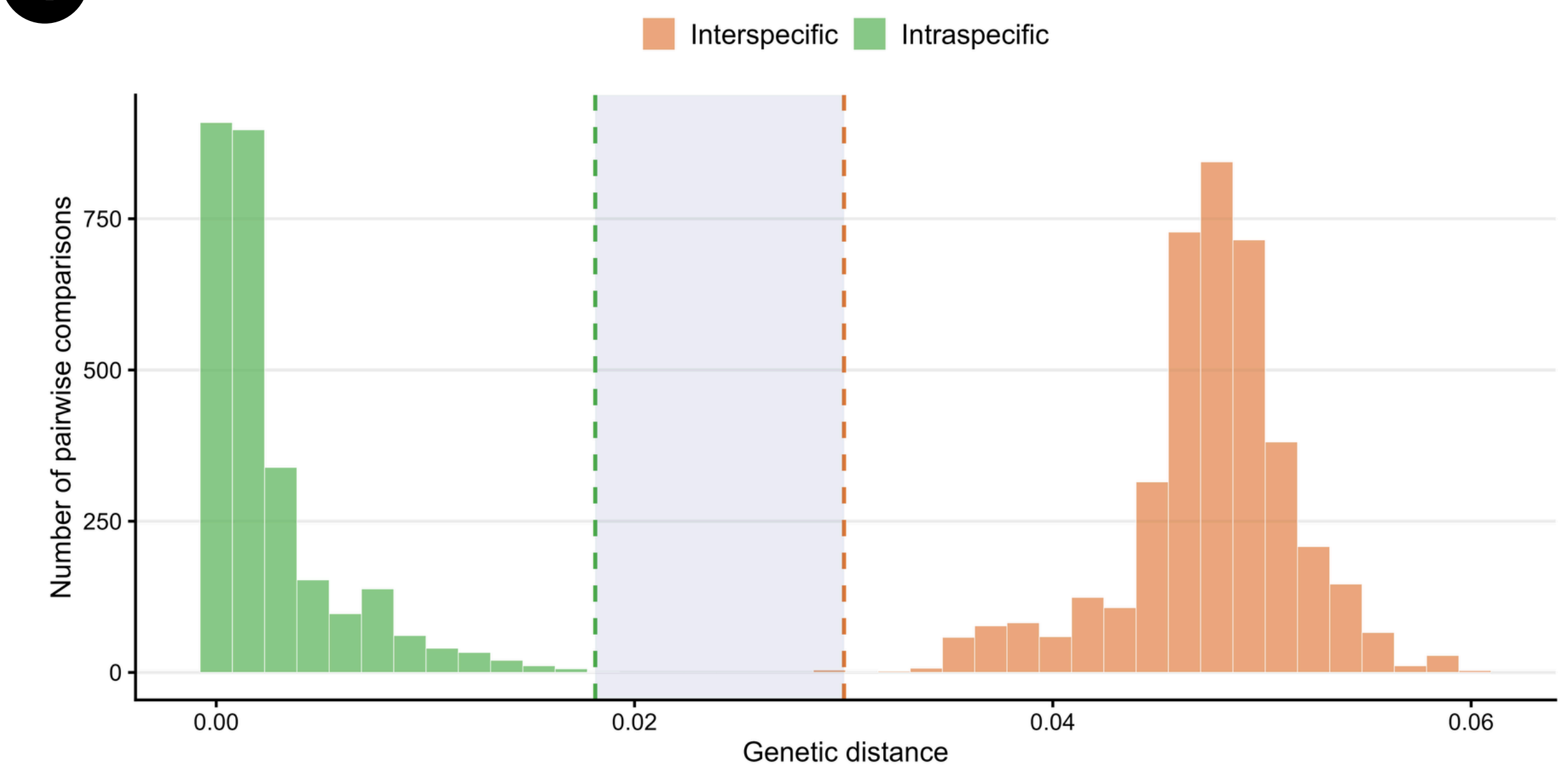

5 COI+DBY

Clare et al., (2011) PloS One

CYTB

Martins et al., (2009) BMC Evol Biol

Figure 20

Fig. 20. Phylogenetic, geographic, and supporting evidence for candidate lineages in *Desmodus rotundus*. (1) Phylogenetic hypothesis based on COI sequences, with colored clades representing candidate lineages identified through molecular species delimitation. (2) Geographic distribution map, where colored points correspond to the lineages recovered in the phylogeny and black points represent the overall known distribution of the species complex. (3) Table of genetic distances (when sufficient data are available), reporting intra- and inter-lineage divergence among delimited groups. (4) Histogram of genetic distances illustrating the barcode gap. (5) Phylogenetic hypotheses from previous studies (when available), provided for comparison with our proposed delimitations. (6) Additional lines of evidence (occasionally available), such as geometric morphometrics or other independent datasets supporting lineage differentiation. Red star indicates holotype.

Note: *When this symbol (⚠) appears next to the phylogeny, it indicates that the delimitation should be interpreted with caution, as additional sampling is needed. When it appears next to the table, it means that genetic distances are low; therefore, we recommend that these results also be considered with caution. In both cases, we have retained the delimitations as inferred by our methods.*

Glyphonycteris sylvestris

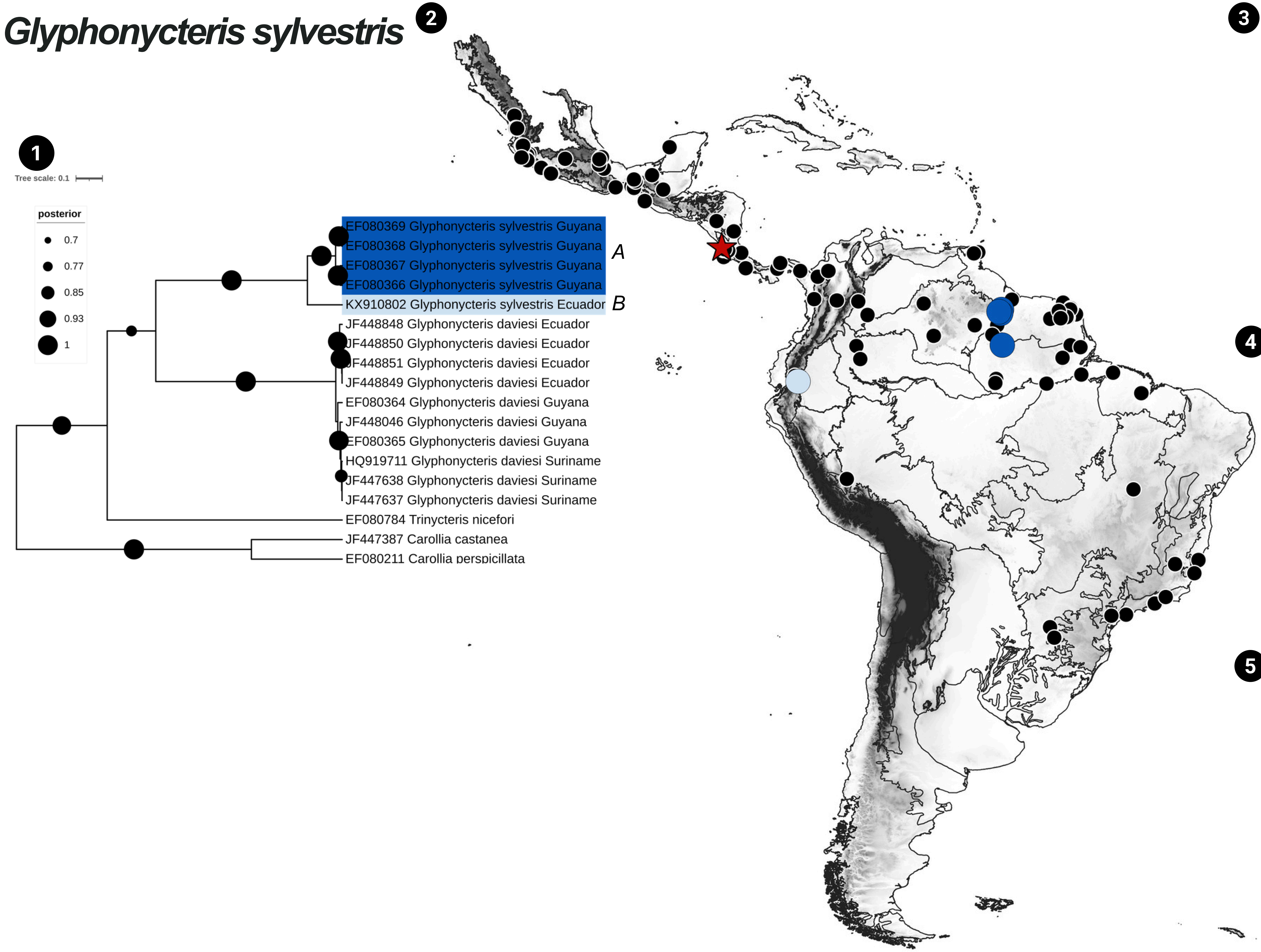

3

Mean genetic distances (Simple Distance)

Diagonal (red) = mean intraspecific distance | Off-diagonal (black) = mean interspecific distance | n = number of unique pairs

Especie	G. *sylvestris* A	G. *sylvestris* B
G. *sylvestris* A	0.0138 (n=6)	0.0742 (n=4)
G. *sylvestris* B	0.0742 (n=4)	--

4

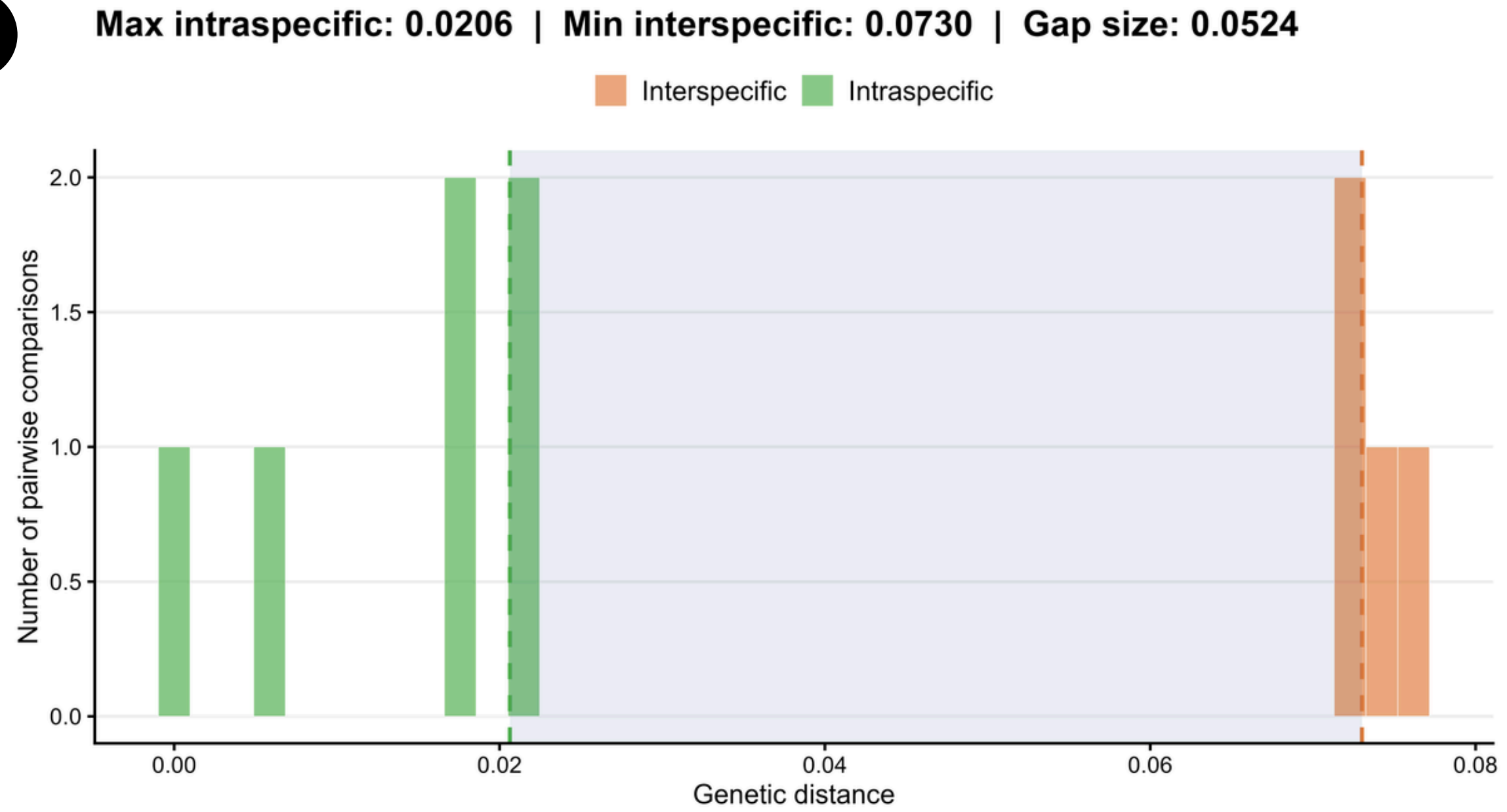

5 CYTB

Tiriria et al., (2016) Check List

Figure 21

Fig. 21. Phylogenetic, geographic, and supporting evidence for candidate lineages in *Glyphonycteris sylvestris.* (1) Phylogenetic hypothesis based on COI sequences, with colored clades representing candidate lineages identified through molecular species delimitation. (2) Geographic distribution map, where colored points correspond to the lineages recovered in the phylogeny and black points represent the overall known distribution of the species complex. (3) Table of genetic distances (when sufficient data are available), reporting intra- and inter-lineage divergence among delimited groups. (4) Histogram of genetic distances illustrating the barcode gap. (5) Phylogenetic hypotheses from previous studies (when available), provided for comparison with our proposed delimitations. (6) Additional lines of evidence (occasionally available), such as geometric morphometrics or other independent datasets supporting lineage differentiation. Red star indicates holotype.

Note: *When this symbol (⚠) appears next to the phylogeny, it indicates that the delimitation should be interpreted with caution, as additional sampling is needed. When it appears next to the table, it means that genetic distances are low; therefore, we recommend that these results also be considered with caution. In both cases, we have retained the delimitations as inferred by our methods.*

Hsunycteris thomasi

1

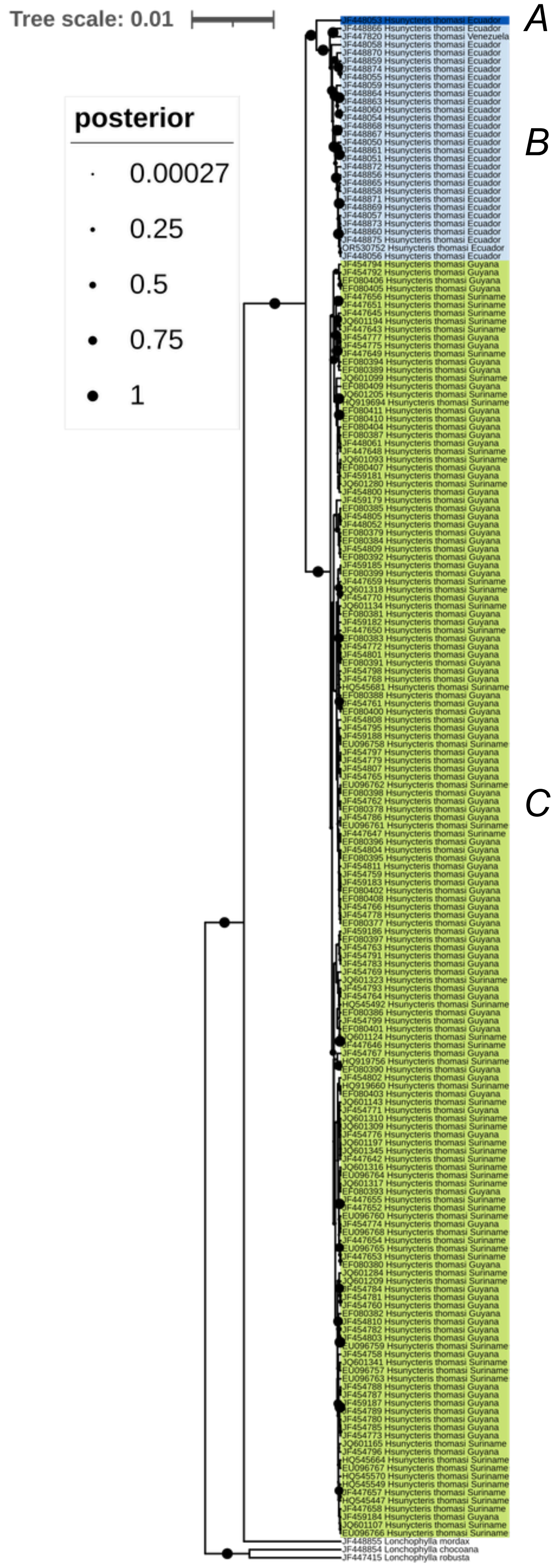

2

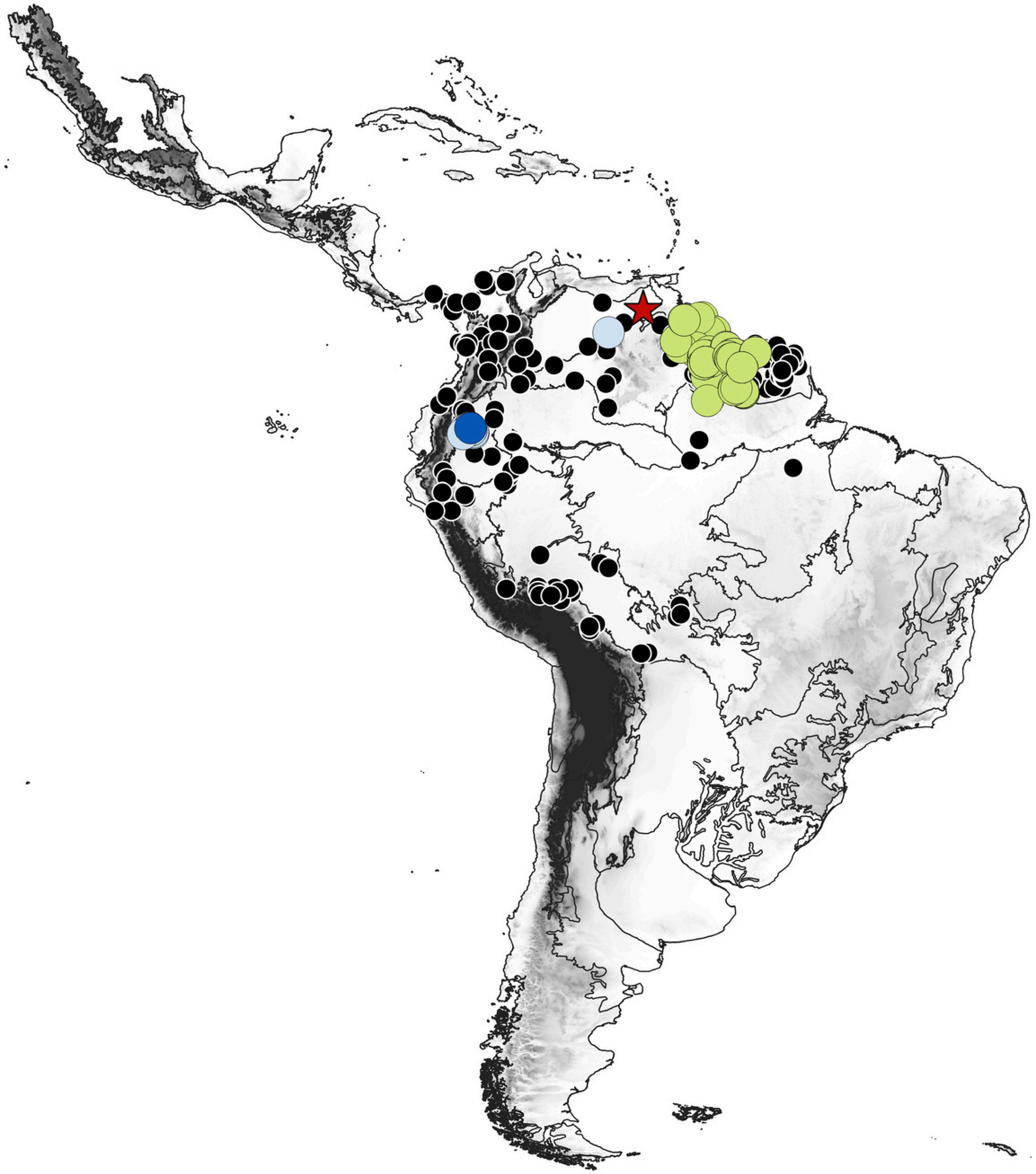

3

Mean genetic distances (Simple Distance)

Diagonal (red) = mean intraspecific distance | Off-diagonal (black) = mean interspecific distance | n = number of unique pairs

Especie	H. *thomasi* A	H. *thomasi* B	H. *thomasi* C
H. *thomasi* A	--	0.0503 (n=30)	0.0730 (n=157)
H. *thomasi* B	0.0503 (n=30)	0.0130 (n=435)	0.0641 (n=4710)
H. *thomasi* C	0.0730 (n=157)	0.0641 (n=4710)	0.0047 (n=12246)

4 No barcoding gap | Max intraspecific: 0.0614 | Min interspecific: 0.0000 | Overla

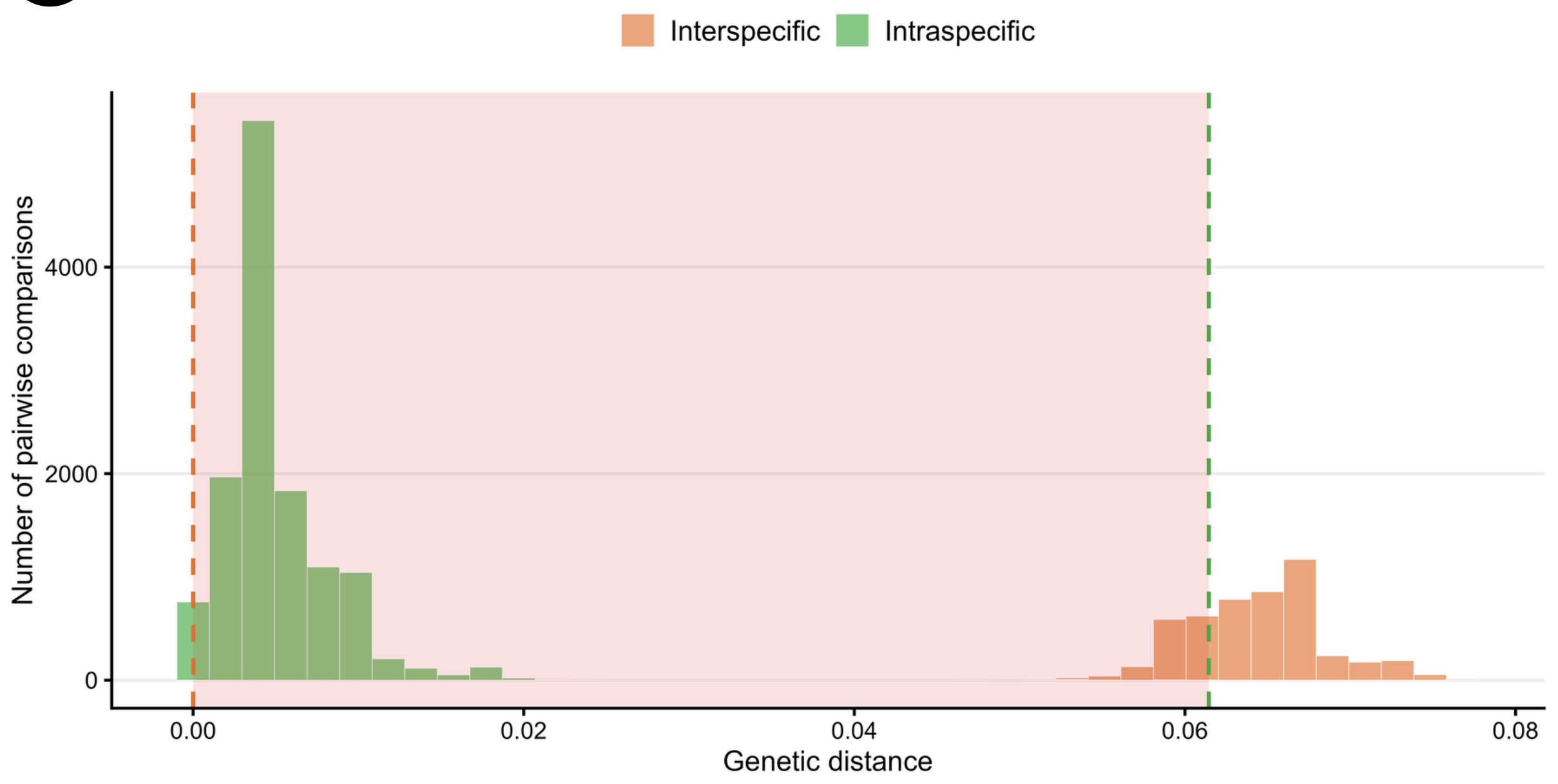

5 These studies support more than three lineages

Pilatti et al., (2025) Acta Chiropterol.

Benathar et al., (2024) Zool. Scr.

Figure 22

Fig. 22. Phylogenetic, geographic, and supporting evidence for candidate lineages in *Hsunycteris thomasi.* (1) Phylogenetic hypothesis based on COI sequences, with colored clades representing candidate lineages identified through molecular species delimitation. (2) Geographic distribution map, where colored points correspond to the lineages recovered in the phylogeny and black points represent the overall known distribution of the species complex. (3) Table of genetic distances (when sufficient data are available), reporting intra- and inter-lineage divergence among delimited groups. (4) Histogram of genetic distances illustrating the barcode gap. (5) Phylogenetic hypotheses from previous studies (when available), provided for comparison with our proposed delimitations. (6) Additional lines of evidence (occasionally available), such as geometric morphometrics or other independent datasets supporting lineage differentiation. Red star indicates holotype.

Note: *When this symbol (⚠) appears next to the phylogeny, it indicates that the delimitation should be interpreted with caution, as additional sampling is needed. When it appears next to the table, it means that genetic distances are low; therefore, we recommend that these results also be considered with caution. In both cases, we have retained the delimitations as inferred by our methods.*

Hylonycteris underwoodi

1

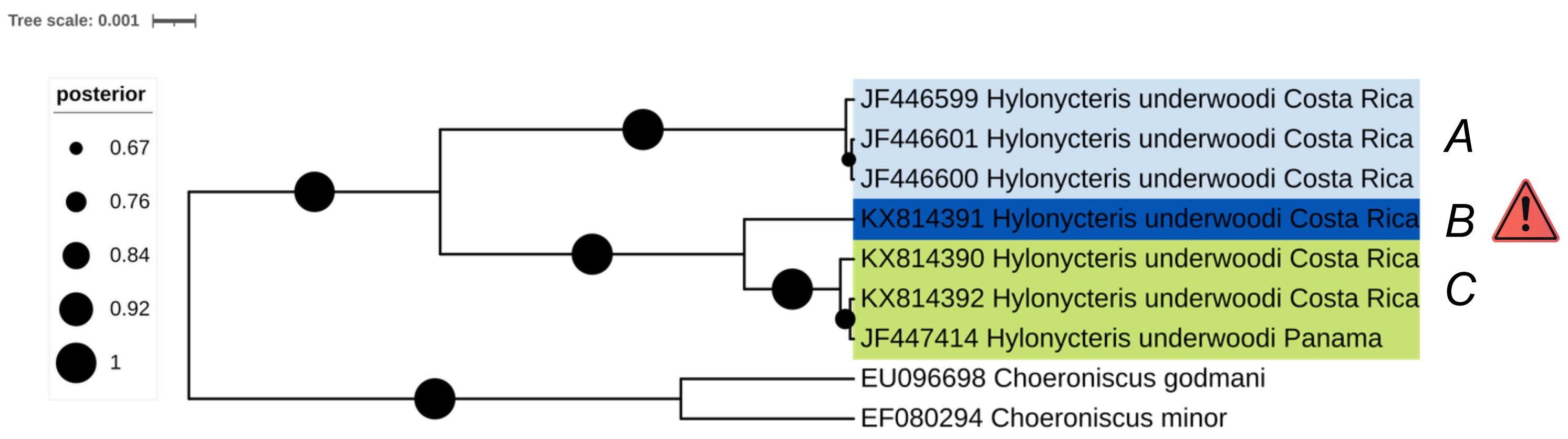

2

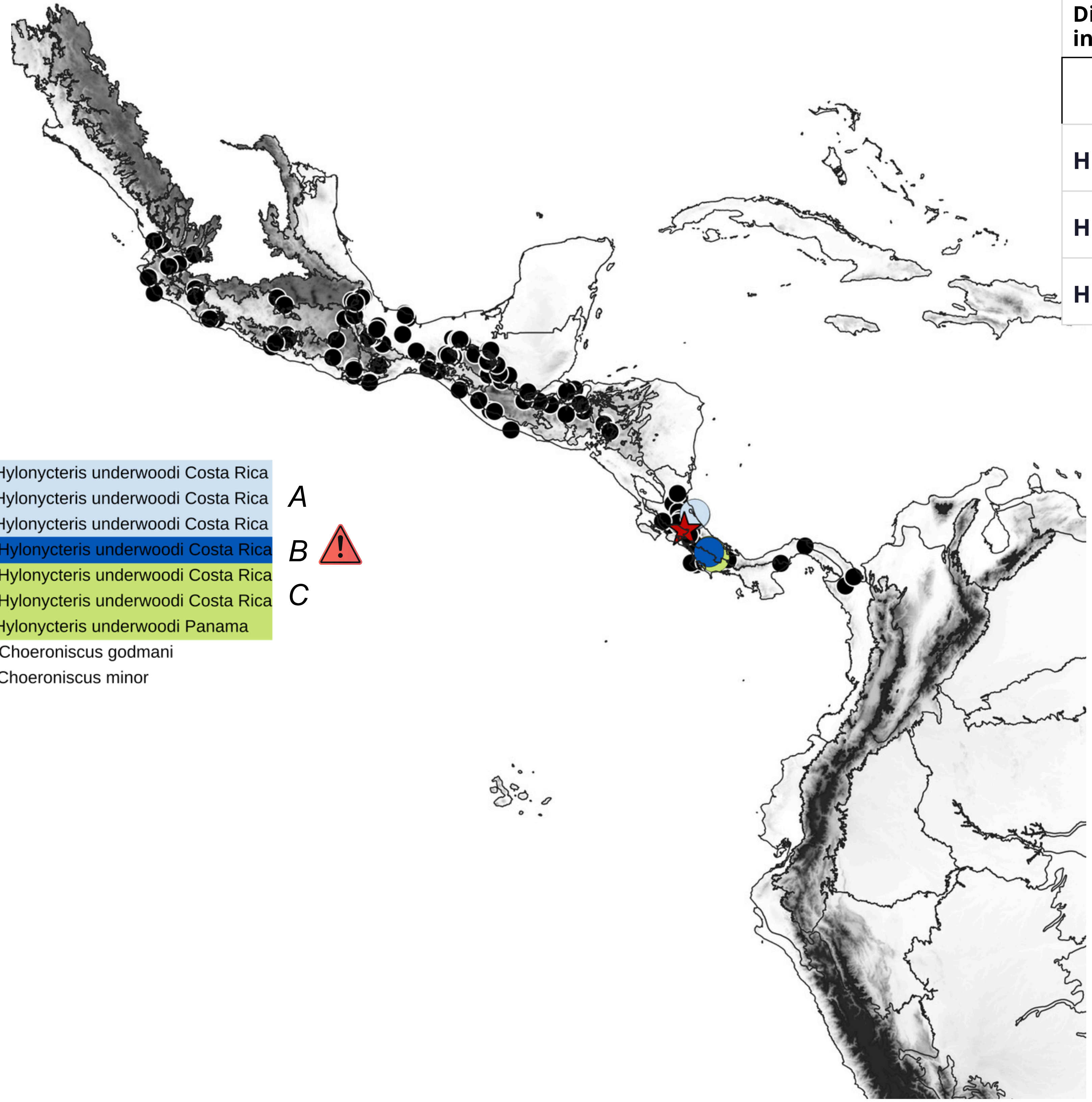

3

Mean genetic distances (Simple Distance)

Diagonal (red) = mean intraspecific distance | Off-diagonal (black) = mean interspecific distance | n = number of unique pairs

Especie	H. *underwoodi* A	H. *underwoodi* B	H. *underwoodi* C
H. *underwoodi* A	0.0011 (n=3)	0.0915 (n=3)	0.0889 (n=9)
H. *underwoodi* B	0.0915 (n=3)	--	0.0312 (n=3)
H. *underwoodi* C	0.0889 (n=9)	0.0312 (n=3)	0.0021 (n=3)

4

Max intraspecific: 0.0032 | Min interspecific: 0.0302 | Gap size: 0.0270

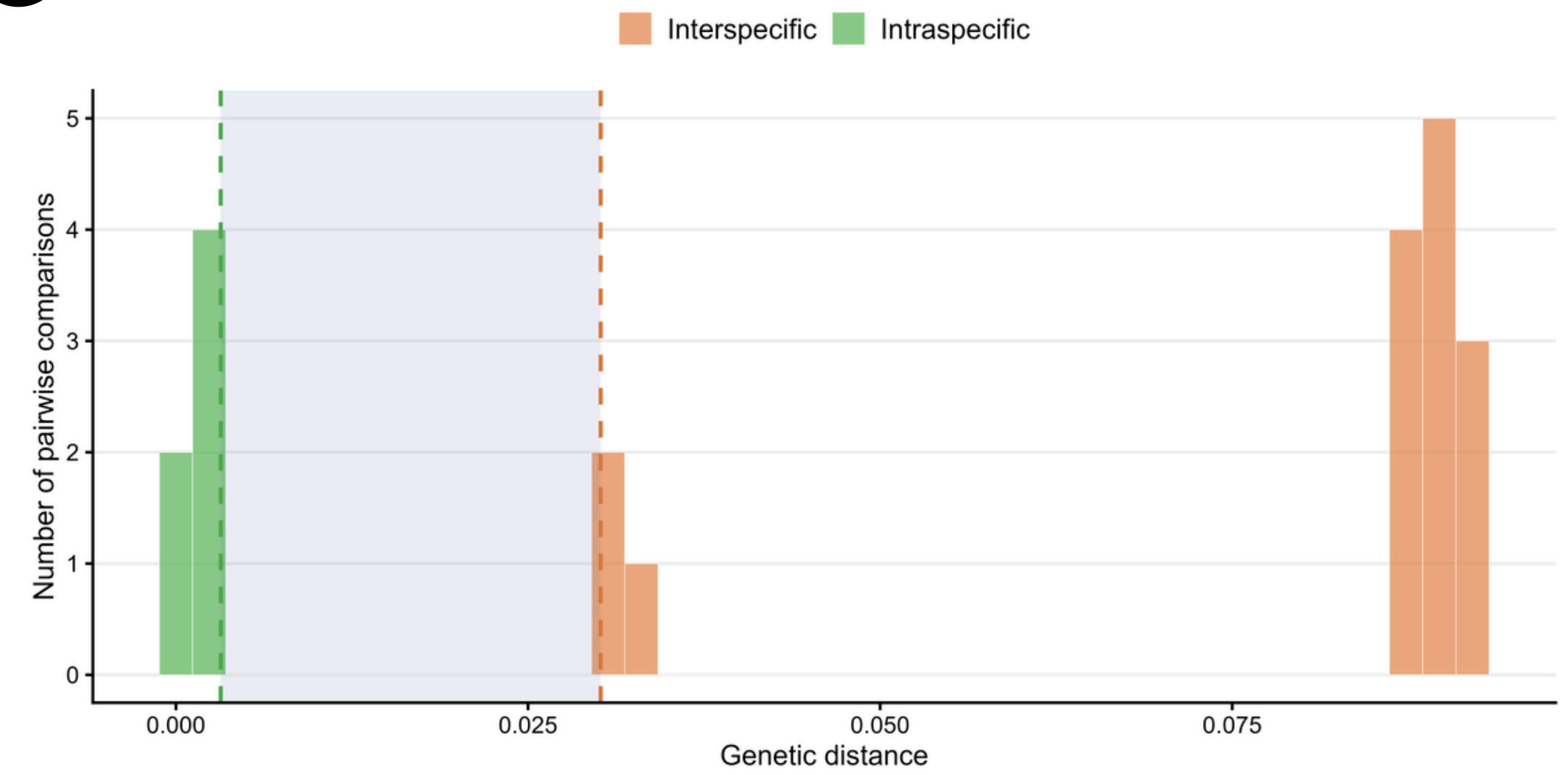

5

COI

Clare et al., (2011) PloS One

Figure 23

Fig. 23. Phylogenetic, geographic, and supporting evidence for candidate lineages in *Hylonycteris underwoodi*. (1) Phylogenetic hypothesis based on COI sequences, with colored clades representing candidate lineages identified through molecular species delimitation. (2) Geographic distribution map, where colored points correspond to the lineages recovered in the phylogeny and black points represent the overall known distribution of the species complex. (3) Table of genetic distances (when sufficient data are available), reporting intra- and inter-lineage divergence among delimited groups. (4) Histogram of genetic distances illustrating the barcode gap. (5) Phylogenetic hypotheses from previous studies (when available), provided for comparison with our proposed delimitations. (6) Additional lines of evidence (occasionally available), such as geometric morphometrics or other independent datasets supporting lineage differentiation. Red star indicates holotype.

Note: *When this symbol (⚠) appears next to the phylogeny, it indicates that the delimitation should be interpreted with caution, as additional sampling is needed. When it appears next to the table, it means that genetic distances are low; therefore, we recommend that these results also be considered with caution. In both cases, we have retained the delimitations as inferred by our methods.*

Lophostoma silvicola

1

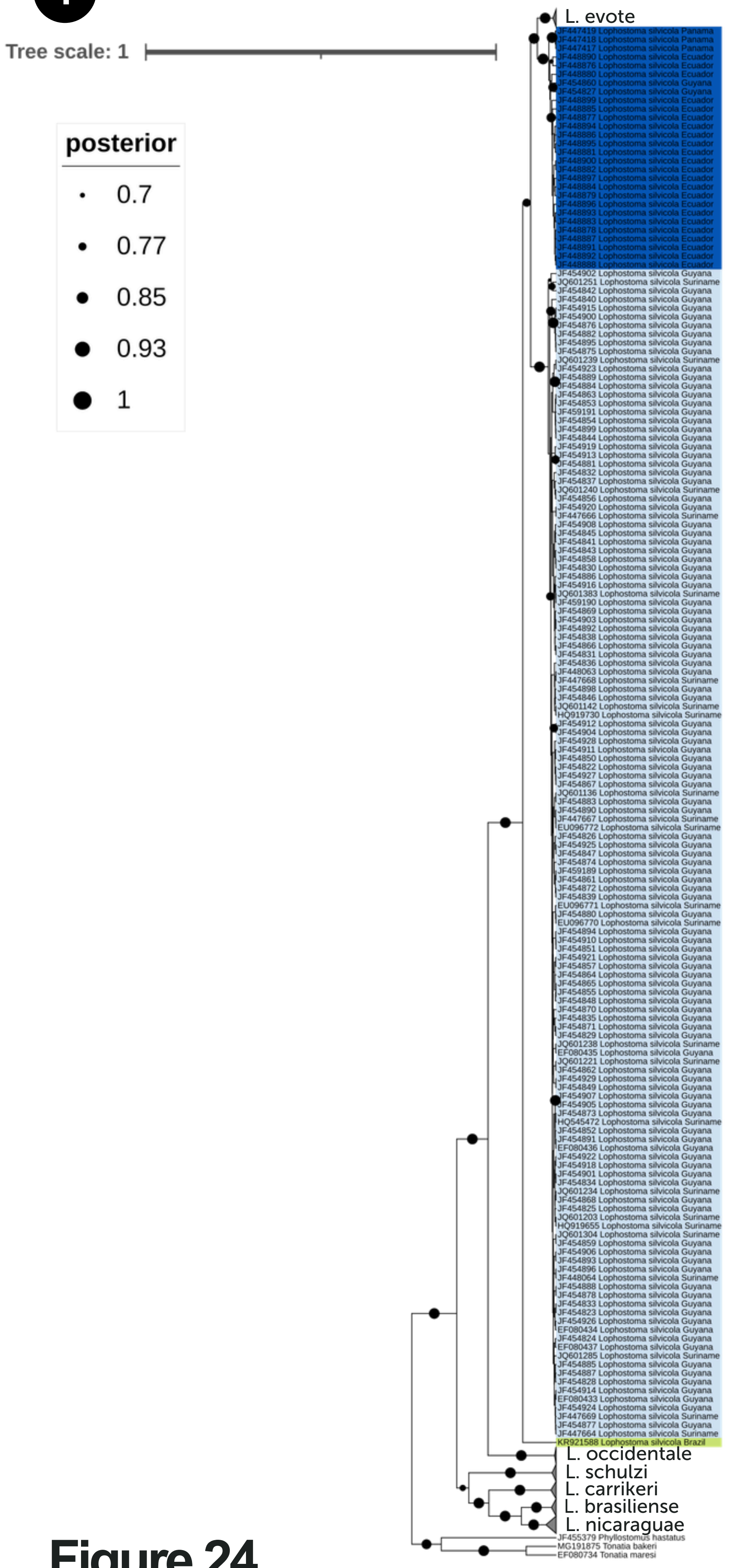

2

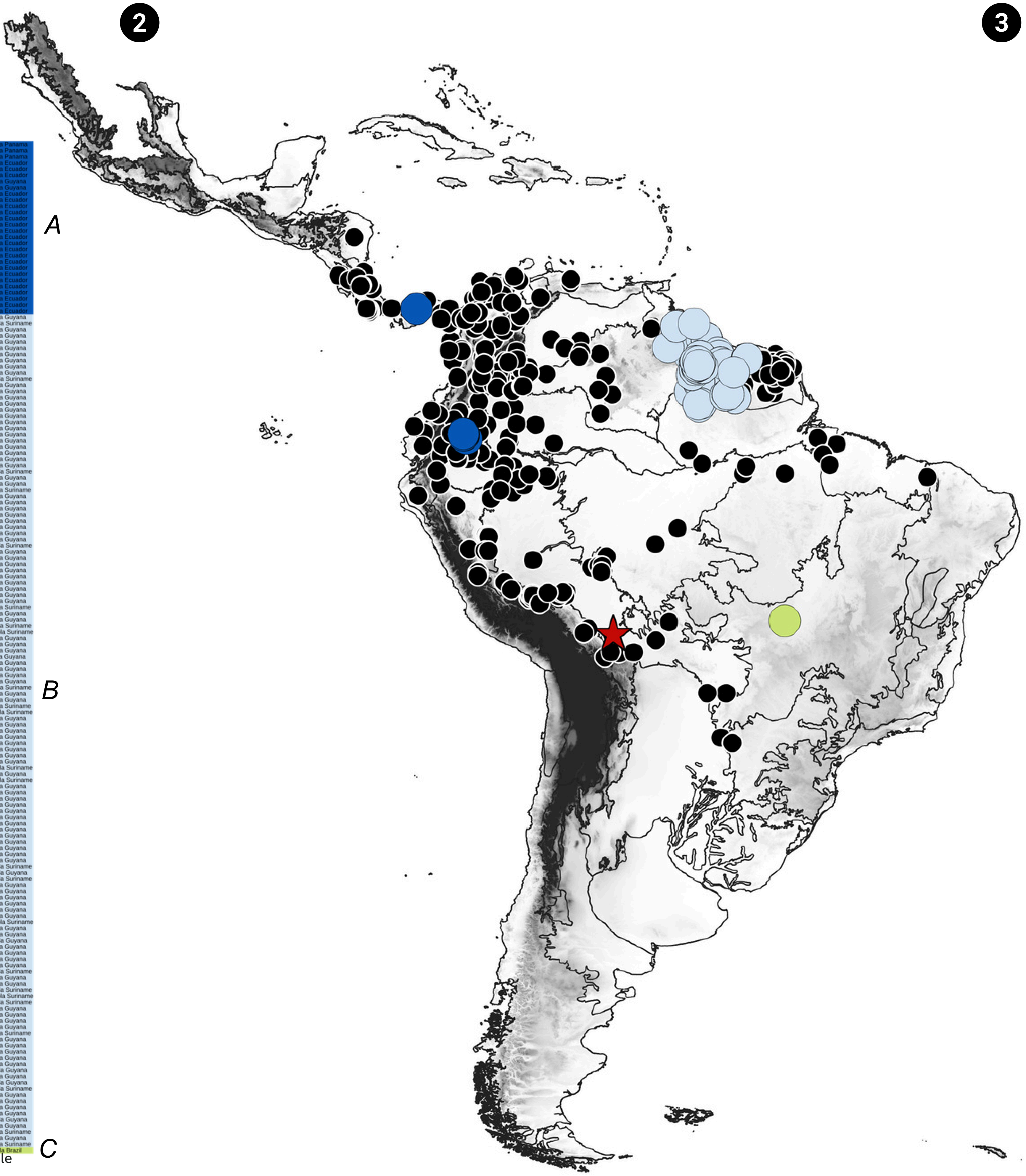

3

Mean genetic distances (Simple Distance)

Diagonal (red) = mean intraspecific distance | Off-diagonal (black) = mean interspecific distance | n = number of unique pairs

Especie	L. *silvicola* A	L. *silvicola* B	L. *silvicola* C
L. *silvicola* A	0.0058 (n=378)	0.0440 (n=3836)	0.0614 (n=28)
L. *silvicola* B	0.0440 (n=3836)	0.0034 (n=9316)	0.0705 (n=137)
L. *silvicola* C	0.0614 (n=28)	0.0705 (n=137)	--

4

No barcoding gap | Max intraspecific: 0.0474 | Min interspecific: 0.0000 | Overla

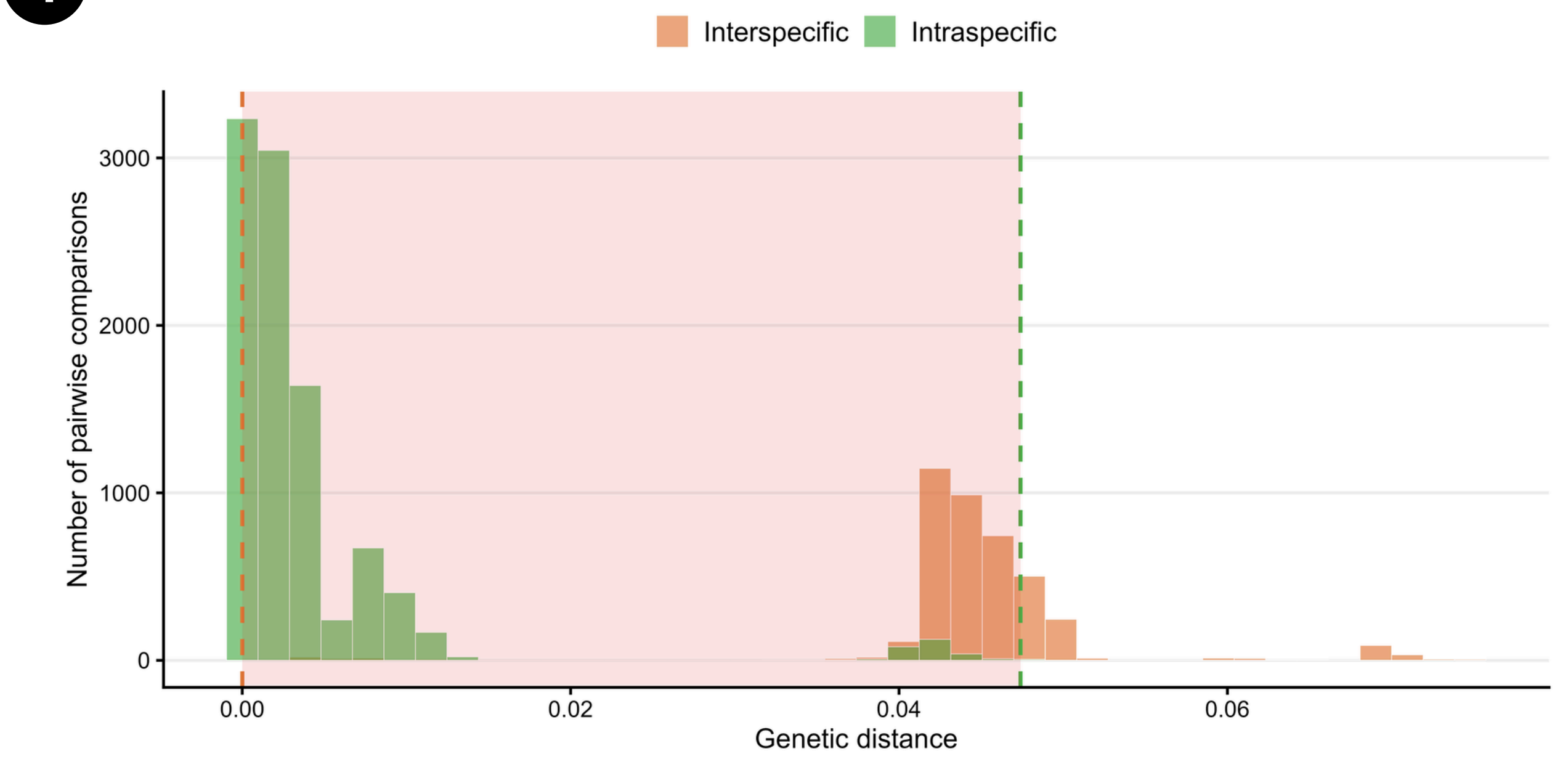

5 COI

Esquivel et al., (2022) Syst. Biodivers.

Figure 24

Fig. 24. Phylogenetic, geographic, and supporting evidence for candidate lineages in *Lophostoma silvicola.* (1) Phylogenetic hypothesis based on COI sequences, with colored clades representing candidate lineages identified through molecular species delimitation. (2) Geographic distribution map, where colored points correspond to the lineages recovered in the phylogeny and black points represent the overall known distribution of the species complex. (3) Table of genetic distances (when sufficient data are available), reporting intra- and inter-lineage divergence among delimited groups. (4) Histogram of genetic distances illustrating the barcode gap. (5) Phylogenetic hypotheses from previous studies (when available), provided for comparison with our proposed delimitations. (6) Additional lines of evidence (occasionally available), such as geometric morphometrics or other independent datasets supporting lineage differentiation. Red star indicates holotype.

Note: *When this symbol (⚠) appears next to the phylogeny, it indicates that the delimitation should be interpreted with caution, as additional sampling is needed. When it appears next to the table, it means that genetic distances are low; therefore, we recommend that these results also be considered with caution. In both cases, we have retained the delimitations as inferred by our methods.*

Macrophyllum macrophyllum

1

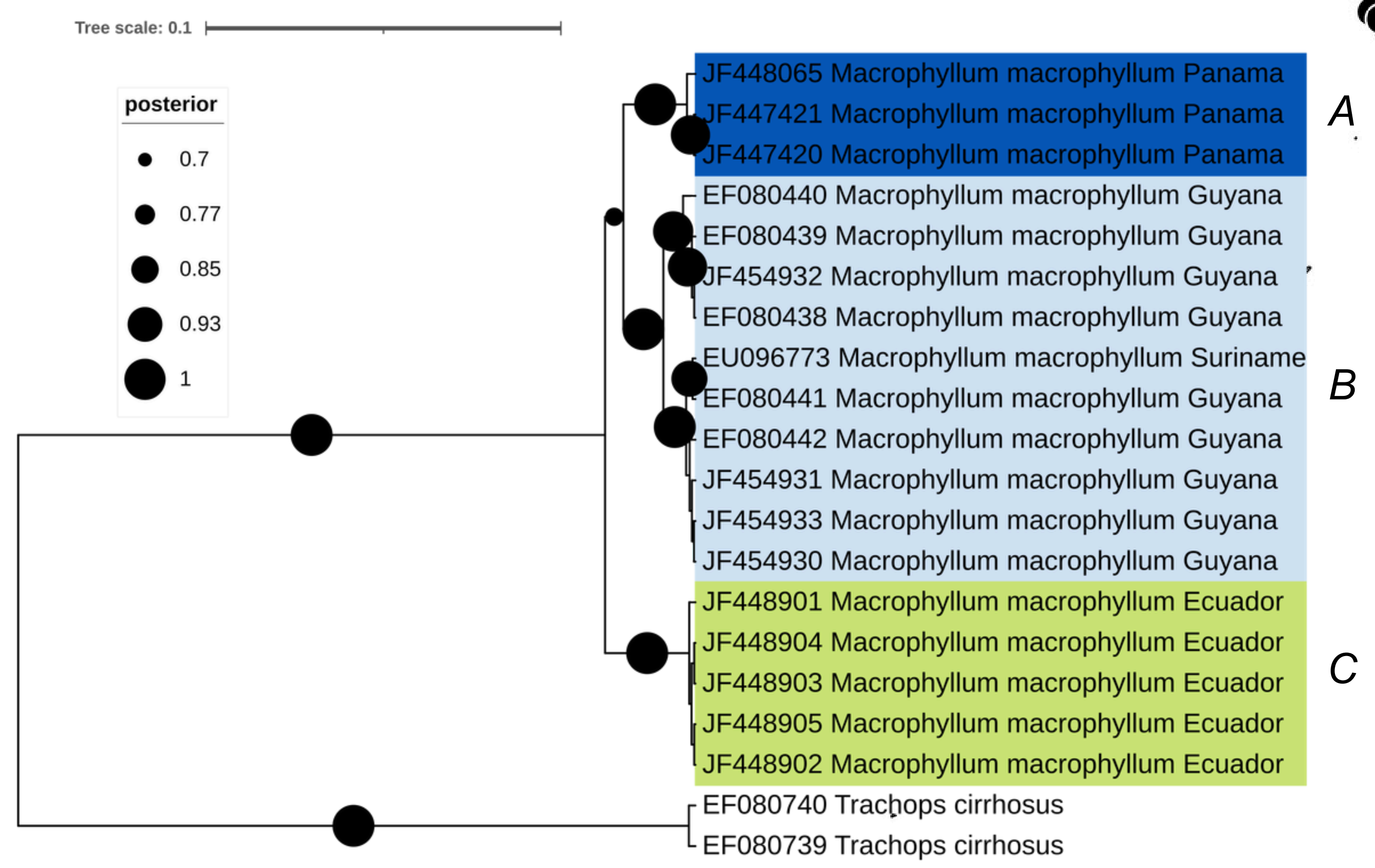

2

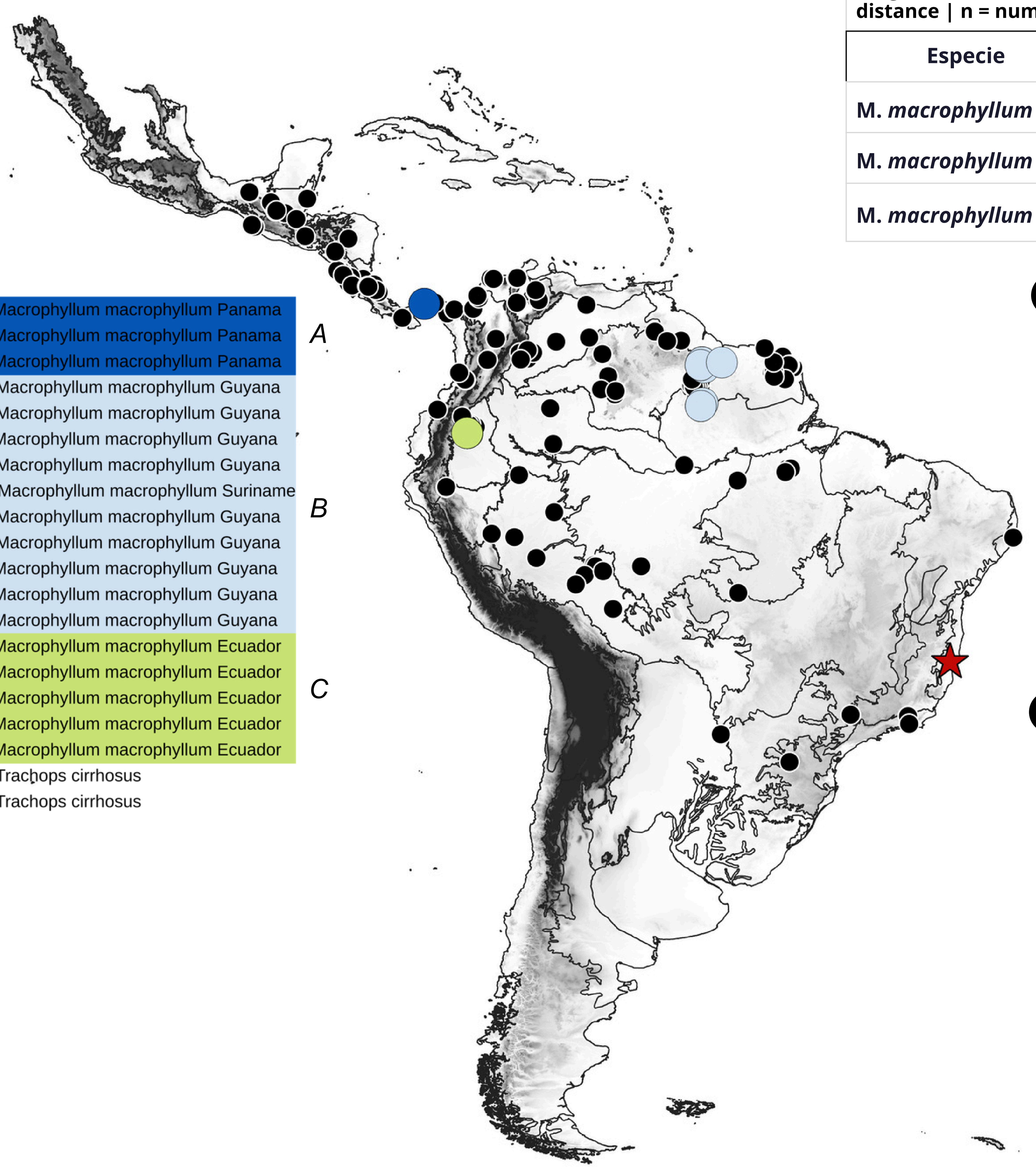

3

Mean genetic distances (Simple Distance)

Diagonal (red) = mean intraspecific distance | Off-diagonal (black) = mean interspecific distance | n = number of unique pairs

Especie	M. *macrophyllum* A	M. *macrophyllum* B	M. *macrophyllum* C
M. *macrophyllum* A	0.0021 (n=3)	0.0348 (n=30)	0.0406 (n=15)
M. *macrophyllum* B	0.0348 (n=30)	0.0079 (n=45)	0.0353 (n=50)
M. *macrophyllum* C	0.0406 (n=15)	0.0353 (n=50)	0.0000 (n=10)

4

Max intraspecific: 0.0154 | Min interspecific: 0.0307 | Gap size: 0.0154

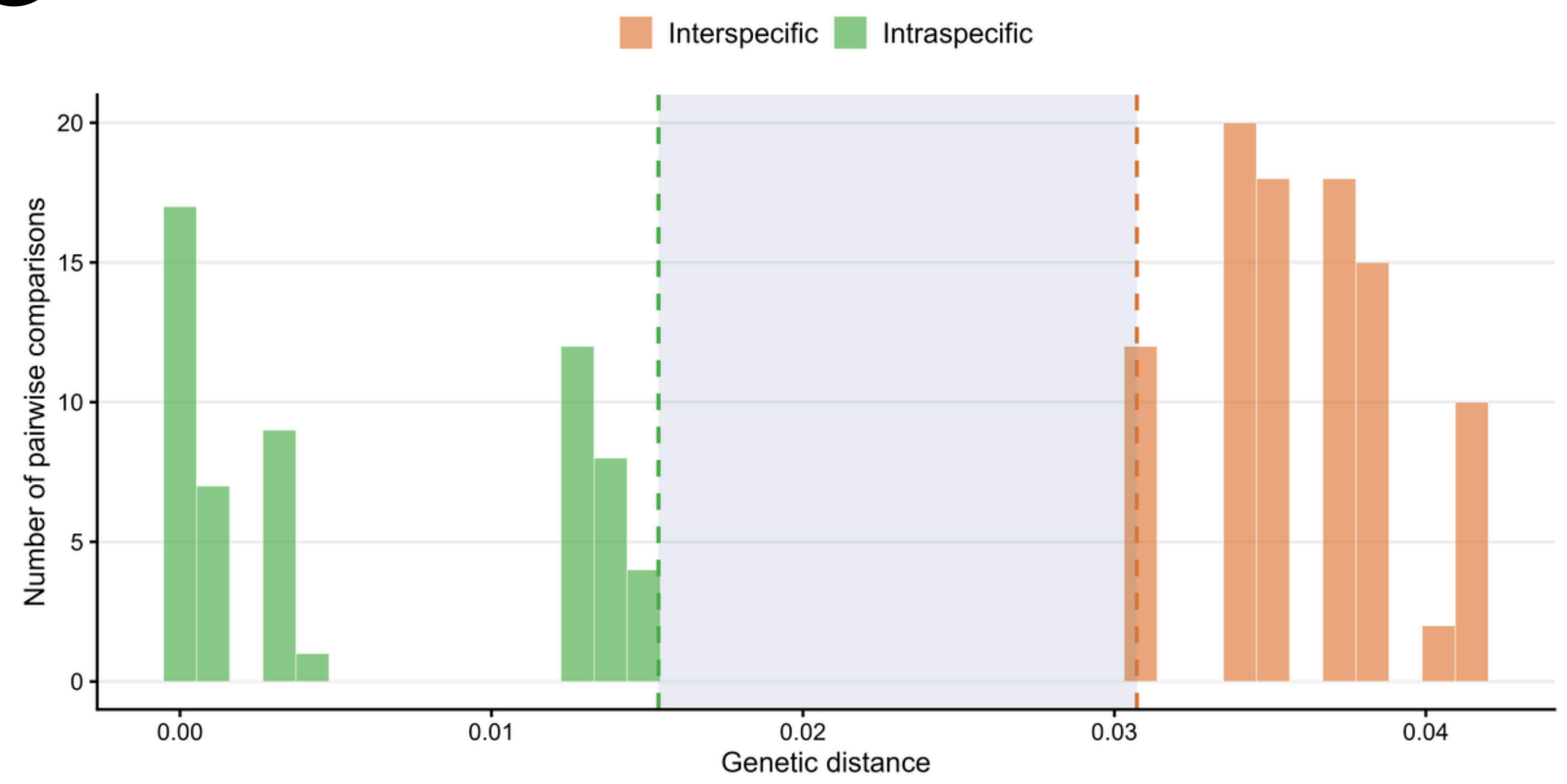

5

CYTB — Garbino et al., (2025) PeerJ

COI — Clare et al., (2011) PloS One

Figure 25

Fig. 25. Phylogenetic, geographic, and supporting evidence for candidate lineages in *Macrophyllum macrophyllum*. (1) Phylogenetic hypothesis based on COI sequences, with colored clades representing candidate lineages identified through molecular species delimitation. (2) Geographic distribution map, where colored points correspond to the lineages recovered in the phylogeny and black points represent the overall known distribution of the species complex. (3) Table of genetic distances (when sufficient data are available), reporting intra- and inter-lineage divergence among delimited groups. (4) Histogram of genetic distances illustrating the barcode gap. (5) Phylogenetic hypotheses from previous studies (when available), provided for comparison with our proposed delimitations. (6) Additional lines of evidence (occasionally available), such as geometric morphometrics or other independent datasets supporting lineage differentiation. Red star indicates holotype.

Note: *When this symbol (⚠) appears next to the phylogeny, it indicates that the delimitation should be interpreted with caution, as additional sampling is needed. When it appears next to the table, it means that genetic distances are low; therefore, we recommend that these results also be considered with caution. In both cases, we have retained the delimitations as inferred by our methods.*

Monophyllus redmani

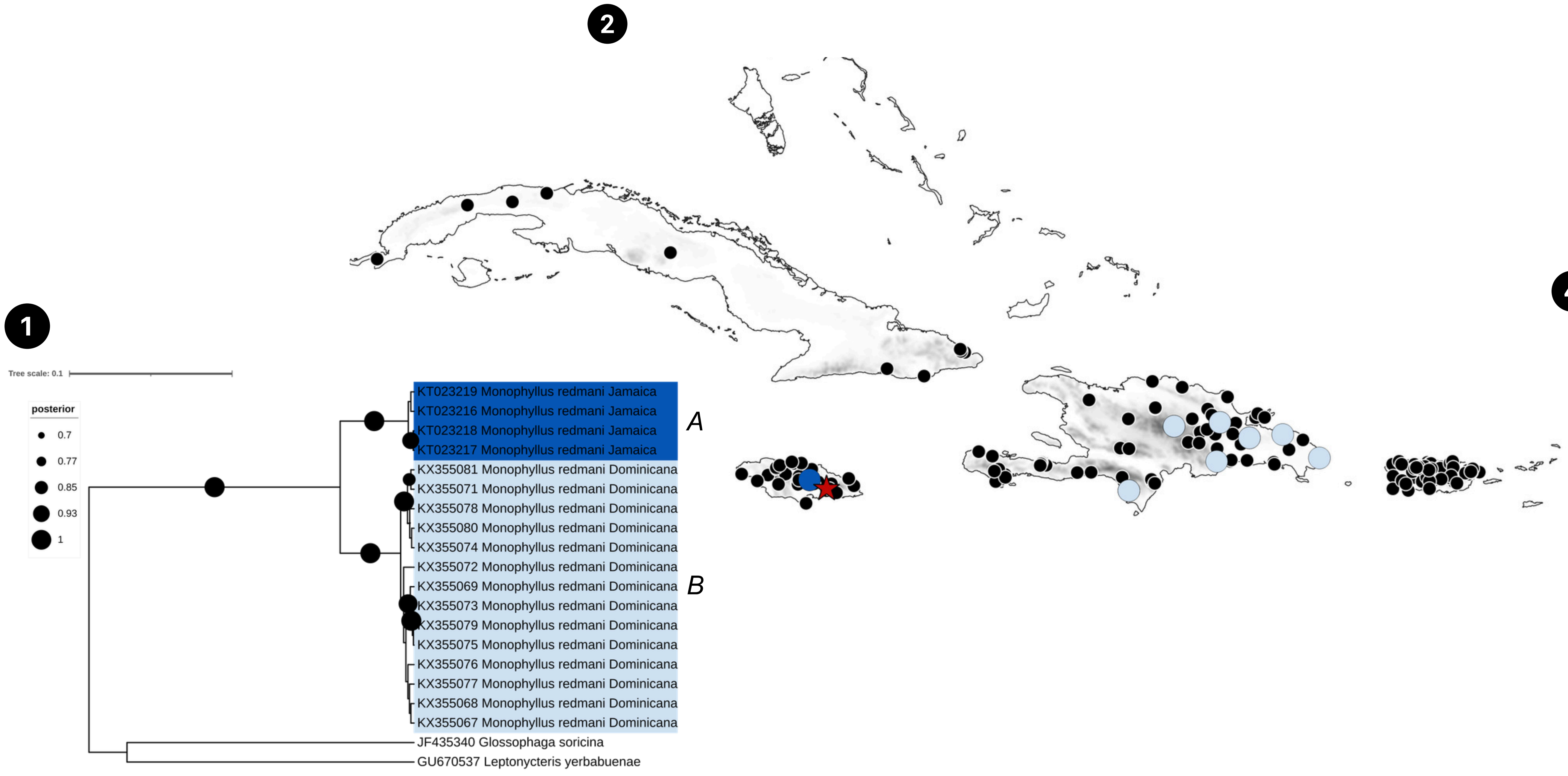

3

Mean genetic distances (Simple Distance)

Diagonal (red) = mean intraspecific distance | Off-diagonal (black) = mean interspecific distance | n = number of unique pairs

Especie	M. *redmani* A	M. *redmani* B
M. *redmani* A	0.0034 (n=6)	0.0756 (n=56)
M. *redmani* B	0.0756 (n=56)	0.0085 (n=91)

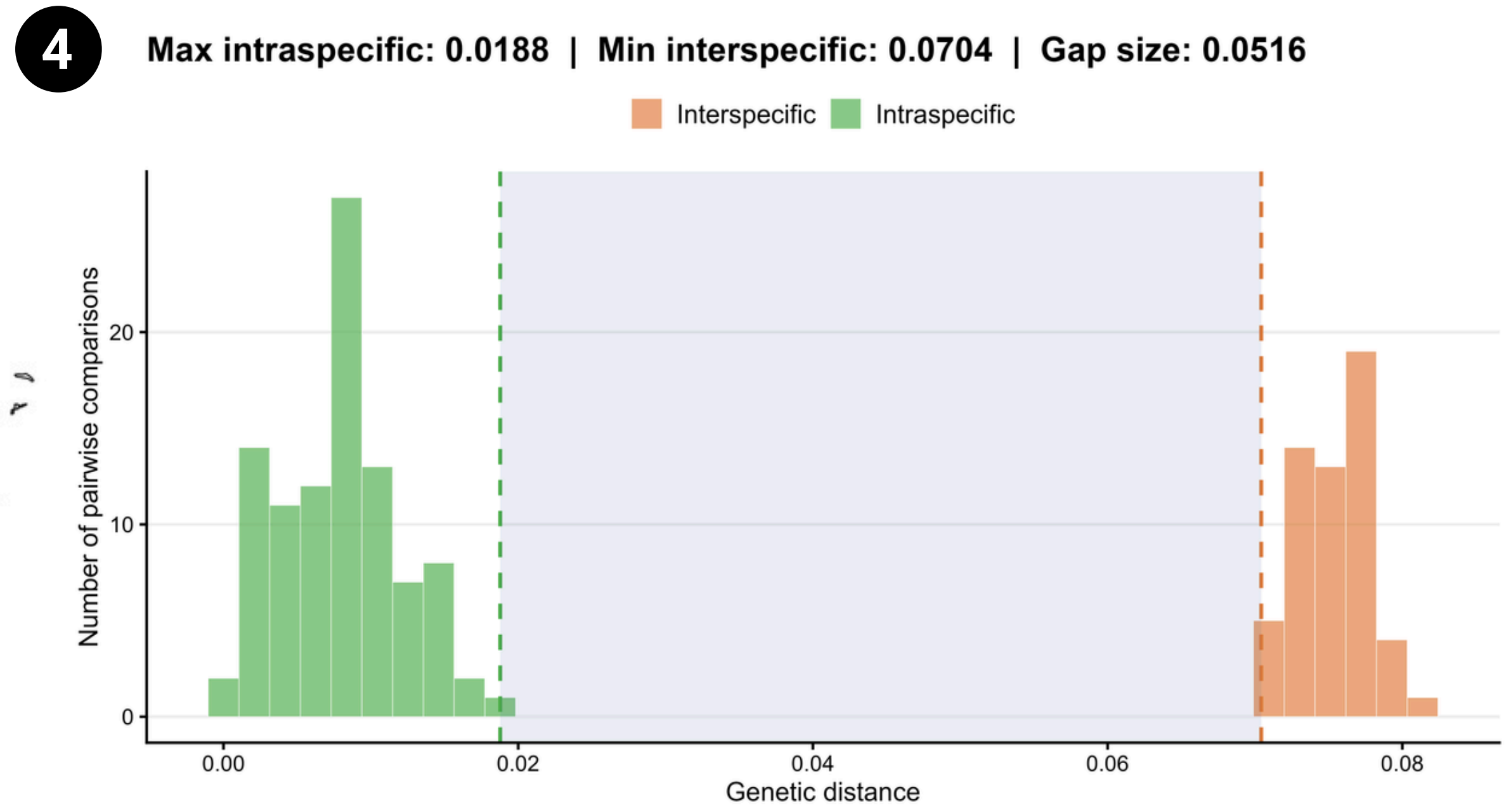

Figure 26

Fig. 26. Phylogenetic, geographic, and supporting evidence for candidate lineages in *Monophyllus redmani.* (1) Phylogenetic hypothesis based on COI sequences, with colored clades representing candidate lineages identified through molecular species delimitation. (2) Geographic distribution map, where colored points correspond to the lineages recovered in the phylogeny and black points represent the overall known distribution of the species complex. (3) Table of genetic distances (when sufficient data are available), reporting intra- and inter-lineage divergence among delimited groups. (4) Histogram of genetic distances illustrating the barcode gap. (5) Phylogenetic hypotheses from previous studies (when available), provided for comparison with our proposed delimitations. (6) Additional lines of evidence (occasionally available), such as geometric morphometrics or other independent datasets supporting lineage differentiation. Red star indicates holotype.

Note: *When this symbol ( ) appears next to the phylogeny, it indicates that the delimitation should be interpreted with caution, as additional sampling is needed. When it appears next to the table, it means that genetic distances are low; therefore, we recommend that these results also be considered with caution. In both cases, we have retained the delimitations as inferred by our methods.*

Phylloderma stenops

1

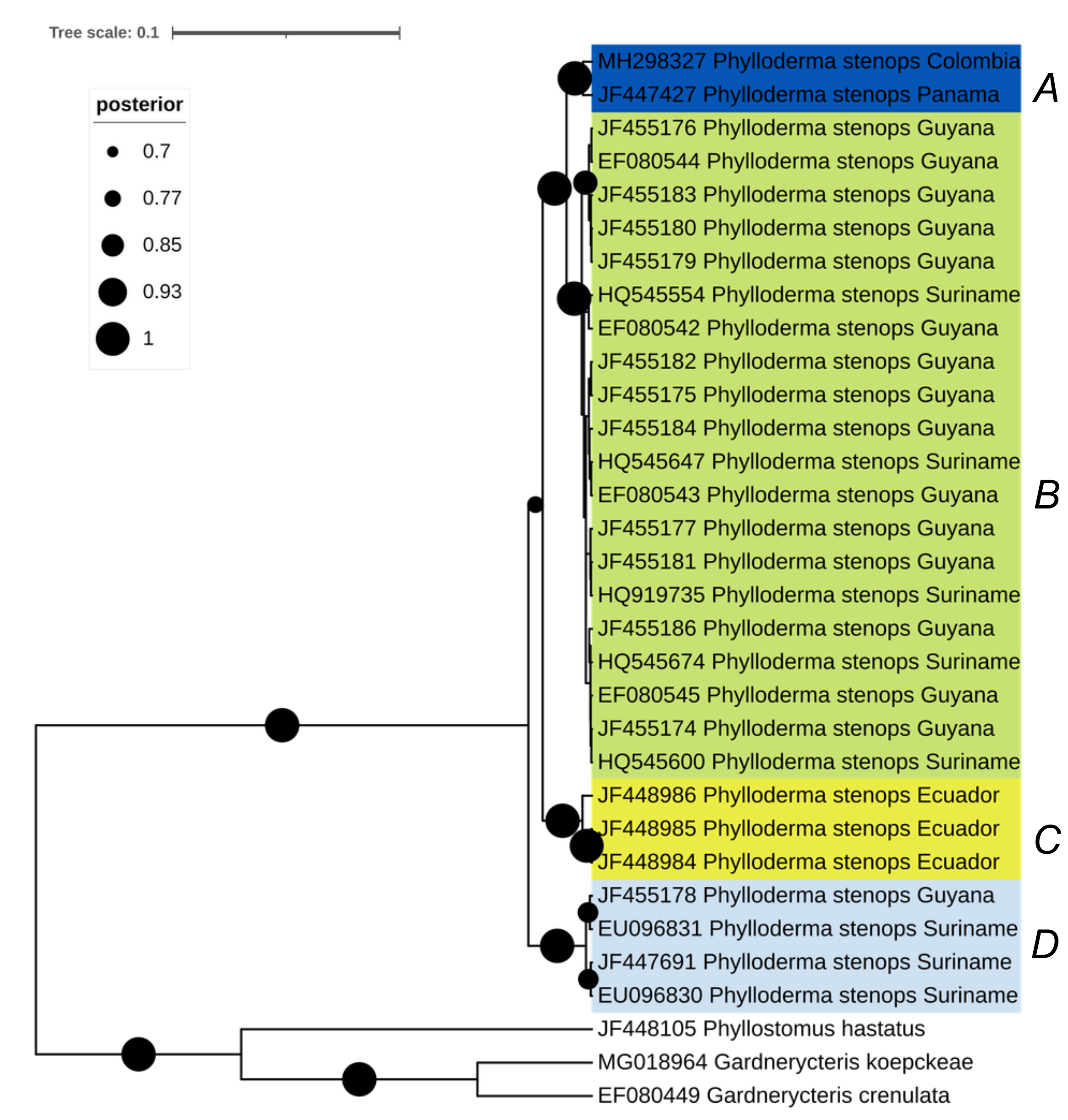

2

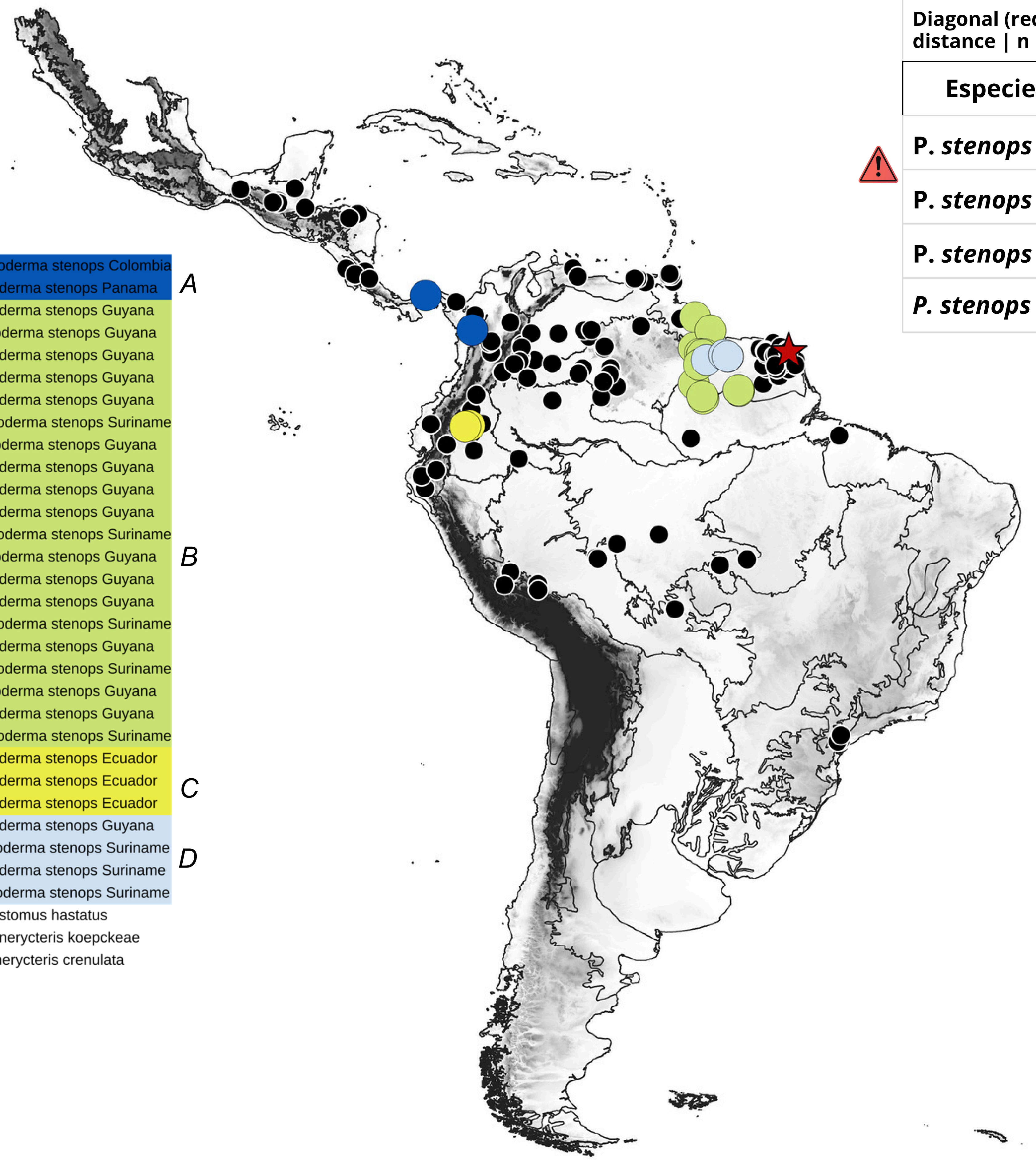

3

Mean genetic distances (Simple Distance)

Diagonal (red) = mean intraspecific distance | Off-diagonal (black) = mean interspecific distance | n = number of unique pairs

Especie	*P. stenops* A	*P. stenops* B	*P. stenops* C	*P. stenops* D
P. stenops A	0.0078 (n=1)	0.0171 (n=40)	0.0389 (n=6)	0.0442 (n=8)
P. stenops B	0.0171 (n=40)	0.0011 (n=190)	0.0335 (n=60)	0.0417 (n=80)
P. stenops C	0.0389 (n=6)	0.0335 (n=60)	0.0042 (n=3)	0.0398 (n=12)
P. stenops D	0.0442 (n=8)	0.0417 (n=80)	0.0398 (n=12)	0.0018 (n=6)

4

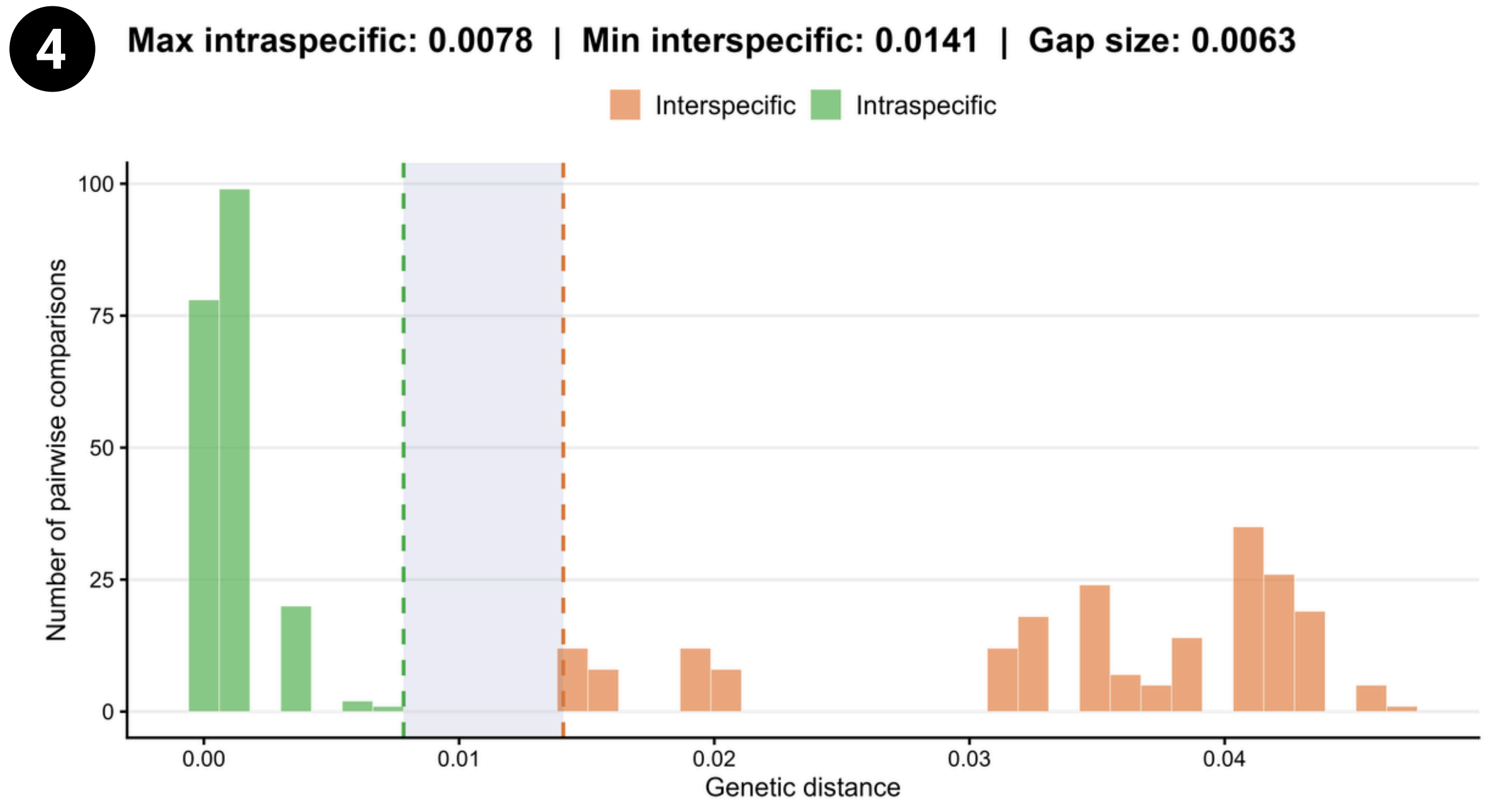

5 COI

Martinez-Ceron et al., (2019) Check List

Figure 27

Fig. 27. Phylogenetic, geographic, and supporting evidence for candidate lineages in *Phylloderma stenops.* (1) Phylogenetic hypothesis based on COI sequences, with colored clades representing candidate lineages identified through molecular species delimitation. (2) Geographic distribution map, where colored points correspond to the lineages recovered in the phylogeny and black points represent the overall known distribution of the species complex. (3) Table of genetic distances (when sufficient data are available), reporting intra- and inter-lineage divergence among delimited groups. (4) Histogram of genetic distances illustrating the barcode gap. (5) Phylogenetic hypotheses from previous studies (when available), provided for comparison with our proposed delimitations. (6) Additional lines of evidence (occasionally available), such as geometric morphometrics or other independent datasets supporting lineage differentiation. Red star indicates holotype.

Note: *When this symbol (⚠) appears next to the phylogeny, it indicates that the delimitation should be interpreted with caution, as additional sampling is needed. When it appears next to the table, it means that genetic distances are low; therefore, we recommend that these results also be considered with caution. In both cases, we have retained the delimitations as inferred by our methods.*

Phyllostomus hastatus

1

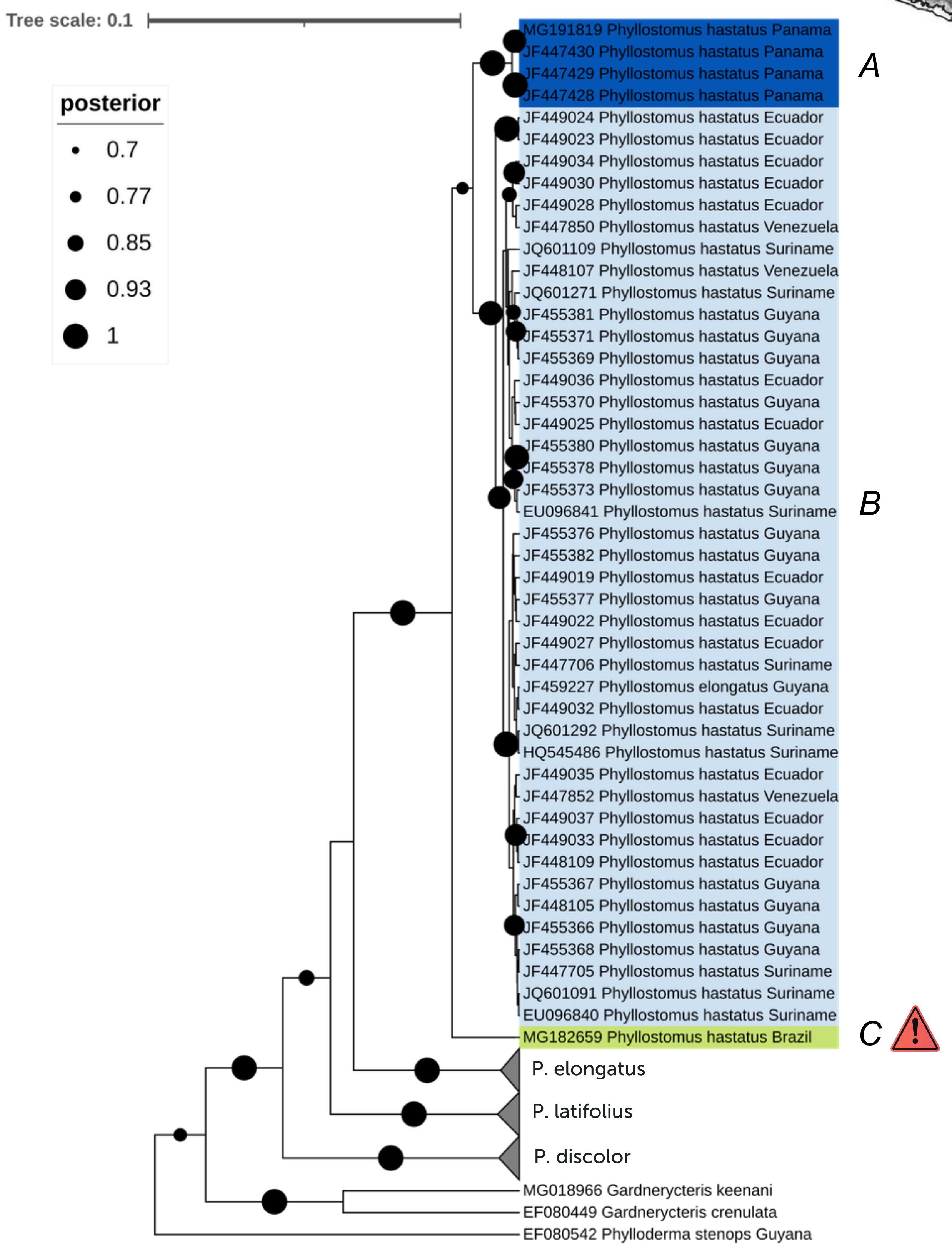

2

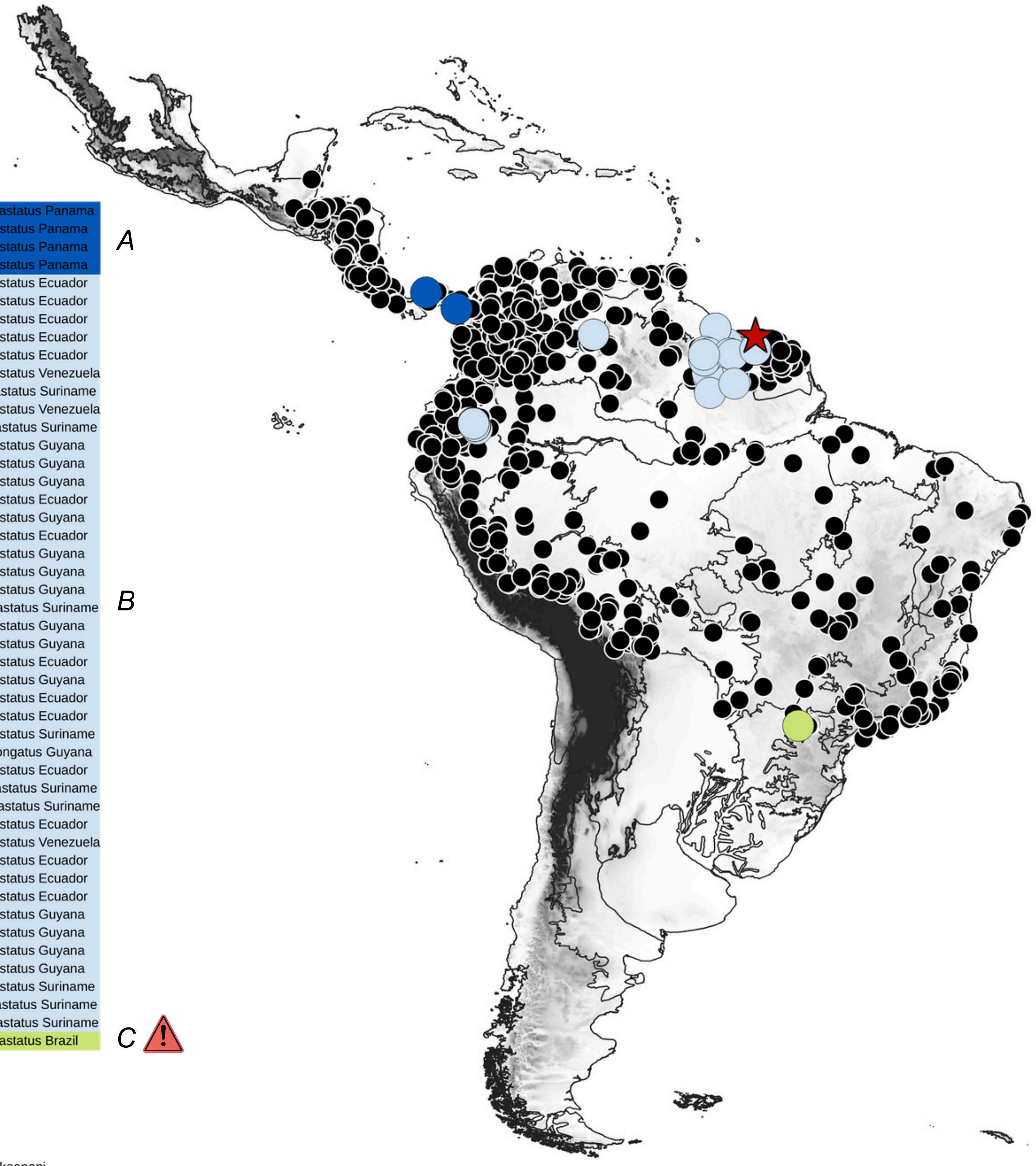

3

Mean genetic distances (Simple Distance)

Diagonal (red) = mean intraspecific distance | Off-diagonal (black) = mean interspecific distance | n = number of unique pairs

Especie	P. hastatus A	P. hastatus B	P. hastatus C
P. hastatus A	0.0039 (n=6)	0.0368 (n=168)	0.0654 (n=4)
P. hastatus B	0.0368 (n=168)	0.0118 (n=861)	0.0549 (n=42)
P. hastatus C	0.0654 (n=4)	0.0549 (n=42)	--

4 No barcoding gap | Max intraspecific: 0.1284 | Min interspecific: 0.0291 | Overlap

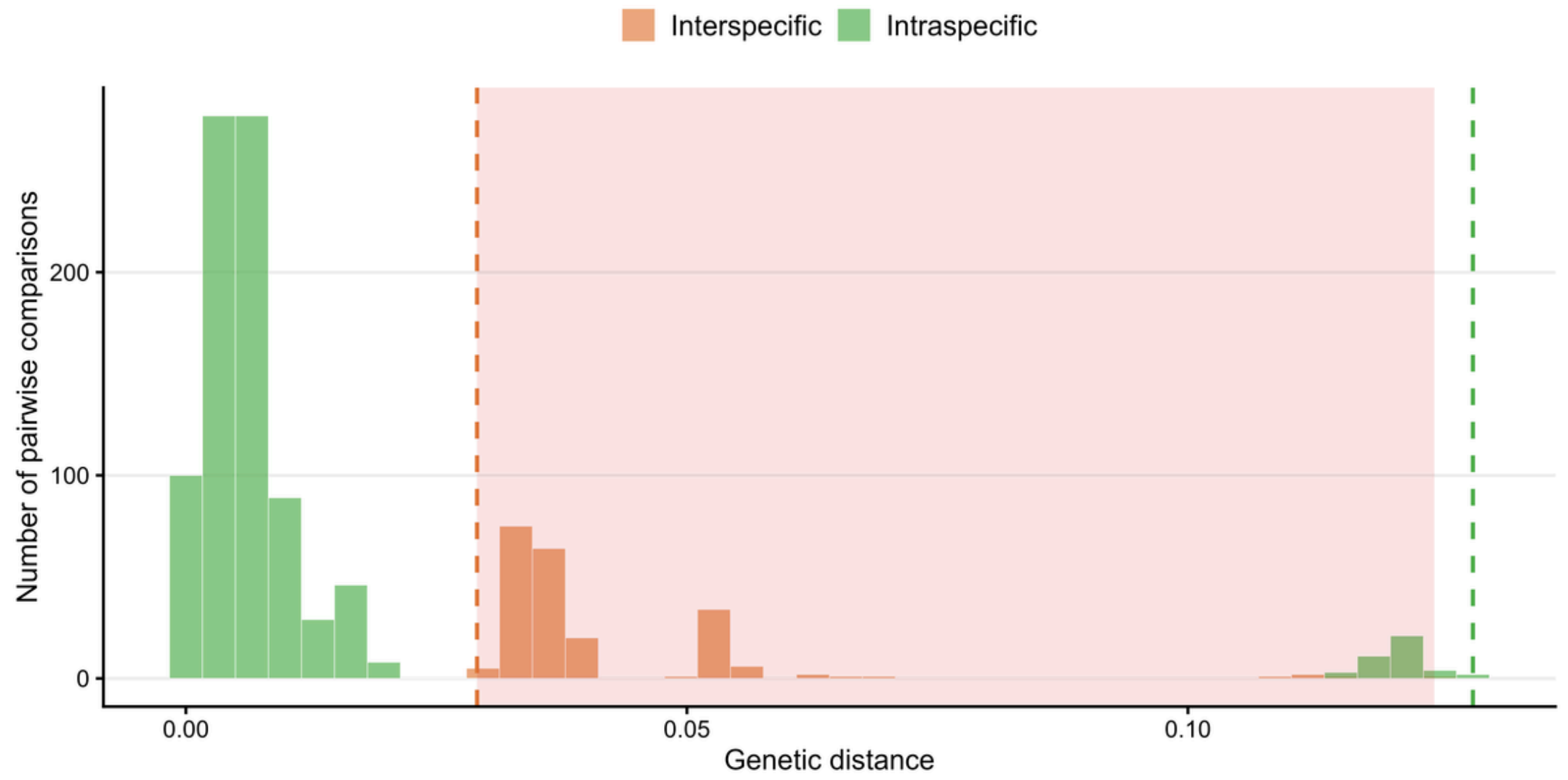

5 COI

Clare et al., (2011) PloS One

Figure 28

Fig. 28. Phylogenetic, geographic, and supporting evidence for candidate lineages in *Phyllostomus hastatus*. (1) Phylogenetic hypothesis based on COI sequences, with colored clades representing candidate lineages identified through molecular species delimitation. (2) Geographic distribution map, where colored points correspond to the lineages recovered in the phylogeny and black points represent the overall known distribution of the species complex. (3) Table of genetic distances (when sufficient data are available), reporting intra- and inter-lineage divergence among delimited groups. (4) Histogram of genetic distances illustrating the barcode gap. (5) Phylogenetic hypotheses from previous studies (when available), provided for comparison with our proposed delimitations. (6) Additional lines of evidence (occasionally available), such as geometric morphometrics or other independent datasets supporting lineage differentiation. Red star indicates holotype.

Note: *When this symbol (⚠) appears next to the phylogeny, it indicates that the delimitation should be interpreted with caution, as additional sampling is needed. When it appears next to the table, it means that genetic distances are low; therefore, we recommend that these results also be considered with caution. In both cases, we have retained the delimitations as inferred by our methods.*

Tonatia maresi

1

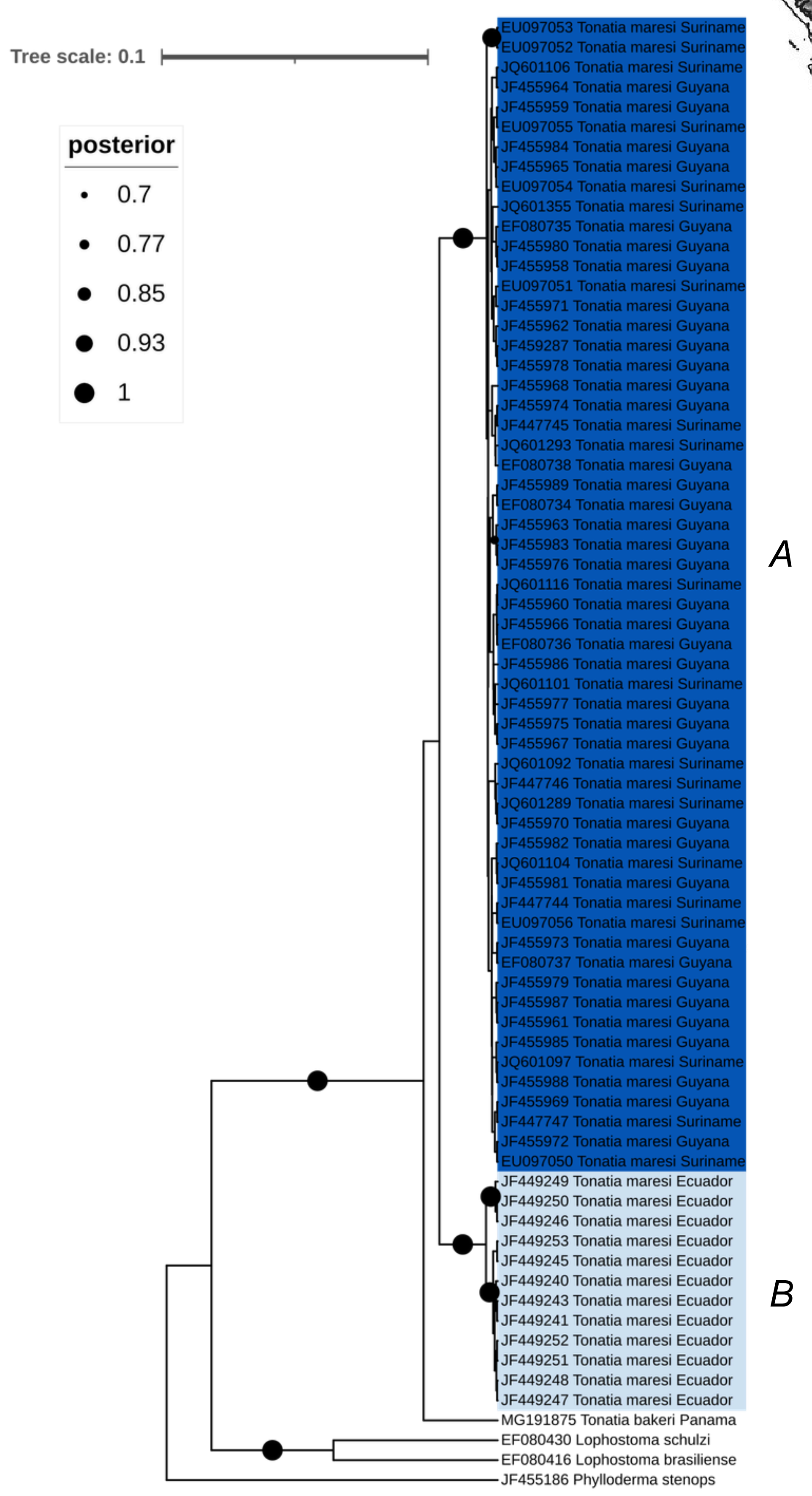

2

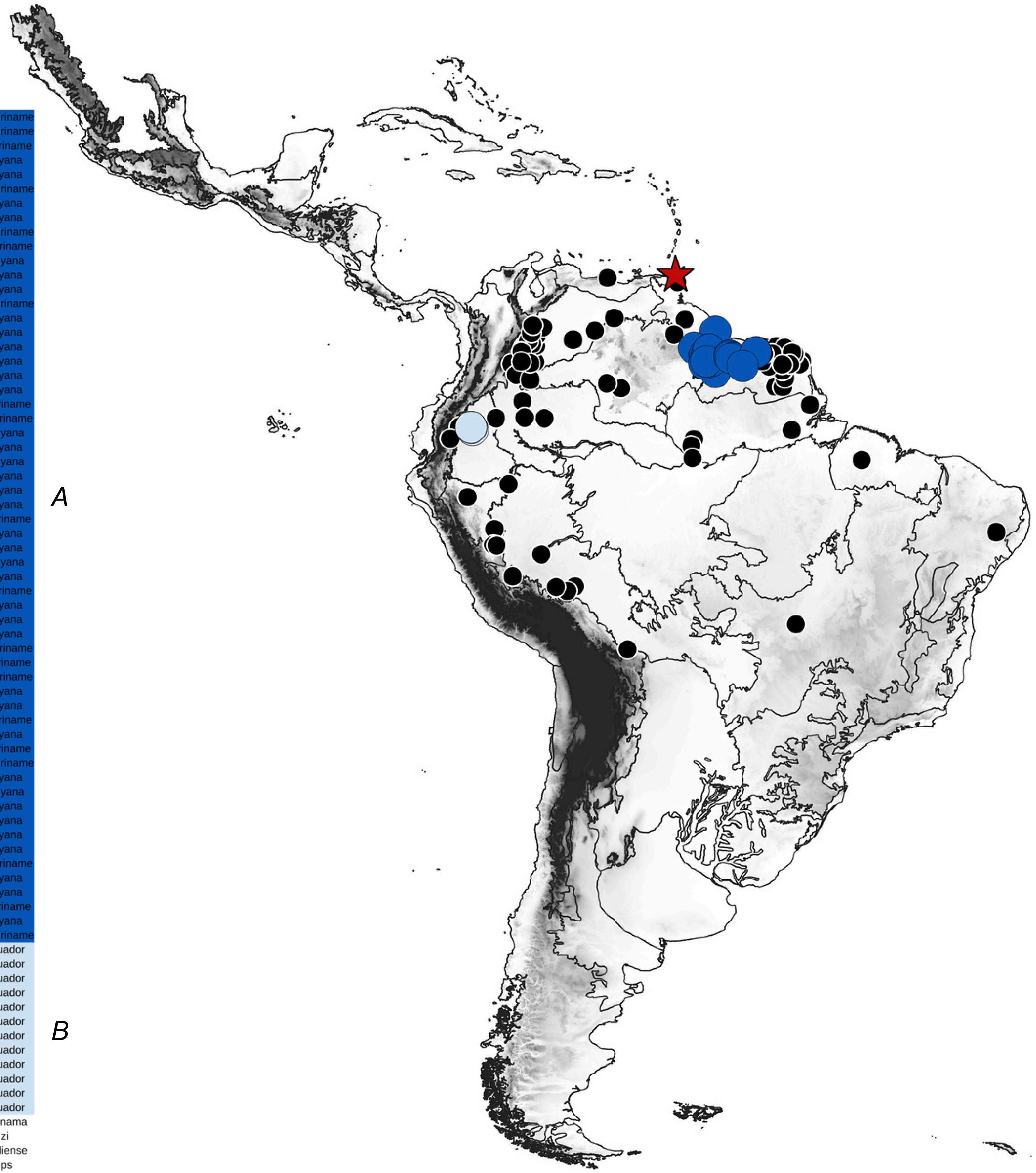

3

Mean genetic distances (Simple Distance)		
Diagonal (red) = mean intraspecific distance | Off-diagonal (black) = mean interspecific distance | n = number of unique pairs		
**Especie**	**T. *maresi* A**	**T. *maresi* B**
**T. *maresi* A**	0.0006 (n=1653)	0.0476 (n=696)
**T. *maresi* B**	0.0476 (n=696)	0.0025 (n=66)

4

Max intraspecific: 0.0061 | Min interspecific: 0.0459 | Gap size: 0.0398

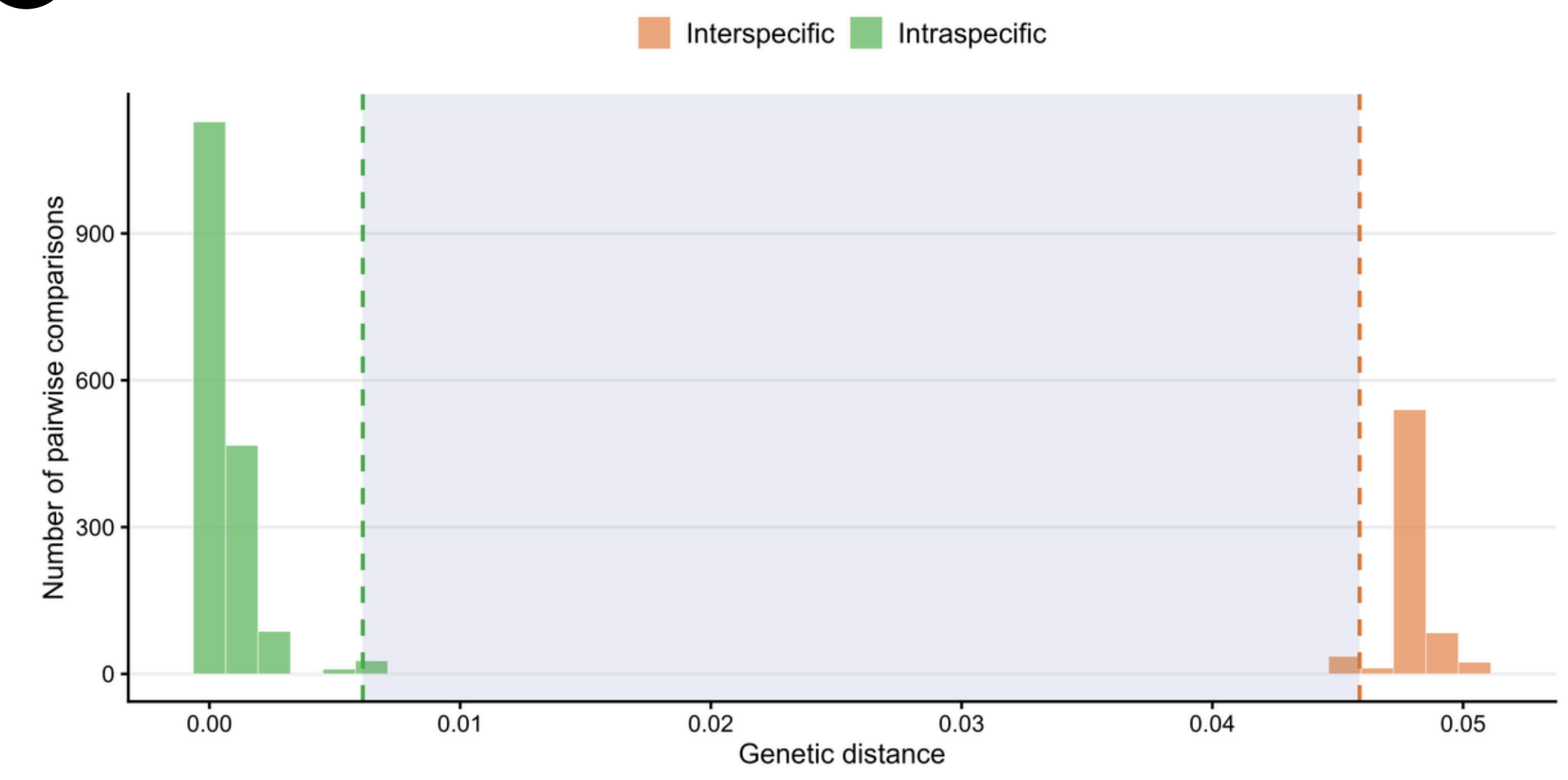

5 CYTB

Basantes et al., (2020) Zookeys

Figure 29

Fig. 29. Phylogenetic, geographic, and supporting evidence for candidate lineages in *Tonatia maresi.* (1) Phylogenetic hypothesis based on COI sequences, with colored clades representing candidate lineages identified through molecular species delimitation. (2) Geographic distribution map, where colored points correspond to the lineages recovered in the phylogeny and black points represent the overall known distribution of the species complex. (3) Table of genetic distances (when sufficient data are available), reporting intra- and inter-lineage divergence among delimited groups. (4) Histogram of genetic distances illustrating the barcode gap. (5) Phylogenetic hypotheses from previous studies (when available), provided for comparison with our proposed delimitations. (6) Additional lines of evidence (occasionally available), such as geometric morphometrics or other independent datasets supporting lineage differentiation. Red star indicates holotype.

Note: *When this symbol (⚠) appears next to the phylogeny, it indicates that the delimitation should be interpreted with caution, as additional sampling is needed. When it appears next to the table, it means that genetic distances are low; therefore, we recommend that these results also be considered with caution. In both cases, we have retained the delimitations as inferred by our methods.*

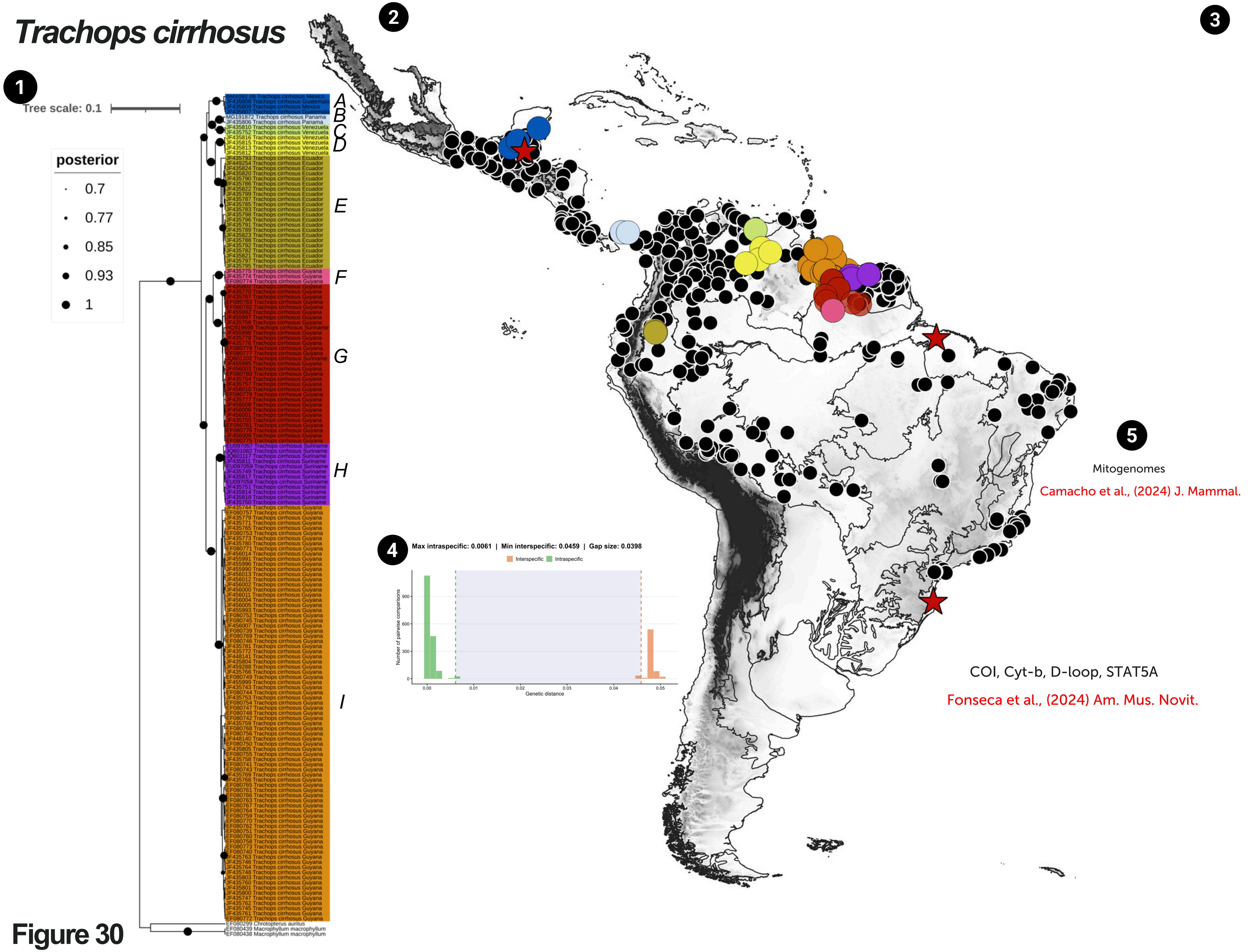

Figure 30

3

Mean genetic distances (Simple Distance)

Diagonal (red) = mean intraspecific distance | Off-diagonal (black) = mean interspecific distance | n = number of unique pairs

Especie	*T. cirrhosus* A	*T. cirrhosus* B	*T. cirrhosus* C	*T. cirrhosus* D	*T. cirrhosus* E	*T. cirrhosus* F	*T. cirrhosus* G	*T. cirrhosus* H	*T. cirrhosus* I
T. cirrhosus A	0.0016 (n=6)	0.0409 (n=8)	0.0527 (n=8)	0.0574 (n=16)	0.0451 (n=88)	0.0763 (n=12)	0.0623 (n=124)	0.0654 (n=48)	0.0562 (n=324)
T. cirrhosus B	0.0409 (n=8)	0.0047 (n=1)	0.0338 (n=4)	0.0527 (n=8)	0.0499 (n=44)	0.0692 (n=6)	0.0581 (n=62)	0.0607 (n=24)	0.0534 (n=162)
T. cirrhosus C	0.0527 (n=8)	0.0338 (n=4)	0.0000 (n=1)	0.0645 (n=8)	0.0614 (n=44)	0.0661 (n=6)	0.0682 (n=62)	0.0630 (n=24)	0.0604 (n=162)
T. cirrhosus D	0.0574 (n=16)	0.0527 (n=8)	0.0645 (n=8)	0.0000 (n=6)	0.0318 (n=88)	0.0771 (n=12)	0.0728 (n=124)	0.0722 (n=48)	0.0699 (n=324)
T. cirrhosus E	0.0451 (n=88)	0.0499 (n=44)	0.0614 (n=44)	0.0318 (n=88)	0.0031 (n=231)	0.0709 (n=66)	0.0608 (n=682)	0.0662 (n=264)	0.0607 (n=1782)
T. cirrhosus F	0.0763 (n=12)	0.0692 (n=6)	0.0661 (n=6)	0.0771 (n=12)	0.0709 (n=66)	0.0000 (n=3)	0.0401 (n=93)	0.0646 (n=36)	0.0589 (n=243)
T. cirrhosus G	0.0623 (n=124)	0.0581 (n=62)	0.0682 (n=62)	0.0728 (n=124)	0.0608 (n=682)	0.0401 (n=93)	0.0013 (n=465)	0.0541 (n=372)	0.0515 (n=2511)
T. cirrhosus H	0.0654 (n=48)	0.0607 (n=24)	0.0630 (n=24)	0.0722 (n=48)	0.0662 (n=264)	0.0646 (n=36)	0.0541 (n=372)	0.0003 (n=66)	0.0233 (n=972)
T. cirrhosus I	0.0562 (n=324)	0.0534 (n=162)	0.0604 (n=162)	0.0699 (n=324)	0.0607 (n=1782)	0.0589 (n=243)	0.0515 (n=2511)	0.0233 (n=972)	0.0021 (n=3240)

Fig. 30. Phylogenetic, geographic, and supporting evidence for candidate lineages in *Trachops cirrhosus*. (1) Phylogenetic hypothesis based on COI sequences, with colored clades representing candidate lineages identified through molecular species delimitation. (2) Geographic distribution map, where colored points correspond to the lineages recovered in the phylogeny and black points represent the overall known distribution of the species complex. (3) Table of genetic distances (when sufficient data are available), reporting intra- and inter-lineage divergence among delimited groups. (4) Histogram of genetic distances illustrating the barcode gap. (5) Phylogenetic hypotheses from previous studies (when available), provided for comparison with our proposed delimitations. (6) Additional lines of evidence (occasionally available), such as geometric morphometrics or other independent datasets supporting lineage differentiation. Red star indicates holotype.

Note: *When this symbol (⚠) appears next to the phylogeny, it indicates that the delimitation should be interpreted with caution, as additional sampling is needed. When it appears next to the table, it means that genetic distances are low; therefore, we recommend that these results also be considered with caution. In both cases, we have retained the delimitations as inferred by our methods.*

Trinycteris nicefori

1

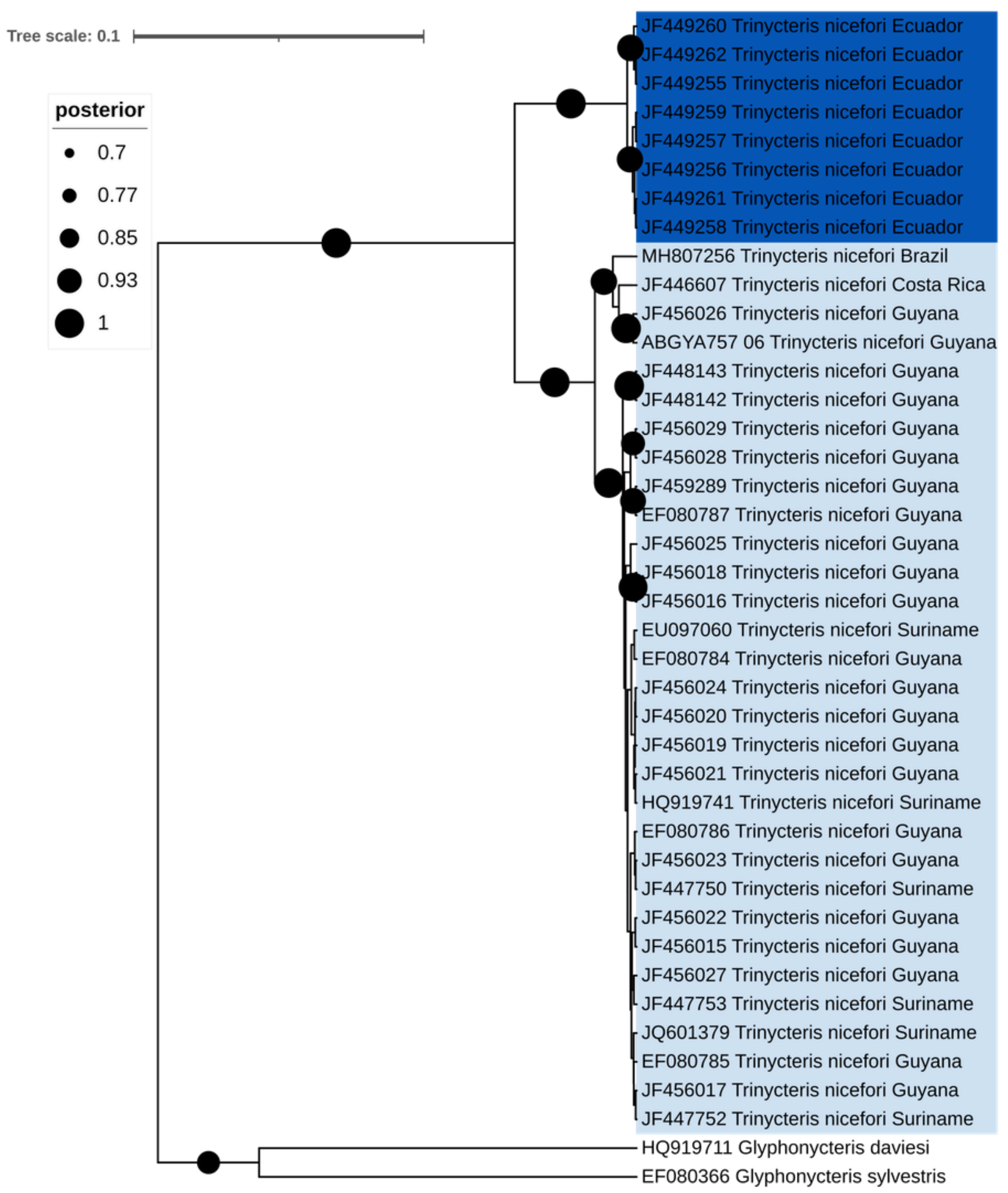

2

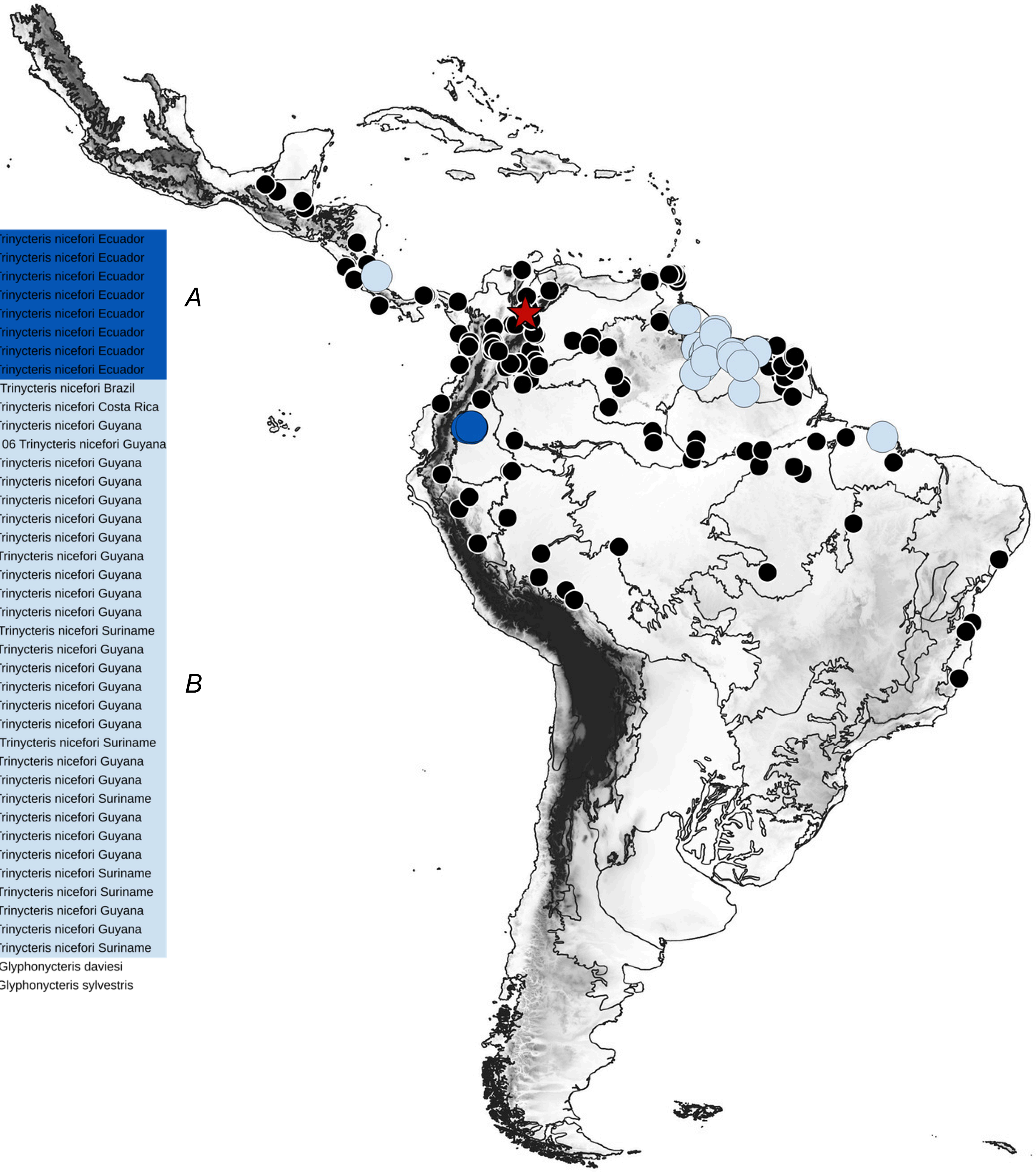

3

Mean genetic distances (Simple Distance)

Diagonal (red) = mean intraspecific distance | Off-diagonal (black) = mean interspecific distance | n = number of unique pairs

Especie	T. *nicefori* A	T. *nicefori* B
T. *nicefori* A	0.0017 (n=28)	0.0695 (n=248)
T. *nicefori* B	0.0695 (n=248)	0.0063 (n=465)

4

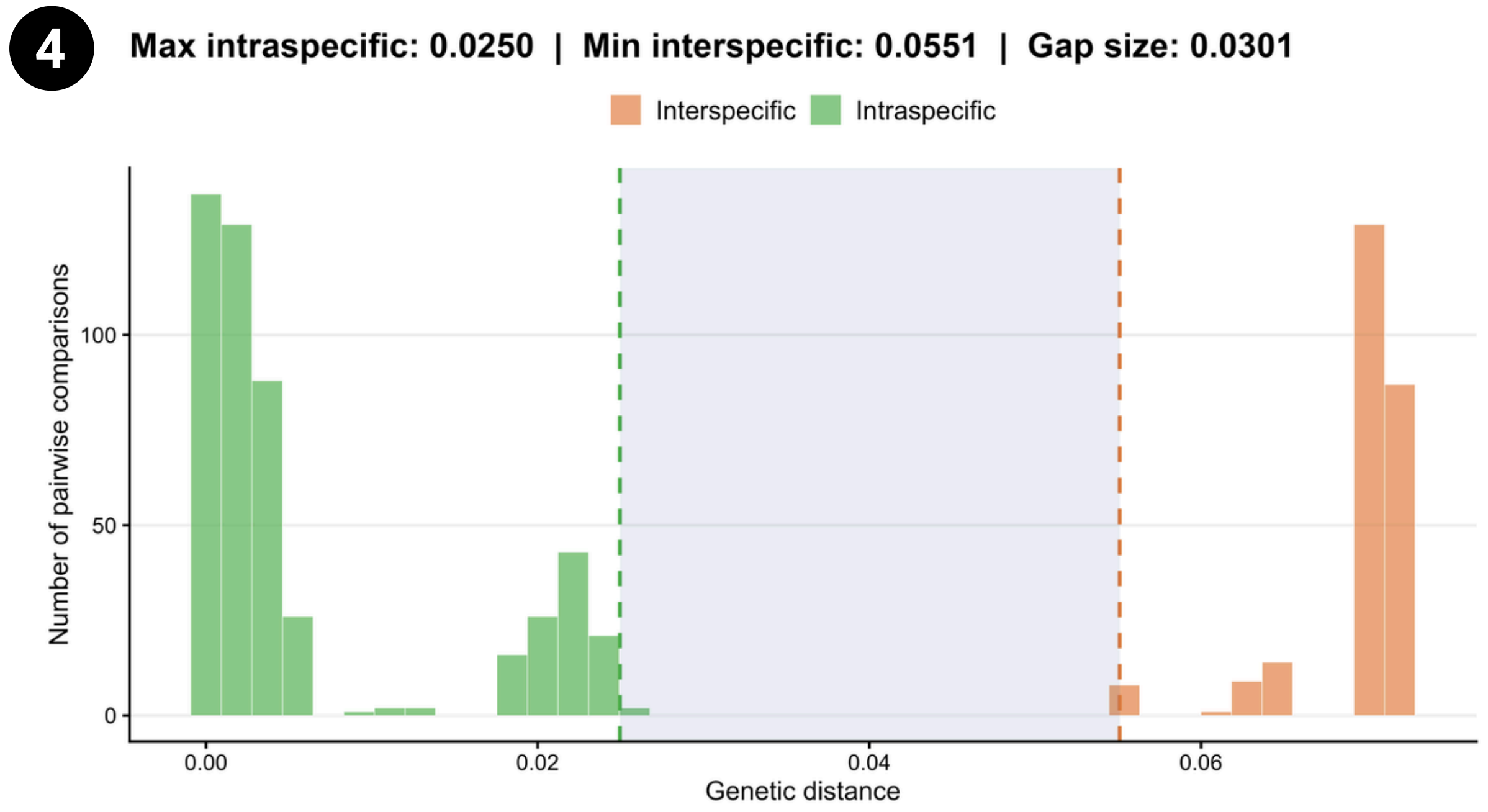

5

COI

Clare et al., (2011) PloS One

Figure 31

Fig. 31. Phylogenetic, geographic, and supporting evidence for candidate lineages in *Trinycteris nicefori*. (1) Phylogenetic hypothesis based on COI sequences, with colored clades representing candidate lineages identified through molecular species delimitation. (2) Geographic distribution map, where colored points correspond to the lineages recovered in the phylogeny and black points represent the overall known distribution of the species complex. (3) Table of genetic distances (when sufficient data are available), reporting intra- and inter-lineage divergence among delimited groups. (4) Histogram of genetic distances illustrating the barcode gap. (5) Phylogenetic hypotheses from previous studies (when available), provided for comparison with our proposed delimitations. (6) Additional lines of evidence (occasionally available), such as geometric morphometrics or other independent datasets supporting lineage differentiation. Red star indicates holotype.

Note: *When this symbol (⚠) appears next to the phylogeny, it indicates that the delimitation should be interpreted with caution, as additional sampling is needed. When it appears next to the table, it means that genetic distances are low; therefore, we recommend that these results also be considered with caution. In both cases, we have retained the delimitations as inferred by our methods.*

Histiotus montanus

1

2

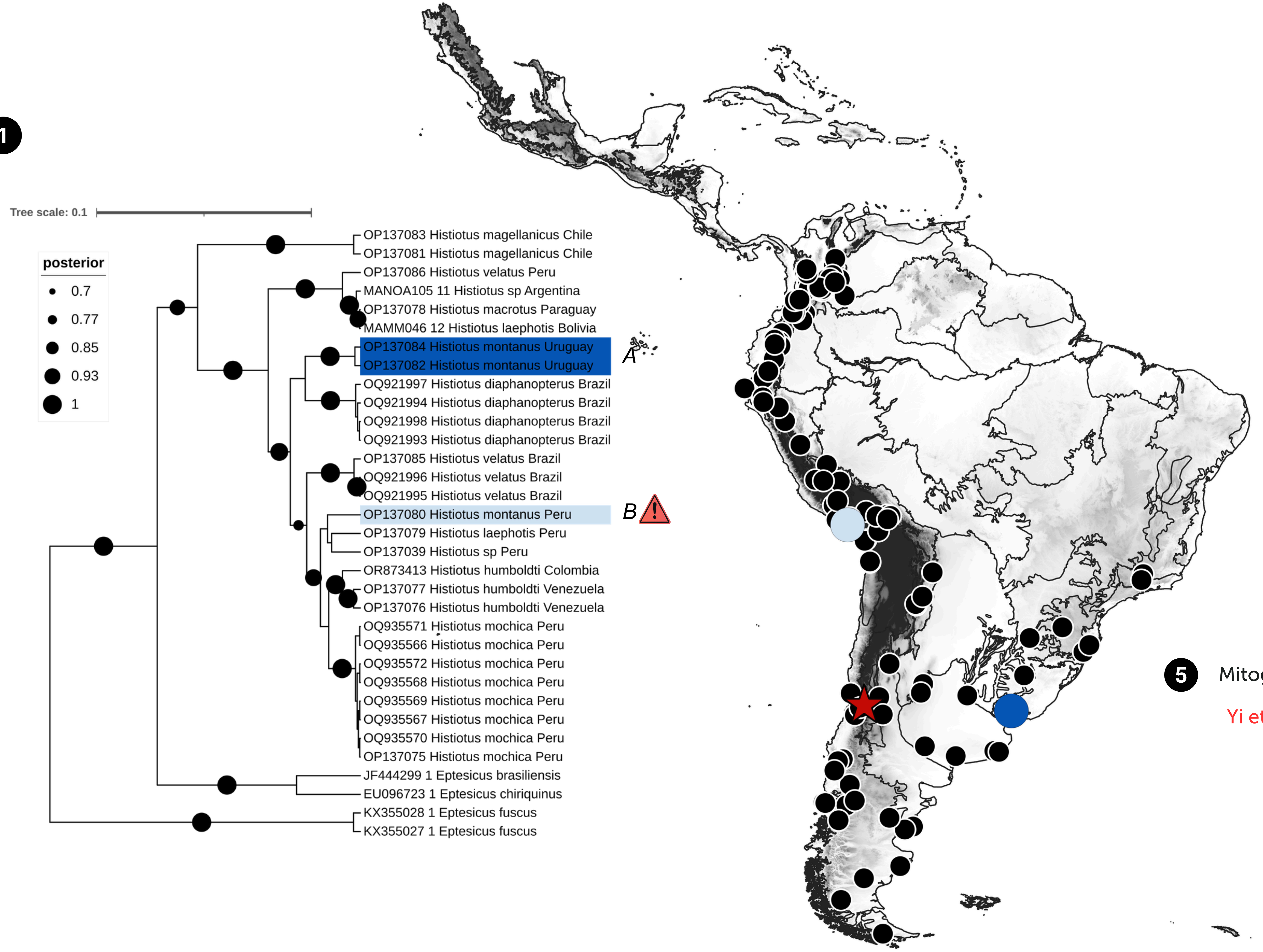

3

Mean genetic distances (Simple Distance)

Diagonal (red) = mean intraspecific distance | Off-diagonal (black) = mean interspecific distance | n = number of unique pairs

Especie	H. *montanus* A	H. *montanus* B
H. *montanus* A	0.0046 (n=1)	0.0499 (n=2)
H. *montanus* B	0.0499 (n=2)	--

5 Mitogenomes and UCEs

Yi et al., (2024) Mamm. Biol.

Figure 32

Fig. 32. Phylogenetic, geographic, and supporting evidence for candidate lineages in *Histiotus montanus*. (1) Phylogenetic hypothesis based on COI sequences, with colored clades representing candidate lineages identified through molecular species delimitation. (2) Geographic distribution map, where colored points correspond to the lineages recovered in the phylogeny and black points represent the overall known distribution of the species complex. (3) Table of genetic distances (when sufficient data are available), reporting intra- and inter-lineage divergence among delimited groups. (4) Histogram of genetic distances illustrating the barcode gap. (5) Phylogenetic hypotheses from previous studies (when available), provided for comparison with our proposed delimitations. (6) Additional lines of evidence (occasionally available), such as geometric morphometrics or other independent datasets supporting lineage differentiation. Red star indicates holotype.

Note: *When this symbol (⚠) appears next to the phylogeny, it indicates that the delimitation should be interpreted with caution, as additional sampling is needed. When it appears next to the table, it means that genetic distances are low; therefore, we recommend that these results also be considered with caution. In both cases, we have retained the delimitations as inferred by our methods.*

Histiotus velatus

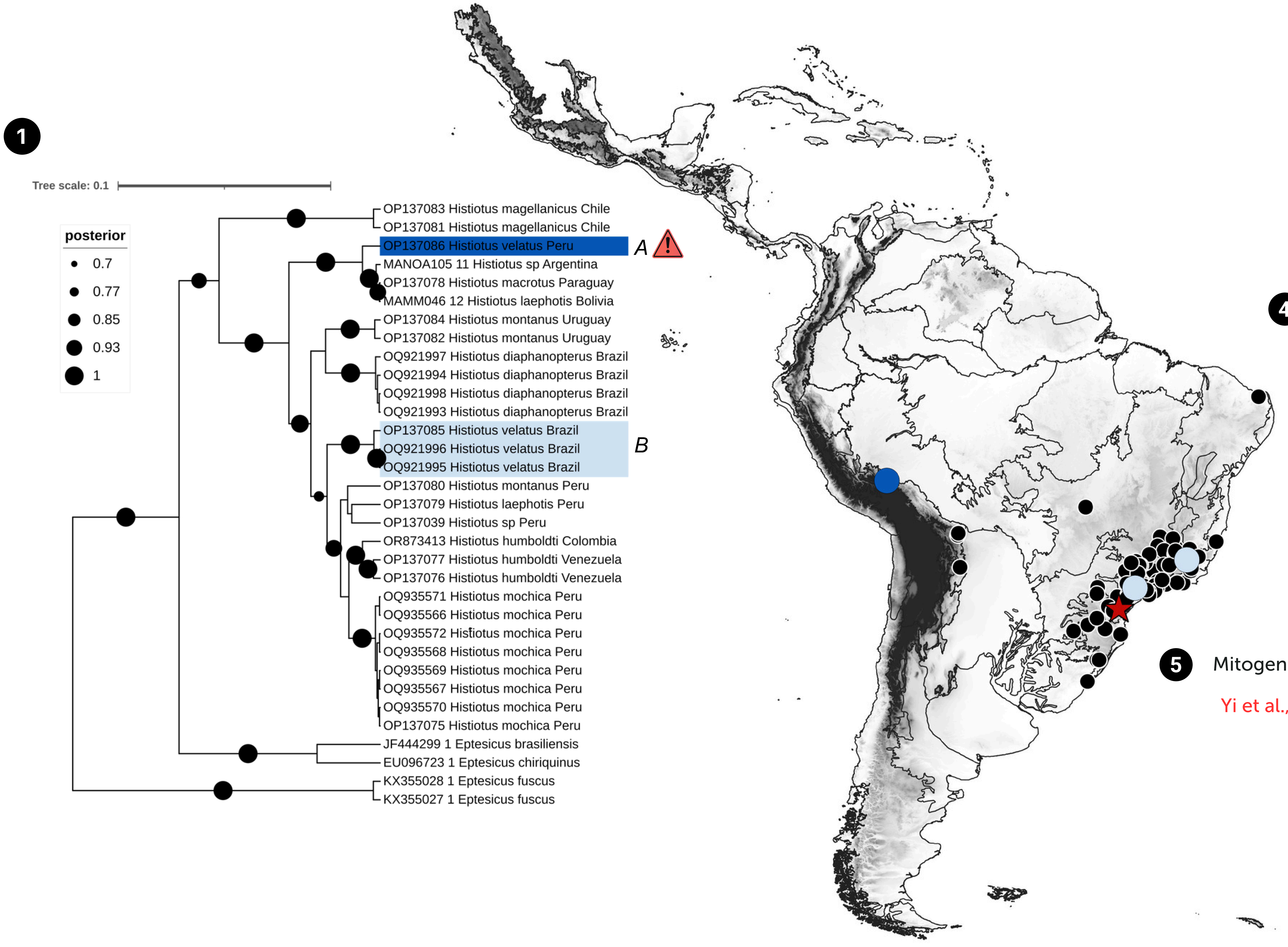

3

Mean genetic distances (Simple Distance)		
Diagonal (red) = mean intraspecific distance | Off-diagonal (black) = mean interspecific distance | n = number of unique pairs		
**Especie**	**H. *velatus* A**	**H. *velatus* B**
**H. *velatus* A**	--	0.0819 (n=3)
**H. *velatus* B**	0.0819 (n=3)	0.0041 (n=3)

4

Max intraspecific: 0.0061 | Min interspecific: 0.0814 | Gap size: 0.0753

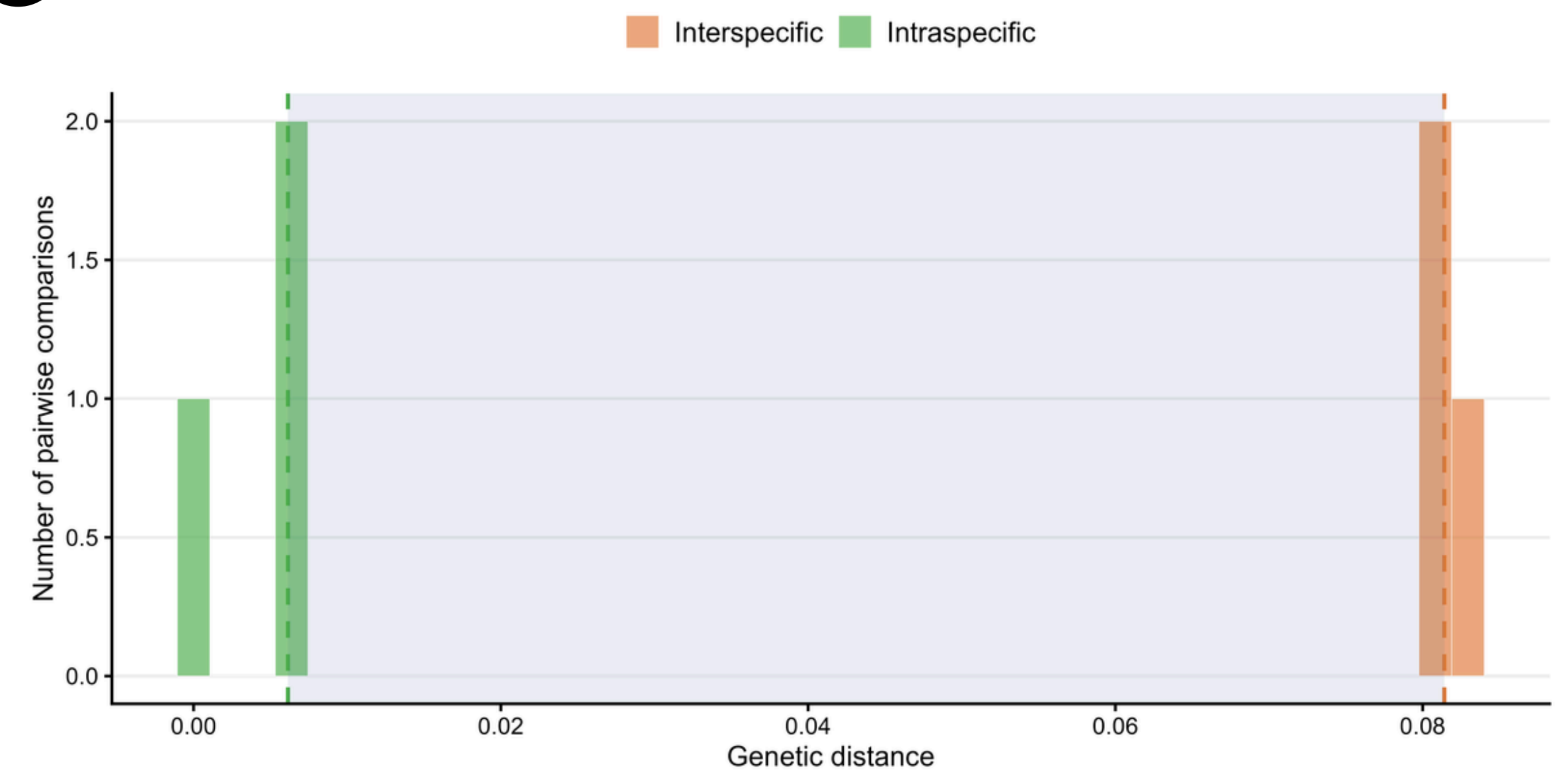

5 Mitogenomes and UCEs

Yi et al., (2024) Mamm. Biol.

Figure 33

Fig. 33. Phylogenetic, geographic, and supporting evidence for candidate lineages in *Histiotus velatus.* (1) Phylogenetic hypothesis based on COI sequences, with colored clades representing candidate lineages identified through molecular species delimitation. (2) Geographic distribution map, where colored points correspond to the lineages recovered in the phylogeny and black points represent the overall known distribution of the species complex. (3) Table of genetic distances (when sufficient data are available), reporting intra- and inter-lineage divergence among delimited groups. (4) Histogram of genetic distances illustrating the barcode gap. (5) Phylogenetic hypotheses from previous studies (when available), provided for comparison with our proposed delimitations. (6) Additional lines of evidence (occasionally available), such as geometric morphometrics or other independent datasets supporting lineage differentiation. Red star indicates holotype.

Note: *When this symbol (⚠) appears next to the phylogeny, it indicates that the delimitation should be interpreted with caution, as additional sampling is needed. When it appears next to the table, it means that genetic distances are low; therefore, we recommend that these results also be considered with caution. In both cases, we have retained the delimitations as inferred by our methods.*

Lasiurus ega

1

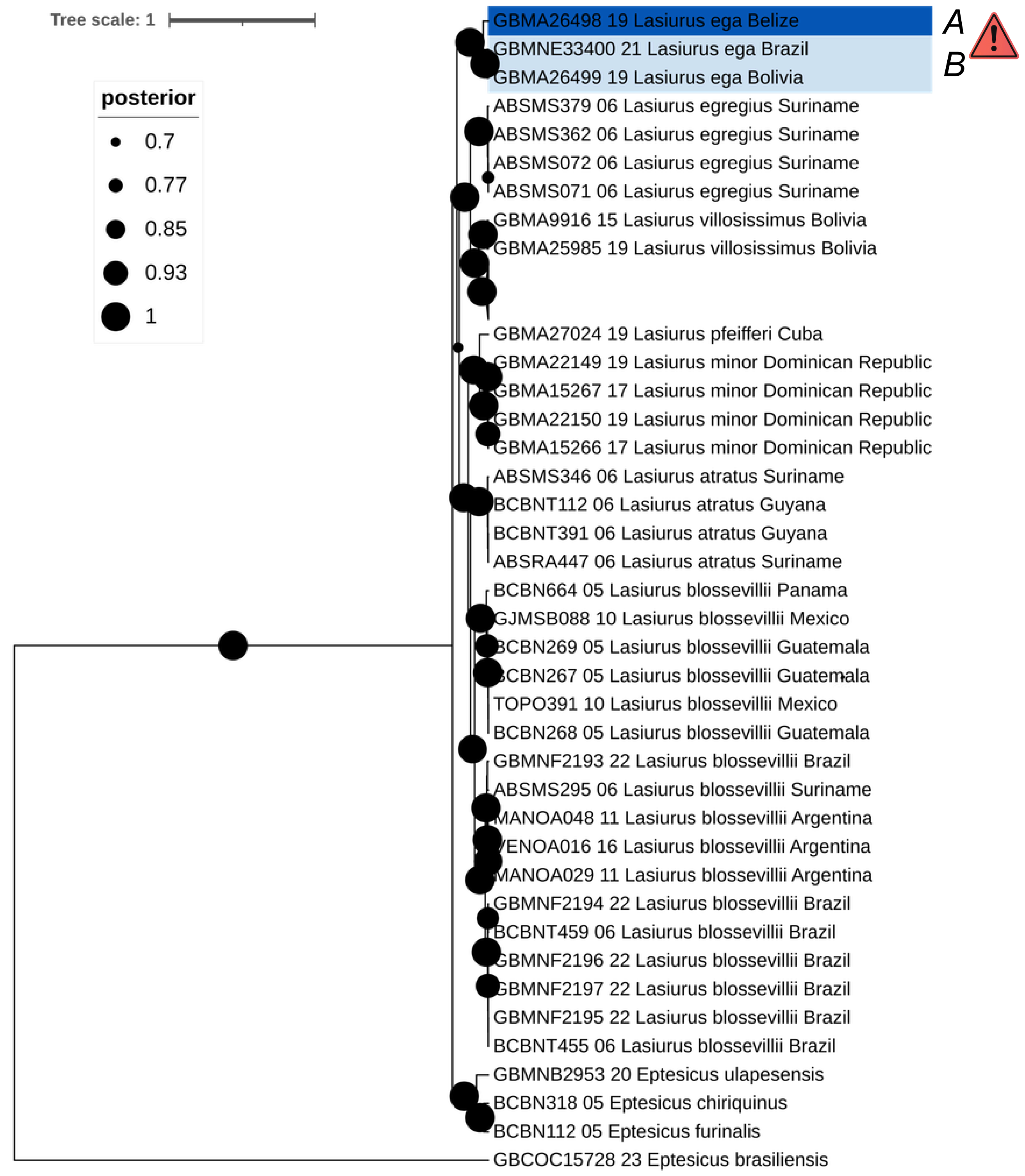

2

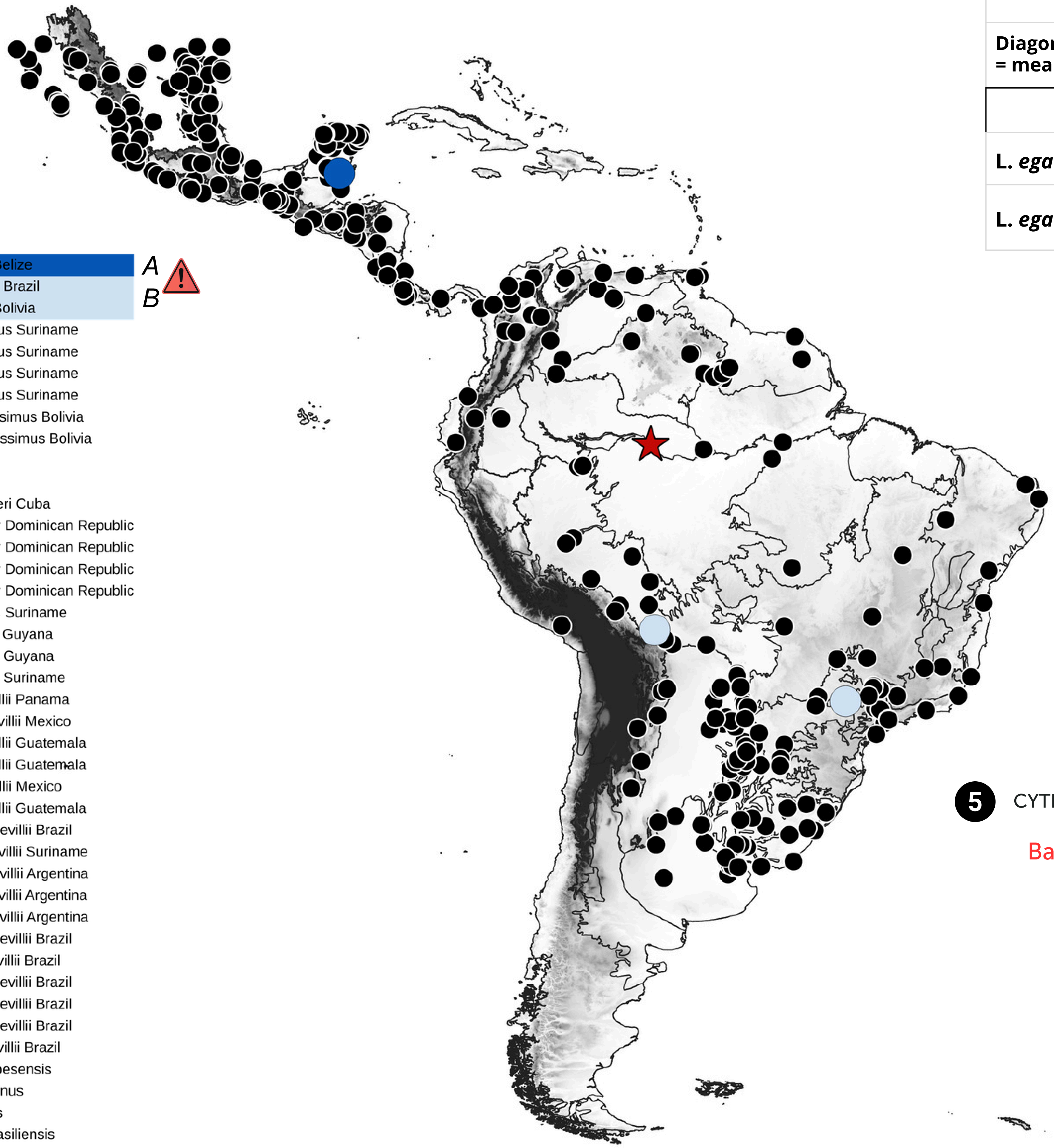

3

Mean genetic distances (Simple Distance)

Diagonal (red) = mean intraspecific distance | Off-diagonal (black) = mean interspecific distance | n = number of unique pairs

Especie	L. *ega* A	L. *ega* B
L. *ega* A	--	0.0515 (n=2)
L. *ega* B	0.0515 (n=2)	0.0186 (n=1)

5 CYTB, ND1, ND2, DBY

Baird et al., (2015) J. Mammal.

Figure 34

Fig. 34. Phylogenetic, geographic, and supporting evidence for candidate lineages in *Lasiurus ega*. (1) Phylogenetic hypothesis based on COI sequences, with colored clades representing candidate lineages identified through molecular species delimitation. (2) Geographic distribution map, where colored points correspond to the lineages recovered in the phylogeny and black points represent the overall known distribution of the species complex. (3) Table of genetic distances (when sufficient data are available), reporting intra- and inter-lineage divergence among delimited groups. (4) Histogram of genetic distances illustrating the barcode gap. (5) Phylogenetic hypotheses from previous studies (when available), provided for comparison with our proposed delimitations. (6) Additional lines of evidence (occasionally available), such as geometric morphometrics or other independent datasets supporting lineage differentiation. Red star indicates holotype.

Note: *When this symbol (⚠) appears next to the phylogeny, it indicates that the delimitation should be interpreted with caution, as additional sampling is needed. When it appears next to the table, it means that genetic distances are low; therefore, we recommend that these results also be considered with caution. In both cases, we have retained the delimitations as inferred by our methods.*

Myotis nigricans

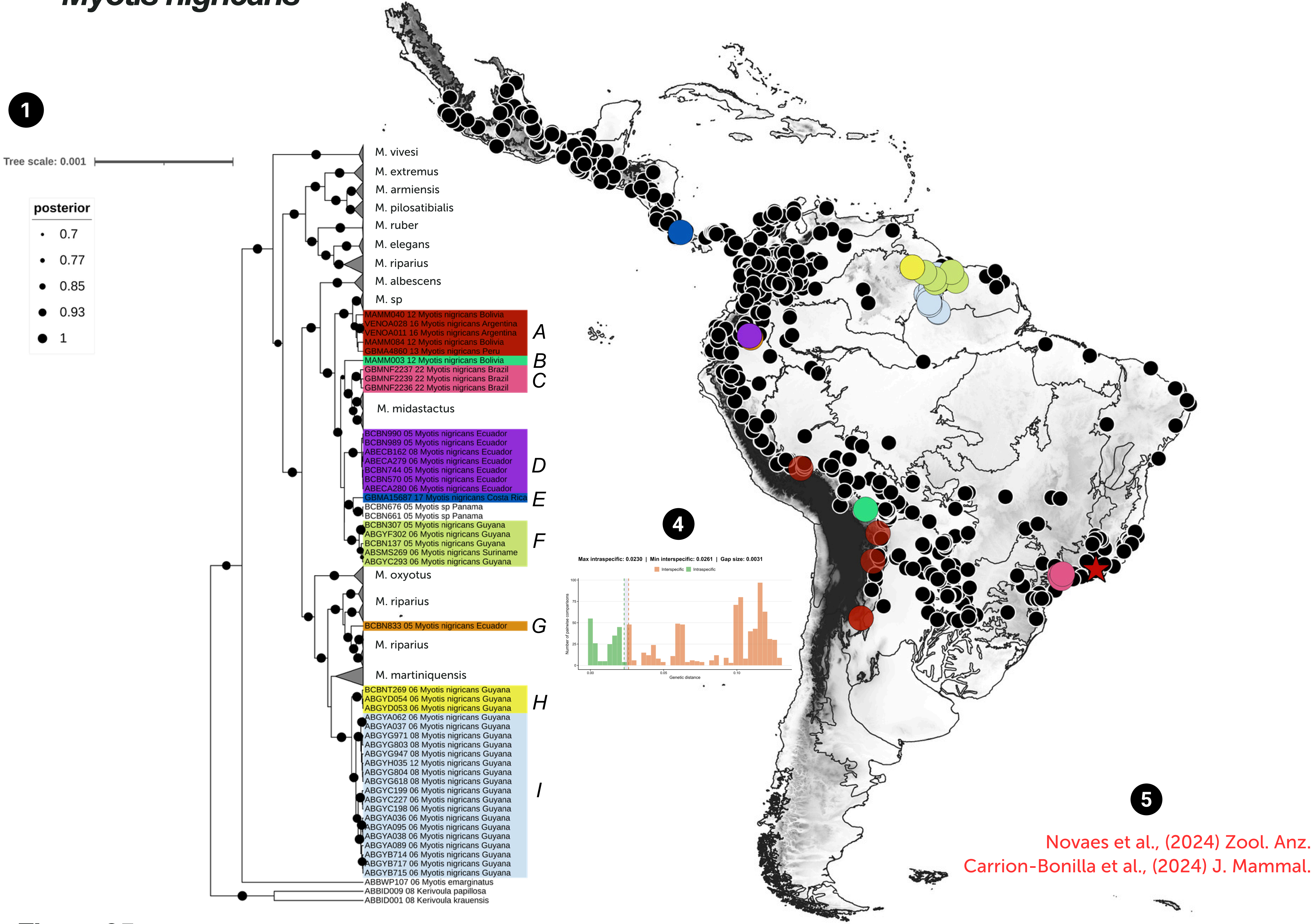

3

Mean genetic distances (Simple Distance)

Diagonal (red) = mean intraspecific distance | Off-diagonal (black) = mean interspecific distance | n = number of unique pairs

Especie	*M. nigricans* A	*M. nigricans* B	*M. nigricans* C	*M. nigricans* D	*M. nigricans* E	*M. nigricans* F	*M. nigricans* G	*M. nigricans* H	*M. nigricans* I
M. nigricans A	0.0130 (n=10)	0.0613 (n=5)	0.0709 (n=15)	0.0610 (n=35)	0.0624 (n=5)	0.0596 (n=25)	0.0956 (n=5)	0.1100 (n=15)	0.1160 (n=90)
M. nigricans B	0.0613 (n=5)		0.0492 (n=3)	0.0445 (n=7)	0.0415 (n=1)	0.0427 (n=5)	0.1106 (n=1)	0.1152 (n=3)	0.1156 (n=18)
M. nigricans C	0.0709 (n=15)	0.0492 (n=3)	0.0061 (n=3)	0.0620 (n=21)	0.0568 (n=3)	0.0589 (n=15)	0.1065 (n=3)	0.1152 (n=9)	0.1223 (n=54)
M. nigricans D	0.0610 (n=35)	0.0445 (n=7)	0.0620 (n=21)	0.0000 (n=21)	0.0461 (n=7)	0.0393 (n=35)	0.0922 (n=7)	0.0998 (n=21)	0.1008 (n=126)
M. nigricans E	0.0624 (n=5)	0.0415 (n=1)	0.0568 (n=3)	0.0461 (n=7)		0.0406 (n=5)	0.0937 (n=1)	0.1121 (n=3)	0.1142 (n=18)
M. nigricans F	0.0596 (n=25)	0.0427 (n=5)	0.0589 (n=15)	0.0393 (n=35)	0.0406 (n=5)	0.0065 (n=10)	0.1029 (n=5)	0.1195 (n=15)	0.1173 (n=90)
M. nigricans G	0.0956 (n=5)	0.1106 (n=1)	0.1065 (n=3)	0.0922 (n=7)	0.0937 (n=1)	0.1029 (n=5)		0.0722 (n=3)	0.0845 (n=18)
M. nigricans H	0.1100 (n=15)	0.1152 (n=3)	0.1152 (n=9)	0.0998 (n=21)	0.1121 (n=3)	0.1195 (n=15)	0.0722 (n=3)	0.0000 (n=3)	0.0272 (n=54)
M. nigricans I	0.1160 (n=90)	0.1156 (n=18)	0.1223 (n=54)	0.1008 (n=126)	0.1142 (n=18)	0.1173 (n=90)	0.0845 (n=18)	0.0272 (n=54)	0.0122 (n=153)

Figure 35

Fig. 35. Phylogenetic, geographic, and supporting evidence for candidate lineages in *Myotis nigricans.* (1) Phylogenetic hypothesis based on COI sequences, with colored clades representing candidate lineages identified through molecular species delimitation. (2) Geographic distribution map, where colored points correspond to the lineages recovered in the phylogeny and black points represent the overall known distribution of the species complex. (3) Table of genetic distances (when sufficient data are available), reporting intra- and inter-lineage divergence among delimited groups. (4) Histogram of genetic distances illustrating the barcode gap. (5) Phylogenetic hypotheses from previous studies (when available), provided for comparison with our proposed delimitations. (6) Additional lines of evidence (occasionally available), such as geometric morphometrics or other independent datasets supporting lineage differentiation. Red star indicates holotype.

Note: *When this symbol (⚠) appears next to the phylogeny, it indicates that the delimitation should be interpreted with caution, as additional sampling is needed. When it appears next to the table, it means that genetic distances are low; therefore, we recommend that these results also be considered with caution. In both cases, we have retained the delimitations as inferred by our methods.*

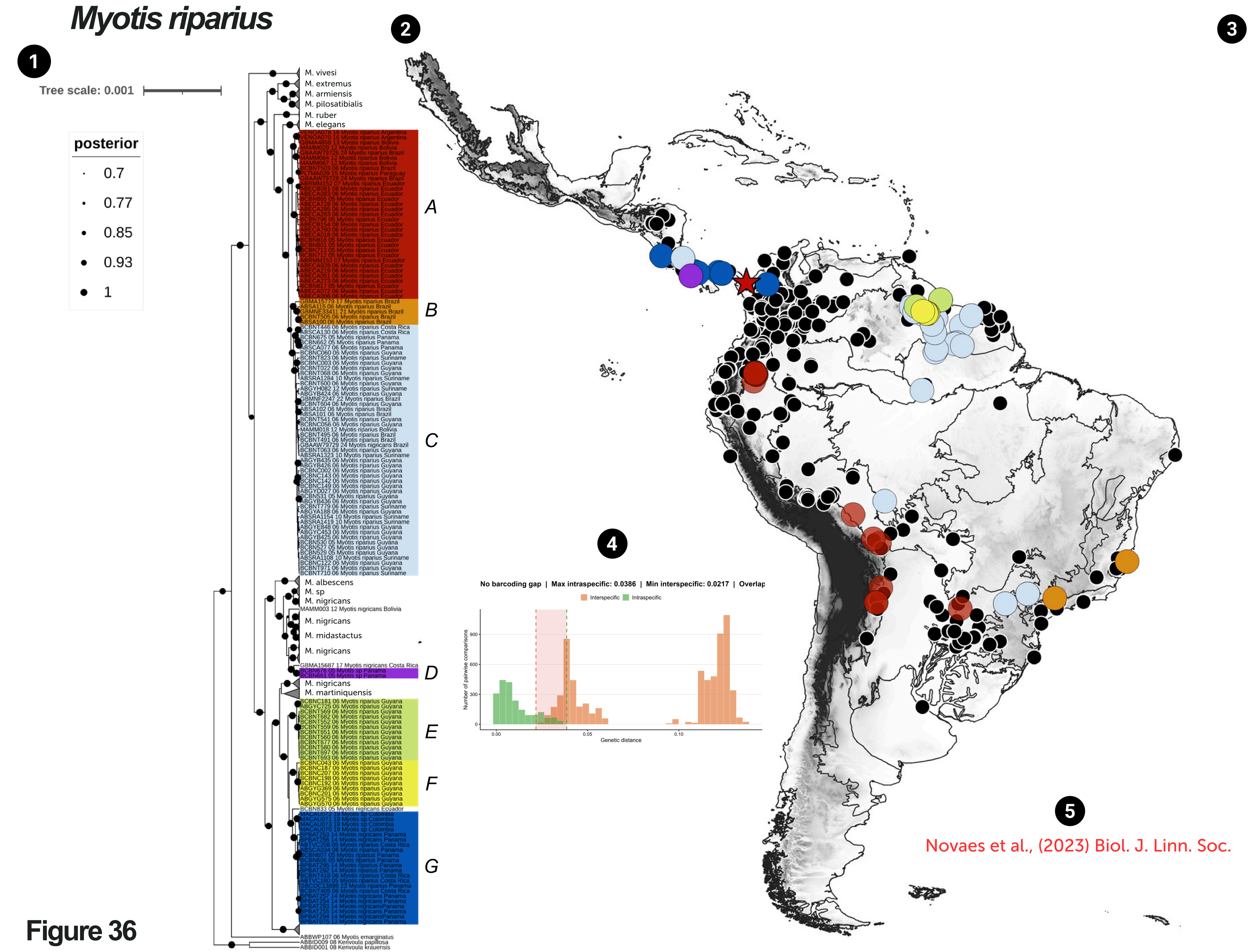

3

Mean genetic distances (Simple Distance)

Diagonal (green) = mean intraspecific distance | Off-diagonal (yellow) = mean interspecific distance | n = number of unique pairs

Especie	*M. riparius* A	*M. riparius* B	*M. riparius* C	*M. riparius* D	*M. riparius* E	*M. riparius* F	*M. riparius* G
M. riparius A	0.0164 (n=528)	0.0439 (n=165)	0.0401 (n=1617)	0.1283 (n=66)	0.1172 (n=396)	0.1240 (n=297)	0.1208 (n=726)
M. riparius B	0.0439 (n=165)	0.0091 (n=10)	0.0325 (n=245)	0.1252 (n=10)	0.1163 (n=60)	0.1266 (n=45)	0.1274 (n=110)
M. riparius C	0.0401 (n=1617)	0.0325 (n=245)	0.0093 (n=1176)	0.1216 (n=98)	0.1141 (n=588)	0.1238 (n=441)	0.1273 (n=1078)
M. riparius D	0.1283 (n=66)	0.1252 (n=10)	0.1216 (n=98)	0.0000 (n=1)	0.1059 (n=24)	0.0964 (n=18)	0.0971 (n=44)
M. riparius E	0.1172 (n=396)	0.1163 (n=60)	0.1141 (n=588)	0.1059 (n=24)	0.0030 (n=66)	0.0419 (n=108)	0.0565 (n=264)
M. riparius F	0.1240 (n=297)	0.1266 (n=45)	0.1238 (n=441)	0.0964 (n=18)	0.0419 (n=108)	0.0014 (n=36)	0.0395 (n=198)
M. riparius G	0.1208 (n=726)	0.1274 (n=110)	0.1273 (n=1078)	0.0971 (n=44)	0.0565 (n=264)	0.0395 (n=198)	0.0057 (n=231)

Figure 36

Fig. 36. Phylogenetic, geographic, and supporting evidence for candidate lineages in *Myotis riparius*. (1) Phylogenetic hypothesis based on COI sequences, with colored clades representing candidate lineages identified through molecular species delimitation. (2) Geographic distribution map, where colored points correspond to the lineages recovered in the phylogeny and black points represent the overall known distribution of the species complex. (3) Table of genetic distances (when sufficient data are available), reporting intra- and inter-lineage divergence among delimited groups. (4) Histogram of genetic distances illustrating the barcode gap. (5) Phylogenetic hypotheses from previous studies (when available), provided for comparison with our proposed delimitations. (6) Additional lines of evidence (occasionally available), such as geometric morphometrics or other independent datasets supporting lineage differentiation. Red star indicates holotype.

Note: *When this symbol (⚠) appears next to the phylogeny, it indicates that the delimitation should be interpreted with caution, as additional sampling is needed. When it appears next to the table, it means that genetic distances are low; therefore, we recommend that these results also be considered with caution. In both cases, we have retained the delimitations as inferred by our methods.*

Neoeptesicus brasiliensis

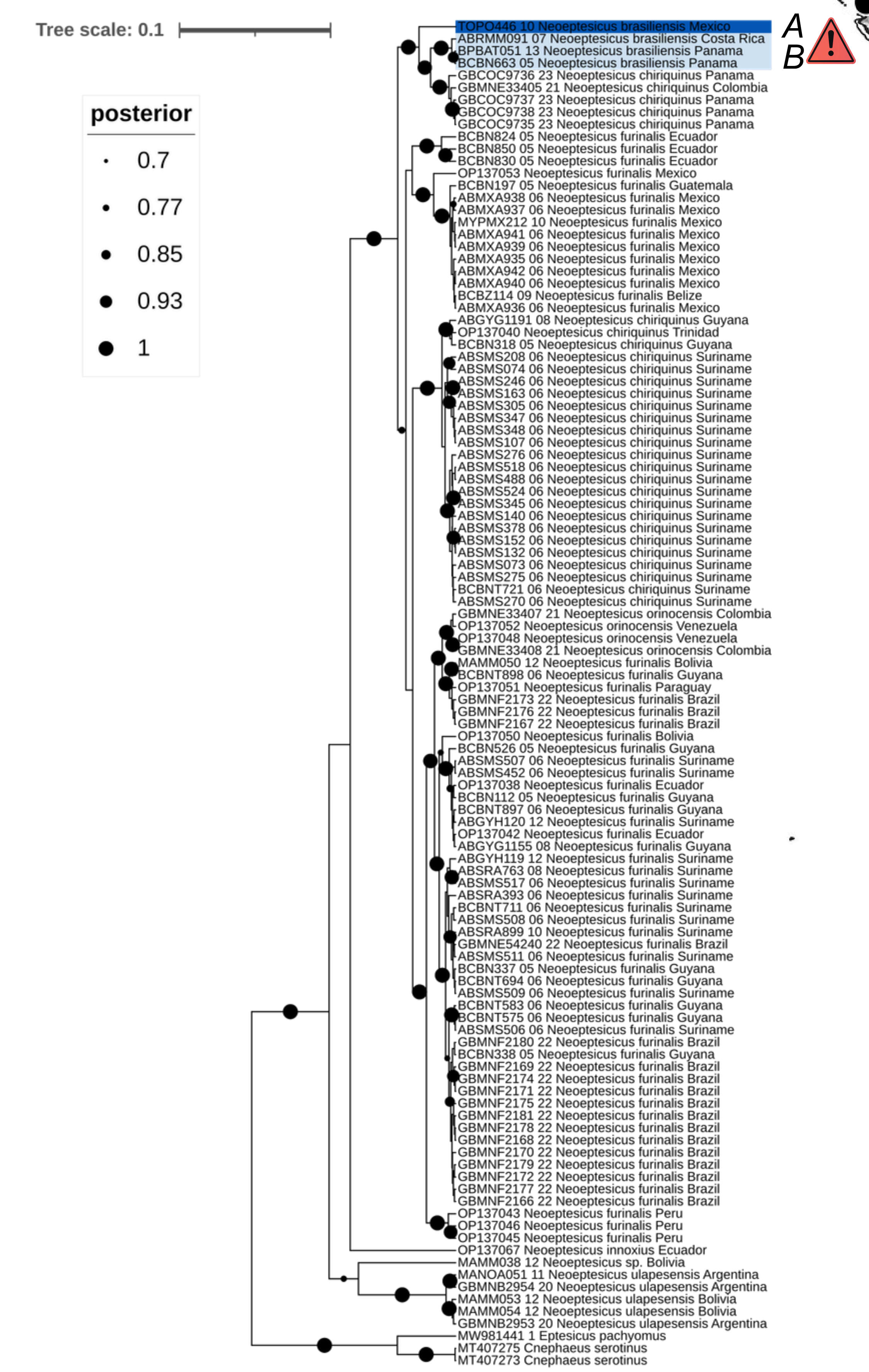

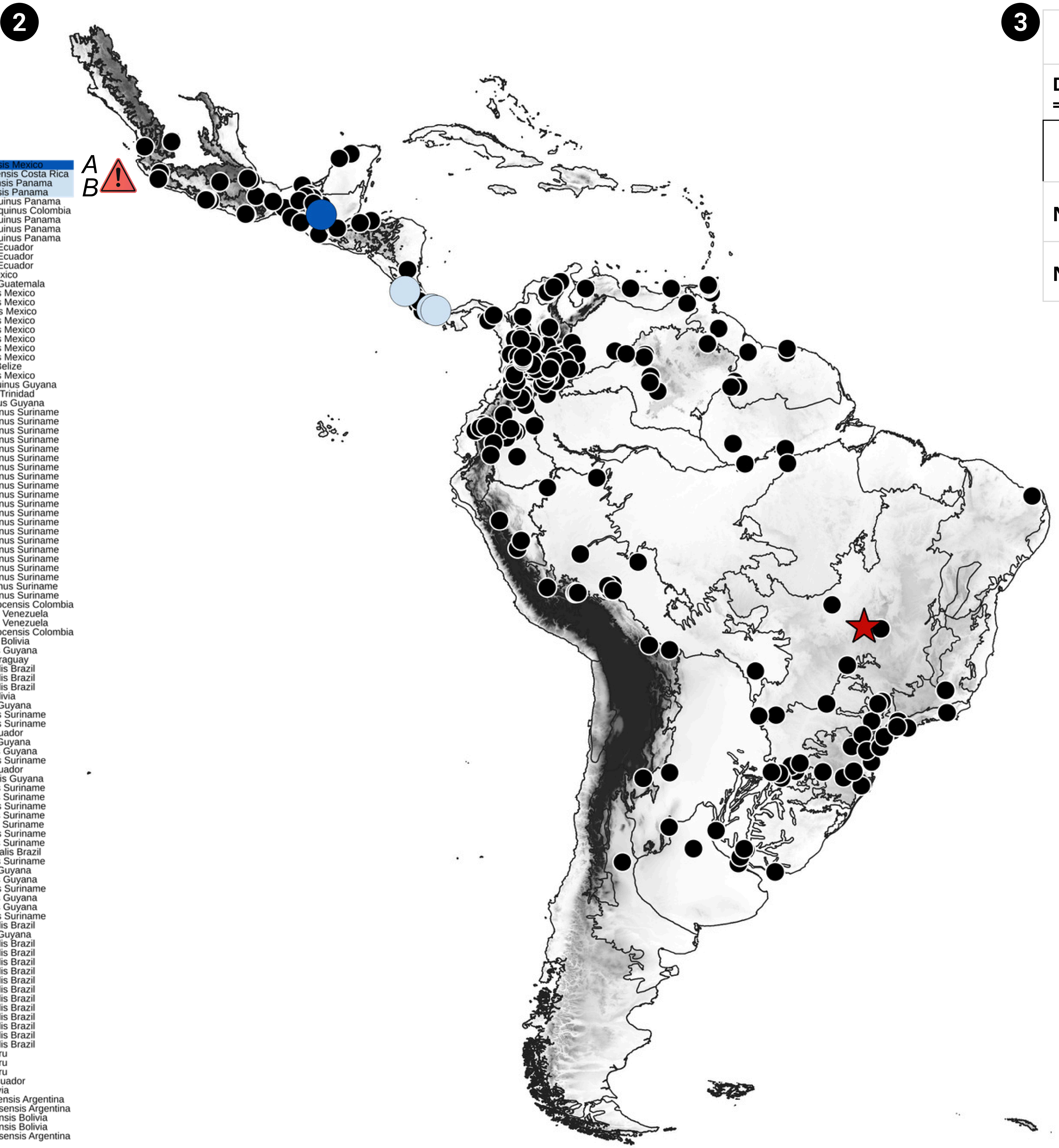

3

Mean genetic distances (Simple Distance)		
Diagonal (red) = mean intraspecific distance | Off-diagonal (black) = mean interspecific distance | n = number of unique pairs		
Especie	N. *brasiliensis* A	*N. brasiliensis B*
N. *brasiliensis* A	--	0.0399 (n=3)
N. *brasiliensis* B	0.0399 (n=3)	0.0031 (n=3)

Figure 37

Fig. 37. Phylogenetic, geographic, and supporting evidence for candidate lineages in *Neoeptesicus brasiliensis.* (1) Phylogenetic hypothesis based on COI sequences, with colored clades representing candidate lineages identified through molecular species delimitation. (2) Geographic distribution map, where colored points correspond to the lineages recovered in the phylogeny and black points represent the overall known distribution of the species complex. (3) Table of genetic distances (when sufficient data are available), reporting intra- and inter-lineage divergence among delimited groups. (4) Histogram of genetic distances illustrating the barcode gap. (5) Phylogenetic hypotheses from previous studies (when available), provided for comparison with our proposed delimitations. (6) Additional lines of evidence (occasionally available), such as geometric morphometrics or other independent datasets supporting lineage differentiation. Red star indicates holotype.

Note: *When this symbol (⚠) appears next to the phylogeny, it indicates that the delimitation should be interpreted with caution, as additional sampling is needed. When it appears next to the table, it means that genetic distances are low; therefore, we recommend that these results also be considered with caution. In both cases, we have retained the delimitations as inferred by our methods.*

Neoeptesicus chiriquinus

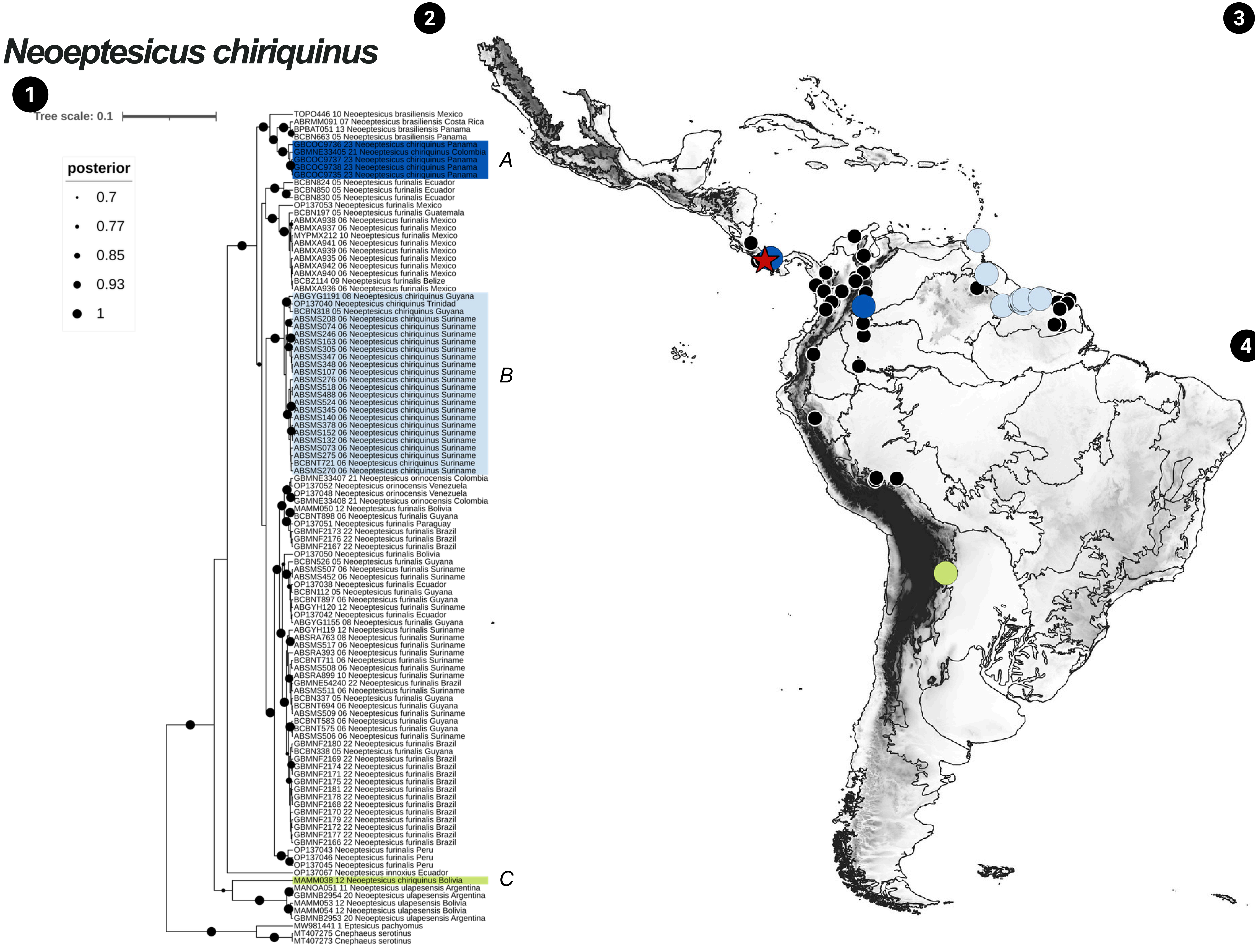

3

Mean genetic distances (Simple Distance)

Diagonal (red) = mean intraspecific distance | Off-diagonal (black) = mean interspecific distance | n = number of unique pairs

Especie	*N. chiriquinus* A	*N. chiriquinus* B	*N. chiriquinus* C
N. chiriquinus A	0.0047 (n=10)	0.0557 (n=120)	0.1102 (n=5)
N. chiriquinus B	0.0557 (n=120)	0.0073 (n=276)	0.1097 (n=24)
N. chiriquinus C	0.1102 (n=5)	0.1097 (n=24)	--

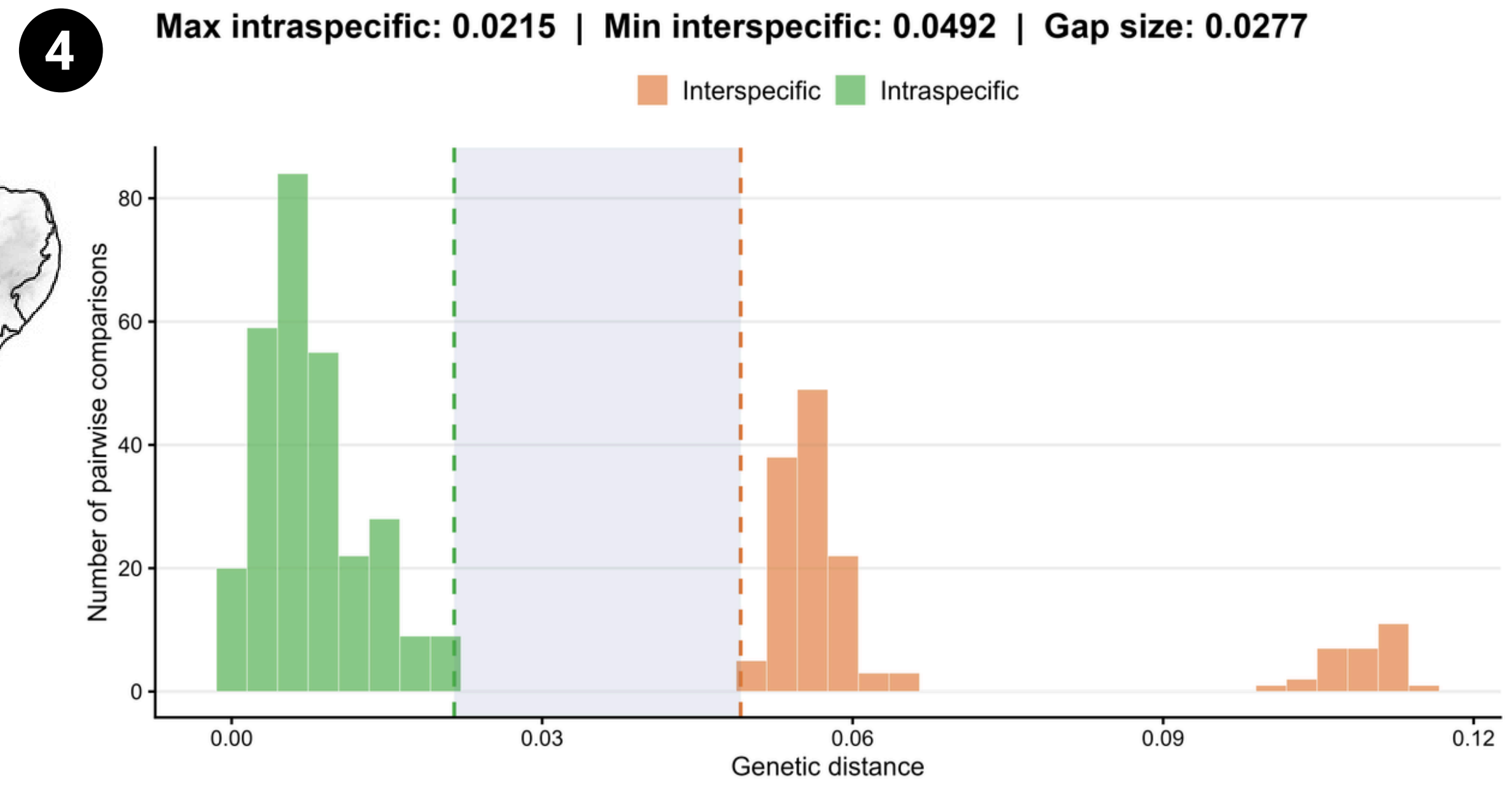

Figure 38

Fig. 38. Phylogenetic, geographic, and supporting evidence for candidate lineages in *Neoeptesicus chiriquinus*. (1) Phylogenetic hypothesis based on COI sequences, with colored clades representing candidate lineages identified through molecular species delimitation. (2) Geographic distribution map, where colored points correspond to the lineages recovered in the phylogeny and black points represent the overall known distribution of the species complex. (3) Table of genetic distances (when sufficient data are available), reporting intra- and inter-lineage divergence among delimited groups. (4) Histogram of genetic distances illustrating the barcode gap. (5) Phylogenetic hypotheses from previous studies (when available), provided for comparison with our proposed delimitations. (6) Additional lines of evidence (occasionally available), such as geometric morphometrics or other independent datasets supporting lineage differentiation. Red star indicates holotype.

Note: *When this symbol (⚠) appears next to the phylogeny, it indicates that the delimitation should be interpreted with caution, as additional sampling is needed. When it appears next to the table, it means that genetic distances are low; therefore, we recommend that these results also be considered with caution. In both cases, we have retained the delimitations as inferred by our methods.*

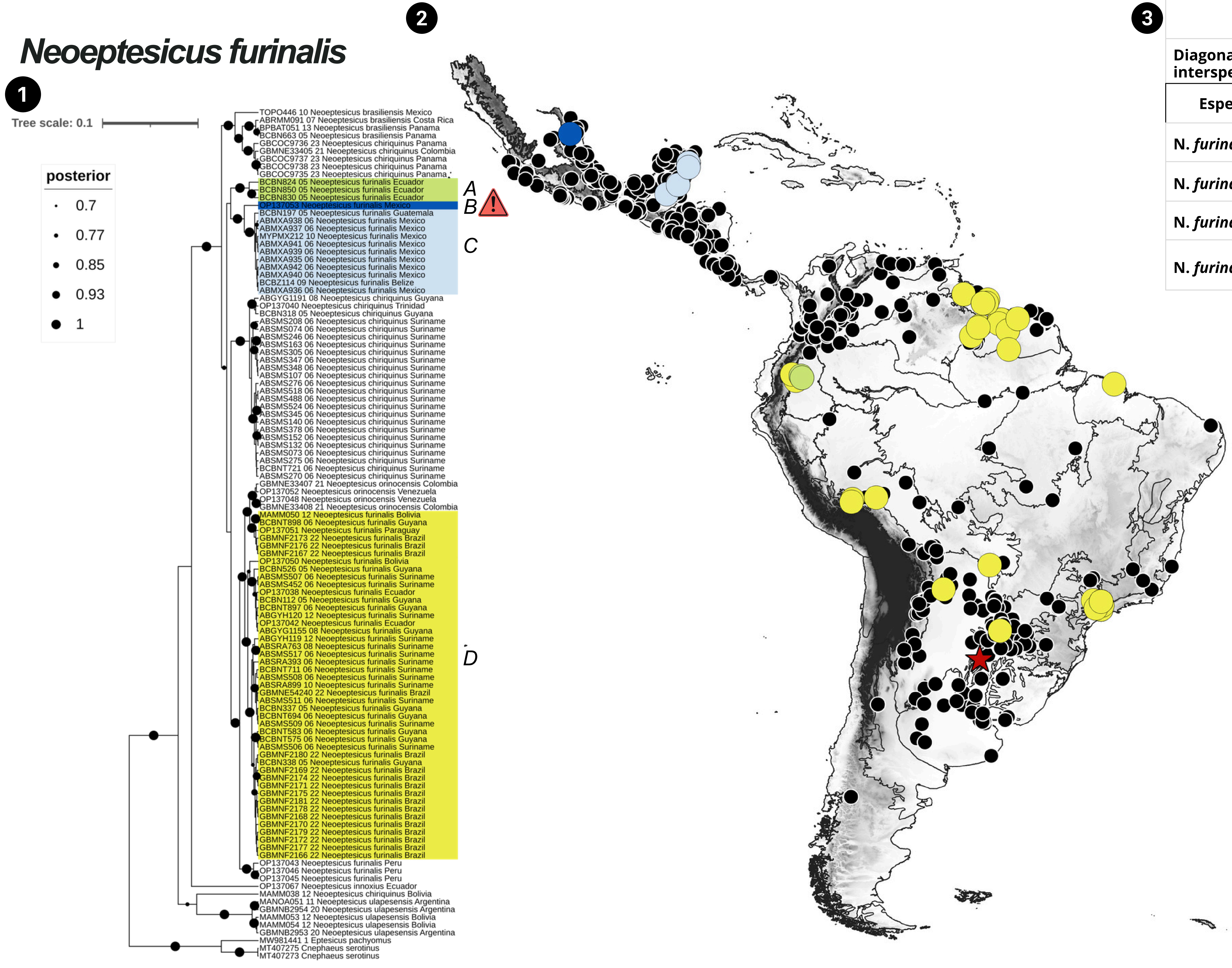

3

Mean genetic distances (Simple Distance)

Diagonal (red) = mean intraspecific distance | Off-diagonal (black) = mean interspecific distance | n = number of unique pairs

Especie	N. *furinalis* A	N. *furinalis* B	N. *furinalis* C	N. *furinalis* D
N. *furinalis* A	0.0133 (n=3)	0.0517 (n=3)	0.0559 (n=33)	0.0411 (n=135)
N. *furinalis* B	0.0517 (n=3)	--	0.0297 (n=11)	0.0580 (n=45)
N. *furinalis* C	0.0559 (n=33)	0.0297 (n=11)	0.0019 (n=55)	0.0570 (n=495)
N. *furinalis* D	0.0411 (n=135)	0.0580 (n=45)	0.0570 (n=495)	0.0118 (n=990)

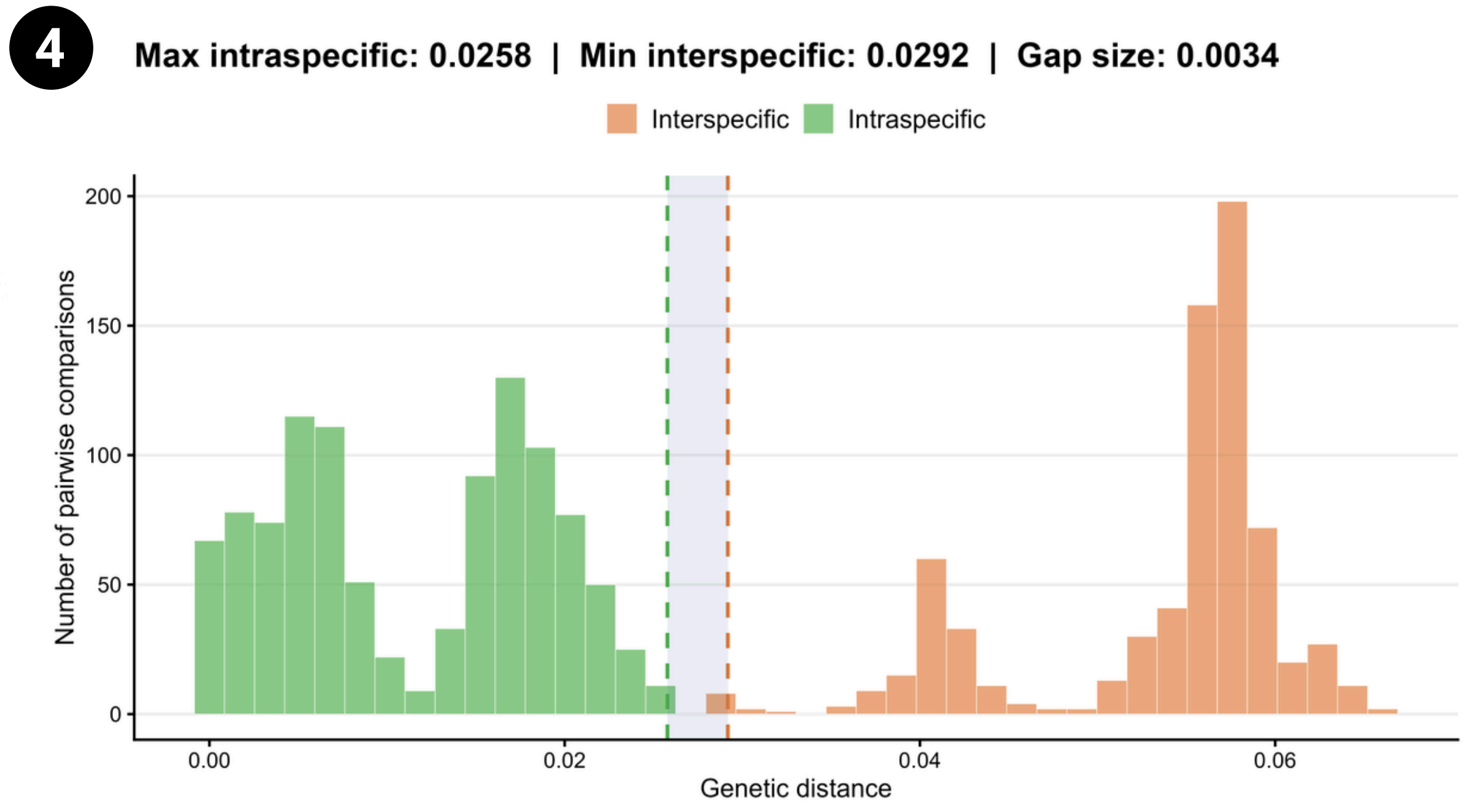

Figure 39

Fig. 39. Phylogenetic, geographic, and supporting evidence for candidate lineages in *Neoeptesicus furinalis.* (1) Phylogenetic hypothesis based on COI sequences, with colored clades representing candidate lineages identified through molecular species delimitation. (2) Geographic distribution map, where colored points correspond to the lineages recovered in the phylogeny and black points represent the overall known distribution of the species complex. (3) Table of genetic distances (when sufficient data are available), reporting intra- and inter-lineage divergence among delimited groups. (4) Histogram of genetic distances illustrating the barcode gap. (5) Phylogenetic hypotheses from previous studies (when available), provided for comparison with our proposed delimitations. (6) Additional lines of evidence (occasionally available), such as geometric morphometrics or other independent datasets supporting lineage differentiation. Red star indicates holotype.

Note: *When this symbol (⚠) appears next to the phylogeny, it indicates that the delimitation should be interpreted with caution, as additional sampling is needed. When it appears next to the table, it means that genetic distances are low; therefore, we recommend that these results also be considered with caution. In both cases, we have retained the delimitations as inferred by our methods.*

Pteronotus personatus

1

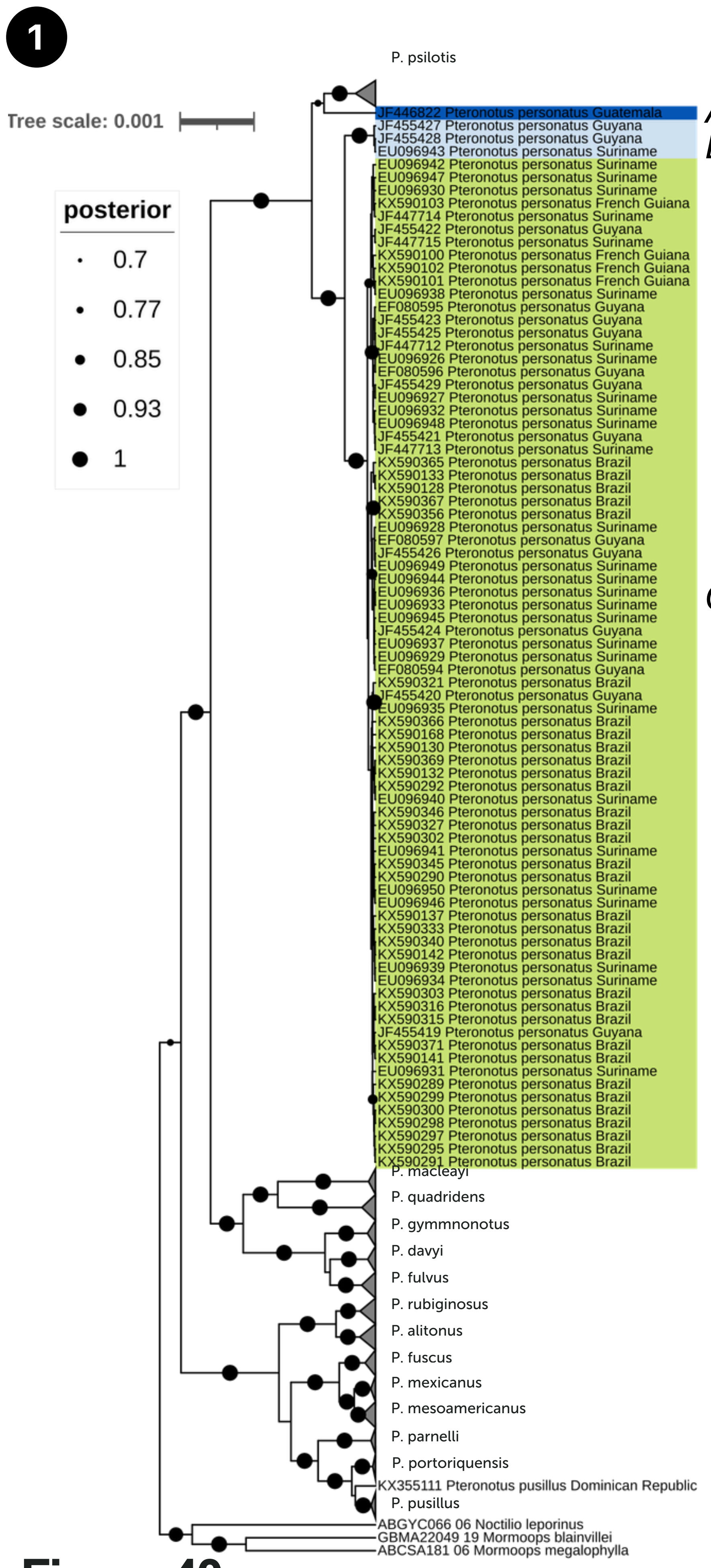

2

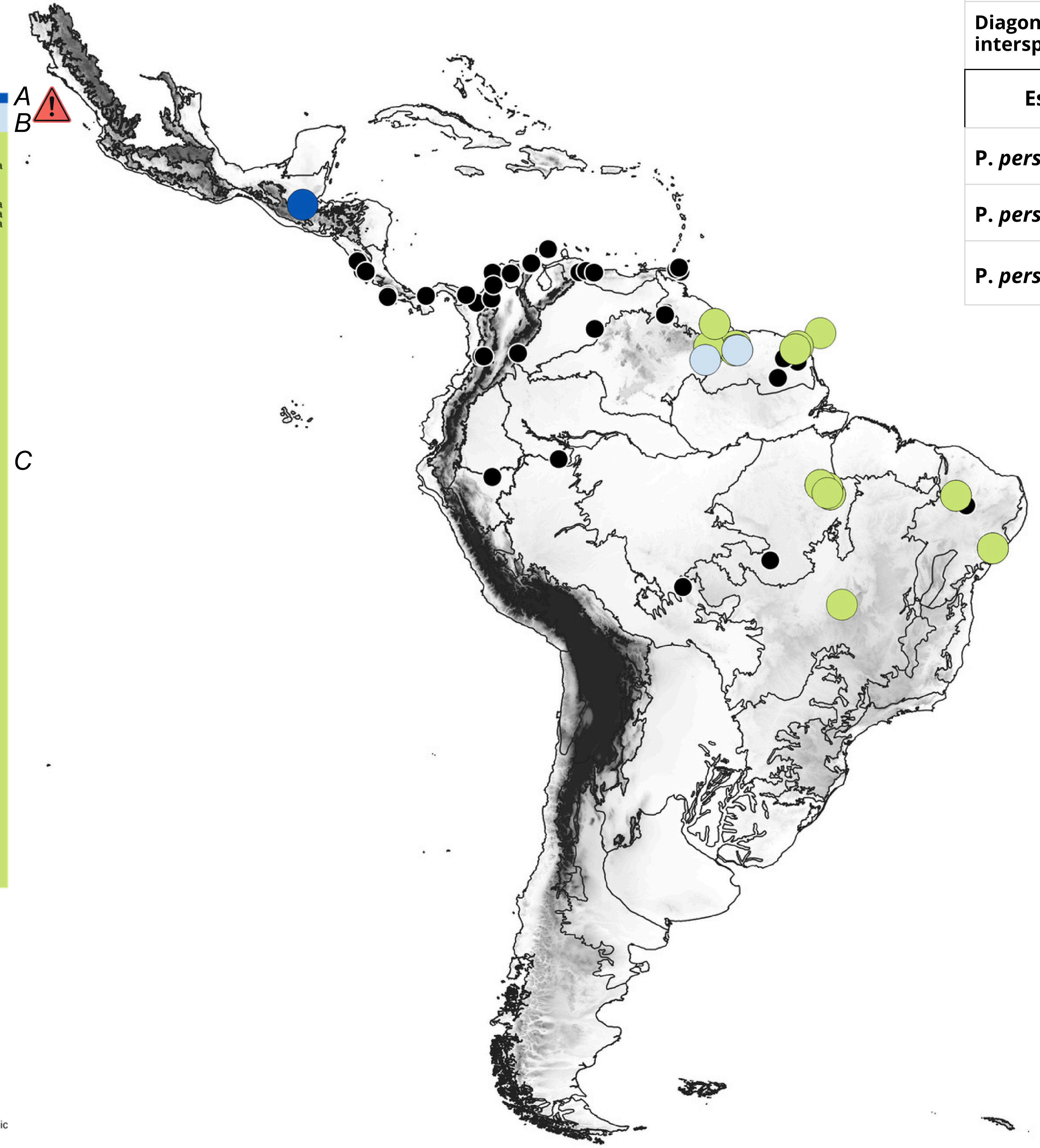

3

Mean genetic distances (Simple Distance)

Diagonal (red) = mean intraspecific distance | Off-diagonal (black) = mean interspecific distance | n = number of unique pairs

Especie	*P. personatus* A	*P. personatus* B	*P. personatus* C
P. personatus A	--	0.0681 (n=3)	0.0832 (n=78)
P. personatus B	0.0681 (n=3)	0.0000 (n=3)	0.0414 (n=234)
P. personatus C	0.0832 (n=78)	0.0414 (n=234)	0.0024 (n=3003)

4 **Max intraspecific: 0.0062 | Min interspecific: 0.0401 | Gap size: 0.0339**

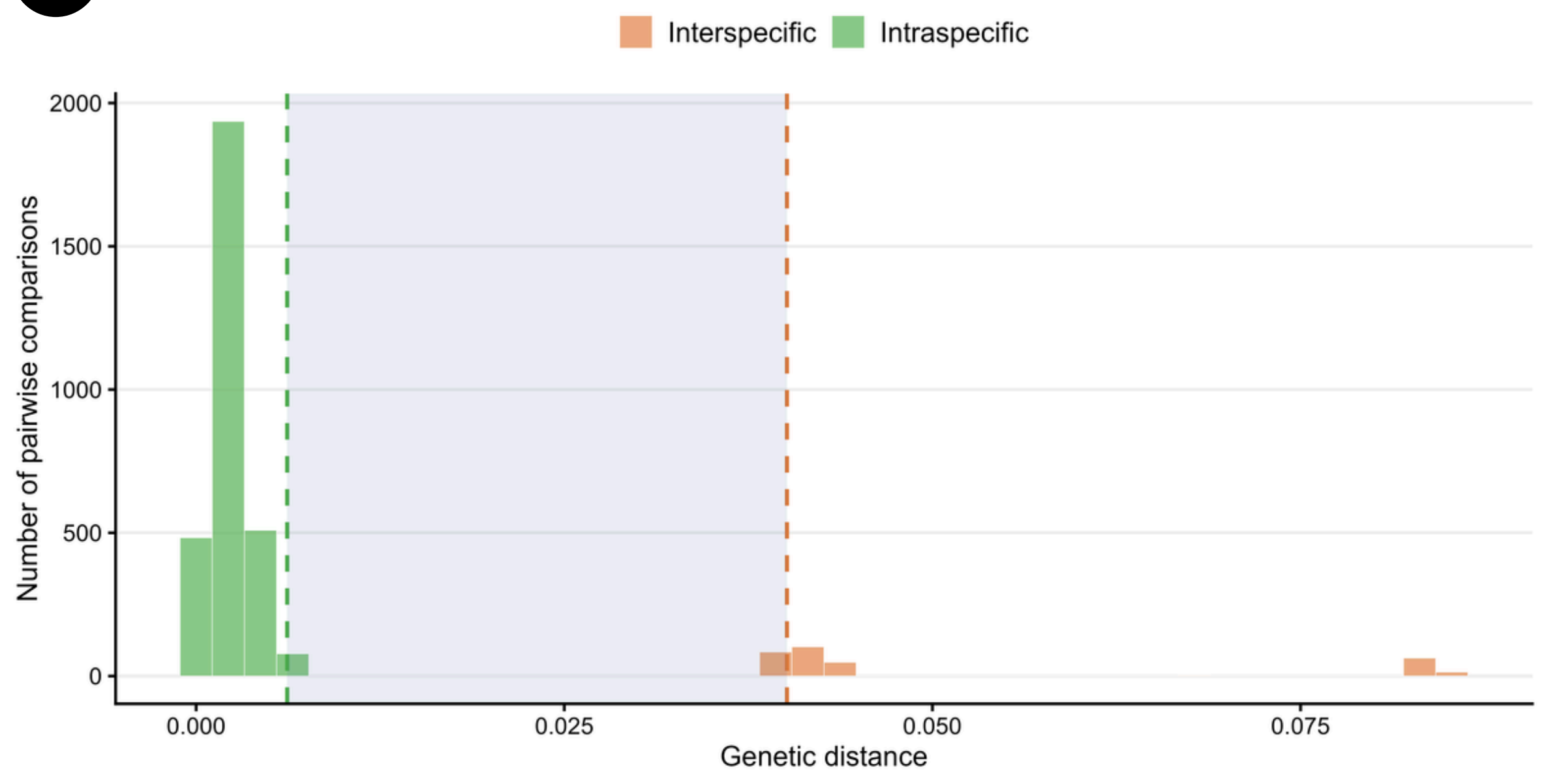

5 Pavan & Marroig (2016) Mol. Phylogenet. Evol.

Figure 40

Fig. 40. Phylogenetic, geographic, and supporting evidence for candidate lineages in *Pteronotus personatus*. (1) Phylogenetic hypothesis based on COI sequences, with colored clades representing candidate lineages identified through molecular species delimitation. (2) Geographic distribution map, where colored points correspond to the lineages recovered in the phylogeny and black points represent the overall known distribution of the species complex. (3) Table of genetic distances (when sufficient data are available), reporting intra- and inter-lineage divergence among delimited groups. (4) Histogram of genetic distances illustrating the barcode gap. (5) Phylogenetic hypotheses from previous studies (when available), provided for comparison with our proposed delimitations. (6) Additional lines of evidence (occasionally available), such as geometric morphometrics or other independent datasets supporting lineage differentiation. Red star indicates holotype.

Note: *When this symbol (⚠) appears next to the phylogeny, it indicates that the delimitation should be interpreted with caution, as additional sampling is needed. When it appears next to the table, it means that genetic distances are low; therefore, we recommend that these results also be considered with caution. In both cases, we have retained the delimitations as inferred by our methods.*

Pteronotus pusillus

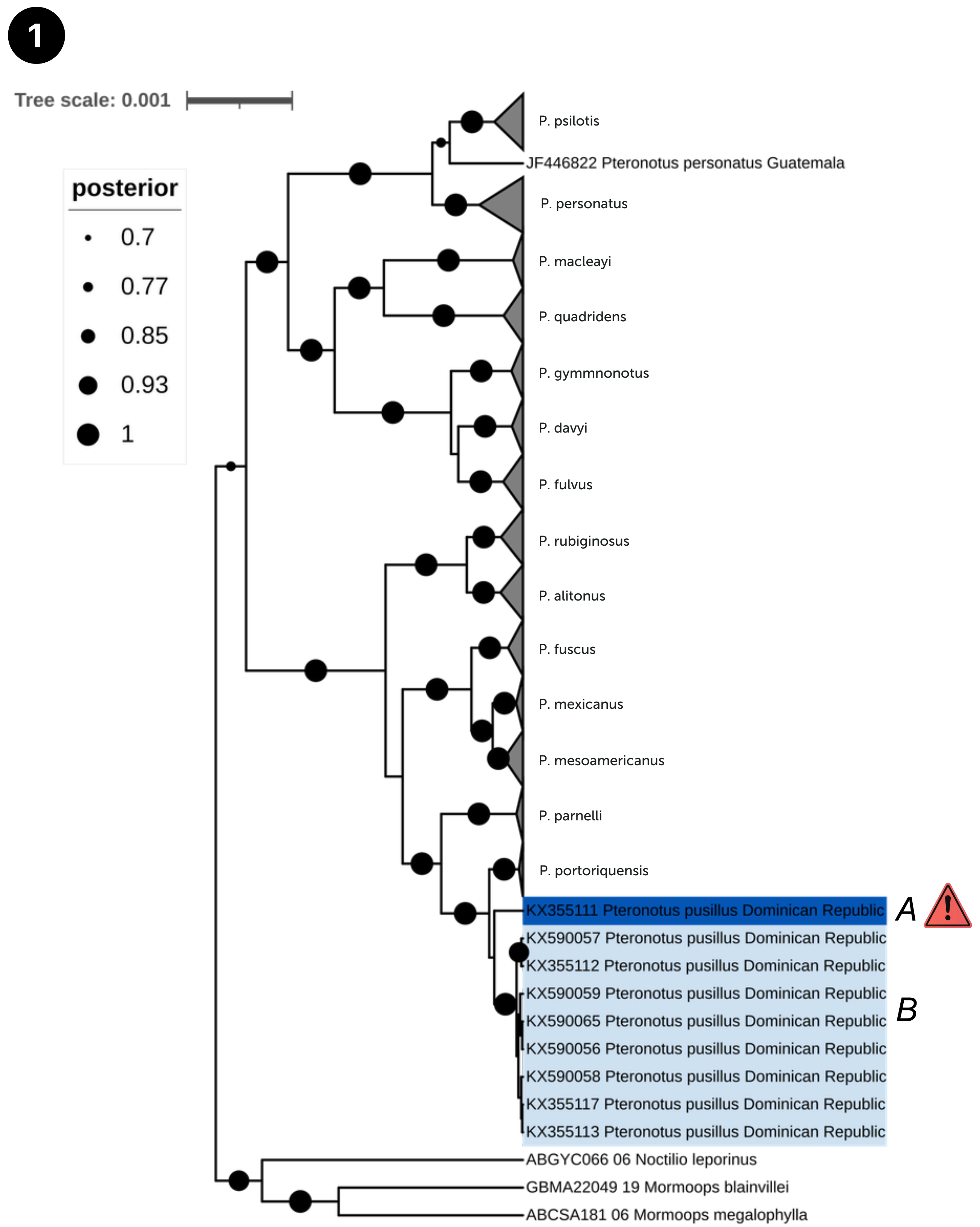

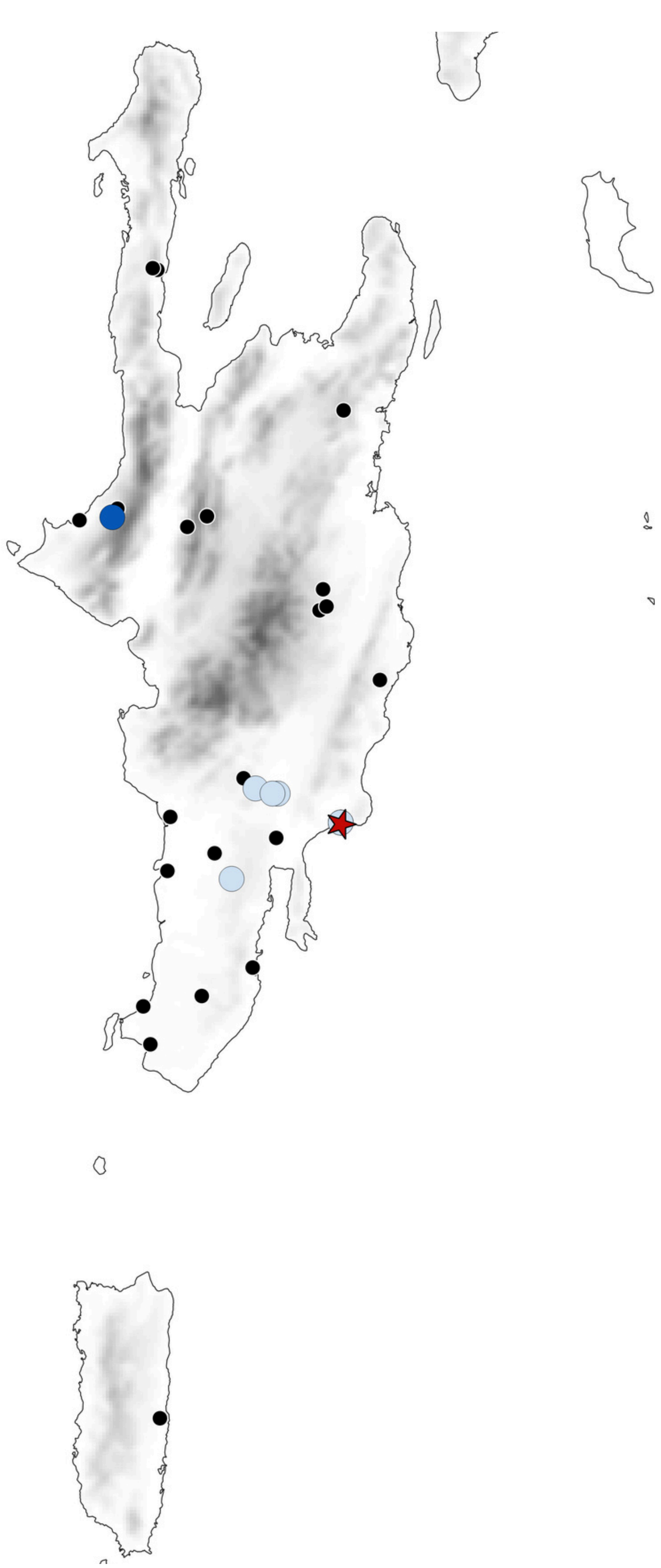

3

Mean genetic distances (Simple Distance)

Diagonal (green) = mean intraspecific distance | Off-diagonal (yellow) = mean interspecific distance | n = number of unique pairs

Especie	*P. pusillus* A	*P. pusillus* B
P. pusillus A	--	0.0334 (n=8)
P. pusillus B	0.0334 (n=8)	0.0018 (n=28)

4

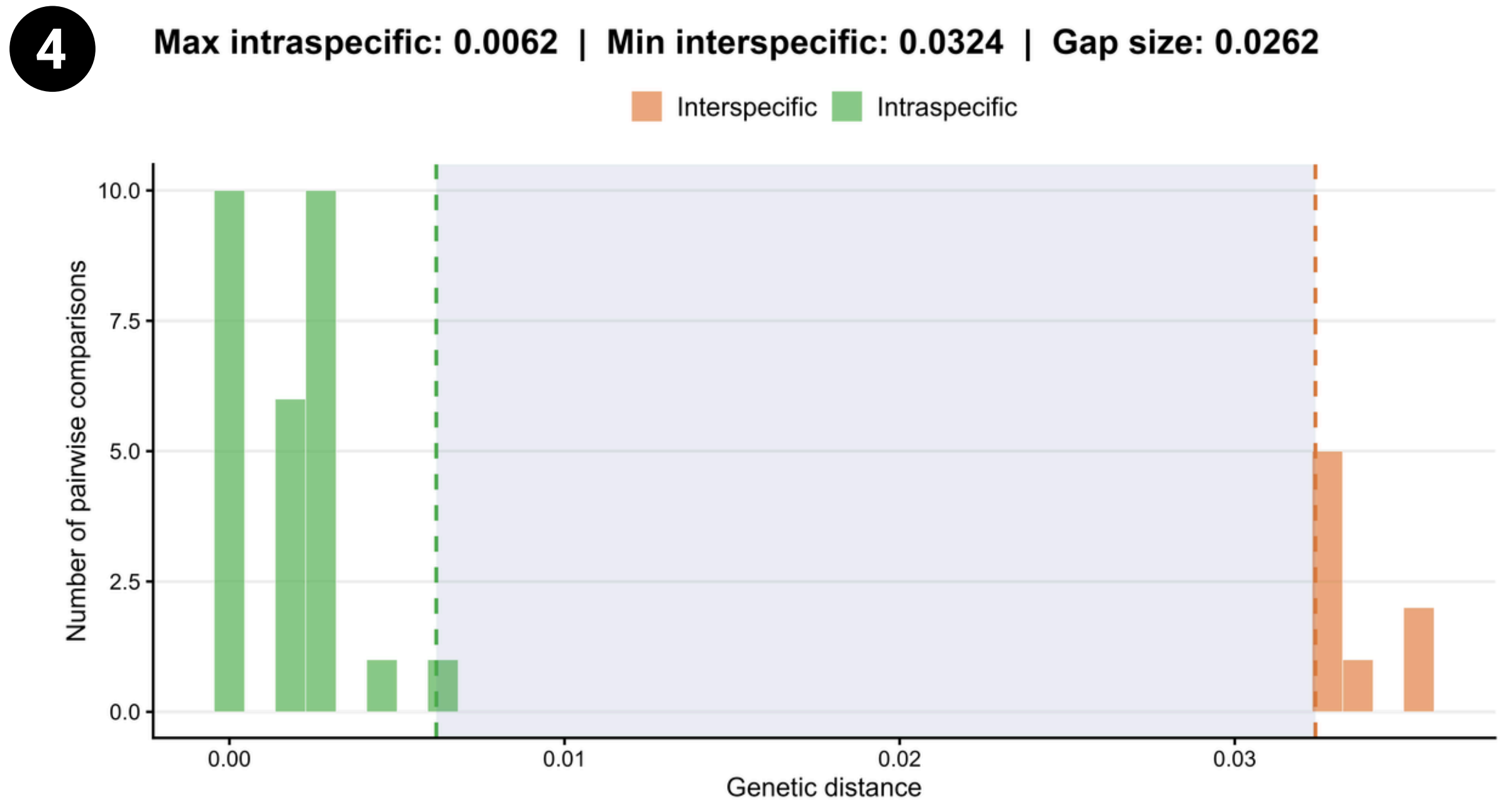

5 Lim et al., (2017). J. Mammal.

Figure 41

Fig. 41. Phylogenetic, geographic, and supporting evidence for candidate lineages in *Pteronotus pusillus.* (1) Phylogenetic hypothesis based on COI sequences, with colored clades representing candidate lineages identified through molecular species delimitation. (2) Geographic distribution map, where colored points correspond to the lineages recovered in the phylogeny and black points represent the overall known distribution of the species complex. (3) Table of genetic distances (when sufficient data are available), reporting intra- and inter-lineage divergence among delimited groups. (4) Histogram of genetic distances illustrating the barcode gap. (5) Phylogenetic hypotheses from previous studies (when available), provided for comparison with our proposed delimitations. (6) Additional lines of evidence (occasionally available), such as geometric morphometrics or other independent datasets supporting lineage differentiation. Red star indicates holotype.

Note: *When this symbol (⚠) appears next to the phylogeny, it indicates that the delimitation should be interpreted with caution, as additional sampling is needed. When it appears next to the table, it means that genetic distances are low; therefore, we recommend that these results also be considered with caution. In both cases, we have retained the delimitations as inferred by our methods.*

Eumops hansae

1

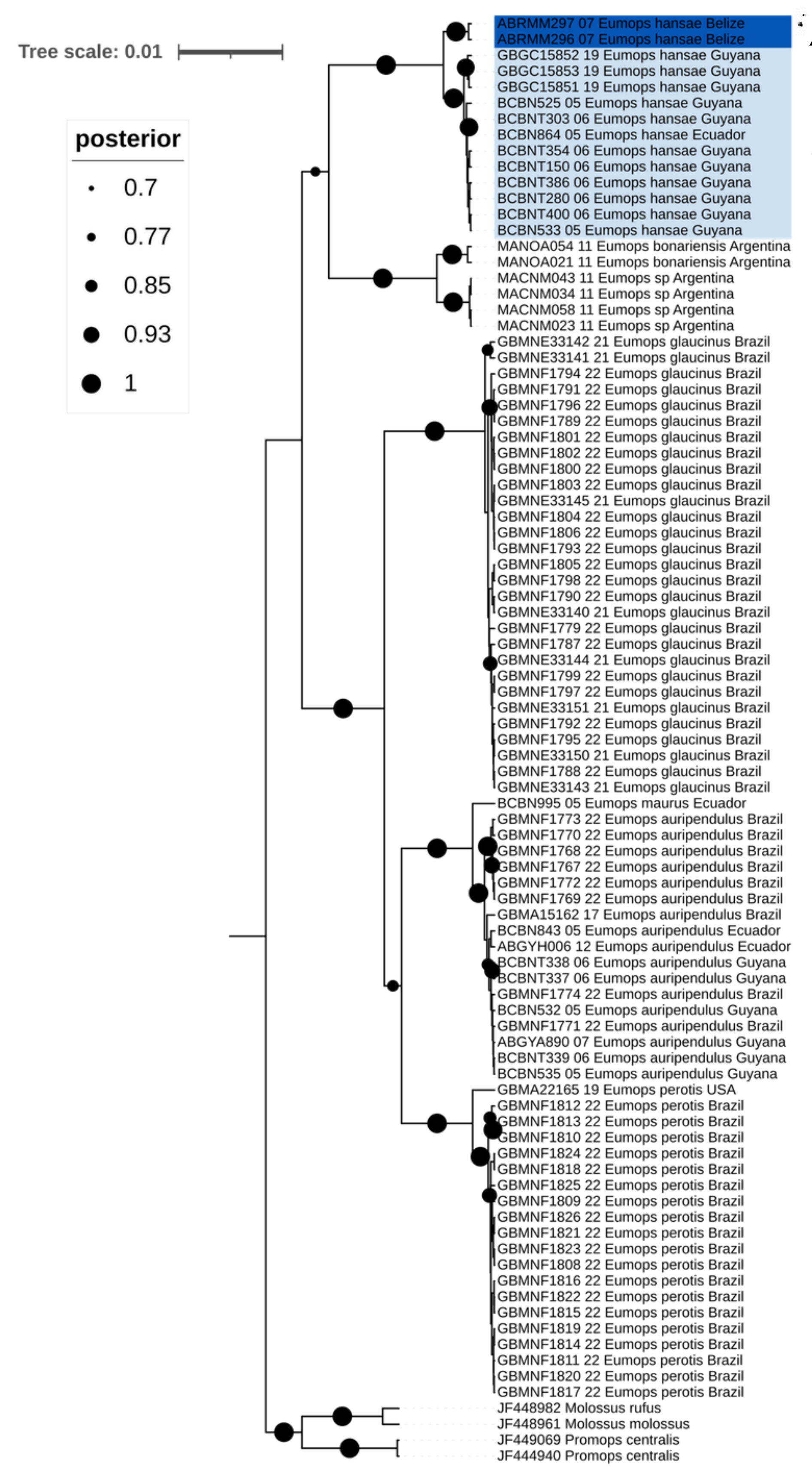

2

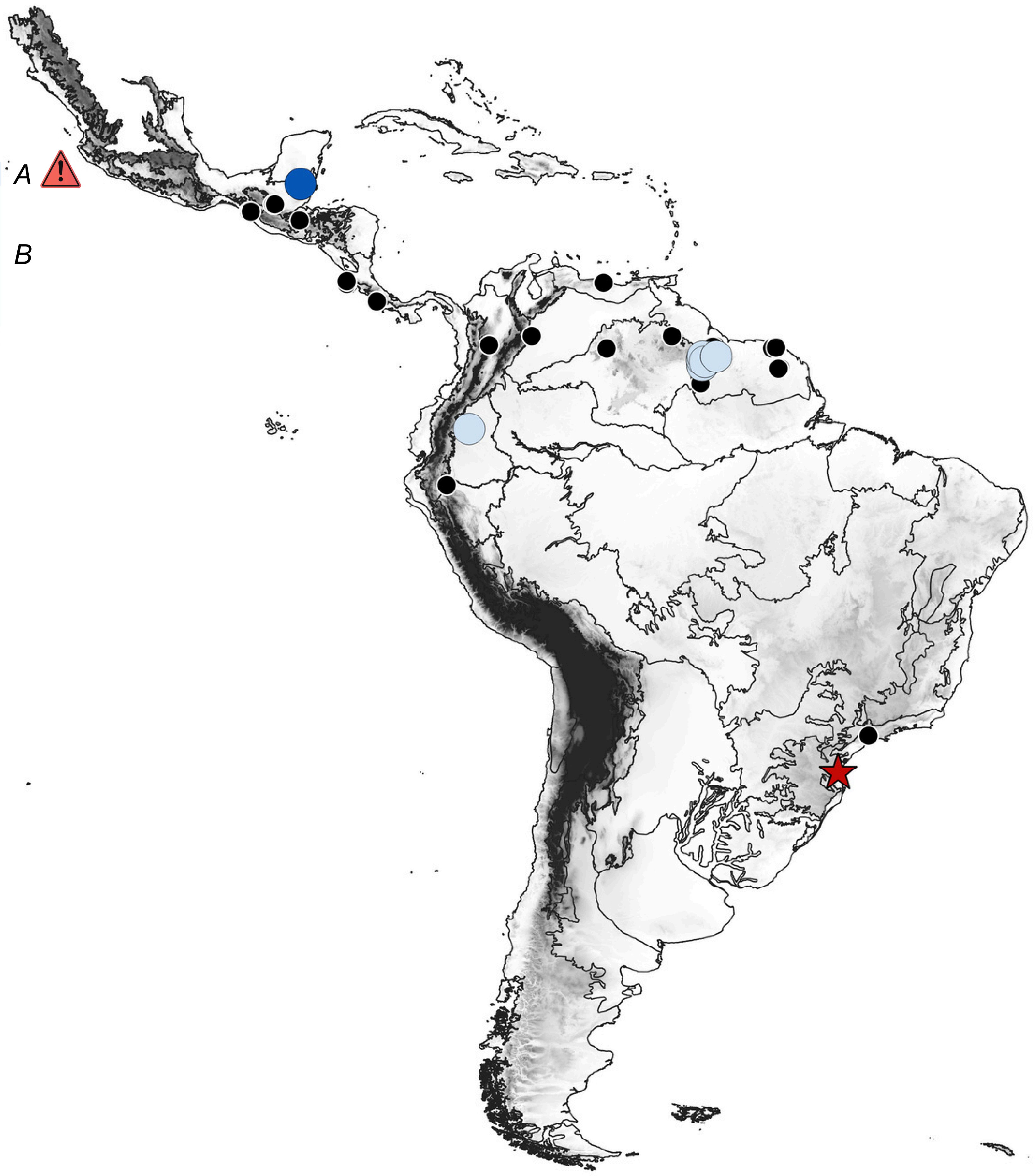

3

Mean genetic distances (Simple Distance)

Diagonal (green) = mean intraspecific distance | Off-diagonal (yellow) = mean interspecific distance | n = number of unique pairs

Especie	E. *hansae* A	E. *hansae* B
E. *hansae* A	0.0033 (n=1)	0.0355 (n=24)
E. *hansae* B	0.0355 (n=24)	0.0039 (n=66)

4

Max intraspecific: 0.0092 | Min interspecific: 0.0329 | Gap size: 0.0237

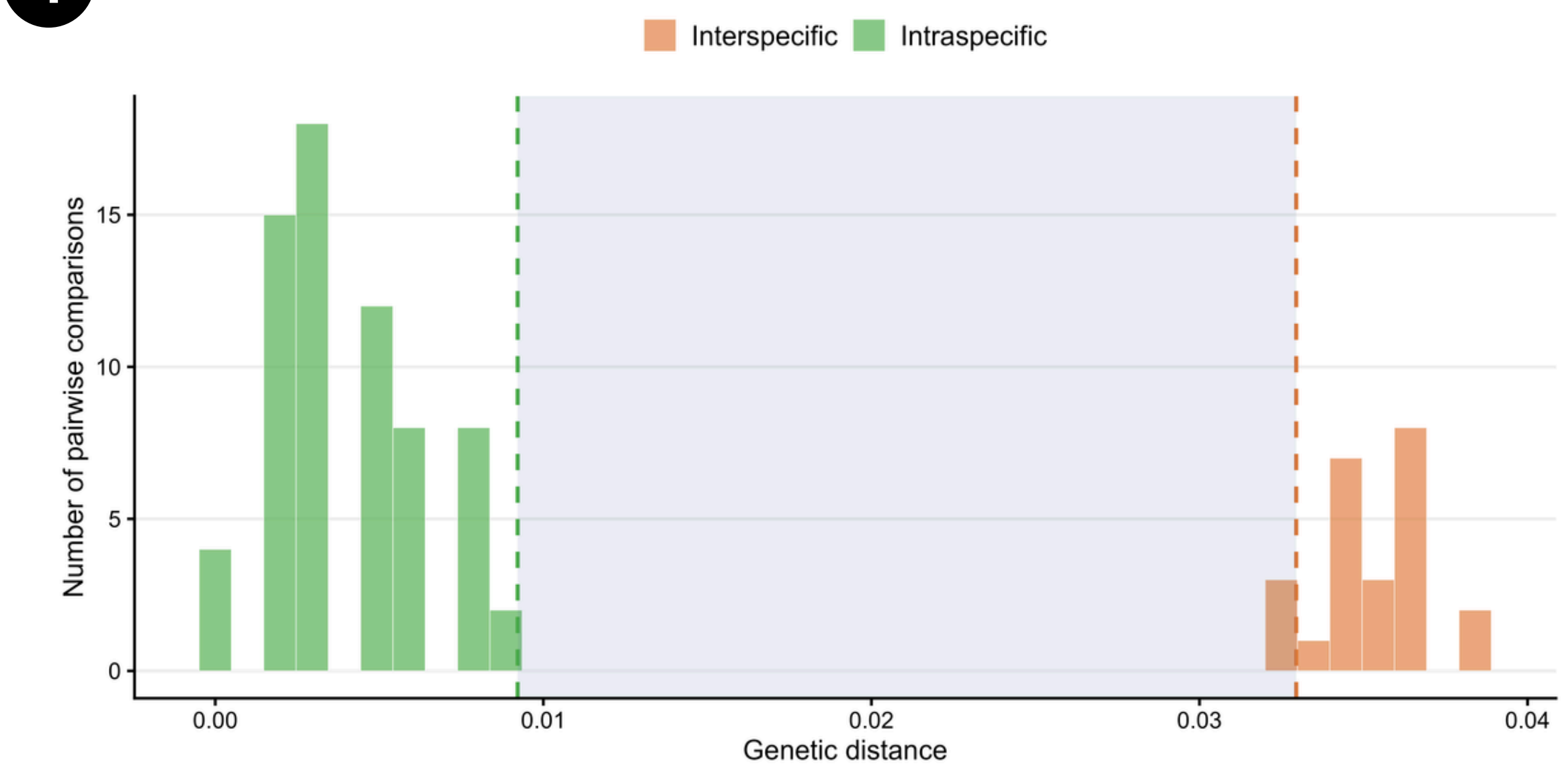

Figure 42

Fig. 42. Phylogenetic, geographic, and supporting evidence for candidate lineages in *Eumops hansae*. (1) Phylogenetic hypothesis based on COI sequences, with colored clades representing candidate lineages identified through molecular species delimitation. (2) Geographic distribution map, where colored points correspond to the lineages recovered in the phylogeny and black points represent the overall known distribution of the species complex. (3) Table of genetic distances (when sufficient data are available), reporting intra- and inter-lineage divergence among delimited groups. (4) Histogram of genetic distances illustrating the barcode gap. (5) Phylogenetic hypotheses from previous studies (when available), provided for comparison with our proposed delimitations. (6) Additional lines of evidence (occasionally available), such as geometric morphometrics or other independent datasets supporting lineage differentiation. Red star indicates holotype.

Note: *When this symbol (⚠) appears next to the phylogeny, it indicates that the delimitation should be interpreted with caution, as additional sampling is needed. When it appears next to the table, it means that genetic distances are low; therefore, we recommend that these results also be considered with caution. In both cases, we have retained the delimitations as inferred by our methods.*

Molossops neglectus

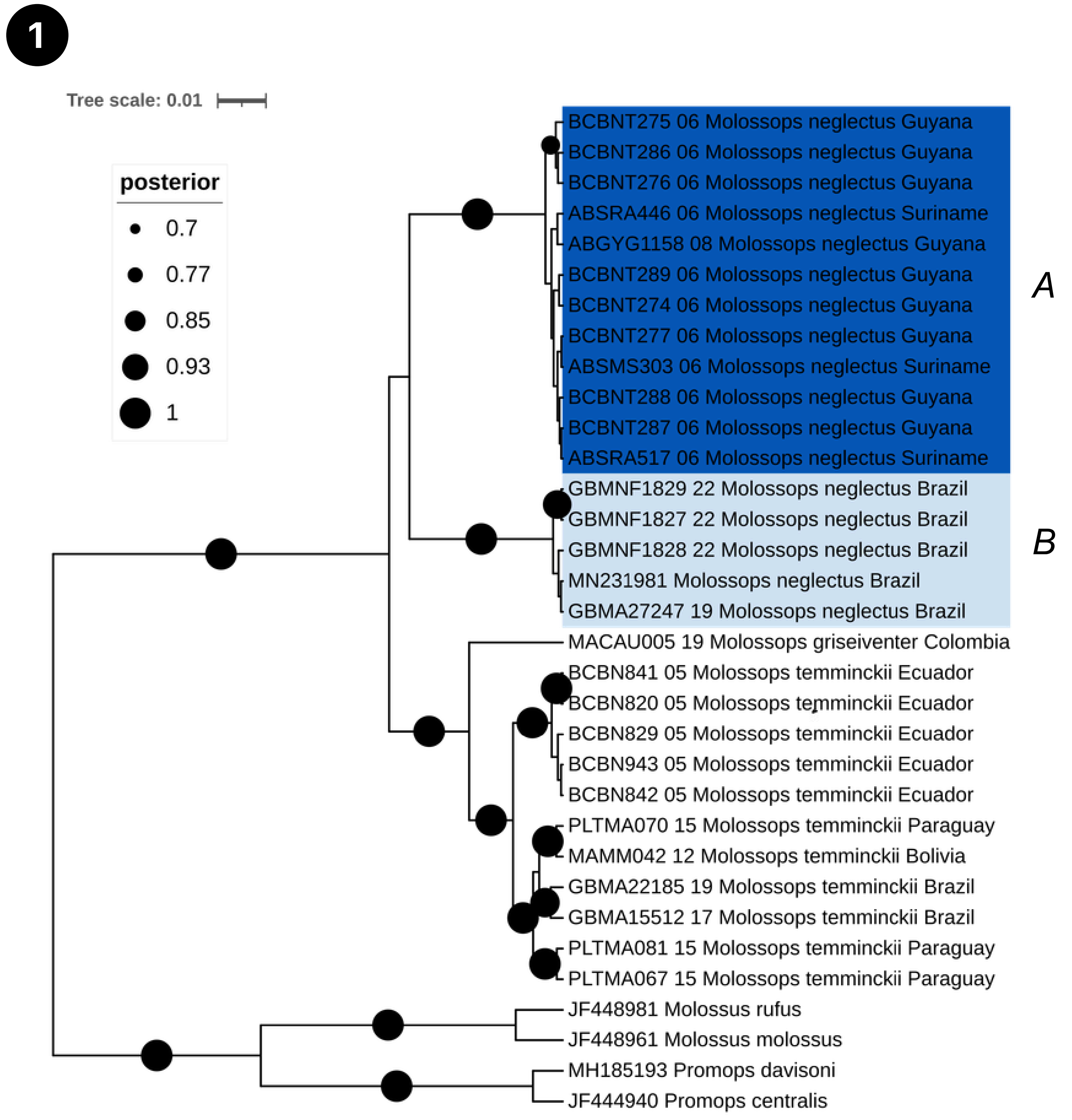

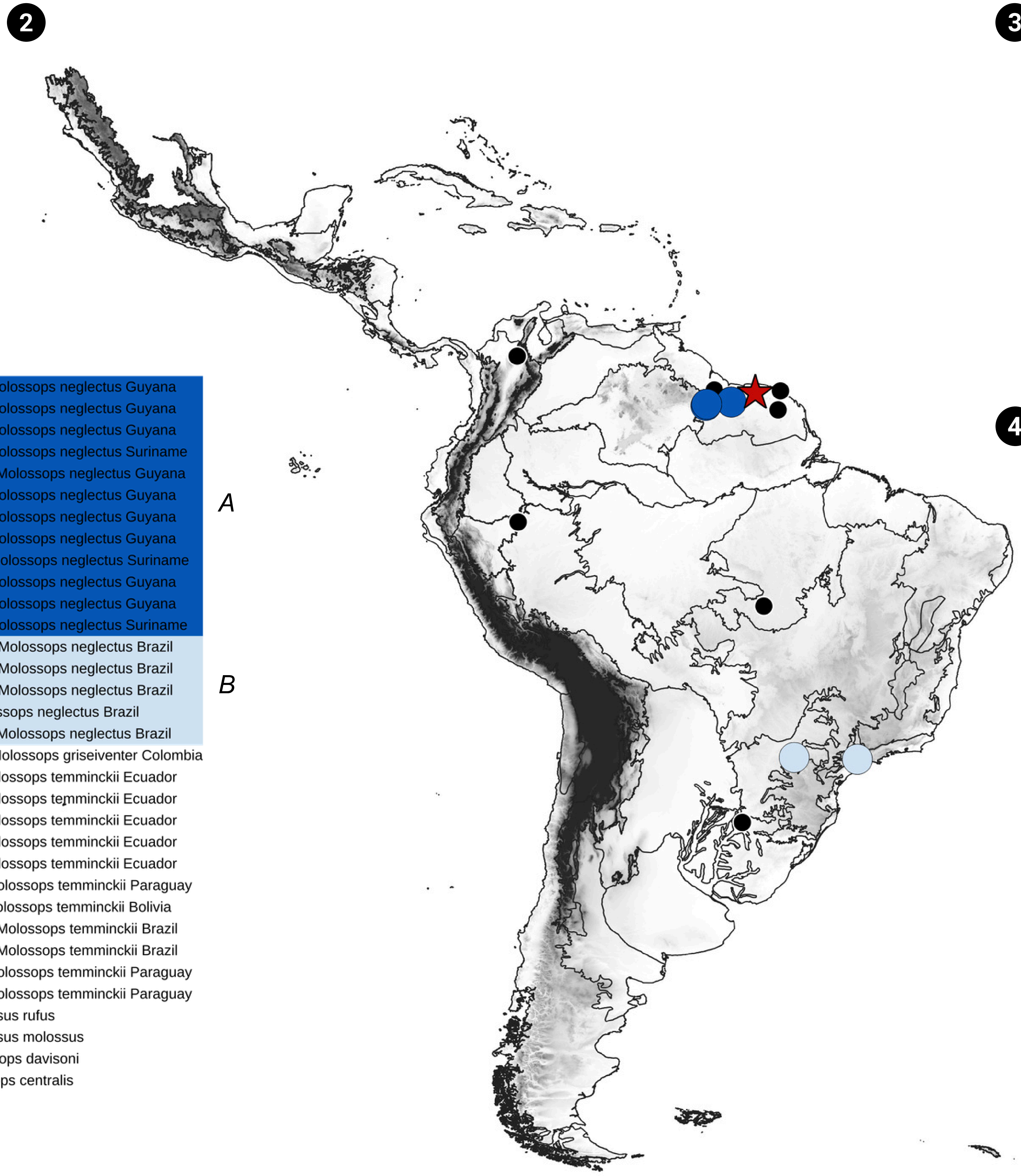

3

Mean genetic distances (Simple Distance)

Diagonal (green) = mean intraspecific distance | Off-diagonal (yellow) = mean interspecific distance | n = number of unique pairs

Especie	M. *neglectus* A	M. *neglectus* B
M. *neglectus* A	0.0019 (n=66)	0.0656 (n=60)
M. *neglectus* B	0.0656 (n=60)	0.0010 (n=10)

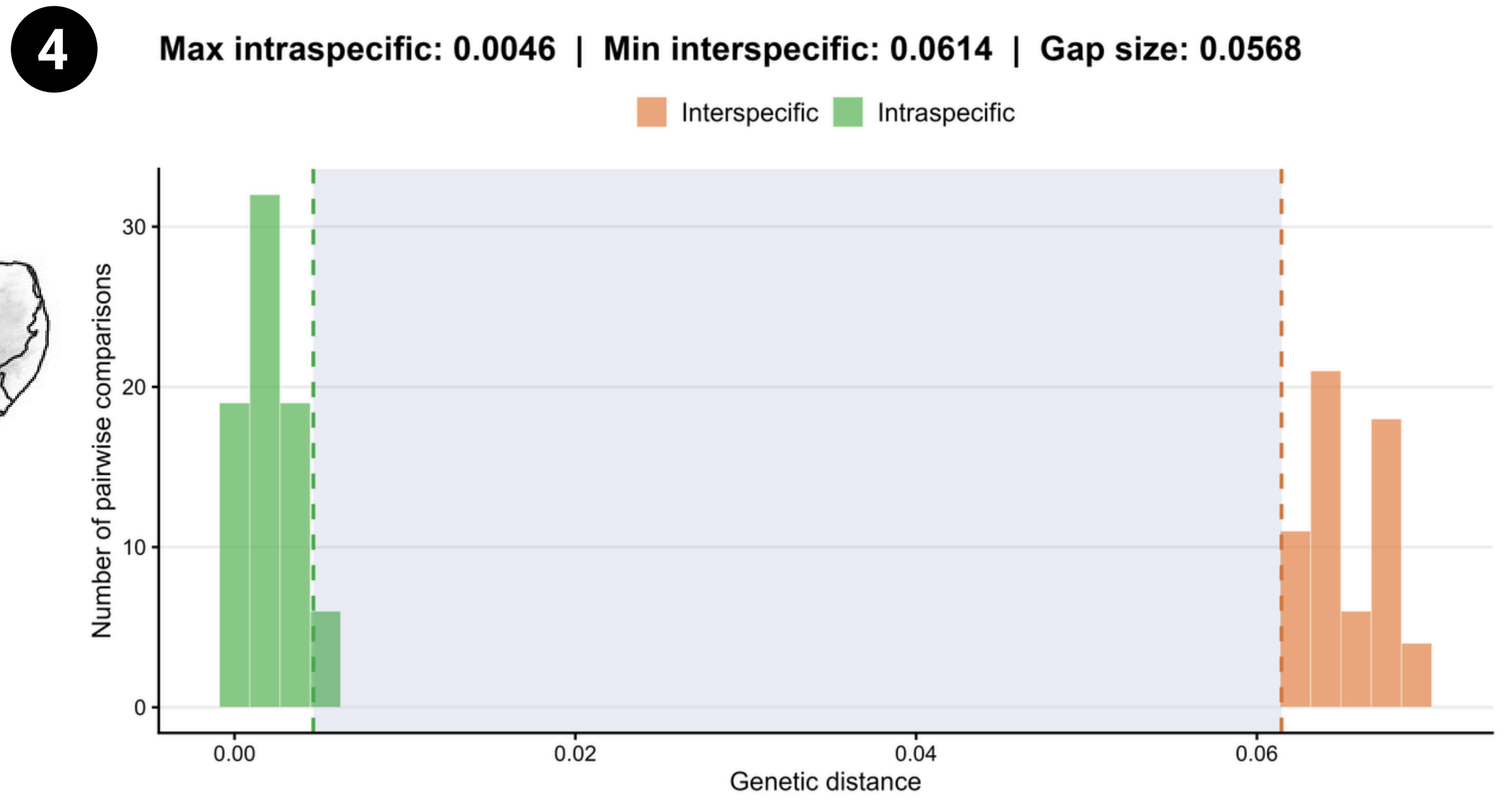

Figure 43

Fig. 43. Phylogenetic, geographic, and supporting evidence for candidate lineages in *Molossops neglectus*. (1) Phylogenetic hypothesis based on COI sequences, with colored clades representing candidate lineages identified through molecular species delimitation. (2) Geographic distribution map, where colored points correspond to the lineages recovered in the phylogeny and black points represent the overall known distribution of the species complex. (3) Table of genetic distances (when sufficient data are available), reporting intra- and inter-lineage divergence among delimited groups. (4) Histogram of genetic distances illustrating the barcode gap. (5) Phylogenetic hypotheses from previous studies (when available), provided for comparison with our proposed delimitations. (6) Additional lines of evidence (occasionally available), such as geometric morphometrics or other independent datasets supporting lineage differentiation. Red star indicates holotype.

Note: *When this symbol (⚠) appears next to the phylogeny, it indicates that the delimitation should be interpreted with caution, as additional sampling is needed. When it appears next to the table, it means that genetic distances are low; therefore, we recommend that these results also be considered with caution. In both cases, we have retained the delimitations as inferred by our methods.*

Nyctinomops laticaudatus

1

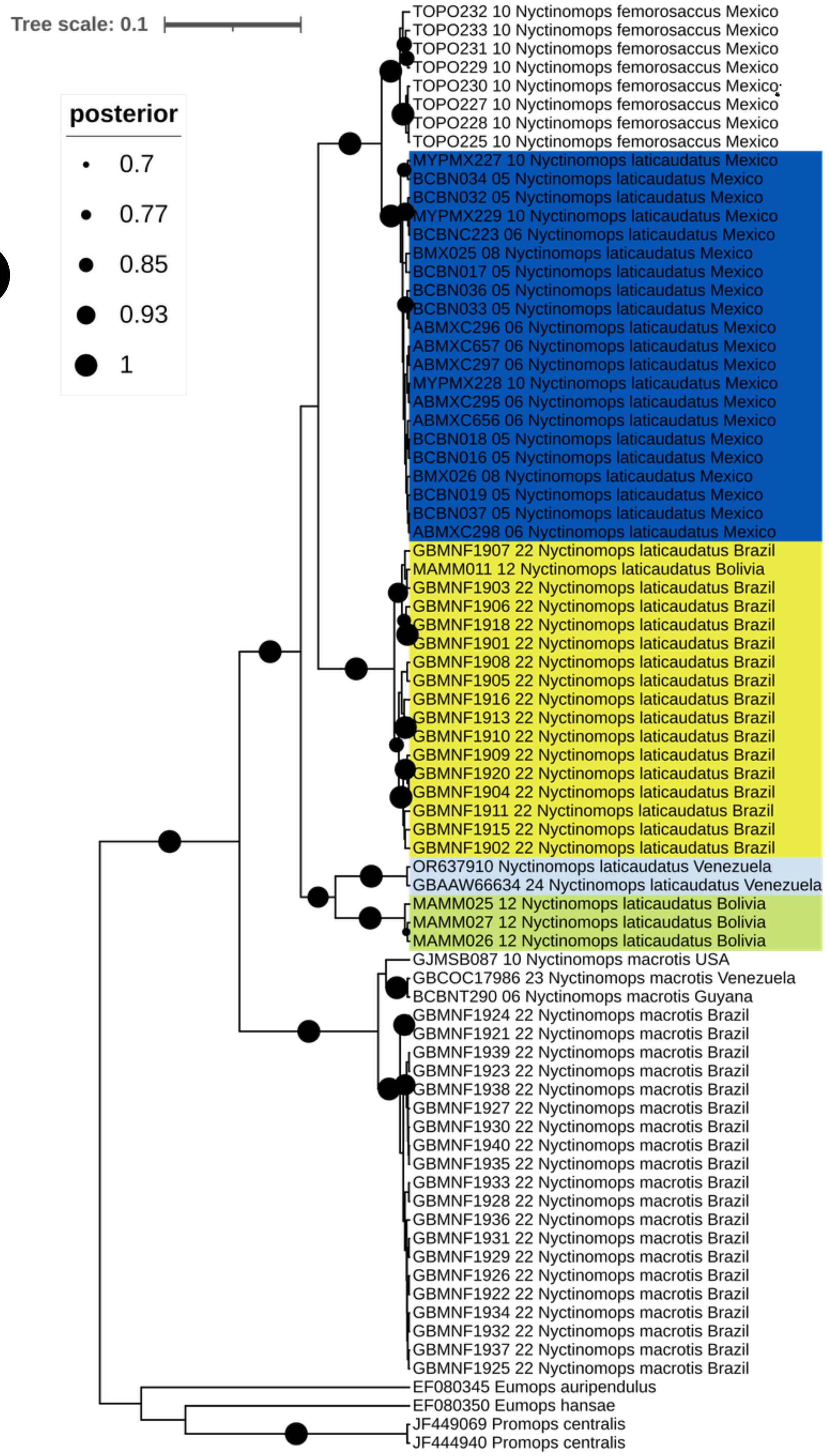

2

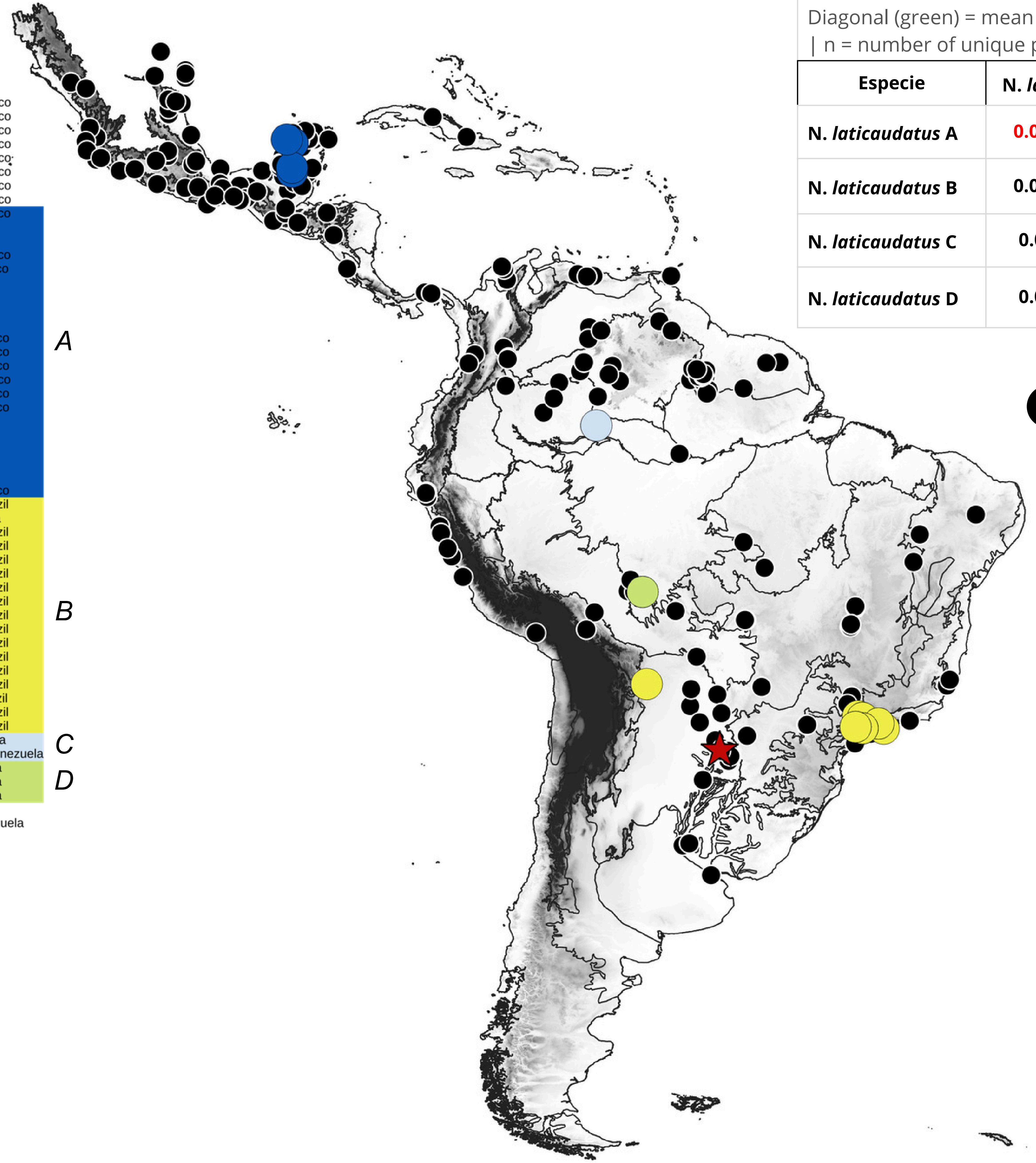

3

Mean genetic distances (Simple Distance)

Diagonal (green) = mean intraspecific distance | Off-diagonal (yellow) = mean interspecific distance | n = number of unique pairs

Especie	*N. laticaudatus* A	*N. laticaudatus* B	*N. laticaudatus* C	*N. laticaudatus* D
N. laticaudatus A	0.0017 (n=210)	0.0768 (n=357)	0.0682 (n=42)	0.0797 (n=63)
N. laticaudatus B	0.0768 (n=357)	0.0080 (n=136)	0.0783 (n=34)	0.0911 (n=51)
N. laticaudatus C	0.0682 (n=42)	0.0783 (n=34)	0.0015 (n=1)	0.0686 (n=6)
N. laticaudatus D	0.0797 (n=63)	0.0911 (n=51)	0.0686 (n=6)	0.0031 (n=3)

4

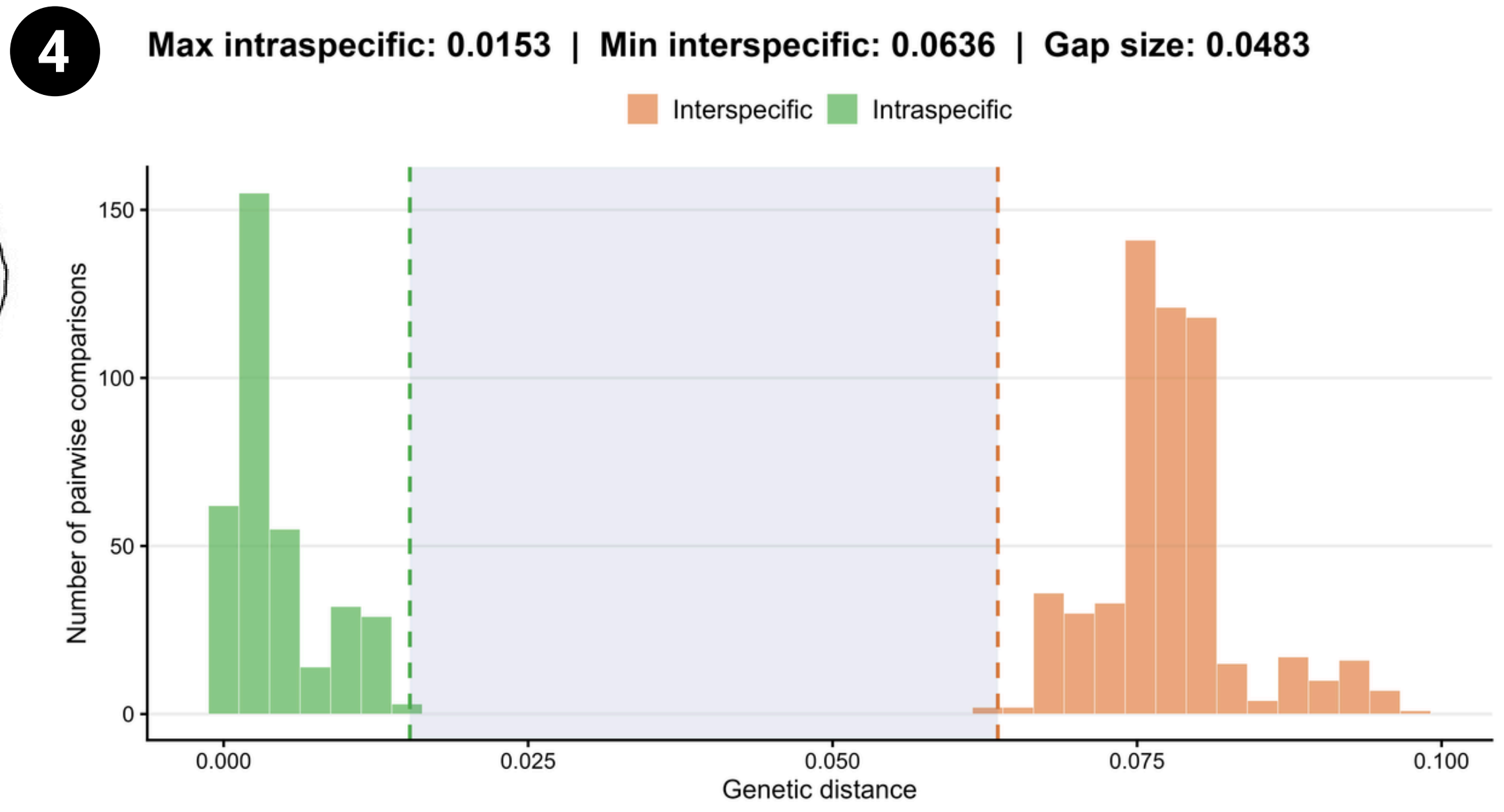

Figure 44

Fig. 44. Phylogenetic, geographic, and supporting evidence for candidate lineages in *Nyctinomops laticaudatus*. (1) Phylogenetic hypothesis based on COI sequences, with colored clades representing candidate lineages identified through molecular species delimitation. (2) Geographic distribution map, where colored points correspond to the lineages recovered in the phylogeny and black points represent the overall known distribution of the species complex. (3) Table of genetic distances (when sufficient data are available), reporting intra- and inter-lineage divergence among delimited groups. (4) Histogram of genetic distances illustrating the barcode gap. (5) Phylogenetic hypotheses from previous studies (when available), provided for comparison with our proposed delimitations. (6) Additional lines of evidence (occasionally available), such as geometric morphometrics or other independent datasets supporting lineage differentiation. Red star indicates holotype.

Note: *When this symbol (⚠) appears next to the phylogeny, it indicates that the delimitation should be interpreted with caution, as additional sampling is needed. When it appears next to the table, it means that genetic distances are low; therefore, we recommend that these results also be considered with caution. In both cases, we have retained the delimitations as inferred by our methods.*

Nyctinomops macrotis

1

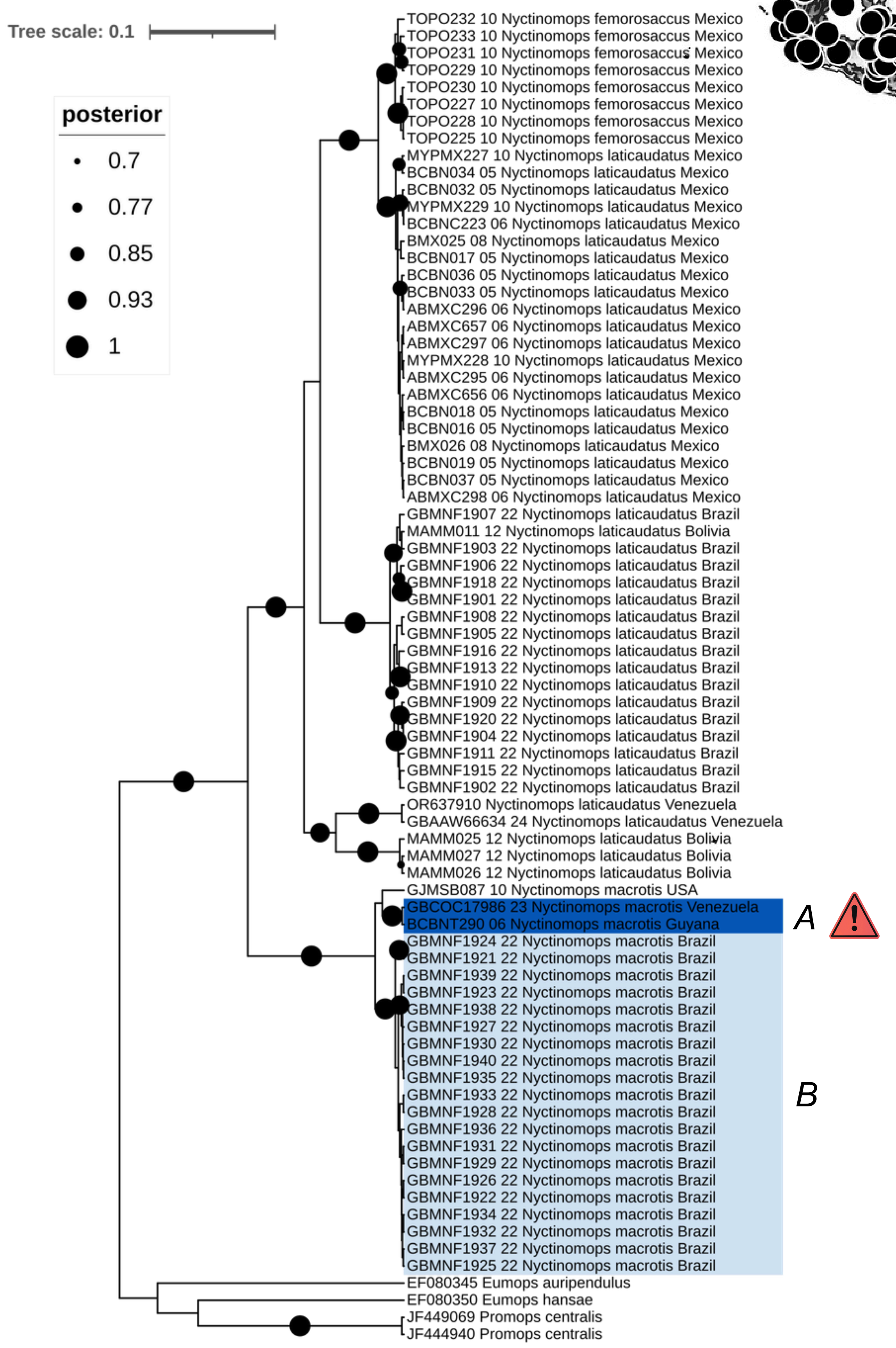

2

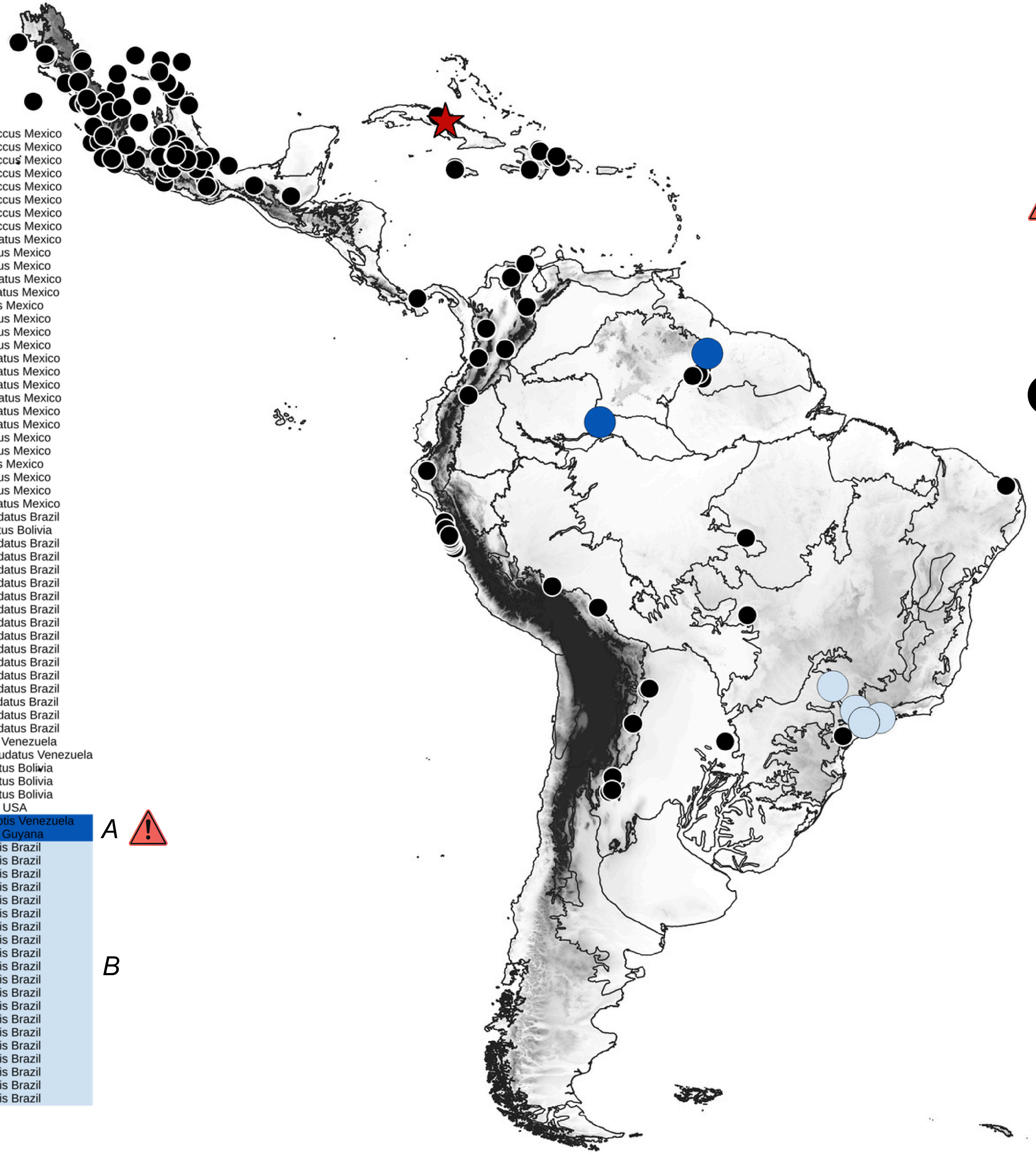

3

Mean genetic distances (Simple Distance)

Diagonal (green) = mean intraspecific distance | Off-diagonal (yellow) = mean interspecific distance | n = number of unique pairs

Especie	*N. macrotis* A	*N. macrotis* B
N. macrotis A	0.0015 (n=1)	0.0310 (n=40)
N. macrotis B	0.0310 (n=40)	0.0020 (n=190)

4

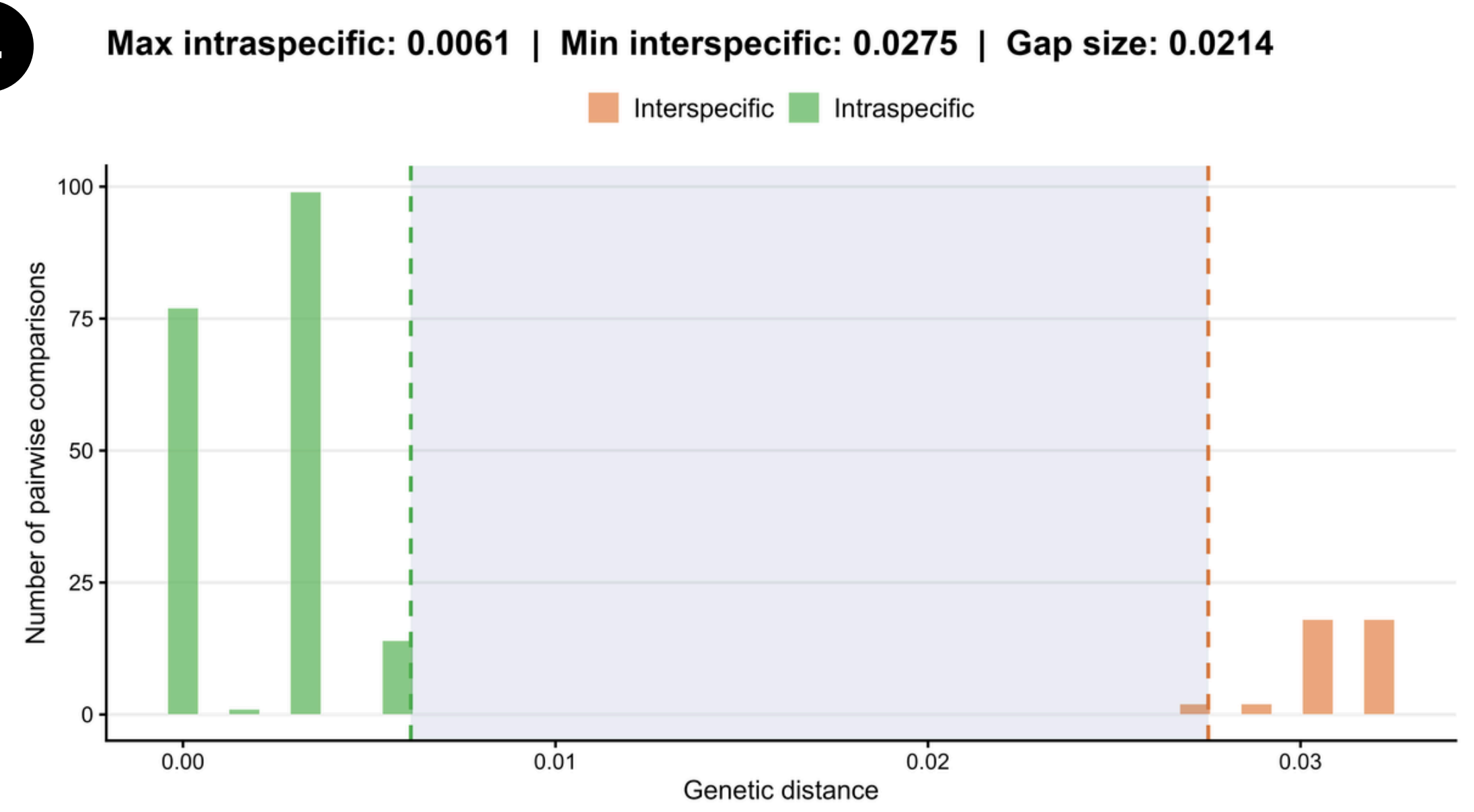

Figure 45

Fig. 45. Phylogenetic, geographic, and supporting evidence for candidate lineages in *Nyctinomops macrotis*. (1) Phylogenetic hypothesis based on COI sequences, with colored clades representing candidate lineages identified through molecular species delimitation. (2) Geographic distribution map, where colored points correspond to the lineages recovered in the phylogeny and black points represent the overall known distribution of the species complex. (3) Table of genetic distances (when sufficient data are available), reporting intra- and inter-lineage divergence among delimited groups. (4) Histogram of genetic distances illustrating the barcode gap. (5) Phylogenetic hypotheses from previous studies (when available), provided for comparison with our proposed delimitations. (6) Additional lines of evidence (occasionally available), such as geometric morphometrics or other independent datasets supporting lineage differentiation. Red star indicates holotype.

Note: *When this symbol (⚠) appears next to the phylogeny, it indicates that the delimitation should be interpreted with caution, as additional sampling is needed. When it appears next to the table, it means that genetic distances are low; therefore, we recommend that these results also be considered with caution. In both cases, we have retained the delimitations as inferred by our methods.*

Thyroptera tricolor

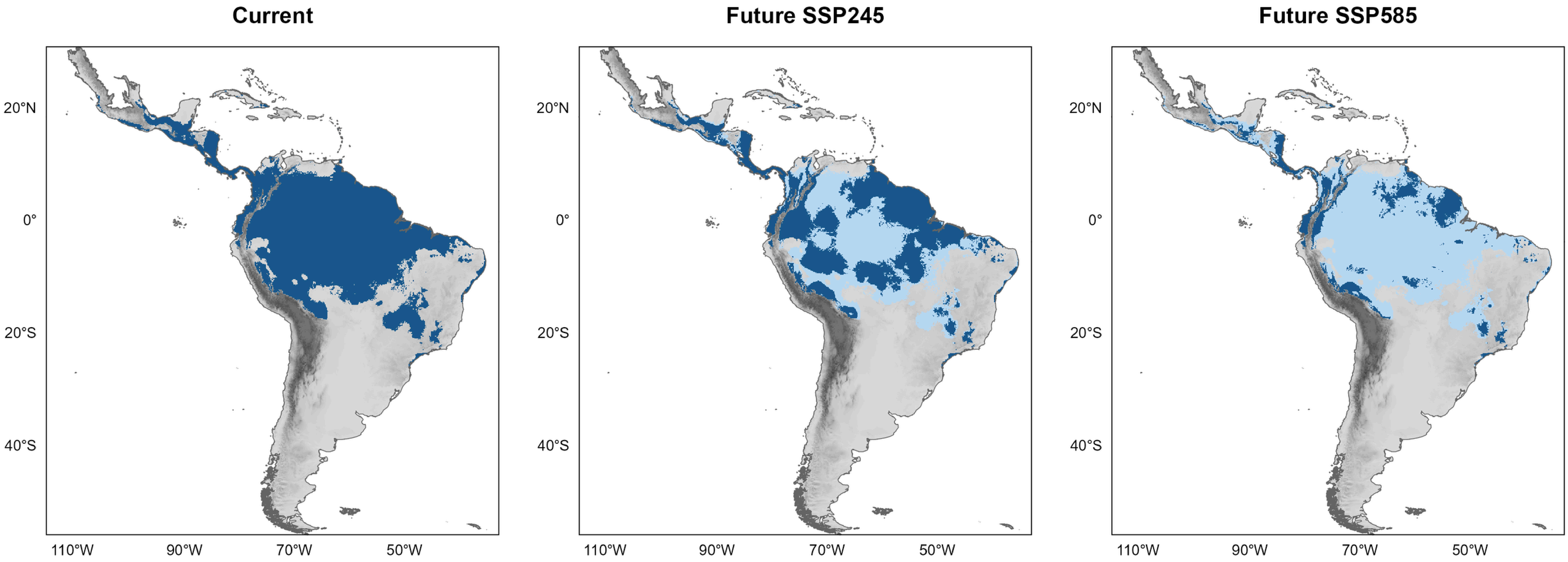

Figure 46

Fig. 46. Potential distribution models for *Thyroptera tricolor* under current and future climate scenarios. Maps display predicted habitat suitability across its range under current conditions (left), moderate emissions scenario SSP245 (middle), and high emissions scenario SSP585 (right) projected to 2070. Darker blue indicates higher probability of suitable habitat. The transition from dark to light blue represents progressive loss of habitat suitability, with areas of highest suitability (dark blue) diminishing substantially under more severe emission scenarios.

Furipterus horrens

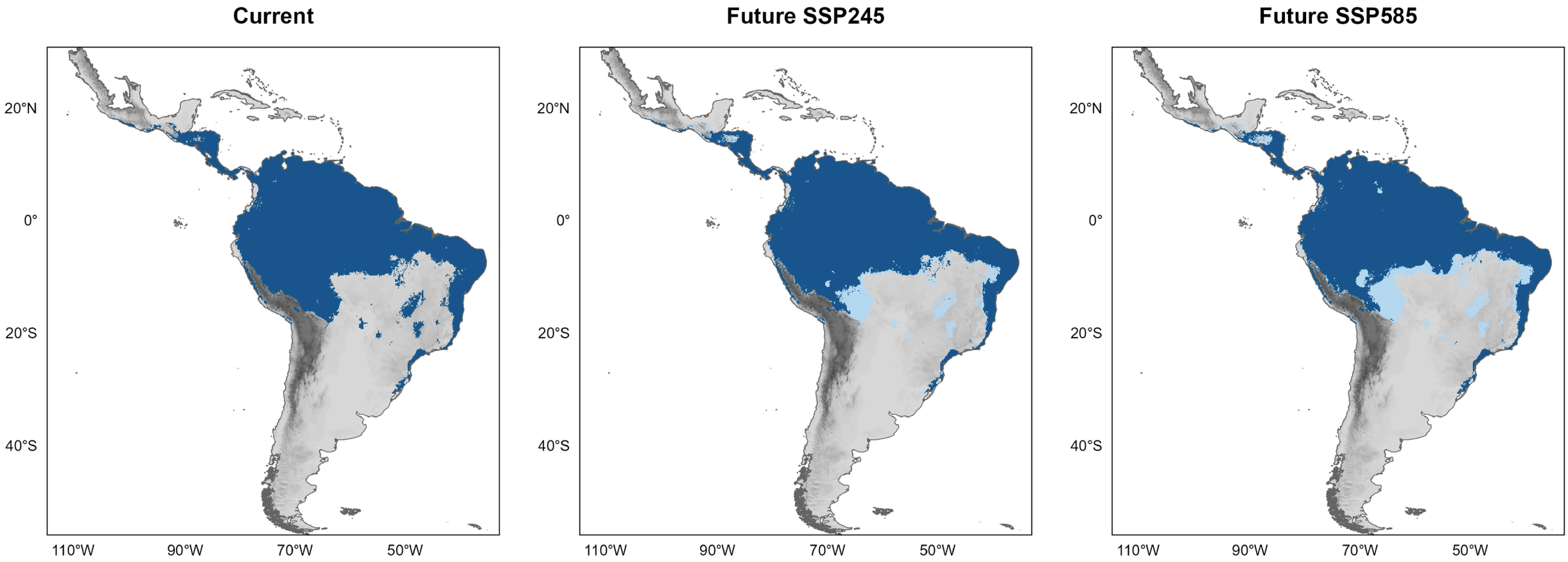

Figure 47

Fig. 47. Potential distribution models for *Furipterus horrens* under current and future climate scenarios. Maps display predicted habitat suitability across its range under current conditions (left), moderate emissions scenario SSP245 (middle), and high emissions scenario SSP585 (right) projected to 2070. Darker blue indicates higher probability of suitable habitat. The transition from dark to light blue represents progressive loss of habitat suitability, with areas of highest suitability (dark blue) diminishing substantially under more severe emission scenarios.

Noctilio albiventris

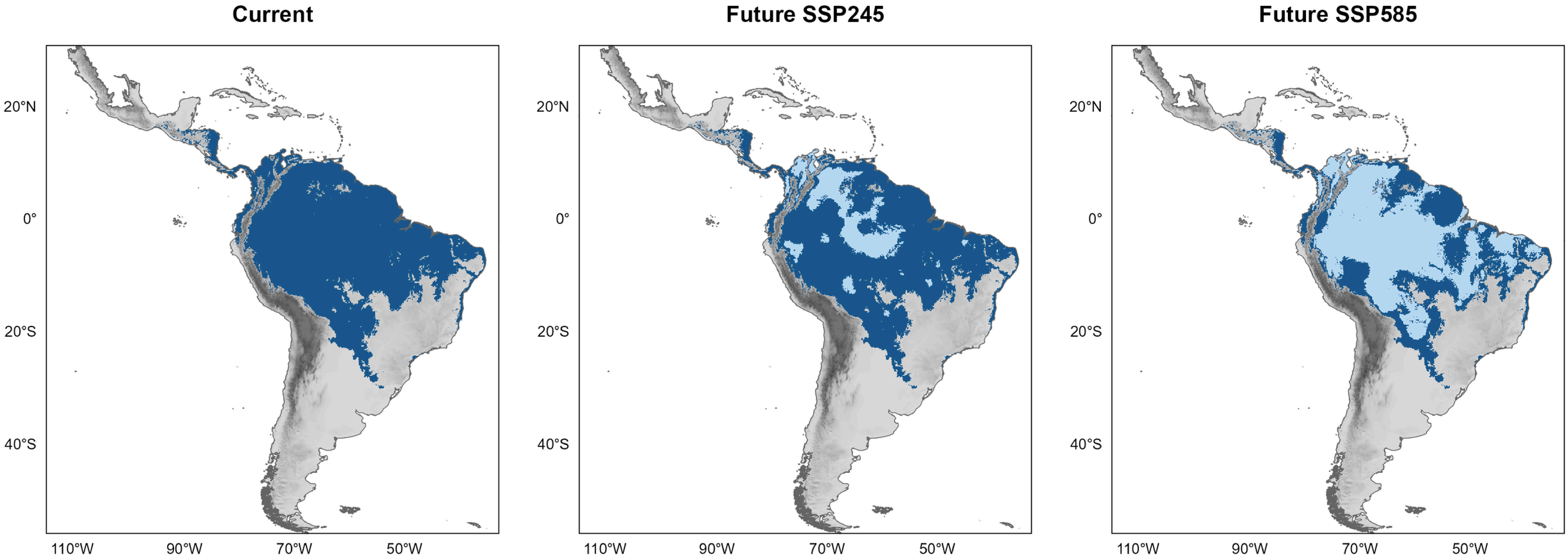

Figure 48

Fig. 48. Potential distribution models for *Noctilio albiventris* under current and future climate scenarios. Maps display predicted habitat suitability across its range under current conditions (left), moderate emissions scenario SSP245 (middle), and high emissions scenario SSP585 (right) projected to 2070. Darker blue indicates higher probability of suitable habitat. The transition from dark to light blue represents progressive loss of habitat suitability, with areas of highest suitability (dark blue) diminishing substantially under more severe emission scenarios.

Noctilio leporinus

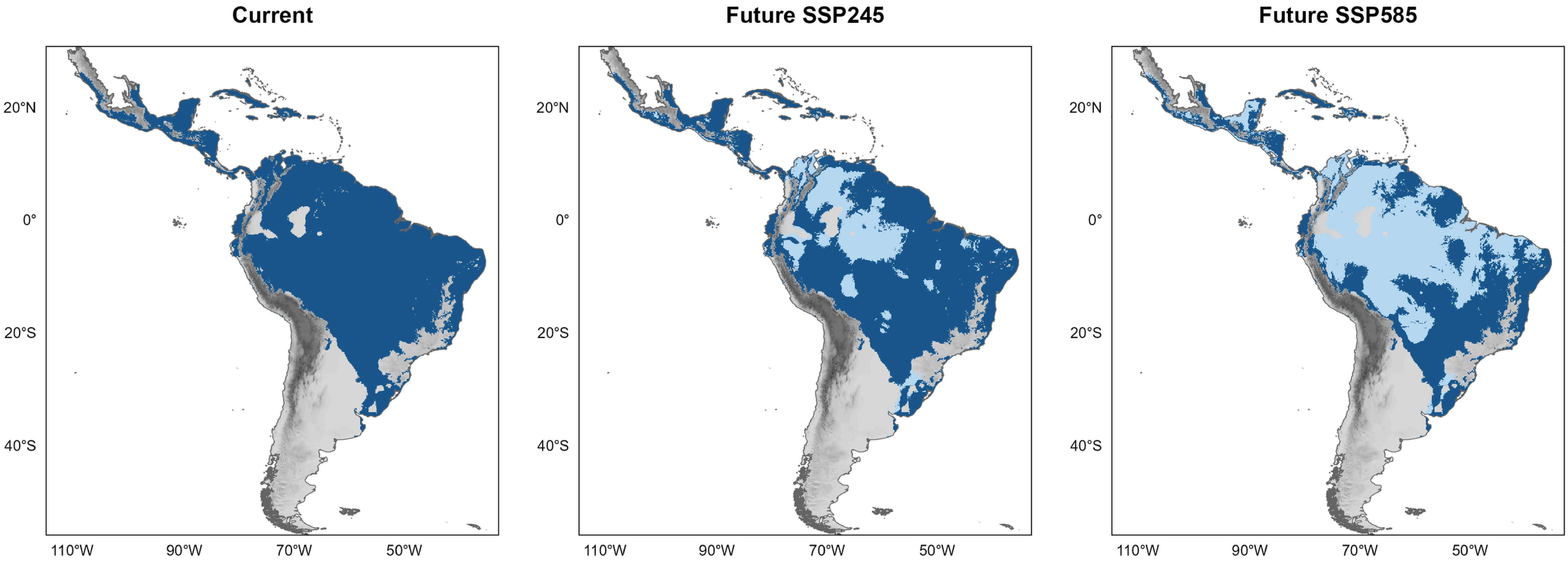

Figure 49

Fig. 49. Potential distribution models for *Noctilio leporinus* under current and future climate scenarios. Maps display predicted habitat suitability across its range under current conditions (left), moderate emissions scenario SSP245 (middle), and high emissions scenario SSP585 (right) projected to 2070. Darker blue indicates higher probability of suitable habitat. The transition from dark to light blue represents progressive loss of habitat suitability, with areas of highest suitability (dark blue) diminishing substantially under more severe emission scenarios.

Cormura brevirostris

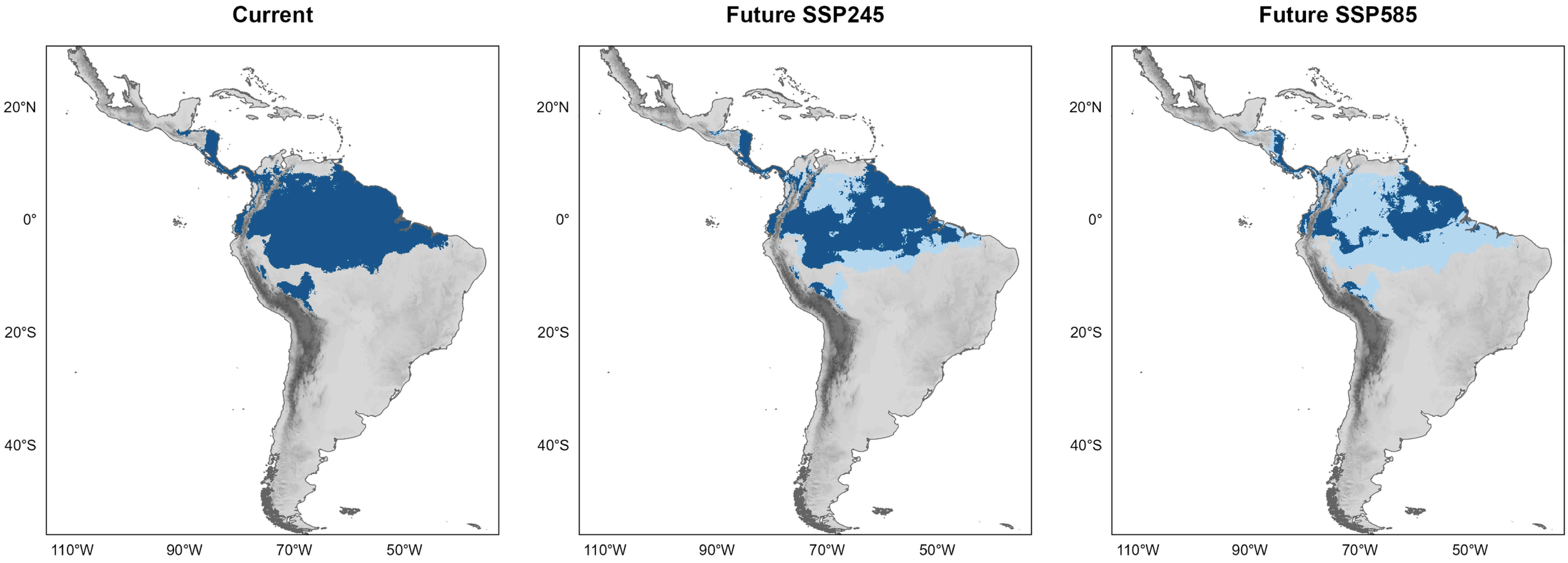

Figure 50

Fig. 50. Potential distribution models for *Cormura brevirostris* under current and future climate scenarios. Maps display predicted habitat suitability across its range under current conditions (left), moderate emissions scenario SSP245 (middle), and high emissions scenario SSP585 (right) projected to 2070. Darker blue indicates higher probability of suitable habitat. The transition from dark to light blue represents progressive loss of habitat suitability, with areas of highest suitability (dark blue) diminishing substantially under more severe emission scenarios.

Cyttarops alecto

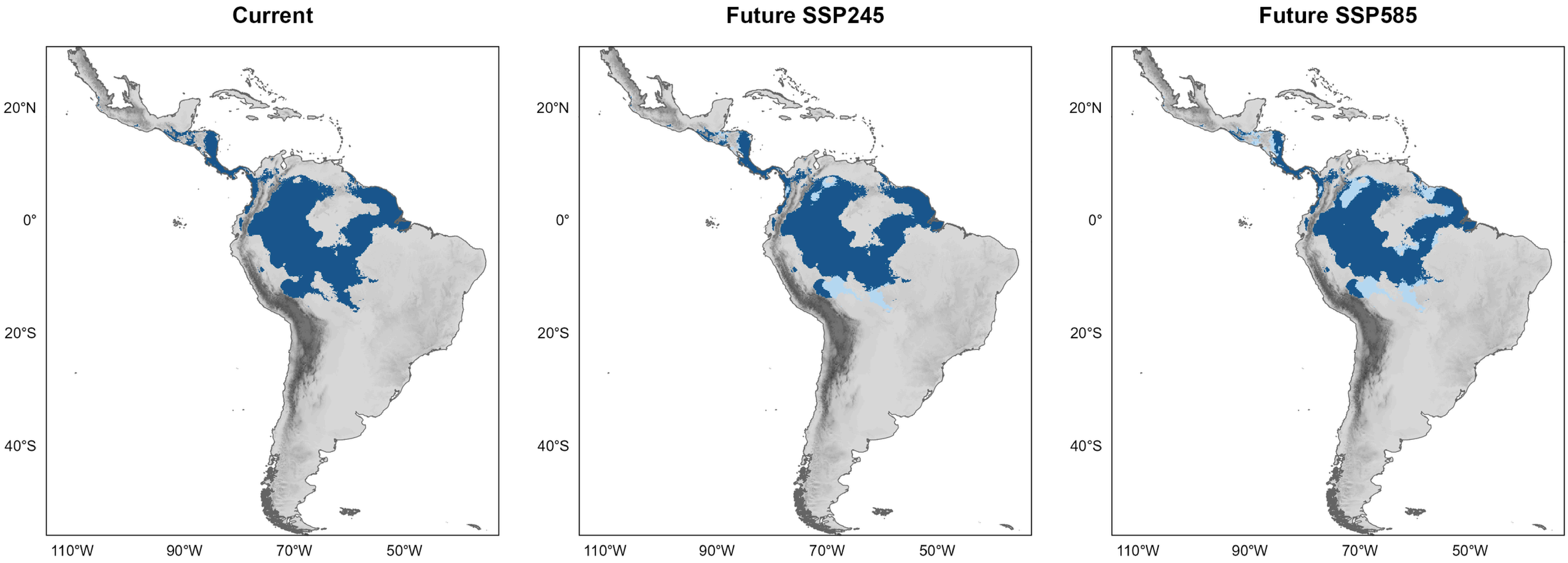

Figure 51

Fig. 51. Potential distribution models for *Cyttarops alecto* under current and future climate scenarios. Maps display predicted habitat suitability across its range under current conditions (left), moderate emissions scenario SSP245 (middle), and high emissions scenario SSP585 (right) projected to 2070. Darker blue indicates higher probability of suitable habitat. The transition from dark to light blue represents progressive loss of habitat suitability, with areas of highest suitability (dark blue) diminishing substantially under more severe emission scenarios.

Diclidurus albus

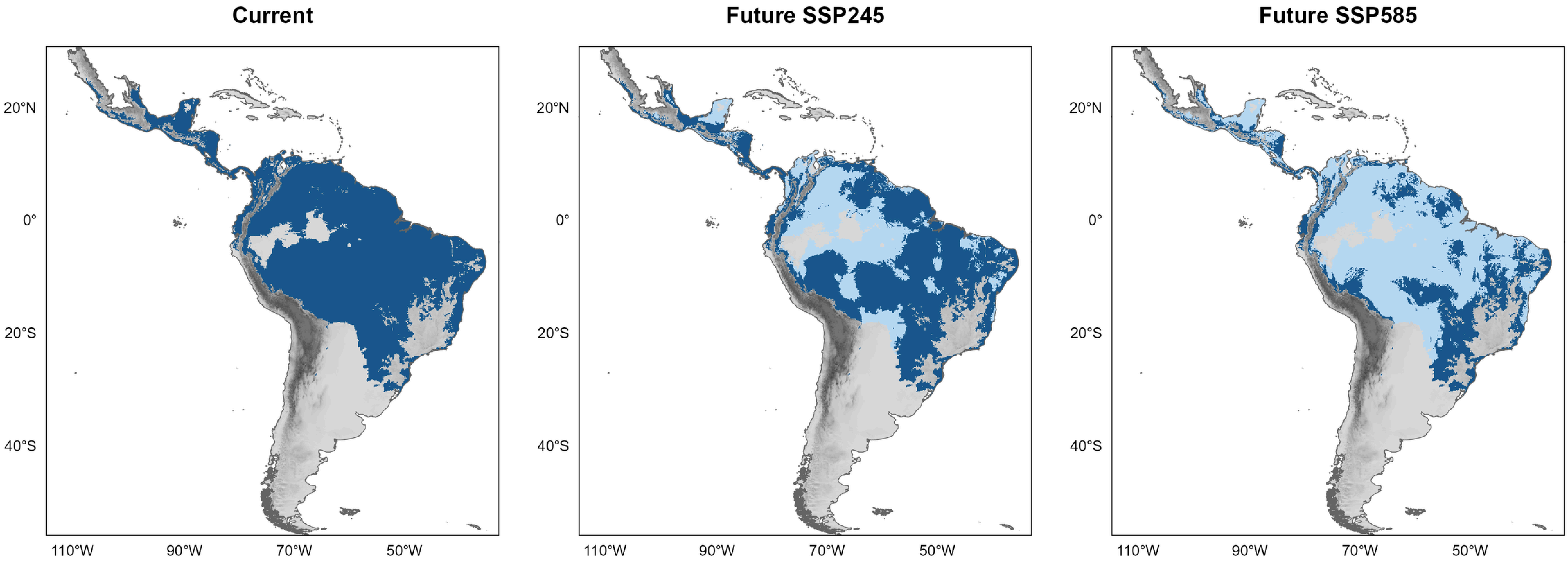

Figure 52

Fig. 52. Potential distribution models for *Diclidurus albus* under current and future climate scenarios. Maps display predicted habitat suitability across its range under current conditions (left), moderate emissions scenario SSP245 (middle), and high emissions scenario SSP585 (right) projected to 2070. Darker blue indicates higher probability of suitable habitat. The transition from dark to light blue represents progressive loss of habitat suitability, with areas of highest suitability (dark blue) diminishing substantially under more severe emission scenarios.

Peropteryx macrotis

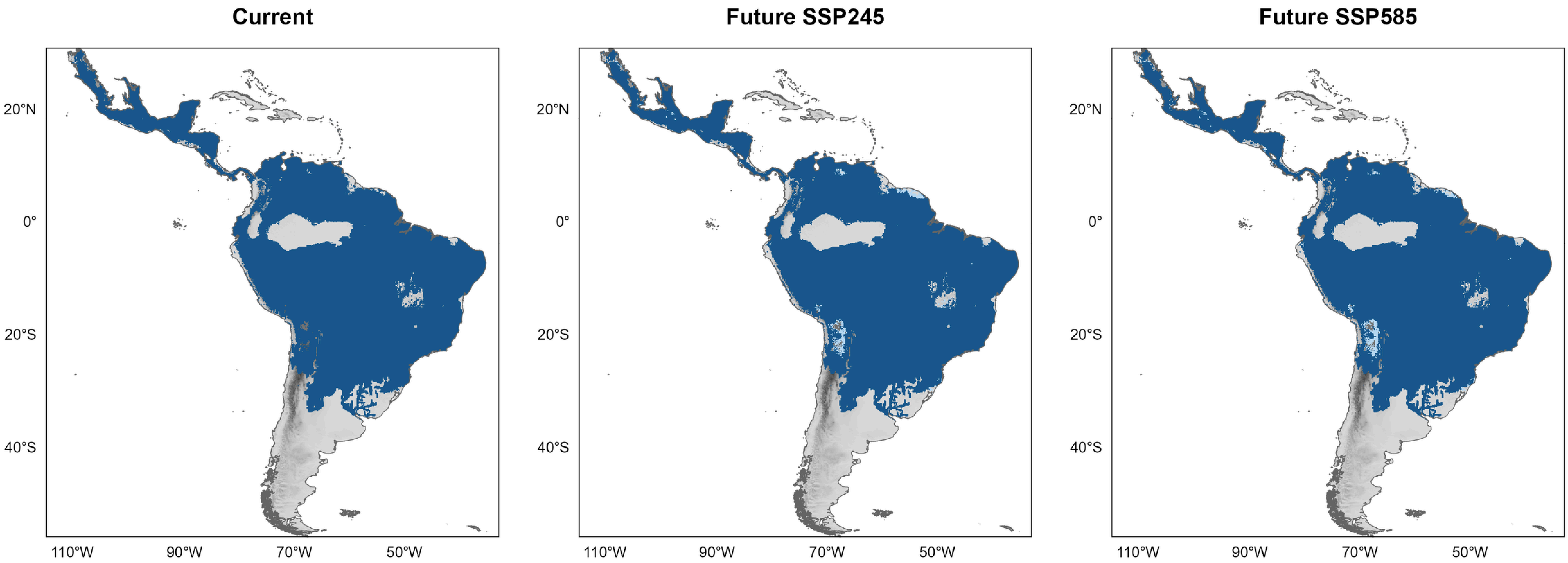

Figure 53

Fig. 53. Potential distribution models for *Peropteryx macrotis* under current and future climate scenarios. Maps display predicted habitat suitability across its range under current conditions (left), moderate emissions scenario SSP245 (middle), and high emissions scenario SSP585 (right) projected to 2070. Darker blue indicates higher probability of suitable habitat. The transition from dark to light blue represents progressive loss of habitat suitability, with areas of highest suitability (dark blue) diminishing substantially under more severe emission scenarios.

Rhynchonycteris naso

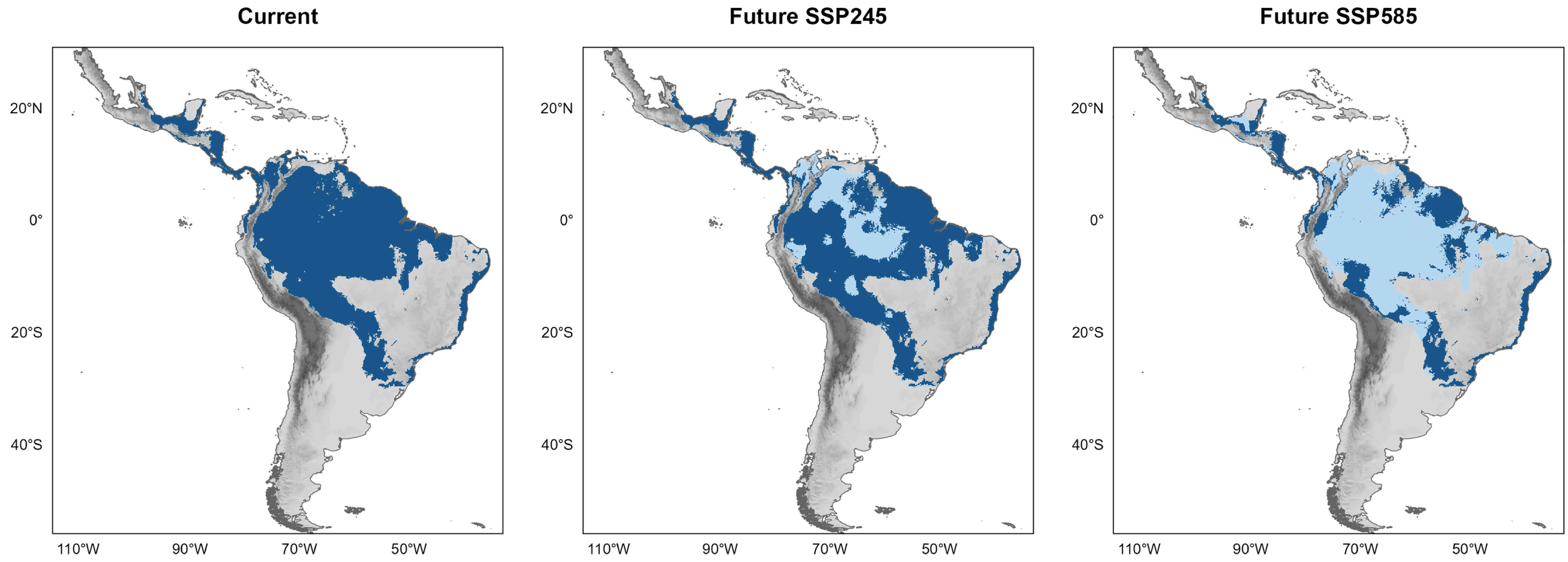

Figure 54

Fig. 54. Potential distribution models for *Rhynchonycteris naso* under current and future climate scenarios. Maps display predicted habitat suitability across its range under current conditions (left), moderate emissions scenario SSP245 (middle), and high emissions scenario SSP585 (right) projected to 2070. Darker blue indicates higher probability of suitable habitat. The transition from dark to light blue represents progressive loss of habitat suitability, with areas of highest suitability (dark blue) diminishing substantially under more severe emission scenarios.

Saccopteryx bilineata

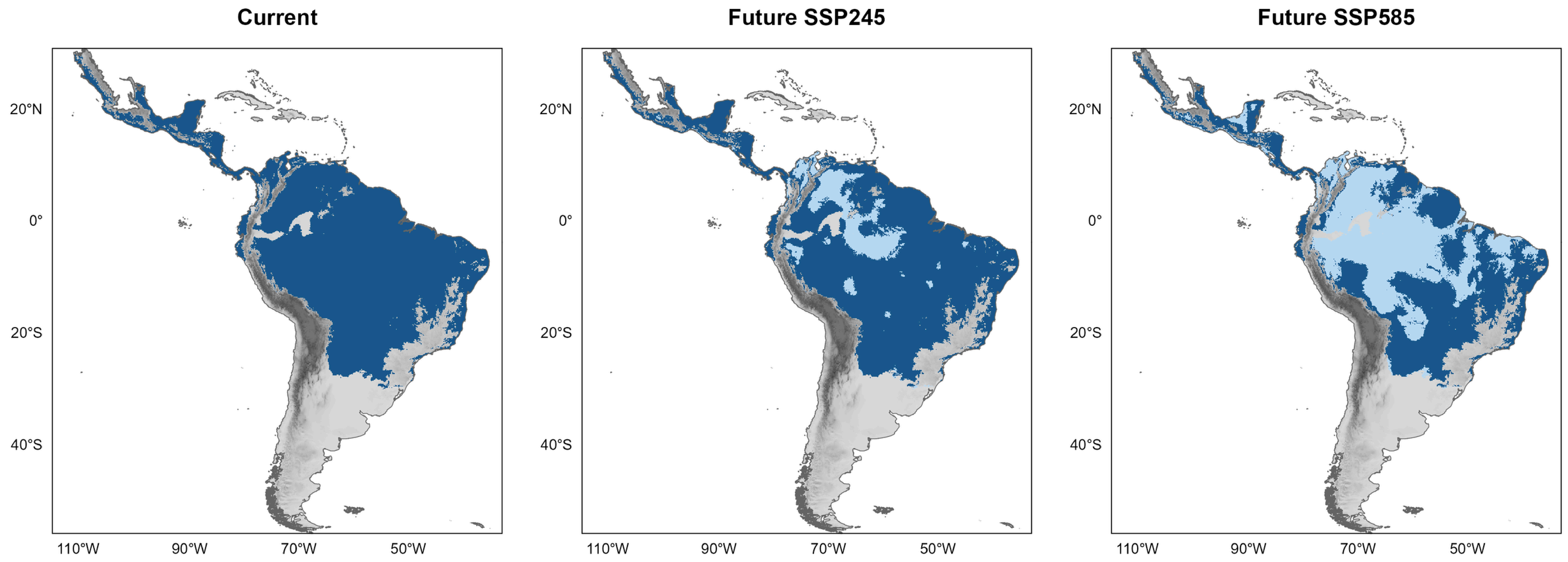

Figure 55

Fig. 55. Potential distribution models for *Saccopteryx bilineata* under current and future climate scenarios. Maps display predicted habitat suitability across its range under current conditions (left), moderate emissions scenario SSP245 (middle), and high emissions scenario SSP585 (right) projected to 2070. Darker blue indicates higher probability of suitable habitat. The transition from dark to light blue represents progressive loss of habitat suitability, with areas of highest suitability (dark blue) diminishing substantially under more severe emission scenarios.

Anoura caudifer

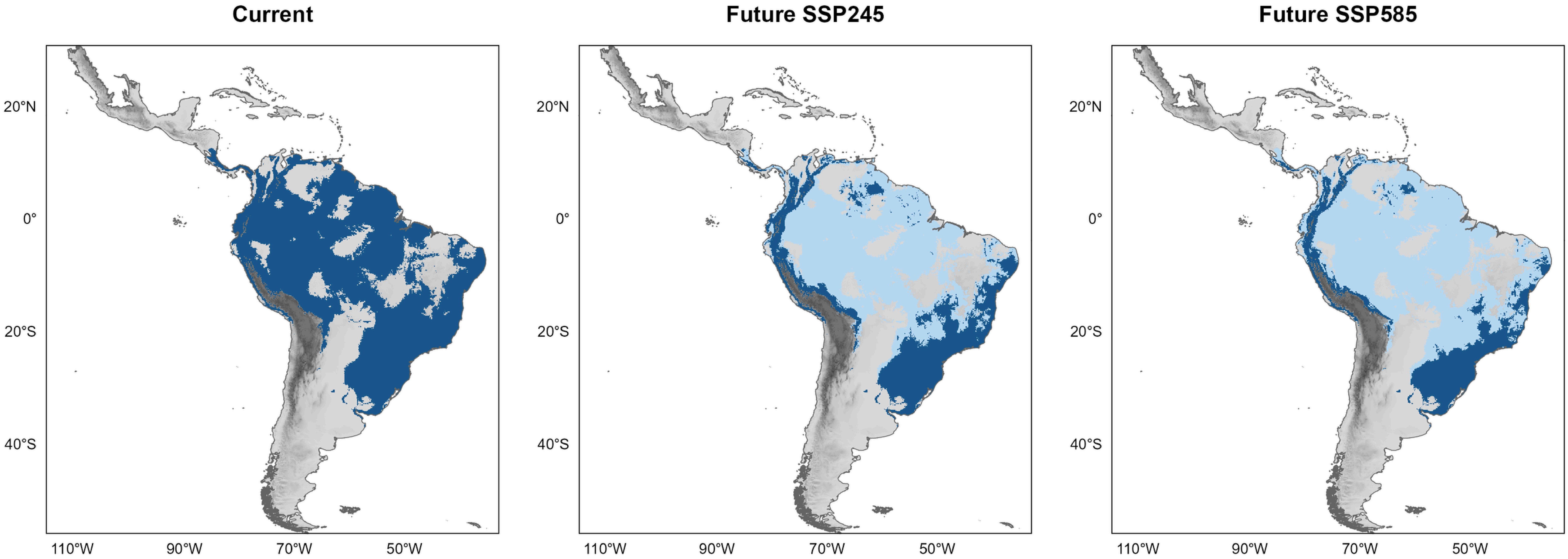

Figure 56

Fig. 56. Potential distribution models for *Anoura caudifer* under current and future climate scenarios. Maps display predicted habitat suitability across its range under current conditions (left), moderate emissions scenario SSP245 (middle), and high emissions scenario SSP585 (right) projected to 2070. Darker blue indicates higher probability of suitable habitat. The transition from dark to light blue represents progressive loss of habitat suitability, with areas of highest suitability (dark blue) diminishing substantially under more severe emission scenarios.

Anoura cultrata

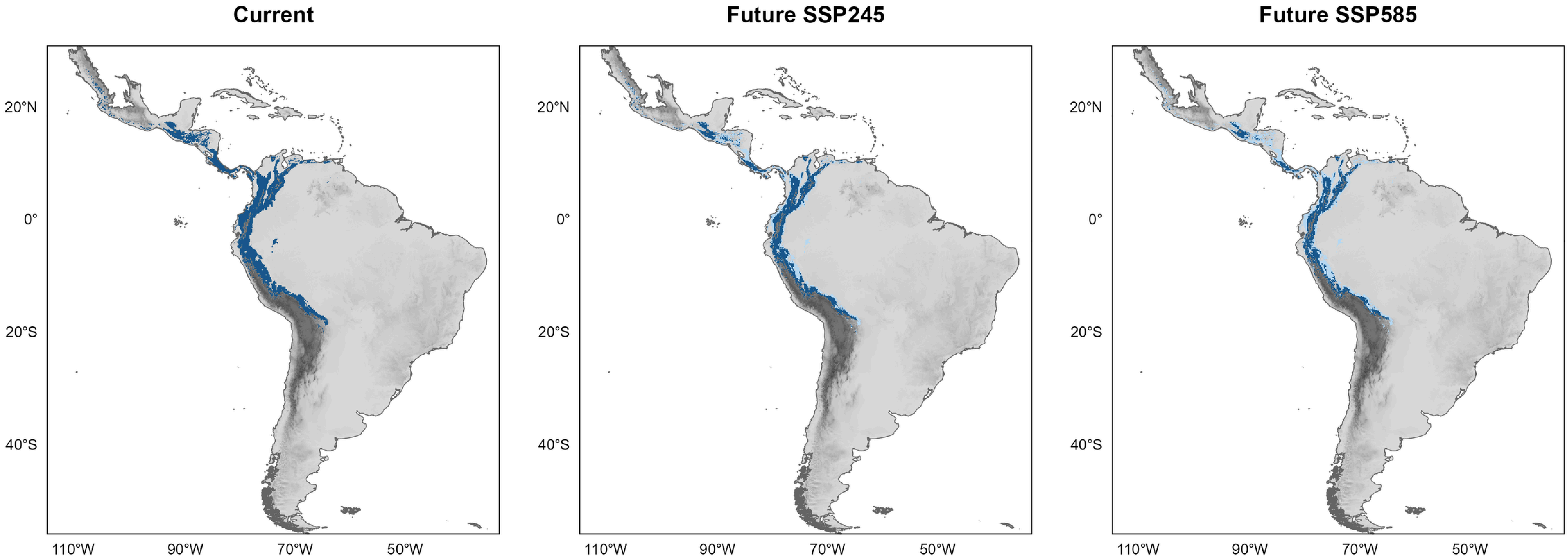

Figure 57

Fig. 57. Potential distribution models for *Anoura cultrata* under current and future climate scenarios. Maps display predicted habitat suitability across its range under current conditions (left), moderate emissions scenario SSP245 (middle), and high emissions scenario SSP585 (right) projected to 2070. Darker blue indicates higher probability of suitable habitat. The transition from dark to light blue represents progressive loss of habitat suitability, with areas of highest suitability (dark blue) diminishing substantially under more severe emission scenarios.

Anoura luismanueli

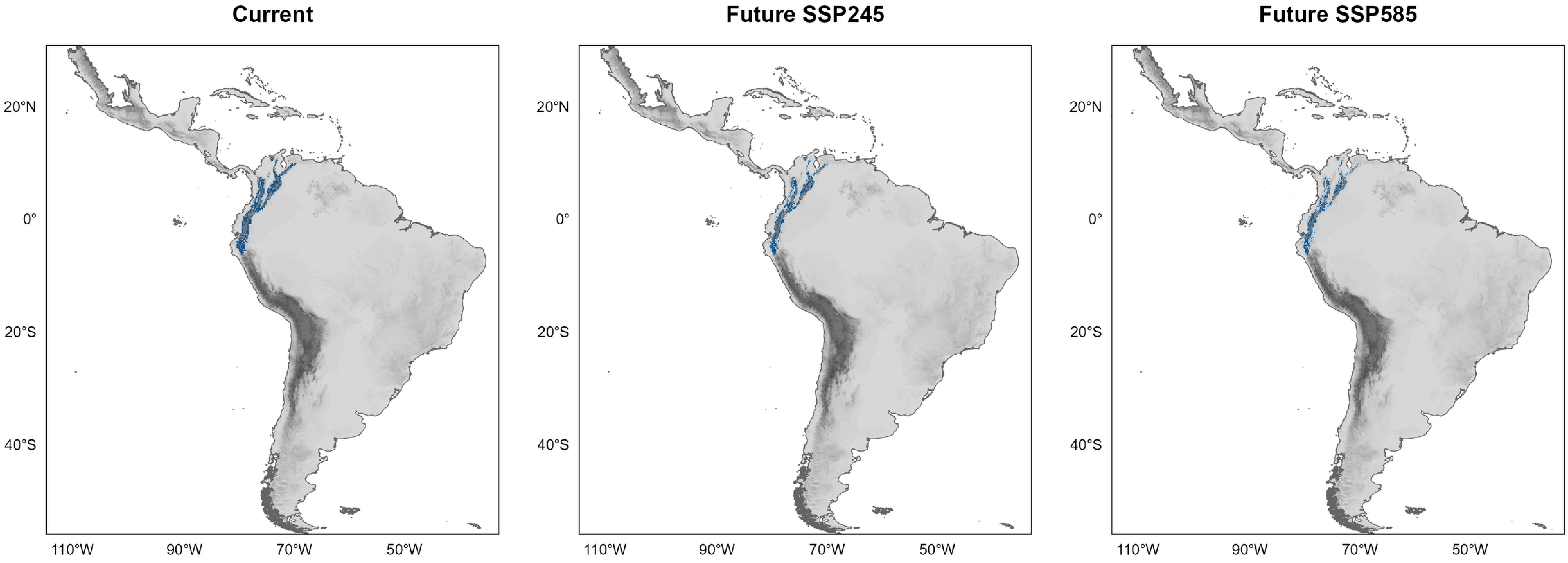

Figure 58

Fig. 58. Potential distribution models for *Anoura luismanueli* under current and future climate scenarios. Maps display predicted habitat suitability across its range under current conditions (left), moderate emissions scenario SSP245 (middle), and high emissions scenario SSP585 (right) projected to 2070. Darker blue indicates higher probability of suitable habitat. The transition from dark to light blue represents progressive loss of habitat suitability, with areas of highest suitability (dark blue) diminishing substantially under more severe emission scenarios.

Anoura peruana

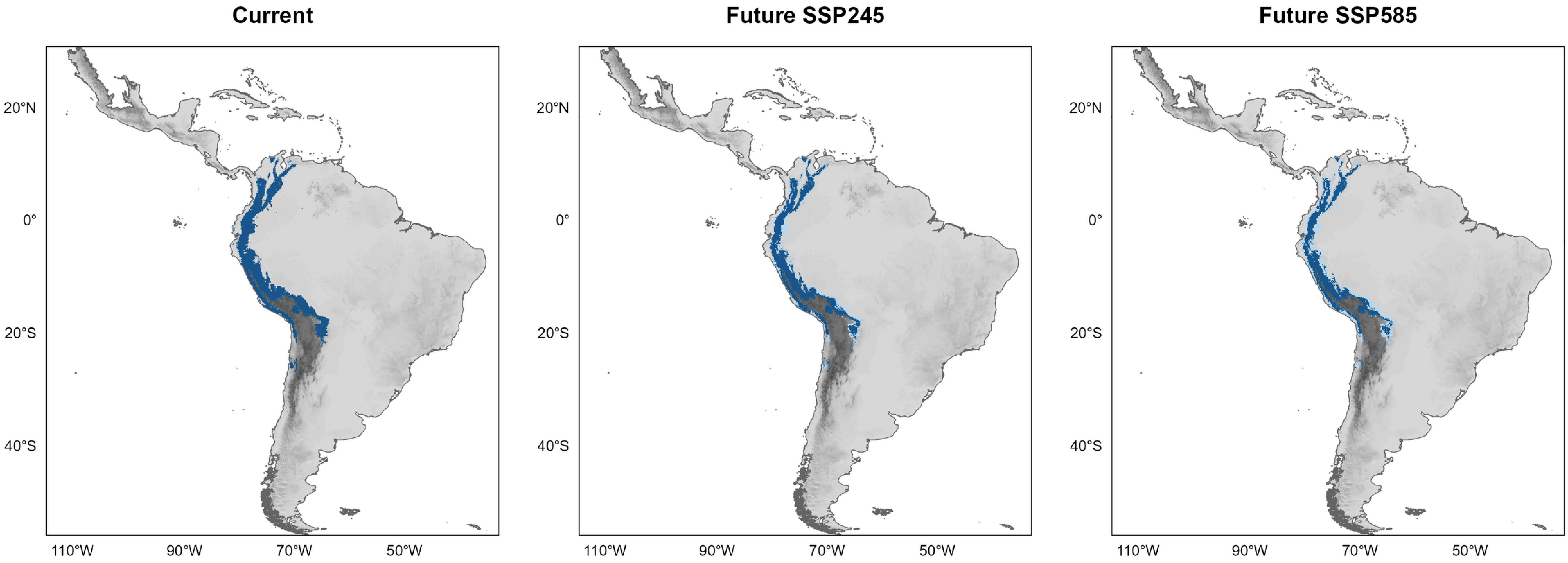

Figure 59

Fig. 59. Potential distribution models for *Anoura peruana* under current and future climate scenarios. Maps display predicted habitat suitability across its range under current conditions (left), moderate emissions scenario SSP245 (middle), and high emissions scenario SSP585 (right) projected to 2070. Darker blue indicates higher probability of suitable habitat. The transition from dark to light blue represents progressive loss of habitat suitability, with areas of highest suitability (dark blue) diminishing substantially under more severe emission scenarios.

Carollia castanea

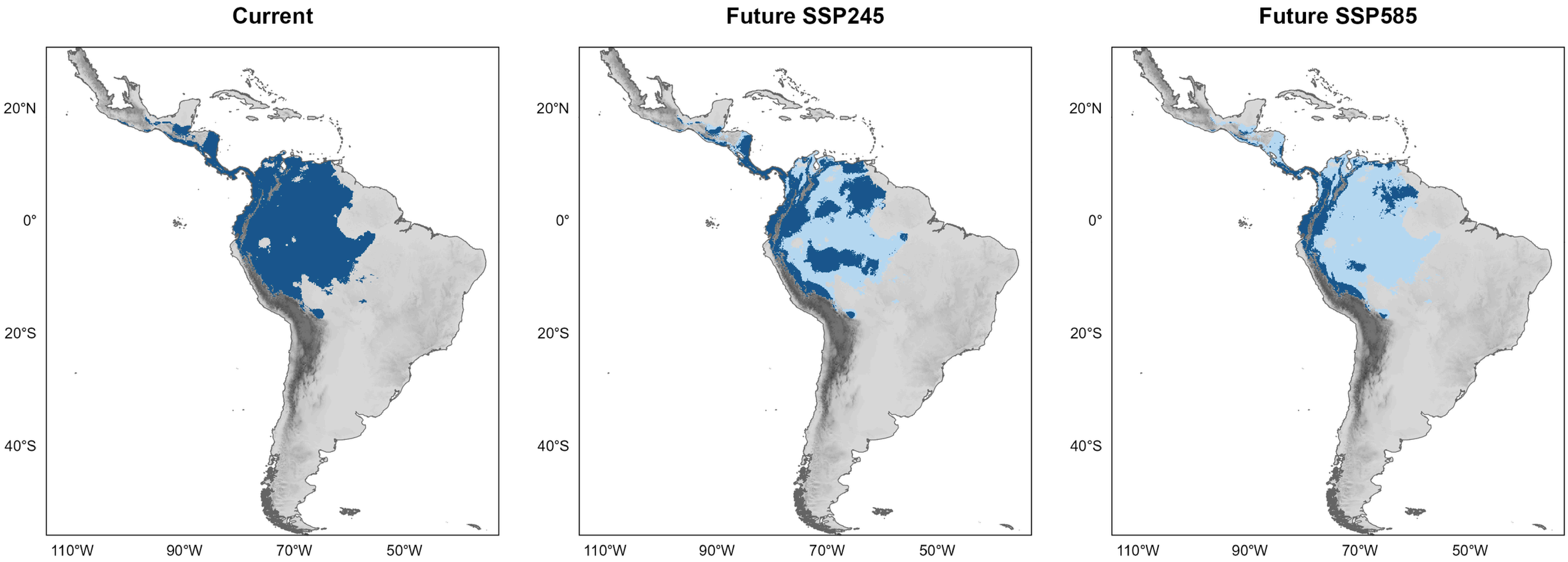

Figure 60

Fig. 60. Potential distribution models for *Carollia castanea* under current and future climate scenarios. Maps display predicted habitat suitability across its range under current conditions (left), moderate emissions scenario SSP245 (middle), and high emissions scenario SSP585 (right) projected to 2070. Darker blue indicates higher probability of suitable habitat. The transition from dark to light blue represents progressive loss of habitat suitability, with areas of highest suitability (dark blue) diminishing substantially under more severe emission scenarios.

Choeroniscus minor

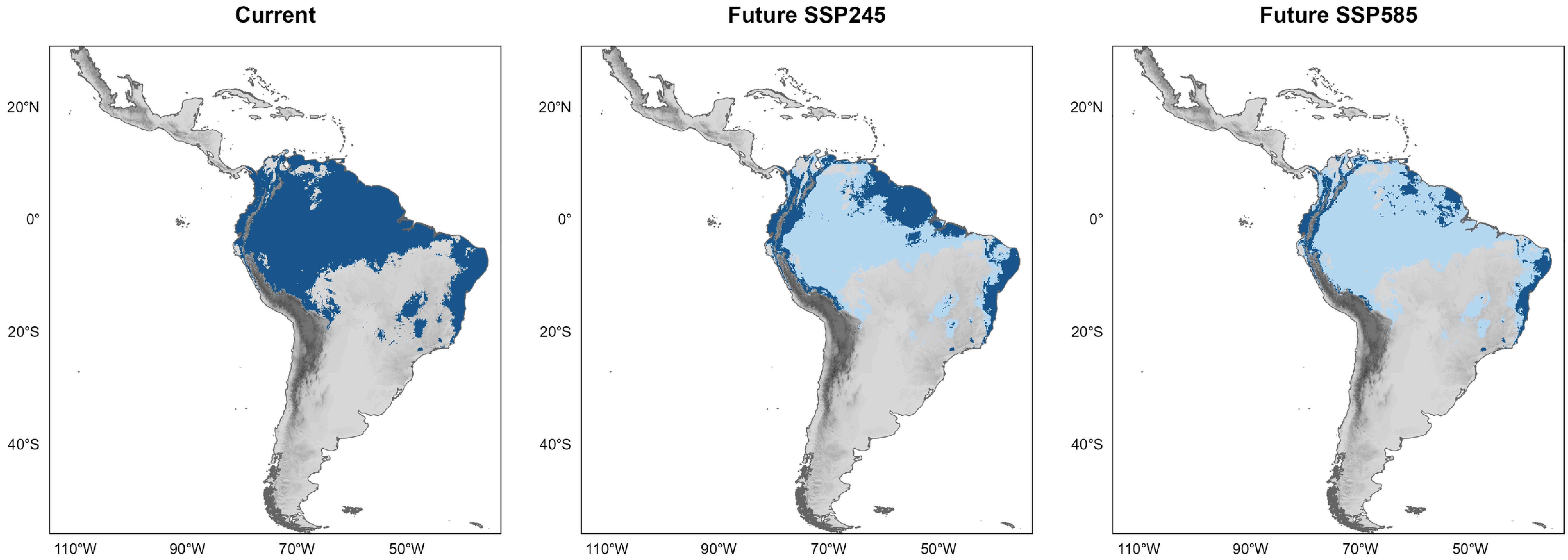

Figure 61

Fig. 61. Potential distribution models for *Choeroniscus minor* under current and future climate scenarios. Maps display predicted habitat suitability across its range under current conditions (left), moderate emissions scenario SSP245 (middle), and high emissions scenario SSP585 (right) projected to 2070. Darker blue indicates higher probability of suitable habitat. The transition from dark to light blue represents progressive loss of habitat suitability, with areas of highest suitability (dark blue) diminishing substantially under more severe emission scenarios.

Chrotopterus auritus

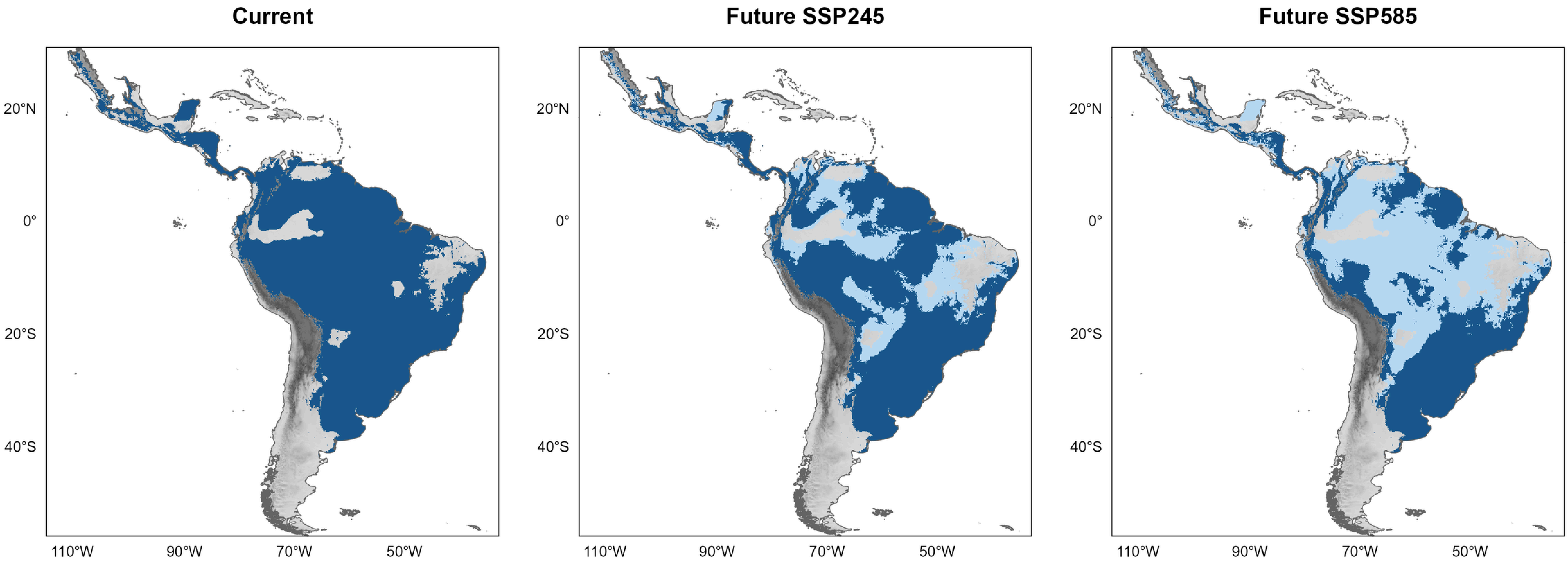

Figure 62

Fig. 62. Potential distribution models for *Chrotopterus auritus* under current and future climate scenarios. Maps display predicted habitat suitability across its range under current conditions (left), moderate emissions scenario SSP245 (middle), and high emissions scenario SSP585 (right) projected to 2070. Darker blue indicates higher probability of suitable habitat. The transition from dark to light blue represents progressive loss of habitat suitability, with areas of highest suitability (dark blue) diminishing substantially under more severe emission scenarios.

Dermanura anderseni

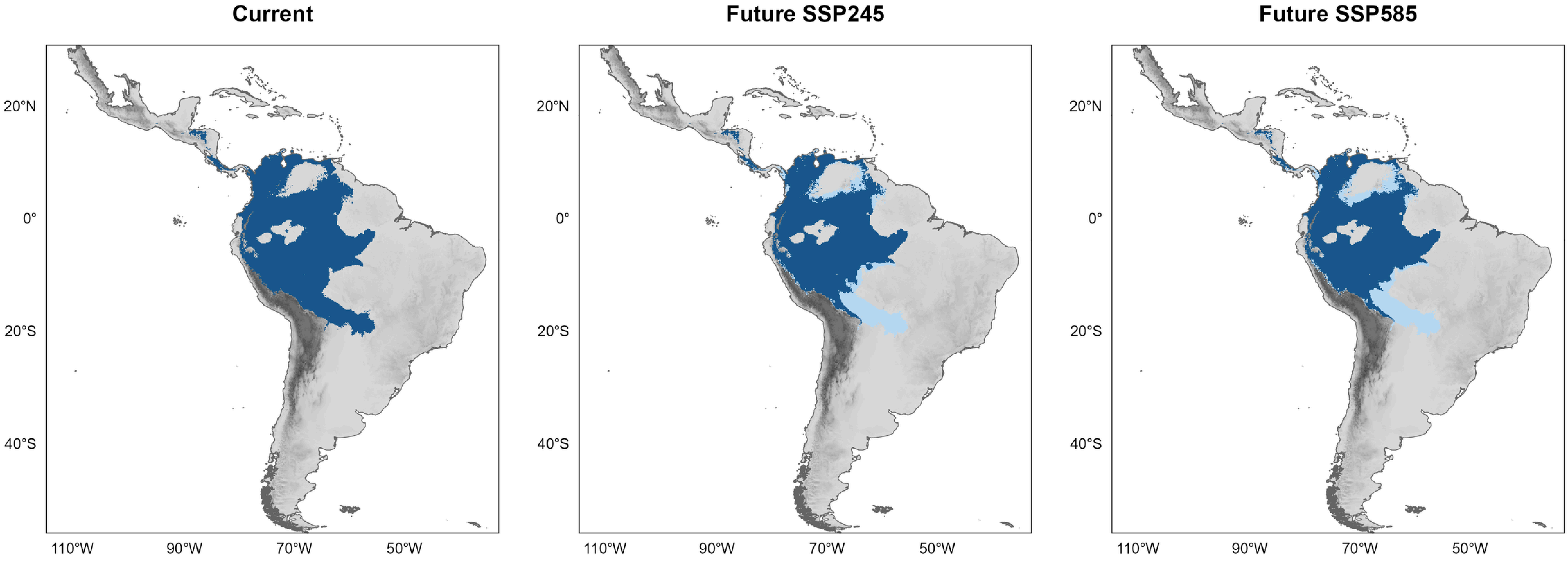

Figure 63

Fig. 63. Potential distribution models for *Dermanura anderseni* under current and future climate scenarios. Maps display predicted habitat suitability across its range under current conditions (left), moderate emissions scenario SSP245 (middle), and high emissions scenario SSP585 (right) projected to 2070. Darker blue indicates higher probability of suitable habitat. The transition from dark to light blue represents progressive loss of habitat suitability, with areas of highest suitability (dark blue) diminishing substantially under more severe emission scenarios.

Dermanura watsoni

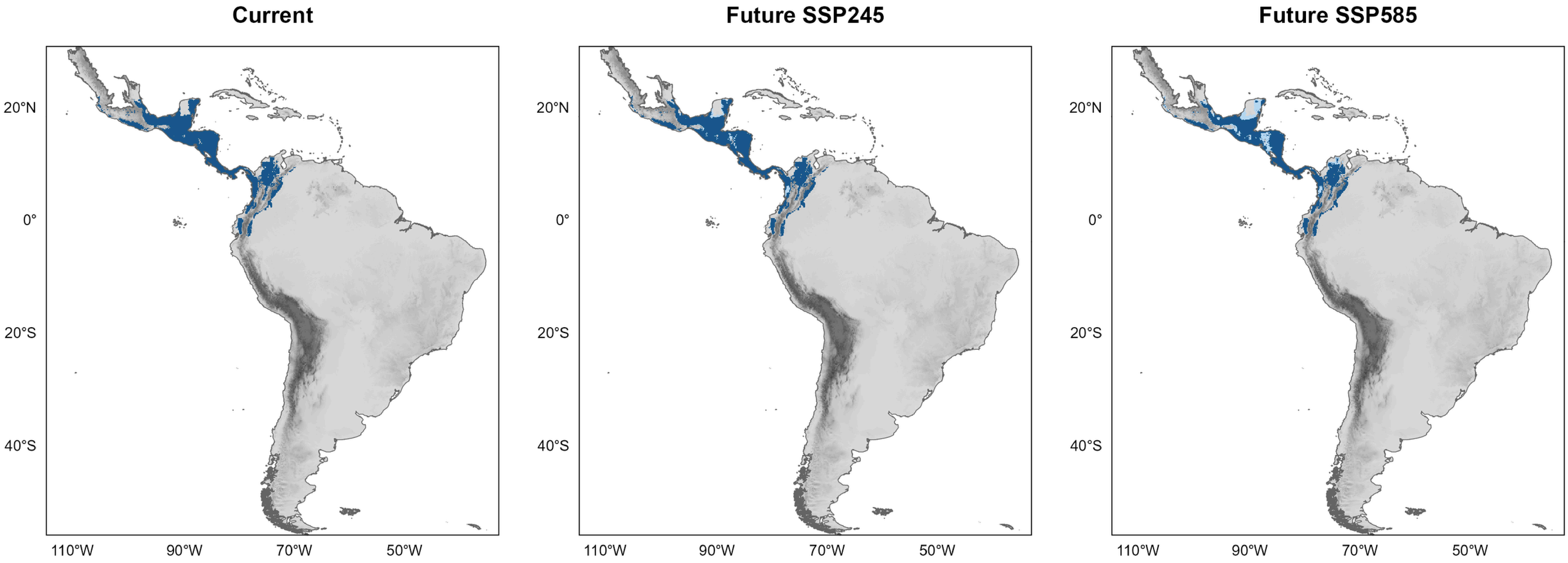

Figure 64

Fig. 64. Potential distribution models for *Dermanura watsoni* under current and future climate scenarios. Maps display predicted habitat suitability across its range under current conditions (left), moderate emissions scenario SSP245 (middle), and high emissions scenario SSP585 (right) projected to 2070. Darker blue indicates higher probability of suitable habitat. The transition from dark to light blue represents progressive loss of habitat suitability, with areas of highest suitability (dark blue) diminishing substantially under more severe emission scenarios.

Desmodus rotundus

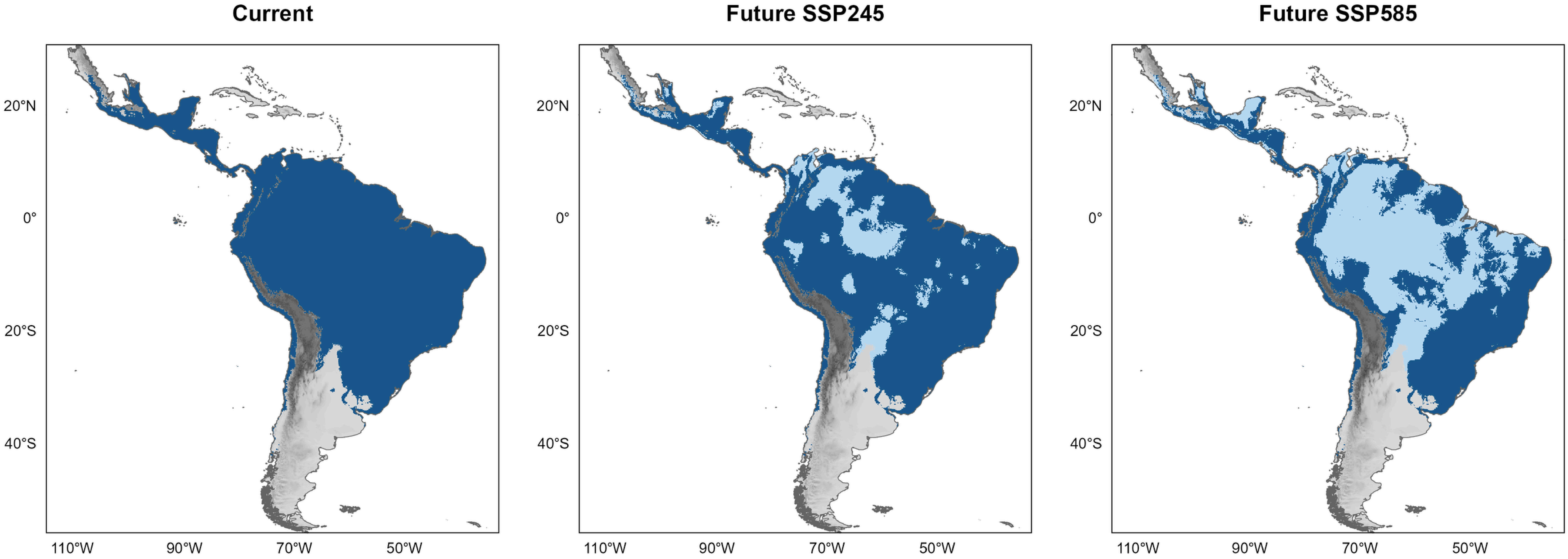

Figure 65

Fig. 65. Potential distribution models for *Desmodus rotundus* under current and future climate scenarios. Maps display predicted habitat suitability across its range under current conditions (left), moderate emissions scenario SSP245 (middle), and high emissions scenario SSP585 (right) projected to 2070. Darker blue indicates higher probability of suitable habitat. The transition from dark to light blue represents progressive loss of habitat suitability, with areas of highest suitability (dark blue) diminishing substantially under more severe emission scenarios.

Glyphonycteris sylvestris

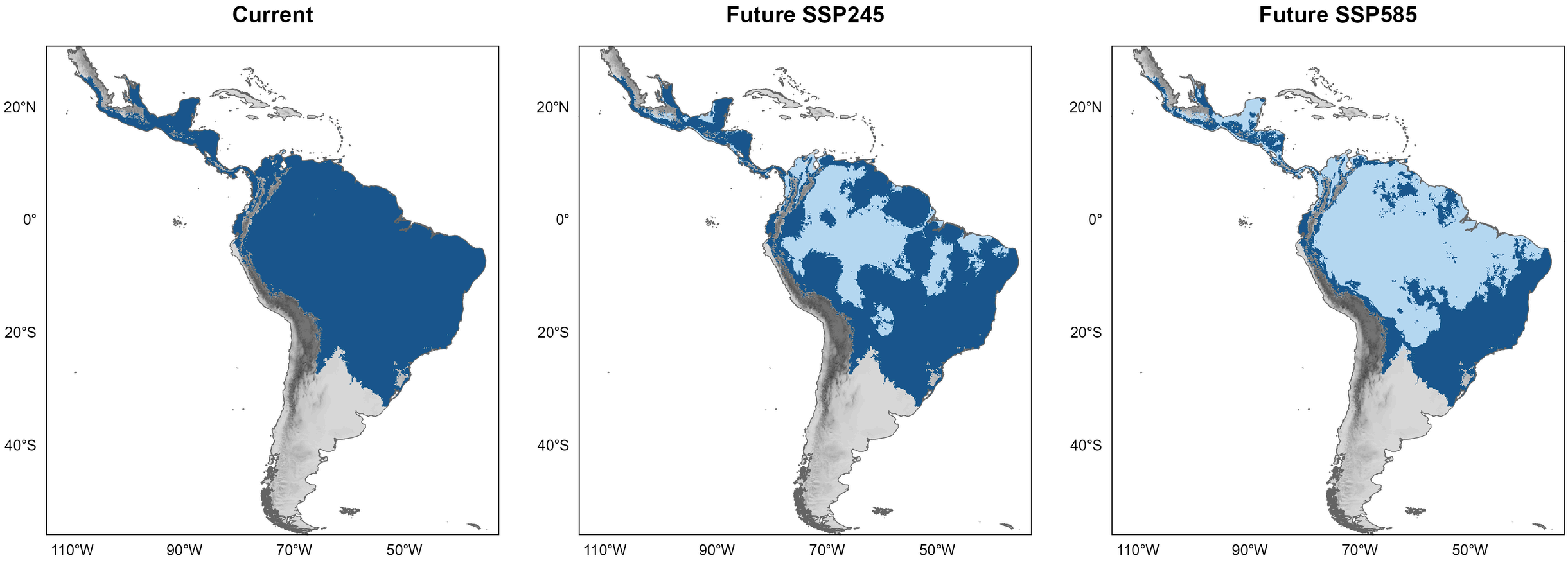

Figure 66

Fig. 66. Potential distribution models for G*lyphonycteris sylvestris* under current and future climate scenarios. Maps display predicted habitat suitability across its range under current conditions (left), moderate emissions scenario SSP245 (middle), and high emissions scenario SSP585 (right) projected to 2070. Darker blue indicates higher probability of suitable habitat. The transition from dark to light blue represents progressive loss of habitat suitability, with areas of highest suitability (dark blue) diminishing substantially under more severe emission scenarios.

Hsunycteris thomasi

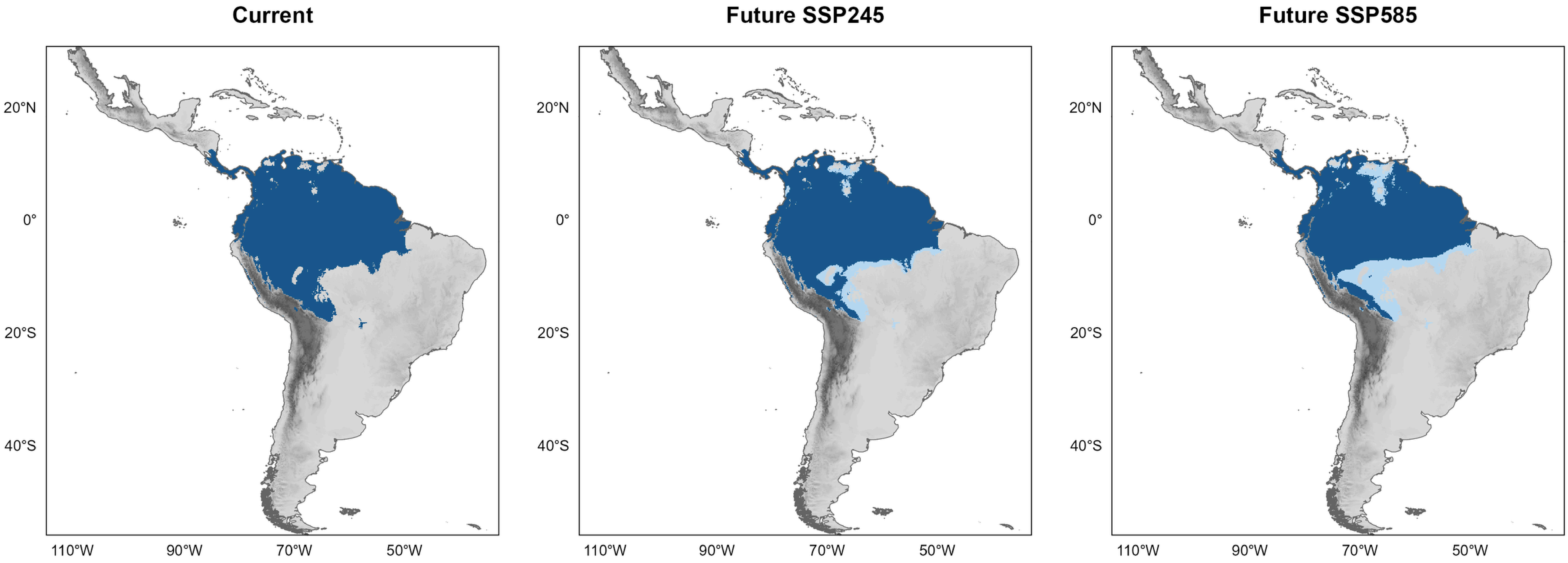

Figure 67

Fig. 67. Potential distribution models for *Hsunycteris thomasi* under current and future climate scenarios. Maps display predicted habitat suitability across its range under current conditions (left), moderate emissions scenario SSP245 (middle), and high emissions scenario SSP585 (right) projected to 2070. Darker blue indicates higher probability of suitable habitat. The transition from dark to light blue represents progressive loss of habitat suitability, with areas of highest suitability (dark blue) diminishing substantially under more severe emission scenarios.

Hylonycteris underwoodi

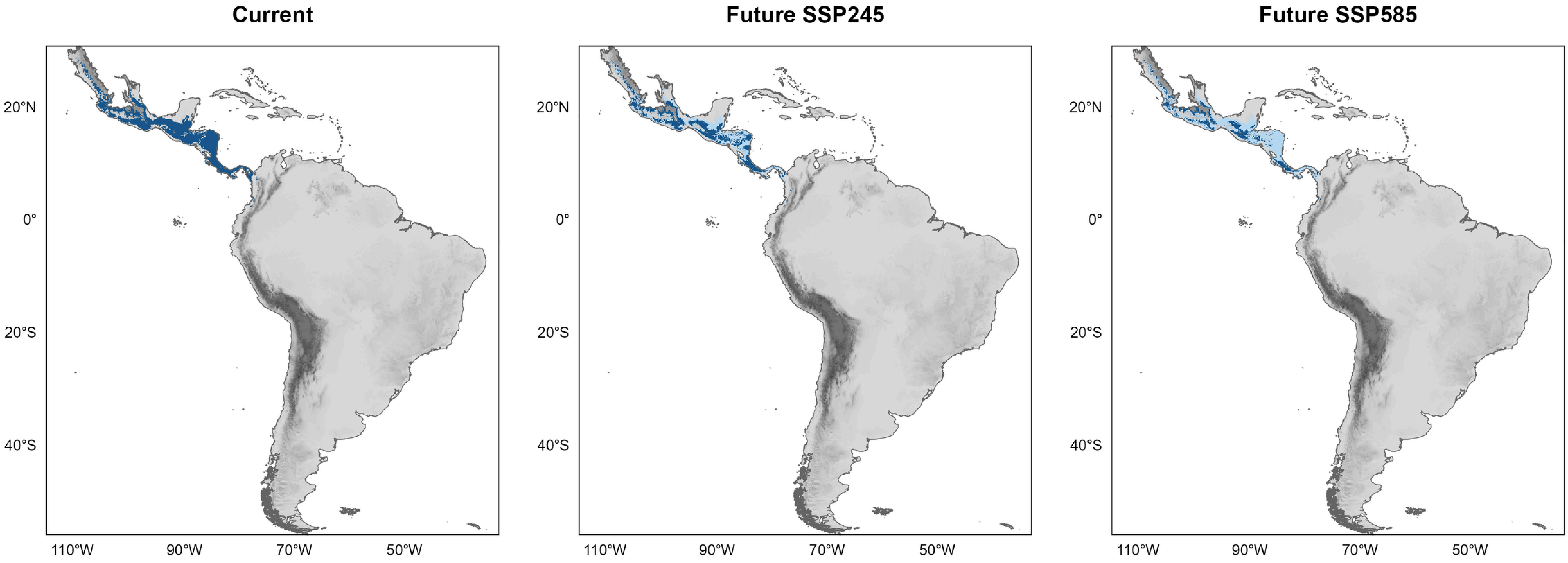

Figure 68

Fig. 68. Potential distribution models for *Hylonycteris underwoodi* under current and future climate scenarios. Maps display predicted habitat suitability across its range under current conditions (left), moderate emissions scenario SSP245 (middle), and high emissions scenario SSP585 (right) projected to 2070. Darker blue indicates higher probability of suitable habitat. The transition from dark to light blue represents progressive loss of habitat suitability, with areas of highest suitability (dark blue) diminishing substantially under more severe emission scenarios.

Lophostoma silvicola

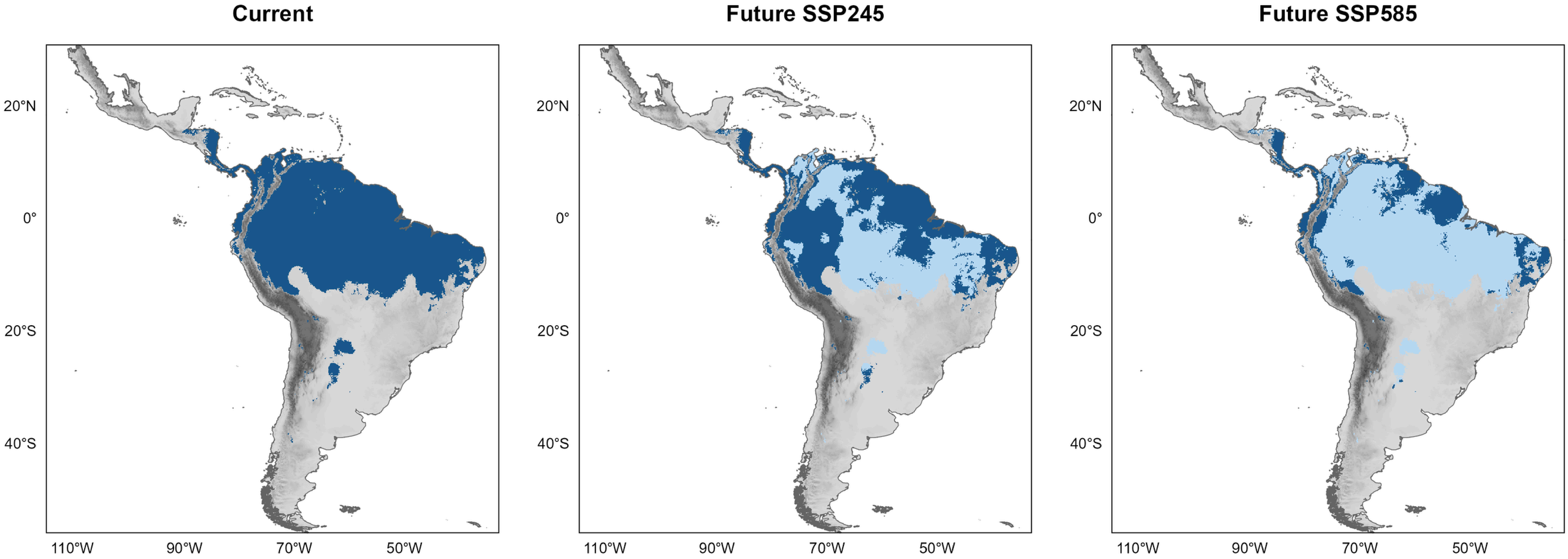

Figure 69

Fig. 69. Potential distribution models for *Lophostoma silvicola* under current and future climate scenarios. Maps display predicted habitat suitability across its range under current conditions (left), moderate emissions scenario SSP245 (middle), and high emissions scenario SSP585 (right) projected to 2070. Darker blue indicates higher probability of suitable habitat. The transition from dark to light blue represents progressive loss of habitat suitability, with areas of highest suitability (dark blue) diminishing substantially under more severe emission scenarios.

Macrophyllum macrophyllum

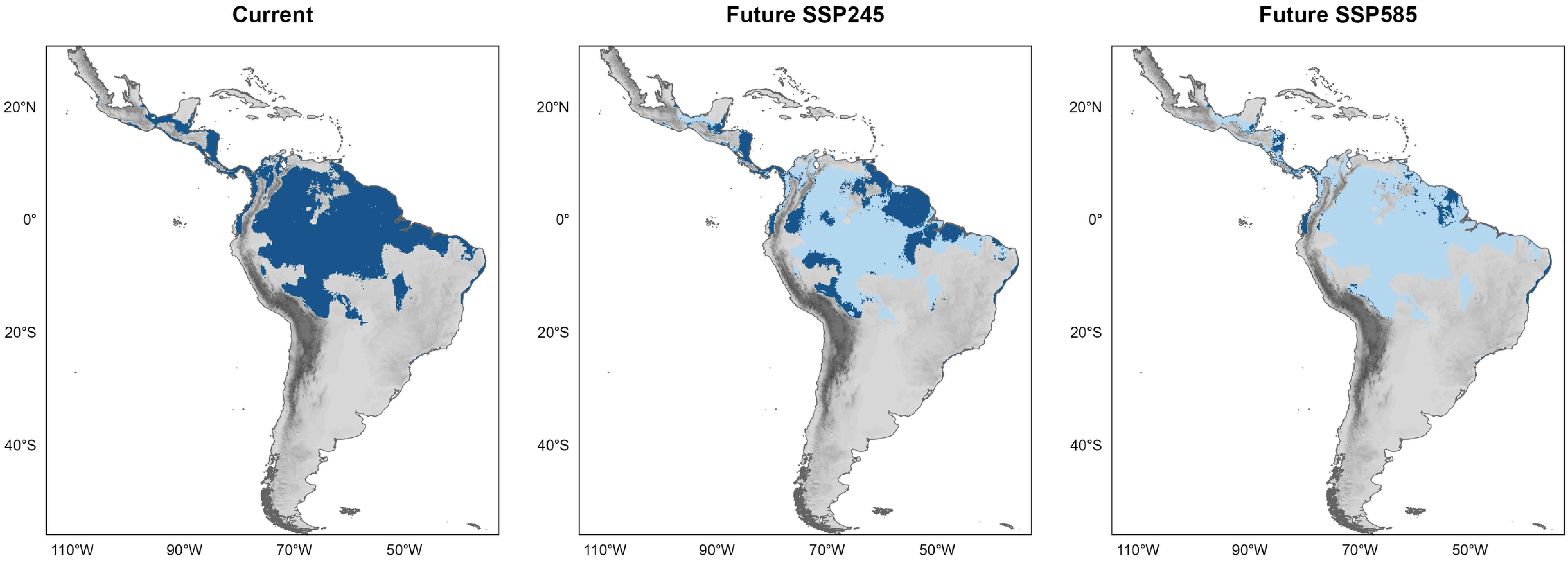

Figure 70

Fig. 70. Potential distribution models for *Macrophyllum macrophyllum* under current and future climate scenarios. Maps display predicted habitat suitability across its range under current conditions (left), moderate emissions scenario SSP245 (middle), and high emissions scenario SSP585 (right) projected to 2070. Darker blue indicates higher probability of suitable habitat. The transition from dark to light blue represents progressive loss of habitat suitability, with areas of highest suitability (dark blue) diminishing substantially under more severe emission scenarios.

Phylloderma stenops

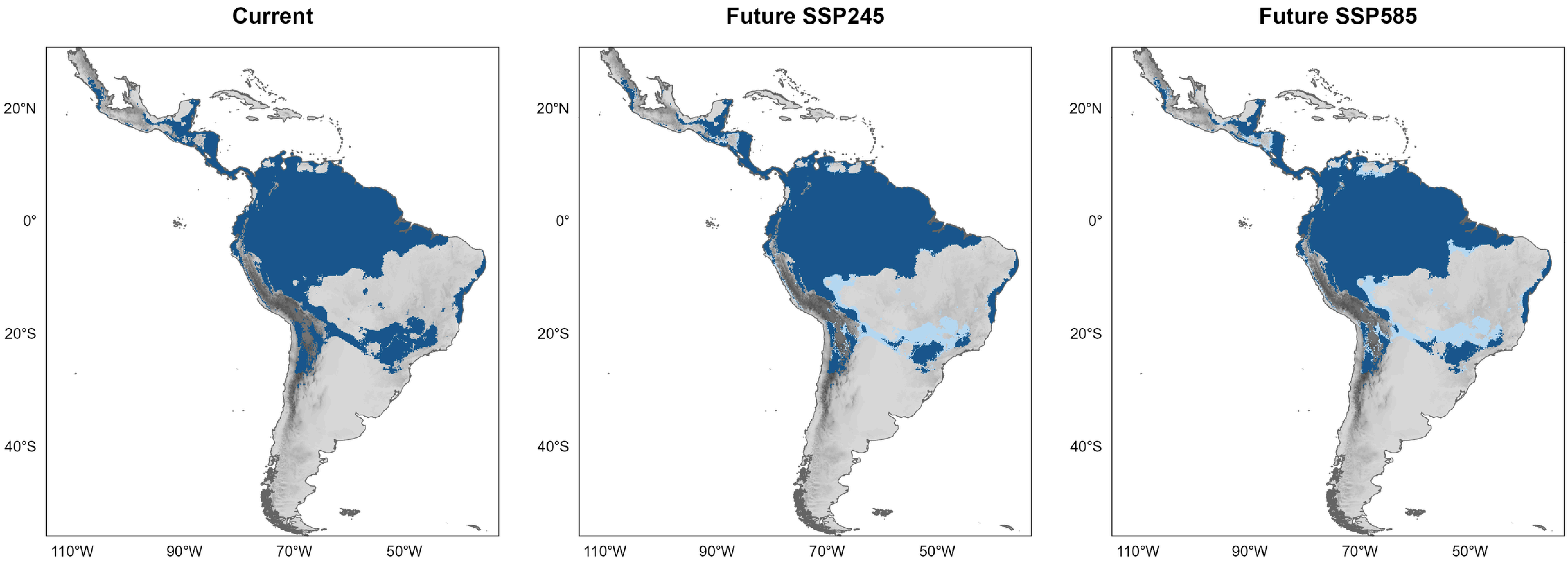

Figure 71

Fig. 71. Potential distribution models for *Phylloderma stenops* under current and future climate scenarios. Maps display predicted habitat suitability across its range under current conditions (left), moderate emissions scenario SSP245 (middle), and high emissions scenario SSP585 (right) projected to 2070. Darker blue indicates higher probability of suitable habitat. The transition from dark to light blue represents progressive loss of habitat suitability, with areas of highest suitability (dark blue) diminishing substantially under more severe emission scenarios.

Phyllostomus hastatus

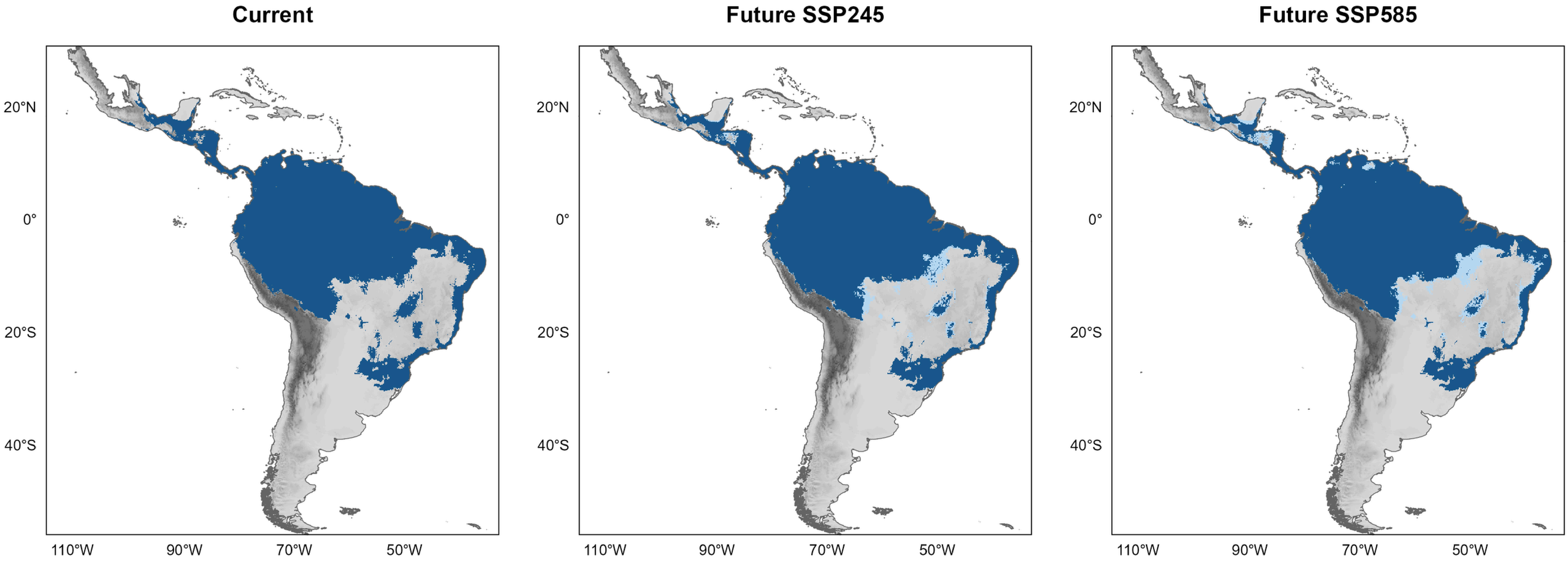

Figure 72

Fig. 72. Potential distribution models for *Phyllostomus hastatus* under current and future climate scenarios. Maps display predicted habitat suitability across its range under current conditions (left), moderate emissions scenario SSP245 (middle), and high emissions scenario SSP585 (right) projected to 2070. Darker blue indicates higher probability of suitable habitat. The transition from dark to light blue represents progressive loss of habitat suitability, with areas of highest suitability (dark blue) diminishing substantially under more severe emission scenarios.

Tonatia maresi

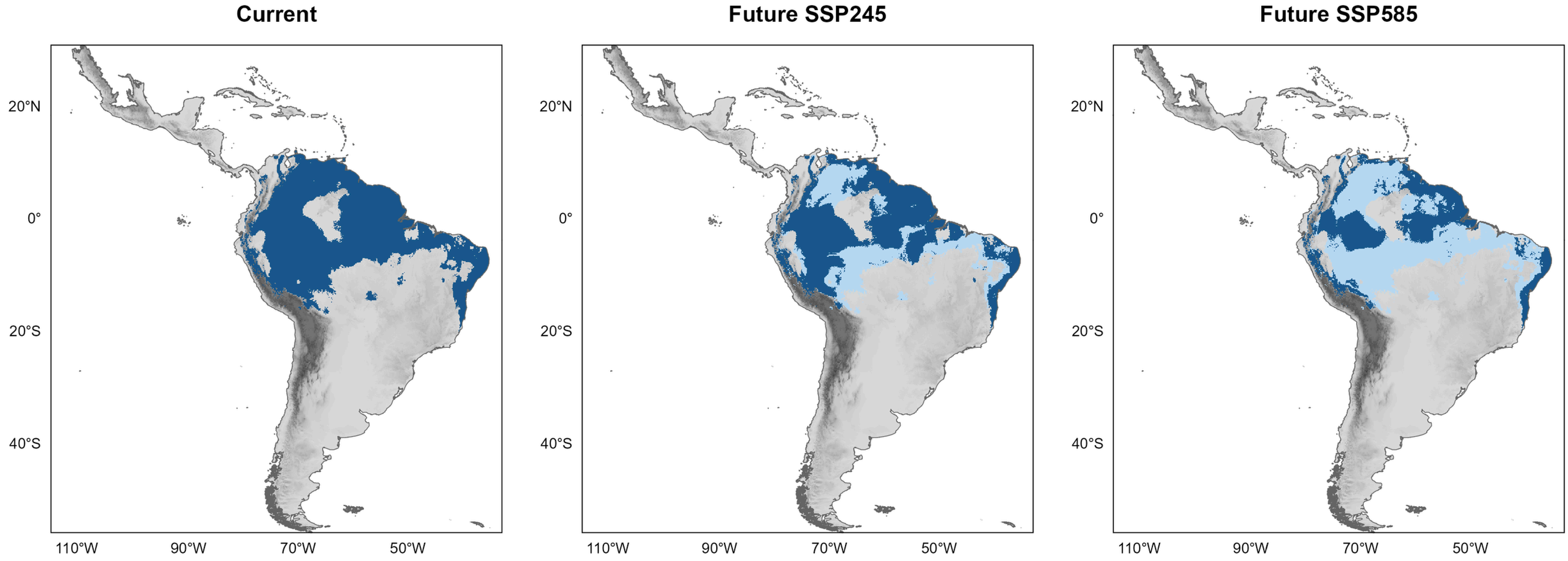

Figure 73

Fig. 73. Potential distribution models for *Tonatia maresi* under current and future climate scenarios. Maps display predicted habitat suitability across its range under current conditions (left), moderate emissions scenario SSP245 (middle), and high emissions scenario SSP585 (right) projected to 2070. Darker blue indicates higher probability of suitable habitat. The transition from dark to light blue represents progressive loss of habitat suitability, with areas of highest suitability (dark blue) diminishing substantially under more severe emission scenarios.

Trachops cirrhosus

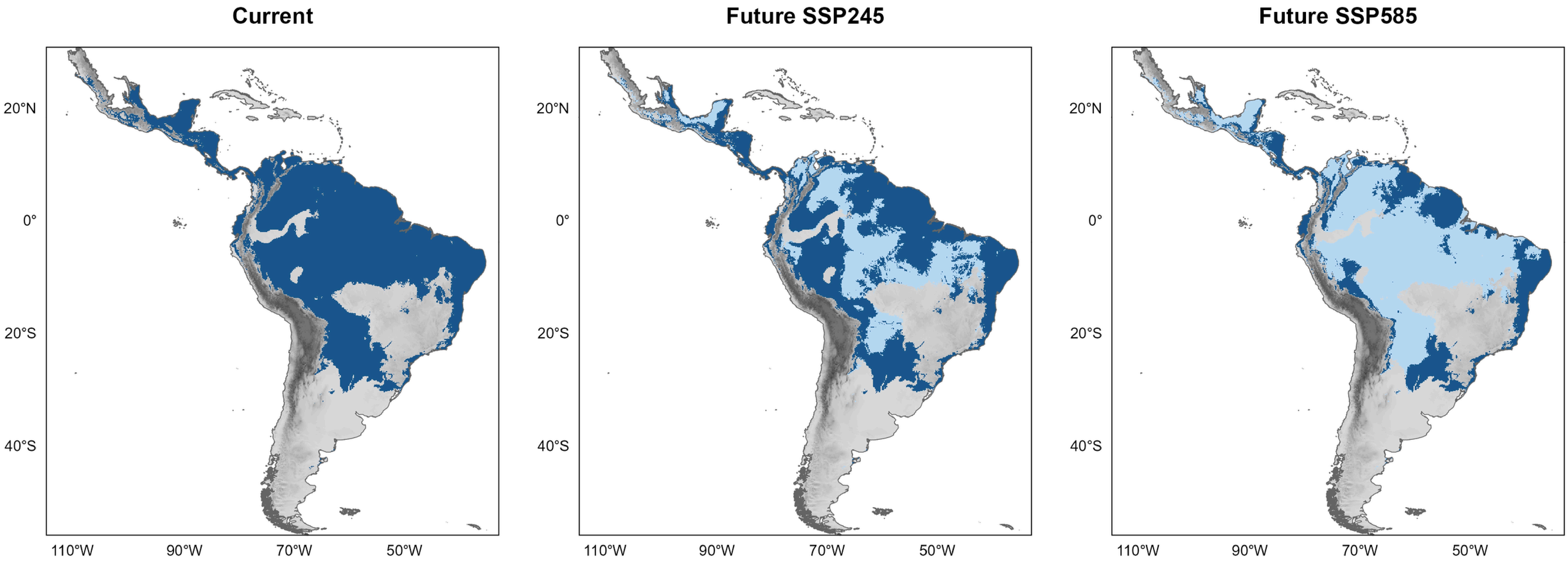

Figure 74

Fig. 74. Potential distribution models for *Trachops cirrhosus* under current and future climate scenarios. Maps display predicted habitat suitability across its range under current conditions (left), moderate emissions scenario SSP245 (middle), and high emissions scenario SSP585 (right) projected to 2070. Darker blue indicates higher probability of suitable habitat. The transition from dark to light blue represents progressive loss of habitat suitability, with areas of highest suitability (dark blue) diminishing substantially under more severe emission scenarios.

Trinycteris nicefori

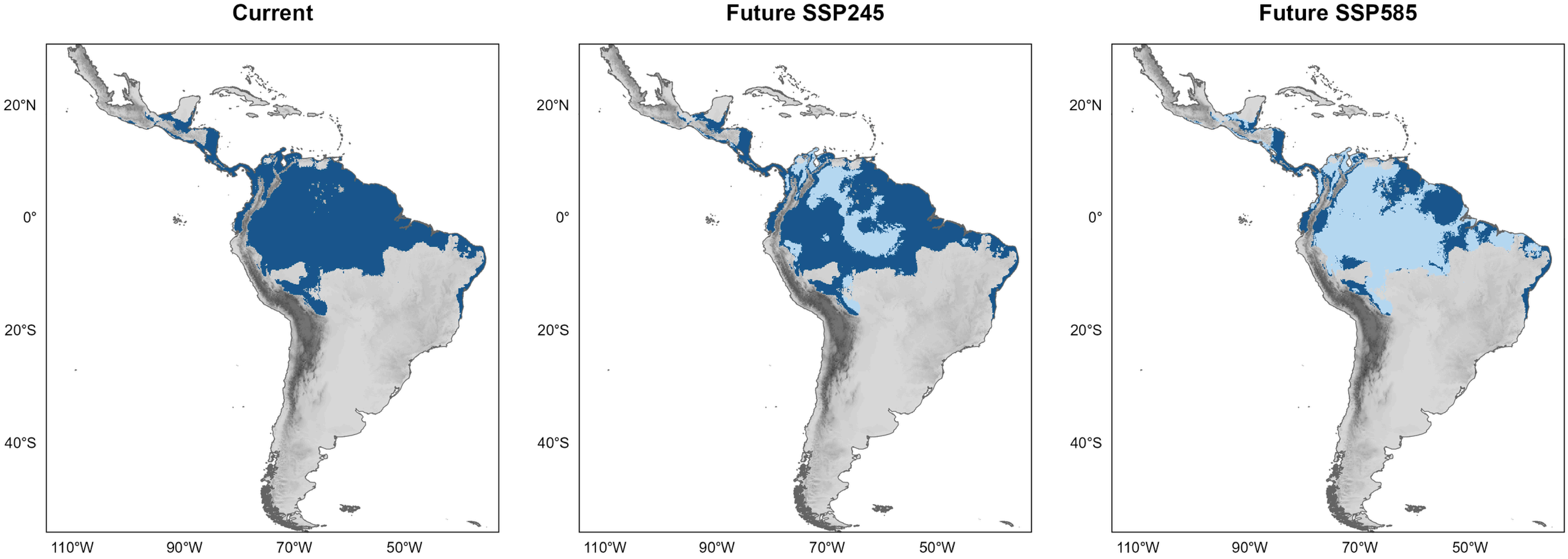

Figure 75

Fig. 75. Potential distribution models for *Trinycteris nicefori* under current and future climate scenarios. Maps display predicted habitat suitability across its range under current conditions (left), moderate emissions scenario SSP245 (middle), and high emissions scenario SSP585 (right) projected to 2070. Darker blue indicates higher probability of suitable habitat. The transition from dark to light blue represents progressive loss of habitat suitability, with areas of highest suitability (dark blue) diminishing substantially under more severe emission scenarios.

Histiotus montanus

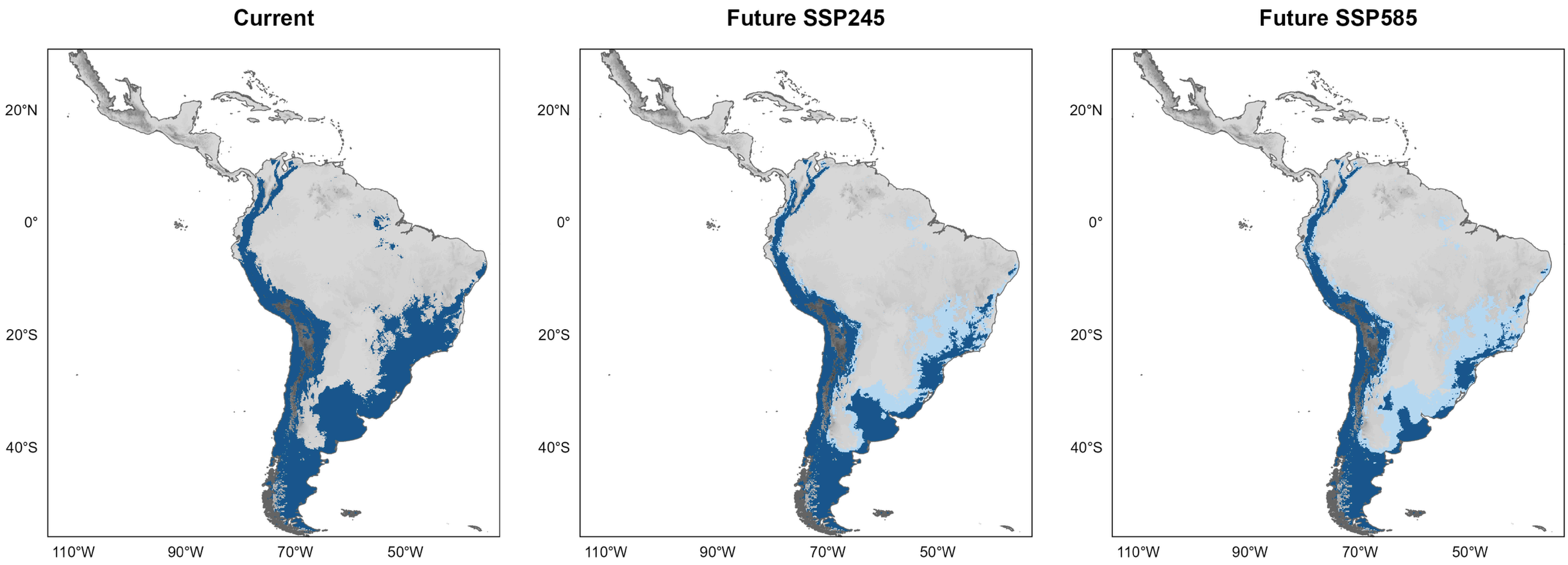

Figure 76

Fig. 76. Potential distribution models for *Histiotus montanus* under current and future climate scenarios. Maps display predicted habitat suitability across its range under current conditions (left), moderate emissions scenario SSP245 (middle), and high emissions scenario SSP585 (right) projected to 2070. Darker blue indicates higher probability of suitable habitat. The transition from dark to light blue represents progressive loss of habitat suitability, with areas of highest suitability (dark blue) diminishing substantially under more severe emission scenarios.

Histiotus velatus

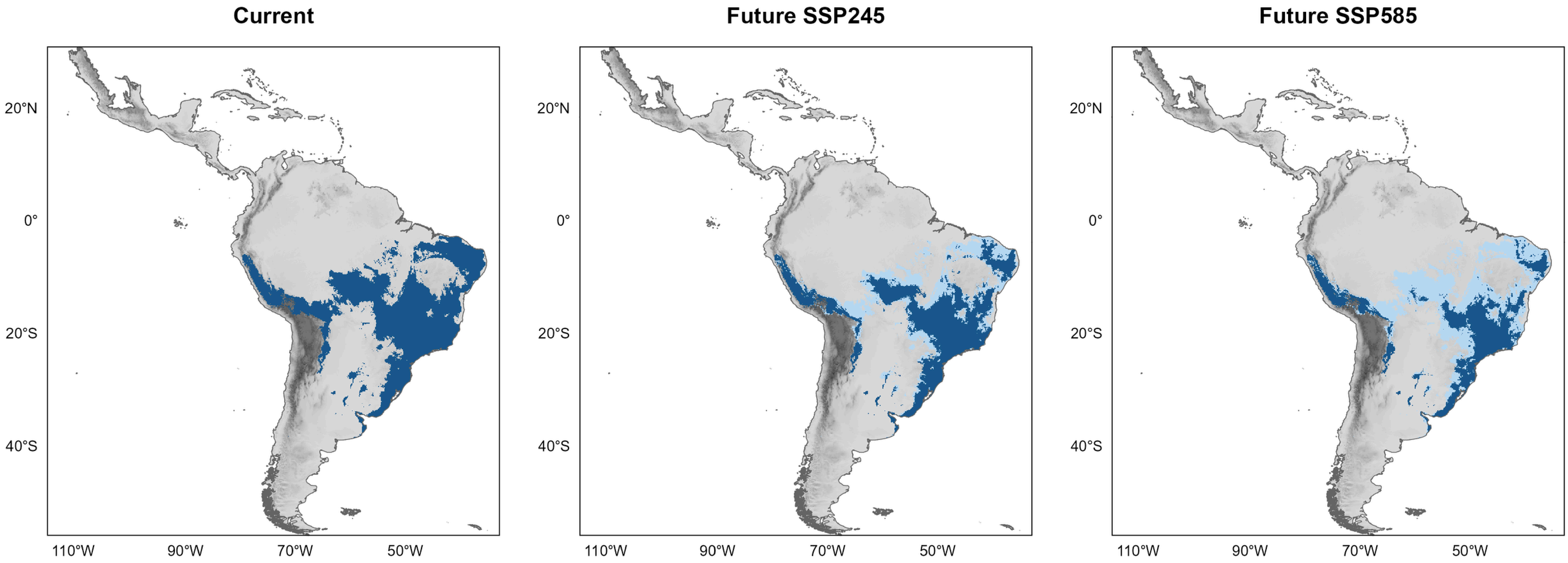

Figure 77

Fig. 77. Potential distribution models for *Histiotus velatus* under current and future climate scenarios. Maps display predicted habitat suitability across its range under current conditions (left), moderate emissions scenario SSP245 (middle), and high emissions scenario SSP585 (right) projected to 2070. Darker blue indicates higher probability of suitable habitat. The transition from dark to light blue represents progressive loss of habitat suitability, with areas of highest suitability (dark blue) diminishing substantially under more severe emission scenarios.

Lasiurus ega

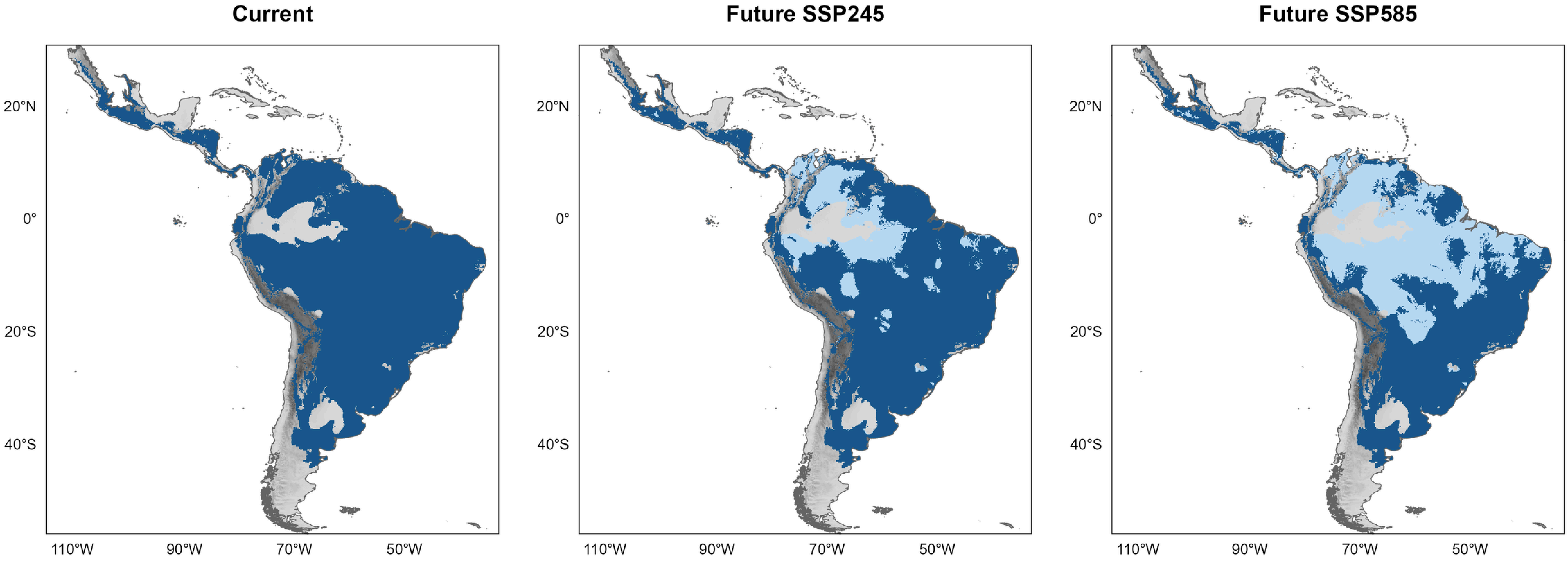

Figure 78

Fig. 78. Potential distribution models for *Lasiurus ega* under current and future climate scenarios. Maps display predicted habitat suitability across its range under current conditions (left), moderate emissions scenario SSP245 (middle), and high emissions scenario SSP585 (right) projected to 2070. Darker blue indicates higher probability of suitable habitat. The transition from dark to light blue represents progressive loss of habitat suitability, with areas of highest suitability (dark blue) diminishing substantially under more severe emission scenarios.

Myotis nigricans

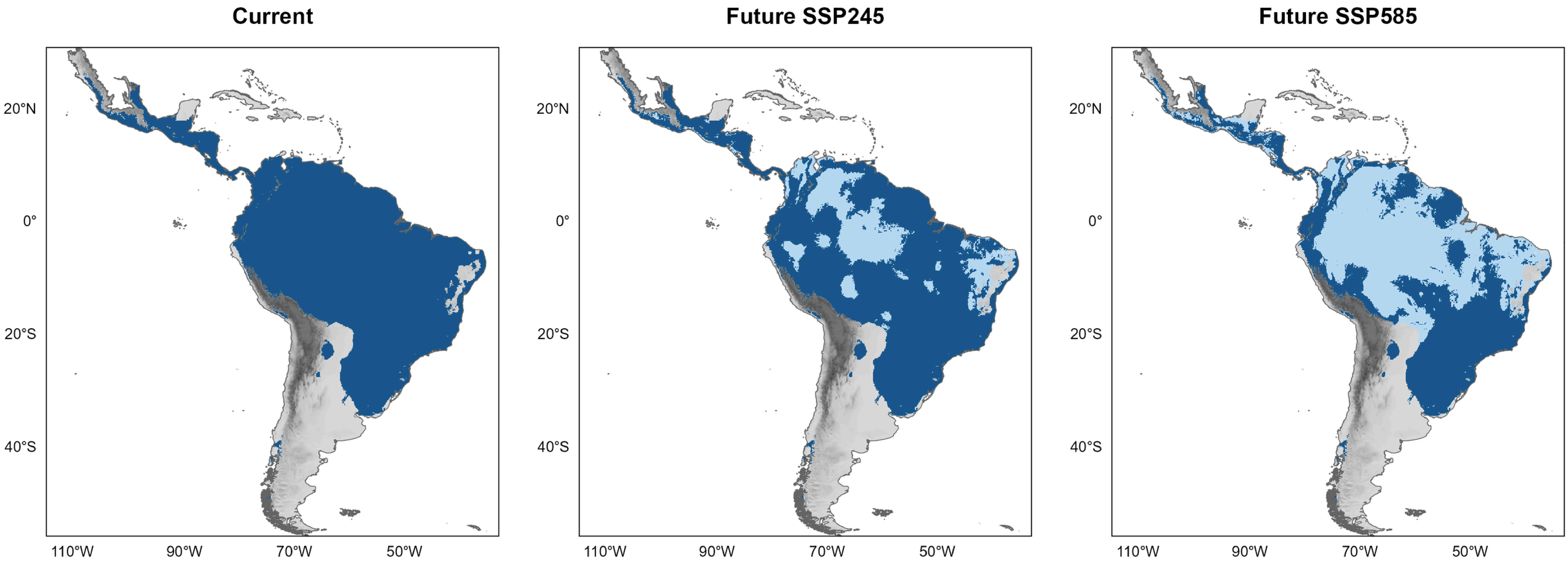

Figure 79

Fig. 79. Potential distribution models for *Myotis nigricans* under current and future climate scenarios. Maps display predicted habitat suitability across its range under current conditions (left), moderate emissions scenario SSP245 (middle), and high emissions scenario SSP585 (right) projected to 2070. Darker blue indicates higher probability of suitable habitat. The transition from dark to light blue represents progressive loss of habitat suitability, with areas of highest suitability (dark blue) diminishing substantially under more severe emission scenarios.

Myotis riparius

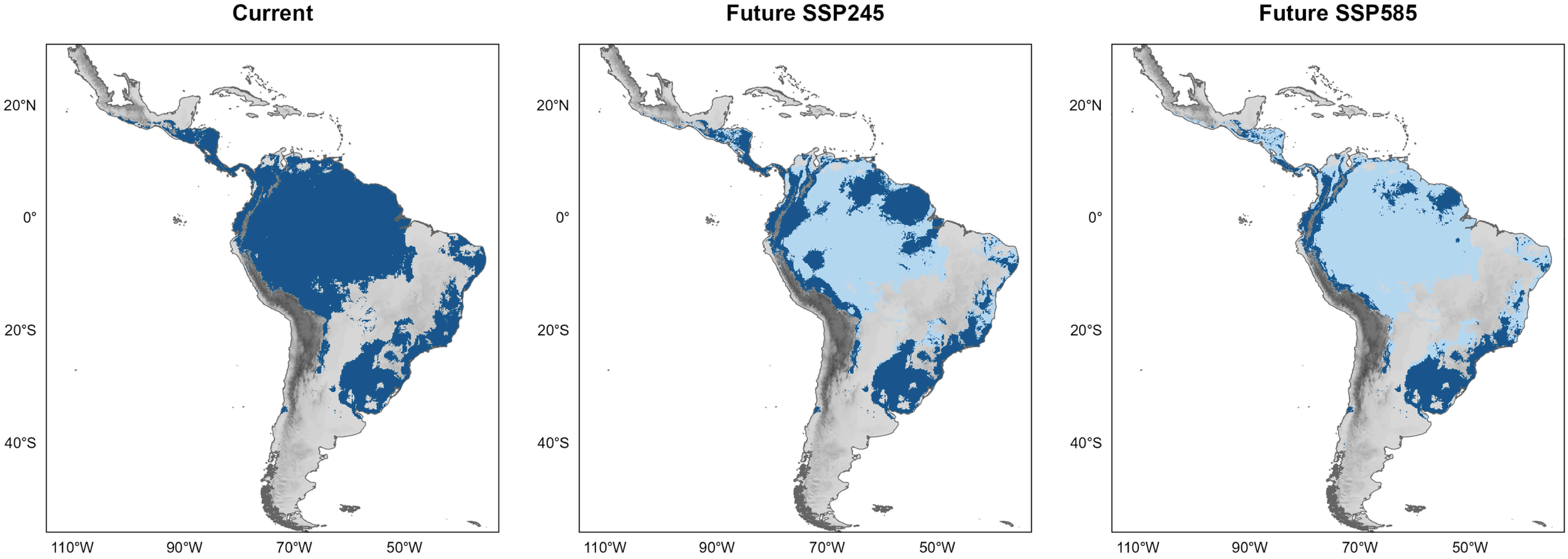

Figure 80

Fig. 80. Potential distribution models for *Myotis riparius* under current and future climate scenarios. Maps display predicted habitat suitability across its range under current conditions (left), moderate emissions scenario SSP245 (middle), and high emissions scenario SSP585 (right) projected to 2070. Darker blue indicates higher probability of suitable habitat. The transition from dark to light blue represents progressive loss of habitat suitability, with areas of highest suitability (dark blue) diminishing substantially under more severe emission scenarios.

Neoeptesicus brasiliensis

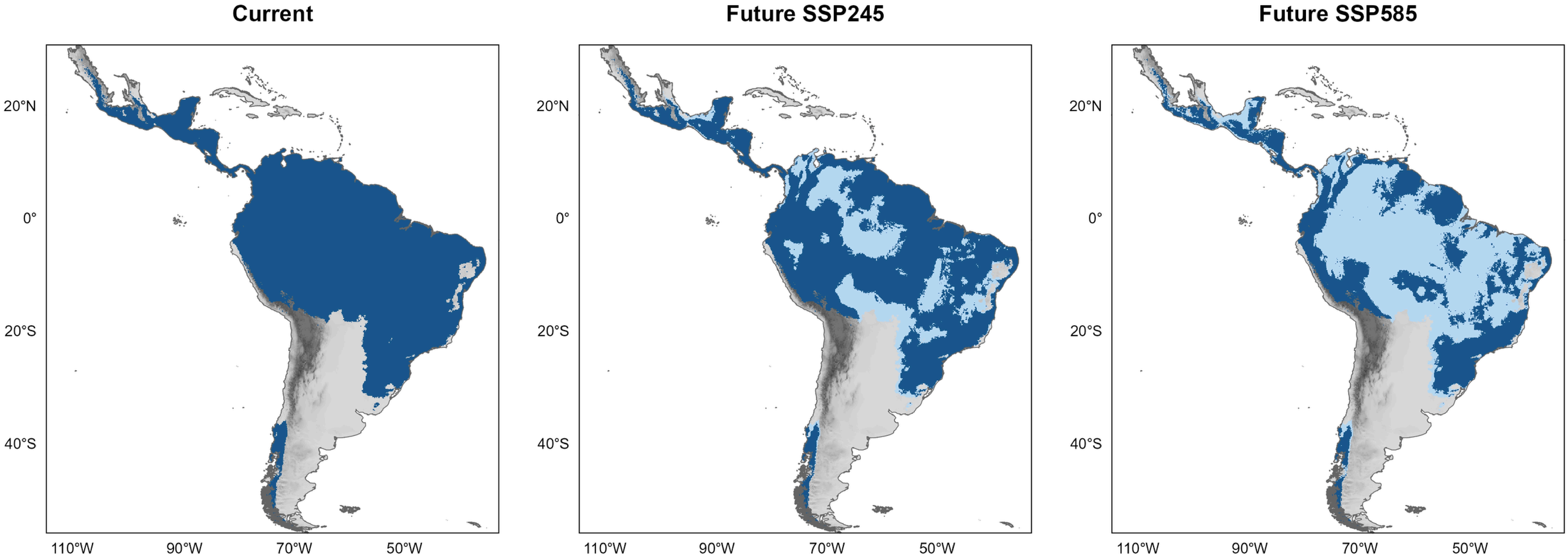

Figure 81

Fig. 81. Potential distribution models for *Neoeptesicus brasiliensis* under current and future climate scenarios. Maps display predicted habitat suitability across its range under current conditions (left), moderate emissions scenario SSP245 (middle), and high emissions scenario SSP585 (right) projected to 2070. Darker blue indicates higher probability of suitable habitat. The transition from dark to light blue represents progressive loss of habitat suitability, with areas of highest suitability (dark blue) diminishing substantially under more severe emission scenarios.

Neoeptesicus chiriquinus

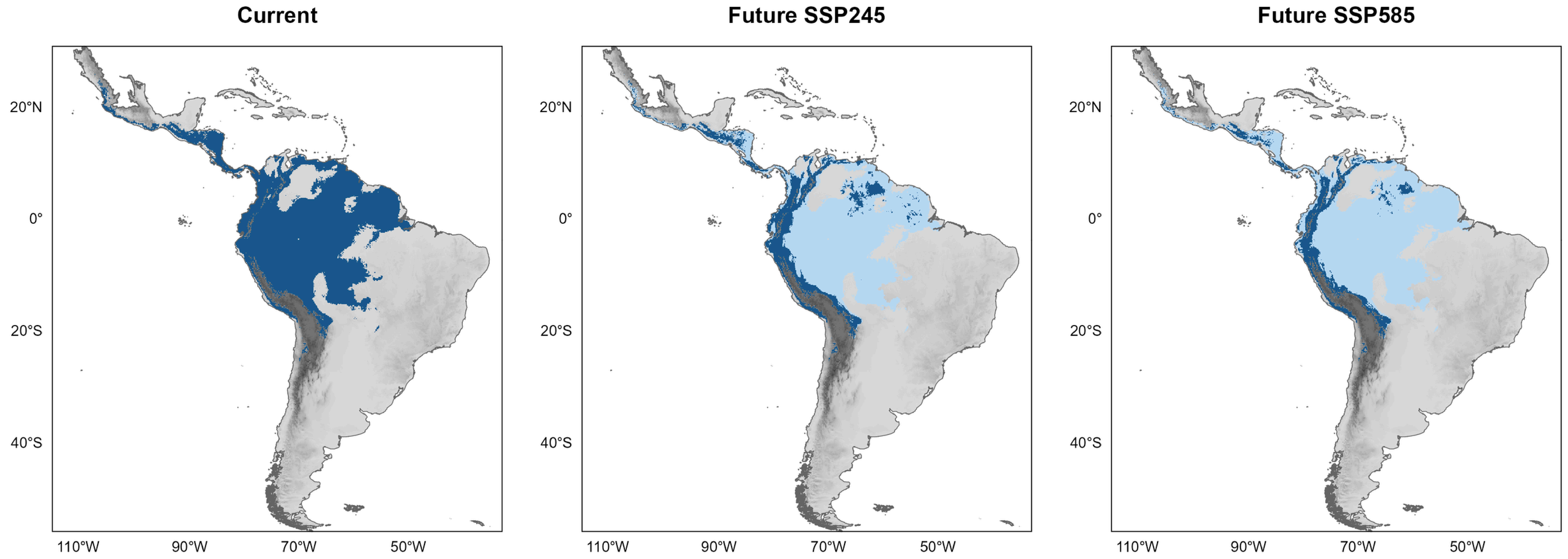

Figure 82

Fig. 82. Potential distribution models for *Neoeptesicus chiriquinus* under current and future climate scenarios. Maps display predicted habitat suitability across its range under current conditions (left), moderate emissions scenario SSP245 (middle), and high emissions scenario SSP585 (right) projected to 2070. Darker blue indicates higher probability of suitable habitat. The transition from dark to light blue represents progressive loss of habitat suitability, with areas of highest suitability (dark blue) diminishing substantially under more severe emission scenarios.

Neoeptesicus furinalis

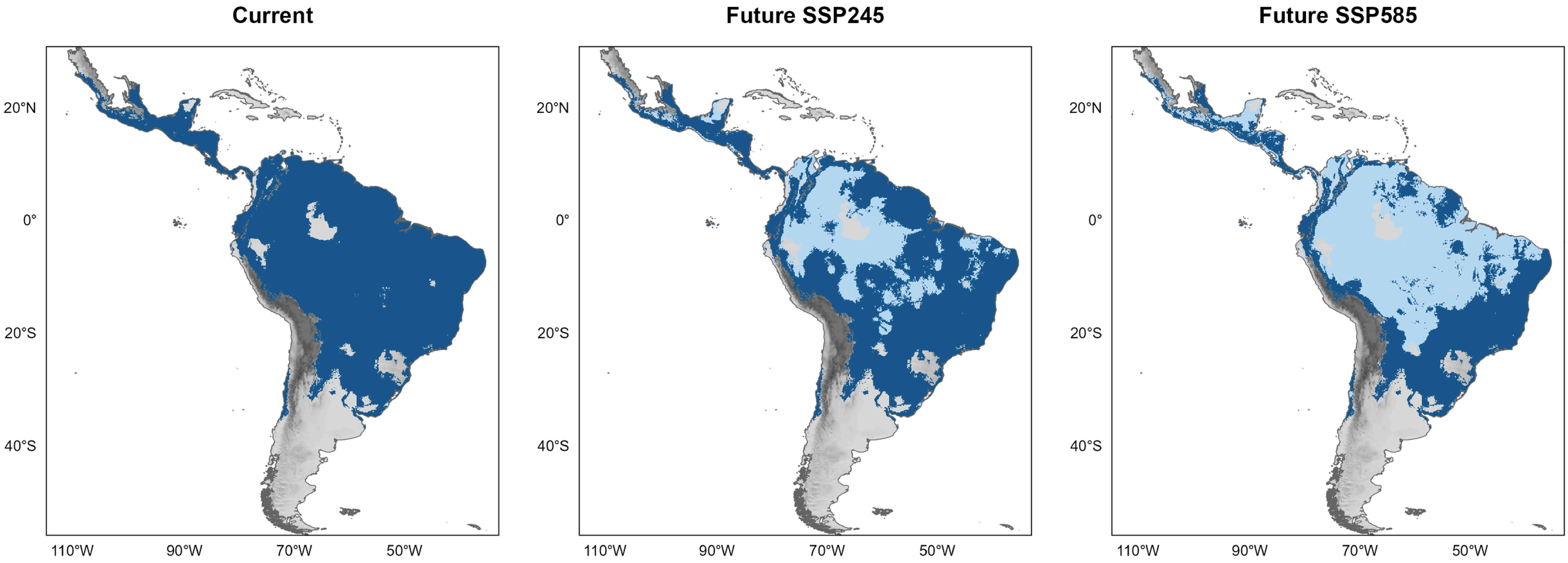

Figure 83

Fig. 83. Potential distribution models for *Neoeptesicus furinalis* under current and future climate scenarios. Maps display predicted habitat suitability across its range under current conditions (left), moderate emissions scenario SSP245 (middle), and high emissions scenario SSP585 (right) projected to 2070. Darker blue indicates higher probability of suitable habitat. The transition from dark to light blue represents progressive loss of habitat suitability, with areas of highest suitability (dark blue) diminishing substantially under more severe emission scenarios.

Pteronotus personatus

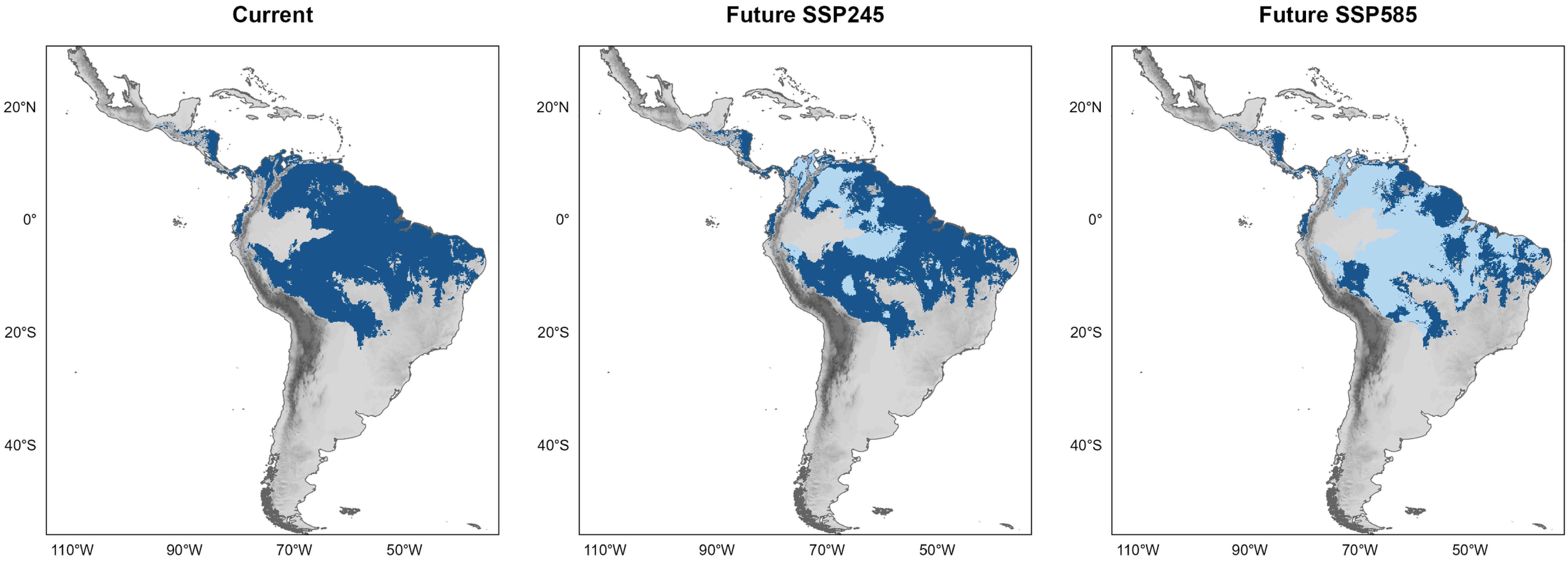

Figure 84

Fig. 84. Potential distribution models for *Pteronotus personatus* under current and future climate scenarios. Maps display predicted habitat suitability across its range under current conditions (left), moderate emissions scenario SSP245 (middle), and high emissions scenario SSP585 (right) projected to 2070. Darker blue indicates higher probability of suitable habitat. The transition from dark to light blue represents progressive loss of habitat suitability, with areas of highest suitability (dark blue) diminishing substantially under more severe emission scenarios.

Eumops hansae

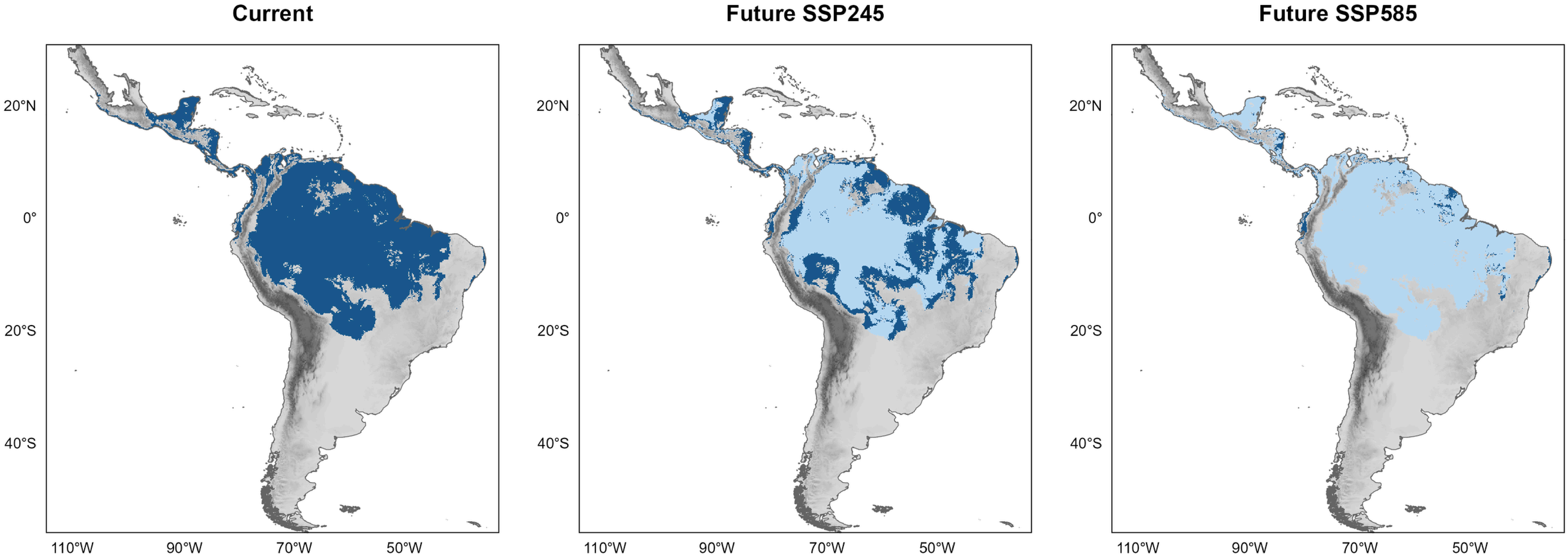

Figure 85

Fig. 85. Potential distribution models for *Eumops hansae* under current and future climate scenarios. Maps display predicted habitat suitability across its range under current conditions (left), moderate emissions scenario SSP245 (middle), and high emissions scenario SSP585 (right) projected to 2070. Darker blue indicates higher probability of suitable habitat. The transition from dark to light blue represents progressive loss of habitat suitability, with areas of highest suitability (dark blue) diminishing substantially under more severe emission scenarios.

Nyctinomops laticaudatus

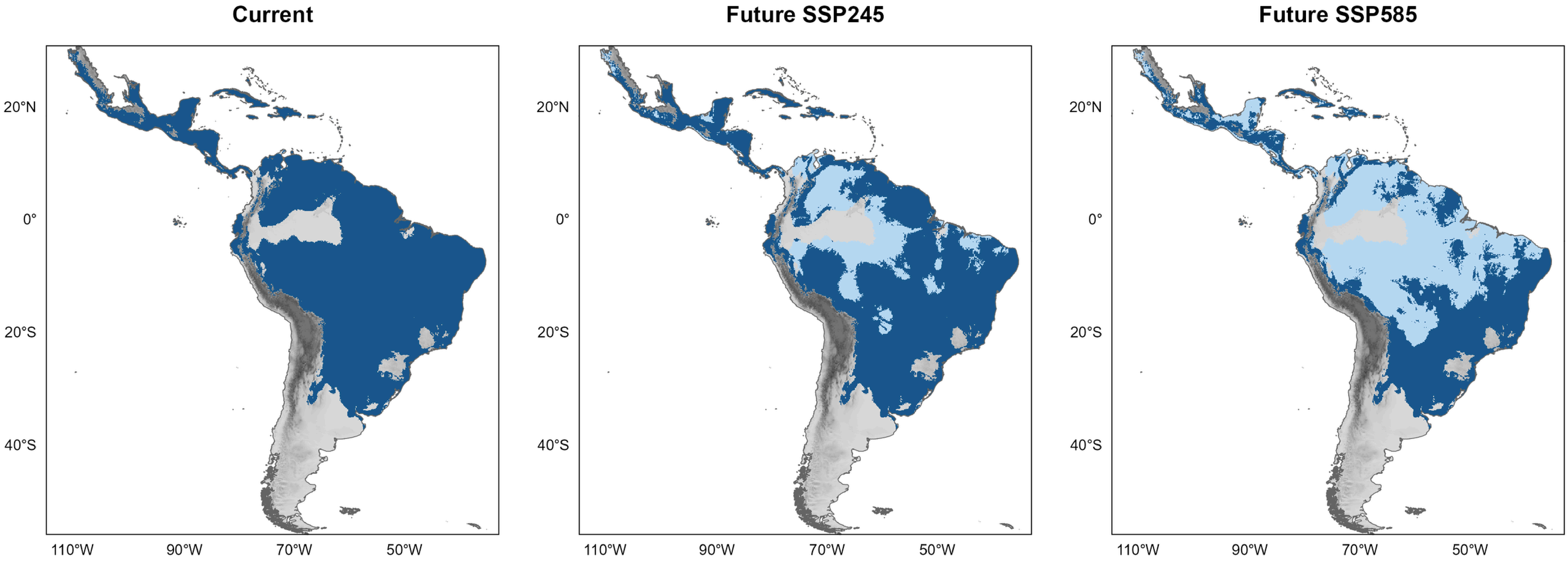

Figure 86

Fig. 86. Potential distribution models for *Nyctinomops laticaudatus* under current and future climate scenarios. Maps display predicted habitat suitability across its range under current conditions (left), moderate emissions scenario SSP245 (middle), and high emissions scenario SSP585 (right) projected to 2070. Darker blue indicates higher probability of suitable habitat. The transition from dark to light blue represents progressive loss of habitat suitability, with areas of highest suitability (dark blue) diminishing substantially under more severe emission scenarios.

Nyctinomops macrotis

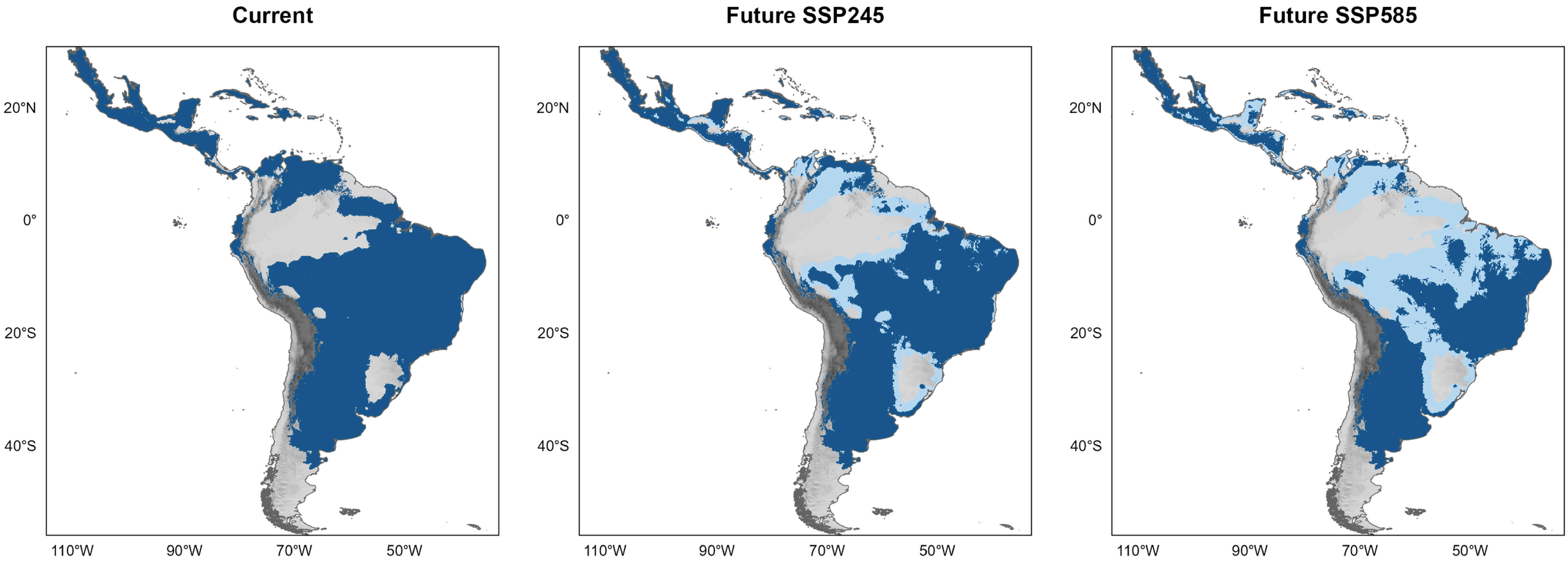

Figure 87

Fig. 87. Potential distribution models for *Nyctinomops macrotis* under current and future climate scenarios. Maps display predicted habitat suitability across its range under current conditions (left), moderate emissions scenario SSP245 (middle), and high emissions scenario SSP585 (right) projected to 2070. Darker blue indicates higher probability of suitable habitat. The transition from dark to light blue represents progressive loss of habitat suitability, with areas of highest suitability (dark blue) diminishing substantially under more severe emission scenarios.

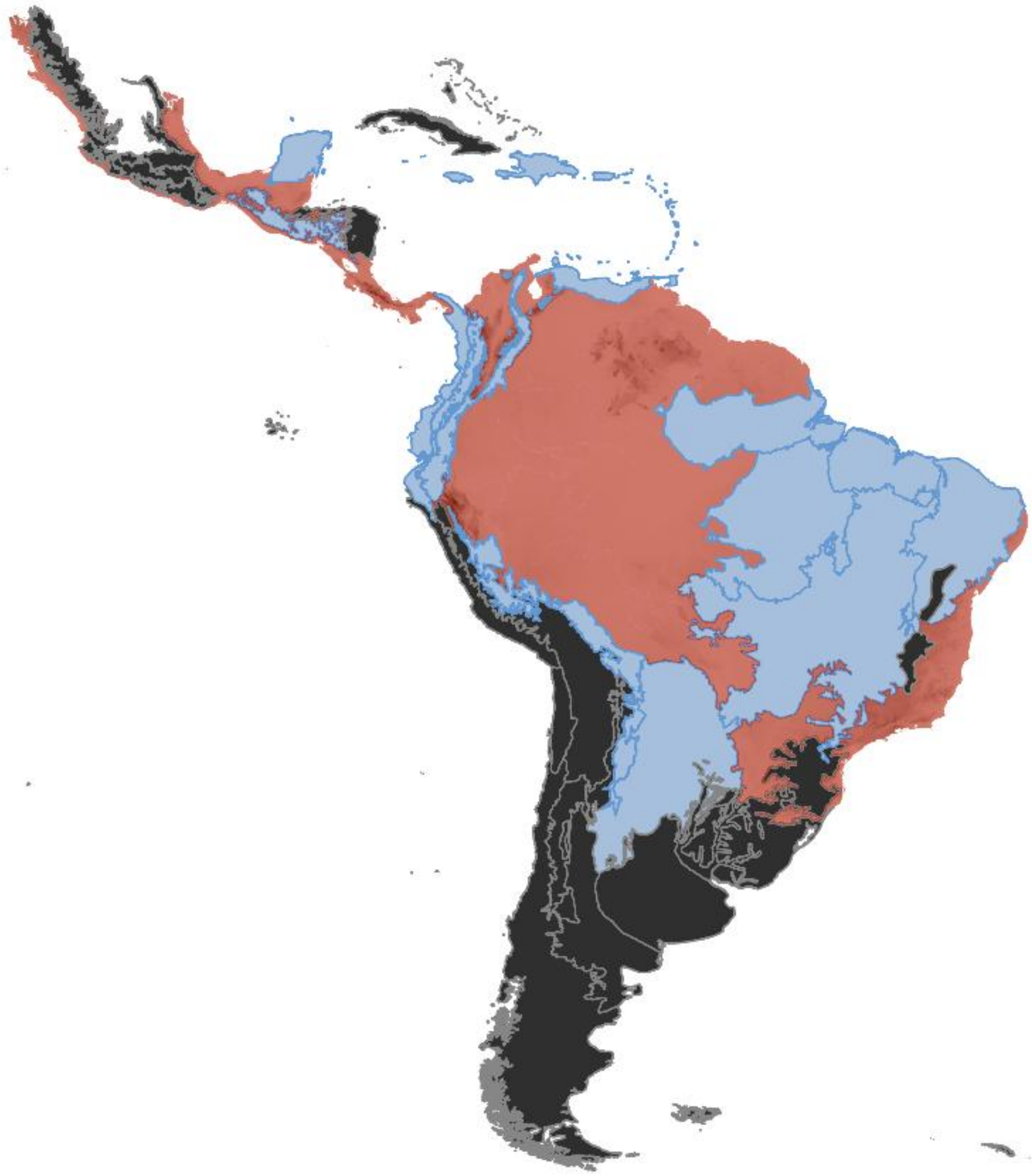

Figure 88. Geographic distribution of bat genetic sampling effort across Latin America. The map illustrates the spatial pattern of genetic data availability for bats, with black regions representing severely under-sampled provinces (< 20 genetic sequences available), blue areas indicating moderate sampling (21-199 sequences), and red (≥ 200 sequences) where sufficient sampling has enabled the identification of hidden species. Note the pronounced sampling bias toward northern South America and Mesoamerica, with extensive data gaps throughout the Southern Cone and Andean regions.

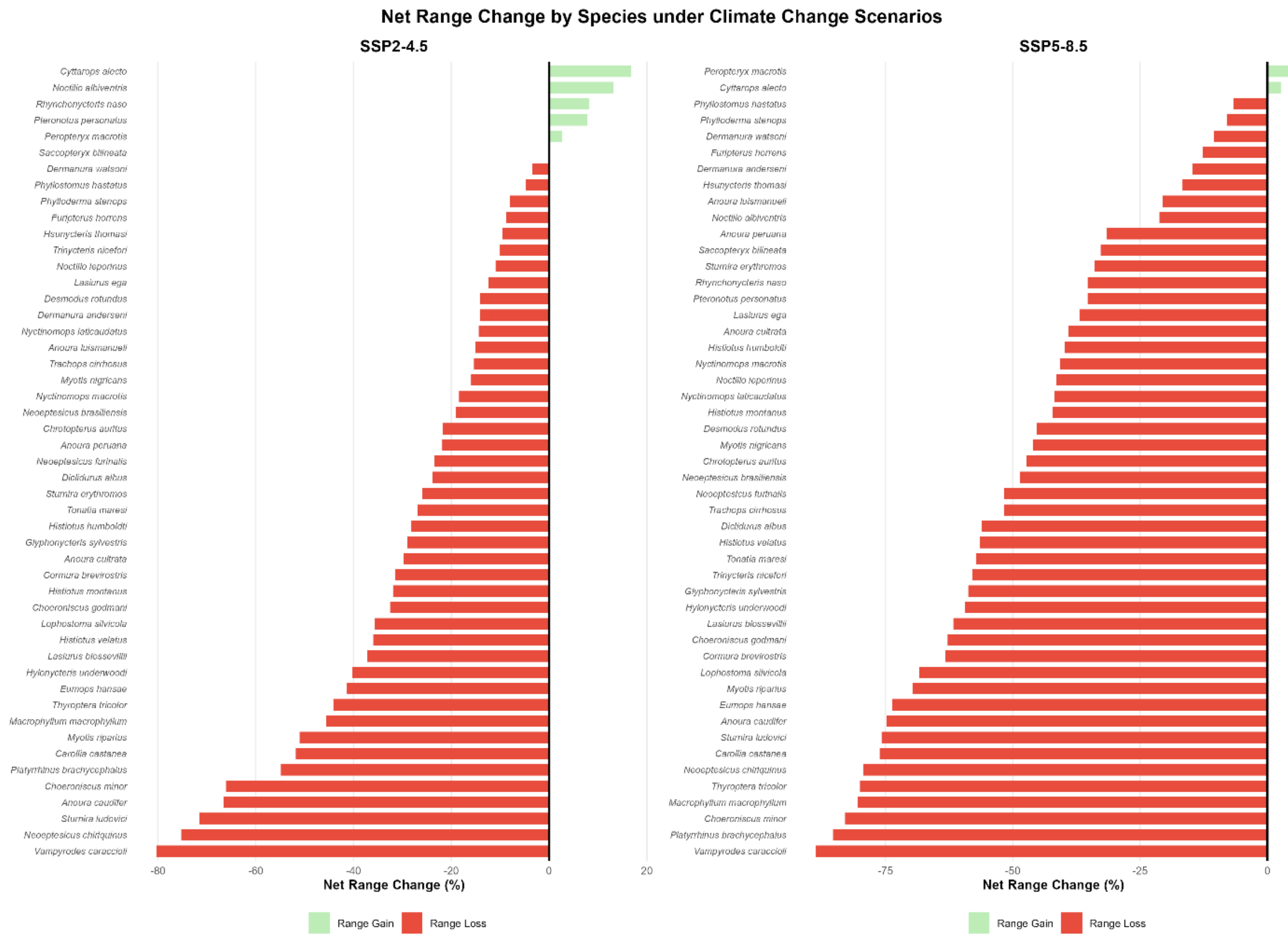
Net Range Change by Species under Climate Change Scenarios
SSP2-4.5
Cyttarops alecto
Noctilio albiventris
Rhynchonycteris naso
Pteronotus personatus
Peropteryx macrotis
Saccopteryx bilineata
Dermanura watsoni
Phyllostomus hastatus
Phylloderma stenops
Furipterus horrens
Hsunycteris thomasi
Trinycteris nicefori
Noctilio leporinus
Lasiurus ega
Desmodus rotundus
Dermanura anderseni
Nyctinomops laticaudatus
Anoura luismanueli
Trachops cirrhosus
Myotis nigricans
Nyctinomops macrotis
Neoeptesicus brasiliensis
Chrotopterus auritus
Anoura peruana
Neoeptesicus furinalis
Diclidurus albus
Sturnira erythromos
Tonatia maresi
Histiotus humboldti
Glyphonycteris sylvestris
Anoura cultrata
Cormura brevirostris
Histiotus montanus
Choeroniscus godmani
Lophostoma silvicola
Histiotus velatus
Lasiurus blossevillii
Hylonycteris underwoodi
Eumops hansae
Thyroptera tricolor
Macrophyllum macrophyllum
Myotis riparius
Carollia castanea
Platyrrhinus brachycephalus
Choeroniscus minor
Anoura caudifer
Sturnira ludovici
Neoeptesicus chiriquinus
Vampyrodes caraccioli
-80
-60
-40
-20
0
20
Net Range Change (%)
Range Gain
Range Loss
SSP5-8.5
Peropteryx macrotis
Cyttarops alecto
Phyllostomus hastatus
Phylloderma stenops
Dermanura watsoni
Furipterus horrens
Dermanura anderseni
Hsunycteris thomasi
Anoura luismanueli
Noctilio albiventris
Anoura peruana
Saccopteryx bilineata
Sturnira erythromos
Rhynchonycteris naso
Pteronotus personatus
Lasiurus ega
Anoura cultrata
Histiotus humboldti
Nyctinomops macrotis
Noctilio leporinus
Nyctinomops laticaudatus
Histiotus montanus
Desmodus rotundus
Myotis nigricans
Chrotopterus auritus
Neoeptesicus brasiliensis
Neoeptesicus furinalis
Trachops cirrhosus
Diclidurus albus
Histiotus velatus
Tonatia maresi
Trinycteris nicefori
Glyphonycteris sylvestris
Hylonycteris underwoodi
Lasiurus blossevillii
Choeroniscus godmani
Cormura brevirostris
Lophostoma silvicola
Myotis riparius
Eumops hansae
Anoura caudifer
Sturnira ludovici
Carollia castanea
Neoeptesicus chiriquinus
Thyroptera tricolor
Macrophyllum macrophyllum
Choeroniscus minor
Platyrrhinus brachycephalus
Vampyrodes caraccioli
-75
-50
-25
0
Net Range Change (%)
Range Gain
Range Loss

Figure 89. Net range change for Neotropical bat species complexes under future climate scenarios. Horizontal bars show the net percentage change in suitable habitat (gain minus loss) for each species complex under SSP245 (left panel) and SSP585 (right panel) climate scenarios for 2070. Green bars indicate species with net range gains (where gains exceed losses), while red bars indicate species with net range losses.

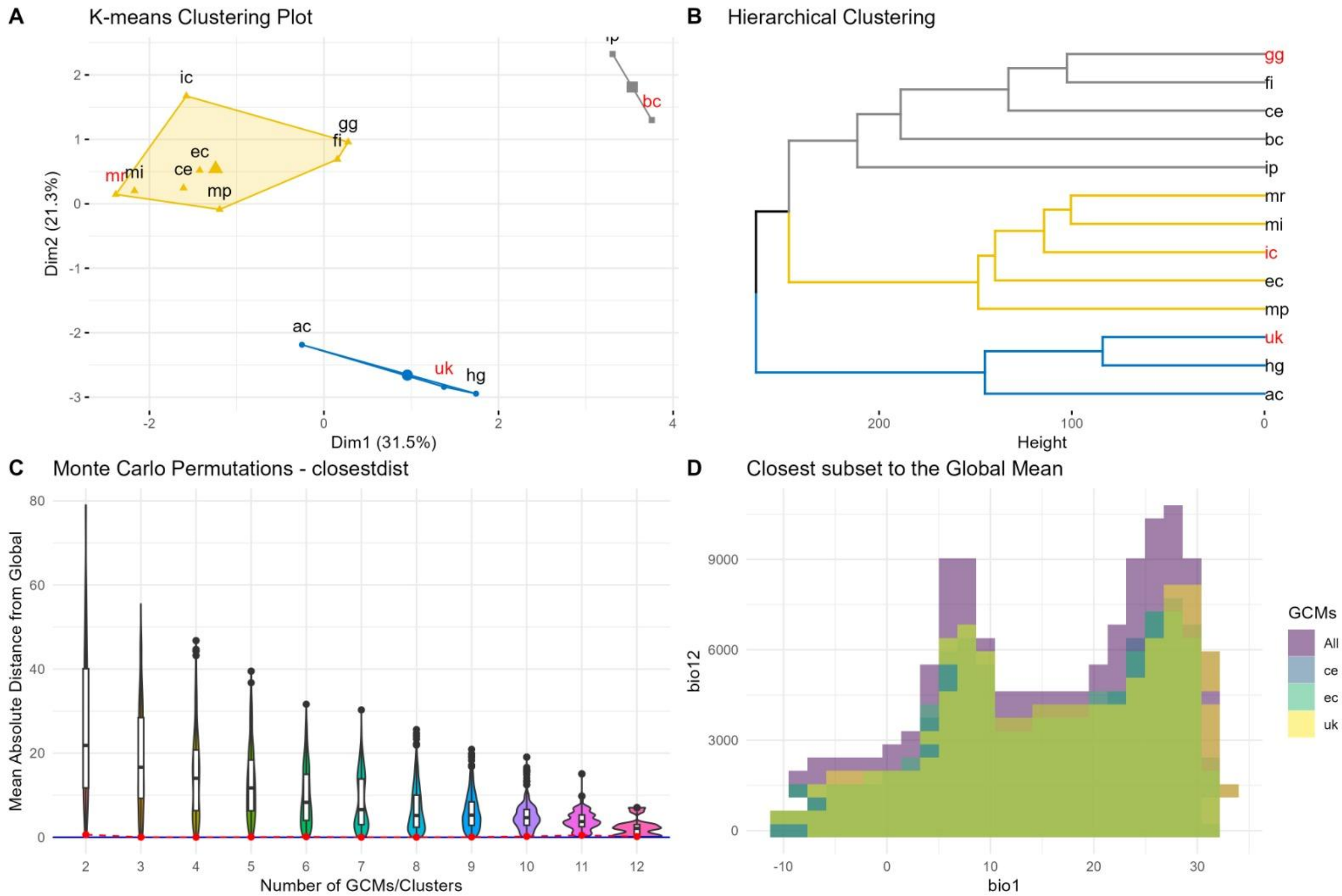

A
K-means Clustering Plot
Dim1 (31.5%)
Dim2 (21.3%)
ic
gg
fi
ec
ce
mr
mi
mp
bc
ac
uk
hg
B
Hierarchical Clustering
gg
fi
ce
bc
ip
mr
mi
ic
ec
mp
uk
hg
ac
Height
C
Monte Carlo Permutations - closestdist
Mean Absolute Distance from Global
Number of GCMs/Clusters
D
Closest subset to the Global Mean
bio12
bio1
GCMs
All
ce
ec
uk

Figure 90. Multi-method approach for selecting optimal Global Circulation Models (GCMs) for climate projections SSP245. The figure demonstrates the statistical clustering of GCMs based on their performance and characteristics: (A) K-means clustering plot showing three distinct clusters (yellow, blue, gray) of GCMs in a two-dimensional space that accounts for 52.8% of variation; (B) Hierarchical clustering dendrogram revealing three main branches corresponding to model families; (C) Monte Carlo permutation analysis of the mean absolute distance from global mean for different numbers of GCMs, showing diminishing returns beyond 9 models; (D) Bivariate distribution plot of climate variables (bio1, bio12) for selected GCMs compared to the ensemble mean. The integrated analysis supports a statistically optimal subset of models for robust climate projections.

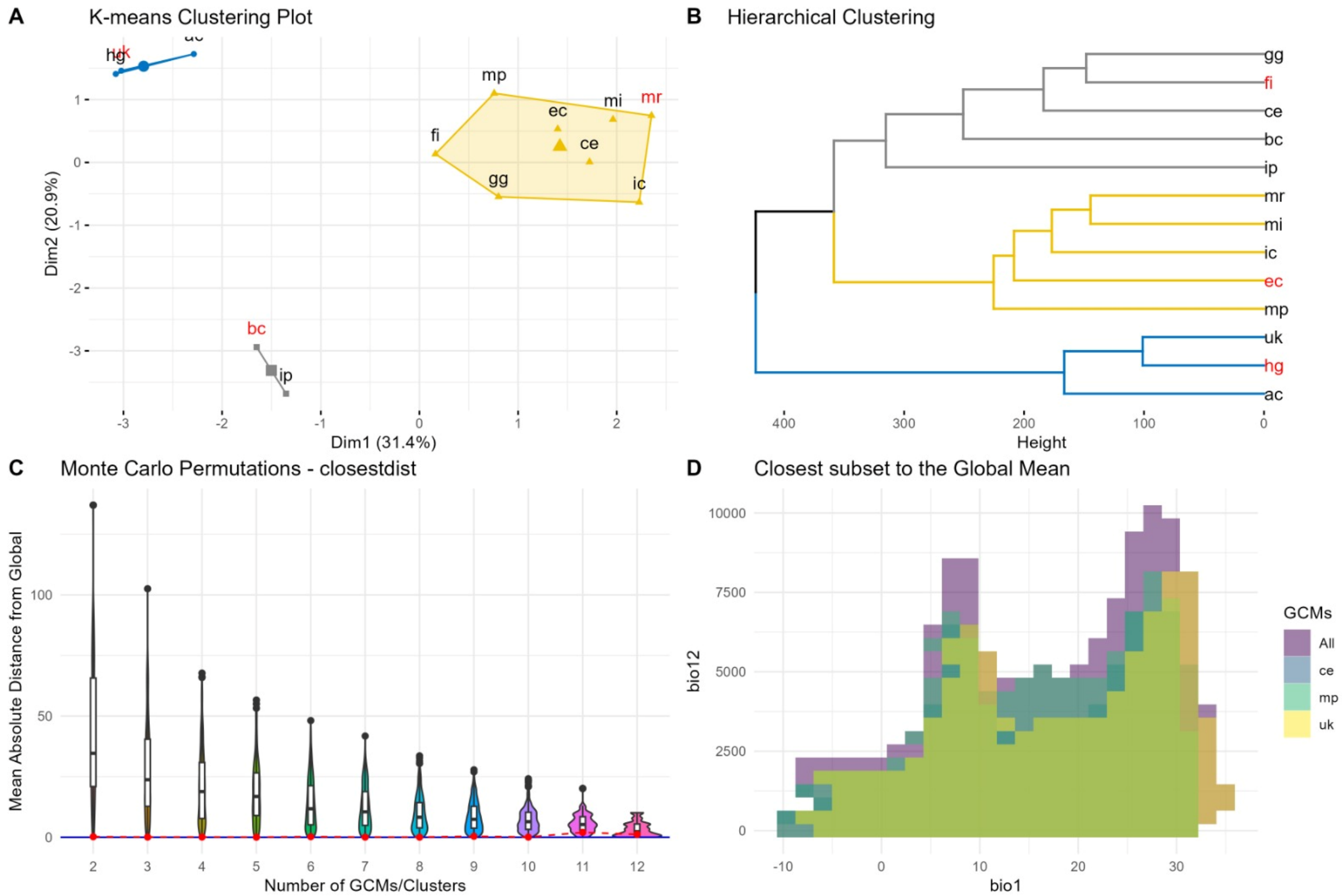
A
K-means Clustering Plot
Dim1 (31.4%)
Dim2 (20.9%)
mp
ec
mi
mr
fi
ce
gg
ic
bc
ip
B
Hierarchical Clustering
gg
fi
ce
bc
ip
mr
mi
ic
ec
mp
uk
hg
ac
Height
C
Monte Carlo Permutations - closestdist
Mean Absolute Distance from Global
Number of GCMs/Clusters
D
Closest subset to the Global Mean
bio12
bio1
GCMs
All
ce
mp
uk

Figure 91. Multi-method approach for selecting optimal Global Circulation Models (GCMs) for climate projections SSP585. The figure demonstrates the statistical clustering of GCMs based on their performance and characteristics: (A) K-means clustering plot showing three distinct clusters (yellow, blue, gray) of GCMs in a two-dimensional space that accounts for 52.3% of variation; (B) Hierarchical clustering dendrogram revealing three main branches corresponding to model families; (C) Monte Carlo permutation analysis of the mean absolute distance from global mean for different numbers of GCMs, showing diminishing returns beyond 9 models; (D) Bivariate distribution plot of climate variables (bio1, bio12) for selected GCMs compared to the ensemble mean. The integrated analysis supports a statistically optimal subset of models for robust climate projections.

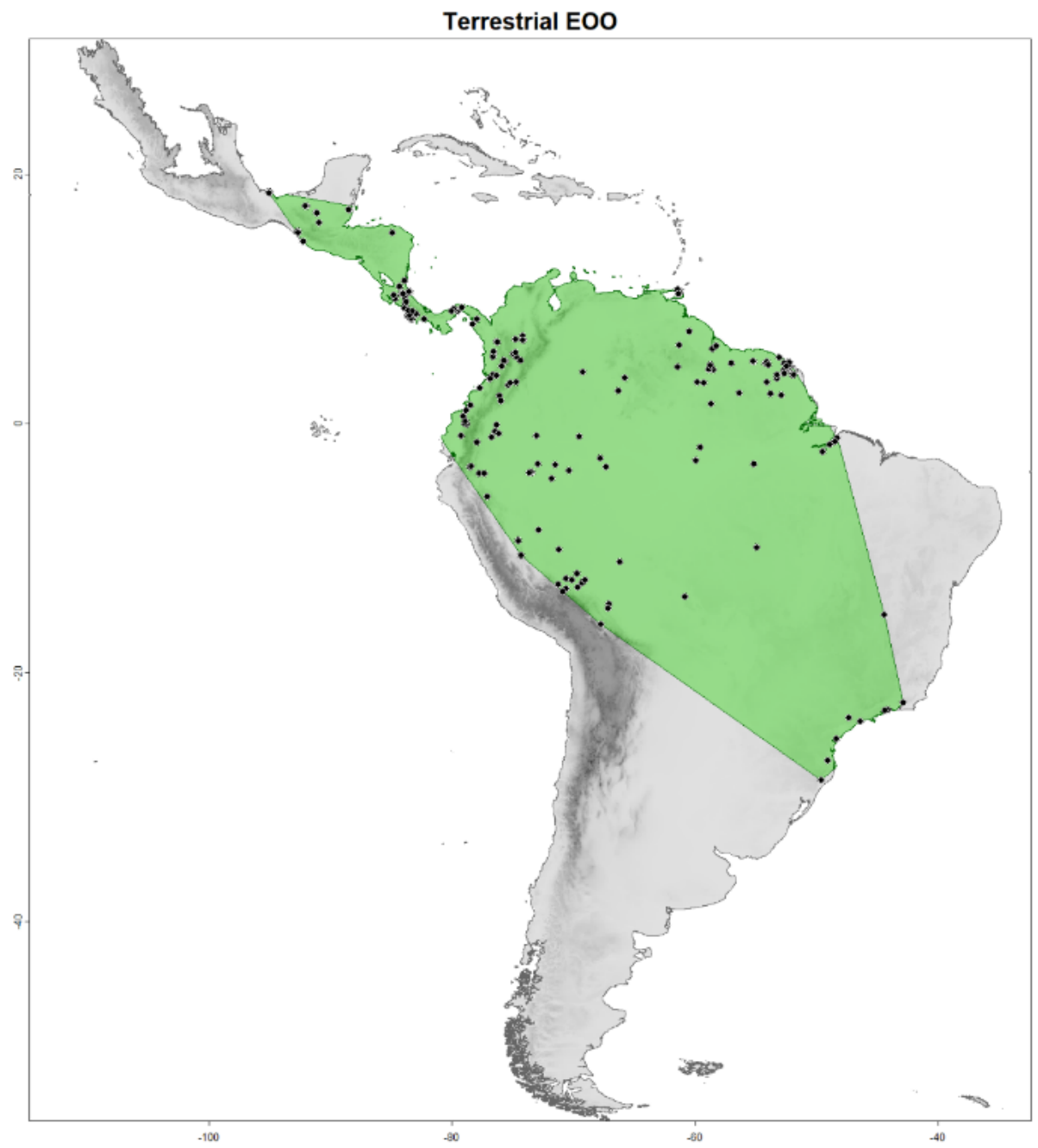

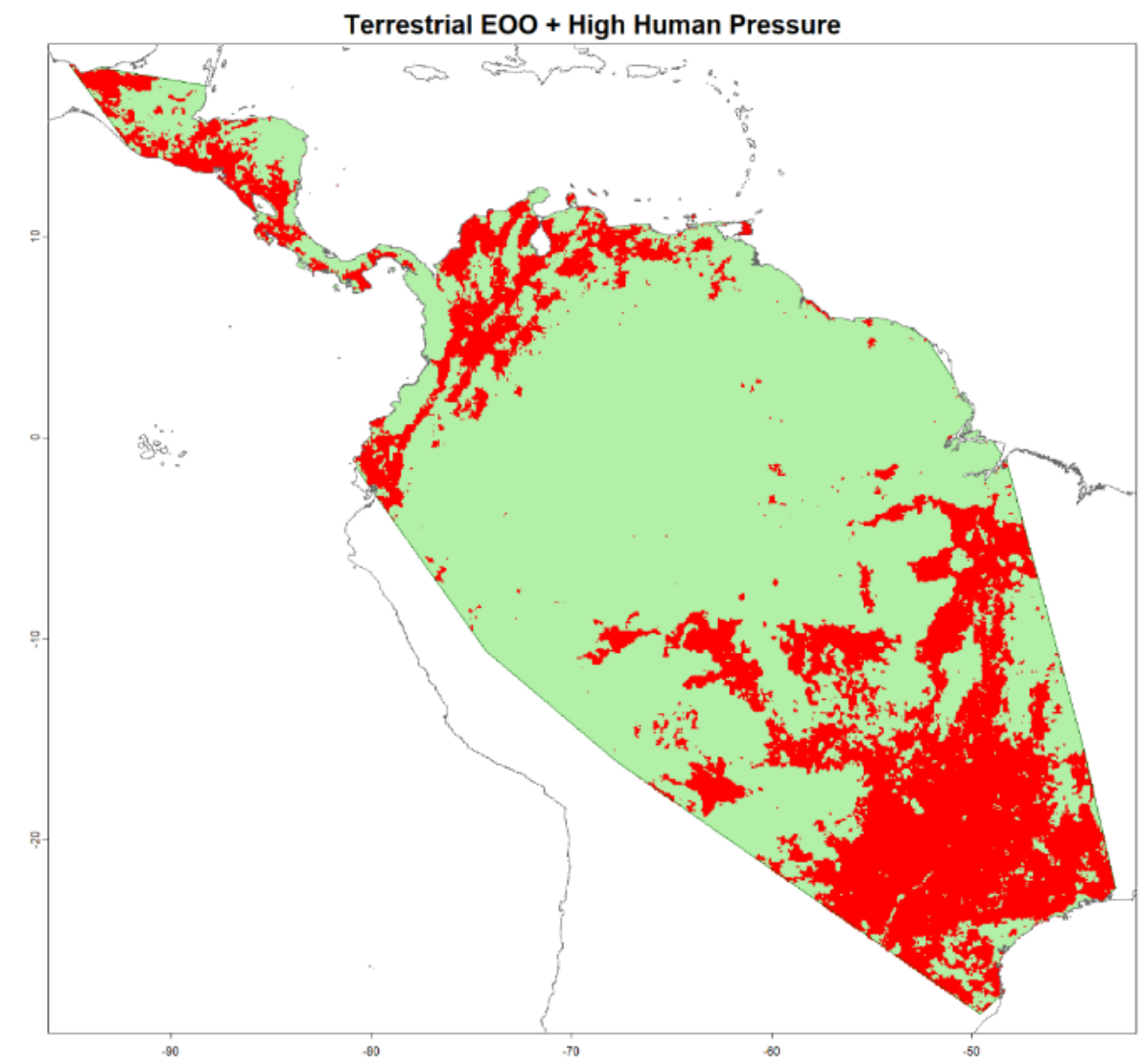

Total terrestrial area: 11277030 km² | High pressure: 3166864 km² (28.08 %)

Figure 92. Terrestrial range and anthropogenic pressure for *Thyroptera tricolor*. Maps illustrate: (left) Terrestrial Extent of Occurrence (EOO) shown in green with black dots representing occurrence records; (right) Areas of high human pressure (red) within the EOO, defined as regions exceeding the 70th percentile of the human footprint index. The caption below presents the total terrestrial area and the proportion experiencing intense human activity. Detailed methodology for calculating terrestrial EOO and human footprint percentage is described in **Supplementary Text 2** and the **main text**.

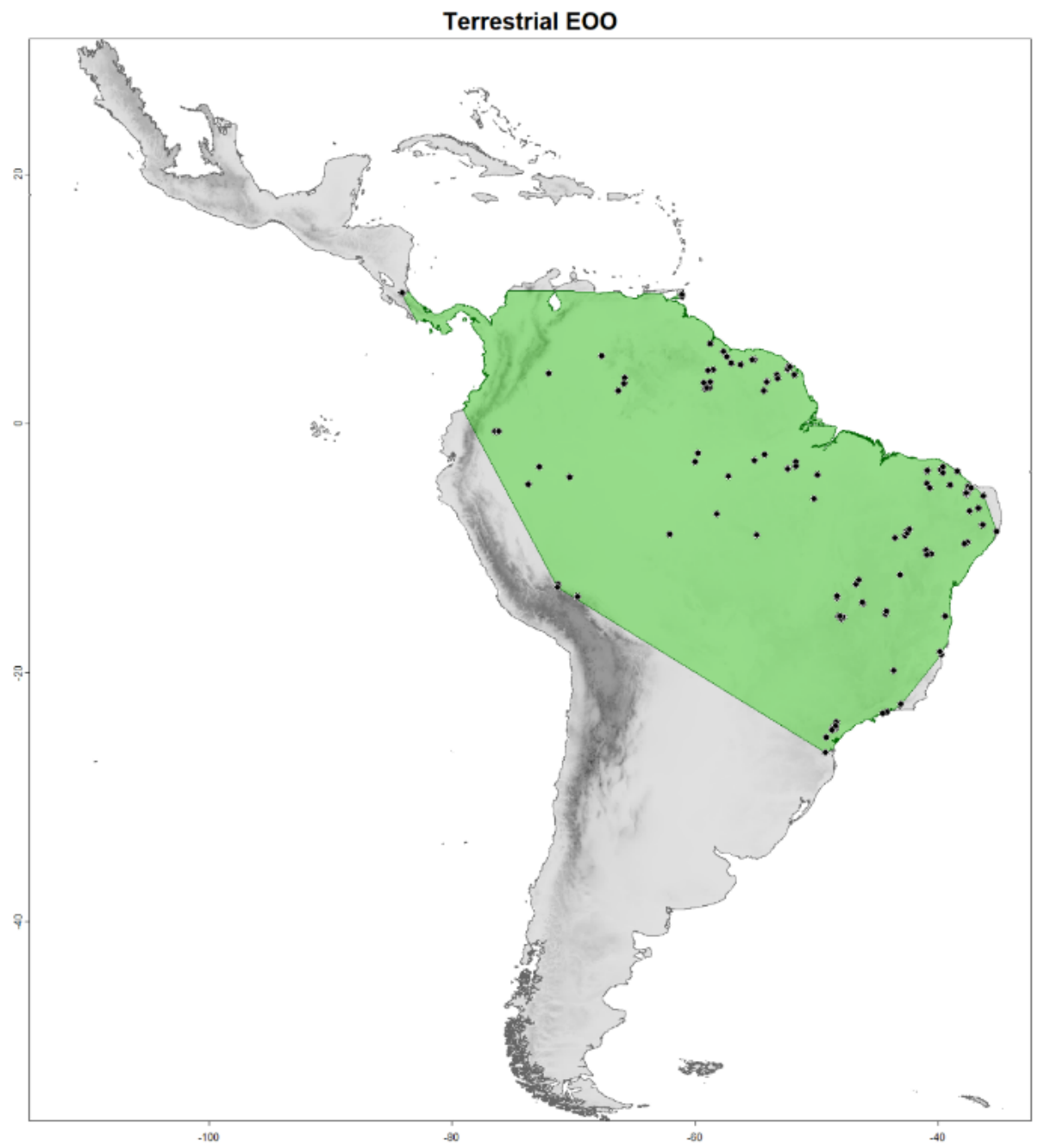

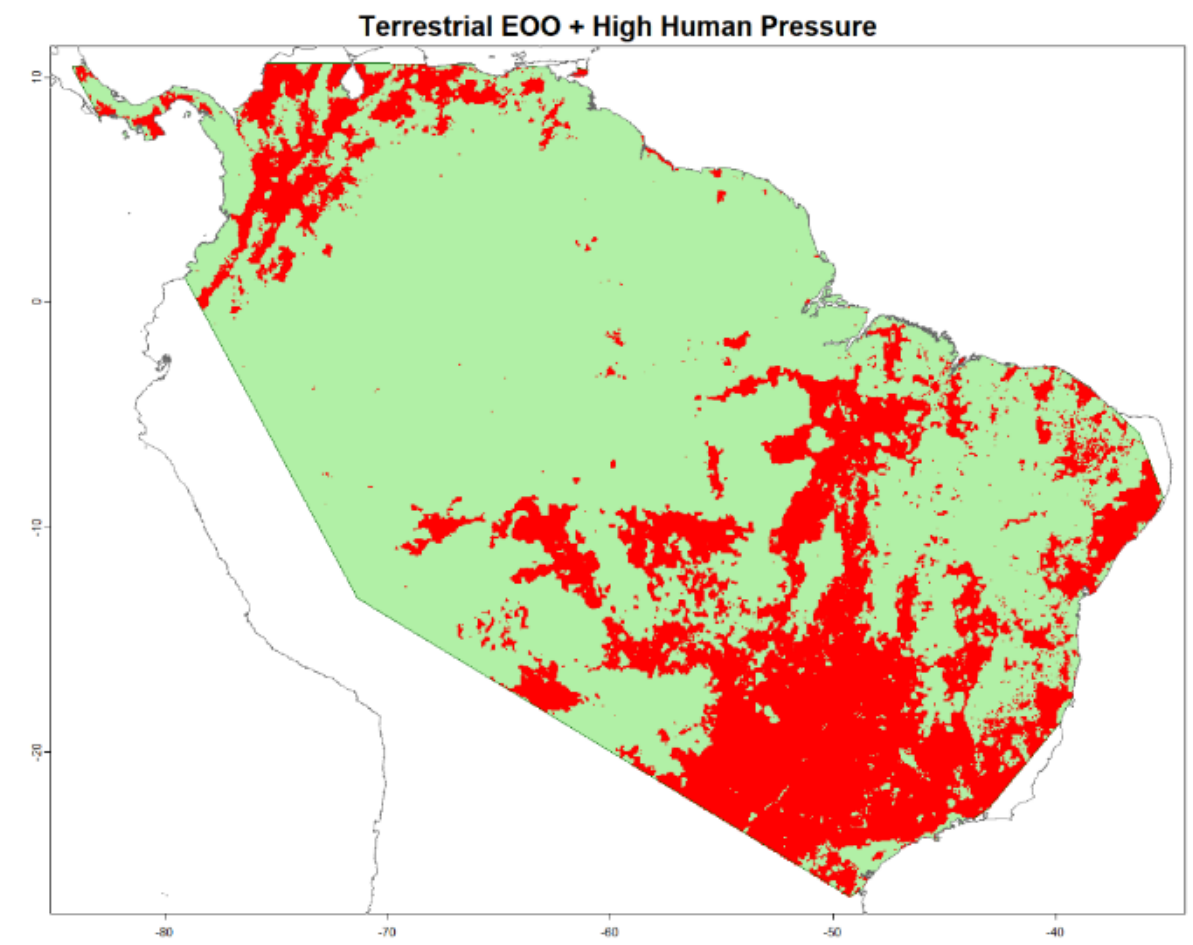

Total terrestrial area: 11738988 km² | High pressure: 3159515 km² (26.91 %)

Figure 93. Terrestrial range and anthropogenic pressure for *Furipterus horrens*. Maps illustrate: (left) Terrestrial Extent of Occurrence (EOO) shown in green with black dots representing occurrence records; (right) Areas of high human pressure (red) within the EOO, defined as regions exceeding the 70th percentile of the human footprint index. The caption below presents the total terrestrial area and the proportion experiencing intense human activity. Detailed methodology for calculating terrestrial EOO and human footprint percentage is described in **Supplementary Text 2** and the **main text**.

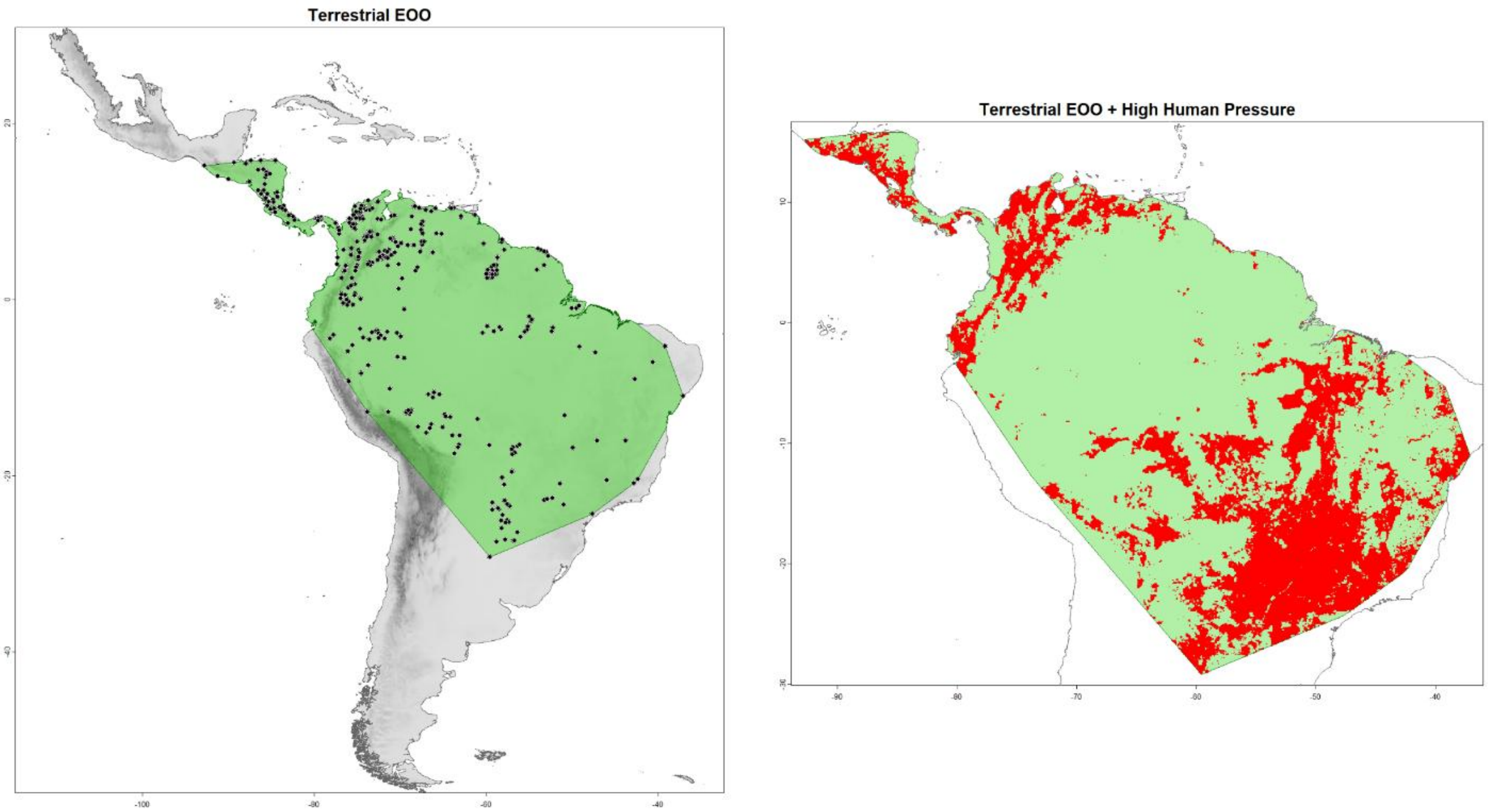

Total terrestrial area: 13437403 km² | High pressure: 3651375 km² (27.17 %)

Figure 94. Terrestrial range and anthropogenic pressure for *Noctilio albiventris*. Maps illustrate: (left) Terrestrial Extent of Occurrence (EOO) shown in green with black dots representing occurrence records; (right) Areas of high human pressure (red) within the EOO, defined as regions exceeding the 70th percentile of the human footprint index. The caption below presents the total terrestrial area and the proportion experiencing intense human activity. Detailed methodology for calculating terrestrial EOO and human footprint percentage is described in **Supplementary Text 2** and the **main text.**

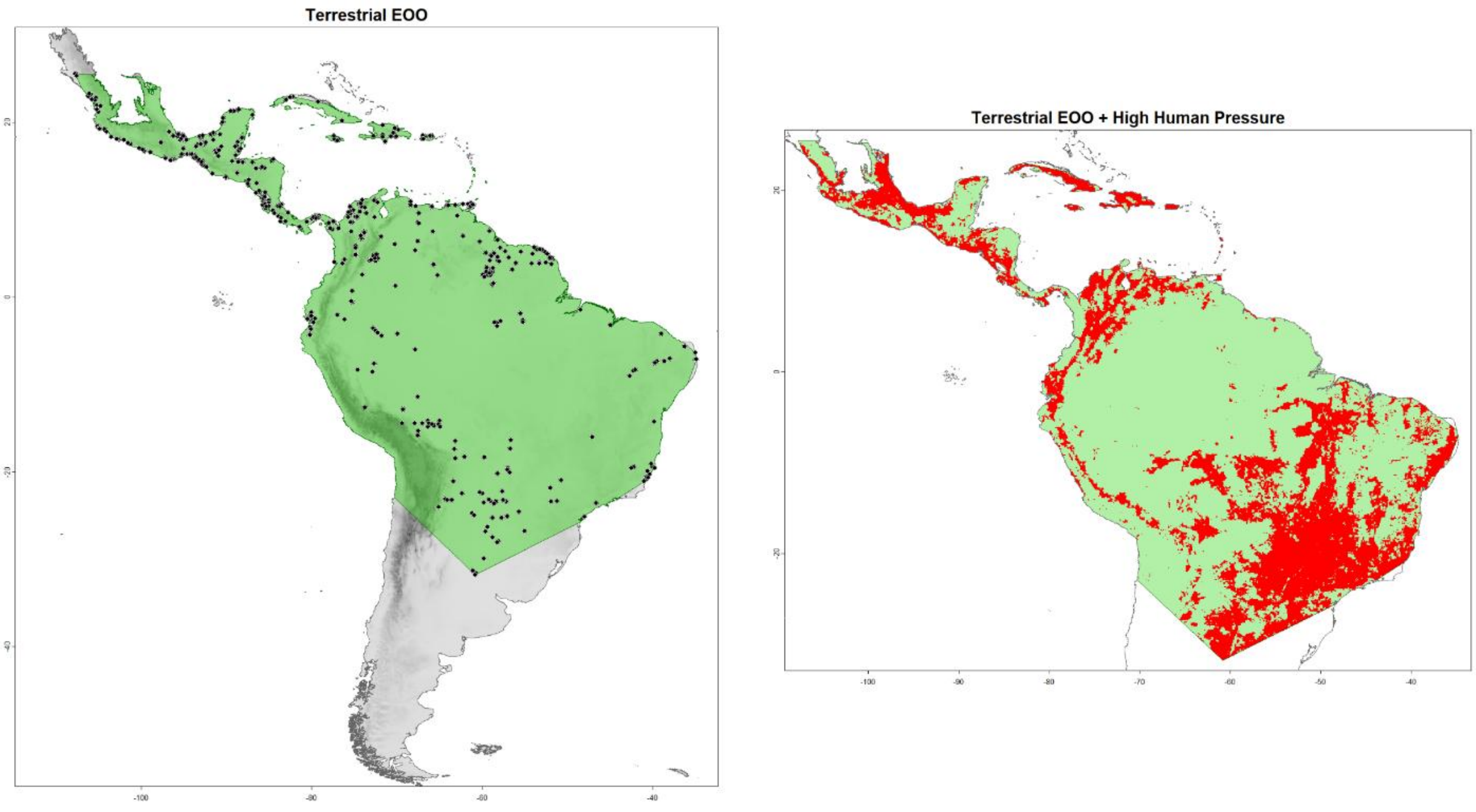

Total terrestrial area: 16143161 km² | High pressure: 4800793 km² (29.74 %)

Figure 95. Terrestrial range and anthropogenic pressure for *Noctilio leporinus*. Maps illustrate: (left) Terrestrial Extent of Occurrence (EOO) shown in green with black dots representing occurrence records; (right) Areas of high human pressure (red) within the EOO, defined as regions exceeding the 70th percentile of the human footprint index. The caption below presents the total terrestrial area and the proportion experiencing intense human activity. Detailed methodology for calculating terrestrial EOO and human footprint percentage is described in **Supplementary Text 2** and the **main text.**

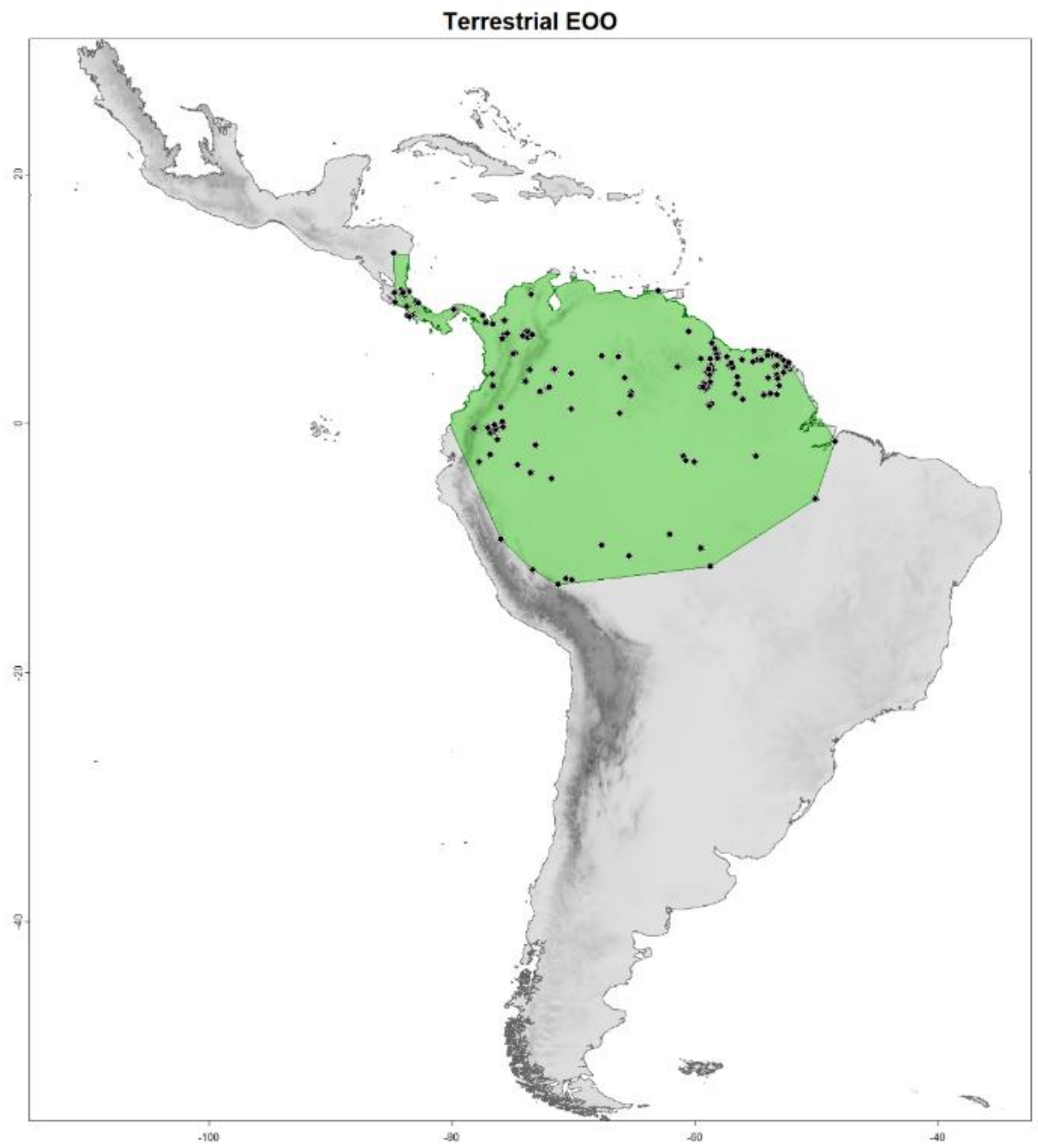

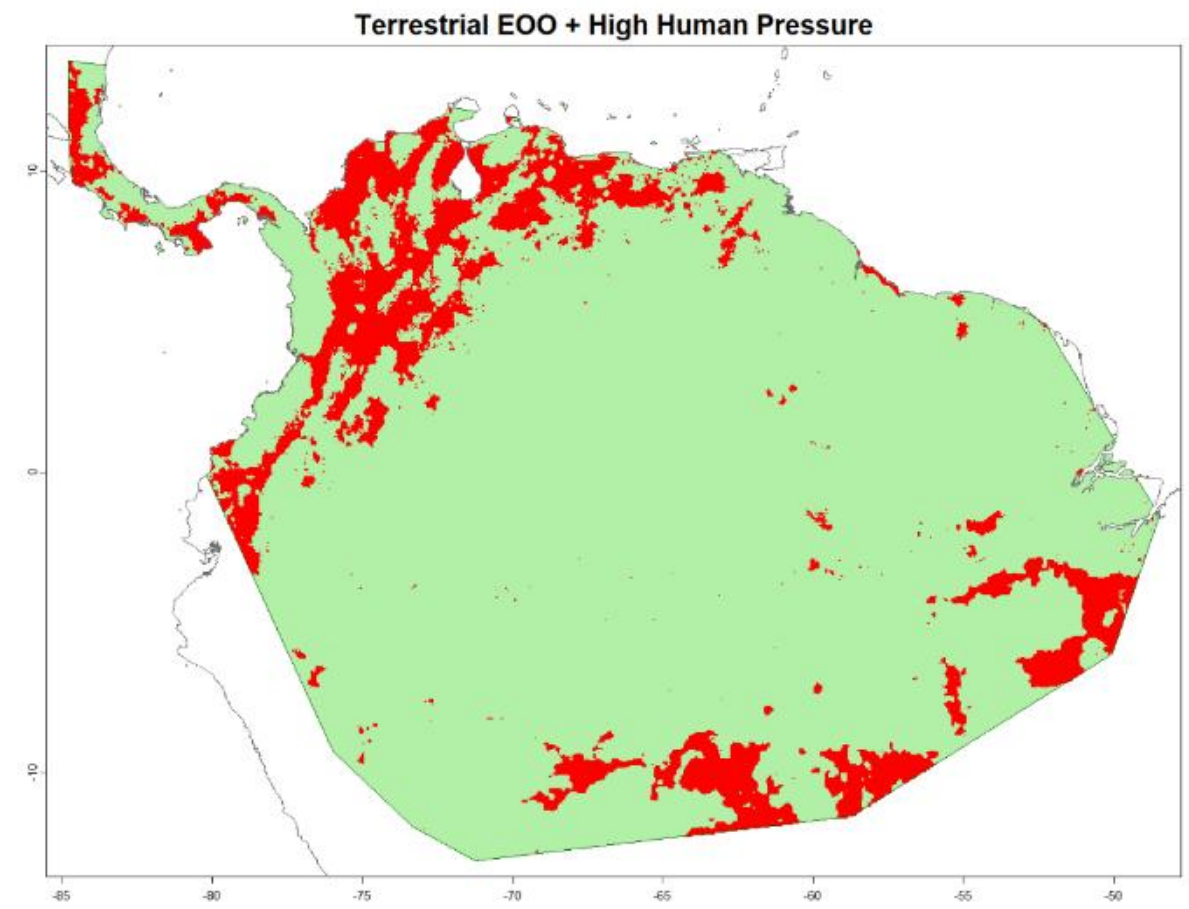

Total terrestrial area: 6880194 km² | High pressure: 883122 km² (12.84 %)

Figure 96. Terrestrial range and anthropogenic pressure for *Cormura brevirostris*. Maps illustrate: (left) Terrestrial Extent of Occurrence (EOO) shown in green with black dots representing occurrence records; (right) Areas of high human pressure (red) within the EOO, defined as regions exceeding the 70th percentile of the human footprint index. The caption below presents the total terrestrial area and the proportion experiencing intense human activity. Detailed methodology for calculating terrestrial EOO and human footprint percentage is described in **Supplementary Text 2** and the **main text.**

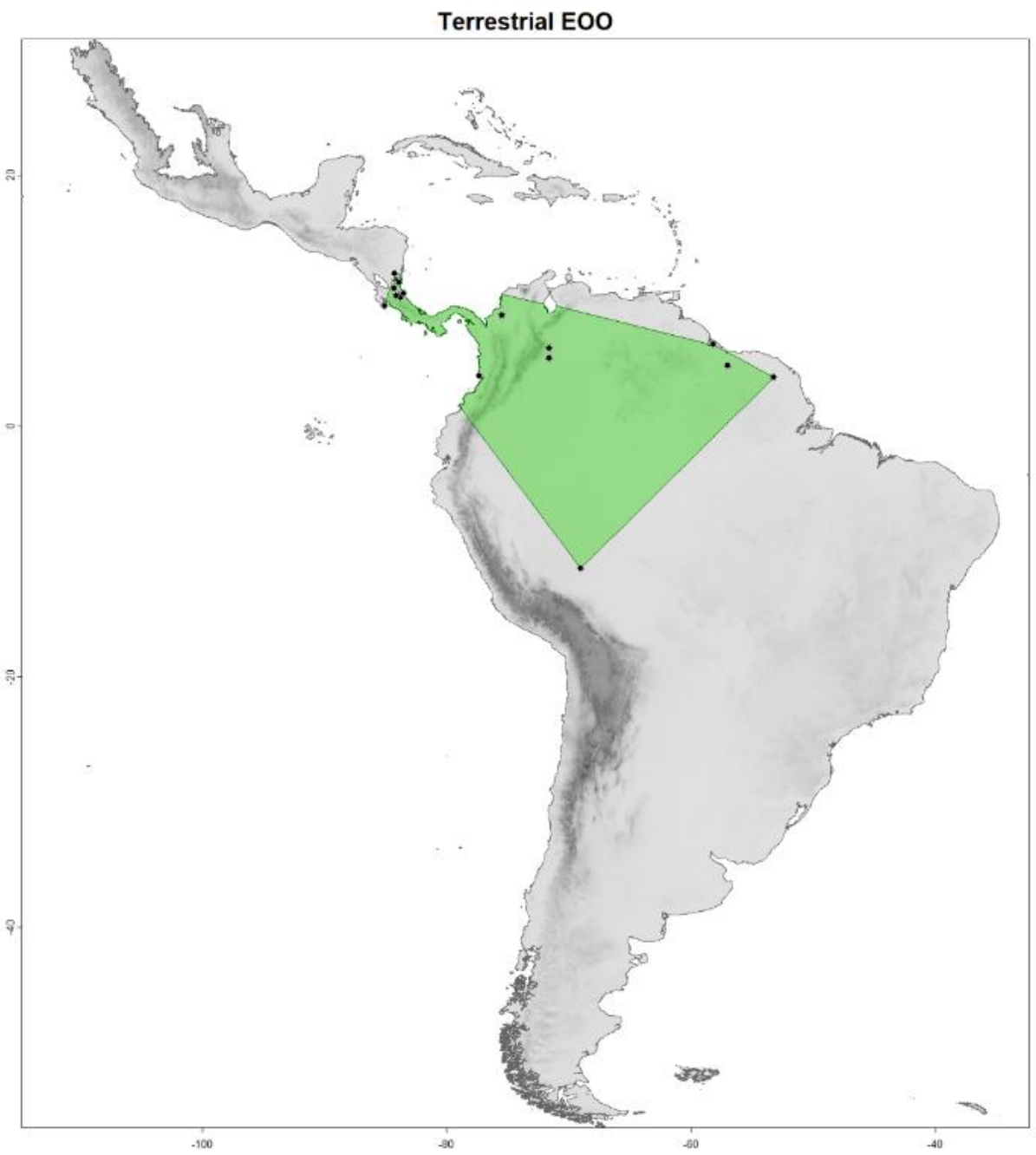

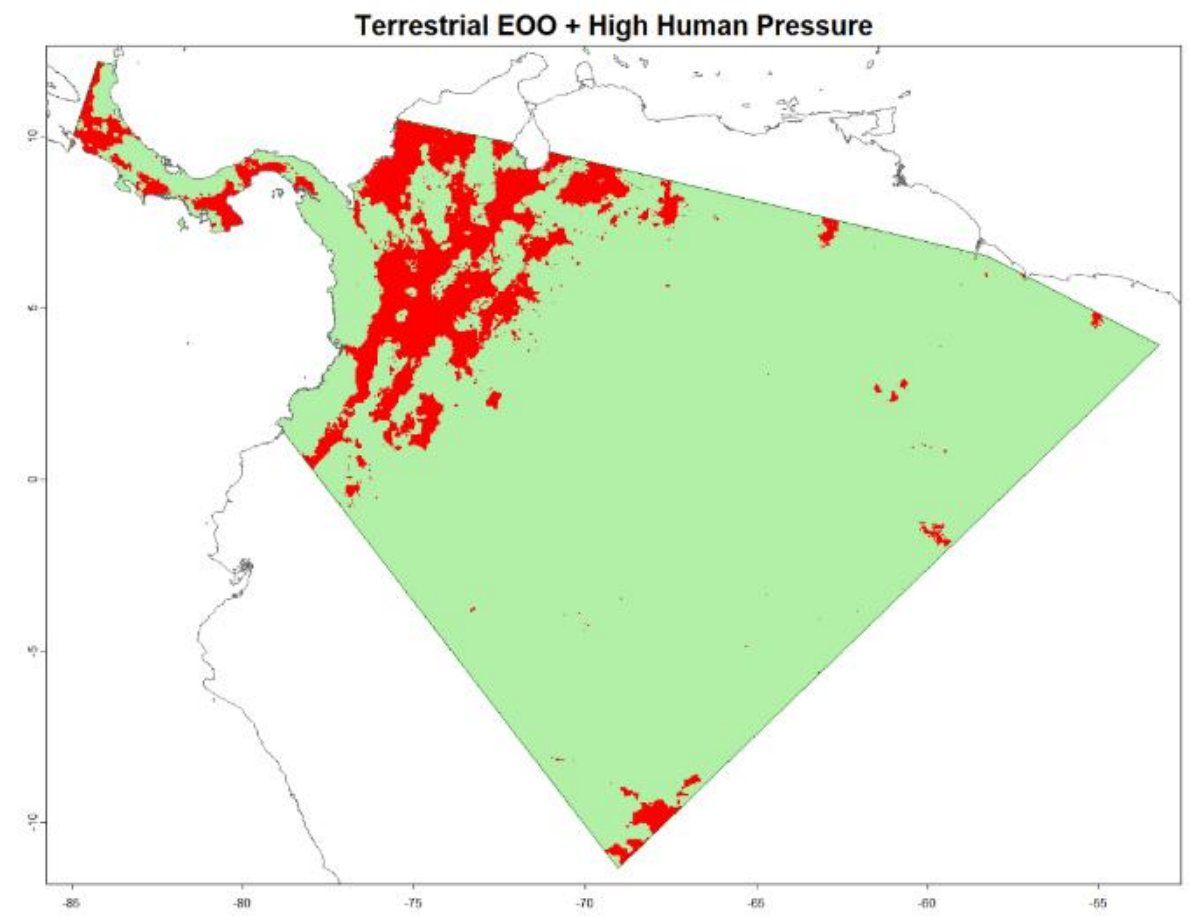

Total terrestrial area: 3842522 km² | High pressure: 398268 km² (10.36 %)

Figure 97. Terrestrial range and anthropogenic pressure for *Cyttarops alecto*. Maps illustrate: (left) Terrestrial Extent of Occurrence (EOO) shown in green with black dots representing occurrence records; (right) Areas of high human pressure (red) within the EOO, defined as regions exceeding the 70th percentile of the human footprint index. The caption below presents the total terrestrial area and the proportion experiencing intense human activity. Detailed methodology for calculating terrestrial EOO and human footprint percentage is described in **Supplementary Text 2** and the **main text.**

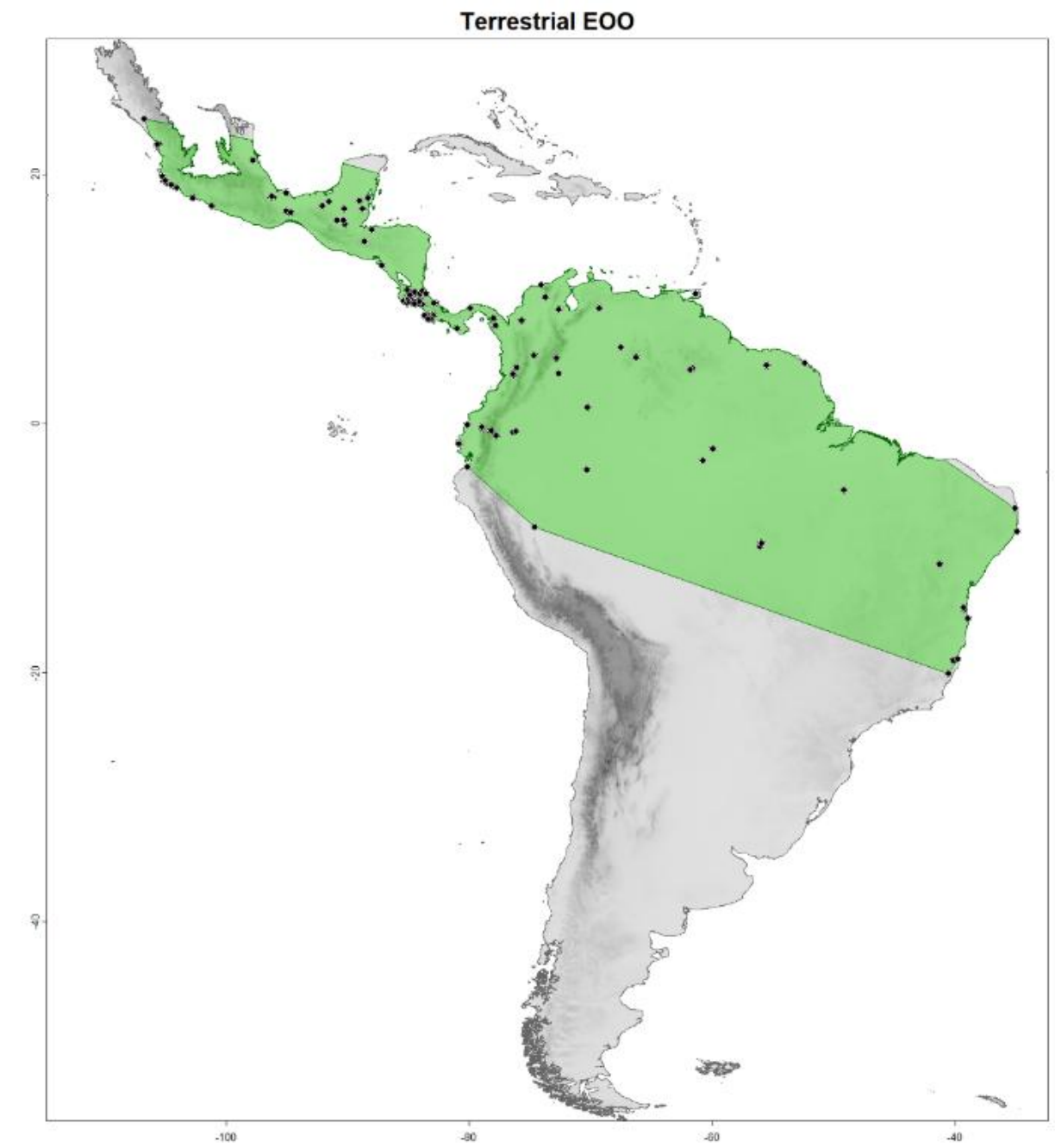

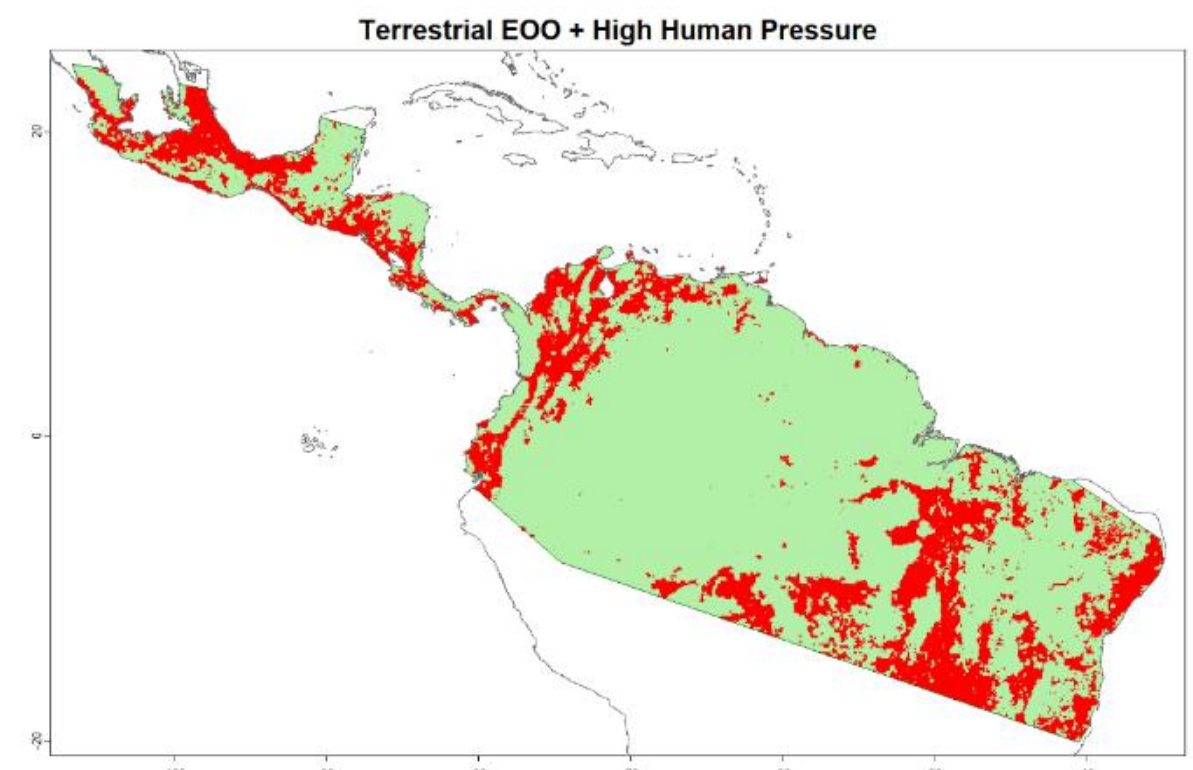

Total terrestrial area: 11115323 km² | High pressure: 2622799 km² (23.6 %)

Figure 98. Terrestrial range and anthropogenic pressure for *Diclidurus albus*. Maps illustrate: (left) Terrestrial Extent of Occurrence (EOO) shown in green with black dots representing occurrence records; (right) Areas of high human pressure (red) within the EOO, defined as regions exceeding the 70th percentile of the human footprint index. The caption below presents the total terrestrial area and the proportion experiencing intense human activity. Detailed methodology for calculating terrestrial EOO and human footprint percentage is described in **Supplementary Text 2** and the **main text.**

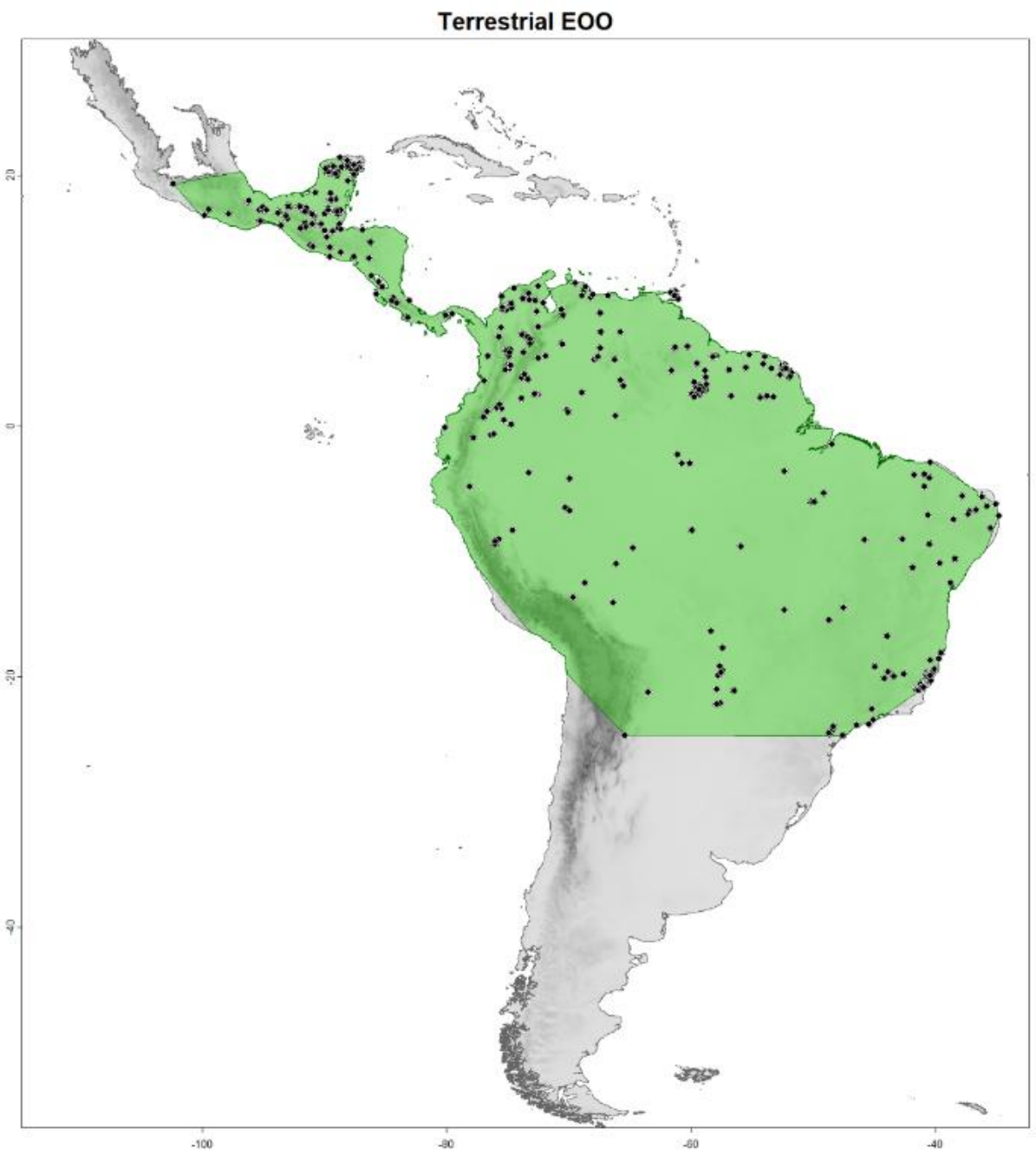

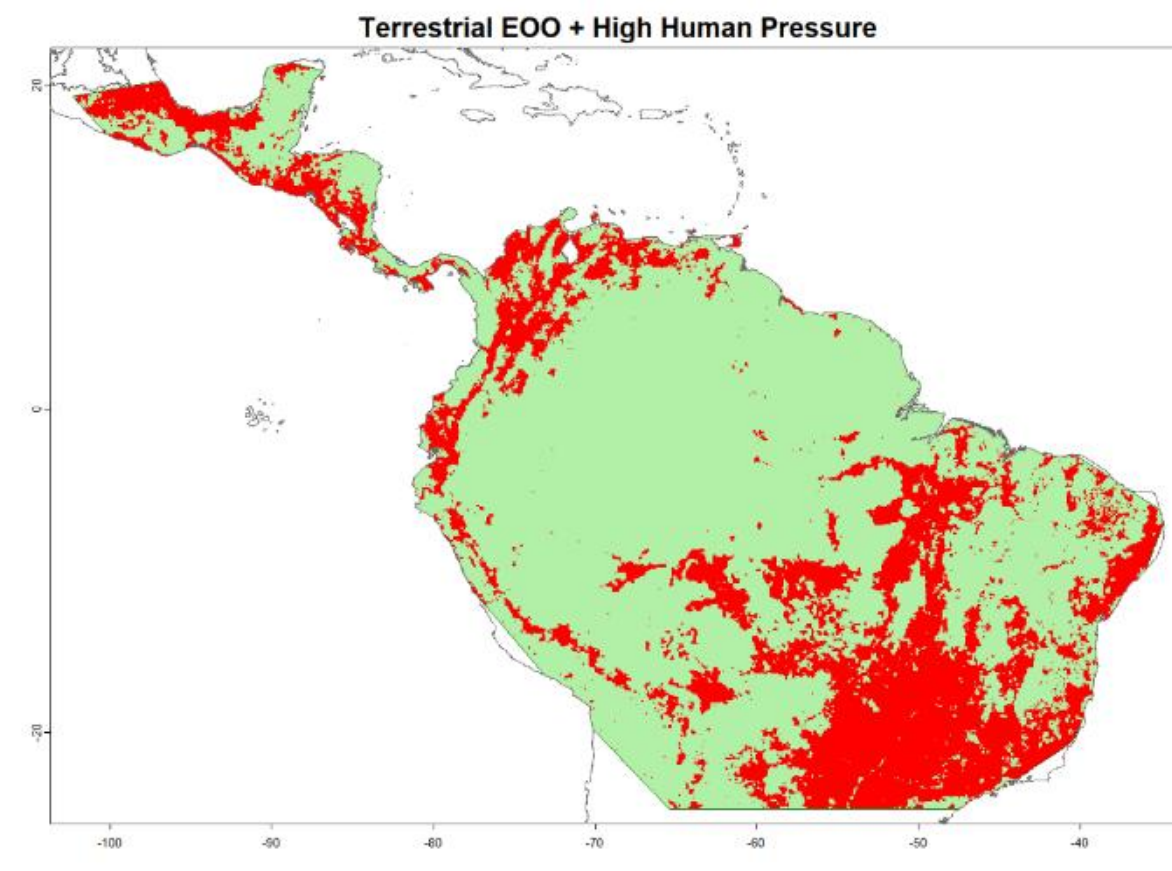

Total terrestrial area: 14591584 km² | High pressure: 4021984 km² (27.56 %)

Figure 99. Terrestrial range and anthropogenic pressure for *Peropteryx macrotis*. Maps illustrate: (left) Terrestrial Extent of Occurrence (EOO) shown in green with black dots representing occurrence records; (right) Areas of high human pressure (red) within the EOO, defined as regions exceeding the 70th percentile of the human footprint index. The caption below presents the total terrestrial area and the proportion experiencing intense human activity. Detailed methodology for calculating terrestrial EOO and human footprint percentage is described in **Supplementary Text 2** and the **main text.**

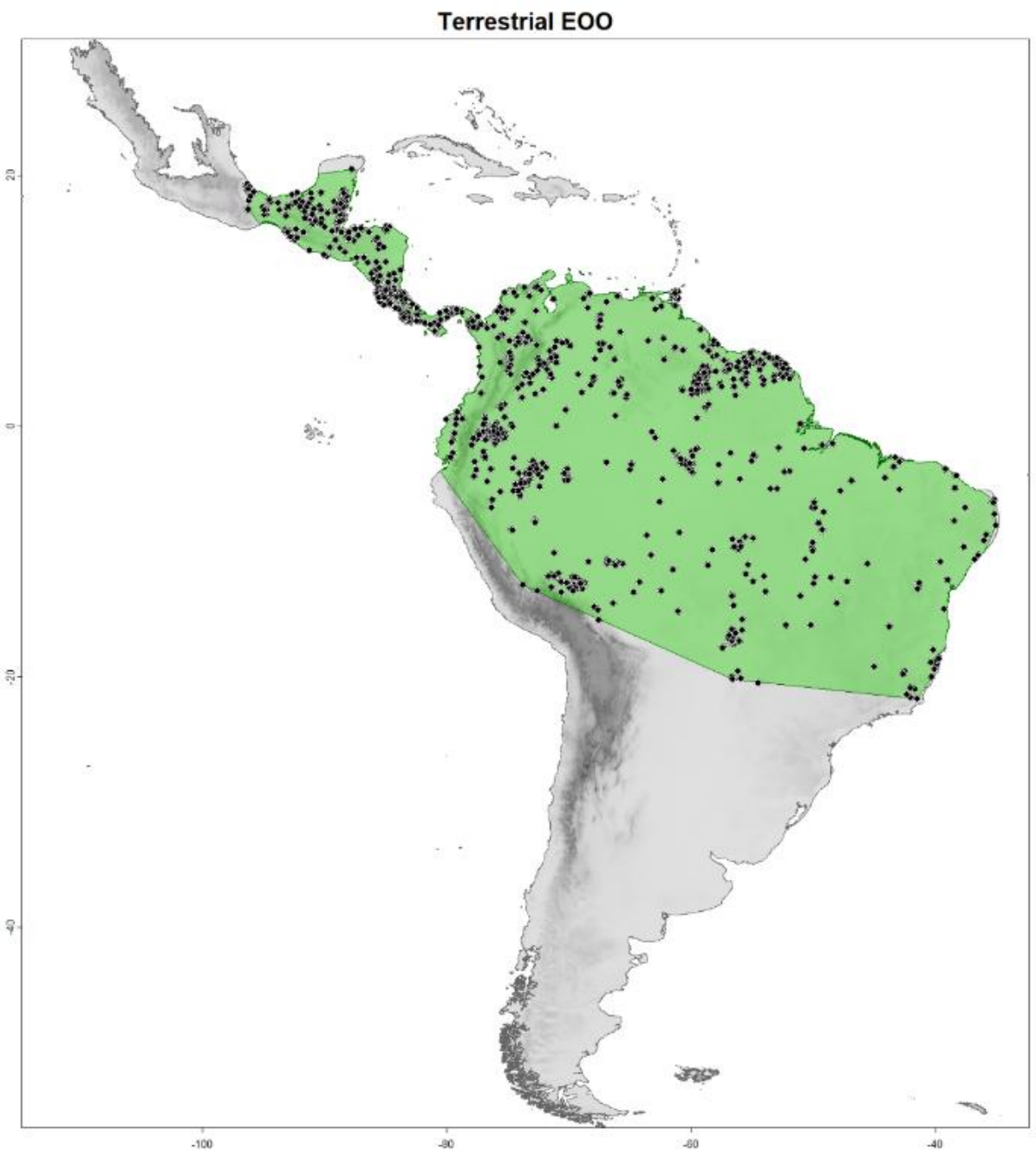

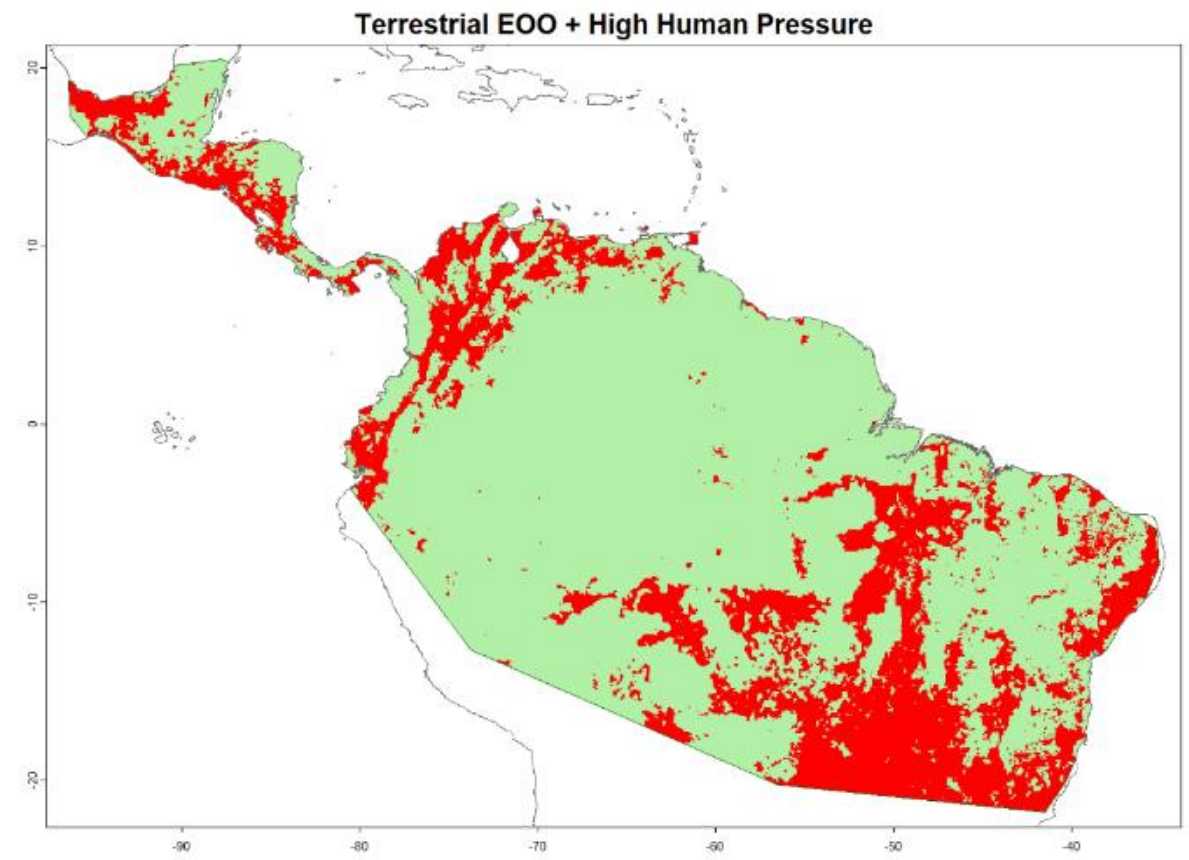

Total terrestrial area: 12372620 km² | High pressure: 3121483 km² (25.23 %)

Figure 100. Terrestrial range and anthropogenic pressure for *Rhynchonycteris naso*. Maps illustrate: (left) Terrestrial Extent of Occurrence (EOO) shown in green with black dots representing occurrence records; (right) Areas of high human pressure (red) within the EOO, defined as regions exceeding the 70th percentile of the human footprint index. The caption below presents the total terrestrial area and the proportion experiencing intense human activity. Detailed methodology for calculating terrestrial EOO and human footprint percentage is described in **Supplementary Text 2** and the **main text.**

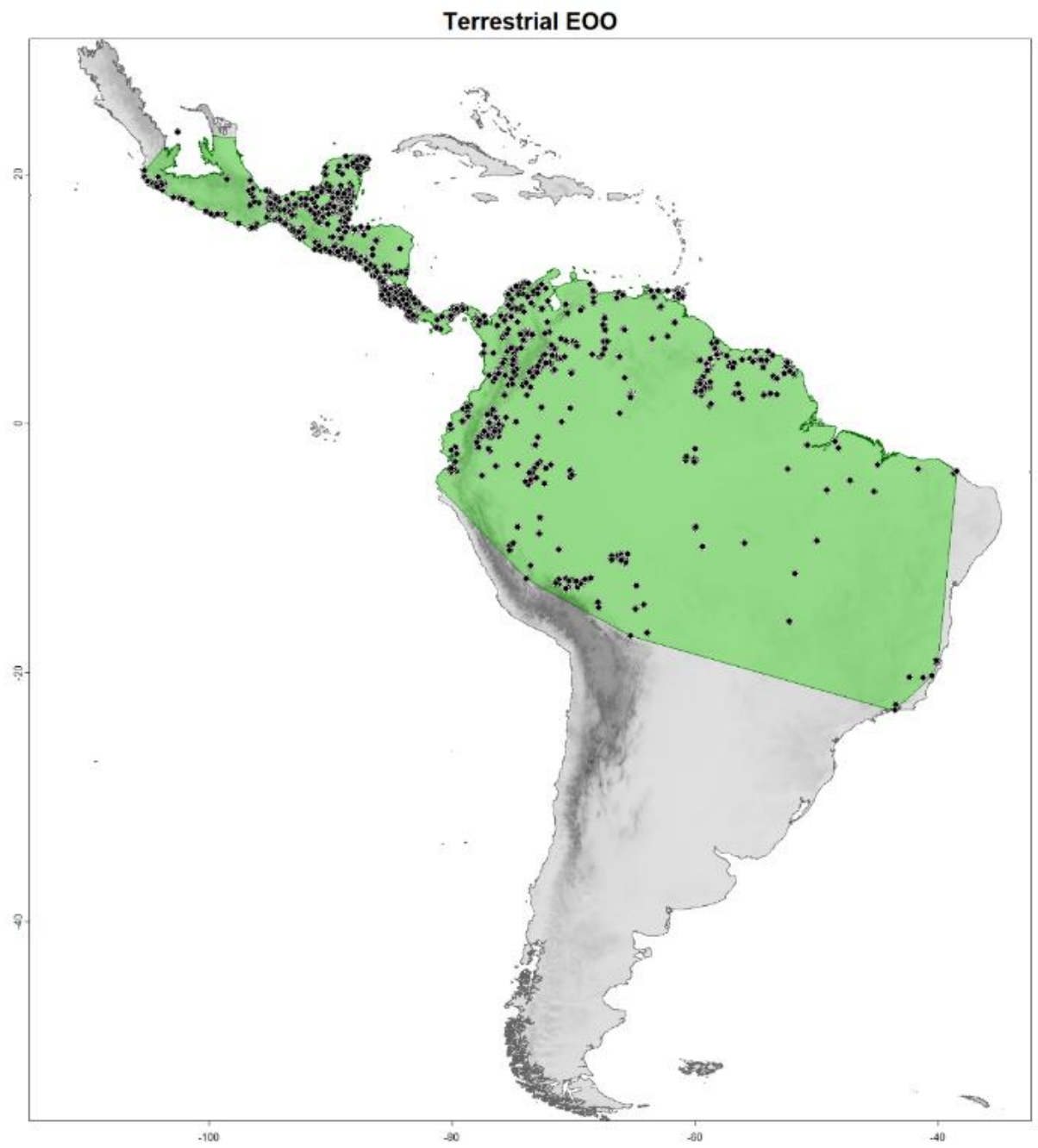

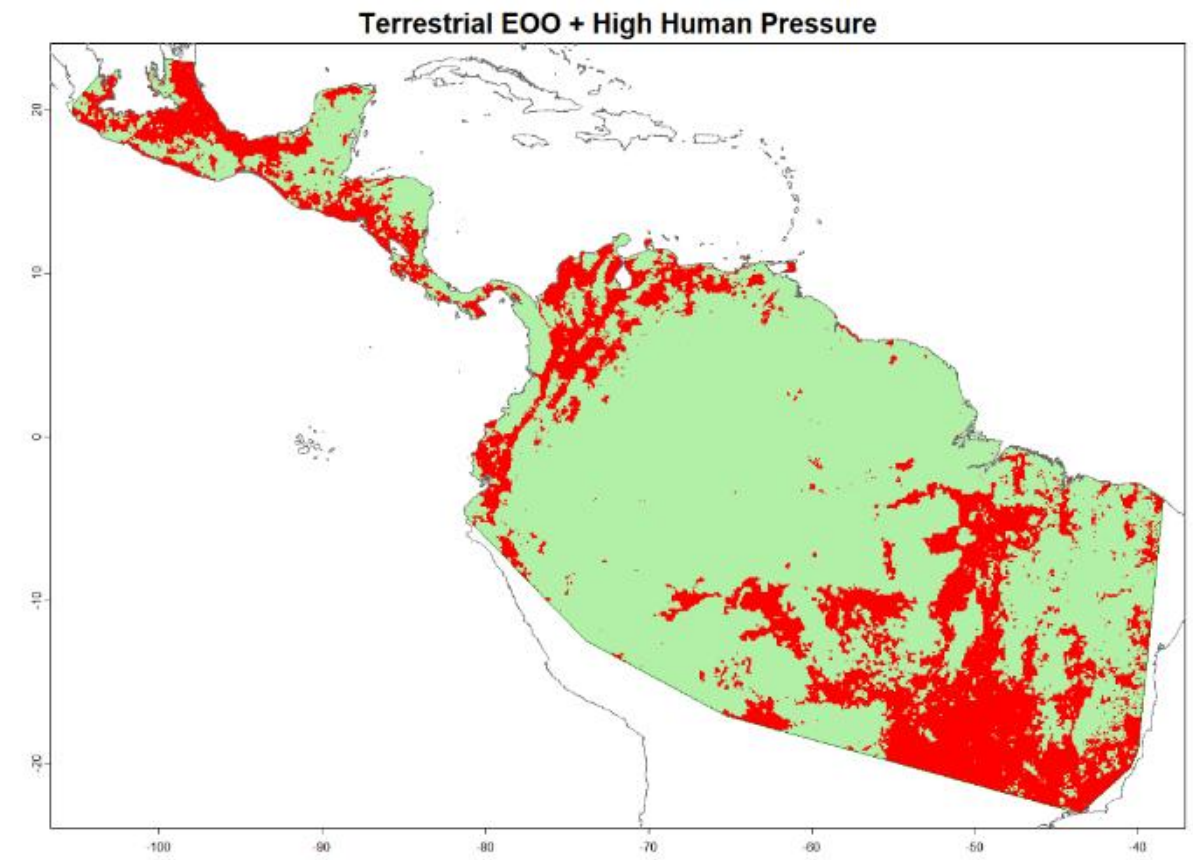

Total terrestrial area: 12684356 km² | High pressure: 3306486 km² (26.07 %)

Figure 101. Terrestrial range and anthropogenic pressure for *Saccopteryx bilineata*. Maps illustrate: (left) Terrestrial Extent of Occurrence (EOO) shown in green with black dots representing occurrence records; (right) Areas of high human pressure (red) within the EOO, defined as regions exceeding the 70th percentile of the human footprint index. The caption below presents the total terrestrial area and the proportion experiencing intense human activity. Detailed methodology for calculating terrestrial EOO and human footprint percentage is described in **Supplementary Text 2** and the **main text.**

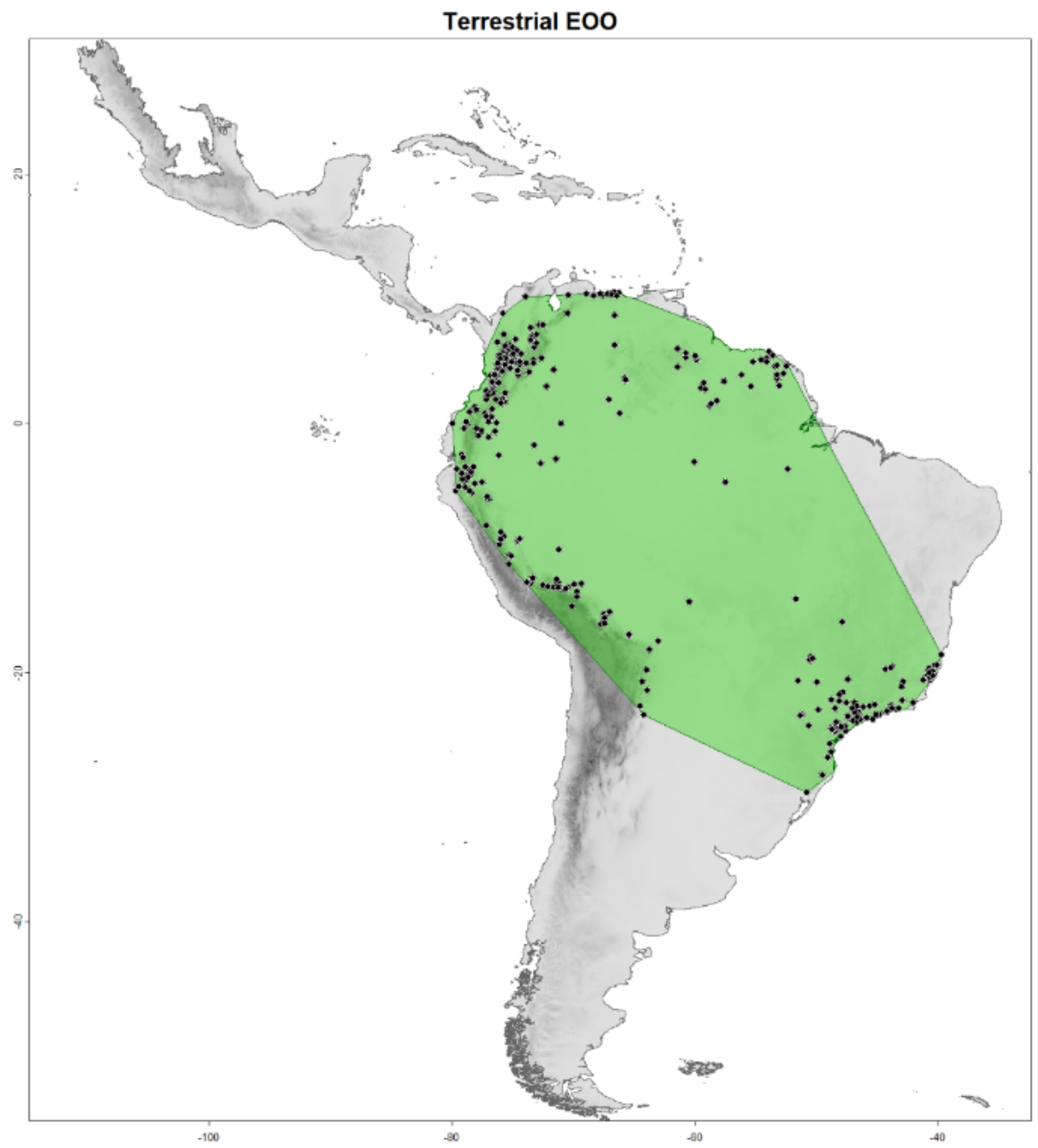

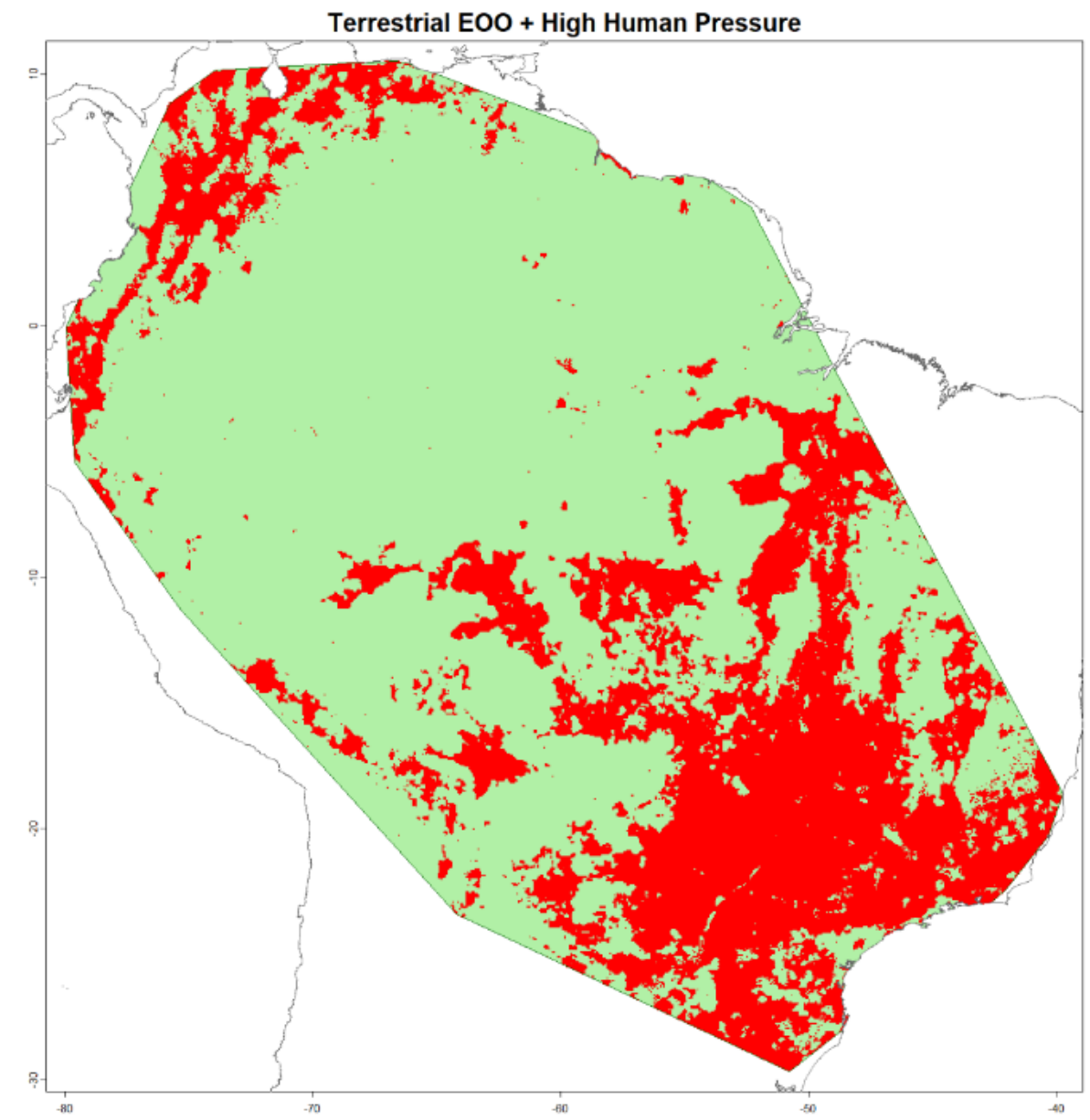

Total terrestrial area: 11833214 km² | High pressure: 3318098 km² (28.04 %)

Figure 102. Terrestrial range and anthropogenic pressure for *Anoura caudifer*. Maps illustrate: (left) Terrestrial Extent of Occurrence (EOO) shown in green with black dots representing occurrence records; (right) Areas of high human pressure (red) within the EOO, defined as regions exceeding the 70th percentile of the human footprint index. The caption below presents the total terrestrial area and the proportion experiencing intense human activity. Detailed methodology for calculating terrestrial EOO and human footprint percentage is described in **Supplementary Text 2** and the **main text.**

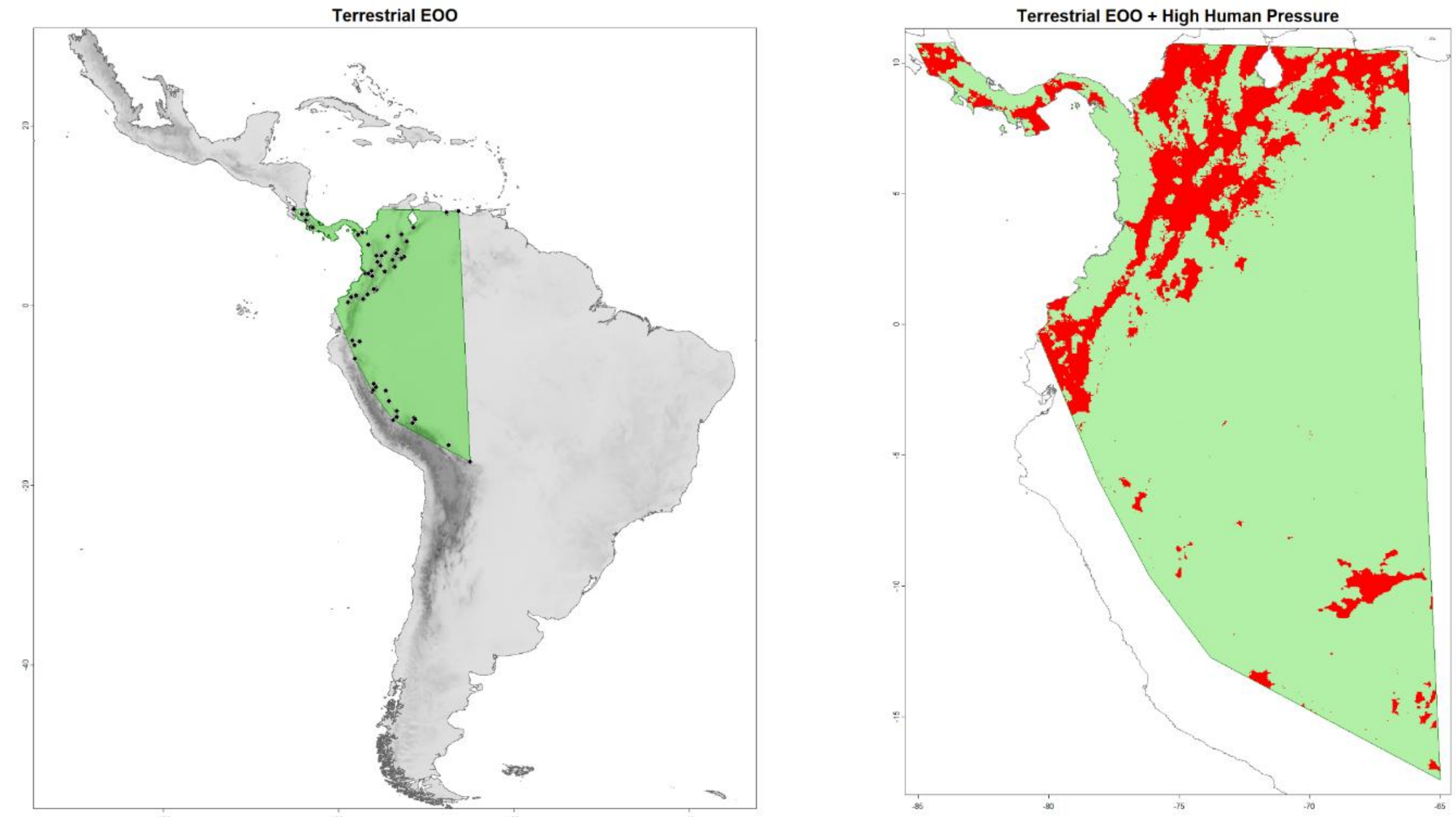

Total terrestrial area: 3724873 km² | High pressure: 555134 km² (14.9 %)

Figure 103. Terrestrial range and anthropogenic pressure for *Anoura cultrata*. Maps illustrate: (left) Terrestrial Extent of Occurrence (EOO) shown in green with black dots representing occurrence records; (right) Areas of high human pressure (red) within the EOO, defined as regions exceeding the 70th percentile of the human footprint index. The caption below presents the total terrestrial area and the proportion experiencing intense human activity. Detailed methodology for calculating terrestrial EOO and human footprint percentage is described in **Supplementary Text 2** and the **main text.**

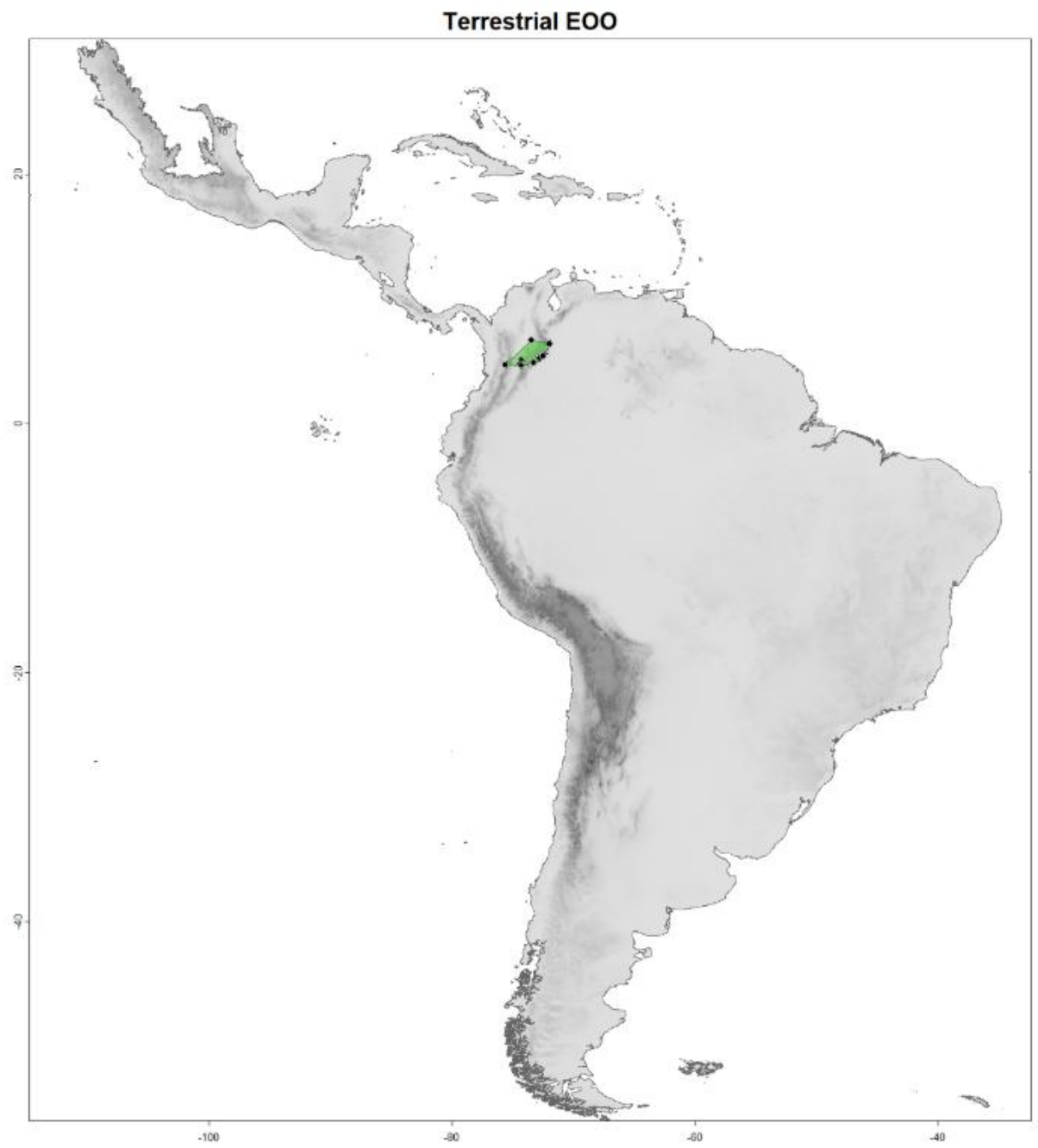

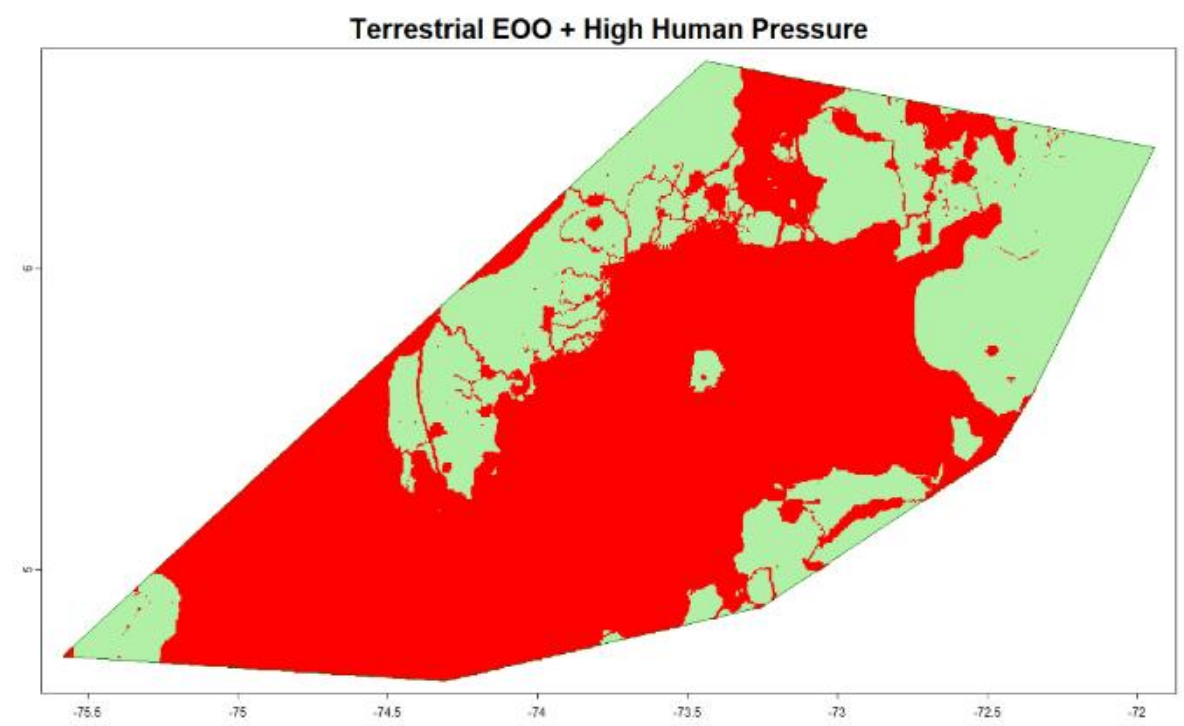

Total terrestrial area: 48136 km² | High pressure: 31409 km² (65.25 %)

Figure 104. Terrestrial range and anthropogenic pressure for *Anoura luismanueli*. Maps illustrate: (left) Terrestrial Extent of Occurrence (EOO) shown in green with black dots representing occurrence records; (right) Areas of high human pressure (red) within the EOO, defined as regions exceeding the 70th percentile of the human footprint index. The caption below presents the total terrestrial area and the proportion experiencing intense human activity. Detailed methodology for calculating terrestrial EOO and human footprint percentage is described in **Supplementary Text 2** and the **main text.**

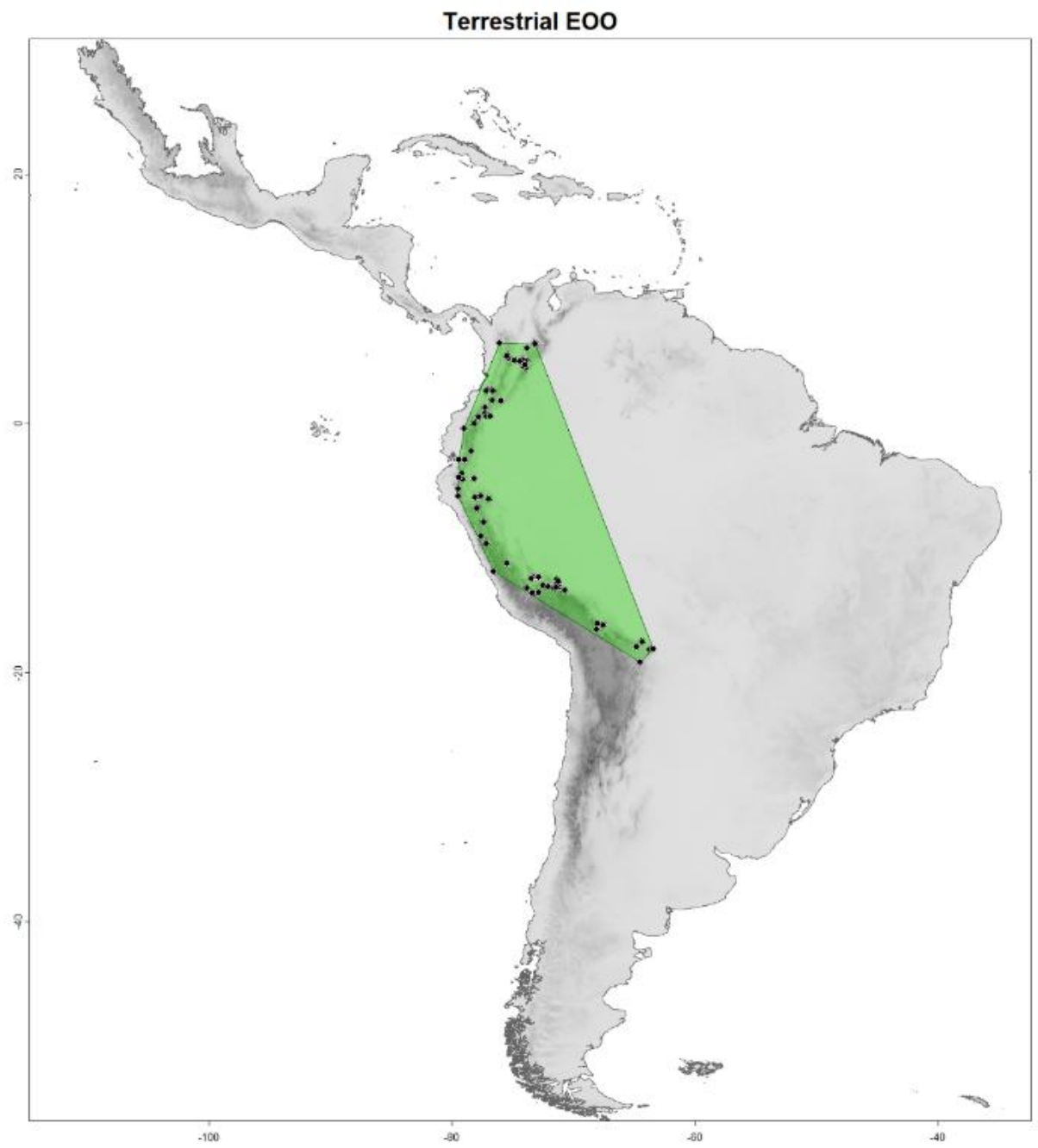

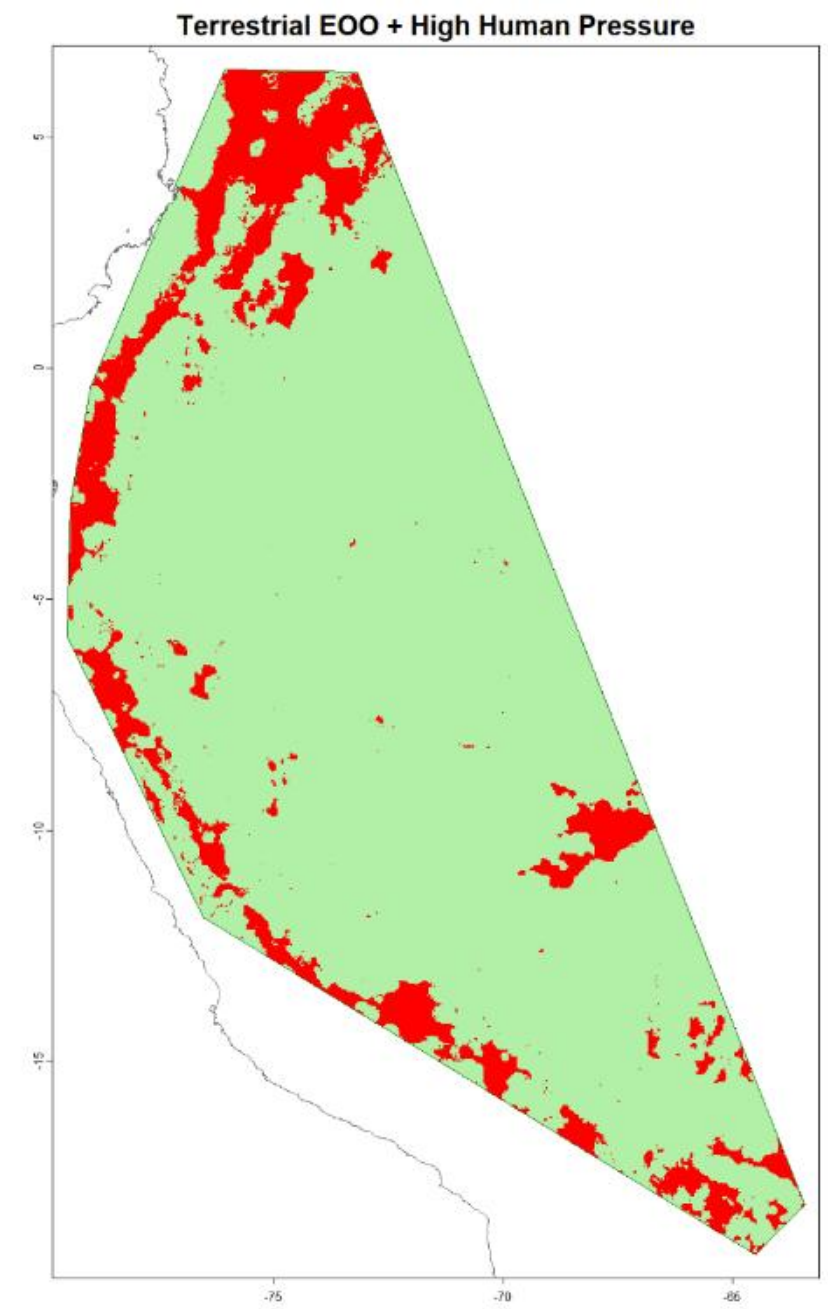

Total terrestrial area: 2454375 km² | High pressure: 330477 km² (13.46 %)

Figure 105. Terrestrial range and anthropogenic pressure for *Anoura peruana*. Maps illustrate: (left) Terrestrial Extent of Occurrence (EOO) shown in green with black dots representing occurrence records; (right) Areas of high human pressure (red) within the EOO, defined as regions exceeding the 70th percentile of the human footprint index. The caption below presents the total terrestrial area and the proportion experiencing intense human activity. Detailed methodology for calculating terrestrial EOO and human footprint percentage is described in **Supplementary Text 2** and the **main text.**

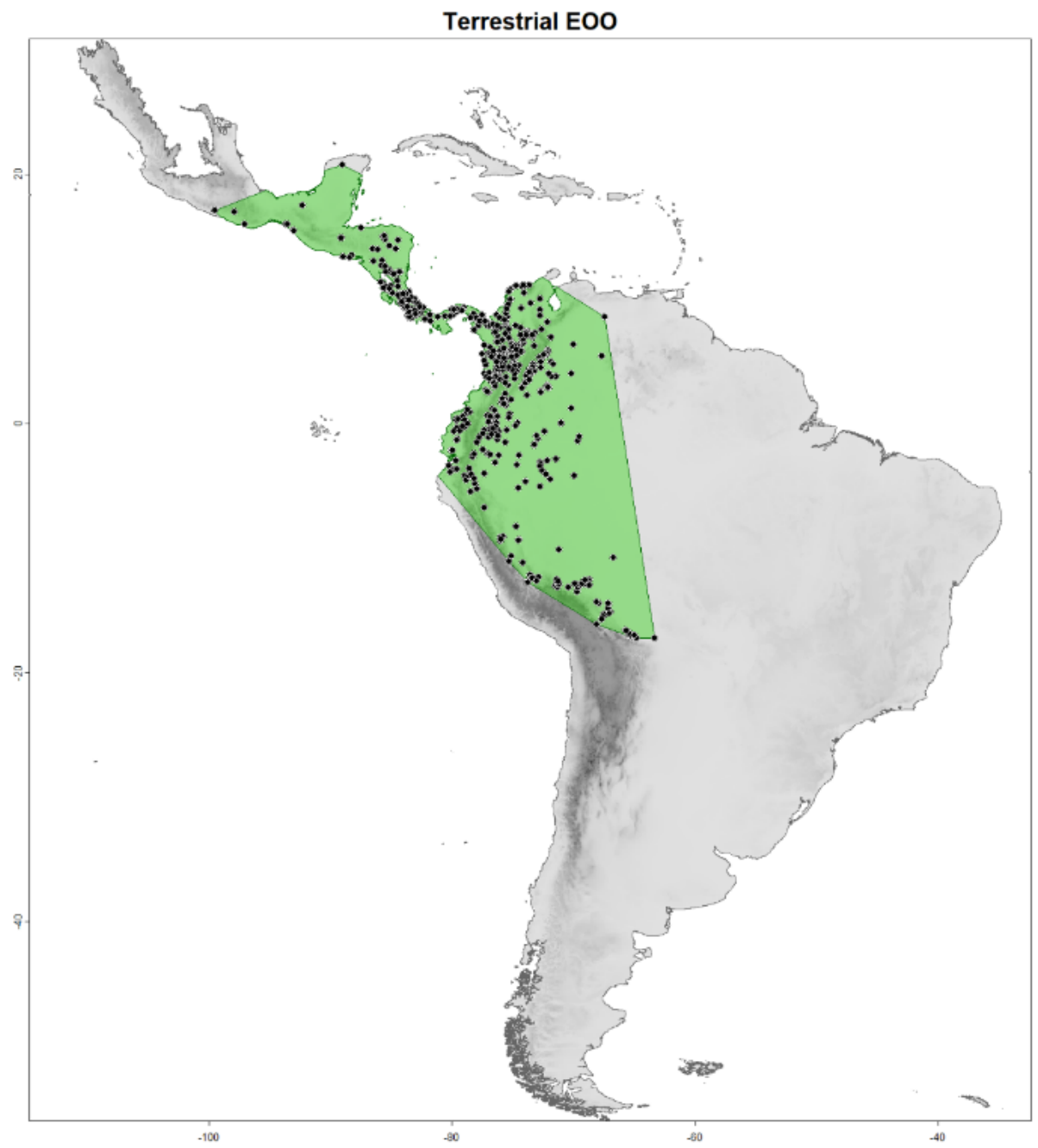

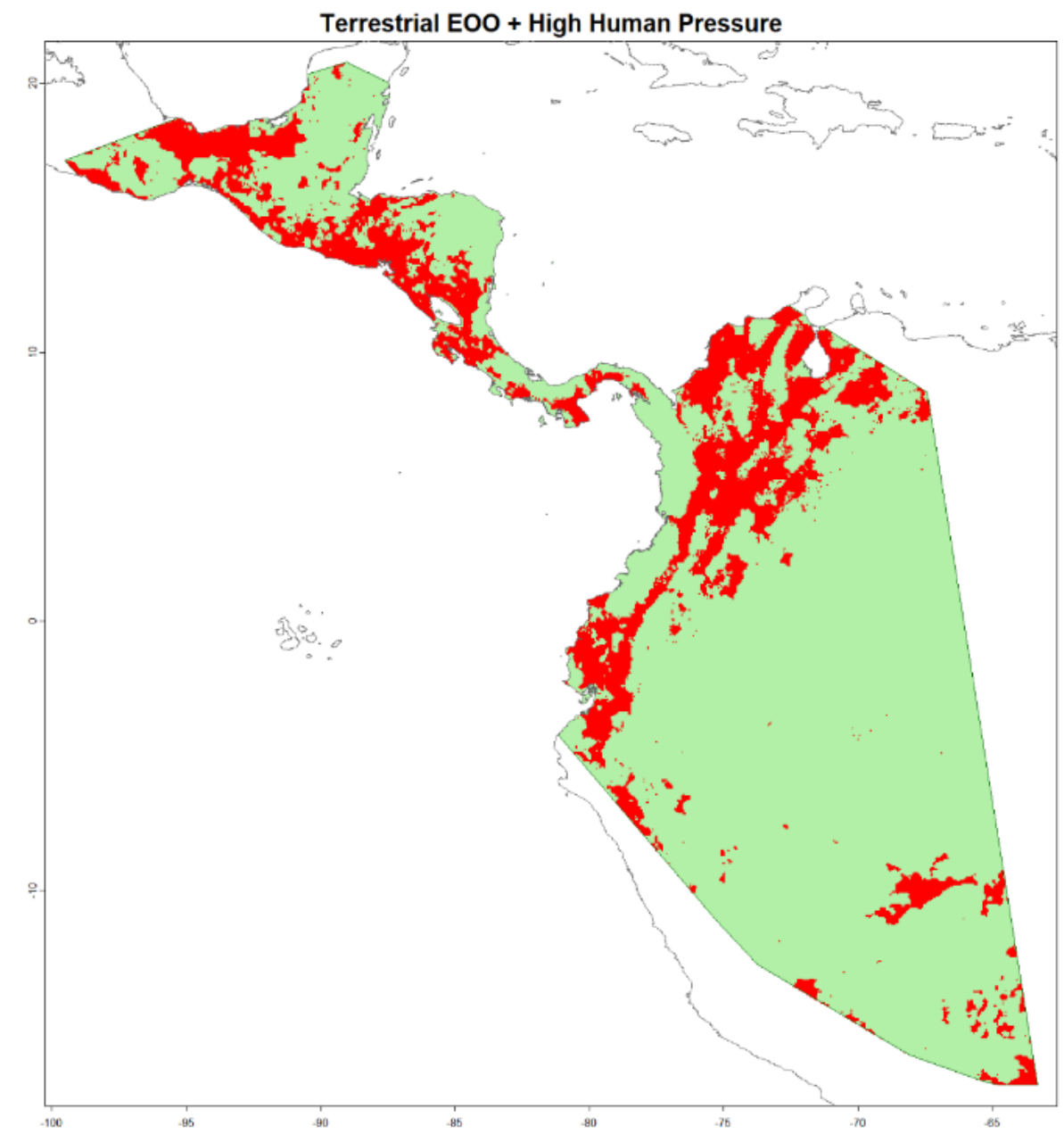

Total terrestrial area: 4622473 km² | High pressure: 884726 km² (19.14 %)

Figure 106. Terrestrial range and anthropogenic pressure for *Carollia castanea*. Maps illustrate: (left) Terrestrial Extent of Occurrence (EOO) shown in green with black dots representing occurrence records; (right) Areas of high human pressure (red) within the EOO, defined as regions exceeding the 70th percentile of the human footprint index. The caption below presents the total terrestrial area and the proportion experiencing intense human activity. Detailed methodology for calculating terrestrial EOO and human footprint percentage is described in **Supplementary Text 2** and the **main text.**

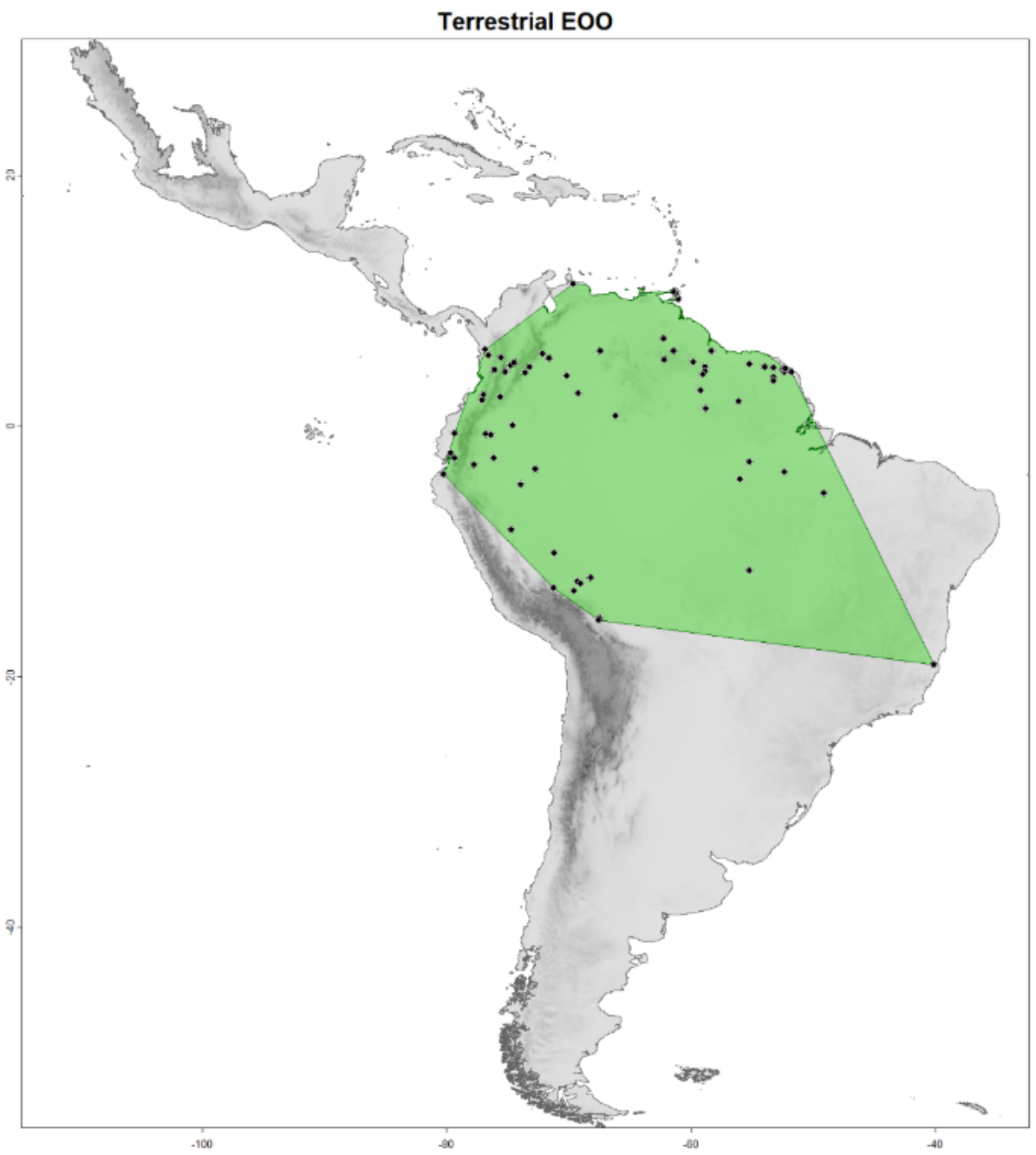

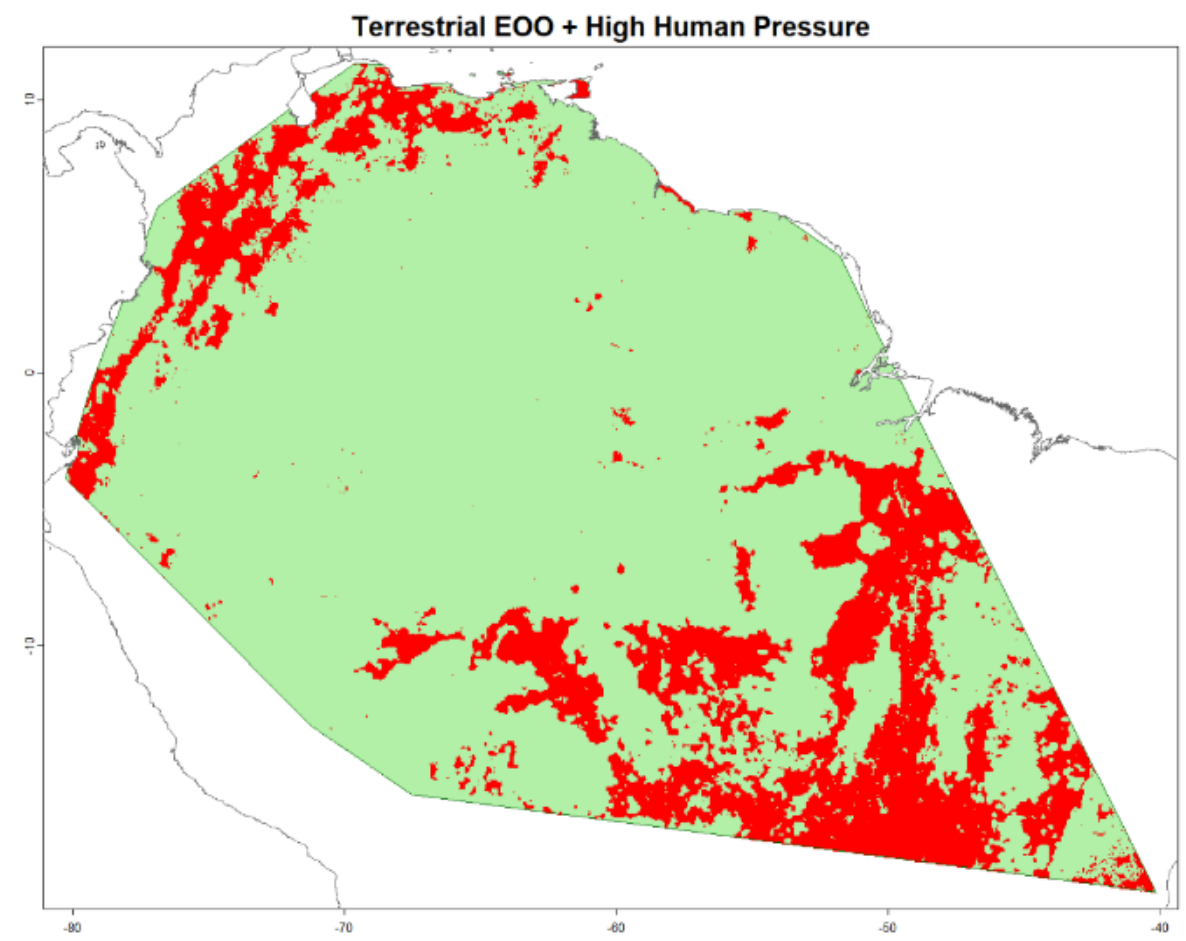

Total terrestrial area: 8991000 km² | High pressure: 1771653 km² (19.7 %)

Figure 107. Terrestrial range and anthropogenic pressure for *Choeroniscus minor*. Maps illustrate: (left) Terrestrial Extent of Occurrence (EOO) shown in green with black dots representing occurrence records; (right) Areas of high human pressure (red) within the EOO, defined as regions exceeding the 70th percentile of the human footprint index. The caption below presents the total terrestrial area and the proportion experiencing intense human activity. Detailed methodology for calculating terrestrial EOO and human footprint percentage is described in **Supplementary Text 2** and the **main text.**

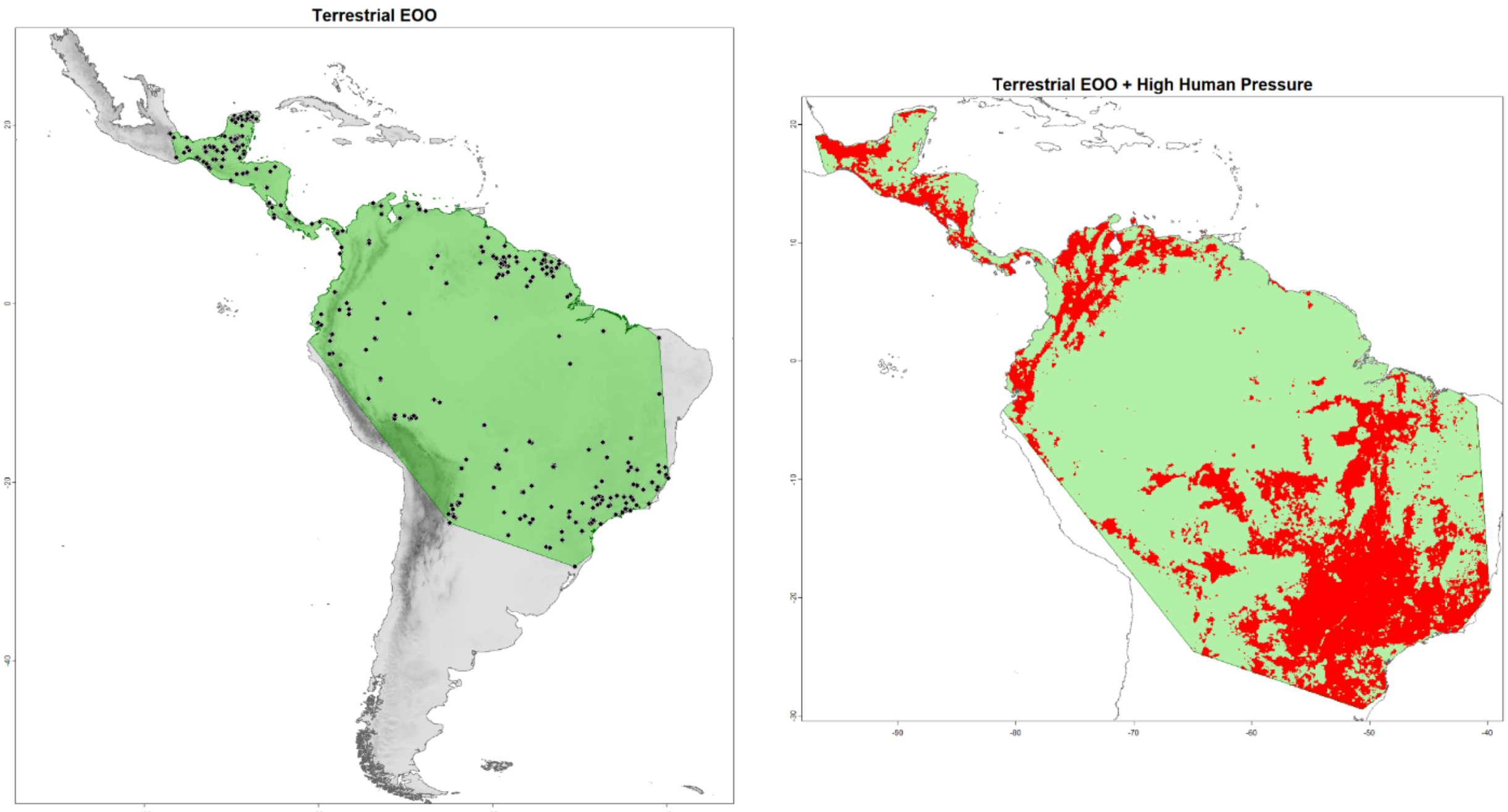

Total terrestrial area: 14044374 km² | High pressure: 3909729 km² (27.84 %)

Figure 108. Terrestrial range and anthropogenic pressure for *Chrotopterus auritus*. Maps illustrate: (left) Terrestrial Extent of Occurrence (EOO) shown in green with black dots representing occurrence records; (right) Areas of high human pressure (red) within the EOO, defined as regions exceeding the 70th percentile of the human footprint index. The caption below presents the total terrestrial area and the proportion experiencing intense human activity. Detailed methodology for calculating terrestrial EOO and human footprint percentage is described in **Supplementary Text 2** and the **main text.**

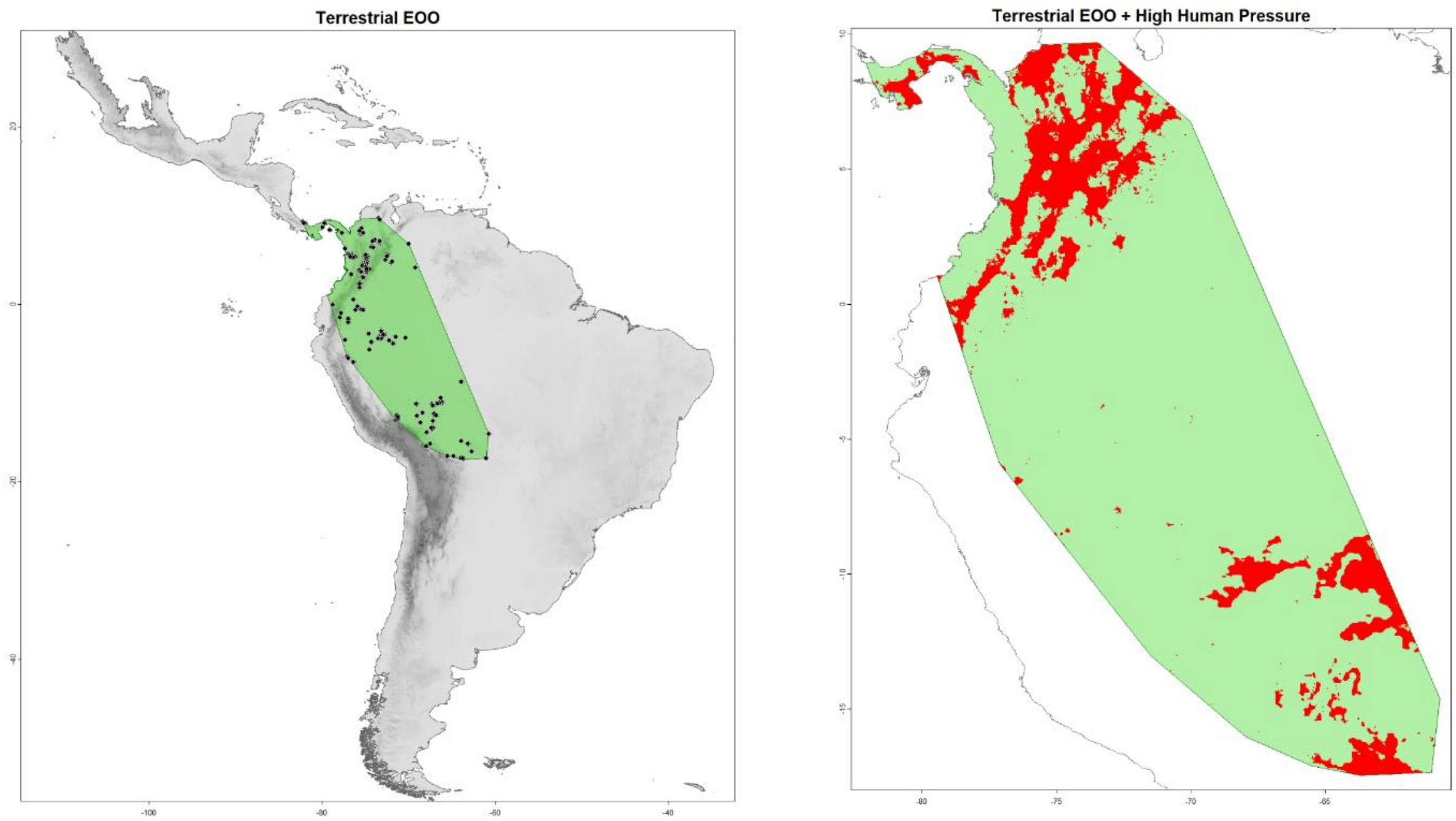

Total terrestrial area: 3297591 km² | High pressure: 443622 km² (13.45 %)

Figure 109. Terrestrial range and anthropogenic pressure for *Dermanura anderseni*. Maps illustrate: (left) Terrestrial Extent of Occurrence (EOO) shown in green with black dots representing occurrence records; (right) Areas of high human pressure (red) within the EOO, defined as regions exceeding the 70th percentile of the human footprint index. The caption below presents the total terrestrial area and the proportion experiencing intense human activity. Detailed methodology for calculating terrestrial EOO and human footprint percentage is described in **Supplementary Text 2** and the **main text.**

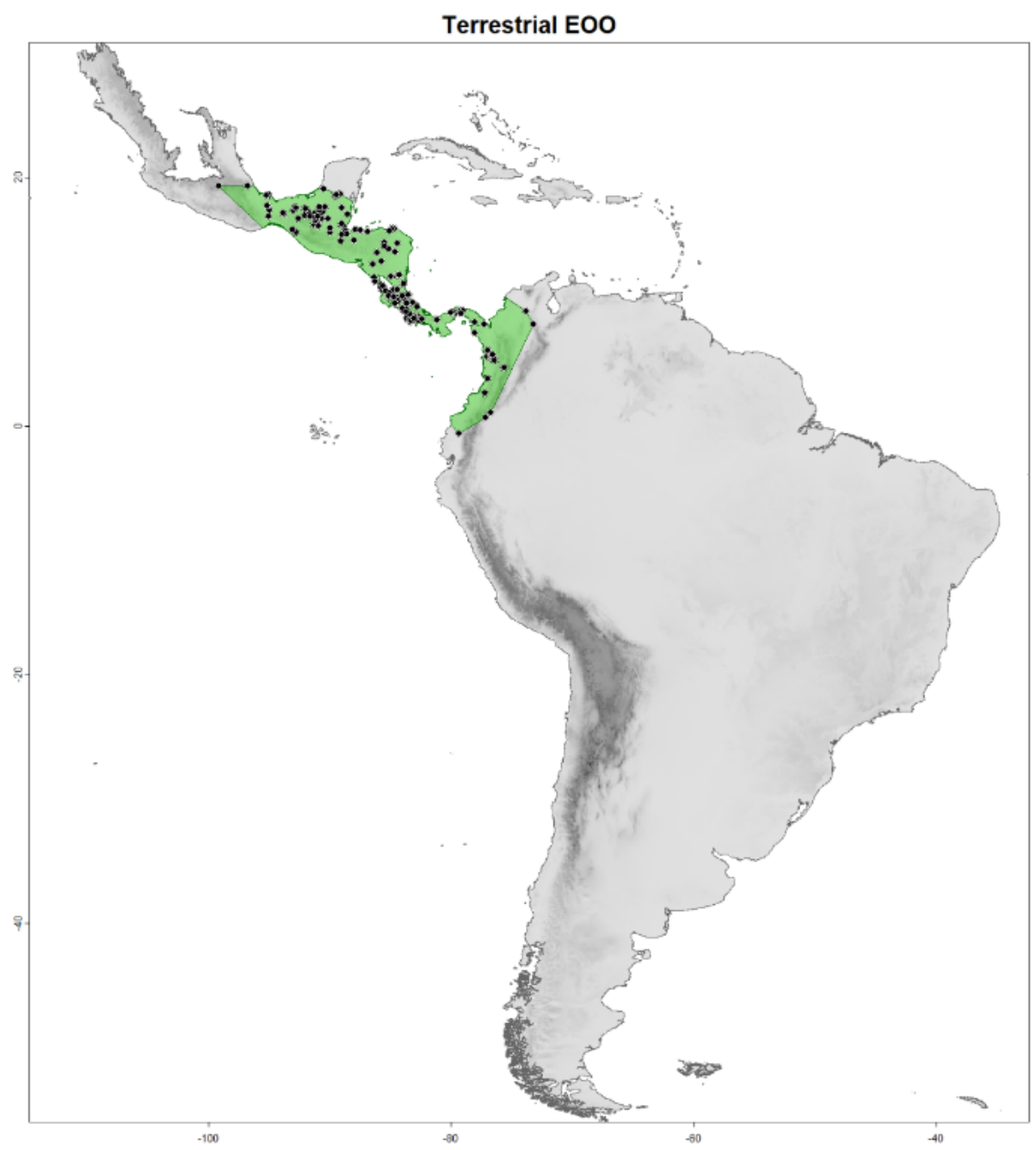

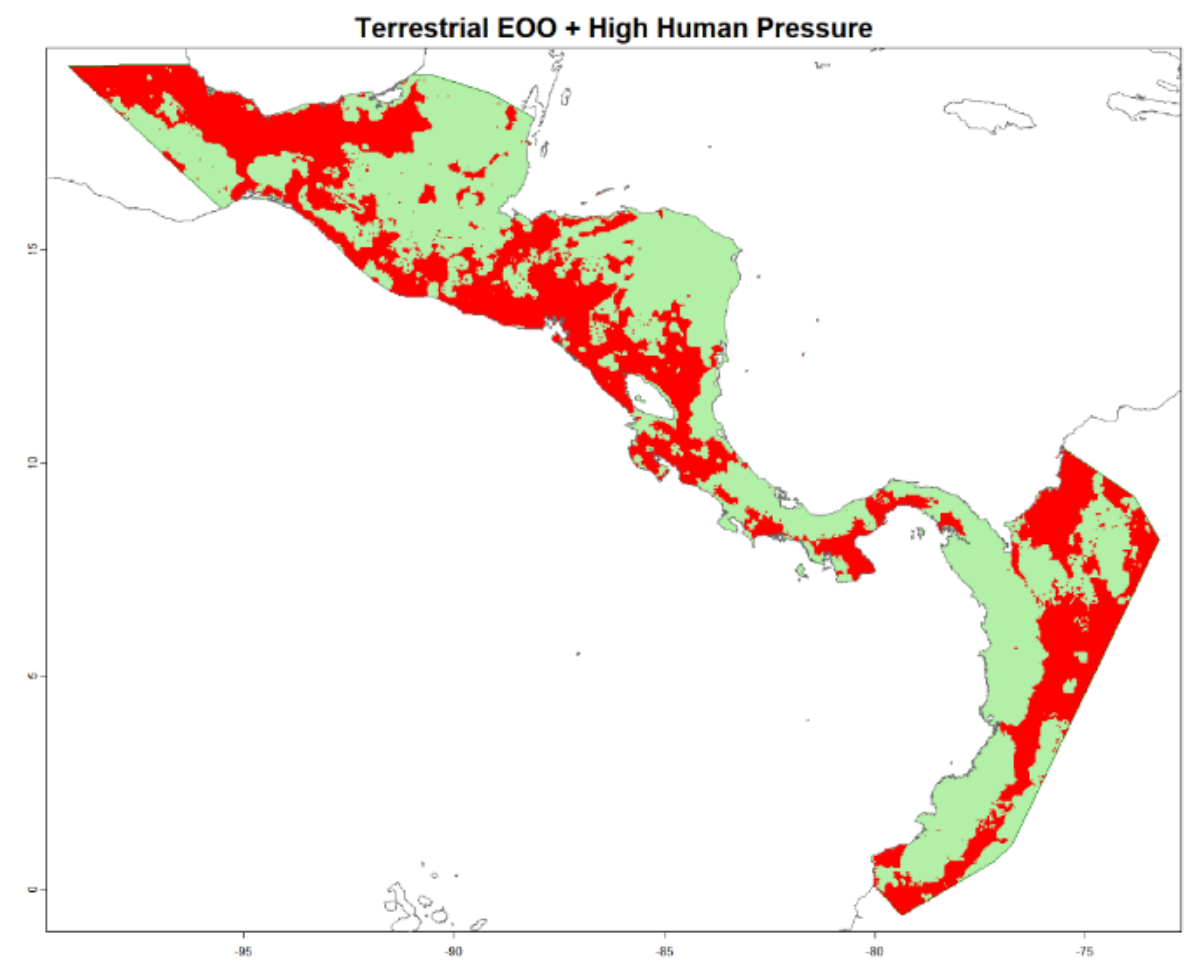

Total terrestrial area: 1084939 km² | High pressure: 469242 km² (43.25 %)

Figure 110. Terrestrial range and anthropogenic pressure for *Dermanura watsoni*. Maps illustrate: (left) Terrestrial Extent of Occurrence (EOO) shown in green with black dots representing occurrence records; (right) Areas of high human pressure (red) within the EOO, defined as regions exceeding the 70th percentile of the human footprint index. The caption below presents the total terrestrial area and the proportion experiencing intense human activity. Detailed methodology for calculating terrestrial EOO and human footprint percentage is described in **Supplementary Text 2** and the **main text.**

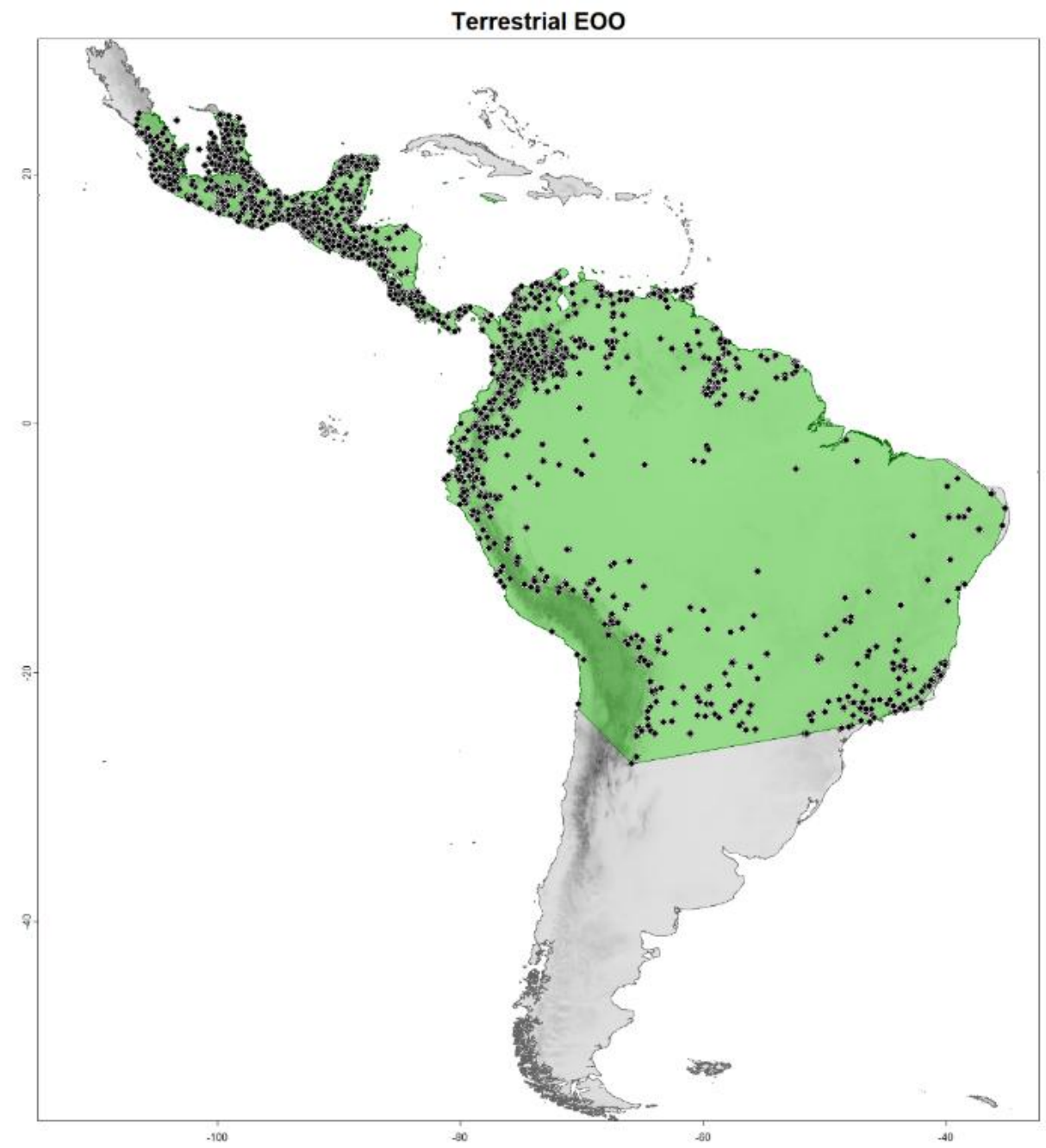

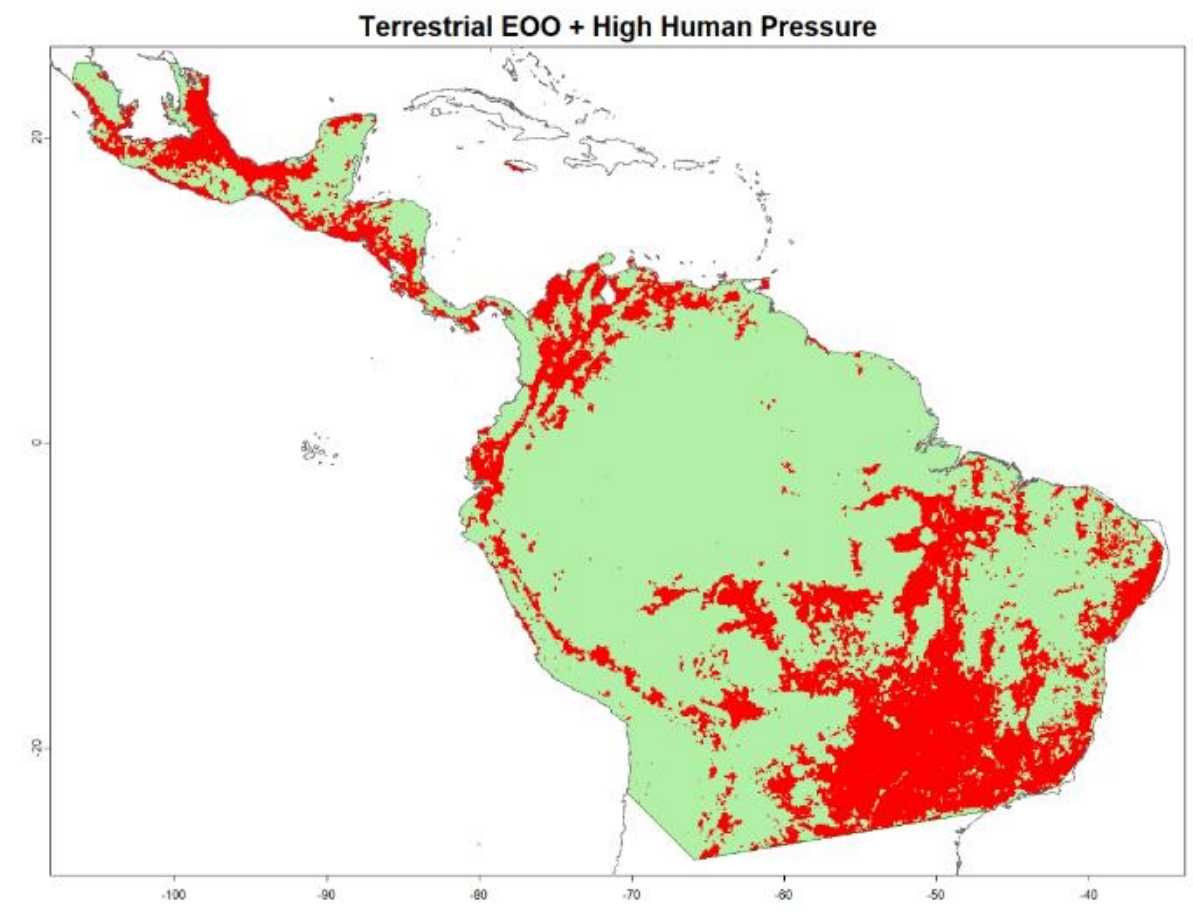

Total terrestrial area: 15337934 km² | High pressure: 4274038 km² (27.87 %)

Figure 111. Terrestrial range and anthropogenic pressure for *Desmodus rotundus*. Maps illustrate: (left) Terrestrial Extent of Occurrence (EOO) shown in green with black dots representing occurrence records; (right) Areas of high human pressure (red) within the EOO, defined as regions exceeding the 70th percentile of the human footprint index. The caption below presents the total terrestrial area and the proportion experiencing intense human activity. Detailed methodology for calculating terrestrial EOO and human footprint percentage is described in **Supplementary Text 2** and the **main text.**

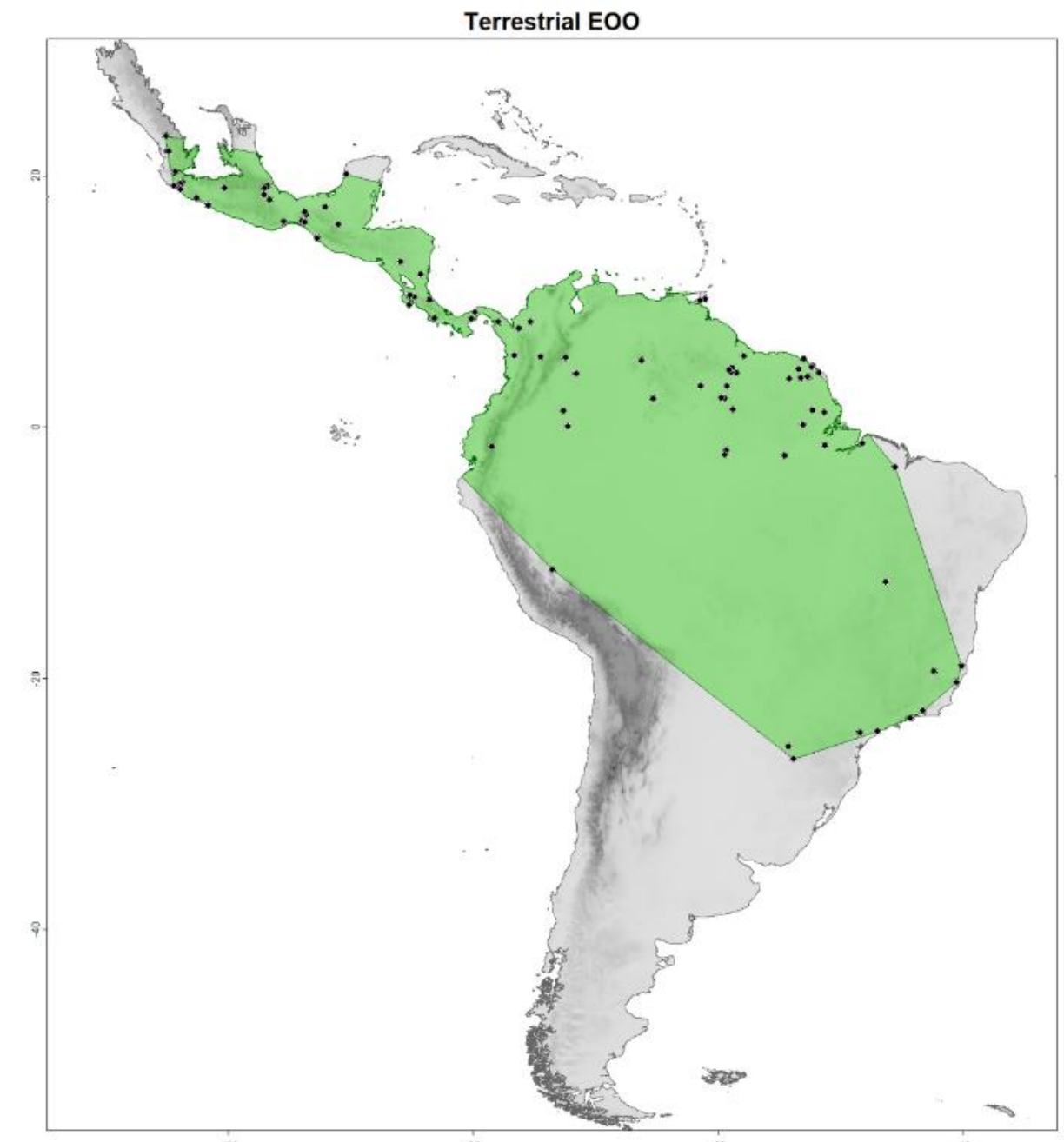

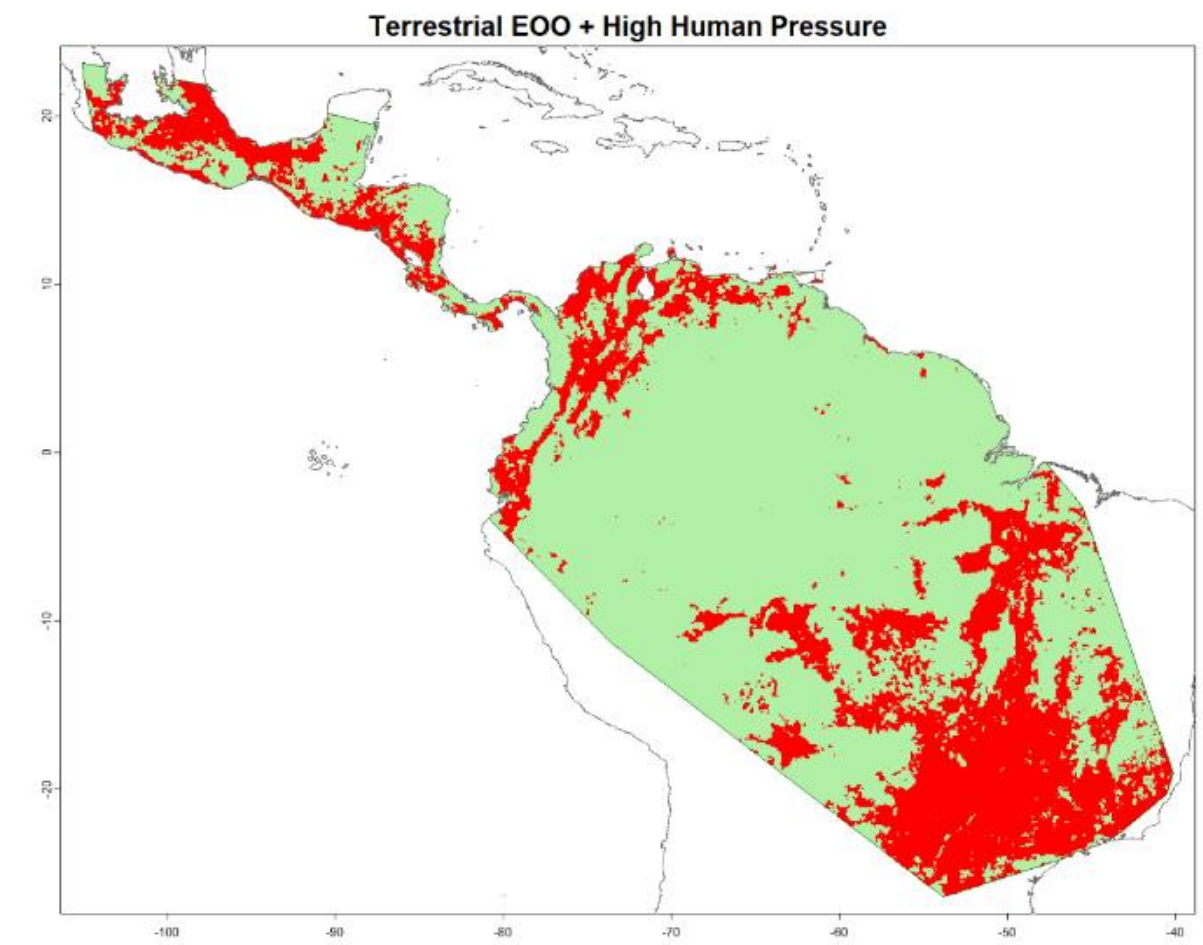

Total terrestrial area: 12487242 km² | High pressure: 3631504 km² (29.08 %)

Figure 112. Terrestrial range and anthropogenic pressure for *Glyphonycteris sylvestris*. Maps illustrate: (left) Terrestrial Extent of Occurrence (EOO) shown in green with black dots representing occurrence records; (right) Areas of high human pressure (red) within the EOO, defined as regions exceeding the 70th percentile of the human footprint index. The caption below presents the total terrestrial area and the proportion experiencing intense human activity. Detailed methodology for calculating terrestrial EOO and human footprint percentage is described in **Supplementary Text 2** and the **main text.**

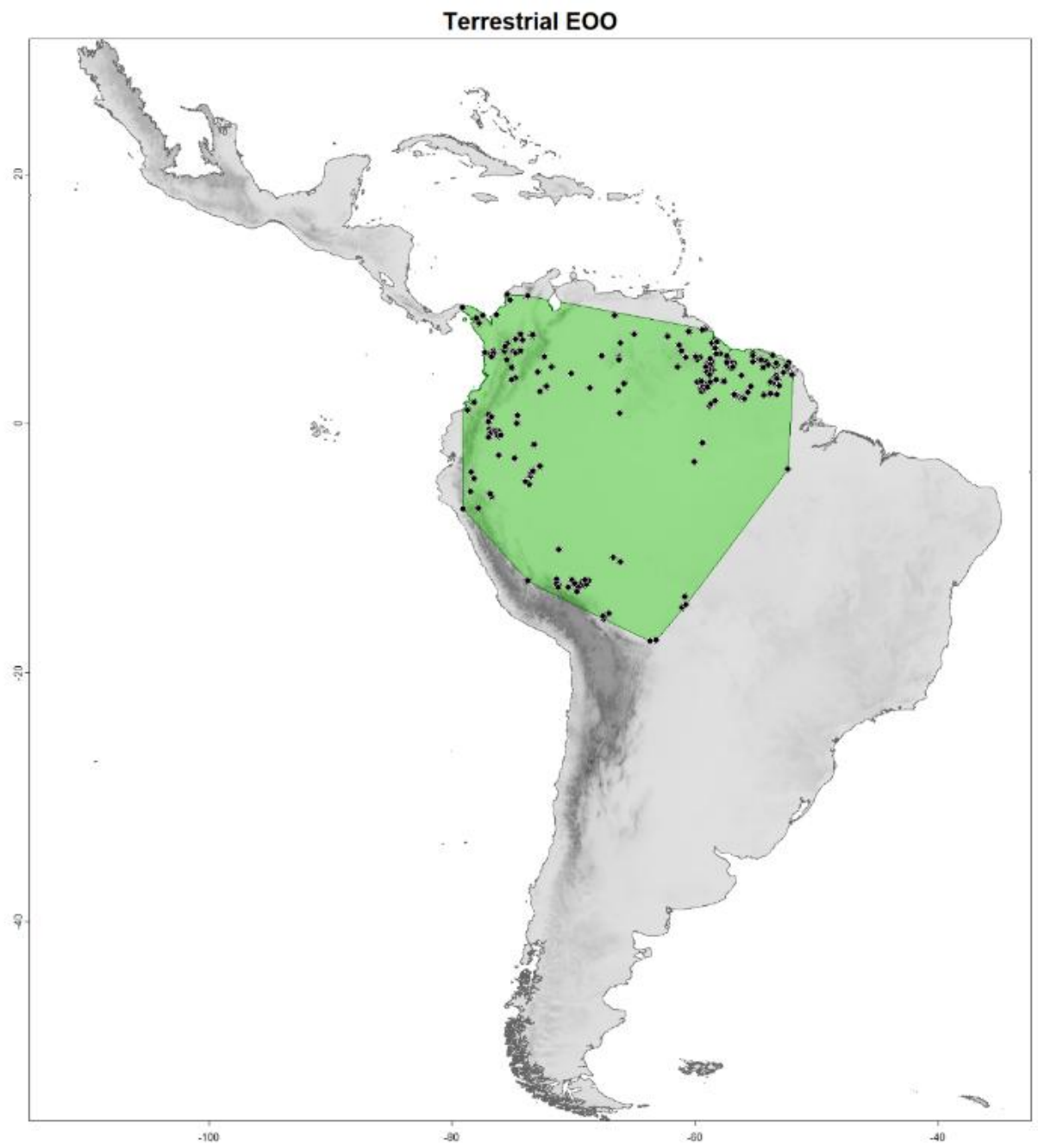

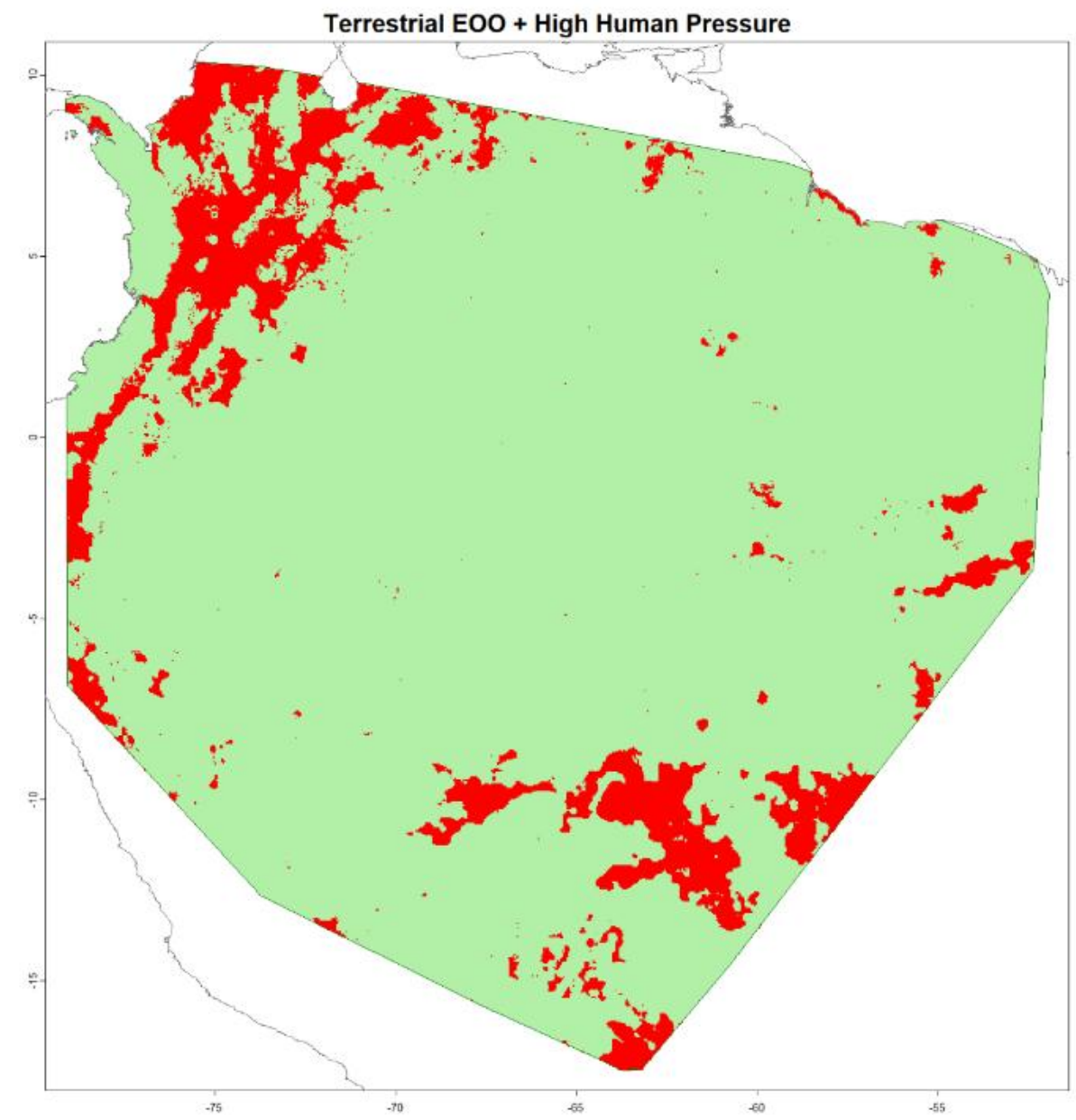

Total terrestrial area: 6637908 km² | High pressure: 691124 km² (10.41 %)

Figure 113. Terrestrial range and anthropogenic pressure for *Hsunycteris thomasi*. Maps illustrate: (left) Terrestrial Extent of Occurrence (EOO) shown in green with black dots representing occurrence records; (right) Areas of high human pressure (red) within the EOO, defined as regions exceeding the 70th percentile of the human footprint index. The caption below presents the total terrestrial area and the proportion experiencing intense human activity. Detailed methodology for calculating terrestrial EOO and human footprint percentage is described in **Supplementary Text 2** and the **main text.**

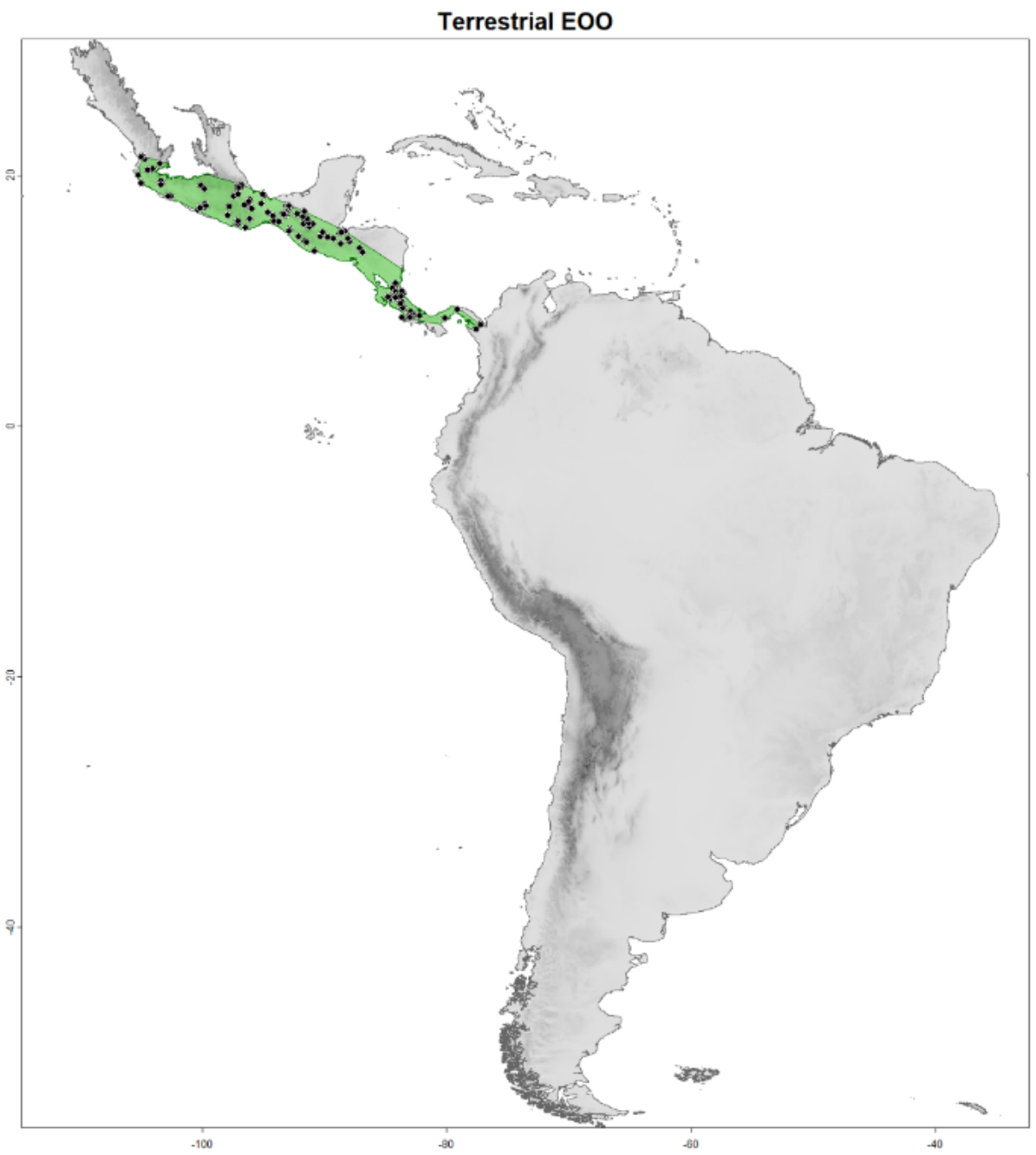

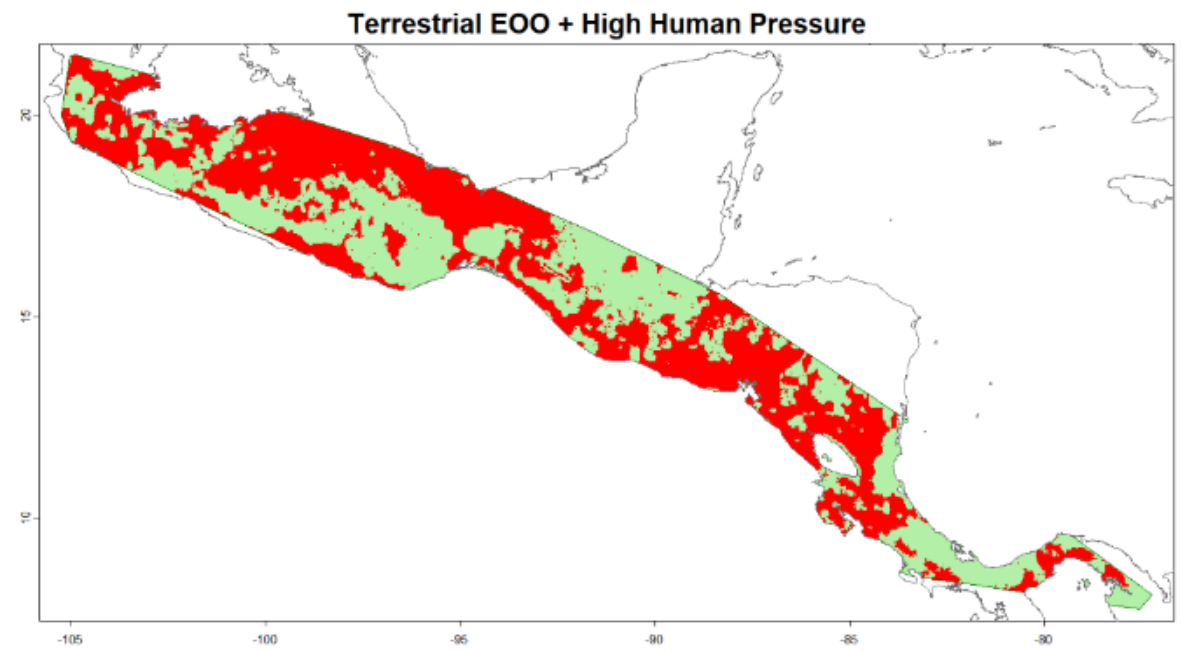

Total terrestrial area: 740184 km² | High pressure: 402243 km² (54.34 %)

Figure 114. Terrestrial range and anthropogenic pressure for *Hylonycteris underwoodi*. Maps illustrate: (left) Terrestrial Extent of Occurrence (EOO) shown in green with black dots representing occurrence records; (right) Areas of high human pressure (red) within the EOO, defined as regions exceeding the 70th percentile of the human footprint index. The caption below presents the total terrestrial area and the proportion experiencing intense human activity. Detailed methodology for calculating terrestrial EOO and human footprint percentage is described in **Supplementary Text 2** and the **main text.**

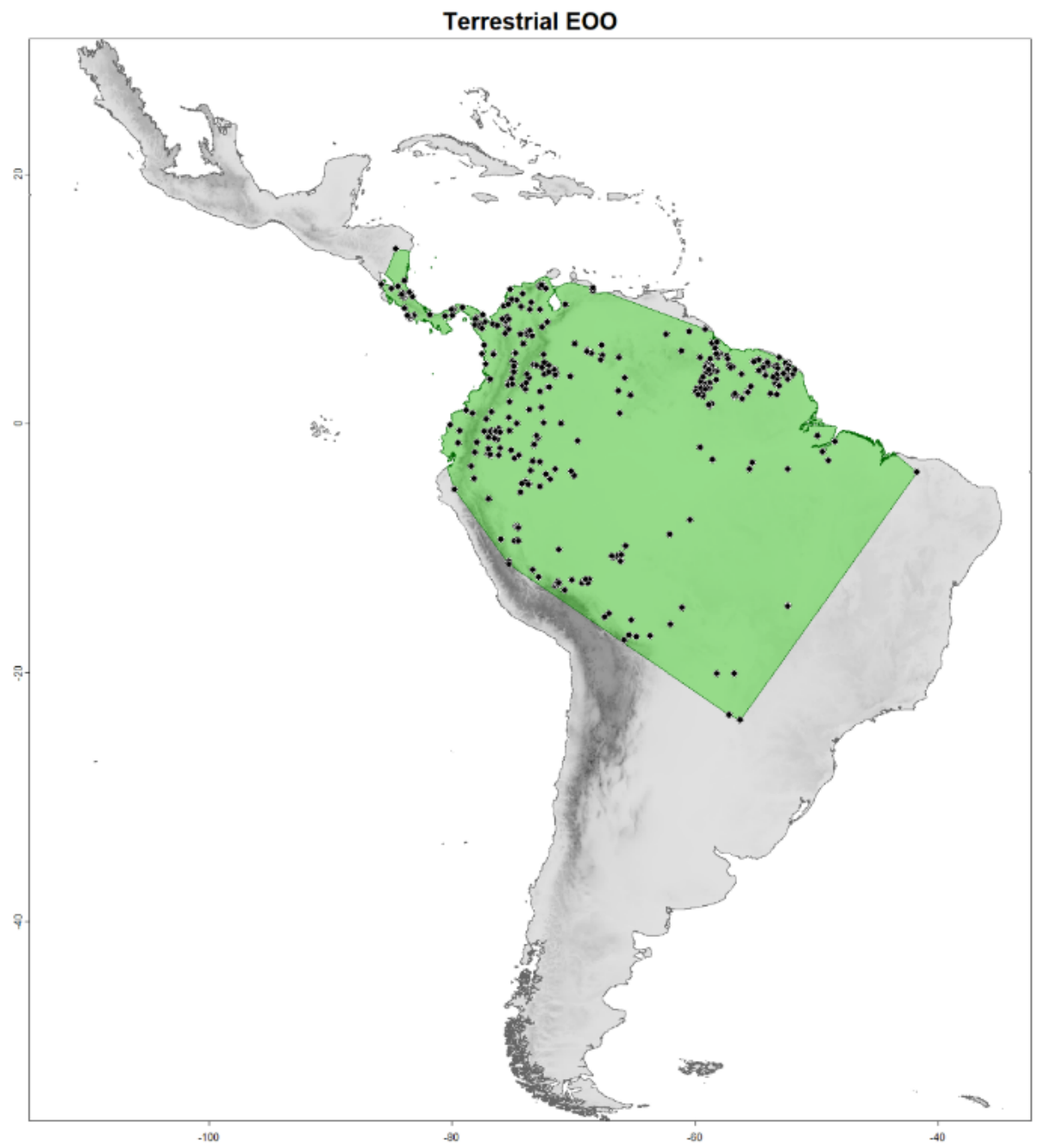

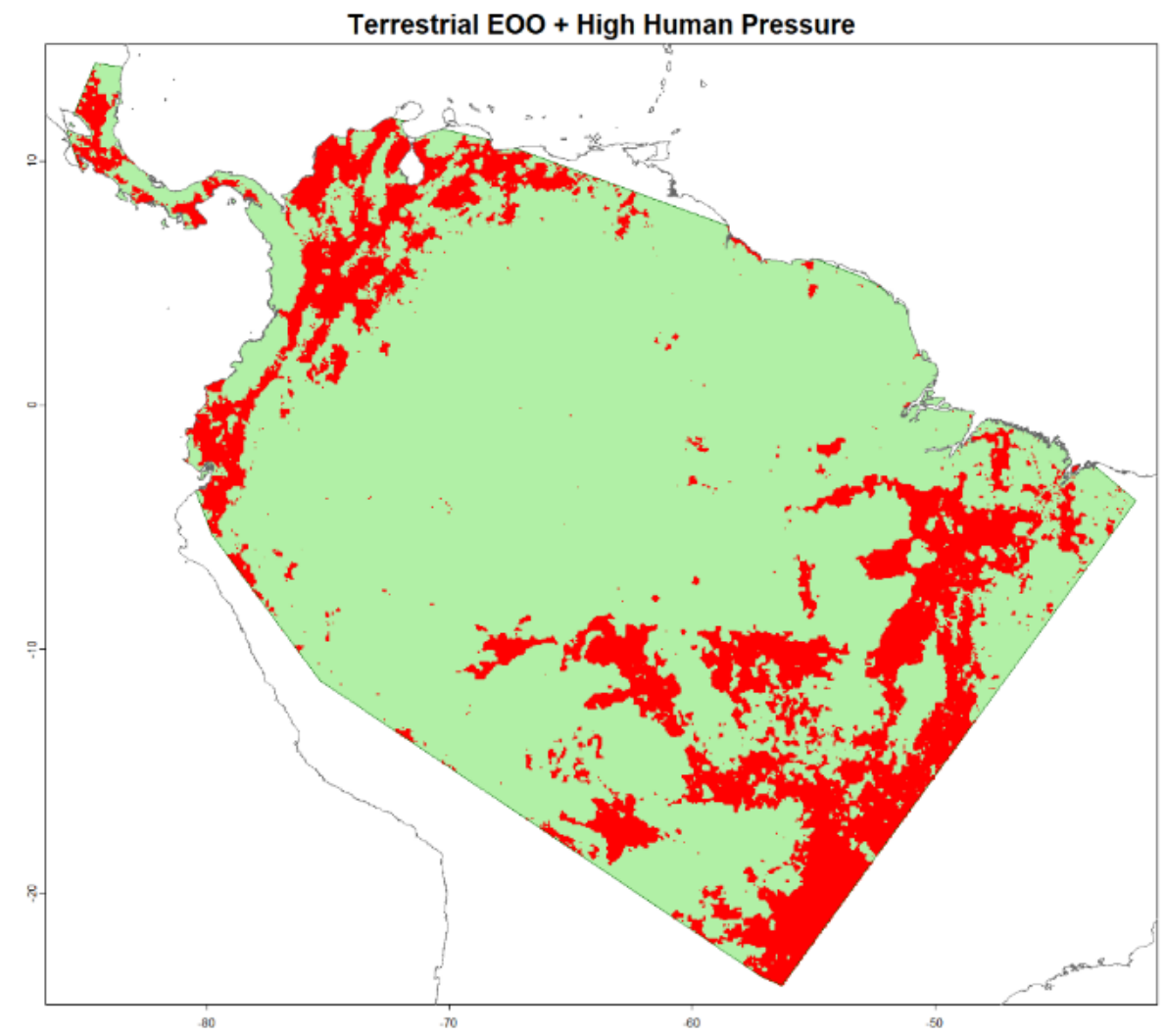

Total terrestrial area: 10024190 km² | High pressure: 2038905 km² (20.34 %)

Figure 115. Terrestrial range and anthropogenic pressure for *Lophostoma silvicola*. Maps illustrate: (left) Terrestrial Extent of Occurrence (EOO) shown in green with black dots representing occurrence records; (right) Areas of high human pressure (red) within the EOO, defined as regions exceeding the 70th percentile of the human footprint index. The caption below presents the total terrestrial area and the proportion experiencing intense human activity. Detailed methodology for calculating terrestrial EOO and human footprint percentage is described in **Supplementary Text 2** and the **main text.**

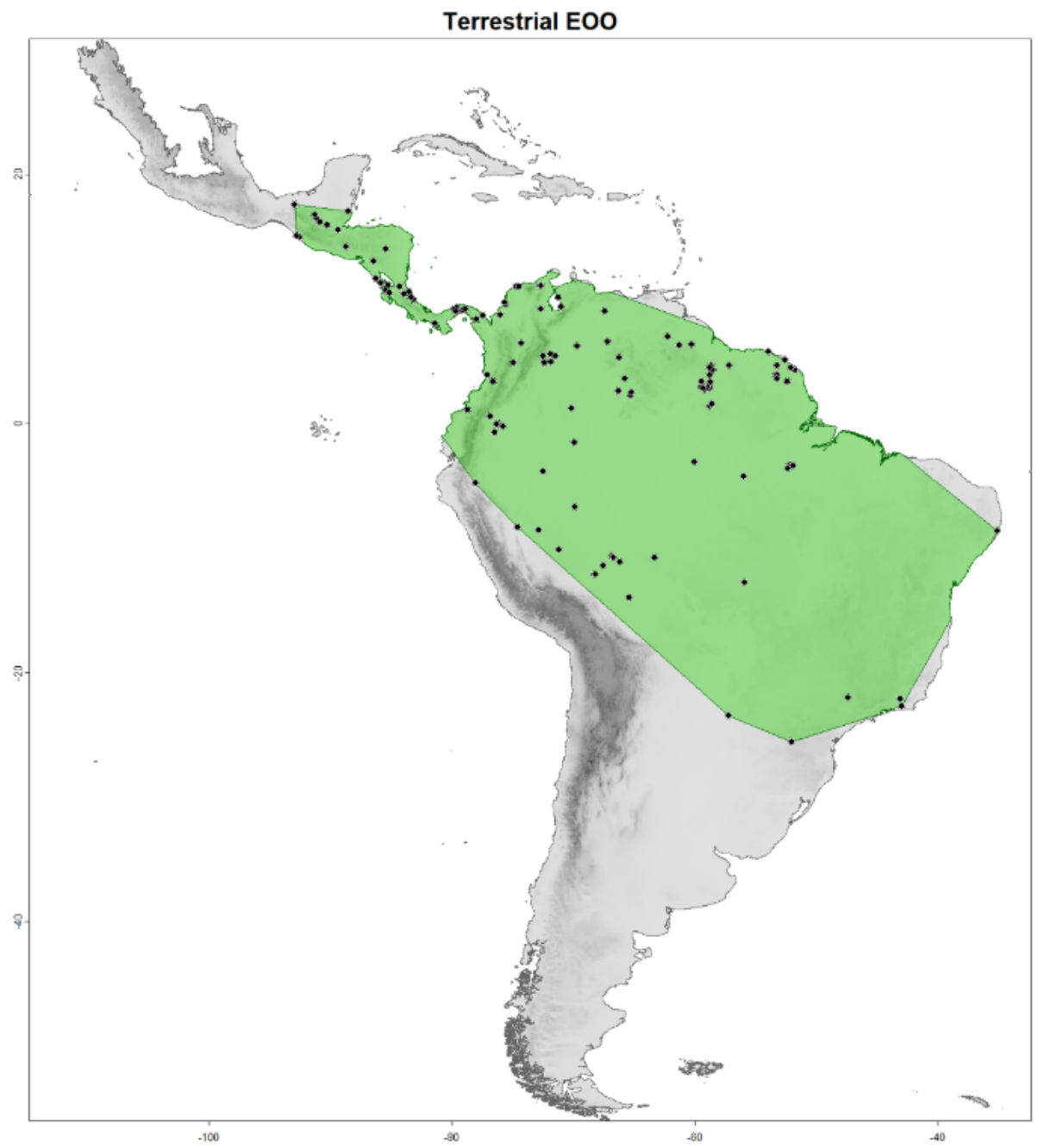

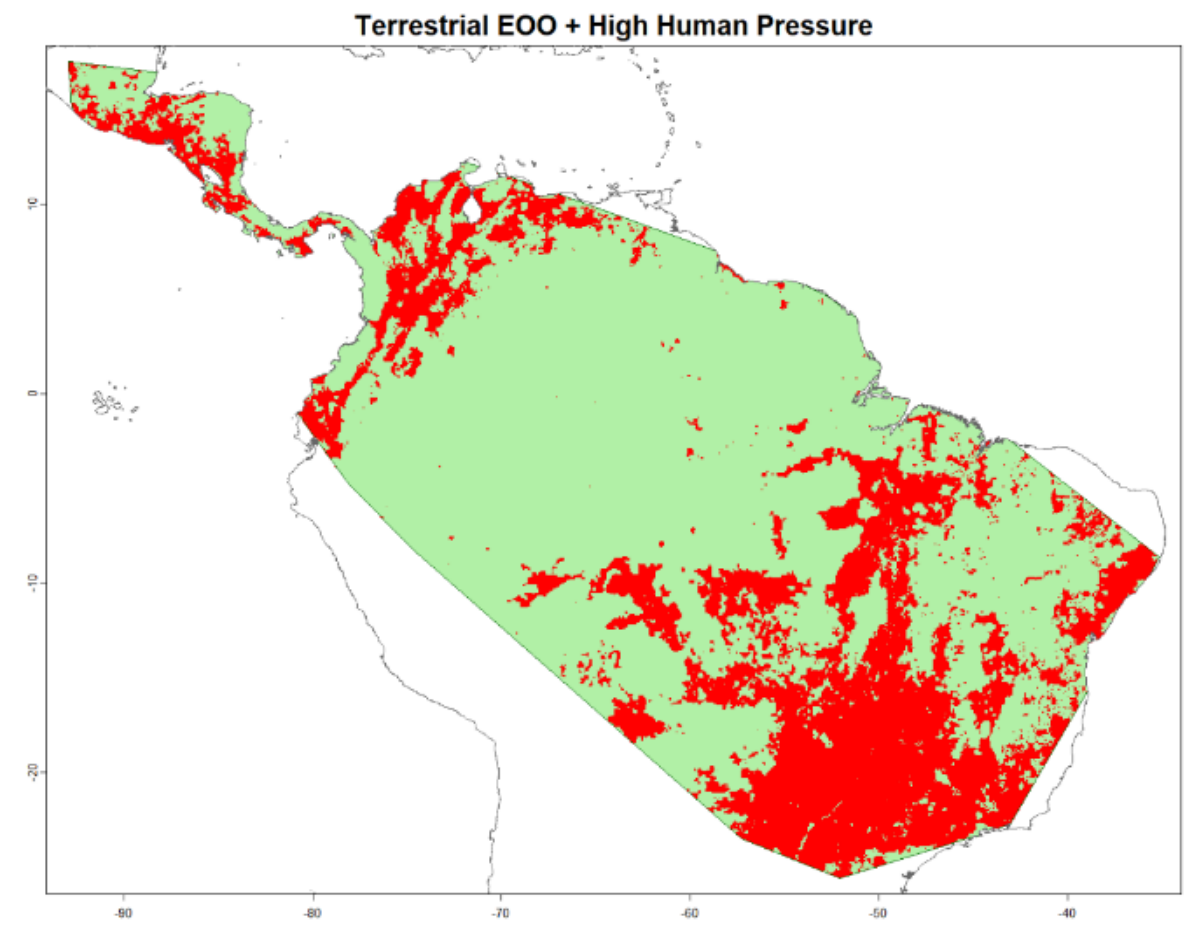

Total terrestrial area: 12181080 km² | High pressure: 3426967 km² (28.13 %)

Figure 116. Terrestrial range and anthropogenic pressure for *Macrophyllum macrophyllum*. Maps illustrate: (left) Terrestrial Extent of Occurrence (EOO) shown in green with black dots representing occurrence records; (right) Areas of high human pressure (red) within the EOO, defined as regions exceeding the 70th percentile of the human footprint index. The caption below presents the total terrestrial area and the proportion experiencing intense human activity. Detailed methodology for calculating terrestrial EOO and human footprint percentage is described in **Supplementary Text 2** and the **main text.**

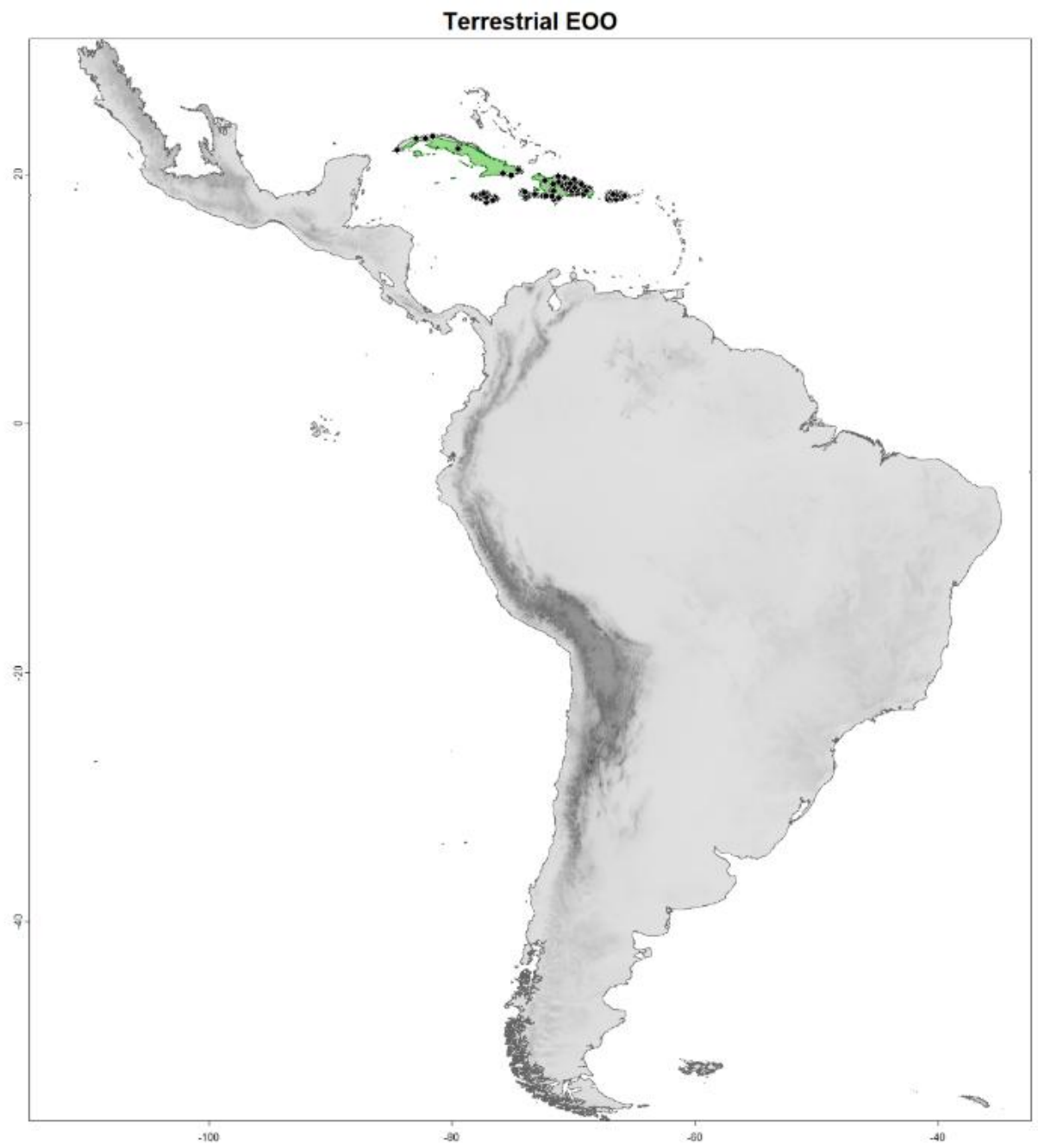

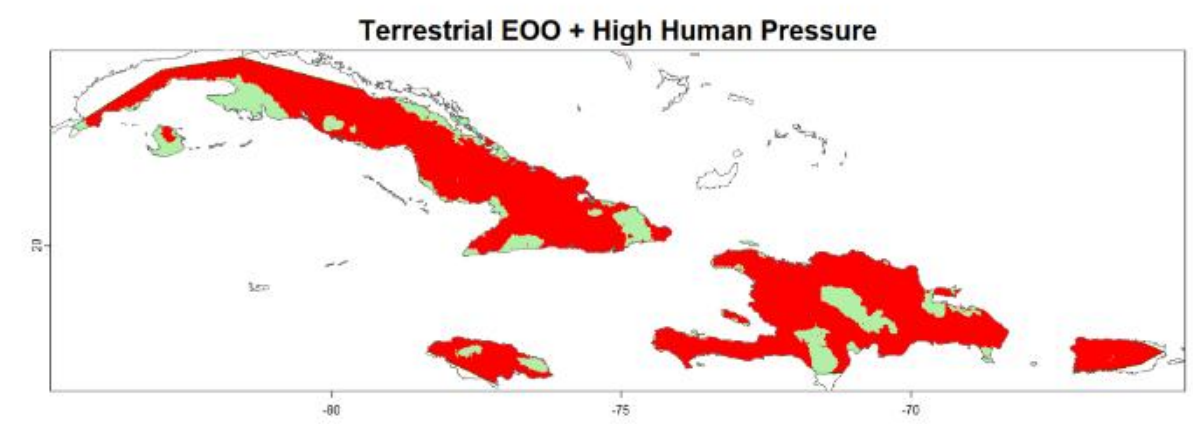

Total terrestrial area: 186743 km² | High pressure: 154504 km² (82.74 %)

Figure 117. Terrestrial range and anthropogenic pressure for *Monophyllus redmani*. Maps illustrate: (left) Terrestrial Extent of Occurrence (EOO) shown in green with black dots representing occurrence records; (right) Areas of high human pressure (red) within the EOO, defined as regions exceeding the 70th percentile of the human footprint index. The caption below presents the total terrestrial area and the proportion experiencing intense human activity. Detailed methodology for calculating terrestrial EOO and human footprint percentage is described in **Supplementary Text 2** and the **main text.**

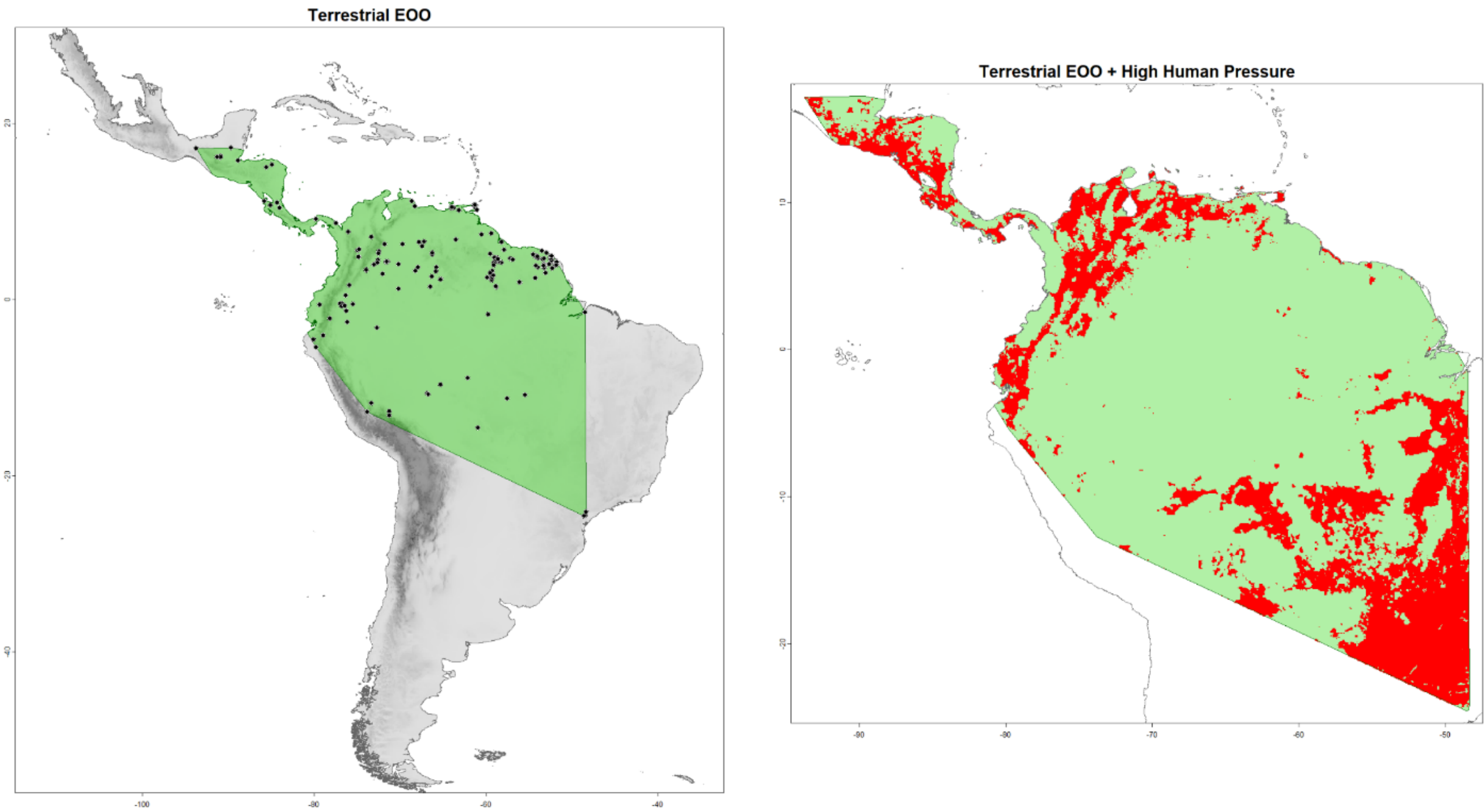

Total terrestrial area: 10070844 km² | High pressure: 2362815 km² (23.46 %)

Figure 118. Terrestrial range and anthropogenic pressure for *Phylloderma stenops*. Maps illustrate: (left) Terrestrial Extent of Occurrence (EOO) shown in green with black dots representing occurrence records; (right) Areas of high human pressure (red) within the EOO, defined as regions exceeding the 70th percentile of the human footprint index. The caption below presents the total terrestrial area and the proportion experiencing intense human activity. Detailed methodology for calculating terrestrial EOO and human footprint percentage is described in **Supplementary Text 2** and the **main text.**

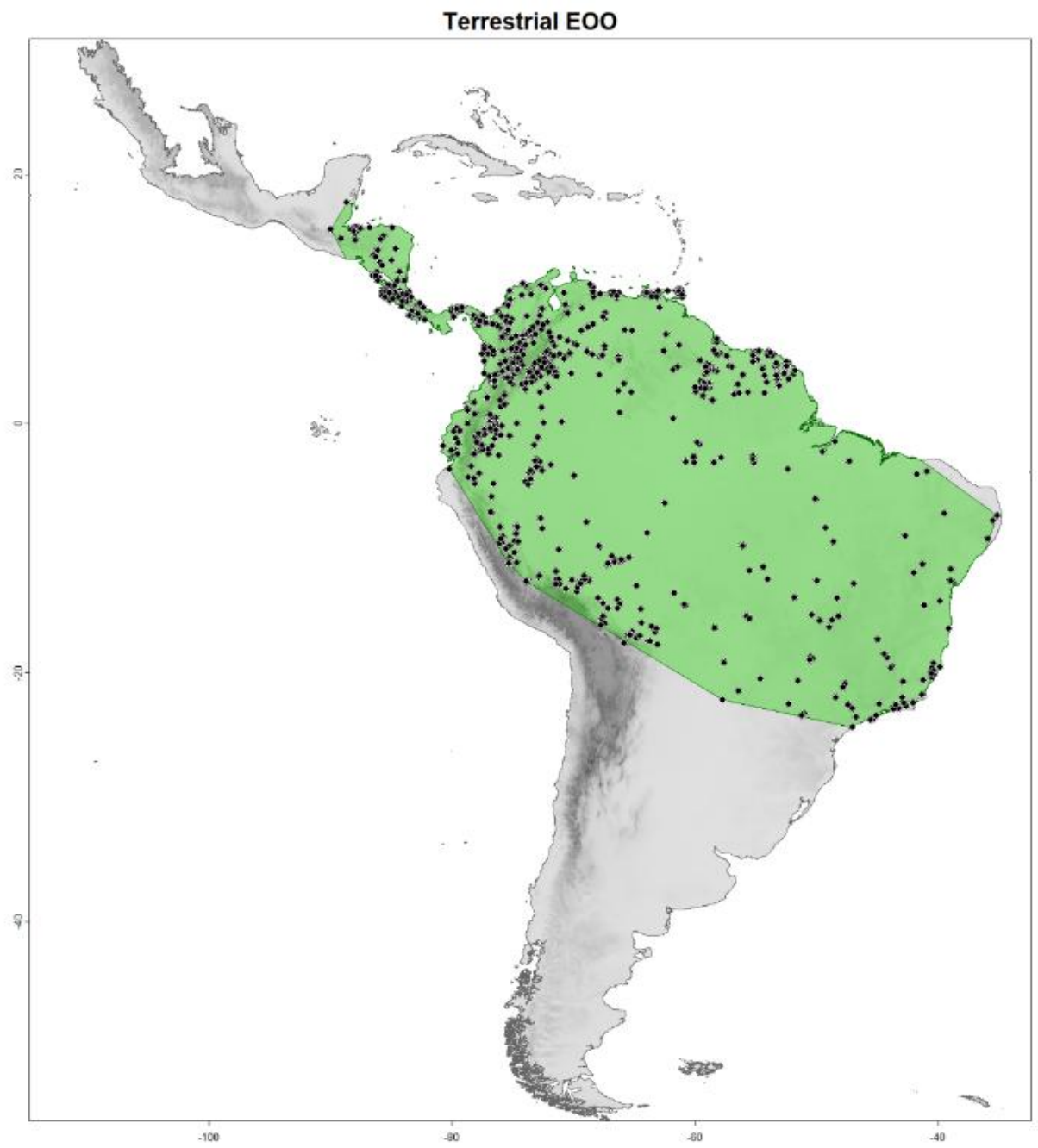

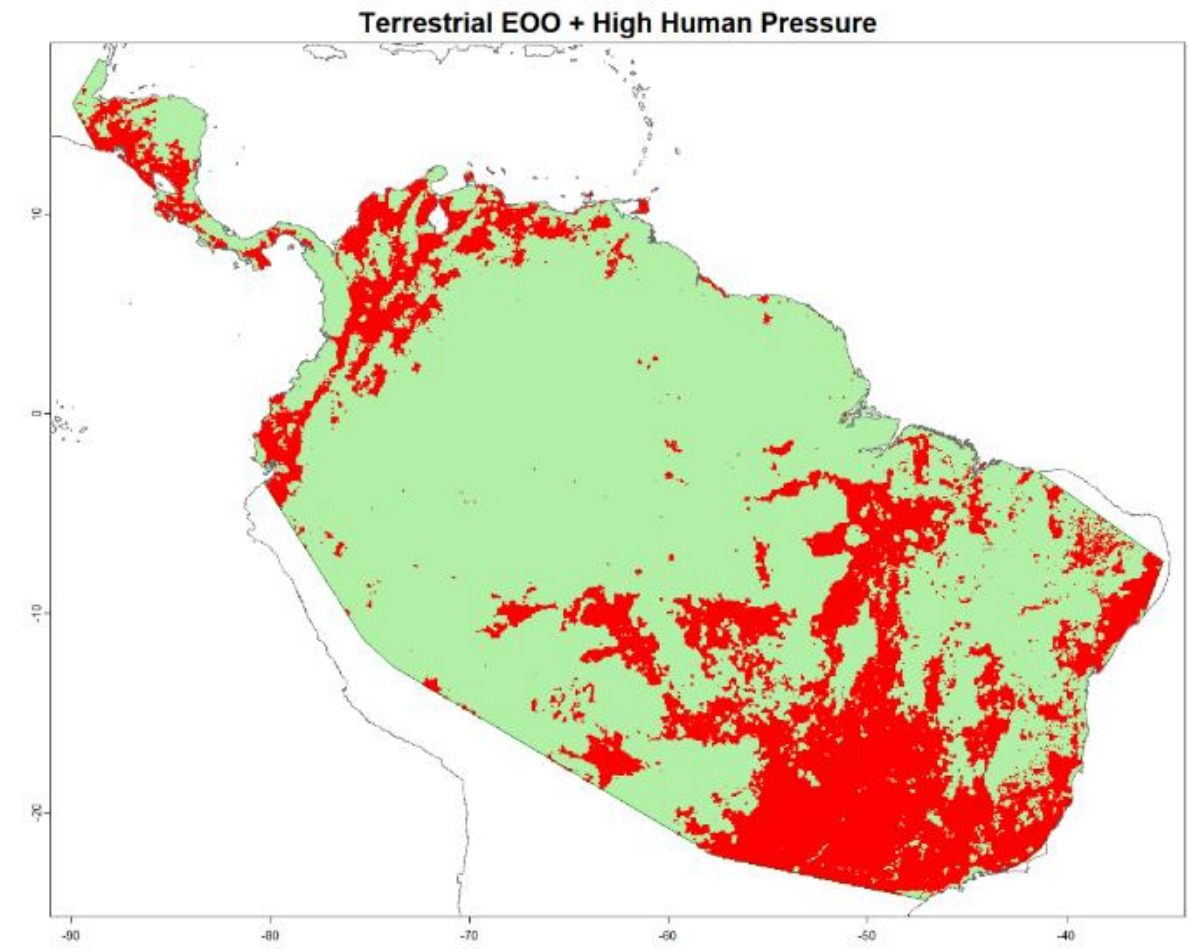

Total terrestrial area: 12604866 km² | High pressure: 3386555 km² (26.87 %)

Figure 119. Terrestrial range and anthropogenic pressure for *Phyllostomus hastatus*. Maps illustrate: (left) Terrestrial Extent of Occurrence (EOO) shown in green with black dots representing occurrence records; (right) Areas of high human pressure (red) within the EOO, defined as regions exceeding the 70th percentile of the human footprint index. The caption below presents the total terrestrial area and the proportion experiencing intense human activity. Detailed methodology for calculating terrestrial EOO and human footprint percentage is described in **Supplementary Text 2** and the **main text.**

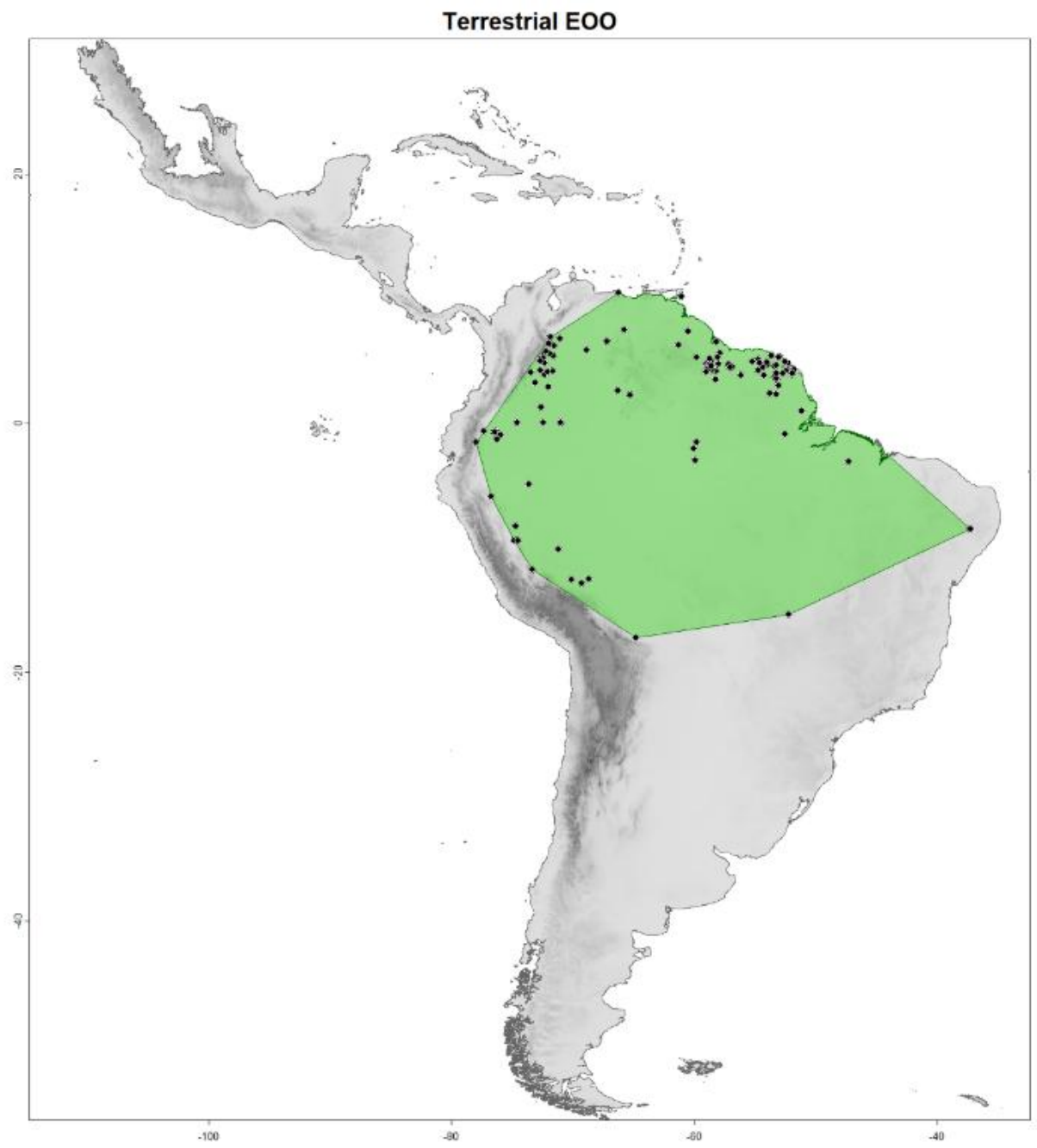

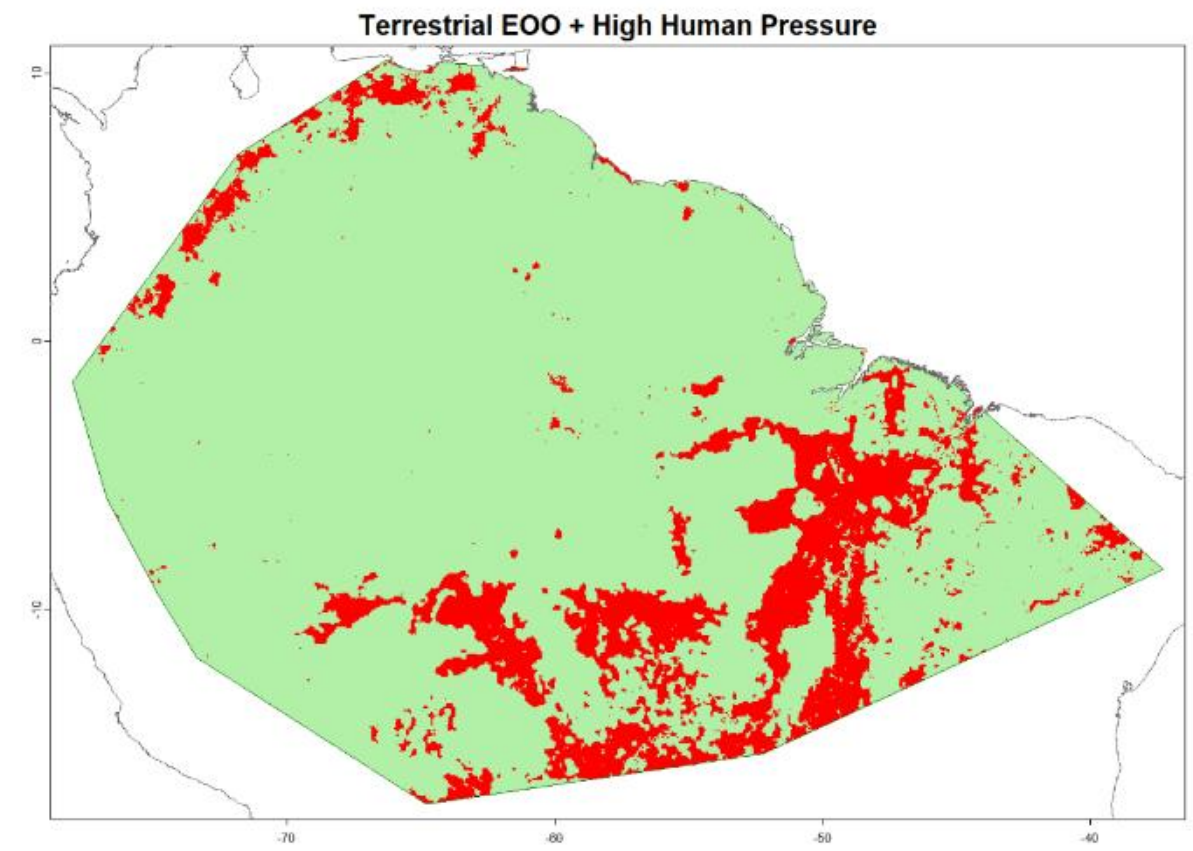

Total terrestrial area: 8350575 km² | High pressure: 1182442 km² (14.16 %)

Figure 120. Terrestrial range and anthropogenic pressure for *Tonatia maresi*. Maps illustrate: (left) Terrestrial Extent of Occurrence (EOO) shown in green with black dots representing occurrence records; (right) Areas of high human pressure (red) within the EOO, defined as regions exceeding the 70th percentile of the human footprint index. The caption below presents the total terrestrial area and the proportion experiencing intense human activity. Detailed methodology for calculating terrestrial EOO and human footprint percentage is described in **Supplementary Text 2** and the **main text.**

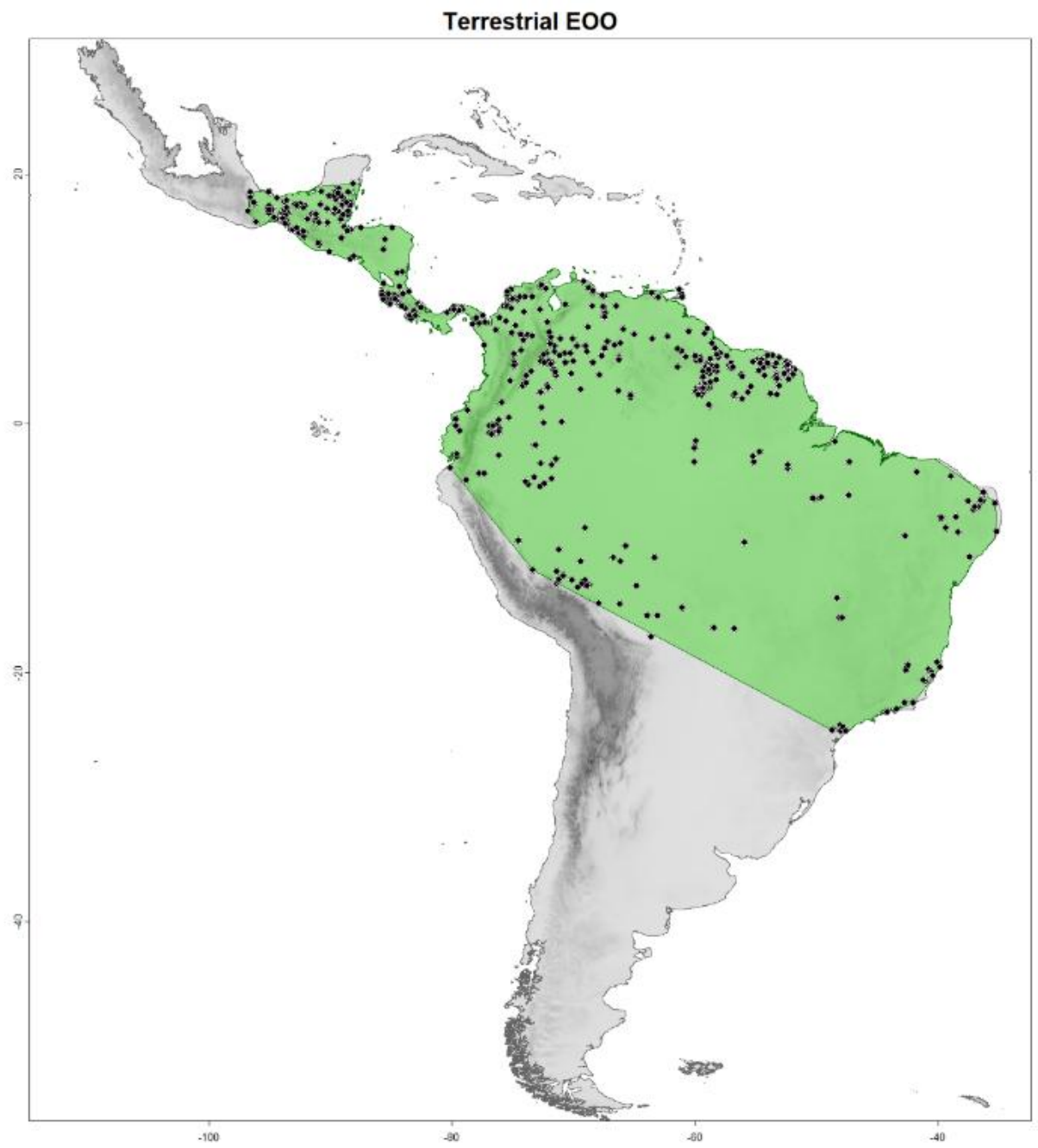

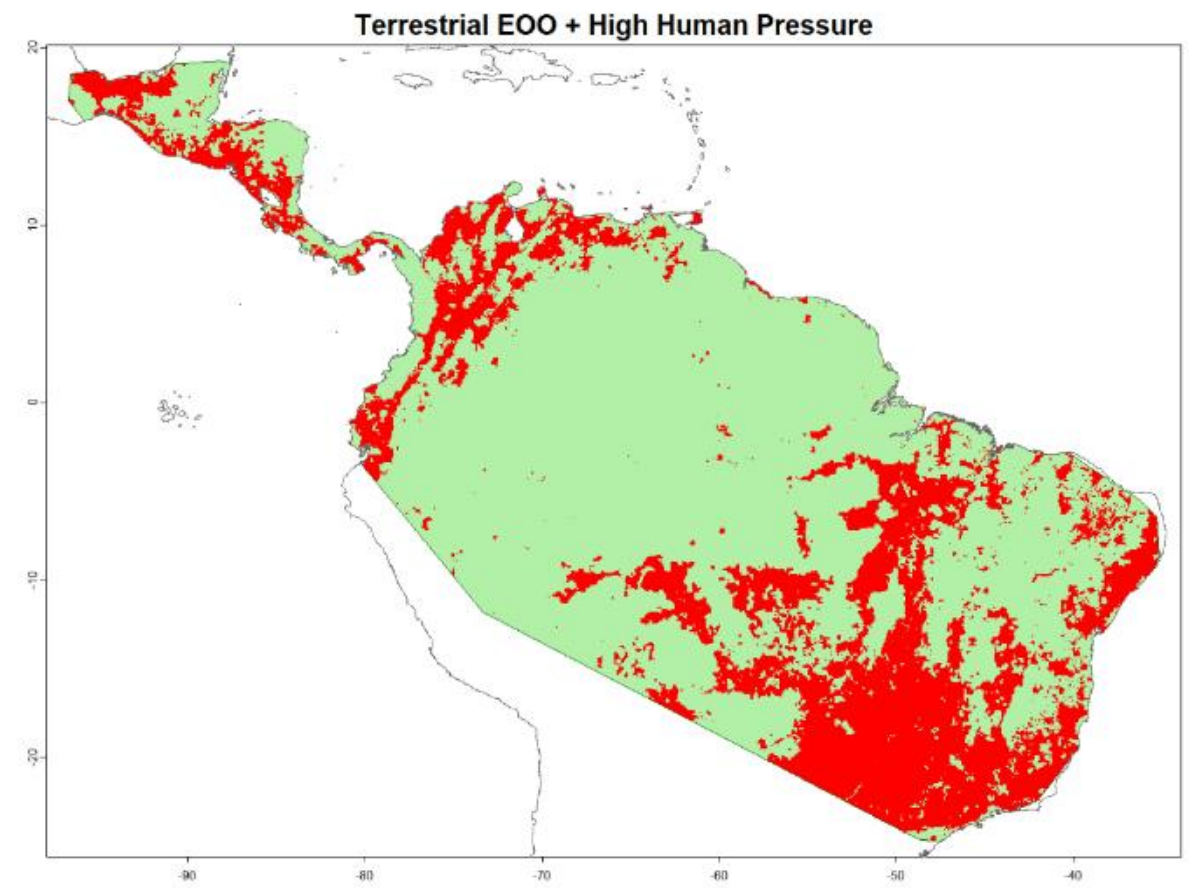

Total terrestrial area: 12594206 km² | High pressure: 3414162 km² (27.11 %)

Figure 121. Terrestrial range and anthropogenic pressure for *Trachops cirrhosus*. Maps illustrate: (left) Terrestrial Extent of Occurrence (EOO) shown in green with black dots representing occurrence records; (right) Areas of high human pressure (red) within the EOO, defined as regions exceeding the 70th percentile of the human footprint index. The caption below presents the total terrestrial area and the proportion experiencing intense human activity. Detailed methodology for calculating terrestrial EOO and human footprint percentage is described in **Supplementary Text 2** and the **main text.**

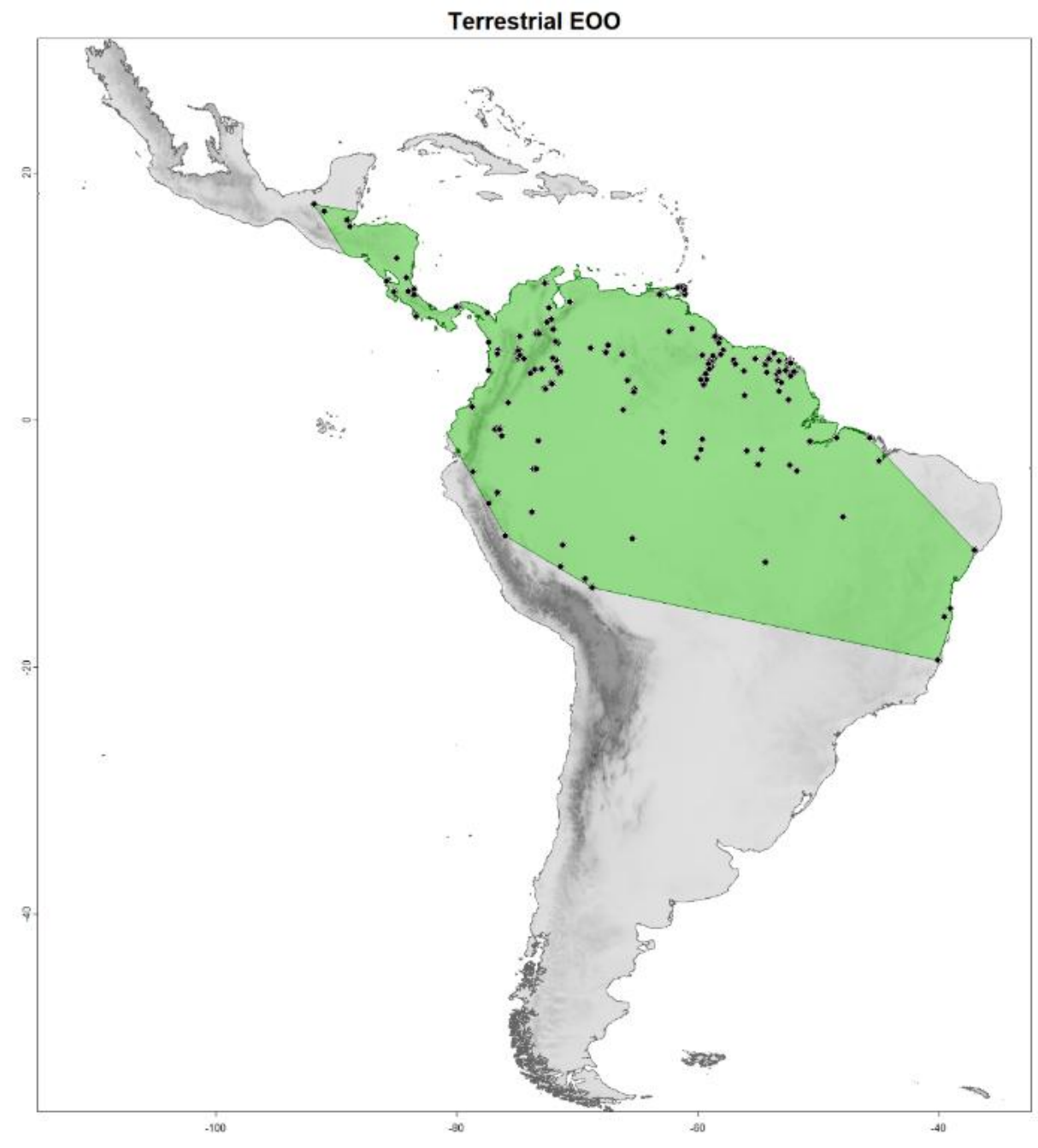

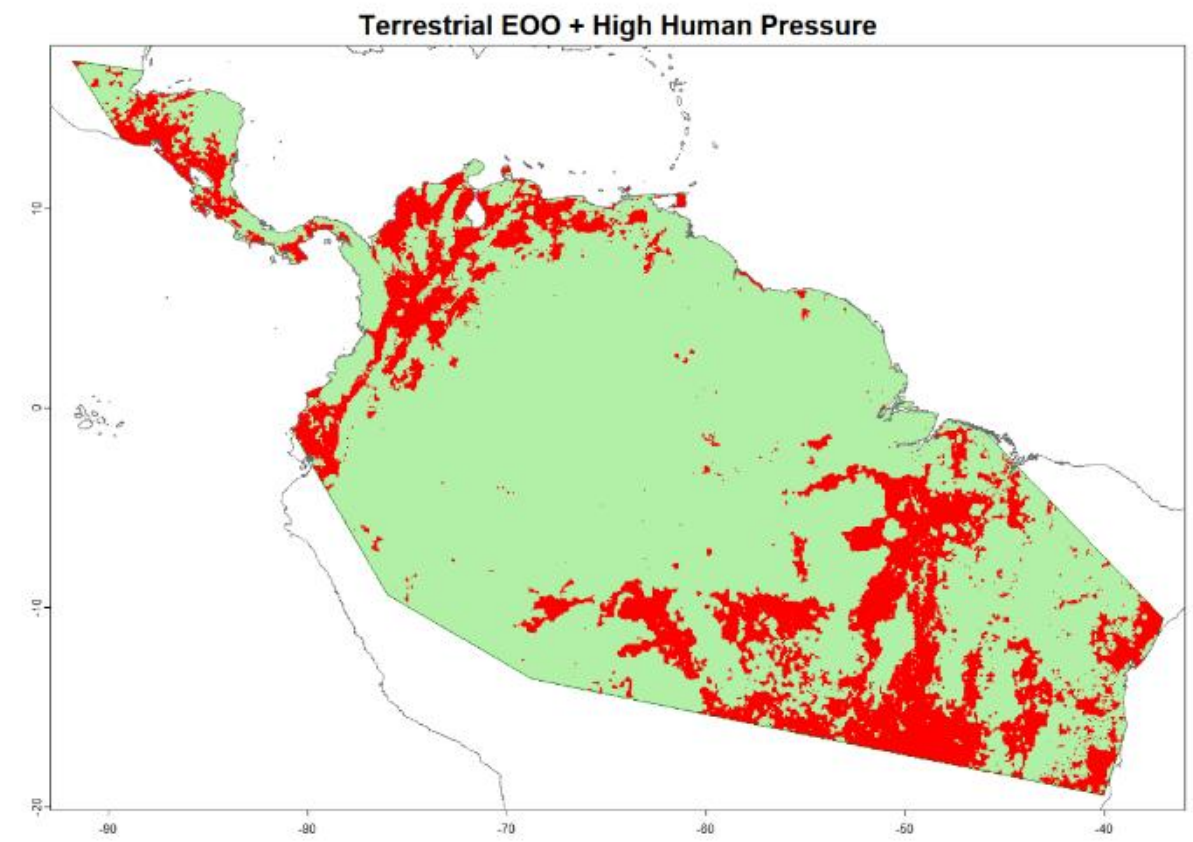

Total terrestrial area: 10466135 km² | High pressure: 2213361 km² (21.15 %)

Figure 122. Terrestrial range and anthropogenic pressure for *Trinycteris nicefori*. Maps illustrate: (left) Terrestrial Extent of Occurrence (EOO) shown in green with black dots representing occurrence records; (right) Areas of high human pressure (red) within the EOO, defined as regions exceeding the 70th percentile of the human footprint index. The caption below presents the total terrestrial area and the proportion experiencing intense human activity. Detailed methodology for calculating terrestrial EOO and human footprint percentage is described in **Supplementary Text 2** and the **main text.**

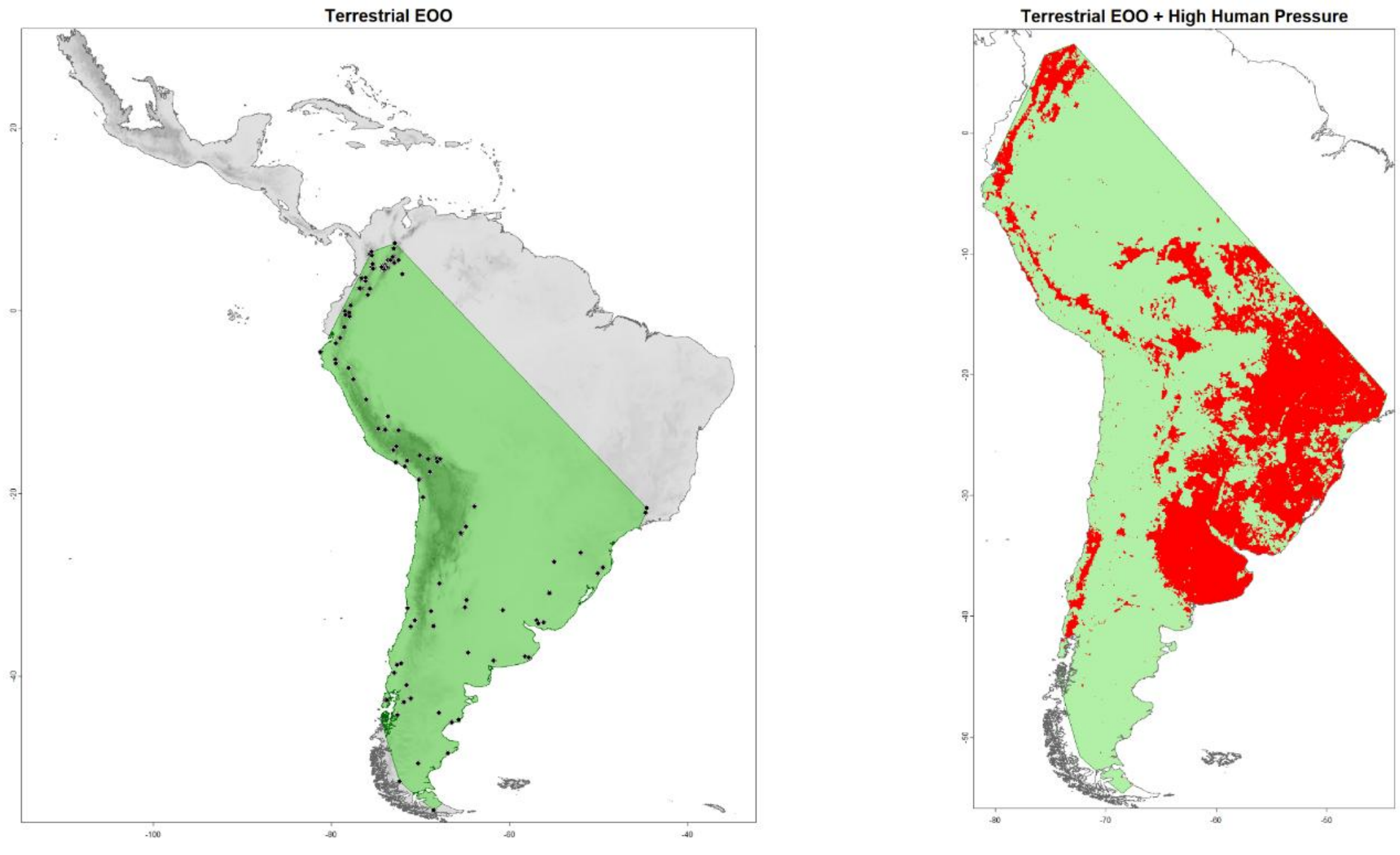

Total terrestrial area: 10758888 km² | High pressure: 3358161 km² (31.21 %)

Figure 123. Terrestrial range and anthropogenic pressure for *Histiotus montanus*. Maps illustrate: (left) Terrestrial Extent of Occurrence (EOO) shown in green with black dots representing occurrence records; (right) Areas of high human pressure (red) within the EOO, defined as regions exceeding the 70th percentile of the human footprint index. The caption below presents the total terrestrial area and the proportion experiencing intense human activity. Detailed methodology for calculating terrestrial EOO and human footprint percentage is described in **Supplementary Text 2** and the **main text.**

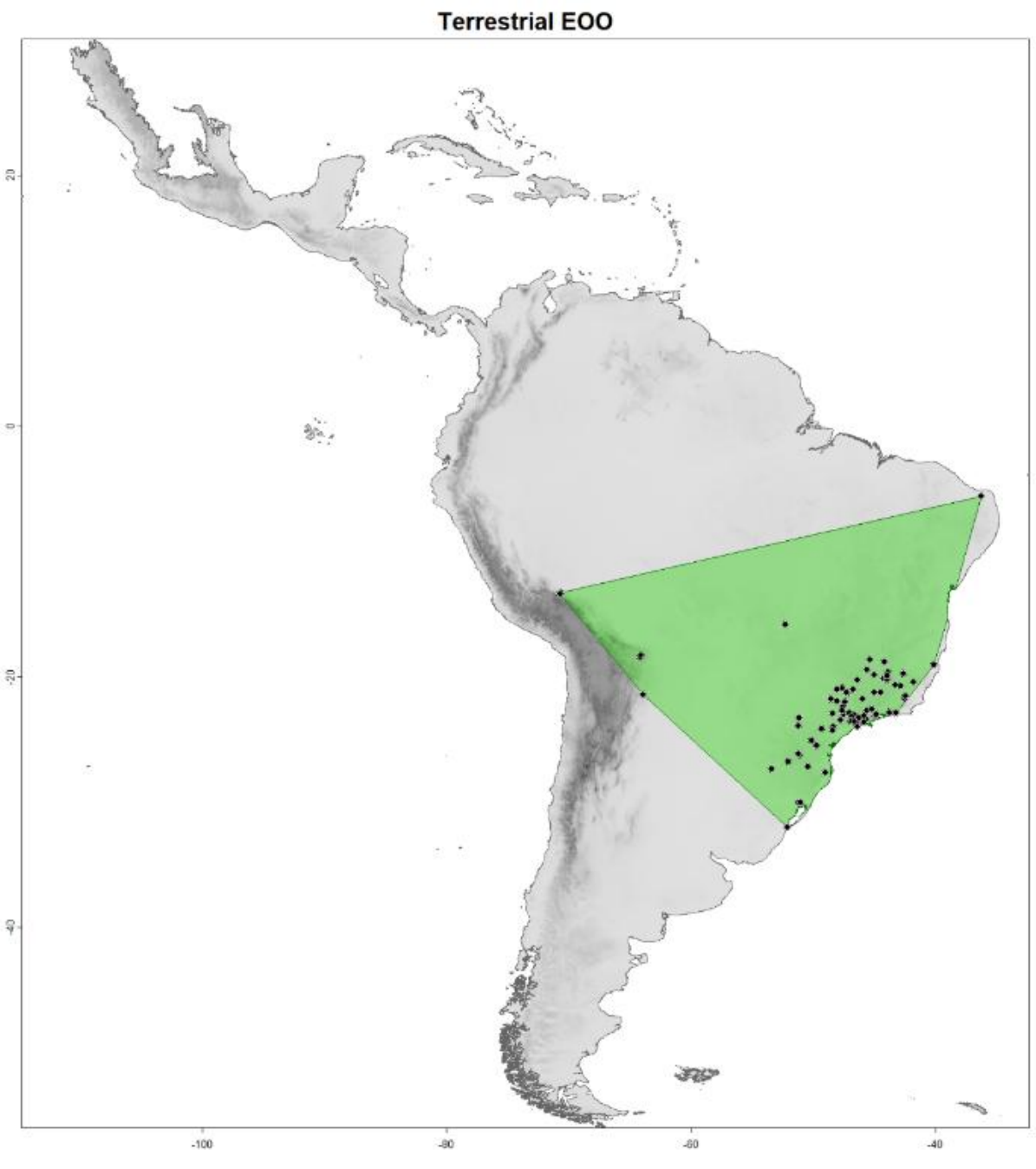

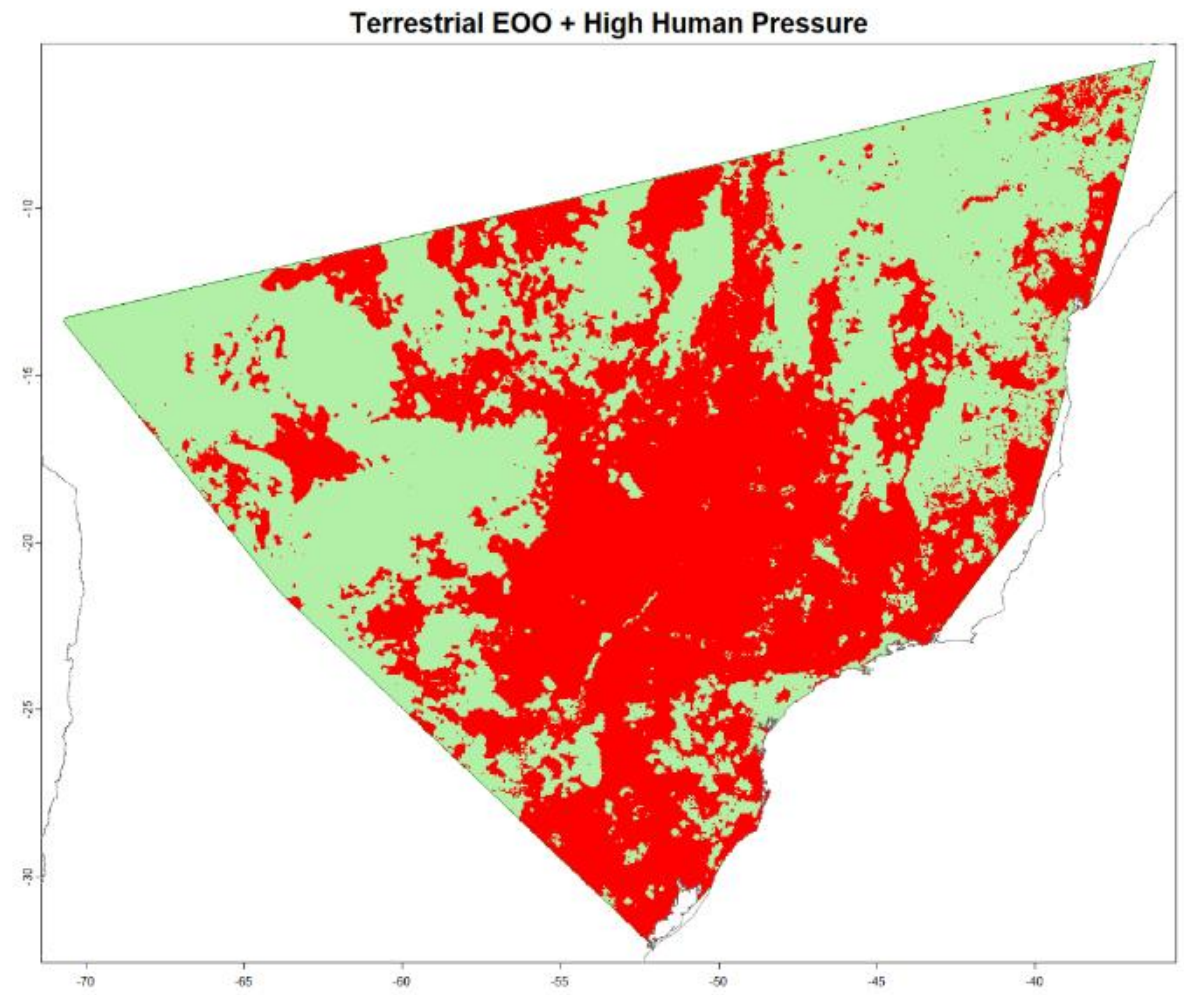

Total terrestrial area: 5236129 km² | High pressure: 2592716 km² (49.52 %)

Figure 124. Terrestrial range and anthropogenic pressure for *Histiotus velatus*. Maps illustrate: (left) Terrestrial Extent of Occurrence (EOO) shown in green with black dots representing occurrence records; (right) Areas of high human pressure (red) within the EOO, defined as regions exceeding the 70th percentile of the human footprint index. The caption below presents the total terrestrial area and the proportion experiencing intense human activity. Detailed methodology for calculating terrestrial EOO and human footprint percentage is described in **Supplementary Text 2** and the **main text.**

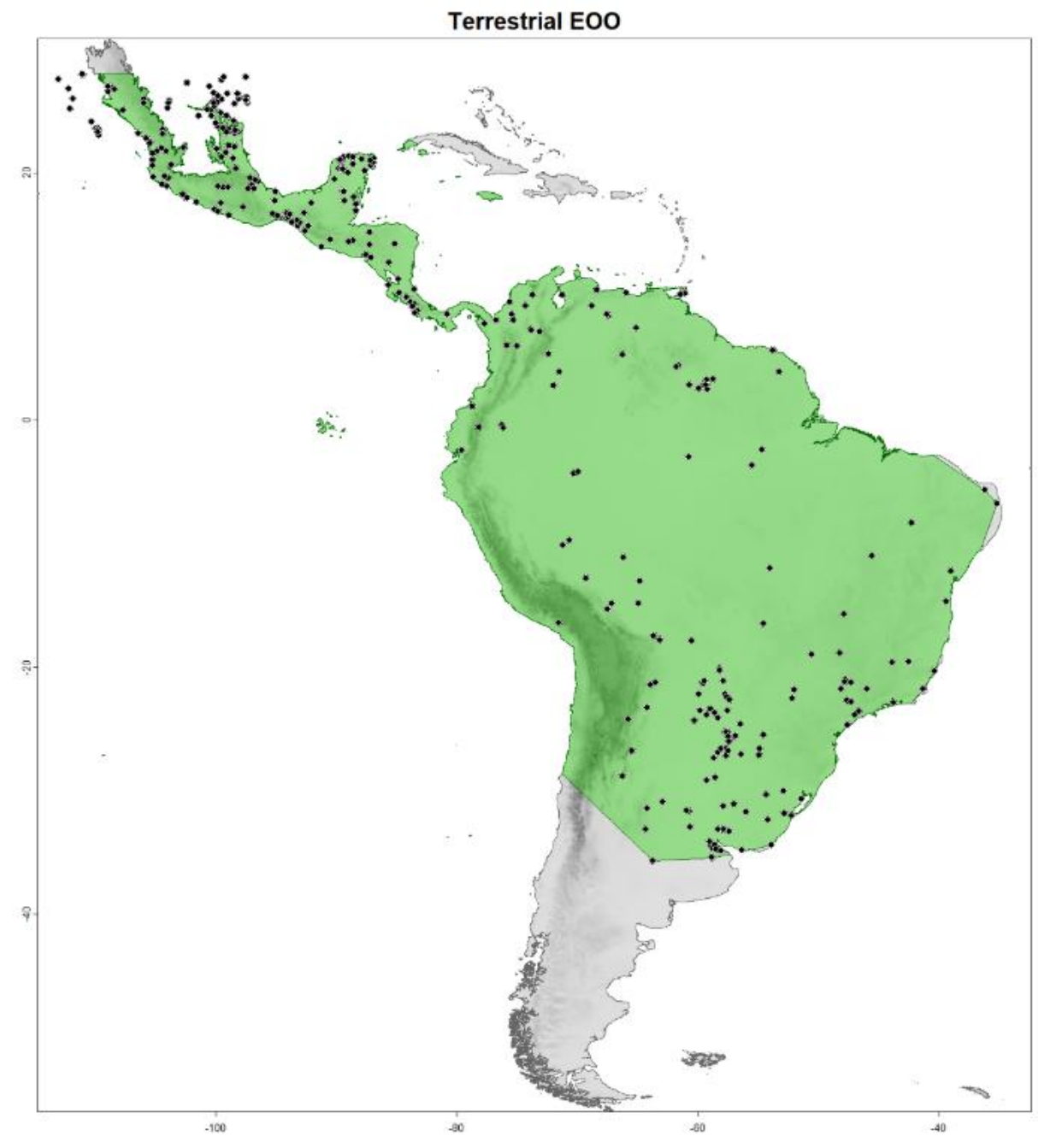

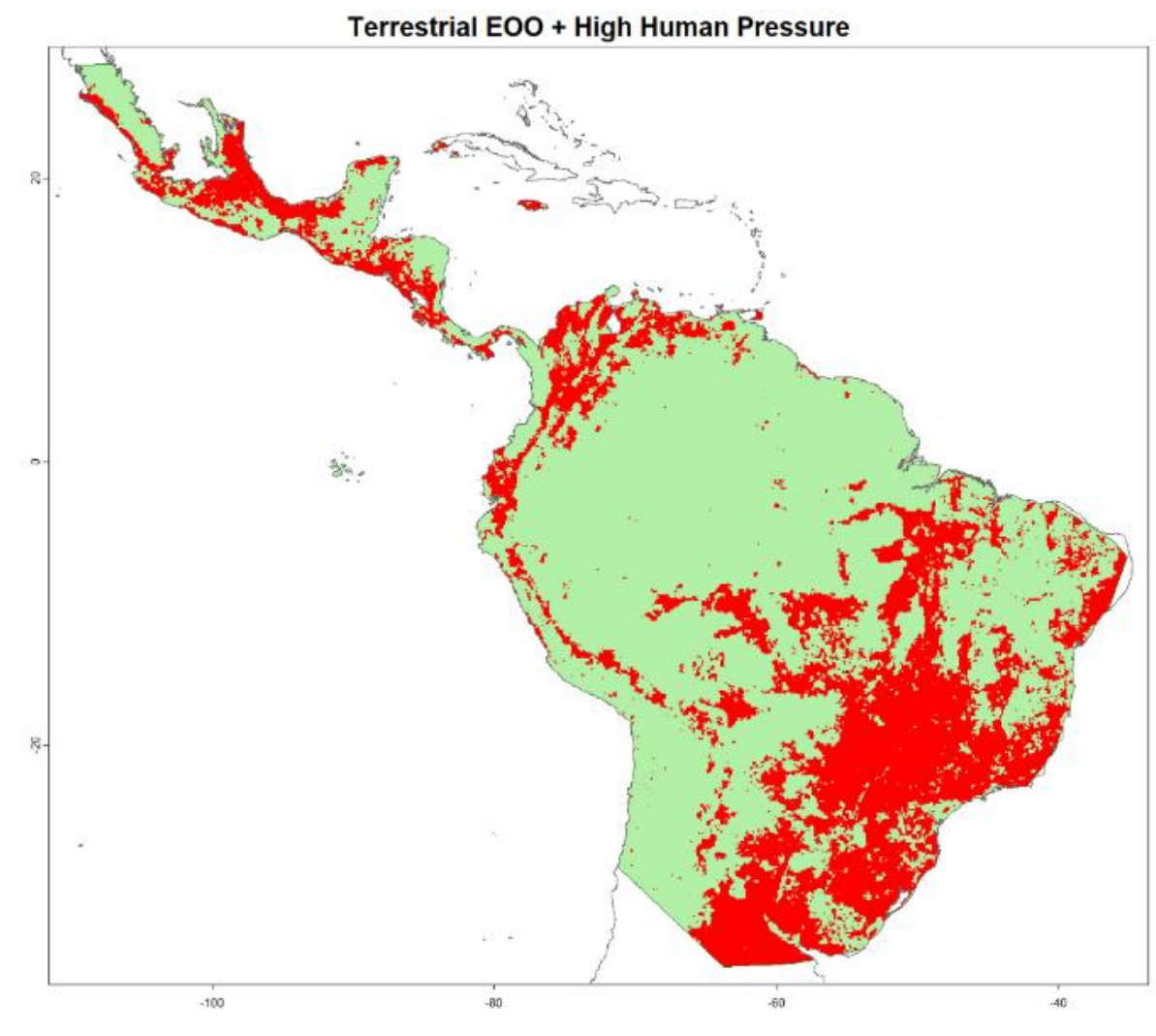

Total terrestrial area: 17276247 km² | High pressure: 5323993 km² (30.82 %)

Figure 125. Terrestrial range and anthropogenic pressure for *Lasiurus ega*. Maps illustrate: (left) Terrestrial Extent of Occurrence (EOO) shown in green with black dots representing occurrence records; (right) Areas of high human pressure (red) within the EOO, defined as regions exceeding the 70th percentile of the human footprint index. The caption below presents the total terrestrial area and the proportion experiencing intense human activity. Detailed methodology for calculating terrestrial EOO and human footprint percentage is described in **Supplementary Text 2** and the **main text.**

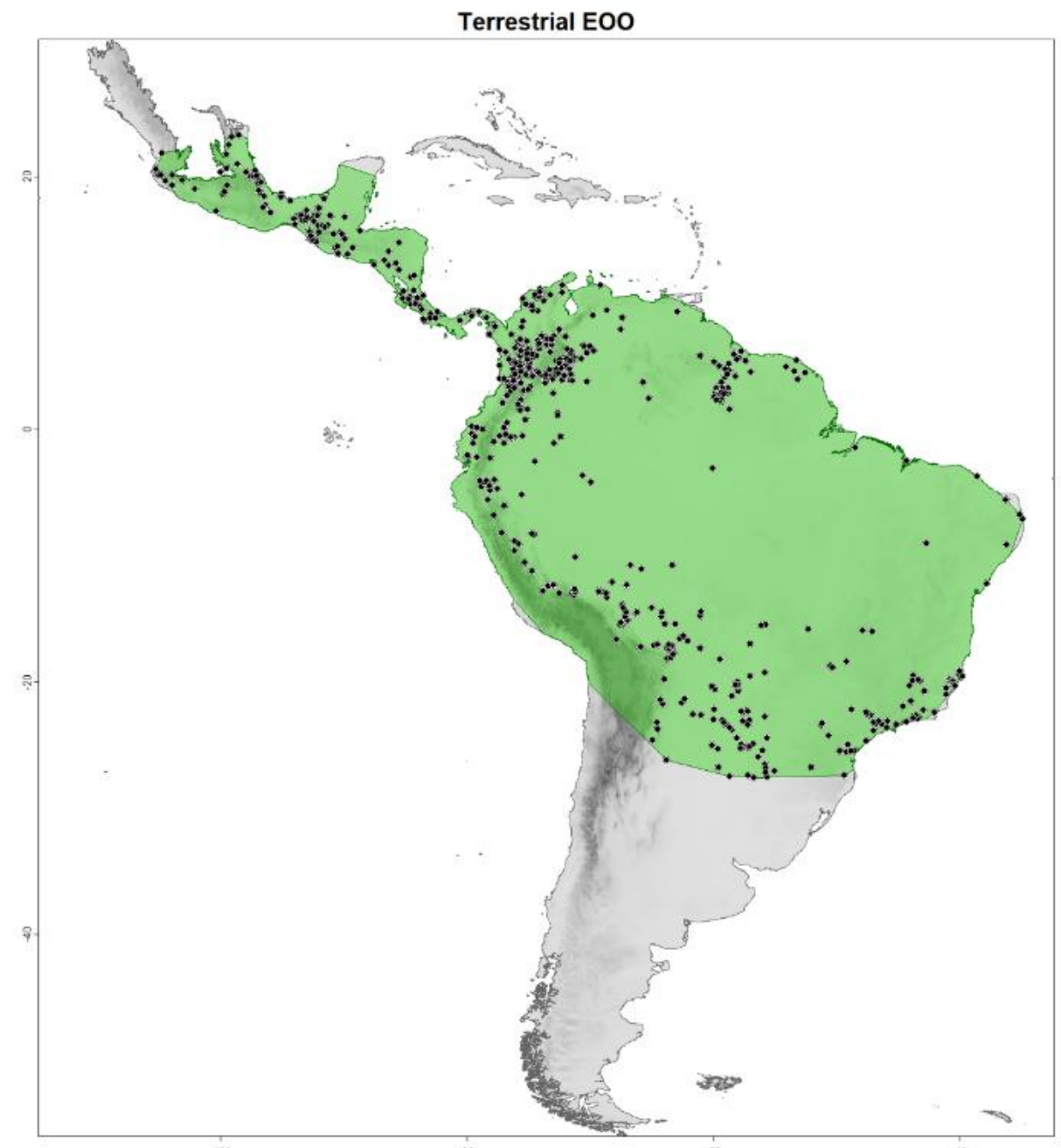

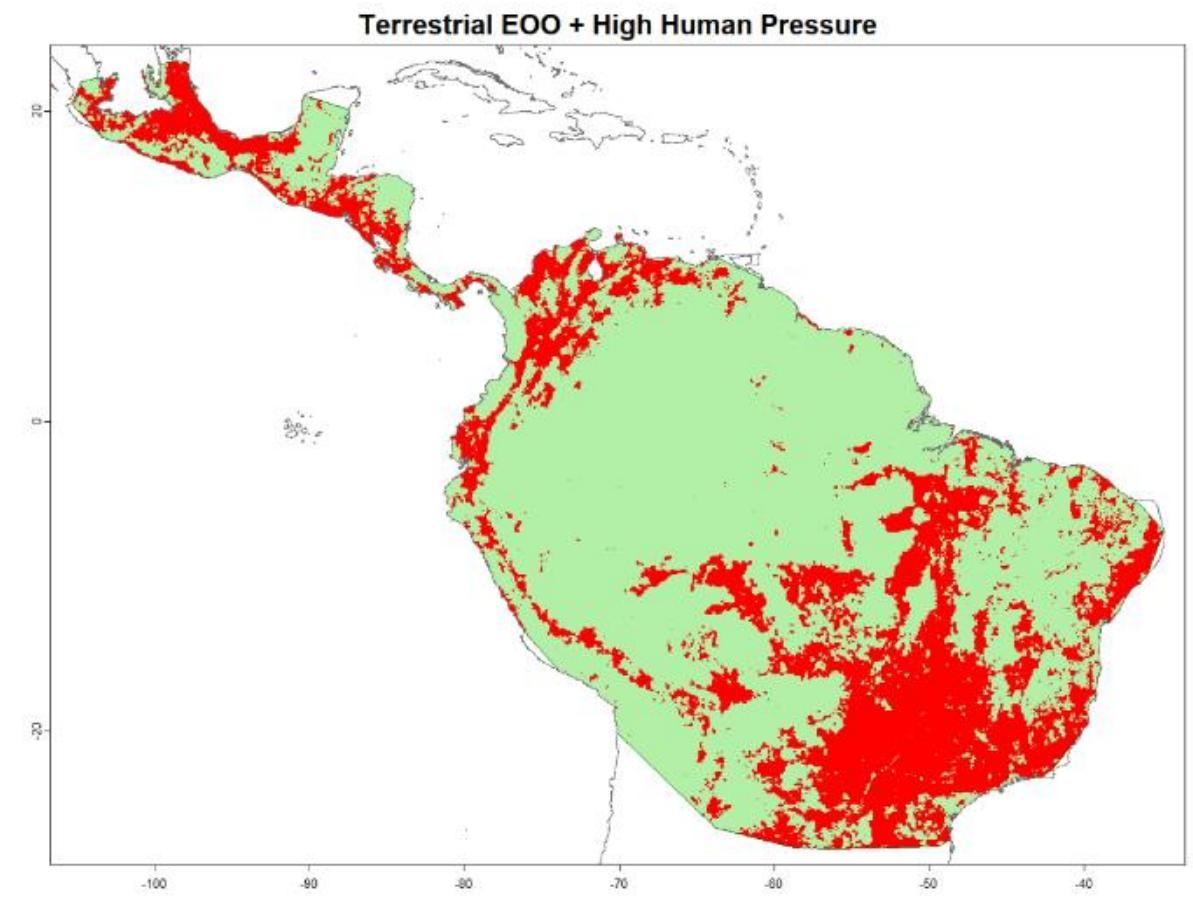

Total terrestrial area: 15215525 km² | High pressure: 4386437 km² (28.83 %)

Figure 126. Terrestrial range and anthropogenic pressure for *Myotis nigricans*. Maps illustrate: (left) Terrestrial Extent of Occurrence (EOO) shown in green with black dots representing occurrence records; (right) Areas of high human pressure (red) within the EOO, defined as regions exceeding the 70th percentile of the human footprint index. The caption below presents the total terrestrial area and the proportion experiencing intense human activity. Detailed methodology for calculating terrestrial EOO and human footprint percentage is described in **Supplementary Text 2** and the **main text.**

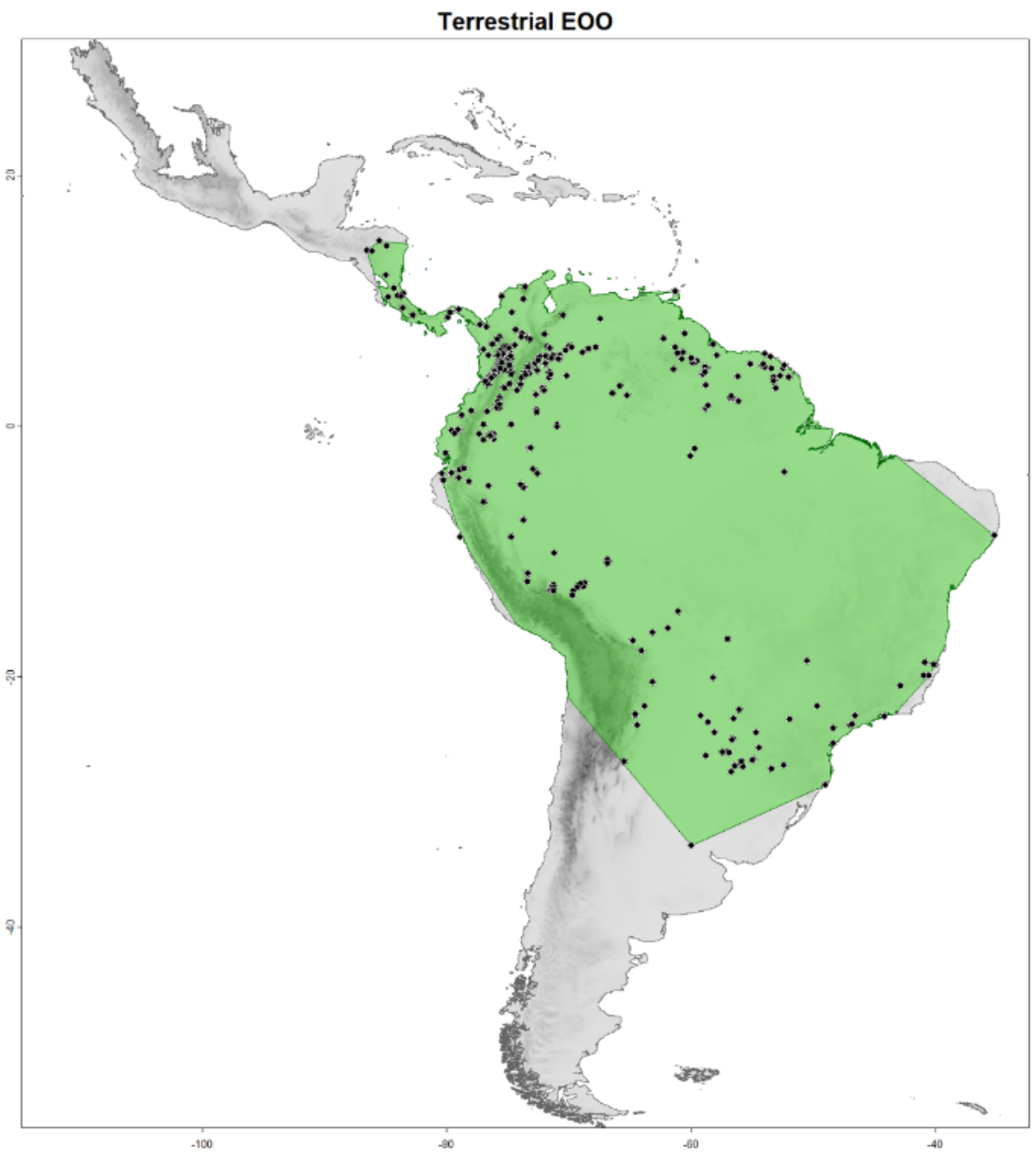

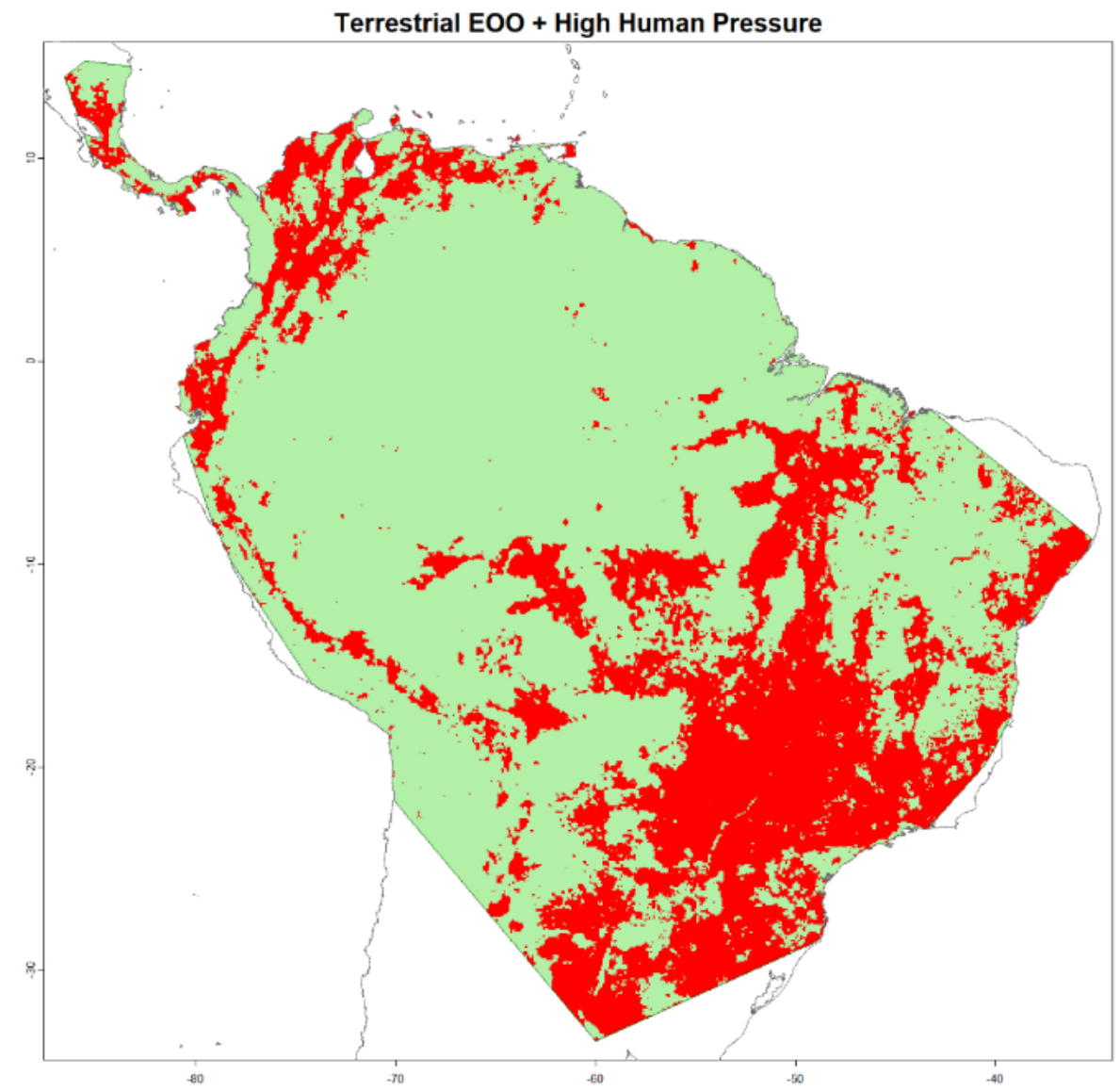

Total terrestrial area: 14824227 km² | High pressure: 4276606 km² (28.85 %)

Figure 127. Terrestrial range and anthropogenic pressure for *Myotis riparius*. Maps illustrate: (left) Terrestrial Extent of Occurrence (EOO) shown in green with black dots representing occurrence records; (right) Areas of high human pressure (red) within the EOO, defined as regions exceeding the 70th percentile of the human footprint index. The caption below presents the total terrestrial area and the proportion experiencing intense human activity. Detailed methodology for calculating terrestrial EOO and human footprint percentage is described in **Supplementary Text 2** and the **main text.**

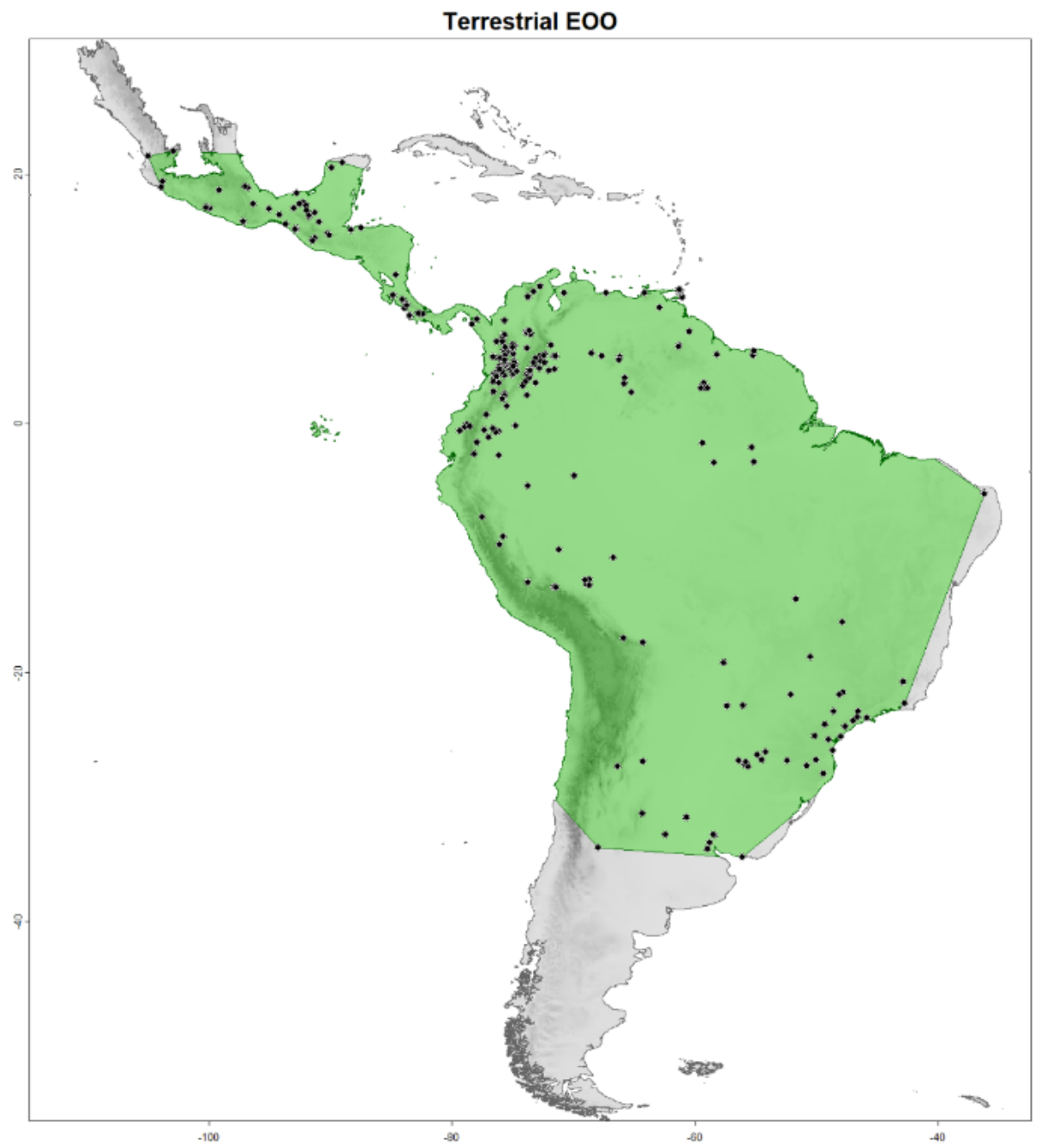

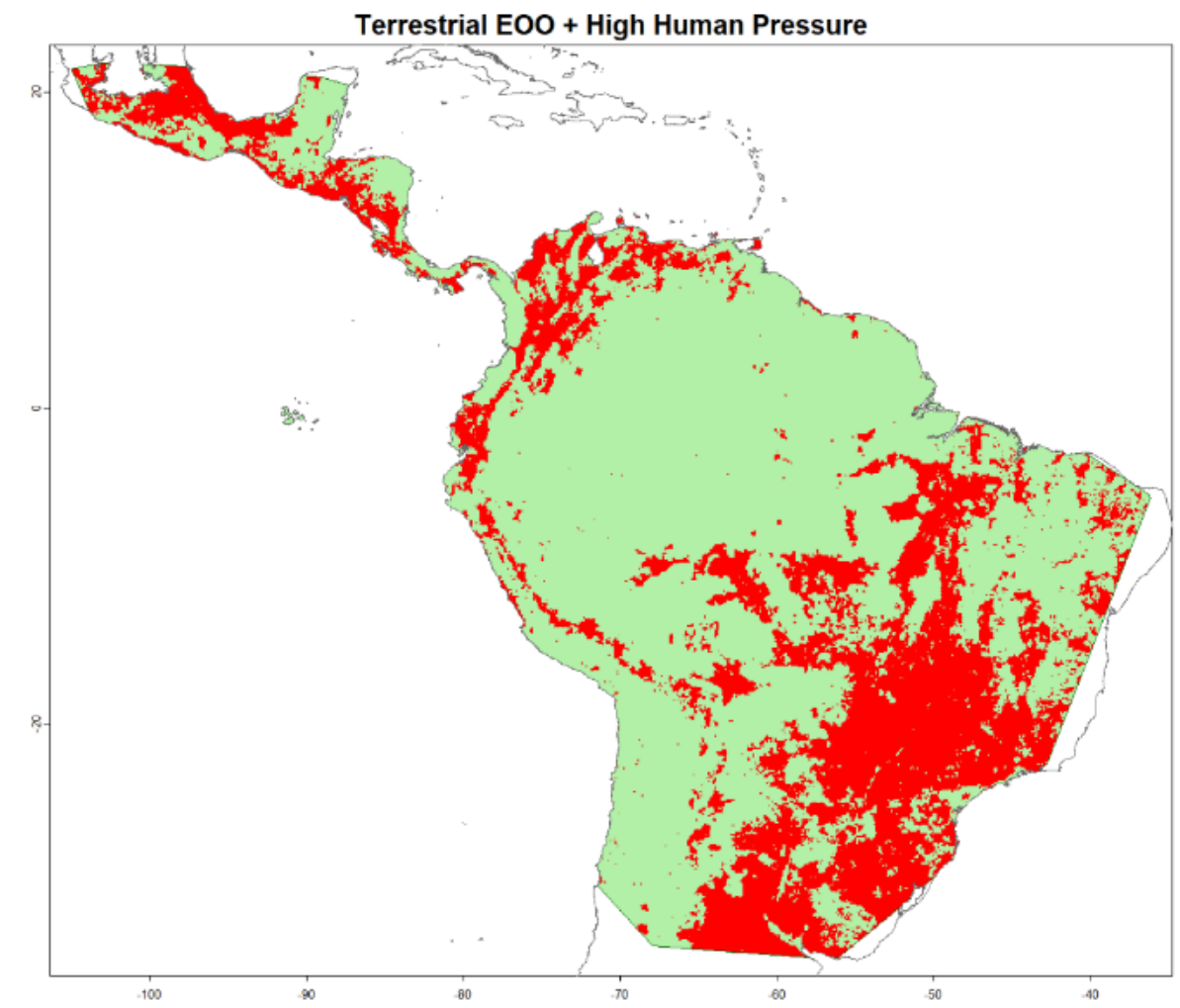

Total terrestrial area: 16701492 km² | High pressure: 4965570 km² (29.73 %)

Figure 128. Terrestrial range and anthropogenic pressure for *Neoeptesicus brasiliensis*. Maps illustrate: (left) Terrestrial Extent of Occurrence (EOO) shown in green with black dots representing occurrence records; (right) Areas of high human pressure (red) within the EOO, defined as regions exceeding the 70th percentile of the human footprint index. The caption below presents the total terrestrial area and the proportion experiencing intense human activity. Detailed methodology for calculating terrestrial EOO and human footprint percentage is described in **Supplementary Text 2** and the **main text.**

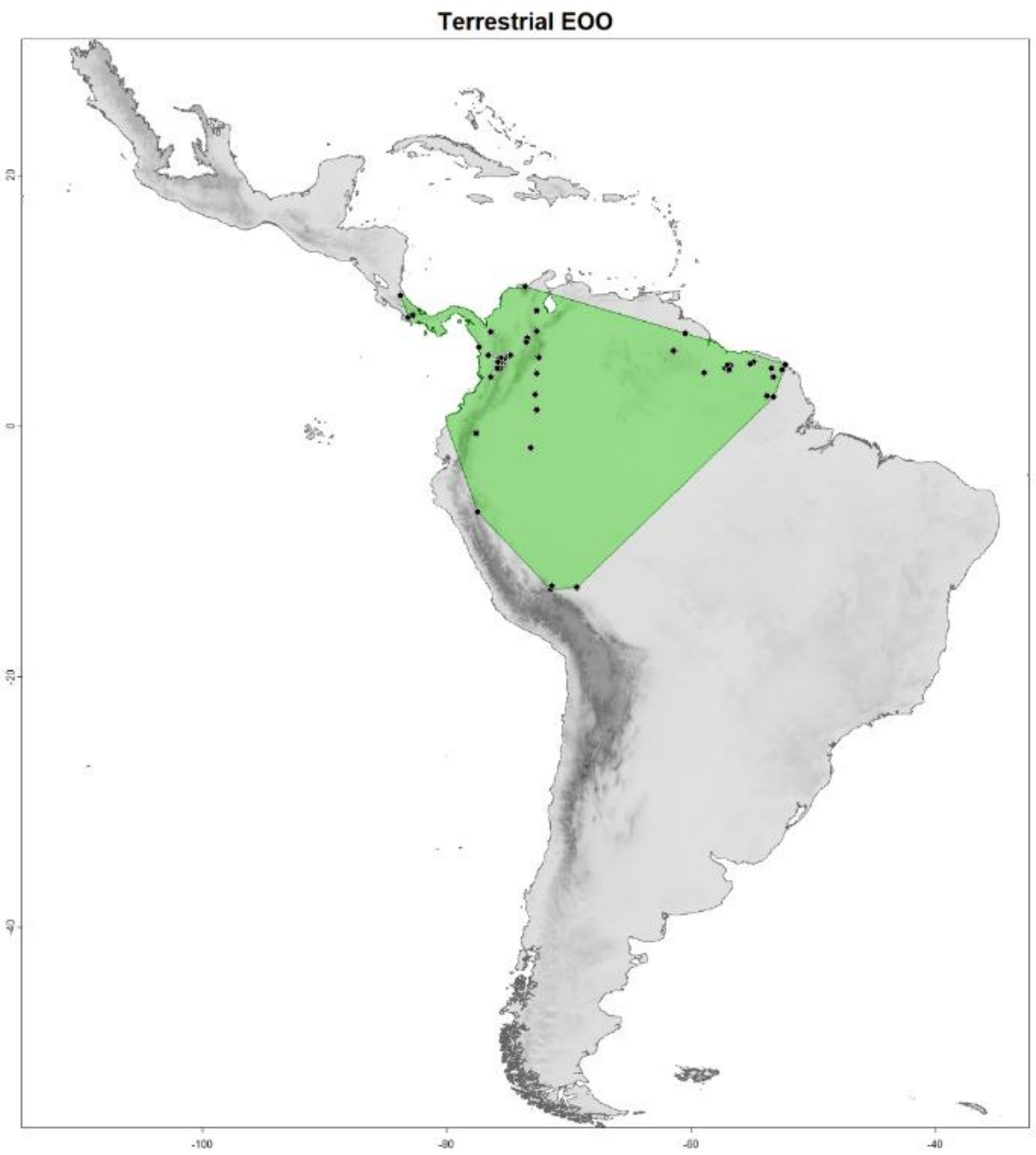

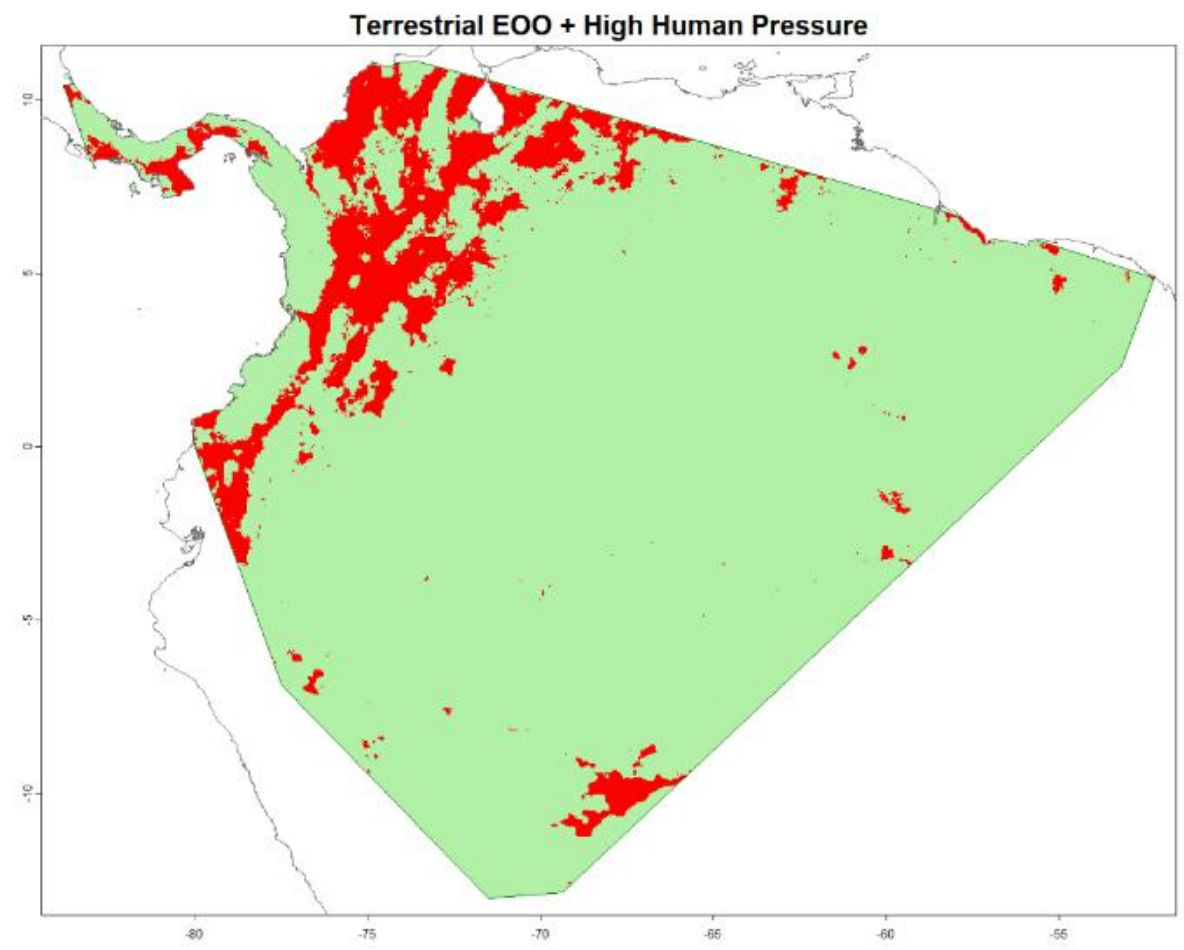

Total terrestrial area: 4905640 km² | High pressure: 503092 km² (10.26 %)

Figure 129. Terrestrial range and anthropogenic pressure for *Neoeptesicus chiriquinus*. Maps illustrate: (left) Terrestrial Extent of Occurrence (EOO) shown in green with black dots representing occurrence records; (right) Areas of high human pressure (red) within the EOO, defined as regions exceeding the 70th percentile of the human footprint index. The caption below presents the total terrestrial area and the proportion experiencing intense human activity. Detailed methodology for calculating terrestrial EOO and human footprint percentage is described in **Supplementary Text 2** and the **main text.**

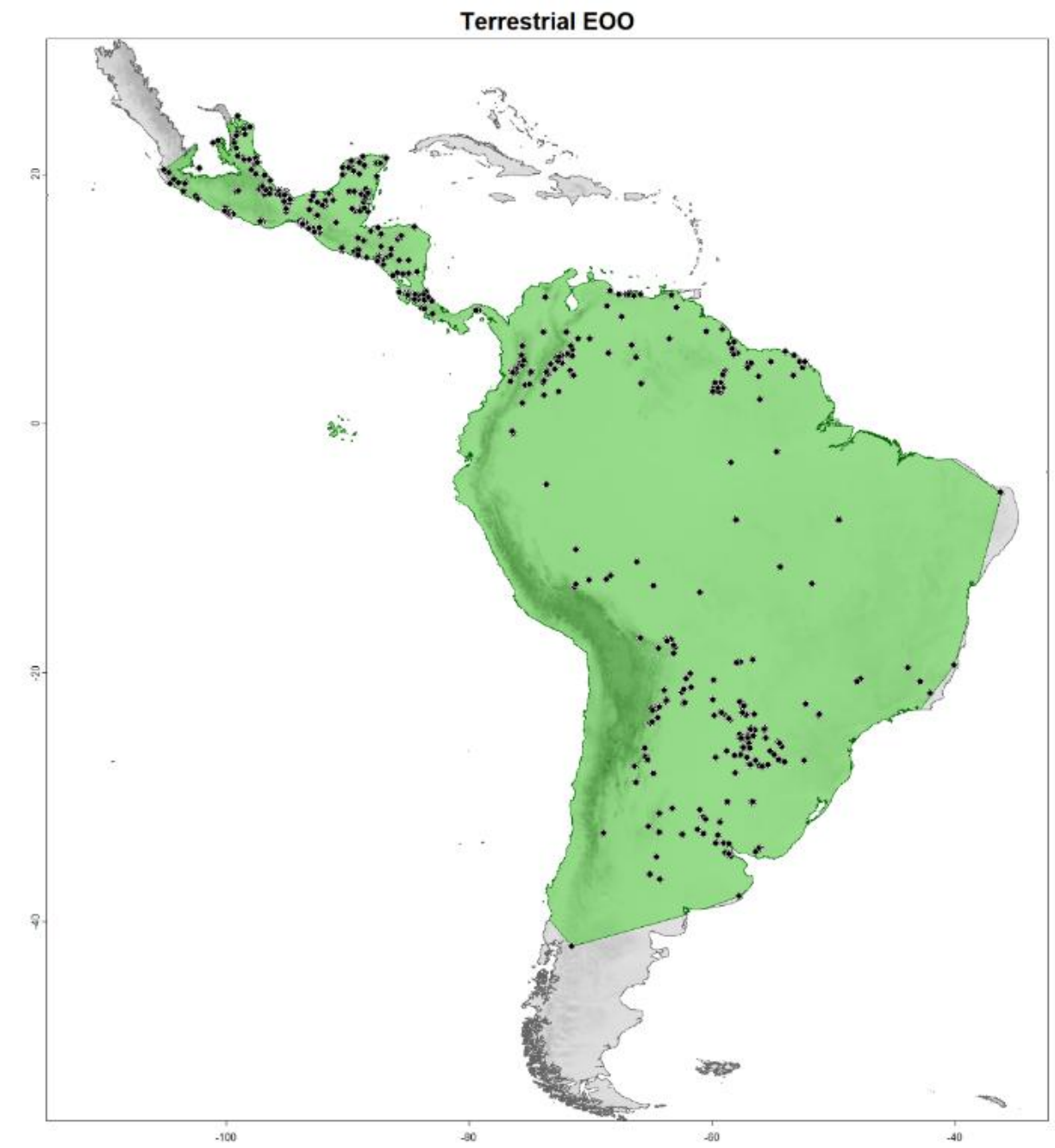

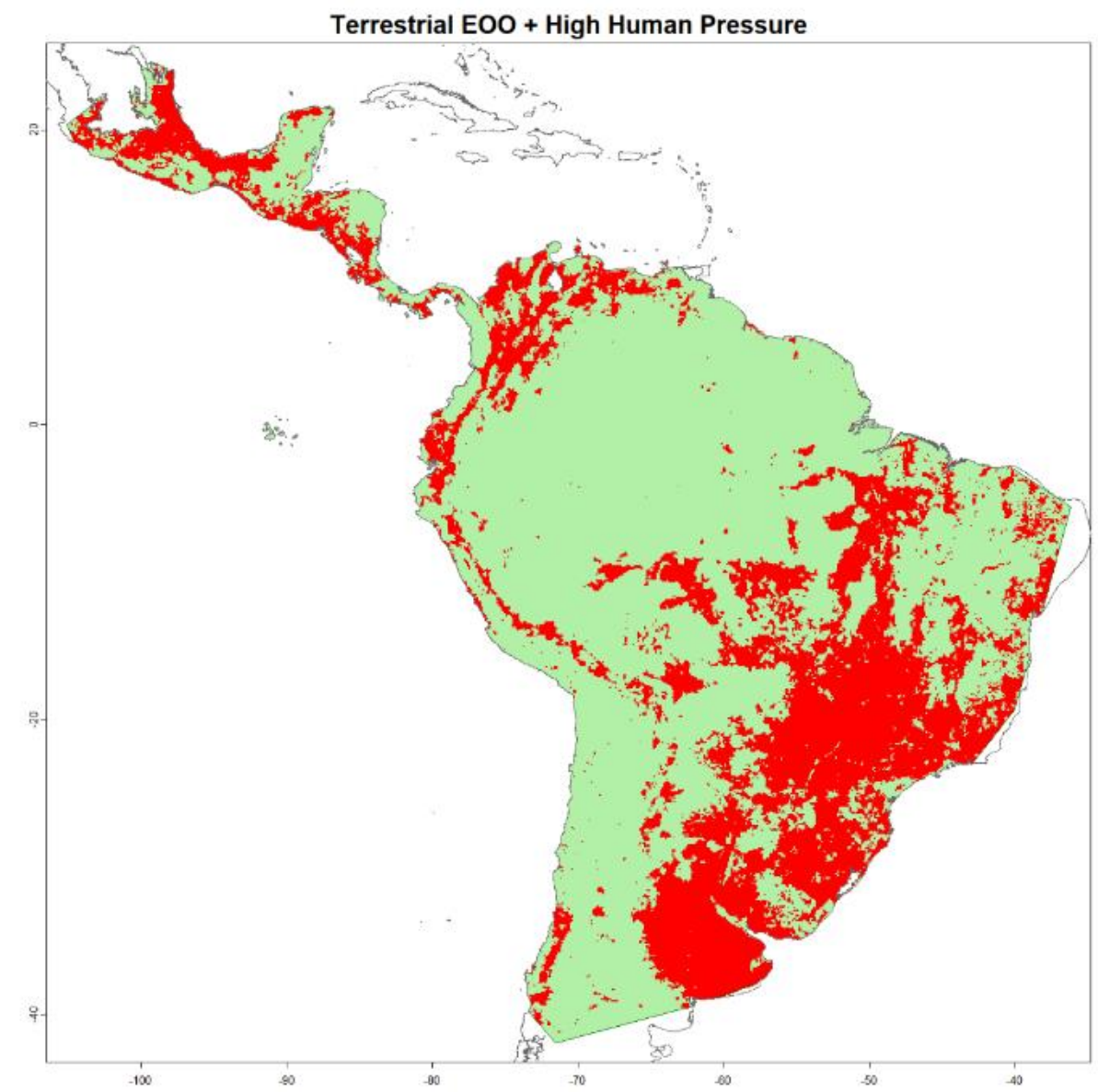

Total terrestrial area: 17925685 km² | High pressure: 5483381 km² (30.59 %)

Figure 130. Terrestrial range and anthropogenic pressure for *Neoeptesicus furinalis*. Maps illustrate: (left) Terrestrial Extent of Occurrence (EOO) shown in green with black dots representing occurrence records; (right) Areas of high human pressure (red) within the EOO, defined as regions exceeding the 70th percentile of the human footprint index. The caption below presents the total terrestrial area and the proportion experiencing intense human activity. Detailed methodology for calculating terrestrial EOO and human footprint percentage is described in **Supplementary Text 2** and the **main text.**

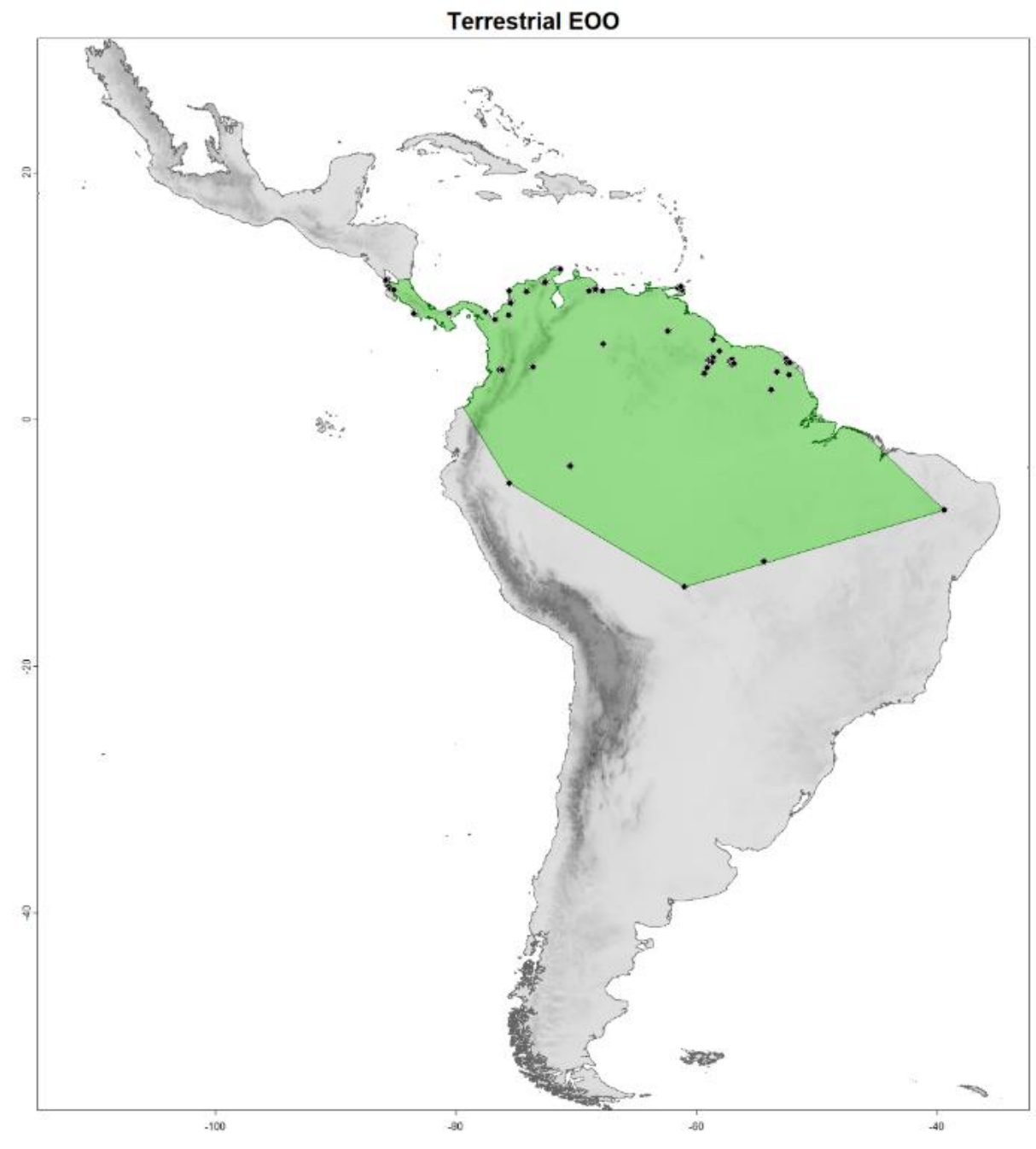

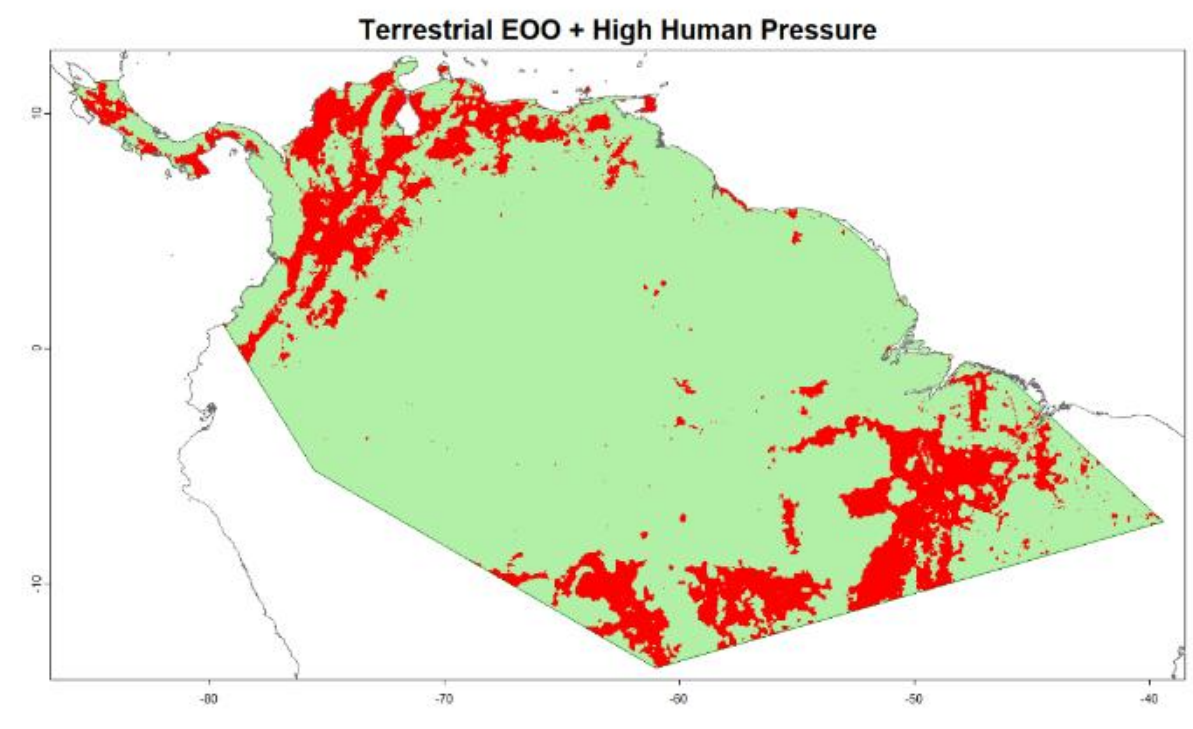

Total terrestrial area: 7296081 km² | High pressure: 1232650 km² (16.89 %)

Figure 131. Terrestrial range and anthropogenic pressure for *Pteronotus personatus*. Maps illustrate: (left) Terrestrial Extent of Occurrence (EOO) shown in green with black dots representing occurrence records; (right) Areas of high human pressure (red) within the EOO, defined as regions exceeding the 70th percentile of the human footprint index. The caption below presents the total terrestrial area and the proportion experiencing intense human activity. Detailed methodology for calculating terrestrial EOO and human footprint percentage is described in **Supplementary Text 2** and the **main text.**

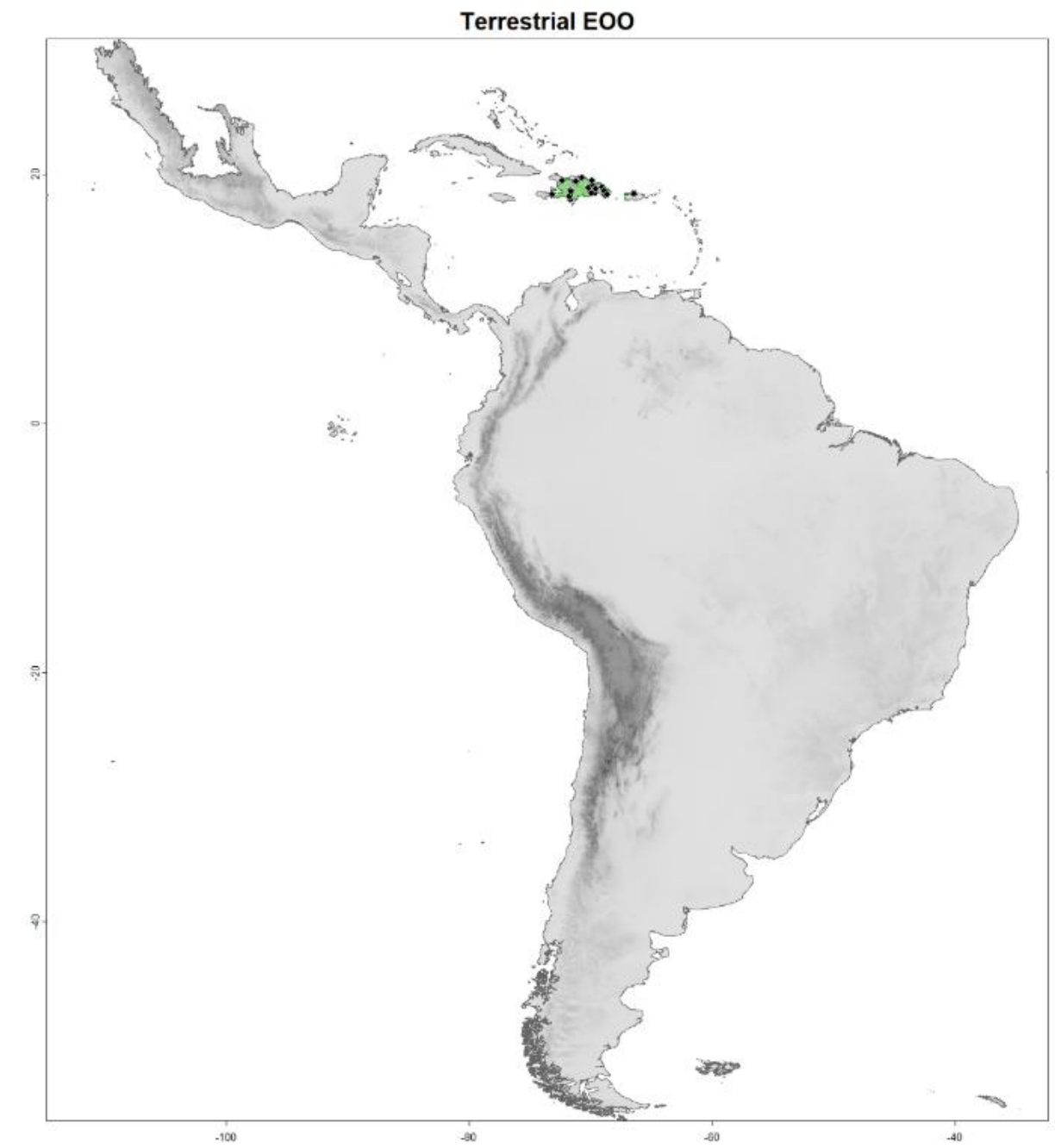

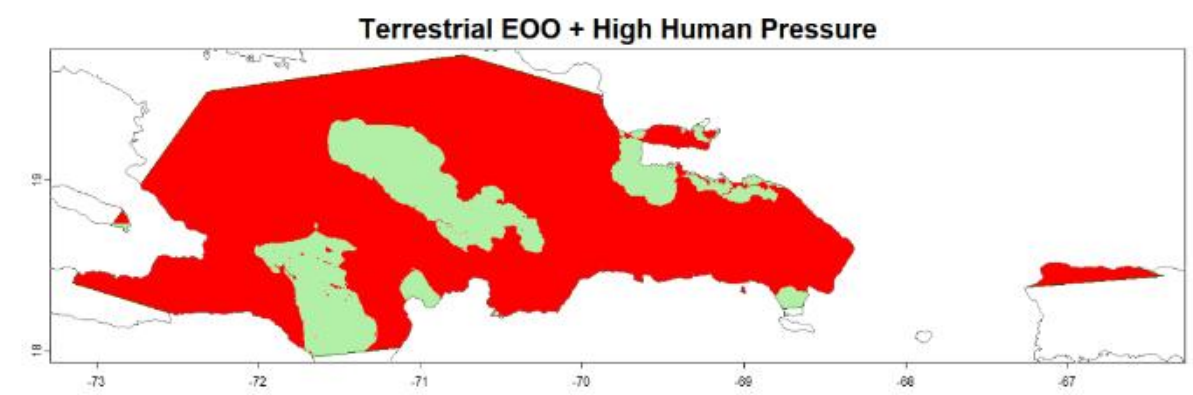

Total terrestrial area: 57103 km² | High pressure: 46639 km² (81.68 %)

Figure 132. Terrestrial range and anthropogenic pressure for *Pteronotus pusillus.* Maps illustrate: (left) Terrestrial Extent of Occurrence (EOO) shown in green with black dots representing occurrence records; (right) Areas of high human pressure (red) within the EOO, defined as regions exceeding the 70th percentile of the human footprint index. The caption below presents the total terrestrial area and the proportion experiencing intense human activity. Detailed methodology for calculating terrestrial EOO and human footprint percentage is described in **Supplementary Text 2** and the **main text.**

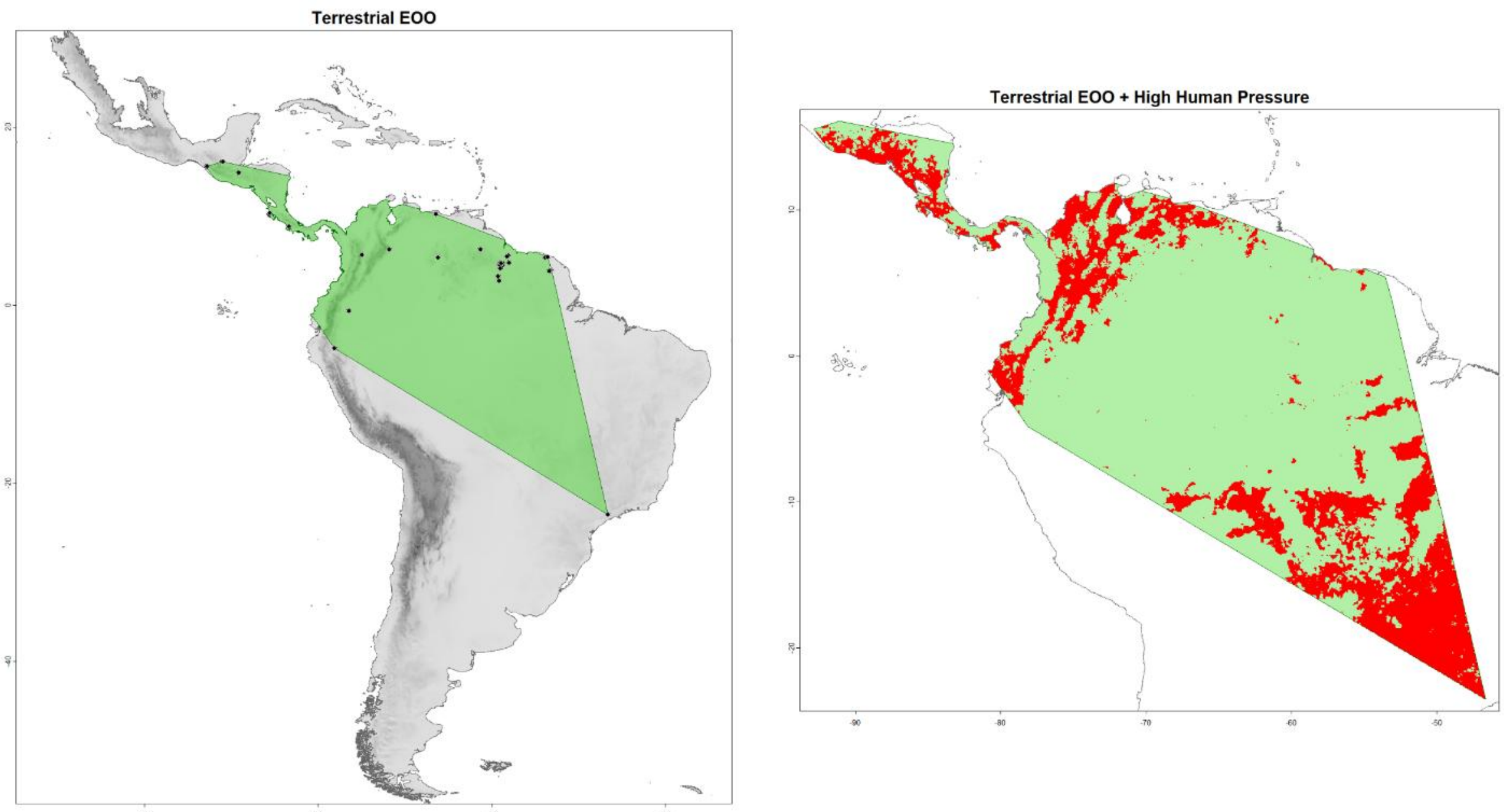

Total terrestrial area: 8093269 km² | High pressure: 1848548 km² (22.84 %)

Figure 133. Terrestrial range and anthropogenic pressure for *Eumops hansae.* Maps illustrate: (left) Terrestrial Extent of Occurrence (EOO) shown in green with black dots representing occurrence records; (right) Areas of high human pressure (red) within the EOO, defined as regions exceeding the 70th percentile of the human footprint index. The caption below presents the total terrestrial area and the proportion experiencing intense human activity. Detailed methodology for calculating terrestrial EOO and human footprint percentage is described in **Supplementary Text 2 and** the **main text.**

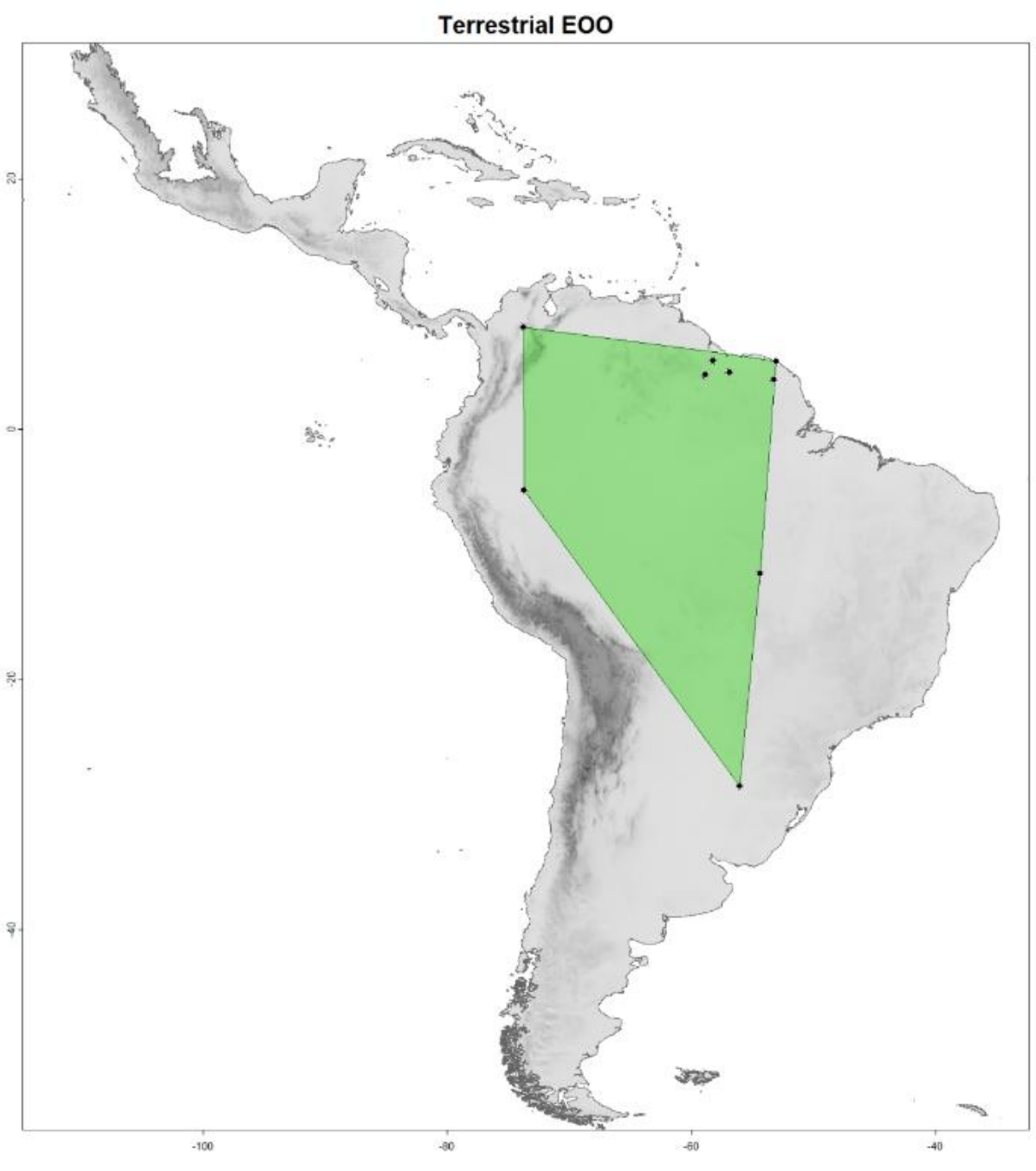

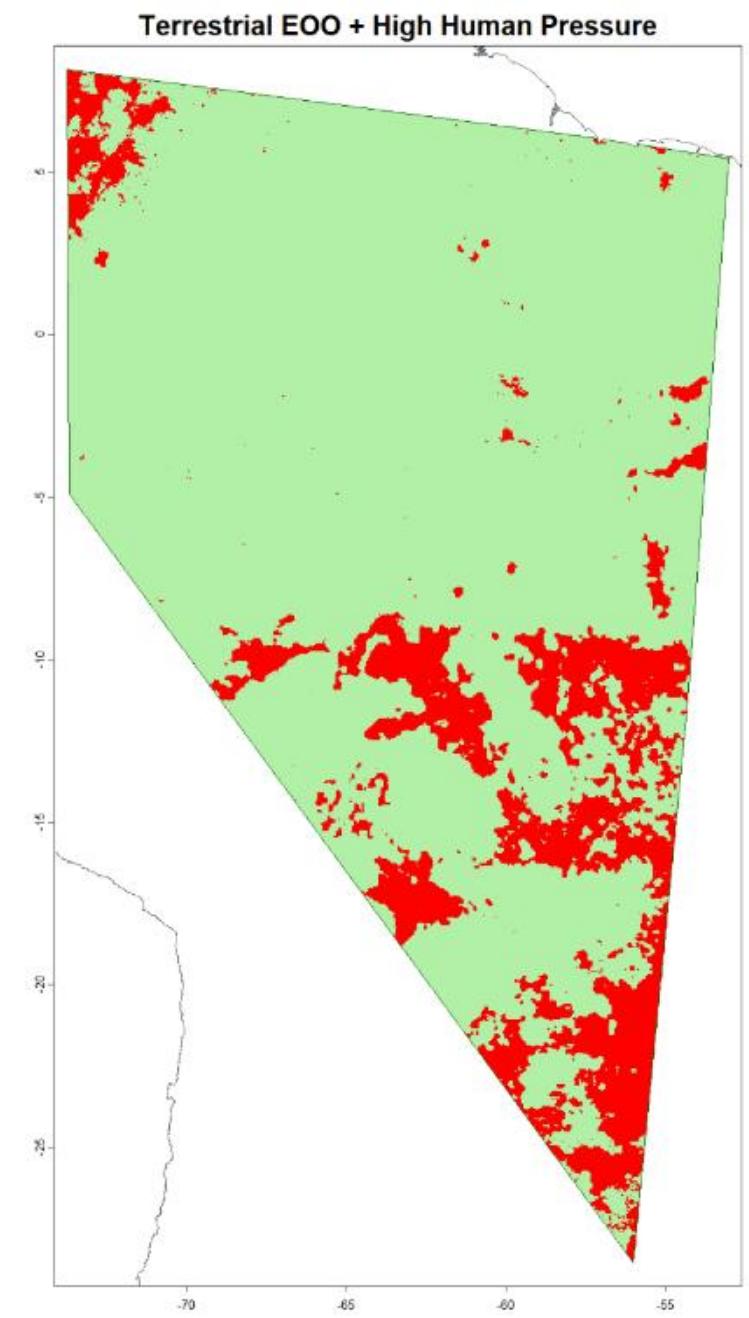

Total terrestrial area: 5887517 km² | High pressure: 819391 km² (13.92 %)

Figure 134. Terrestrial range and anthropogenic pressure for *Molossops neglectus.* Maps illustrate: (left) Terrestrial Extent of Occurrence (EOO) shown in green with black dots representing occurrence records; (right) Areas of high human pressure (red) within the EOO, defined as regions exceeding the 70th percentile of the human footprint index. The caption below presents the total terrestrial area and the proportion experiencing intense human activity. Detailed methodology for calculating terrestrial EOO and human footprint percentage is described in **Supplementary Text 2** and the **main text.**

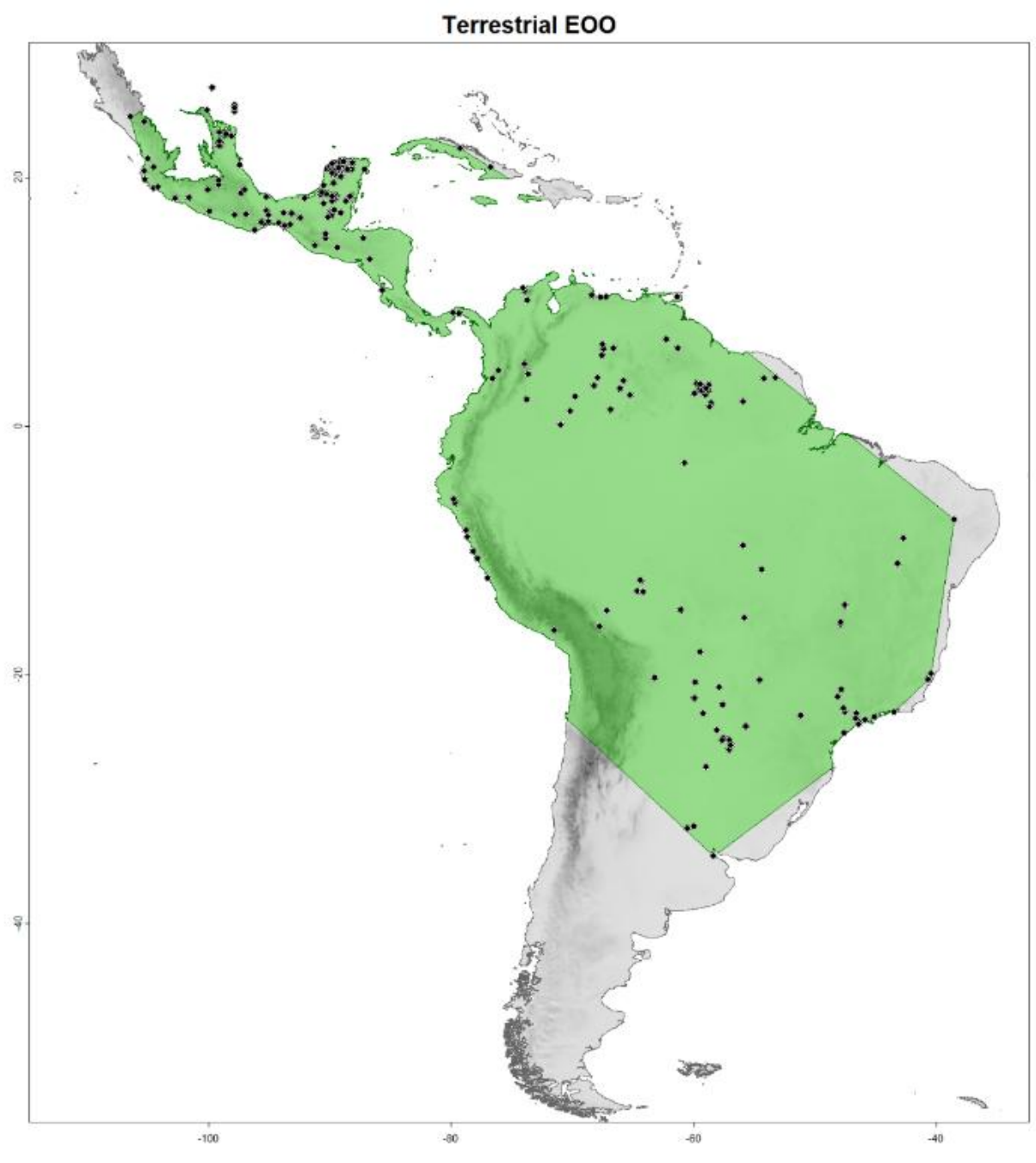

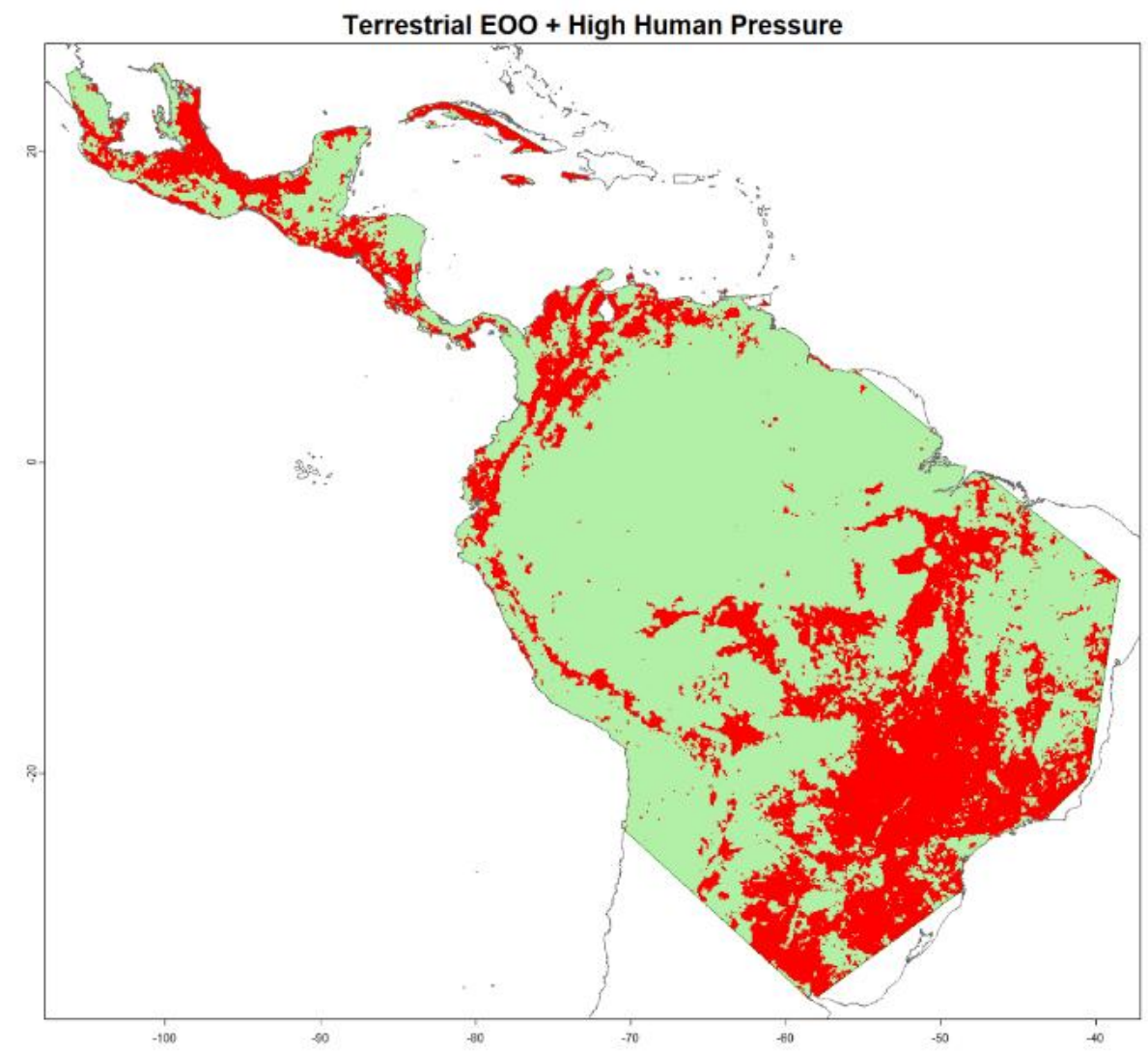

Total terrestrial area: 15876917 km² | High pressure: 4771924 km² (30.06 %)

Figure 135. Terrestrial range and anthropogenic pressure for *Nyctinomops laticaudatus*. Maps illustrate: (left) Terrestrial Extent of Occurrence (EOO) shown in green with black dots representing occurrence records; (right) Areas of high human pressure (red) within the EOO, defined as regions exceeding the 70th percentile of the human footprint index. The caption below presents the total terrestrial area and the proportion experiencing intense human activity. Detailed methodology for calculating terrestrial EOO and human footprint percentage is described in **Supplementary Text 2** and the **main text.**

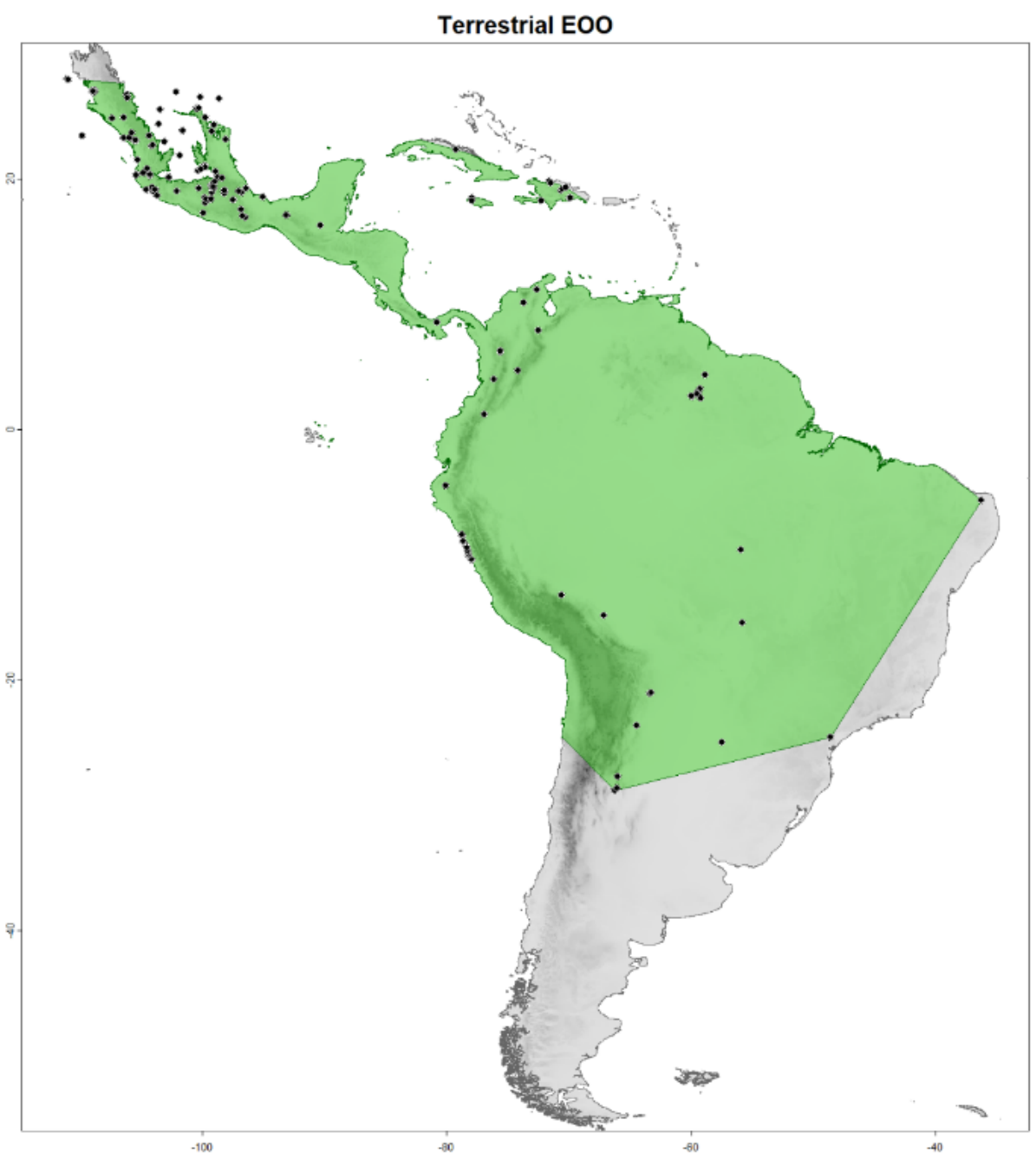

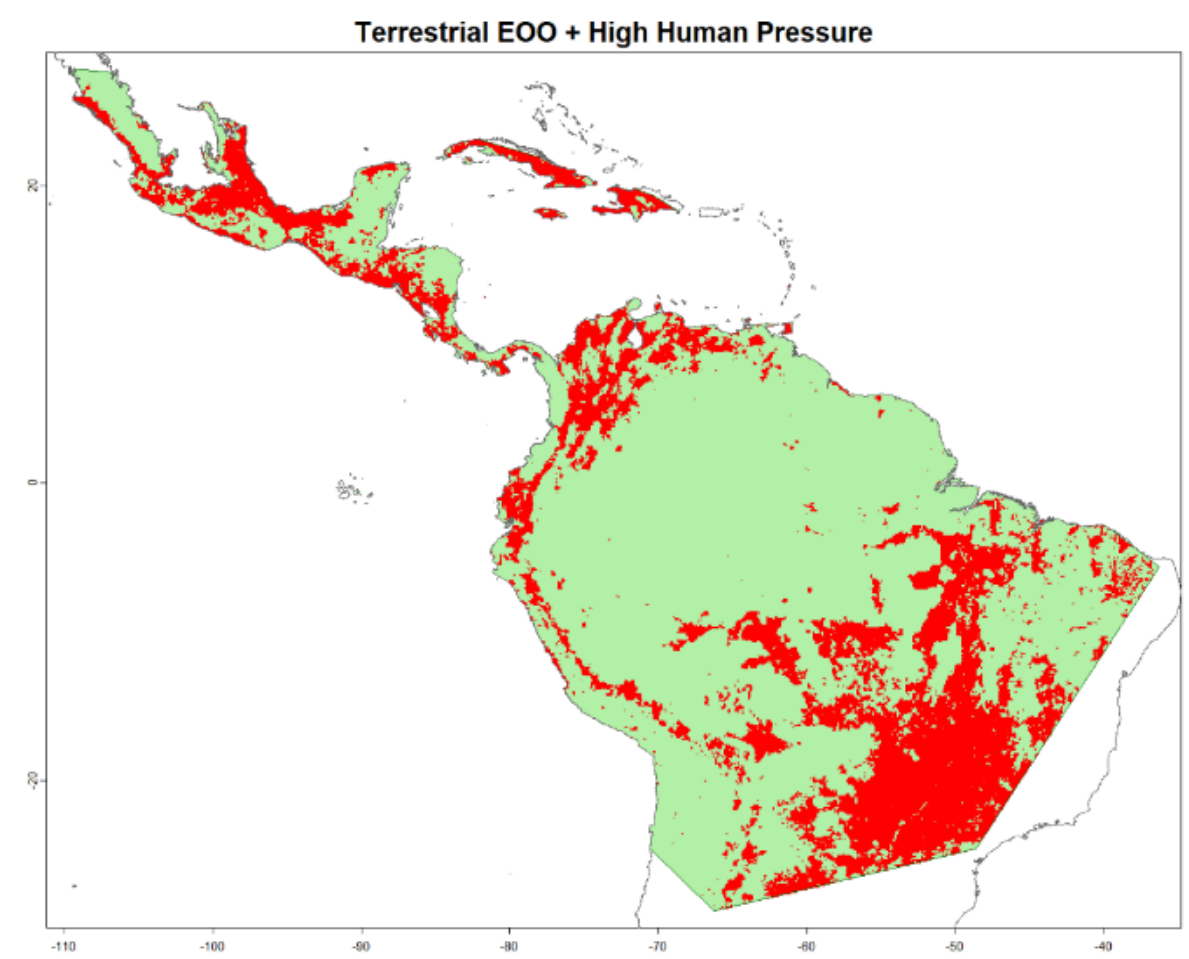

Total terrestrial area: 15159902 km² | High pressure: 4080905 km² (26.92 %)

Figure 136. Terrestrial range and anthropogenic pressure for *Nyctinomops macrotis.* Maps illustrate: (left) Terrestrial Extent of Occurrence (EOO) shown in green with black dots representing occurrence records; (right) Areas of high human pressure (red) within the EOO, defined as regions exceeding the 70th percentile of the human footprint index. The caption below presents the total terrestrial area and the proportion experiencing intense human activity. Detailed methodology for calculating terrestrial EOO and human footprint percentage is described in **Supplementary Text 2** and the **main text.**

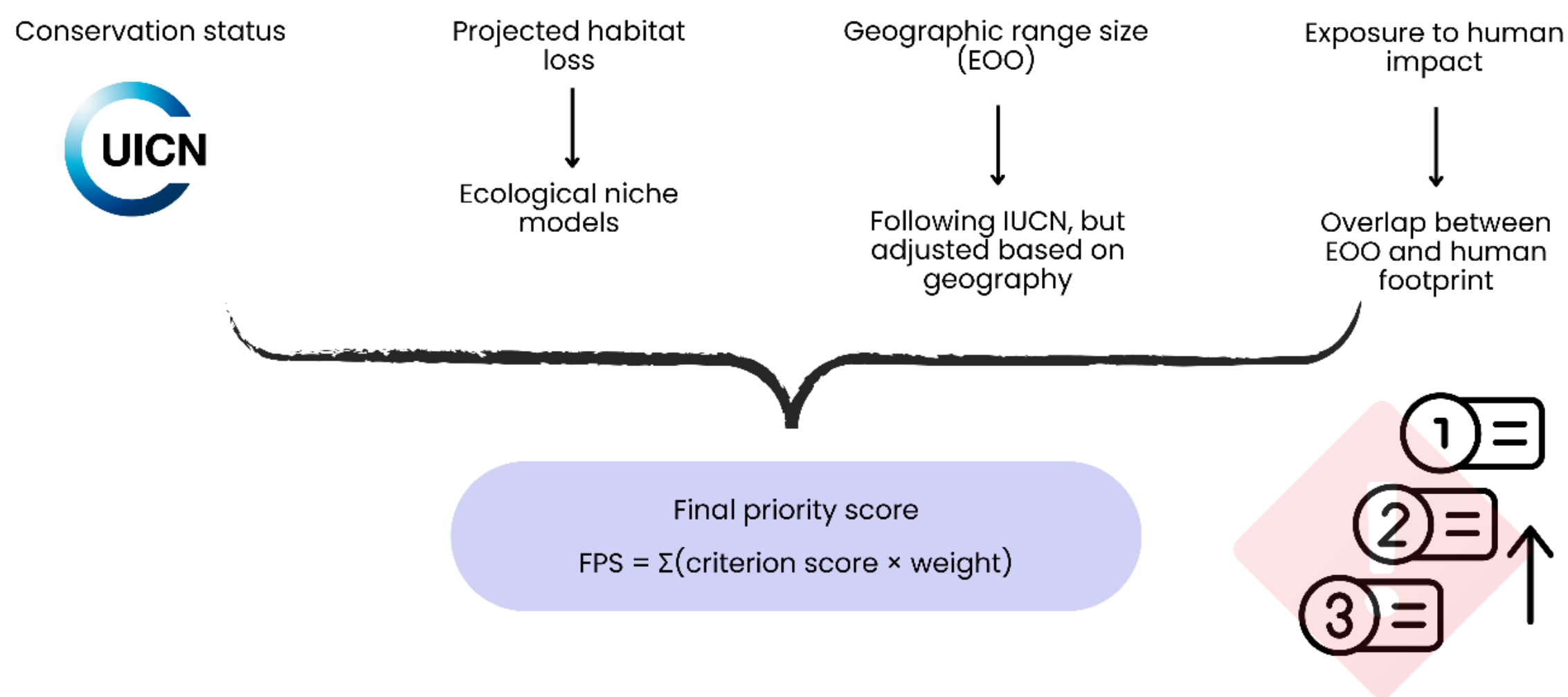
Conservation status
UICN
Projected habitat loss
Ecological niche models
Geographic range size (EOO)
Following IUCN, but adjusted based on geography
Exposure to human impact
Overlap between EOO and human footprint
Final priority score
FPS = Σ(criterion score × weight)
1
2
3

Figure 137. Selected criteria for assessing the priority level of hidden species complexes. All criteria were based on IUCN standards.

Conservation status

UICN

Projected habitat loss → Ecological niche models

Geographic range size (EOO) → Following IUCN, but adjusted based on geography

Exposure to human impact → Overlap between EOO and human footprint

Weight

25%

Amenazado

	CR	EN	VU	NT	LC	DD	NE (No Evaluado)
**Score**	7	6	5	4	3	4	1

Figure 138. Criteria 1: IUCN category. Here, we used the official IUCN category for the taxon currently recognized as a species by the IUCN, even though our analyses reveal it contains hidden species. According to the category, a score is assigned that will be used to calculate the priority index.

3.2 Scoring matrix

Conservation status

UICN

Projected habitat loss

↓

Ecological niche models

Geographic range size (EOO)

↓

Following IUCN, but adjusted based on geography

Exposure to human impact

↓

Overlap between EOO and human footprint

Weight

25%

	Score
Extreme > 80%	**5**
Severe 60-79%	**4**
Moderate 40-59%	**3**
Low 20-39%	**2**
Minimal < 20%	**1**

Figure 139. Criteria 2: Projected habitat loss. A score is assigned based on the percentage of loss.

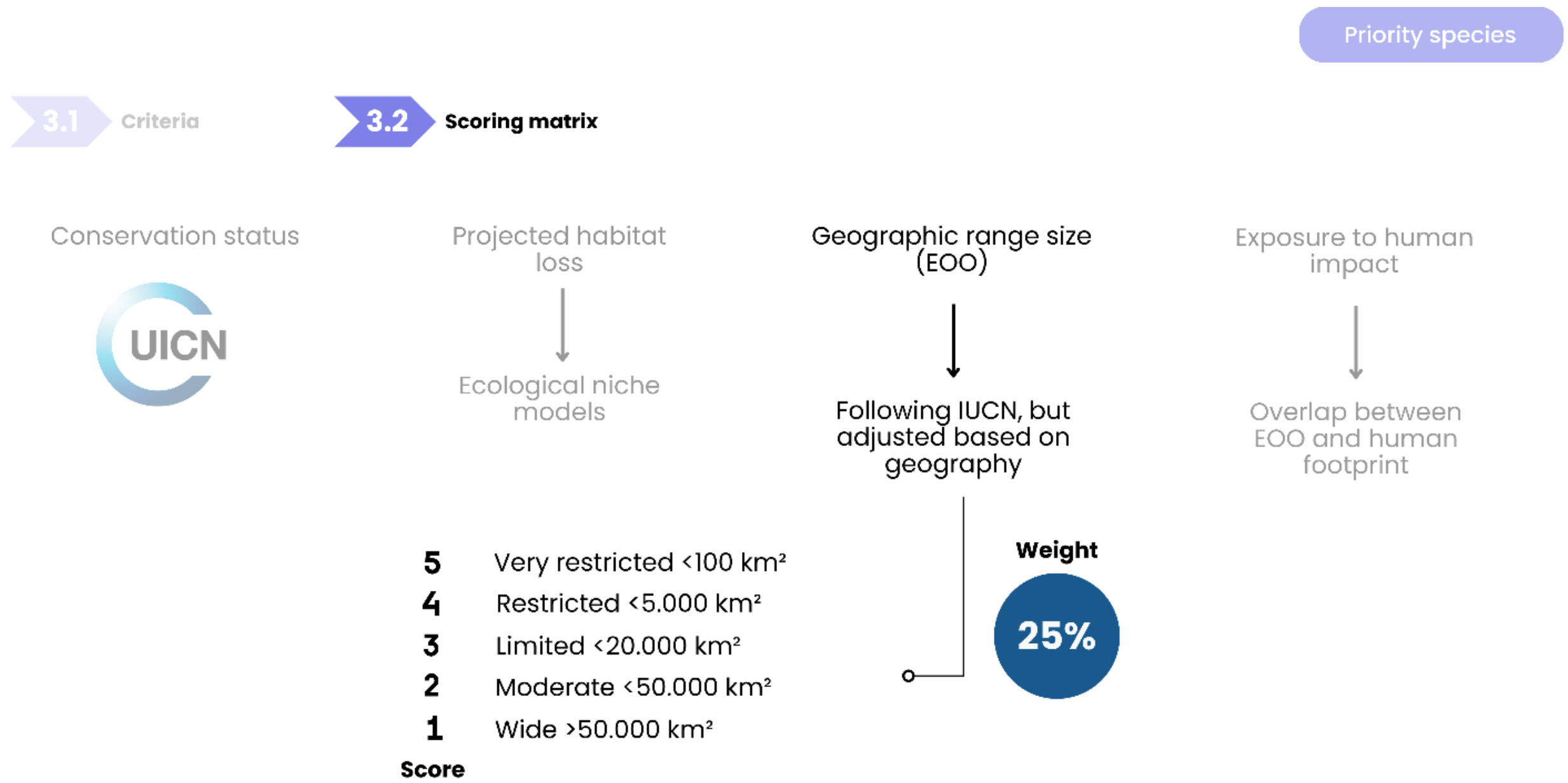

Figure 140. Criteria 3: EOO as a proxy for geographic distribution. Wide distributions receive low scores, while restricted distributions receive high scores.

Conservation status

UICN

Projected habitat loss

↓

Ecological niche models

Geographic range size (EOO)

↓

Following IUCN, but adjusted based on geography

Exposure to human impact

↓

Overlap between EOO and human footprint

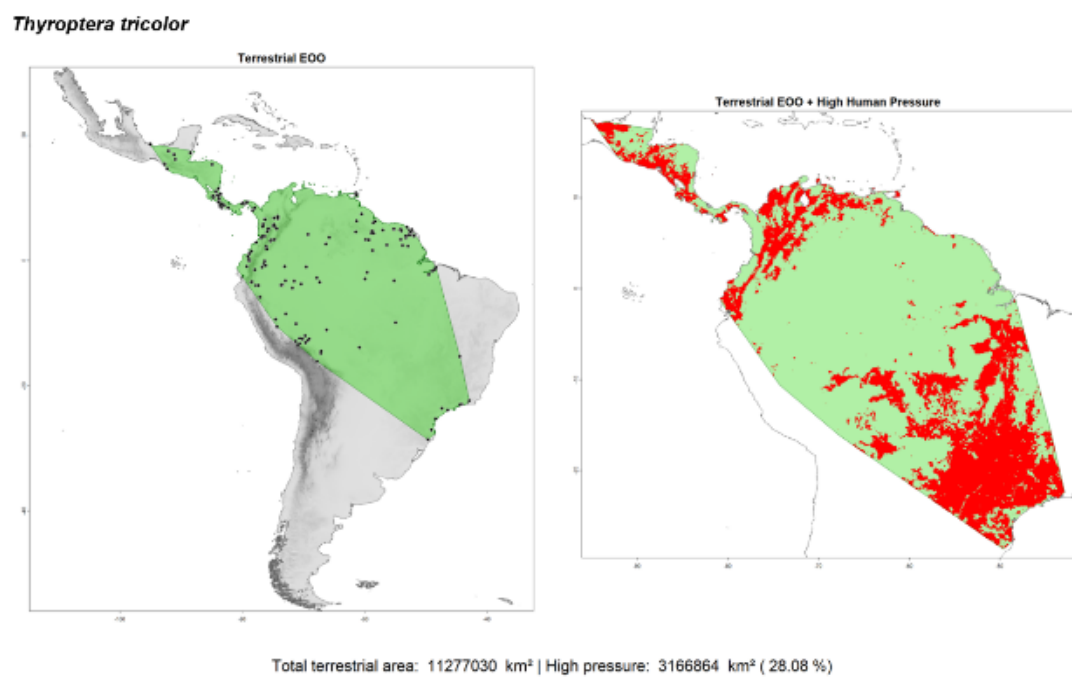

Score	
5	Extreme > 80%
4	Severe 60-79%
3	Moderate 40-59%
2	Low 20-39%
1	Minimal < 20%

Weight

25%

Figure 141. Criteria 4: Proportion of EOO affected by strong human impacts. A score is assigned according to the proportion of affected area.

Supplementary Table 1. Summary of species delimitation results and potential hidden diversity by genus. For each genus, we present the current number of recognized Neotropical species, the number of species analyzed in this study based on the validated sequences, and the potential hidden species identified through species delimitation approaches (ASAP, ABGD, bPTP, mPTP, and GMYC). Number of species complex identified represents the potential number of lineages recognized as harboring hidden species; consensus indicates the number of different lineages in the species complex consistently recovered across five or most methods, while newly estimated number of Neotropical species represents the updated species count including the potential hidden lineages recognized here. Asterisks: * Insufficient Data, ** Only species with/or over 70% Neotropical distribution. For phylogenies, maps, and hidden species identified for each genus refer to **fig. S1-S45**.

Family	Genus	Current Number of Recognized Neotropical Species	Number Species Analyzed	Number Species Complexes identified	ASAP	ABGD	bPTP	mPTP	GYMC	Consensus	Newly Estimated Number of Neotropical Species	Obs.
					How many lineages are hidden within the complex?							
**Thyropteridae**	Thyroptera	5	1	1	3	3	3	3	3	3	7	
**Furipteridae**	Furipterus	2	1	1	3	2	1	1	2	2	3	1!
**Natalidae**	Natalus	11	1	0							11	
**Noctilionidae**	Noctilio	2	2	2	7	7	8	7	7	7	7	
**Mormoopidae**	Mormoops	2	2	0							2	
	Pteronotus	16	13	2	5	5	5	2	7	5	19	
**Emballonuridae**	Balantiopteryx	3	2	0							3	
	Centronycteris*	2	0	0							2	
	Cormura	1	1	1	2	3	3	2	3	3	3	
	Cyttarops	1	1	1	1	2	2	1	2	2	2	
	Diclidurus	4	2	1	1	2	2	1	2	2	5	

	Peropteryx	5	2	1	2	2	2	2	2	2	7	**2!**
	Rhynchonycteris	1	1	1	2	2	3	2	2	2	2	
	Saccopteryx	5	2	1	4	4	4	4	5	4	8	
**Phyllostomidae**												
	Ametrida	1	1	0							1	
	Anoura	10	7	4	4	9	10	18	9	9	15	**3!**
	Ardops*	1	0	0							1	
	Ariteus*	1	0	0							1	
	Artibeus	13	9	0							13	
	Brachyphylla*	2	0	0							2	
	Carollia	8	5	1	3	1	3	3	3	3	10	
	Centurio	1	1	0							1	
	Chiroderma	7	7	0							7	
	Choeroniscus	3	2	1	2	3	1	1	3	2	4	**4!**
	Choeronycteris*	1	0	0							1	
	Chrotopterus	1	1	1	3	3	3	3	6	3	3	
	Dermanura	11	7	2	6	5	5	5	5	5	14	
	Desmodus	1	1	1	6	6	1	1	8	6	6	**5!**
	Diaemus*	1	0	0							1	
	Diphylla*	1	0	0							1	
	Dryadonycteris*	1	0	0							1	
	Ectophylla*	1	0	0							1	
	Enchisthenes*	1	0	0							1	
	Erophylla	2	1	0							2	
	Gardnerycteris	3	1	0							3	
	Glossophaga	9	3	0							9	
	Glyphonycteris	3	2	1	2	1	2	1	2	2	4	
	Hsunycteris	4	1	1	2	3	3	3	3	3	6	
	Hylonycteris	1	1	1	3	3	3	1	3	3	3	
	Lampronycteris	1	1	0							1	
	Leptonycteris*	3	0	0							3	

Lichonycteris*	2	0	0							2	
Lionycteris	1	1	0							1	
Lonchophylla	13	1	0							13	
Lonchorhina	6	1	0							6	
Lophostoma	8	6	1	3	3	4	3	3	3	11	
Macrophyllum	1	1	1	3	3	4	1	4	3	3	6!
Macrotus	2	2	0							2	
Mesophylla	1	1	0							1	
Micronycteris	13	4	0							13	
Mimon	2	1	0							2	
Monophyllus	2	1	1	2	2	4	1	2	2	3	
Musonycteris*	1	0	0							1	
Neonycteris*	1	0	0							1	
Phylloderma	1	1	1	4	4	1	1	4	4	4	
Phyllonycteris*	2	0	0							2	
Phyllops	1	1	0							1	
Phyllostomus	4	3	1	3	1	9	3	3	3	6	
Platalina*	1	0	0							1	
Platyrrhinus	19	9	0							19	
Pygoderma*	1	0	0							1	
Rhinophylla	3	1	0							3	
Scleronycteris*	1	0	0							1	
Sphaeronycteris*	1	0	0							1	
Stenoderma*	1	0	0							1	
Sturnira	25	9	0							25	
Tonatia	3	1	1	2	2	2	2	2	2	4	
Trachops	3	3	1	5	9	9	8	13	9	9	7!
Trinycteris	1	1	1	2	3	2	2	2	2	2	
Uroderma	5	2	0							5	
Vampyressa	6	2	0							6	
Vampyriscus	3	3	0							3	
Vampyrodes	2	2	0							2	

	Vampyrum	1	1	0							1	
	Xeronycteris*	1	0	0							1	
**Molossidae**	Cabreramops*	1	0	0							1	
	Cynomops	9	3	0							9	
	Eumops**	15	4	1	1	2	2	2	2	2	16	
	Molossops	3	2	1	2	2	2	2	2	2	4	
	Molossus	16	10	0							16	
	Mormopterus*	3	0	0							3	
	Neoplatymops*	1	0	0							1	
	Nyctinomops**	3	3	2	5	6	6	5	6	6	7	
	Promops	3	2	0							3	
	Tomopeas*	1	0	0							1	
**Vespertilionidae**	Baeodon*	2	0	0							2	
	Bauerus*	1	0	0							1	
	Corynorhinus**	2	0	0							2	
	Eptesicus**	1	0	0							1	
	Histiotus	11	7	2	5	6	1	1	4	4	15	**8!**
	Lasiurus**	14	3	1	1	2	2	1	3	2	15	**9!**
	Myotis**	36	10	2	17	17	17	15	9	17	51	
	Neoeptesicus	10	4	3	9	9	11	11	14	9	16	**10!**
	Nycticeius**	1	0	0							1	
	Rhogeessa	12	3	0							12	
	**Total**	**435**	**191**	**45**							**530**	

Observations

1! Partial agreement: same two lineages recovered by the majority of algorithms.

2! an additional unnamed lineage recognized as sp. from Ecuador increases the total to seven.

3! Partial agreement: same nine lineages recovered by the majority of algorithms.

4! Partial agreement: same two lineages recovered by the majority of algorithms.

5! Partial agreement: same six lineages recovered by the majority of algorithms.

6! Partial agreement: same three lineages recovered by the majority of algorithms.

7! Partial agreement: same nine lineages recovered by the majority of algorithms.

8! Delimitation methods recovered two complexes comprising four lineages; however, two additional unnamed lineages recognized increases the total to 15.

9! Partial agreement: same two lineages recovered by the majority of algorithms.

10! Partial agreement: same nine lineages recovered by the majority of algorithms.

Supplementary Table 2. Hidden species complex by provinces.

Provinces	num_hidden_species	species_list
Atlantic province	8	Furipterus_horrens, Molossops_neglectus, Myotis_nigricans, Myotis_riparius, Myotis_spBrazil, Neoeptesicus_furinalis, Nyctinomops_laticaudatus, Nyctinomops_macrotis
Caatinga province	1	Pteronotus_personatus
Cauca province	4	Anoura_cultrata, Carollia_castanea, Dermanura_watsoni, Phylloderma_stenops
Cerrado province	5	Lophostoma_silvicola, Myotis_spBrazil, Neoeptesicus_furinalis, Nyctinomops_laticaudatus, Pteronotus_personatus
Chaco province	4	Neoeptesicus_furinalis, Noctilio_albiventris, Noctilio_leporinus, Nyctinomops_laticaudatus
Chiapas Highlands province	2	Anoura_peruana, Desmodus_rotundus
Choco-Darien province	3	Carollia_castanea, Dermanura_anderseni, Phyllostomus_hastatus
Desert province	1	Histiotus_montanus
Ecuadorian province	1	Noctilio_leporinus
Esteros del Ibera province	2	Histiotus_montanus, Noctilio_albiventris
Guajira province	4	Anoura_caudifer, Anoura_cultrata, Anoura_luismanueli, Anoura_peruana
Guatuso-Talamanca province	21	Anoura_cultrata, Anoura_peruana, Carollia_castanea, Dermanura_anderseni, Dermanura_watsoni, Desmodus_rotundus, Hylonycteris_underwoodi, Lophostoma_silvicola, Macrophyllum_macrophyllum, Myotis_nigricans, Myotis_riparius, Neoeptesicus_chiriquinus, Noctilio_albiventris, Noctilio_leporinus, Phylloderma_stenops, Phyllostomus_hastatus, Rhynchonycteris_naso, Saccopteryx_bilineata, Thyroptera_tricolor, Trachops_cirrhosus, Trinycteris_nicefori
Guianan Lowlands province	31	Anoura_caudifer, Choeroniscus_minor, Chrotopterus_auritus, Cormura_brevirostris, Cyttarops_alecto, Desmodus_rotundus, Diclidurus_albus, Eumops_hansae, Furipterus_horrens, Glyphonycteris_silvestris, Hsunycteris_thomasi, Lophostoma_silvicola, Macrophyllum_macrophyllum, Molossops_neglectus, Myotis_nigricans, Myotis_riparius, Neoeptesicus_chiriquinus, Neoeptesicus_furinalis, Noctilio_albiventris, Noctilio_leporinus, Nyctinomops_macrotis, Peropteryx_macrotis, Phylloderma_stenops, Phyllostomus_hastatus, Pteronotus_personatus, Rhynchonycteris_naso, Saccopteryx_bilineata, Thyroptera_tricolor, Tonatia_maresi, Trachops_cirrhosus, Trinycteris_nicefori
Guianan province	17	Anoura_caudifer, Choeroniscus_minor, Chrotopterus_auritus, Cormura_brevirostris, Desmodus_rotundus, Hsunycteris_thomasi, Lophostoma_silvicola, Myotis_nigricans, Myotis_riparius, Noctilio_albiventris, Noctilio_leporinus, Phylloderma_stenops, Rhynchonycteris_naso, Saccopteryx_bilineata, Tonatia_maresi, Trachops_cirrhosus, Trinycteris_nicefori
Hispaniola province	2	Monophyllus_redmani, Pteronotus_pusillus
Imeri province	2	Nyctinomops_laticaudatus, Nyctinomops_macrotis
Jamaica province	2	Monophyllus_redmani, Noctilio_leporinus
Lesser Antilles province	1	Noctilio_leporinus
Magdalena province	4	Carollia_castanea, Dermanura_anderseni, Desmodus_rotundus, Myotis_riparius
Napo province	23	Anoura_caudifer, Carollia_castanea, Choeroniscus_minor, Chrotopterus_auritus, Cormura_brevirostris, Dermanura_anderseni, Desmodus_rotundus, Eumops_hansae, Furipterus_horrens, Hsunycteris_thomasi, Lophostoma_silvicola, Macrophyllum_macrophyllum, Myotis_nigricans, Myotis_riparius, Neoeptesicus_furinalis, Phylloderma_stenops, Phyllostomus_hastatus, Rhynchonycteris_naso, Saccopteryx_bilineata, Thyroptera_tricolor, Tonatia_maresi, Trachops_cirrhosus, Trinycteris_nicefori

Pacific Lowlands province	5	Desmodus_rotundus, Myotis_riparius, Noctilio_albiventris, Noctilio_leporinus, Saccopteryx_bilineata
Para province	3	Neoeptesicus_furinalis, Noctilio_albiventris, Trinycteris_nicefori
Paramo province	5	Anoura_caudifer, Anoura_luismanueli, Anoura_peruana, Glyphonycteris_silvestris, Noctilio_albiventris
Parana Forest province	11	Chrotopterus_auritus, Desmodus_rotundus, Histiotus_velatus, Lasiurus_ega, Molossops_neglectus, Myotis_riparius, Myotis_spBrazil, Neoeptesicus_furinalis, Nyctinomops_laticaudatus, Nyctinomops_macrotis, Phyllostomus_hastatus
Puna province	1	Myotis_nigricans
Puntarenas-Chiriqui province	9	Anoura_peruana, Carollia_castanea, Desmodus_rotundus, Hylonycteris_underwoodi, Myotis_riparius, Neoeptesicus_brasiliensis, Noctilio_albiventris, Noctilio_leporinus, Rhynchonycteris_naso
Rondonia province	5	Lasiurus_ega, Myotis_riparius, Neoeptesicus_furinalis, Noctilio_albiventris, Nyctinomops_laticaudatus
Roraima province	6	Chrotopterus_auritus, Cormura_brevirostris, Hsunycteris_thomasi, Myotis_riparius, Neoeptesicus_furinalis, Phylloderma_stenops
Sabana province	7	Desmodus_rotundus, Neoeptesicus_chiriquinus, Noctilio_albiventris, Phyllostomus_hastatus, Rhynchonycteris_naso, Saccopteryx_bilineata, Trachops_cirrhosus
Trinidad province	1	Neoeptesicus_chiriquinus
Ucayali province	5	Anoura_cultrata, Anoura_peruana, Carollia_castanea, Dermanura_anderseni, Noctilio_albiventris
Venezuelan province	4	Anoura_caudifer, Anoura_cultrata, Anoura_peruana, Trachops_cirrhosus
Veracruzan province	11	Chrotopterus_auritus, Desmodus_rotundus, Diclidurus_albus, Eumops_hansae, Lasiurus_ega, Neoeptesicus_brasiliensis, Neoeptesicus_furinalis, Pteronotus_personatus, Rhynchonycteris_naso, Saccopteryx_bilineata, Trachops_cirrhosus
Western Ecuador province	1	Noctilio_leporinus
Xingu-Tapajos province	1	Pteronotus_personatus
Yucatan Peninsula Province	7	Chrotopterus_auritus, Desmodus_rotundus, Neoeptesicus_furinalis, Nyctinomops_laticaudatus, Peropteryx_macrotis, Saccopteryx_bilineata, Trachops_cirrhosus
Yungas province	6	Histiotus_sp1, Histiotus_velatus, Myotis_nigricans, Myotis_riparius, Neoeptesicus_chiriquinus, Neoeptesicus_furinalis

Supplementary Table 3. Biogeographic province metrics for Hidden Species Ratio (HSR) calculation. This table presents the comprehensive dataset used to develop the Hidden Species Index across biogeographic provinces. Columns include geographic accessibility metrics (median distances to roads, cities, and protected areas), observed hidden diversity (hidden species), sampling effort (n points), standardized accessibility (Z accessibility), area metrics, and the resulting HSR score. The HSR integrates hidden species counts, sampling intensity, spatial biases, and geographic accessibility factors to provide a standardized measure of potential undiscovered diversity across regions. For detailed methodology on HSR calculation and implementation, refer to the formulas and procedures developed in the main text.

Provinces	median_dist_road	median_dist_city	median_dist_pa	n_points	mean_accessibility	mean_accessibility_log	Z_accessibility	area_km2	area_km2_log	Z_size	num_hidden_species	num_genetic_sequences	base_ratio	adjustment_factor	HSR
Guianan Lowlands province	101.819	162.134	0.156	6151	88.036	4.489	1.745	550446	13.218	1.215	31	6151	3.553	1.592	5.657
Napo province	129.763	66.158	0	997	65.307	4.194	1.4	265250	12.488	0.401	23	997	3.331	1.36	4.531
Guianan province	104.243	150.125	13.646	1174	89.338	4.504	1.762	745410	13.522	1.554	17	1174	2.405	1.663	4
Guatuso-Talamanca province	5.644	27.491	0.059	375	11.065	2.49	-0.594	78463	11.27	-0.957	21	375	3.542	0.69	2.443
Imeri province	78.066	80.021	0	3	52.695	3.983	1.153	702031	13.462	1.487	2	3	1.443	1.528	2.204
Yungas province	9.275	69.918	3.474	32	27.556	3.352	0.414	391705	12.878	0.836	6	32	1.716	1.25	2.145
Roraima province	47.822	413.065	0	151	153.629	5.041	2.391	622711	13.342	1.353	6	151	1.194	1.749	2.089
Ucayali province	11.52	41.08	17.949	12	23.516	3.199	0.236	192906	12.17	0.046	5	12	1.949	1.056	2.059
Parana Forest province	5.068	10.601	1.01	366	5.56	1.881	-1.307	720279	13.487	1.515	11	366	1.863	1.042	1.94
Rondonia province	49.382	131.378	1.705	89	60.822	4.124	1.318	1316365	14.09	2.188	5	89	1.111	1.701	1.89
Chaco province	5.457	132.851	0	46	46.102	3.852	1	1010604	13.826	1.893	4	46	1.039	1.579	1.64
Veracruzan province	6.485	19.682	0.21	516	8.792	2.282	-0.838	266082	12.492	0.405	11	516	1.761	0.913	1.608
Sabana province	7.649	17.233	0	93	8.294	2.229	-0.899	435815	12.985	0.955	7	93	1.541	1.011	1.558
Desert province	2.912	30.309	14.923	1	16.048	2.836	-0.189	185045	12.128	0	1	1	1.443	0.962	1.388
Cerrado province	1.937	8.683	7.864	110	6.162	1.969	-1.204	1940140	14.478	2.62	5	110	1.062	1.283	1.362
Puntarenas-Chiriqui province	5.133	41.346	9.855	144	18.778	2.985	-0.015	45555	10.727	-1.563	9	144	1.808	0.684	1.238
Puna province	5.187	42.679	3.534	2	17.133	2.898	-0.117	622870	13.342	1.353	1	2	0.91	1.247	1.135

Atlantic province	4.112	4.579	1.627	222	3.439	1.491	-1.764	282387	12.551	0.471	8	222	1.48	0.742	1.097
Yucatan Peninsula Province	1.4	54.557	1.16	491	19.039	2.998	0	133173	11.799	-0.367	7	491	1.129	0.927	1.046
Venezuelan province	0.018	24.503	0	22	8.174	2.216	-0.914	124204	11.73	-0.445	4	22	1.276	0.728	0.929
Esteros del Ibera province	1.115	34.883	0.357	6	12.118	2.574	-0.496	167378	12.028	-0.112	2	6	1.028	0.878	0.903
Paramo province	1.886	12.248	10.176	136	8.103	2.209	-0.923	219438	12.299	0.19	5	136	1.016	0.853	0.867
Guajira province	2.7	5.908	0	14	2.869	1.353	-1.924	148904	11.911	-0.242	4	14	1.477	0.567	0.837
Pacific Lowlands province	0.755	21.499	3.205	355	8.486	2.25	-0.875	270032	12.506	0.421	5	355	0.851	0.909	0.774
Choco-Darien province	13.14	109.351	0	61	40.83	3.734	0.861	81715	11.311	-0.911	3	61	0.727	0.99	0.72
Magdalena province	3.739	11.527	9.152	101	8.139	2.213	-0.919	195292	12.182	0.06	4	101	0.865	0.828	0.716
Cauca province	10.726	32.808	8.435	163	17.323	2.908	-0.105	119631	11.692	-0.486	4	163	0.784	0.882	0.692
Hispaniola province	28.141	764.098	0.96	126	264.4	5.581	3.023	75469	11.231	-1	2	126	0.413	1.405	0.58
Para province	2.788	13.342	0	176	5.377	1.853	-1.34	359297	12.792	0.74	3	176	0.58	0.88	0.51
Jamaica province	2.683	792.367	4.127	99	266.392	5.589	3.032	11056	9.311	-3.142	2	99	0.434	0.978	0.425
Caatinga province	7.189	47.08	13.061	34	22.443	3.155	0.184	730348	13.501	1.531	1	34	0.281	1.343	0.378
Xingu-Tapajos province	111.258	49.401	0	78	53.553	3.999	1.172	1022563	13.838	1.906	1	78	0.229	1.616	0.37
Chiapas Highlands province	6.735	12.903	0	104	6.546	2.021	-1.143	133831	11.804	-0.361	2	104	0.43	0.699	0.3
Ecuadorian province	6.49	25.116	0.186	21	10.597	2.451	-0.64	41366	10.63	-1.67	1	21	0.324	0.538	0.174
Lesser Antilles province	4.474	399.53	0.519	61	134.841	4.911	2.239	6445	8.771	-3.743	1	61	0.242	0.699	0.169
Western Ecuador province	15.939	15.686	5.729	59	12.451	2.599	-0.466	64684	11.077	-1.172	1	59	0.244	0.672	0.164
Trinidad province	23.328	121.703	0.161	14	48.397	3.9	1.056	5198	8.556	-3.983	1	14	0.369	0.415	0.153
Araucaria Forest province	7.238	5.868	3.681	3	5.596	1.886	-1.3	216062	12.283	0.173	0	3	0	0.775	0
Balsas Basin province	2.39	8.379	13.595	2	8.121	2.211	-0.921	76597	11.246	-0.983	0	2	0	0.619	0

Cayman Islands province	0.629	624.456	0.771	10	208.619	5.345	2.747	279	5.634	-7.242	0	10	0	0.101	0
Chapada Diamantina province	23.596	40.464	0	5	21.354	3.107	0.128	49687	10.814	-1.466	0	5	0	0.732	0
Cuban province	15.617	960.753	9.944	1	328.771	5.798	3.277	110583	11.614	-0.574	0	1	0	1.541	0
Madeira province	21.251	125.705	0	23	48.985	3.912	1.07	1335048	14.104	2.203	0	23	0	1.655	0
Mosquito province	3.209	25.648	0	8	9.619	2.363	-0.743	114050	11.644	-0.54	0	8	0	0.743	0
N/A	40.938	42.82	30.152	2	37.97	3.663	0.778	1137160	13.944	2.024	0	2	0	1.561	0
Pampean province	0.761	20.507	9.943	6	10.404	2.434	-0.66	968326	13.783	1.845	0	6	0	1.237	0
Puerto Rico province	1.793	796.445	0.272	16	266.17	5.588	3.031	9534	9.163	-3.307	0	16	0	0.945	0
Sierra Madre Occidental province	10.949	229.249	119.81	9	120.003	4.796	2.104	172795	12.06	-0.076	0	9	0	1.406	0
Sierra Madre Oriental province	88.49	223.189	145.605	6	152.428	5.033	2.382	52537	10.869	-1.404	0	6	0	1.196	0
Sierra Madre del Sur province	8.341	48.681	29.377	1	28.8	3.395	0.464	92811	11.438	-0.769	0	1	0	0.939	0
Southern Espinhaco province	2.618	7.567	6.865	2	5.683	1.9	-1.285	61233	11.022	-1.233	0	2	0	0.496	0

Supplementary Table 4. Uncorrelated bioclimatic variables used to calibrate ENMs.

Species complex	Uncorrelated bioclimatic variables							
Anoura caudifer	1	3	7	12	15			
Anoura cultrata	1	3	7	12	17			
Anoura luismanueli	1	2	3	7	12			
Anoura peruana	1	3	7	12	15			
Carollia castanea	1	3	7	12	15			
Choeroniscus minor	1	3	4	7	12	13	17	
Chrotopterus auritus	1	7	12	17				
Cormura brevirostris	1	3	4	7	12	16	17	
Cyttarops alecto	1	3	7	12	17			
Dermanura anderseni	1	2	3	12	15			
Dermanura watsoni	1	3	7	12				
Desmodus rotundus	1	7	12					
Diclidurus albus	1	3	5	7	12	15		
Eumops hansae	1	3	7	12	17			
Furipterus horrens	1	3	4	5	7	12	13	17
Glyphonycteris sylvestris	1	3	5	7	12	15		
Histiotus montanus	1	2	3	7	12	15		
Histiotus velatus	1	2	3	7	12	15	16	
Hsunycteris thomasi	1	3	5	7	12	17		
Hylonycteris underwoodi	1	3	5	12	17			
Lasiurus ega	1	7	12	15				
Lophostoma silvicola	1	3	5	7	13	14		
Macrophyllum macrophyllum	1	3	4	5	7	13	14	
Myotis nigricans	1	3	5	12	17			
Myotis riparius	1	2	3	12	15			
Neoeptesicus brasiliensis	1	3	12	17				
Neoeptesicus chiriquinus	1	3	7	12	17			
Neoeptesicus furinalis	1	2	3	5	12	15		
Noctilio albiventris	1	3	7	12	17			
Noctilio leporinus	1	5	7	12	15			
Nyctinomops laticaudatus	1	2	3	5	12	15		
Nyctinomops macrotis	1	2	3	10	12	15	17	
Peropteryx macrotis	1	2	3	12	17			
Phylloderma stenops	1	3	4	7	12	15		
Phyllostomus hastatus	1	3	7	12	14			
Pteronotus personatus	1	3	5	7	12	13	17	
Rhynchonycteris naso	1	3	4	5	7	12	17	
Saccopteryx bilineata	1	3	5	7	12	17		
Thyroptera tricolor	1	3	7	12	13	17		
Tonatia maresi	1	3	7	12	13	17		

Trachops cirrhosus	1	3	5	7	12	17		
Trinycteris nicefori	1	3	7	12	13	17		
