# Supplementary material for: Substantial Neotropical bat diversity likely remains undescribed and vulnerable to extinction
Source: Nat Commun. 2026 Nov 3;17:11368. doi: 10.1038/s41467-026-78393-0 (PMC13731659; doi:10.1038/s41467-026-78393-0)
Supplement: Supplementary file 2 [file 41467_2026_78393_MOESM2_ESM.pdf]

Supplementary Data 1.

Genetic data sources and accession information, including GenBank accession numbers and BOLD IDs for all curated sequences analysed, together with associated specimen voucher, taxonomic, and geographic information.

Supplementary Data 2.

Genetic sequences in FASTA analysed in this study.
