# Supplementary material for: Substantial Neotropical bat diversity likely remains undescribed and vulnerable to extinction
Source: Nat Commun. 2026 Nov 3;17:11368. doi: 10.1038/s41467-026-78393-0 (PMC13731659; doi:10.1038/s41467-026-78393-0)
Supplement: Supplementary file 3 [file 41467_2026_78393_MOESM5_ESM.pdf]

nature portfolio

Corresponding author(s): Diego A. Esquivel

Last updated by author(s): Aug 11, 2026

Reporting Summary

Nature Portfolio wishes to improve the reproducibility of the work that we publish. This form provides structure for consistency and transparency in reporting. For further information on Nature Portfolio policies, see our Editorial Policies and the Editorial Policy Checklist.

Statistics

For all statistical analyses, confirm that the following items are present in the figure legend, table legend, main text, or Methods section.

n/a	Confirmed	
[ ]	[x]	The exact sample size (n) for each experimental group/condition, given as a discrete number and unit of measurement
[x]	[ ]	A statement on whether measurements were taken from distinct samples or whether the same sample was measured repeatedly
[ ]	[x]	The statistical test(s) used AND whether they are one- or two-sided<br>*Only common tests should be described solely by name; describe more complex techniques in the Methods section.*
[ ]	[x]	A description of all covariates tested
[ ]	[x]	A description of any assumptions or corrections, such as tests of normality and adjustment for multiple comparisons
[ ]	[x]	A full description of the statistical parameters including central tendency (e.g. means) or other basic estimates (e.g. regression coefficient) AND variation (e.g. standard deviation) or associated estimates of uncertainty (e.g. confidence intervals)
[ ]	[x]	For null hypothesis testing, the test statistic (e.g. F, t, r) with confidence intervals, effect sizes, degrees of freedom and P value noted<br>*Give P values as exact values whenever suitable.*
[x]	[ ]	For Bayesian analysis, information on the choice of priors and Markov chain Monte Carlo settings
[x]	[ ]	For hierarchical and complex designs, identification of the appropriate level for tests and full reporting of outcomes
[ ]	[x]	Estimates of effect sizes (e.g. Cohen's d, Pearson's r), indicating how they were calculated

Our web collection on statistics for biologists contains articles on many of the points above.

Software and code

Policy information about availability of computer code

Data collection	Genetic sequence data were obtained from publicly available databases using custom Python script. Occurrence and geographic data were compiled from publicly available databases and museum collections. Sequence and occurrence datasets were subsequently cleaned, filtered, and quality-controlled using R [v. 4.5] and Python [v. 3.14]. The specific databases, data sources, filtering criteria, and procedures are described in detail in the Methods and Supplementary Information. Data and code supporting the findings of this study are provided on the public GitHub repository (https://github.com/diegodaem/)
Data analysis	Phylogenetic analyses were conducted using MAFFT [v. 7.505], trimAl [v. 1.2], ModelFinder [v. 2.2], IQ-TREE [v. 2.4], BEAST/BEAUti [v. 2.7.6], Tracer [v. 1.7.2], LogCombiner, TreeAnnotator [v. 2.6.3], and FigTree [v. 1.4.4]. Molecular species delimitation was performed using ABGD, ASAP, bPTP, mPTP, and GMYC. Spatial analyses included spThin [v.0.2] and chooseGCM [v.1.3], and ecological niche models were developed using kuenm and MaxEnt [v. 3.4.4]. Statistical, spatial, and data-processing analyses were conducted in R [v. 4.5] and Python [v. 3.14]. The Hidden Species Richness (HSR) Index was implemented in R using custom code developed by the authors and is available under an MIT licence on GitHub. Detailed analytical procedures are provided in the Methods and Supplementary Information. Data and code supporting the findings of this study are provided on the public GitHub repository (https://github.com/diegodaem/)

For manuscripts utilizing custom algorithms or software that are central to the research but not yet described in published literature, software must be made available to editors and reviewers. We strongly encourage code deposition in a community repository (e.g. GitHub). See the Nature Portfolio guidelines for submitting code & software for further information.

Data

Policy information about availability of data

All manuscripts must include a data availability statement. This statement should provide the following information, where applicable:

- Accession codes, unique identifiers, or web links for publicly available datasets
- A description of any restrictions on data availability
- For clinical datasets or third party data, please ensure that the statement adheres to our policy

All data generated in this study are provided in the Supplementary Information. Data and code supporting the findings of this study are provided on the public GitHub repository (https://github.com/diegodaem/). It is also possible to request them from the corresponding author.

Research involving human participants, their data, or biological material

Policy information about studies with human participants or human data. See also policy information about sex, gender (identity/presentation), and sexual orientation and race, ethnicity and racism.

Reporting on sex and gender	This information has not been collected.
Reporting on race, ethnicity, or other socially relevant groupings	This information has not been collected.
Population characteristics	This information has not been collected.
Recruitment	This information has not been collected.
Ethics oversight	This information has not been collected.

Note that full information on the approval of the study protocol must also be provided in the manuscript.

Field-specific reporting

Please select the one below that is the best fit for your research. If you are not sure, read the appropriate sections before making your selection.

☐ Life sciences ☐ Behavioural & social sciences ☒ Ecological, evolutionary & environmental sciences

For a reference copy of the document with all sections, see nature.com/documents/nr-reporting-summary-flat.pdf

Ecological, evolutionary & environmental sciences study design

All studies must disclose on these points even when the disclosure is negative.

Study description	We conducted a large-scale, multi-method quantitative assessment to identify and prioritize hidden bat diversity across the Neotropical region. The study integrated four main analytical components: (1) curation of a mitochondrial COI dataset comprising 12,813 sequences representing 65 genera; (2) genus-level phylogenetic analyses and species delimitation using five complementary algorithms to identify hidden species; (3) spatial analyses to map occurrences and compute a Hidden Species Richness index across biogeographic provinces while correcting for sampling bias; and (4) ecological niche models for 45 species complexes under current and future climate scenarios (ssp245 and ssp585), using Maxent. Each species complex was treated as an experimental unit, and threat metrics (habitat loss, human modification, range size, and IUCN status) were integrated into a standardized scoring framework to derive conservation priority categories.
Research sample	The study relies on an assembled mitochondrial COI dataset comprising 12,813 sequences of Neotropical bats, representing 65 genera. Sequences were obtained from public repositories (GenBank and BOLD) and complemented with records from published sources. All sequences correspond to wild-caught individuals deposited in scientific collections or contributed by prior peer-reviewed studies. No organisms were captured or manipulated for this project.<br><br>The sample is intended to represent the full documented geographic and taxonomic breadth of Neotropical bat diversity. For each sequence, available metadata (locality, coordinates, voucher information, and collection source) were retained when reported by the original contributors. To ensure analytical reliability, sequences shorter than 500 bp, lacking genus identification, or flagged as problematic during quality control were excluded.
Sampling strategy	Our study did not employ predetermined sample size calculations. We conducted an exhaustive compilation of all available COI sequences for Neotropical bats from public repositories (GenBank and BoldSystems), rather than a prospective sampling design.
Data collection	We used genetic sequences collected by hundreds of different researchers in the Neotropical region. The analyses and other data processing were performed by the first author.
Timing and spatial scale	The genetic sequences used range from 1990 to 2025. They cover the entire Neotropical region as shown in the ms.
Data exclusions	Data were excluded from analyses based on pre-established quality control criteria (see Methods section: Quality Control).

Reproducibility	We ensured reproducibility through the following measures:<br>1. Data availability: All cleaned and filtered sequences are provided in Supplementary Data S1 and Supplementary Data S2.<br>2. Code availability: All custom Python scripts and R scripts are publicly available at https://github.com/diegodaem/HSR-Index.git with detailed documentation of our pipeline (Fig. 7).<br>3. Methodological transparency: We provide complete parameters for all analyses including:<br><br>Alignment settings (MAFFT with '--auto' strategy)<br>Phylogenetic analyses (IQ-TREE with 10,000 bootstraps; BEAST with 50 million generations)<br>Species delimitation methods (web server settings specified for ABGD, ASAP, GMYC, bPTP, mPTP)<br>ENM parameters (Maxent settings, regularization multipliers, feature classes)<br><br>4. Bayesian convergence verification: For BEAST analyses, we verified convergence by ensuring ASDSF <0.01, ESS >200, and PSRF approached 1. When these criteria were not met, analyses were re-run until convergence was achieved (see Methods section 1.2).<br>5. Model validation: ENM models were validated through cross-validation, partial ROC tests, and omission rate evaluations.
Randomization	Randomization is not applicable to our study. Our research is a comprehensive, non-experimental analysis of existing genetic sequences from public repositories. We did not allocate samples into treatment groups or experimental conditions. Instead, we analyzed all available COI sequences that met our quality criteria. Groupings were based on taxonomic identity (genus-level phylogenies) and biogeographic provinces, which are determined by biological and geographic factors, not experimental assignment.
Blinding	Blinding is not relevant to our study. Our analyses are based on computational and phylogenetic methods applied to genetic sequences with objective, algorithmically-determined outcomes. Species delimitation was performed using five independent algorithms (ABGD, ASAP, GMYC, bPTP, mPTP) with pre-defined parameters, and hidden species complexes were only recognized when ≥3 algorithms agreed. This conservative, algorithm-based approach eliminates subjective bias without requiring investigator blinding.

Did the study involve field work? ☐ Yes ☒ No

Reporting for specific materials, systems and methods

We require information from authors about some types of materials, experimental systems and methods used in many studies. Here, indicate whether each material, system or method listed is relevant to your study. If you are not sure if a list item applies to your research, read the appropriate section before selecting a response.

Materials & experimental systems

n/a	Involved in the study
☒	☐ Antibodies
☒	☐ Eukaryotic cell lines
☒	☐ Palaeontology and archaeology
☒	☐ Animals and other organisms
☒	☐ Clinical data
☒	☐ Dual use research of concern
☒	☐ Plants

Methods

n/a	Involved in the study
☒	☐ ChIP-seq
☒	☐ Flow cytometry
☒	☐ MRI-based neuroimaging

Plants

Seed stocks	Our study does not involve plant material or seed stocks.
Novel plant genotypes	Our study does not involve novel plant genotypes.
Authentication	Our study does not involve plant material or seed stocks.
