# Supplementary material for: Substantial Neotropical bat diversity likely remains undescribed and vulnerable to extinction
Source: Nat Commun. 2026 Nov 3;17:11368. doi: 10.1038/s41467-026-78393-0 (PMC13731659; doi:10.1038/s41467-026-78393-0)
Supplement: Supplementary file 4 [file 41467_2026_78393_MOESM6_ESM.pdf]

nature portfolio

Peer Review File

Substantial Neotropical bat diversity likely remains undescribed and vulnerable to extinction

Corresponding Author: Mr Diego Esquivel

This file contains all reviewer reports in order by version, followed by all author rebuttals in order by version.

Version 0:

Reviewer comments:

Reviewer #1

(Remarks to the Author)
Review: Racing Against Time to Unveil Hidden Bat Diversity
This is an elegant study that aims at identifying cryptic species diversity in Neotropical bats. Overall, the manuscript to be well written, the analytical methods seem robust and aligned with the research questions. The narrative flow from the introduction to the discussion is strong, with clear and convincing interpretations supported by the results. The figures are informative, well-presented and relevant and the work should appeal to a broad readership. The remarks below are intended as constructive feedback to further strengthen the presented work.

1. The title does not currently reflect the breadth of the study, and it would benefit from including the geographic area.

2. The introduction requires more depth: a greater exploration of cryptic species complexes is required, and further examples of the implications of data deficiency for conservation should be added. Additionally, the manuscript of benefit of Neotropical bats being presented as a case study for the study of cryptic diversity, and their treats. Currently, they are mentioned as having high levels of uncovered hidden diversity and in need of a comprehensive revision but are not put across as a suitable case study for the assessment of how cryptic diversity potentially impacts conservation in a more holistic manner.

3. ENMs could be strengthened by using an ensemble approach with multiple algorithms - currently only the MaxEnt algorithm is used. There are areas of the methods where greater detail and justification is needed, and the ENM approach can be refined by adding more algorithms and using an ensemble approach.

4. The discussion would greatly benefit from a broad concluding paragraph linking the findings of this bat-specific neotropics study to global scales and cryptic species complexes more generally.

Specific Comments

Title: The title should reflect that the study was carried out for the Neotropics. I assumed it was a global study until I reached line 92 of the manuscript.

Abstract

Line 28: Consider “the exceptional bat diversity of the Neotropical” instead of “the Neotropics' exceptional bat diversity”

Introduction

Line 52-53: Sentence is awkwardly phrased. The term “accelerated anthropogenic change” should be rephrased so that it lists biodiversity threats, of which one is anthropogenic disturbance.
Line 53-56: This is primarily due to unresolved cryptic species complexes, i.e., hidden biodiversity that can not be detected by traditional means. This warrants discussion here.
Line 58: Better rephrased to state that individual species are the primary currency of biodiversity (i.e., species richness).
Line 62: Is the sixth “mass extinction” really ongoing?
Line 66:70: There are also countless species that are not yet classified, either because they are recently described, or simply

because there is no data to enable their assessment.
Line 70-72: There are a few definitions of cryptic species. Perhaps expand on this to avoid confusion with sibling species, sister species etc.
Line 72-73: Supporting reference required for this statement.
Line 74: I am assuming this is because we need species-specific data for applied conservation? If this is what you mean, you should state it here.
Line 76: You state “several examples” but provide only one in this paragraph. I advise adding at least a couple more examples to reinforce the point you are making.
Line 83: Can specify the most diverse order here too (rodents?).
Line 85-87: Can also state their volant trait – flying animals are more difficult to observe and study.
Line 87-89: Why is there a sudden taxonomic expansion, and why weren’t these species detected until now?
Line 92-97: This sentence is excessively long. I suggest breaking it up into two or three shorter sentences for readability.
Line 97: No need for “pressing”.
Line 97-102: What did you predict to find prior to carrying out the study? Tested hypotheses and predictions are not clear.
Line 99: the meaning of “human pressure” is unclear.

Methods

Line 386-387: It is unclear whether “panamerican” is also referring to North America.
Line 404: I can imagine there is some ambiguity here as there are a lot of unresolved taxonomic groups of bats within the Neotropics, and throughout the World more generally.
Line 406-408: Perhaps add a sentence to state how these quality control measures impacted sample size and whether it left any gaps within the dataset.
Figure 7: The caption should contain greater. At least provide a brief study on the different stages of the pipeline that are visualised within the figure itself.
Line 447: What exactly is meant by the “stationary” here? This is not clear to the non-specialist.
Line 476: To those unfamiliar with the Morrone et al. paper, it will not be clear which environmental variables are captured in this analysis. I advise adding a couple of sentences to provide more detail on this.
Line 485-494: I agree that this is a unique and interesting method to quantify hidden species diversity over a spatial scale, but it is currently unclear to me how this controls for sampling bias. I may have missed something, but I am not seeing this information in the text.
Line 498: There is a missing closing parenthesis for “main roads”.
Line 519: I like the approach and how it compares accessibility across a large spatial scale. There could of course be instances where accessible areas are not sampled due to other logistical issues or constraints (finances, hazards etc.), and this is something that may be worth discussing.
Line 551: Anthropogenic threats are restricted only to habitat-related threats? E.g., direct killings, artificial light at night, etc are not quantified, correctly? If so, the restriction to habitat-only threats should be more explicit.
Line 561: Try to provide as a comprehensive list as possible.
Line 568: Provide some further details on how this spatial thinning was conducted (which packages were used, how many runs etc.).
Line 572: The choice to use 2.5 minute resolution layers is odd, as there is finer-resolution data available.
Line 573: Were these the only GCMs available for the neotropics? It is always good practice to make an ensemble forecast using all available models.
Line 581-587: You can list the retained post-correlation test variables that were used to construct the ENMs.
Line 588-589: To me, this sentence makes it sound like only the MaxEnt algorithm was used. Whilst MaxEnt is a generally reliable model, an ensemble approach using multiple algorithms (GAM, GLM, RF, etc.) would be superior, and would result in an averaged model. I strongly urge the authors to consider this approach.
Line 597: Which specific threshold values? There are a number to choose from.
Line 621-623: Perhaps provide some information on the percentage of species that currently have such a classification.
Line 638: Low scores for “Not Evaluated” species is misleading. These species may yet to receive assessments because they are recently described, but they may be highly threatened nonetheless.

Results

Line 114 and 116: The genus level information conflicts between these two lines.
Figure 1: Very nice figure. This is more of a suggestion thana comment, but it would be nice to colour code the sampling records (i.e., the points on the map) to each family.
Figure 2: The image of the bat and its silhouette is not necessary. The numerical information for described and undescribed species is already provided.
Line 160-165: This information is detailed in the Methods section so no need to repeat it here.
Figure 3: It is interesting that HSR is quantified for each biogeographic region, whilst the other three metrics produced continuous layers across the whole of the neotropics. Is this not achievable for HSR? This would greatly inform conservation efforts.
Line 205: Check phrasing on this line.
Table 1: This table is interesting but is extensive, and may therefore be better suited to the supplementary information.
Line 240: I would not call a geographic range size of 662,476 km² “very restricted”! It may be comparatively smaller than other species in this study, but it is a very large area and would not meet the criteria for a threatened IUCN classification. Should this comma be a decimal place?

Discussion

Line 263: “disciplinary”
Line 265: The phrase “harbor undescribed taxa” is strange to use when referring to already described species. You can just state that these species complexes may contain new taxa.
Line 278: What exactly is meant by “environmentally complex”?
Line 285-286: Extend this sentence to clearly explain why conservation planning at these sites is not currently possible. This would not be obvious to readers not working in/around these zones.
Line 305: Here you can specify which specific climate change scenarios. Your methodology looked at two separate scenarios, but here it is not clear whether you are referring to both, or a single one of these.
Line 309-312: This is very true for bats in temperate regions, but I think torpor is less of an issue in tropical zones, such as your study area, where bats may be active year-round.
Line 316-319: Certainly, disturbed and degraded habitats provide new opportunities for some species. You can expand on this here to state why.
Line 328-330: I wouldn’t necessarily agree with this. While there is certainly a decline in the number of taxonomic experts, this is not necessarily the case for the bat evolution field. Further, integrative taxonomy is becoming evermore common, and so accurate and reliable species descriptions can be made by multidisciplinary teams of geneticists, ecologists, morphologists, and bioacousticians.
Line 333-335: Perhaps provide an example or two of such conservation approaches that cover all species.
Line 362-363: See my previous comments concerning the ENMs. An ensemble approach would improve this robustness.
Line 371: Neotropical bats are presented as one of the most studied groups of mammals, but where is the evidence for this? Do you have a reference to support your statement?
Line 369-376: Currently I feel as though this discussion is missing a paragraph linking the findings to the broader picture. The points discussed are applicable to bats in other areas of the world, and to cryptic species more generally, and linking this study to the global-scale conservation situation will greatly enhance the scope of the manuscript.

(Remarks on code availability)

Reviewer #2

(Remarks to the Author)
I co-reviewed this manuscript with one of the reviewers who provided the listed reports. This is part of the Nature Communications initiative to facilitate training in peer review and to provide appropriate recognition for Early Career Researchers who co-review manuscripts.

(Remarks on code availability)

Reviewer #3

(Remarks to the Author)
This is a well-written and well thought out study, which presents a few novel indices for identifying priority areas for taxonomic investigations e.g. the Hidden Species Richness (HSR) index. The manuscript is also important (perhaps critical) in drawing attention to the taxonomy crisis that is affecting not just Neotropical bats, but possibly most vertebrate groups (and no doubt, all invertebrate groups and plants too). Most of my comments on this ms are broad, and presented below in no apparent order of importance.
1) Visually, the HSR index (Fig 3A) looks to be very strongly correlated with the locations of the genetic samples (Fig 1), but no mention is made of this. Despite the weightings of the HSR, I get the feeling that it simply represents where past work has been done (rather than show where hidden species richness occurs). I think that the authors need to convince the reader that: 1) there is no correlation between the two figures mentioned above, and 2) if there is a correlation, explain why we should believe in the HSR (an easy explanation could be that taxonomists have preferentially sampled species rich areas in the past, avoiding areas with low diversity - it is hard to justify funding an expedition to a species poor location!).
2) I couldn't help noticing that the future distributions of the species listed in Table 1 (supplementary figs S53 to S101) almost invariably show reduced future distributions. Are there no "winners" in this group? Species that would benefit from climate change? I find this hard to believe. At the very least, this requires some comment in the Discussion, with comparisons to other relevant studies from elsewhere in the world.
3) Considering the critical state of the taxonomy of Neotropical bats (shown by this study), I am surprised that they have not made any effort to compare this with other tropical hotspots for bats (Africa and South-East Asia). I think a paragraph in the Discussion, showing parallels with other hotspots, would strengthen their argument here (by showing that this is a global problem, and not just a South/Central American problem).
4) I am not familiar with the geographic definition of "Neotropics" (and I'm certain that most readers of this manuscript will not be either). I am particularly interested in the total lack of records from the central and southern parts of Argentina (interestingly, there are two records from Chile, within this zone). Is this because this region lies outside of the Neotropics? An easy solution to this is to draw an east-west line on the map in Fig 1C to show the southern limits of the area of interest.
5) I like the fact that they have created a list of priority species (Table 1), which includes input from various sources e.g. human modification, climate change, red list, etc. Is there nothing to compare this table to? For example, is there no study that has tried to prioritise bat species for conservation for this region? Or perhaps individual countries (e.g. Brasil). In this

case, drawing on these studies to support their claims for their priority listing, would be useful?
6) The authors end the manuscript with a plea "The race against extinction...... never been more important". I fully support this claim, but the problem is that nobody seems to be listening. Could they not include a statement for the support of more taxonomists (or at least for more taxonomic studies)? Perhaps if taxonomists could be enticed to start working on the tabulated list, starting with the highest priority species, it would make for more meaningful progress.
7) I have added their manuscript with a few very minor comments/corrections.

(Remarks on code availability)

Reviewer #4

(Remarks to the Author)
This article examines Neotropical bat diversity from the angle of conservation of “hidden” species diversity. The task at hand is ambitious and the manuscript includes some interesting bits. Overall, the approach presented is similar to what Carstens et al. have developed as a platform called PhyloGatR, except that genetic and spatial data is unmatched. The authors use a combination of single locus species delimitation methods, climate-based niche model projections, and a novel index developed to identify/prioritize hidden species. The manuscript is generally well written with very few typos; although at times the authors pose some statements that are speculative. Below I summarize some of my criticisms.
Major concerns
Species delimitation: It is typically straightforward to use single locus species delimitation software on barcode sequences. However, the authors failed to thoroughly vet the assumptions of each species delimitation model in light of their sampling. This is a major drawback with the single locus (COI barcode) species delimitation approach used here. The limited and biased sampling that exists in COI sequences for most of the species mined artificially boosts up genetic structure, which is a violation of one of the main assumptions of each of the delimitation models used. This is especially the case for the top two species in Table 1, which are considered here as the highest priority for hidden species complex, yet they are represented by very few samples and a very biased geographic distribution of sequences. Interestingly, the authors suggest that these two top species each could be a complex of 2 species. It is unclear why these then rank higher than others that may have a higher estimate of hidden diversity and are in geographic regions that are equally imperiled. Finally, this analysis lacks a thorough quality control step. It is assumed that sequences deposited in these databases are appropriately curated and the taxonomy is void of errors, which is not the case in these databases.
Understandably, the authors are limited to the sampling available in public databases. However, single-locus species delimitation here is confounded with taxonomic designation. Unreliable delimitation results could artificially lead to incorrect prioritization for conservation in the context of this paper. Furthermore,
1. it is interesting that the authors chose the bPTP model, which assumes constant partition rates, over the more recent mPTP version that relaxes this assumption -- this is not justified in the paper.
2. bPTP assumes that branching events are independent from one another. However, this assumption is violated given the sparse sampling of the genetic data.
3. ABGD strongly relies on large datasets to make delimitation inferences. For many of the datasets presented herein, potential or "hidden" species are represented by as few as 1 sample, as is the case of the top two bats in Table 1, which would produce unreliable estimates of barcode gaps.
4. ASAP uses pairwise genetic distances and does not rely on phylogenetic reconstruction. Uneven and spatially biased sampling poses a problem here by artificially increasing distances.

Mapping hidden species: The method developed here poses an interesting way to examine potential hidden diversity. While the authors clearly describe their HSR index, sampling still becomes an issue. For many bats examined here (including those species that are considered as "high priority") there are only one or a handful of sequences used as evidence for "hidden diversity". This clearly assumes that these bats are evenly distributed across provinces, which is not true. It is unclear how this assumption is treated in the geographic designation. This is important especially because sampling is uneven. For example, as described in the manuscript, habitats across the Neotropics are quite heterogeneous. Thus, a sampled bat in Guyana, where a disproportionately large sample of the COI sequences come from, is not indication that the species is evenly distributed through the Dry Diagonal or the Atlantic Forest, regardless of geographic proximity.

Niche modeling: The niche modeling section is quite standard for what studies focused on spatio-temporal range changes typically do. There are several major issues with this as implemented in the manuscript to reach the overall goal: 1. there is no connection between the niche modeling data and the sequence data. Many species have only a few COI sequences available, yet the niche models are built with the entire observation dataset for each species, which goes against what the authors are trying to do with their estimation of hidden diversity. 2. the niche models were built for each species independently. This assumes that the species are immutable and not responding to any biotic interaction with sympatric/syntopic members of the bat community. 3. each species niche model is independent and based on different factors selected by the Maxent algorithm, so a change in one species cannot be compared to a change in another species because the models were essentially built with a different set of variables. It is unclear how this affects the overall goal of identifying species conservation priorities. 4. the issue of non-transferrability of the models into non-analog future environments is a big problem here because there is a strong reliance on the results of the niche models to estimate the "conservation priority" ranking.

Some specific line points:
L119: this argument is unsupported and misleading as sequencing is 1. uneven across ecoregions and 2. biased towards easily captured bats that are common across Neotropical communities. The assumption that these species are "dominant

across Neotropical ecosystems" omits that many bat species are not as easily captured with the typical sampling methods.
Figure1: Panel B in Figure 1 is clearly missing an axis label. Maybe a little bit too picky, but it is strange that the bat used in the center of panel D in Figure 1 clearly does not represent any bat species found in the Neotropics. Why not use the same as in Figure 2?
L146: What is the definition of an evolutionary unit used in this manuscript? Given the biased sampling of COI sequences, I would question the estimates presented in Figure 2.
L171: This result is greatly dependent on biased COI sampling.
Figure3: Panel A in Figure 3 exemplifies the issue with the genetic data mining and biased sampling. Note how the "HSR" hotspots are centered only where a high number of available COI sequences are present.
L228: This sentence makes no sense.
L262: This genetic dataset is arguably extensive given that the focus is unevenly sampled COI barcode sequences.
L263: “multi-disciplinary”
L266: This is a wild speculation not based on how evolving lineages work.
L285: This is unsubstantiated.
L578: The model calibration here uses a 200 km buffer around the species observation areas. This seems to have been applied arbitrarily to all species modeled without considering the biology of the species or the potential for overfitting the models given the disparity between presences and background localities used to model.

(Remarks on code availability)
While I reviewed the code cited, I did not have time to do a full test to examine if the analyses are replicable because it entailed data downloads from multiple sources. As written, it is not possible to run the code on a subset of the species without spending additional time modifying it.

Version 1:

Reviewer comments:

Reviewer #1

(Remarks to the Author)
Dear editor,

I am writing to confirm that both my co-reviewer and I are satisfied that the authors have adequately addressed all of our comments. We feel that the revisions have substantially improved the manuscript and that the authors’ responses were thorough and appropriate.

Thank you for the opportunity to review this work.

Best regards,

Ricardo Rocha & Daniel Hending

(Remarks on code availability)

Reviewer #2

(Remarks to the Author)
I co-reviewed this manuscript with one of the reviewers who provided the listed reports. This is part of the Nature Communications initiative to facilitate training in peer review and to provide appropriate recognition for Early Career Researchers who co-review manuscripts.

(Remarks on code availability)

Reviewer #3

(Remarks to the Author)
I am happy with the way the authors have dealt with my comments and concerns

(Remarks on code availability)

Reviewer #5

(Remarks to the Author)
General Comments
This study is highly original and presents results that are significantly relevant to the research and conservation of

Neotropical bats. Innovatively, the work identifies priority species and regions for future phylogenetic and taxonomic investigations.
In this review, I have focused my comments on the species delimitation aspects of the manuscript. As my expertise in Ecological Niche Modeling (ENM) is limited, and I only joined this second round of review, I could not evaluate the entire paper in detail.
Overall, the authors have conducted excellent work in compiling, filtering, and analyzing an extensive COI database. It is well-established that mitochondrial markers can provide critical evidence regarding cryptic diversity, often preempting patterns later confirmed by more complex multilocus analyses.
However, I find that some conclusions drawn from the results lack sufficient caution — specifically, the emphatic assertion regarding the existence of 101 potential undescribed species across 52 complexes. The tone of this conclusion should be more conservative. The discussion should more explicitly state that these findings are based on single-locus species delimitation approaches with heterogeneous geographic coverage, while also highlighting potential gaps in the data.
Finally, I suggest increasing the transparency of certain results within the supplementary material, thereby allowing readers — particularly taxonomists — to draw their own informed conclusions.

Specific Comments on Species Delimitation
For several taxa, such as Myotis, Neoplatymops, and Nyctinomops, the phylogenetic pattern is very clear: species are represented by a large number of COI sequences forming paraphyletic assemblages, suggesting that deep taxonomic investigation is needed. However, for many other species, I wonder if the phylogenetic patterns shown in the supplementary data might: 1) represent phylogeographic structuring, as two sister-clades with no reported genetic divergence are presented; 2) be explained by stochastic processes in the mtDNA (e.g., heteroplasmy, introgression, gene tree discordance) or curation errors related to a single individual used in the analysis.
Regarding this matter, the case of Histiotus is a pertinent example. In Table S1, I noted that 3 out of the 7 analyzed species of Histiotus were identified as complexes. Nevertheless, the delimitation methods do not point to a majority consensus, as argued by the authors in the rebuttal letter (page 22). While I understand that the authors adopted the most frequent result, I do not agree that this was a conservative choice. For example, the two lineages found in H. humboldti (page 42) are only 2% divergent. Moreover, for all three species, hidden lineages are inferred based on a single specimen, which violates the assumptions of the delimitation models used (as noted by Reviewer #4). This also appears to occur for Platyrrhinus brachycephalus, for which the divergence among the five COI sequences displayed in the tree is nearly 3% (a value potentially representing intraspecific variation), as well as for some Caribbean species, as also noticed by this same reviewer.
Therefore, although the authors show, on average, good sample representation at the genus level for the delimitation inferences, I believe these specific cases might have inflated their results and should be examined more carefully. I have highlighted some examples of lack of congruence in the species delimitation table that deserve further explanation or support. While many of them might have additional studies as evidence of hidden diversity, I found a lack of proper discussion in the text regarding these cases.
Lastly, I believe the results would be much clearer if the authors provided the mean divergence among COI lineages for each taxon in the supplementary material. I suggest including this information in the phylogeny/map figures. There is no need for two maps to show the geographic distribution of molecular clades and overall species sampling in Figures S1 to S52; instead, I suggest the authors use this space to show the divergence estimates among clades, displayed either as a table or within the phylogenetic scheme. Also, the authors should draw attention to taxa with low genetic sampling, since downstream analyses using fewer than three sequences per COI lineage preclude geographic inferences and might bias the interpretation of results (perhaps by including a remark in Table S1, as they did for *insufficient data).
I have additional observations regarding the supplementary data.
Figs. S1 to S52:
- Node Symbols: the circles representing node sizes/posterior probabilities are not clear; they should be replaced with more distinct symbols.
- Contrast: the light blue circles are difficult to see against the map background; I suggest a color change.
- Taxonomic Context: in some trees, neighboring taxa were omitted (e.g., Phyllostomus hastatus, Platyrrhinus brachycephalus, Choeroniscus, Anoura). I suggest presenting the complete phylogenetic arrangement to assist taxonomists.
- Page 18: The symbol size is inconsistent (smaller) compared to other maps.
- Page 35: The tree for P. brachycephalus includes a sequence for P. helleri. Please verify if this is a curation error.
- Pages 36, 37: The phylogenetic trees for the two Sturnira species are missing. Additionally, the maps shown appear to be duplicates of the Platyrrhinus brachycephalus maps.
- Page 43: Lineage colors are inverted on the map.
I also have included a few comments in the rebuttal letter, main text and supplementary material documents.

(Remarks on code availability)

Version 2:

Reviewer comments:

Reviewer #5

(Remarks to the Author)
I am satisfied with the latest version of the manuscript. All comments have been promptly addressed or corrected in the text. The authors also did an excellent job in revising the supplementary material. The data presented in Figures S1 to S45 are

now much more transparent and informative, and they will be highly useful for readers interested in exploring the species-specific phylogenies in greater detail. I have only a few minor comments on the supplementary data, which are included in the pdf file.
Regards,
Ana Pavan

(Remarks on code availability)

Open Access This Peer Review File is licensed under a Creative Commons Attribution 4.0 International License, which permits use, sharing, adaptation, distribution and reproduction in any medium or format, as long as you give appropriate credit to the original author(s) and the source, provide a link to the Creative Commons license, and indicate if changes were made.
In cases where reviewers are anonymous, credit should be given to 'Anonymous Referee' and the source.
The images or other third party material in this Peer Review File are included in the article's Creative Commons license, unless indicated otherwise in a credit line to the material. If material is not included in the article's Creative Commons license and your intended use is not permitted by statutory regulation or exceeds the permitted use, you will need to obtain permission directly from the copyright holder.
To view a copy of this license, visit https://creativecommons.org/licenses/by/4.0/

REVIEWER COMMENTS

Reviewer #1

1. **The title does not currently reflect the breadth of the study, and it would benefit from including the geographic area.**
   Answer: We are thankful for the suggestion. We edited the title adding the geographic focus of the study.

2. **The introduction requires more depth: a greater exploration of cryptic species complexes is required, and further examples of the implications of data deficiency for conservation should be added. Additionally, the manuscript of benefit of Neotropical bats being presented as a case study for the study of cryptic diversity, and their threats. Currently, they are mentioned as having high levels of uncovered hidden diversity and in need of a comprehensive revision but are not put across as a suitable case study for the assessment of how cryptic diversity potentially impacts conservation in a more holistic manner.**
   Answer: We appreciate the reviewer for these recommendations that have greatly enhanced our introduction. We have now edited our text to provide further information on cryptic species complexes, including examples demonstrating the conservation implications of data insufficiency, and highlighting Neotropical bats as an ideal case study to explore the role of hidden diversity with respect to conservation. Importantly, our revisions (along with more minor edits recommended throughout the introduction) strengthen our narrative and situate our study more broadly within the context of the worldwide hidden diversity challenge.

3. **ENMs could be strengthened by using an ensemble approach with multiple algorithms - currently only the MaxEnt algorithm is used. There are areas of the methods where greater detail and justification is needed, and the ENM approach can be refined by adding more algorithms and using an ensemble approach.**
   Answer: We appreciate the reviewer's suggestion regarding ensemble modeling. While ensemble methods are valuable and widely used in Ecological Niche Modelling (ENM), we made a deliberate decision to use MaxEnt after weighing the tradeoffs in face of the specific characteristics of our study design, including its continental scale, the use of presence-only data, and our species complexes. Valavi et al. (2022, *Ecol. Monogr*.) provide one of the most extensive comparison of ENM methods evaluating 21 modeling approaches across 225 species. Their findings were highly relevant to our decision. The authors show that a meticulously computer-demanding constructed ensemble of finely tuned individual models performed only marginally better than well-tuned MaxEnt models.

   Below, we discuss five key aspects that support the use of MaxEnt in our study:

   First, its robustness with presence-only data. Its maximum entropy framework is inherently designed for presence-only data, while many algorithms that are used in ensemble methods (e.g., GLM, GAM, BRT) have been adapted from techniques that use true absences or

pseudo-absences, which to us, can adds layers of assumptions and potential biases [Elith et al. (2011) *Divers. Distrib*.; Renner & Warton (2013) *Biometrics*].

Second, MaxEnt has a top-tier predictive performance. In several studies MaxEnt produced distribution maps of comparable accuracy to ensembles, highlighting that single-algorithm approaches can suffice without added complexity [Kaky et al. (2020) *Ecol. Inform*.]. For example, in Valavi et al. 's (2022) comprehensive study, MaxEnt ranked among the top-performing models, showed high consistency across species and regions, and performed comparably to other top methods. The authors conclude that MaxEnt "*is still among the top performing models*" and praise its overall reliability. Recent reviews echo this, noting MaxEnt's dominance in 85% of ENM studies due to its adaptability and precision (Vasconcelos et al. (2024) *earth*).

Third, there are potential complications introduced by ensembles that can reduce prediction quality. Combining algorithms with diverse mathematical foundations and data requirements can sometimes add unnecessary complexity, and dilute strong signals from high-performing models with low-performing ones, leading to biologically less accurate predictions on average compared to a single robust method like MaxEnt [Valavi et al. (2022) *Ecol. Monogr*.]. This is particularly relevant when working with numerous species with distinct ecological preferences and uneven occurrence data available, as in our study.

Fourth, ensuring consistency and comparability across species complexes. Using a single, theoretically grounded algorithm like MaxEnt promotes methodological coherence, enabling reliable comparisons of climate vulnerability patterns among our 52 complexes. Due to the taxonomic implications, having a single model for all complexes is preferable to an ensemble where complexes may have algorithms that perform better for some complexes than for others; this would limit the comparability across groups.

Fifth, practical feasibility for a continental-scale analysis. Building an effective ensemble requires individual tuning of each component model, which [Valavi et al. (2022) *Ecol. Monogr*.] showed could multiply computational demands by 5-10 times for optimal results, demands that escalate further with cross-validation across 52 complexes. Ensemble approach ranks among the most computationally expensive options, often with only marginal gains in accuracy in relation to MaxEnt.

For all these reasons, we believe MaxEnt aligns with best practices for our research goals. We have added a new paragraph in the Methods to justify the use of MaxEnt:

> "We selected MaxEnt given its robust performance with presence-only data and its proven reliability across diverse taxa and regions. A single well-tuned algorithm ensures consistent and comparable predictions across our 52 species complexes, whereas other methods can introduce variability that complicates cross-taxa comparisons at continental scales".

4. **The discussion would greatly benefit from a broad concluding paragraph linking the findings of this bat-specific neotropics study to global scales and cryptic species complexes more generally.**
   Answer: We thank the reviewer for this suggestion. We have added a final paragraph in the Discussion linking our findings for Neotropical bat diversity to the broader global challenge of hidden biodiversity. The additional paragraph illustrates that there is hidden diversity globally across every taxonomic group and region, demonstrates the transferability of our proposed methodological framework (specifically HSR index and threat layer component) to other systems, and discusses the global conservation implications. This addition enhances the scope of the manuscript by situating our bat-focused research within the broad topic of biodiversity conservation, thereby making our findings and approaches applicable to researchers from all taxonomic groups and in different geographic regions.

5. **Specific Comments**

   Title
   The title should reflect that the study was carried out for the Neotropics. I assumed it was a global study until I reached line 92 of the manuscript.
   Answer: We have revised the title to clarify the geographical focus of our study.

   Abstract
   Line 28: Consider "the exceptional bat diversity of the Neotropical" instead of "the Neotropics' exceptional bat diversity"
   Answer: Modified as suggested.

   Introduction
   Line 52-53: Sentence is awkwardly phrased. The term "accelerated anthropogenic change" should be rephrased so that it lists biodiversity threats, of which one is anthropogenic disturbance.
   Answer: We have revised the sentence and added specific biodiversity threats to improve clarity. The section now reads as follows: "Scientists race against time to discover and describe new species as global biodiversity faces <u>unprecedented threats, including habitat destruction, climate change, invasive species, and anthropogenic disturbance</u>"

   Line 53-56: This is primarily due to unresolved cryptic species complexes, i.e., hidden biodiversity that can not be detected by traditional means. This warrants discussion here.
   Answer: We acknowledge the reviewer's point about discussing cryptic species complexes earlier. Since we define cryptic species in the second paragraph, we have made a small but important addition to better connect these concepts in the first paragraph.

   > "Although taxonomists describe hundreds of new species every year, the true extent of global biodiversity and the number of undiscovered species remain elusive (1,2). Conservative estimates suggest our planet harbors between 5 to 9 million species (3,4),

yet scientists have formally named only about 1.9 million (5,6), with much of this discrepancy attributed to cryptic and unrecognized diversity".

Line 58: Better rephrased to state that individual species are the primary currency of biodiversity (i.e., species richness).
Answer: We edited the text accordingly. "This knowledge gap, known as the Linnean shortfall (7,8), is particularly concerning, because individual species are the primary currency of biodiversity research, as well as the foundation in ecology, evolution, and conservation (9)".

Line 62: Is the sixth "mass extinction" really ongoing?
Answer: The scientific community continues to debate whether current extinction rates constitute a true "mass extinction". While some studies suggest we are in the early stages of a sixth mass extinction based on elevated extinction rates [Barnosky et al. (2011) *Nature*; Ceballos et al. (2015) *Sci. Adv*.; Ceballos et al. (2017) *Proc. Natl. Acad. Sci. USA*; Cowie et al. (2017) *Nautilus*; Cowie et al. (2022) *Biol. Rev*.; Ceballos & Ehrlich (2023) *Proc. Natl. Acad. Sci. USA*], others argue that current rates, though concerning, have not yet reached the magnitude of past mass extinctions [Rull (2022) *EMBO*; Wiens & Saban (2025) *Trends Ecol. Evol*.]. We have revised our sentences to reflect the ongoing scientific debate surrounding this.

We made these changes: "Current extinction rates, while debated in scale, are exceeding natural levels by several orders of magnitude (14,15 but see 16), underscoring the urgency of addressing the Linnean shortfall"

Line 66-70: There are also countless species that are not yet classified, either because they are recently described, or simply because there is no data to enable their assessment.
Answer: Indeed, we edited the sentence to address this issue.

Line 70-72: There are a few definitions of cryptic species. Perhaps expand on this to avoid confusion with sibling species, sister species etc.
Answer: We appreciate this suggestion to clarify the definition and have expanded the text to distinguish cryptic species from related concepts: "Among these species are the so-called "cryptic species", distinct evolutionary lineages that have diverged genetically but remain morphologically indistinguishable (17)".

Line 72-73: Supporting reference required for this statement.
Answer: We have added an appropriate reference as suggested.

Line 74: I am assuming this is because we need species-specific data for applied conservation? If this is what you mean, you should state it here.
Answer: We have clarified this statement to be more explicit about why hidden diversity falls outside conservation frameworks. The section now reads as follows:

> "This diversity often lies outside conservation priorities, because unrecognized species are not included in conservation planning or policy frameworks, exacerbating the Linnean shortfall and leaving many species at risk of extinction before they are even documented (19)".

Line 76: You state "several examples" but provide only one in this paragraph. I advise adding at least a couple more examples to reinforce the point you are making.
Answer: We followed this, but added only one more example due to the space constraint, focused on our taxonomic group of interest.

Line 83: Can specify the most diverse order here too (rodents?).
Answer: We revised the text accordingly: "They comprise the second most diverse mammalian order after rodents, with…"

Line 85-87: Can also state their volant trait – flying animals are more difficult to observe and study.
Answer: We agree this addition would strengthen the explanation of why bats are challenging to study. The text now reads as follows: "Nevertheless, due to their nocturnal habits, elusive behaviors, the ability to fly, and the challenges of assessing them in the wild…"

Line 87-89: Why is there a sudden taxonomic expansion, and why weren't these species detected until now?
Answer: The reviewer raises an important point about explaining the drivers behind this taxonomic expansion. We have added context to explain this:

> "This group has seen a significant increase in new species descriptions in recent years, driven primarily by advances in molecular techniques that can readily detect cryptic diversity despite morphological similarity. Over the past two decades, taxonomists have described over 300 bat species (24), many of which were previously hidden within morphologically similar complexes".

Line 92-97: This sentence is excessively long. I suggest breaking it up into two or three shorter sentences for readability.
Answer: We followed the reviewer' suggestion and simplified this sentence.

Line 97: No need for "pressing".
Answer: Indeed. Removed as suggested.

Line 97-102: What did you predict to find prior to carrying out the study? Tested hypotheses and predictions are not clear.
Answer: We acknowledge that explicit hypotheses were missing and have added our predictions, as follows:

"Here, we aim to investigate the extent and magnitude of bat hidden diversity in the Neotropics, one of Earth's most biodiverse yet threatened regions. In particular, we address four key questions: (1) How many hidden bat species remain undiscovered in the Neotropics? (2) Where are they? (3) Which areas, considering human-induced landscape modification and climate change, are critical for prioritizing time, funding, research, and conservation efforts focused on describing and protecting hidden species? and (4) How many hidden species could lose significant areas of suitable habitat in the next decades due to climate change? We expect that: (1) substantial hidden diversity exists in Neotropical bats, particularly in morphologically conservative groups; (2) hidden diversity is concentrated in climatically and topographically complex regions with high habitat heterogeneity; and (3) unrecognized species face disproportionate extinction risk due to climate change and uneven human-induced landscape modification".

Line 99: the meaning of "human pressure" is unclear.
Answer: We have changed "human pressure" for "human-induced landscape modification".

Methods
Line 386-387: It is unclear whether "panamerican" is also referring to North America.
Answer: We edited the sentence to make it clearer.

"For bat genera with a Panamerican continental distribution extending beyond the Neotropics, we only considered species whose distribution covers at least…"

Line 404: I can imagine there is some ambiguity here as there are a lot of unresolved taxonomic groups of bats within the Neotropics, and throughout the World more generally.
Answer: Taxonomic ambiguity does exist within Neotropical bats, which is why we used a conservative multi-algorithm approach requiring agreement among five independent methods (ABGD, ASAP, GMYC, bPTP, and mPTP) before designating lineages as hidden species. We also updated nomenclature following current databases (Mammal Diversity Database and batnames) to ensure the taxonomic consistency with the most up-to-date information.

Line 406-408: Perhaps add a sentence to state how these quality control measures impacted sample size and whether it left any gaps within the dataset.
Answer: We agree this information would be valuable and have added details about the impact of our quality control procedures. We added the following information:

"These quality control filters resulted in the exclusion of approximately 12% of initially retrieved sequences, primarily due to length requirements and the removal of sequences with stop codons or indels. While this filtering reduced our overall dataset size, it ensured high-quality phylogenetic analyses and did not create substantial geographic gaps".

Figure 7: The caption should contain greater. At least provide a brief study on the different stages of the pipeline that are visualised within the figure itself.
Answer: We offer more detail and have expanded it to describe the pipeline stages. We added the following information:

> "Figure 7. Pipeline used to obtain and clean genetic data. The workflow includes: (1) Data acquisition from GenBank and BOLD using iterative searches; (2) Data processing including data completeness, georeferencing, and taxonomic updates; (3) Quality control filtering to remove artifacts; and (4) Mapping and data preparation for phylogenetic analyses".

Line 447: What exactly is meant by the "stationary" here? This is not clear to the non-specialist.
Answer: We have revised the sentence to make it clearer. We changed and added:

> "We used Tracer v.1.7 (68) to assess the statistical convergence of MCMC chains (stationary) by ensuring that the Average Standard Deviation of Split Frequencies (ASDSF) was < 0.01, the Estimated Sample Size (ESS) was > 200, and the Potential Scale Reduction Factor (PSRF) approached 1"

Line 476: To those unfamiliar with the Morrone et al. paper, it will not be clear which environmental variables are captured in this analysis. I advise adding a couple of sentences to provide more detail on this.
Answer: Indeed. We changed our paragraph and included more details as follows:

> "We plotted and analyzed them using the biogeographic provinces according to Morrone et al. (58) for the Neotropical realm. These provinces are defined based on biotic and abiotic factors including climate, topography, geological history, and endemic taxa distributions, incorporating distinct ecological and evolutionary processes that define species distributions. The provinces are widely recognized for their ability to capture the complexity of biogeographic patterns, making them particularly suitable for our study."

Line 485-494: I agree that this is a unique and interesting method to quantify hidden species diversity over a spatial scale, but it is currently unclear to me how this controls for sampling bias. I may have missed something, but I am not seeing this information in the text.
Answer: Our HSR index specifically addresses sampling bias through three mechanisms built into the formula. First, we apply normalization based on the logarithm of the total sequence number (log (N_sequences + 1)) to reduce the sampling effort bias across regions. Second, we incorporate accessibility Z-scores that weight provinces with lower accessibility (farther from roads, cities, and protected areas) higher, as these areas are typically undersampled. Third, we include province size Z-scores to account for the fact that larger provinces may appear more diverse simply due to area effects.

We improved text clarity in the manuscript, which now reads as follows: "We consider that this metric enables a more equitable comparison of hidden species richness across regions by controlling for three major sources of sampling bias: uneven sampling effort (through logarithmic normalization), geographic accessibility (through accessibility weighting), and province size effects (through area normalization)."

Additional information about HSR is also described in Supplementary Data. Also, in the GitHub repository with additional details and a tutorial for readers and potential users at https://github.com/diegodaem/HSR-Index

Line 498: There is a missing closing parenthesis for "main roads".
Answer: Thanks for noticing this, we've fixed it.

Line 519: I like the approach and how it compares accessibility across a large spatial scale. There could of course be instances where accessible areas are not sampled due to other logistical issues or constraints (finances, hazards etc.), and this is something that may be worth discussing.
Answer: The reviewer raises an important point about additional sampling constraints beyond geographic accessibility. We agree this limitation should be discussed and have added this consideration to our Discussion section where we address the limitations of our HSR approach. We added the following:

> "While our accessibility index captures geographic constraints to sampling (distance to roads, cities, and protected areas), we acknowledge that other factors such as political instability, funding limitations, or safety concerns may prevent sampling in otherwise accessible areas. However, our approach provides a standardized, large-scale approximation of sampling likelihood that can be consistently applied across the entire Neotropical realm".

Line 551: Anthropogenic threats are restricted only to habitat-related threats? E.g., direct killings, artificial light at night, etc are not quantified, correctly? If so, the restriction to habitat-only threats should be more explicit.
Answer: We have clarified this scope in the methods section to specify that our dataset captures habitat-related pressures:

> "This dataset primarily captures landscape-scale habitat modifications integrating multiple anthropogenic pressures, as urban development, agricultural expansion, livestock production, resource extraction, transportation infrastructure, and other human activities…"

Line 561: Try to provide as a comprehensive list as possible.
Answer: We have expanded this to provide a more comprehensive list of occurrence databases consulted. The text now reads as follows:

> "We collected occurrence data representing the total distribution of each hidden species complex from databases such as Arctos, GBIF (Global Biodiversity Information Facility), SpeciesLink (Brazil), SIB Colombia, VertNet, iNaturalist, and different museum collections including the Field Museum of Natural History (Chicago, USA), American Museum of Natural History (New York, USA), and National Museum of Natural History (Washington D.C., USA)".

Line 568: Provide some further details on how this spatial thinning was conducted (which packages were used, how many runs etc.).
Answer: We have added additional methodological details. Some of these were not included in the previous version of the ms due to word limit constraints. We have now added the following:

> "…and applied spatial thinning using a minimum distance of 5 km to reduce autocorrelation and minimize overrepresentation of well-sampled areas. Spatial thinning was conducted using the spThin package in R with 100 iterations."

Line 572: The choice to use 2.5 minute resolution layers is odd, as there is finer-resolution data available.
Answer: We acknowledge that higher-resolution data are available. However, we deliberately selected a 2.5-minute (4.6 km) resolution considering several important factors. First, ecological relevance: a ~ 5 km resolution can adequately account for the relevant environmental heterogeneity for bats given their ecology, as bats are flying mammals and typically have home ranges and dispersal movements that operate on that spatial scale. Second, data reliability: finer resolution climate projections will often have greater interpolation uncertainty, which is important in data-scarce areas like the Amazon and the Andes. Finally, methodological consistency: because we perform a spatial autocorrelation that corresponds in the ecological niche process to 5km.

Line 573: Were these the only GCMs available for the neotropics? It is always good practice to make an ensemble forecast using all available models.
Answer: The reviewer is correct. There are several GCMs available for the Neotropics. But many of the GCMs share similar backgrounds and produce redundant projections. We used the *chooseGCM* R package (Esser et al. 2025. chooseGCM: a Toolkit to Select General Circulation Models in R. *Glob. Chang Biol*) which applies a systematic selection framework to determine representative GCMs that capture the range of climatic projections avoiding redundancy. That is, it measures the variability among all GCMs and provides a set of models that maximize display of diversity, but avoid redundancy and computation time. So, the three GCMs that we chose: BCC-CSM2-MR, MRI-ESM2-0, and UKESM1-0-LL represent different climate ranges and scenarios. With these three chosen GCMs, we constructed our ensemble forecast. Here is an example of the results provided by chooseGCM (see complete figures in Supplementary Data).

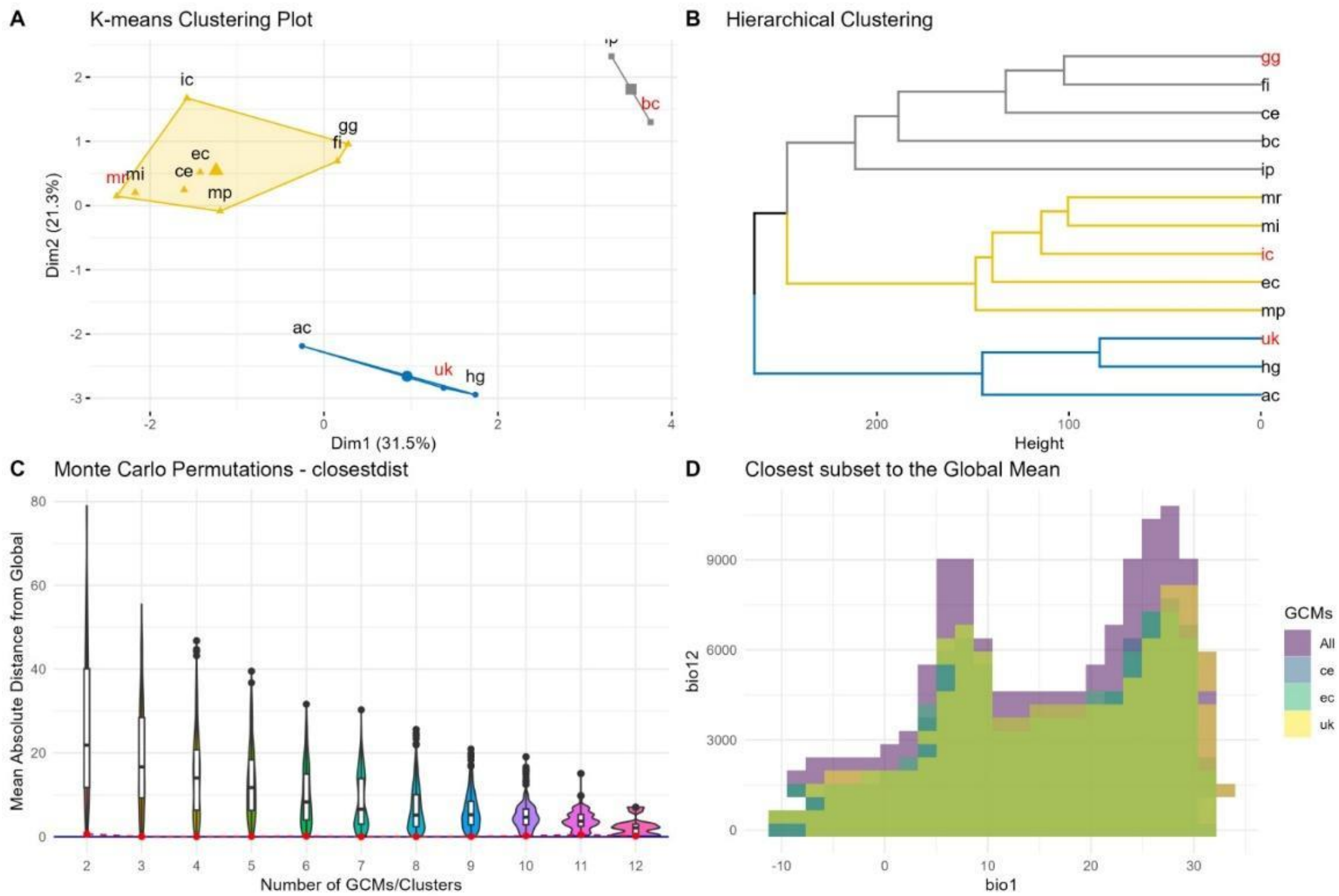

Line 581-587: You can list the retained post-correlation test variables that were used to construct the ENMs.

Answer: Thank you for your suggestion. We have placed this information in the new **Supplementary Table S5.** Here, readers can find the set of uncorrelated variables used to calibrate the niche models for each species complex.

Line 588-589: To me, this sentence makes it sound like only the MaxEnt algorithm was used. Whilst MaxEnt is a generally reliable model, an ensemble approach using multiple algorithms (GAM, GLM, RF, etc.) would be superior, and would result in an averaged model. I strongly urge the authors to consider this approach.

Answer: Please, see our reply to general comment #3.

Line 597: Which specific threshold values? There are a number to choose from.

Answer: We have specified the exact threshold value: "Using threshold values derived from omission rates at 5% (E = 5%), we converted these maps…"

Line 621-623: Perhaps provide some information on the percentage of species that currently have such a classification.

Answer: Added as suggested:

> "…current extinction risk using IUCN criteria (21); where approximately 17% are listed as Data Deficient or Not Evaluated".

Line 638: Low scores for "Not Evaluated" species is misleading. These species may yet to receive assessments because they are recently described, but they may be highly threatened nonetheless.

Answer: Certainly. We understand that scoring Not Evaluated (NE) species lower is a conservative approach that might not reflect their real level of threat. However, without formal assessment data, we cannot justify a higher priority score. This is different from Data Deficient (DD) species, which received a higher score because they were considered during evaluations that recognized potential conservation issues, yet failed to present sufficient data for a definitive categorization. So, we recognize that the NE status indicates an absence of evaluation rather than confirmed low risk, and it is possible that one or more NE complexes in our study may emerge as significant threatened under evaluation.

Results

Line 114 and 116: The genus level information conflicts between these two lines.

Answer: The reviewer is correct. We analyzed 65 genera through 63 phylogenetic analyses. Two genera with limited number of sequence data were analyzed with their respective closely related taxa to ensure adequate phylogenetic resolution and stable species delimitation results. We have clarified this in the main text: "We analyzed a molecular dataset of 12,813 sequences of Neotropical bats and constructed 63 phylogenies covering 65 genera to evaluate the hidden diversity patterns in this group".

Figure 1: Very nice figure. This is more of a suggestion than comment, but it would be nice to colour code the sampling records (i.e., the points on the map) to each family.

Answer: We thank the reviewer for the appreciation and suggestion. We have placed family-level maps in the Supplementary Data that will enable readers interested in specific taxonomic groups to assess the geographic distribution of genetic data for individual families.

Figure 2: The image of the bat and its silhouette is not necessary. The numerical information for described and undescribed species is already provided.

Answer: We respectfully maintain the bat silhouette visualization for its communicative implications beyond just the numerical data. The size of the proportional shadow gives readers an instantaneous visual grasp of the extent of hidden diversity in described species.

Line 160-165: This information is detailed in the Methods section so no need to repeat it here.

Answer: Reviewer is correct. We agree and removed this part.

Figure 3: It is interesting that HSR is quantified for each biogeographic region, whilst the other three metrics produced continuous layers across the whole of the neotropics. Is this not achievable for HSR? This would greatly inform conservation efforts.

Answer: HSR is a composite metric incorporating discrete sampling events (genetic sequences), accessibility calculations, and locations determined through biogeographic delimitations, all normalized to the provincial boundaries. Trying to interpolate HSR

between provinces would introduce artificial gradients that did not reflect real gradients of hidden diversity or sampling effort. Further, the province level fits precisely with the realities of ecology and biogeography because these provinces represent separate evolutionary and environmental units, which inherently differs between them in terms of species assemblages and the processes of diversification.

Line 205: Check phrasing on this line.
Answer: We agree the phrasing was unclear and have revised the line to improve flow.

Table 1: This table is interesting but is extensive, and may therefore be better suited to the supplementary information.
Answer: We agree that the table is extensive, but it is also the primary actionable output of our analysis, the ranked list of species complexes that urgently need taxonomic action. Conservation practitioners, funders, and taxonomists need this information in an accessible format that does not require them to review supplementary materials. For this reason, we opted for maintaining it in the main text, based on our approach to conservation planning.

Line 240: I would not call a geographic range size of 662,476 km² "very restricted"! It may be comparatively smaller than other species in this study, but it is a very large area and would not meet the criteria for a threatened IUCN classification. Should this comma be a decimal place?
Answer: Indeed. We changed "very restricted" to "comparatively restricted".

Discussion
Line 263: "disciplinary"
Answer: Corrected as indicated.

Line 265: The phrase "harbor undescribed taxa" is strange to use when referring to already described species. You can just state that these species complexes may contain new taxa.
Answer: We have changed the text accordingly. "…which means that approximately a quarter of currently recognized bat species may contain new taxa".

Line 278: What exactly is meant by "environmentally complex"?
Answer: We agree this term needs clarification and have made it more specific. We remove "environmental complex" by:

> "…which predicts higher speciation rates in areas with high environmental heterogeneity, including complex topography, diverse habitat types, variable climatic conditions…"

Line 285-286: Extend this sentence to clearly explain why conservation planning at these sites is not currently possible. This would not be obvious to readers not working in/around these zones.

Answer: We have expanded this information as suggested:

> "Based on patterns observed in adjacent well-sampled regions, these areas may harbor undocumented diversity, but the absence of genetic data currently prevents their inclusion in conservation planning. Limited sampling caused by insufficient funding, difficult access, social instability, and lack of institutional support perpetuates these knowledge gaps. Without genetic data to identify hidden species and evaluate their distributions and threats, effective conservation strategies cannot be formulated"

Line 305: Here you can specify which specific climate change scenarios. Your methodology looked at two separate scenarios, but here it is not clear whether you are referring to both, or a single one of these.
Answer: We have clarified this section as suggested: "…harbor hidden diversity in regions projected to lose between 25 to 50% of their suitable habitat under both moderate (SSP245) and severe (SSP585) climate change scenarios. This is particularly concerning in the Amazon…"

Line 309-312: This is very true for bats in temperate regions, but I think torpor is less of an issue in tropical zones, such as your study area, where bats may be active year-round.
Answer: We thank the reviewer for this observation. Although torpor occurs in some tropical regions, and the Neotropics also includes some subtropical and temperate climates where torpor can occur more frequently, we agree that this is not the case for the majority of the taxa and for most of the region and, as such, have removed the reference to torpor in the text.

Line 316-319: Certainly, disturbed and degraded habitats provide new opportunities for some species. You can expand on this here to state why.
Answer: Indeed. We have added an explanation about this: "...many undescribed lineages persist in anthropogenically modified landscapes, as evidenced by the significant overlap between high HSR values and elevated Human Modification Index scores. These patterns may reflect the ecological plasticity of some bat taxa, such as generalists relying on anthropogenic resources such as crops, insects associated with livestock, or roosts in human-created habitat. Moderate habitat disturbance may also create edge habitat and resource heterogeneity that can benefit some bat guilds.".

Line 328-330: I wouldn't necessarily agree with this. While there is certainly a decline in the number of taxonomic experts, this is not necessarily the case for the bat evolution field. Further, integrative taxonomy is becoming evermore common, and so accurate and reliable species descriptions can be made by multidisciplinary teams of geneticists, ecologists, morphologists, and bioacousticians.
Answer: This is a very interesting point. We agree with the reviewer regarding the advances of integrative taxonomy that have improved species description potential in some places, including the neotropical region. That said, we respectfully disagree that taxonomic

capacity is not a limitation in the Neotropics (and perhaps elsewhere). While there are integrative taxonomic tools, and there are many and extremely expert researchers engaging in taxonomic work in the Neotropics, the capacity for species description in this part of the world is slower than it should be due to continuing limitations. In many neotropical countries (not all, we agree) researchers experience funding restrictions related to conducting molecular analyses, restricted access to sequencing facilities and access to museum collections, bureaucratic barriers for obtaining fieldwork permits and conducting international collaborative work, and limited institutional infrastructure. While these limitations do not preclude taxonomic research, they slow it down tremendously, which is a worrying matter for a taxonomic community when undescribed species are experiencing rapid habitat loss. What may take months to complete at a high-capacity institution could take years (many years sometimes) in some Neotropical contexts. This is obviously also true for other regions of the World, even if the scenario has been significantly improved globally in the XXI century. We have toned down our sentence which now reads as follows:

> "Taxonomic expertise itself is a limited resource, with a critical imbalance between the vast number of species yet to be described and the number of trained taxonomists available to describe them (*45, 46*)".

Line 333-335: Perhaps provide an example or two of such conservation approaches that cover all species.
Answer: We have added specific examples to illustrate conservation strategies applicable across different range sizes:

> "For instance, strategies for habitat protection must include both small critical areas for endemic lineages as well as landscape-scale connections for widespread species. Climate change mitigation needs to include both the preservation of local microrefugia protection for range-restricted taxa as creation and maintenance of regional connectivity for mobile widespread complexes".

Line 362-363: See my previous comments concerning the ENMs. An ensemble approach would improve this robustness.
Answer: Please, see our reply to same answer as the one given to general comment #3.

Line 371: Neotropical bats are presented as one of the most studied groups of mammals, but where is the evidence for this? Do you have a reference to support your statement?
Answer: We have added two references to support this statement.

Line 369-376: Currently I feel as though this discussion is missing a paragraph linking the findings to the broader picture. The points discussed are applicable to bats in other areas of the world, and to cryptic species more generally, and linking this study

to the global-scale conservation situation will greatly enhance the scope of the manuscript.
Answer: We agree with this valuable suggestion and have added a paragraph to the Discussion that contextualizes our findings within the global biodiversity crisis and their applicability to other taxonomic groups and regions.

Reviewer #2

Answer: We would like to thank Reviewer #2 for participation in Nature's co-review program and for sharing comments as part of the peer review process. We appreciate the time and energy spent on reviewing our manuscript alongside an anonymous reviewer.

Reviewer #3

1. **Visually, the HSR index (Fig 3A) looks to be very strongly correlated with the locations of the genetic samples (Fig 1), but no mention is made of this. Despite the weightings of the HSR, I get the feeling that it simply represents where past work has been done (rather than show where hidden species richness occurs). I think that the authors need to convince the reader that: 1) there is no correlation between the two figures mentioned above, and 2) if there is a correlation, explain why we should believe in the HSR (an easy explanation could be that taxonomists have preferentially sampled species rich areas in the past, avoiding areas with low diversity - it is hard to justify funding an expedition to a species poor location!).**
Answer: We appreciate this important point raised by the reviewer. The reviewer is right in pointing out that there is a correlation between the values of HSR and the locations of the genetic samples, but this correlation is moderate (Spearman $\rho = 0.584$, $p < 0.001$; see figure below). Importantly, this correlation does not undermine the validity of our HSR index, instead, it reflects historical realities in that genuinely diverse places have been subject to more research focus while the HSR framework tries to disentangle this confounding effect. The key significance is that raw sampling intensity alone cannot reveal hidden diversity patterns. The HSR index explicitly accounts for sampling bias with the use of logarithmic normalizations and weighting for accessibility, maintaining comparability among provinces irrespective of sampling intensity. The moderate correlation with sampling demonstrates that HSR is capturing real hidden diversity that may be partially (but not completely) reflected in historical sampling.

   To illustrate this quantitatively, we would like to highlight two aspects:

   1. HSR also highlights undersampled diversity hotspots. We found eight provinces with high HSR scores despite low sampling (e.g. Imeri, Ucayali, etc.). These represent diverse provinces that have been systematically understudied. Without the HSR framework, these hotspots would remain invisible.
   2. HSR differentiates truly diverse regions from heavily sampled but mostly non-diverse regions. For example, eight sampling-rich provinces (Western Ecuador, Antilles, etc.)

exhibit low HSR values. These regions have received substantial research attention over time but harbor lower hidden diversity, a pattern that raw sampling data alone would obscure.

Overall, these evidences clearly show that HSR does not simply mirror sampling effort.

Nevertheless, we acknowledge that, in some areas, historic sampling patterns are so strong that the HSR index may not fully eliminate their visual signature. Statistically, the HSR framework is designed to account for and correct for historic sampling. But when the historic research effort decades long have been so pronounced in certain areas (e.g., Guianan Lowlands with > 6000 sequences) a strong spatial structure still exists. This does not reflect a methodological limitation of HSR, rather, it demonstrates that historical research has preferentially targeted some diverse regions. The critical point is that statistical analysis reveals significant deviations from this pattern (the 8+8 provinces mentioned above), and these deviations represent the actionable conservation priorities that HSR is designed to identify.

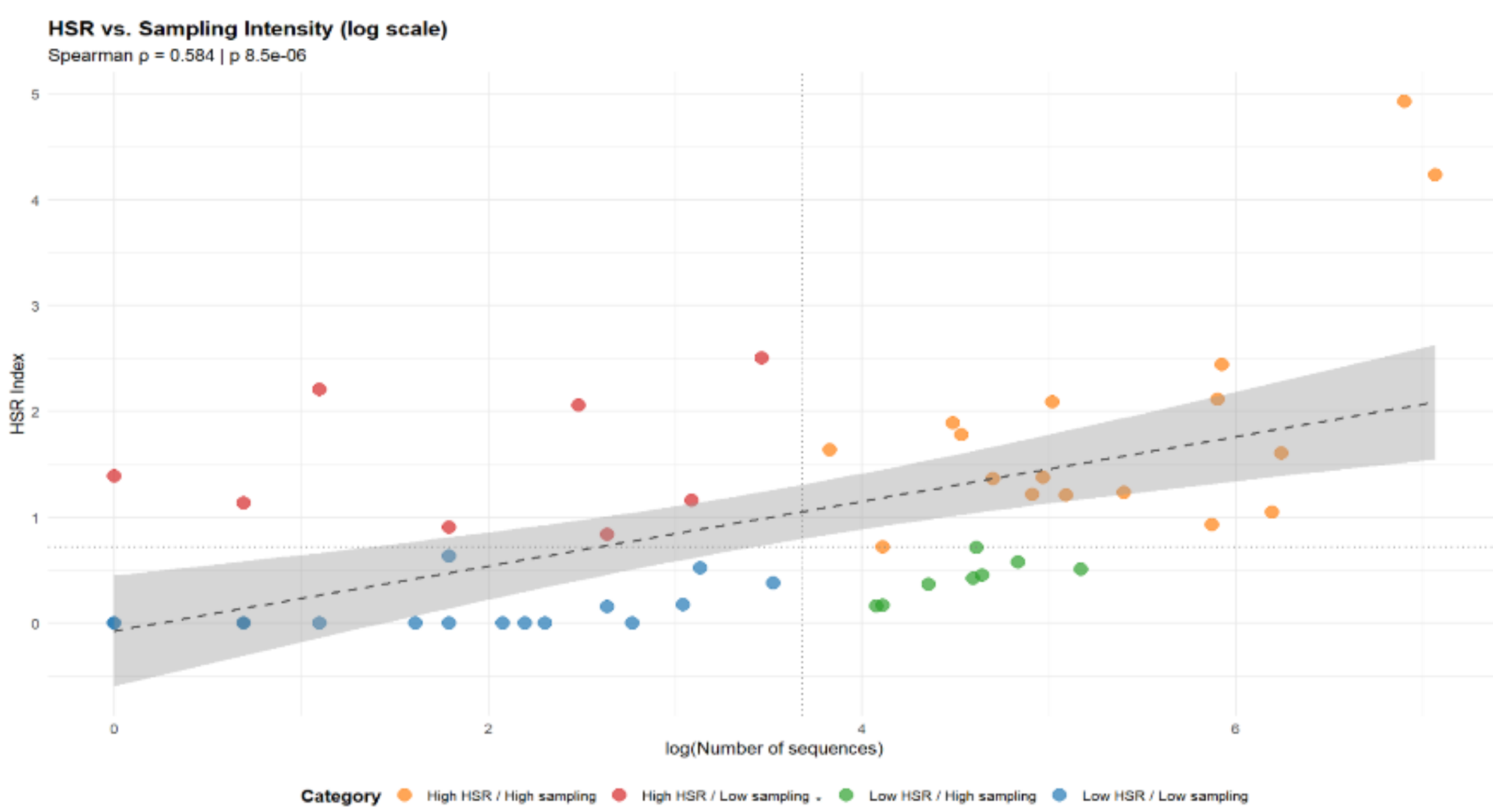

Relationship between HSR scores and sampling effort

We also explored other approaches, such as residual-based refinements, which could further reduce sampling correlation. However, they introduced artifacts due to over-prioritizing areas with insufficient data (for example, provinces with n < 50 sequences ranking highly under the priority index despite the limited data). We have added an explanation to the readers in the Discussion section, where we address the correlation of HRS index and sampling intensity, and explaining and clarifying this issue:

"Although our HSR index shows moderate correlation with sampling intensity (Spearman $\rho = 0.584$, $p < 0.01$), this relationship is expected: taxonomists have historically concentrated fieldwork in regions with high described species richness, where research funding is more readily justified. Nevertheless, the HSR framework successfully identifies critical deviations from sampling patterns, demonstrating its potential for revealing overlooked biodiversity hotspots".

2. **I couldn't help noticing that the future distributions of the species listed in Table 1 (supplementary figs S53 to S101) almost invariably show reduced future distributions. Are there no "winners" in this group? Species that would benefit from climate change? I find this hard to believe. At the very least, this requires some comment in the Discussion, with comparisons to other relevant studies from elsewhere in the world.**

   Answer: This is an important point, thank you for noticing it. While we agree with the reviewer that this consistent pattern of reduced ranges may seem unlikely, other studies have reached similar conclusions. Indeed, our finding aligns with several regional and global studies, which consistently predict that range contraction and extinction risk outweigh expansion, especially in systems where anthropogenic disturbance is high and specialized habitats are critical. Aguiar et al. (2016; https://doi.org/10.1016/j.gecco.2015.11.011) found that if Cerrado bat species were not able to move to new suitable areas and were unable to adapt, 31.6% could lose over 80% of their current distribution area, and five species would lose more than 98% of their distribution area in the Brazilian Cerrado; even in the unlikely scenario where species could be able to disperse to suitable areas in the future, they would still have their distribution ranges reduced. The majority of species would not lose, but also would not gain suitable area, while eleven would have slight gains in range. Recently, Bührs et al. (2025; https://doi.org/10.1002/2688-8319.70086) modeled future distribution of birds in urban areas, and even in this human-altered scenario, all species would either lose or not change their ranges. In another large-scale analysis by our own research group, we modeled the future habitat suitability for Atlantic Forest birds (n=775 species), and between 80% to 92% of the evaluated species would lose potential suitable areas, depending on the scenario, with only 8% of the evaluated species showing expansion of their potential area (Franco et al. in press).

   Consistent with these previous studies, we also found that the majority of species complexes exhibit net range losses (~ 90% under SSP2-4.5, ~96% under SSP5-8.5). However, we did identify five species complexes showing net range gains under SSP245 (*Cyttarops alecto* +16.86%, *Noctilio albiventris* +13.12%, *Rhynchonycteris naso* +8.19%, *Pteronotus personatus* +7.84%, and *Peropteryx macrotis* +2.67%) and two under SSP585 (*Peropteryx macrotis* +4.65% and *Cyttarops alecto* +2.76%). Nevertheless, the magnitude of these gains is substantially smaller than losses, and gains diminish markedly under more extreme scenarios (see Figure below). We have now included this in our Discussion.

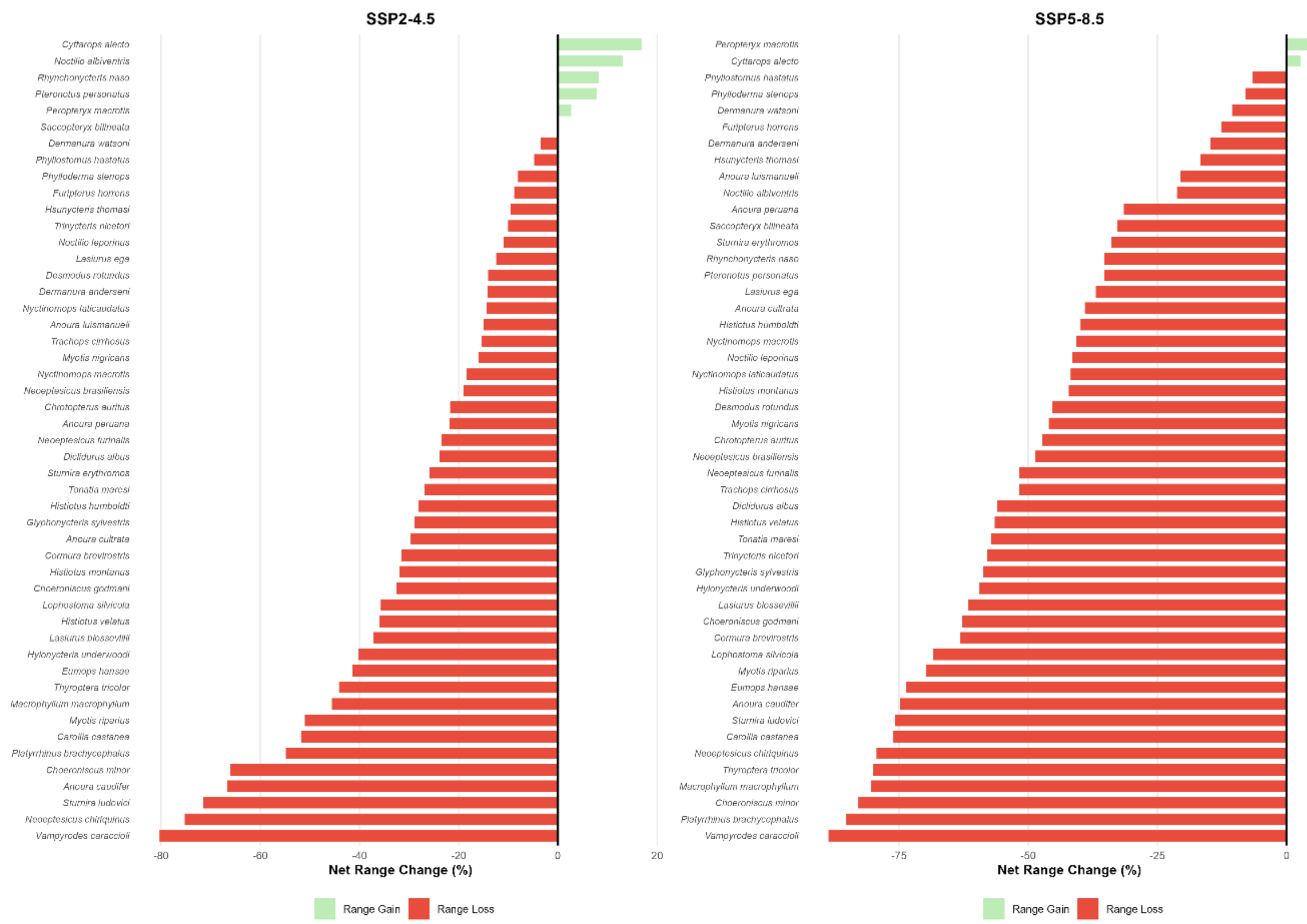

Net range change by species under climate change scenarios

3. **Considering the critical state of the taxonomy of Neotropical bats (shown by this study), I am surprised that they have not made any effort to compare this with other tropical hotspots for bats (Africa and South-East Asia). I think a paragraph in the Discussion, showing parallels with other hotspots, would strengthen their argument here (by showing that this is a global problem, and not just a South/Central American problem).**

   Answer: We agree with the reviewer that contrasting our findings against other tropical bat hotspots adds to the global significance of our work. We have added to the final paragraph of the Discussion specific comparisons with Southeast Asian and Afrotropical bat faunas. These additions demonstrate that the patterns we document in Neotropical bats, a high level of hidden diversity in threatened biodiversity areas, are also trends that have emerged from molecular studies in other tropical regions. Thus, the similarities across biogeographic realms indicate that the taxonomic crisis we address in this study is a global conservation problem that needs urgent international coordination, not solely a Neotropic case. The text reads as follows:

   > "While our study focuses on bats, the trends and issues we identify have broad implications for the biodiversity conservation globally. Hidden diversity is not unique to bats or the Neotropics; undescribed species complexes are increasingly identified across a multitude of taxonomic groups and biogeographic regions, from Southeast Asian amphibians, to small mammals in Africa, and invertebrates across the globe

(53). The taxonomic challenges that we report for Neotropical bats are consistent with patterns that emerge from other hotspots of bat diversity in the tropics. Recent phylogeographic studies with Asian chiropterans have identified comparable levels of hidden diversity, especially in morphologically conservative groups, such as Hipposideridae and Rhinolophidae, where 20-40% of currently recognized species contained lineages yet to be formally described (54,55). Similarly, Afrotropical bat assemblages show extensive hidden diversity (56,57) in areas with rapid land-use conversions. These convergent patterns across bat faunas demonstrate that the race to document hidden diversity before extinction is a global imperative for its conservation. Our results highlight the urgent need for international collaboration to accelerate taxonomic research, prioritizing biodiversity hotspots where institutional capacity and funding are limited. Addressing hidden diversity is not only an academic exercise in the field of systematic biology, it is essential to understanding the true scale of the current extinction crisis, and to implement conservation strategies that consider the undocumented biodiversity worldwide".

4. **I am not familiar with the geographic definition of "Neotropics" (and I'm certain that most readers of this manuscript will not be either). I am particularly interested in the total lack of records from the central and southern parts of Argentina (interestingly, there are two records from Chile, within this zone). Is this because this region lies outside of the Neotropics? An easy solution to this is to draw an east-west line on the map in Fig 1C to show the southern limits of the area of interest.**
   Answer: We thank the reviewer for this comment. First, we have added the definition of Neotropics we followed in the text. Second, surprisingly, the lack of genetic data is not due to geographic exclusion, indeed these results reflect a significant lack of genetic sampling in the southern part of the Neotropics. After applying our quality control filters (sequence length >590 bp, removal of stop codons/indels, taxonomic validation), we were not able to find sequences from those areas. We think that the absence of data is a result of the combined effects of several factors: (1) areas that have naturally low bat species richness and abundance relative to tropical latitudes, (2) poor funding and infrastructure for genetic sampling especially in more recent times in Argentina, (3) typical extreme conditions (extreme weather, low population densities) that can limit sample collection, and (4) a historical bias towards sampling areas in tropical assemblages that are more diverse. Thus, this gap is exactly the type of "genetic deserts" we are referring to in the manuscript (lines 285-295), large regions that have been undersampled and where more genetic data are needed.

5. **I like the fact that they have created a list of priority species (Table 1), which includes input from various sources e.g. human modification, climate change, red list, etc. Is there nothing to compare this table to? For example, is there no study that has tried to prioritise bat species for conservation for this region? Or perhaps individual countries (e.g. Brasil). In this case, drawing on these studies to support their claims for their priority listing, would be useful?**

Answer: We appreciate this valuable suggestion to contextualize our study within existing conservation efforts. Several regional studies have addressed bat conservation (specially for endemics and threatened species) in portions of the Neotropical region, and have been included with the corresponding references. The recent national re-evaluation of the conservation status of all recognized Brazilian bat species is highly important. However, it is also notable that all species categorized as officially threatened (VU, EN, CR) in this national assessment are cave-dwelling bats. While this national Red List assessment is extremely valuable for species-specific management, the fact that only cave-dwelling taxa were categorized as threatened limits its utility as a direct comparator to our multi-criteria list, which includes a broad taxonomic and ecological range of non-cave-dwelling species facing climate and landscape modification threats across the entire Neotropical region.

6. **The authors end the manuscript with a plea "The race against extinction...... never been more important". I fully support this claim, but the problem is that nobody seems to be listening. Could they not include a statement for the support of more taxonomists (or at least for more taxonomic studies)? Perhaps if taxonomists could be enticed to start working on the tabulated list, starting with the highest priority species, it would make for more meaningful progress.**
Answer: We totally agree with this suggestion. Action is required! We add the following sentence in the Discussion section:

> "…We urge the taxonomic community to focus on those species' complexes assigned to very high and high priority levels (Table 1) given their elevated risk of extinction. Targeted taxonomic efforts on these 24 complexes, using integrative approaches that include evidence from molecular, morphological, acoustic, and ecological data, would permit the formal recognition of dozens of species in a ten-year timeframe, potentially allowing incorporation into a conservation framework before local extinction occurs".

7. **I have added their manuscript with a few very minor comments/corrections.**
Answer: We thank the reviewer for his/her thorough revision. All comments and corrections have been incorporated into the main text.

Reviewer #4

1. **Species delimitation: It is typically straightforward to use single locus species delimitation software on barcode sequences. However, the authors failed to thoroughly vet the assumptions of each species delimitation model in light of their sampling. This is a major drawback with the single locus (COI barcode) species delimitation approach used here. The limited and biased sampling that exists in COI sequences for most of the species mined artificially boosts up genetic structure, which is a violation of one of the main assumptions of each of the delimitation models used. This is especially the case for the top two species in Table 1, which are considered here as the highest priority for hidden species complex, yet they are represented by very few samples and a very biased geographic distribution of sequences. Interestingly, the authors suggest that these two top species each could be a complex of 2 species. It is unclear why these then rank higher than others that may have a higher estimate of hidden diversity and are in geographic regions that are equally imperiled**

   Answer: We appreciate this value comment about our species delimitation procedure. These methodological concerns require clarification about our approach and the decisions we made.

   First, we acknowledge that our analyses were constrained by the number of genetic sequences available in public databases. However, this represents the current state of genetic sampling for Neotropical bats, a reality that required us to work strategically within the existing data. Despite these constraints, our final curated dataset comprised 12,813 high-quality COI sequences, representing one of the most comprehensive genetic dataset of Neotropical bat diversity to date. This dataset provided sufficient data density for most taxa we analyzed.

   Second, we conducted pilot analyses to determine the optimal analytical scale. We systematically tested delimitation at multiple taxonomic levels (families, tribes, and genera). Analyses at broader scales (families and tribes) produced algorithmic instability and biologically implausible results that conflicted with established taxonomic knowledge and previous molecular studies. In contrast, genus-level analyses yielded results that were both analytically stable and biologically coherent. This empirical testing, documented in our Methods section, provided evidence that genus-level analysis represents the optimal balance between data availability and analytical reliability for single-locus delimitation, in the context of our study.

   Third, the genus-level approach offered specific methodological advantages that address the reviewer's concerns about sampling limitations. Species within each genus exhibit more similar evolutionary rates, which is fundamentally important for single-locus delimitation methods because rate homogeneity allows these methods to more accurately distinguish intraspecific variation from interspecific divergence and properly identify barcode gaps [Puillandre et al. (2012) *Mol. Ecol*.; Hubert et al. (2024) *Methods Mol. Biol*.]. In addition, our genus-level datasets showed adequate sample representation, with a good average of

sequences per genus (mean = 173) across the 63 analyzed phylogenies. Importantly, in the few exceptional cases where genera had insufficient sequence data, we proactively combined them with their phylogenetically closest relatives in single analyses. This strategy ensured more sampling density, and adequate resolution while minimizing artifacts associated with sparse sampling in specific groups, as the combined genera were closely related and thus maintained an evolutionary rate that was not so extremely distant.

Fourth, our results are frequently corroborated by independent studies employing different molecular markers, which provides strong, albeit indirect, external validation that our delimitations reflect genuine evolutionary patterns rather than sampling artifacts. For example, *Furipterus horrens* has relatively limited COI representation in our dataset, in which we find that what is recognized as one species is at least two. A recent published study using *Cytb* found even greater hidden diversity than we report with at least four species [Moras et al. (2025) *Mamm. Biol*.] Similarly, studies in *Pteronotus*, *Sturnira*, *Lophostoma* and many others genera showed patterns of hidden diversity using multi-locus phylogenetic approaches that were consistent with our findings [Pavan & Marroig (2016) *Mol Phylogenet Evol*.; Velazco & Patterson (2014) *ZooKeys*; Larsen et al. (2010) *Zootaxa*; Velazco et al. (2011) *Zootaxa*]. This convergence of evidence from other independent molecular datasets and analytical approaches reflects the reliability of our COI-based species delimitation methods.

Fifth, we implemented a conservative multi-algorithm consensus approach specifically designed to guard against over-delimitation. We required agreement among at least three of four independent methods (ABGD, ASAP, GMYC, bPTP) before designating any lineage as potentially containing hidden diversity. This consensus is methodologically critical because these methods operate under different assumptions and exhibit differential sensitivities to data characteristics. When multiple independent algorithms with these differential sensitivities converge on the same delimitation, this concordance can provide stronger evidence for genuine evolutionary divergence than any single method could offer [Luo et al. (2018) *Syst. Biol*.; Karbstein et al. (2024) *Trends Ecol. Evol*.).

Sixth, the reviewer raises concerns about specific complexes with limited sampling, particularly *Monophyllus redmani* and *Anoura luismanueli*, which was ranked as "Very High" conservation priority despite having relatively few sequences. However, several factors support this classification. These taxa possess naturally restricted geographic distributions (186,742 km² and 48,136 km², respectively), and for range-restricted species, smaller effective population sizes and reduced phylogeographic structure mean that even limited sampling can adequately capture genetic variation [Miravalles et al. (2024) *DNA barcoding*; Carstens et al. (2013) *Mol. Ecol*.]. Moreover, these complexes showed concordant delimitation across independent methods, suggesting robust evolutionary signals despite sampling constraints. Importantly, their "Very High" priority ranking derives not from species counts but from our integrated Unified Score, which synthesizes four independent criteria (each weighted 25%): IUCN conservation status, projected habitat loss under climate scenarios, current human-induced landscape modification, and

geographic range size. These complexes rank highly because they face extreme conservation threats, *M. redmani* has 82.74% of its range already under intense human modification as an island endemic, while *A. luismanueli* faces 65.25% current habitat modification and 44.93% projected climate-driven habitat loss. Thus, following our approach, a complex with two potential undescribed species facing 80% habitat loss requires more urgent attention than a complex with five potential undescribed species in relatively stable habitat (Table 1).

Finally, we consider that these lines of evidence indicate that our approach produces conservative estimates rather than inflated species counts. We identified 101 potentially undescribed species from 12,813 sequences. By requiring 3/4 method agreement and analyzing at optimal taxonomic scale with adequate evolutionary rate homogeneity, we built in multiple safeguards against over-estimation. In addition, independent molecular studies support our COI-based analysis, also indicating our estimates are conservative rather than inflated.

2. **Finally, this analysis lacks a thorough quality control step. It is assumed that sequences deposited in these databases are appropriately curated and the taxonomy is void of errors, which is not the case in these databases.**

   Answer: Indeed, GenBank and BOLD are known to have taxonomically misidentified sequences and other problems in data curation. However, we did apply several quality filters which were detailed in our methodology:

   > "… (2) *Data Completion*. Missing information (e.g., coordinates or voucher details) was retrieved from related publications or by contacting authors; (3) *Taxonomic Updates.* We updated nomenclature following the Mammal Diversity Database (52) and the batnames database (22). (4) *Quality Control*. We removed duplicate sequences, as well as those shorter than 90% of the typical gene length (COI < 590 bp). We also conducted a preliminary alignment for each dataset using MEGA X (58) with default parameters. During this process, we identified and excluded sequences with nonsense mutations, stops codons, insertions, and deletions as these anomalies were likely results of low-quality sequencing, erroneous amplification, laboratory contamination, or other errors. We tested for substitution saturation using DAMBE, following Xia (59). In all cases, Iss was significantly lower than Iss.c ($p < 0.05$), indicating that the data are not saturated and are appropriate for phylogenetic inference. We also corroborated the taxonomic identification of each sequence through phylogenetic trees and using BLAST. These quality control filters resulted in the exclusion of approximately 12% of the initially retrieved sequences, primarily due to length requirements and the removal of sequences with stop codons or indels. While this filtering reduced our overall dataset size, it ensured high-quality phylogenetic analyses and did not create substantial geographic gaps".

3. **Understandably, the authors are limited to the sampling available in public databases. However, single-locus species delimitation here is confounded with taxonomic designation. Unreliable delimitation results could artificially lead to incorrect prioritization for conservation in the context of this paper.**

   Answer: As indicated in our methodology, we performed several quality filters to ensure the phylogenetic identity of each sample. We also performed comparisons, when possible, with other studies. After the initial quality filters, each genetic sequence was traced back to its original publication, compared to the BLAST database, included in our pilot phylogenetic trees to identify inconsistencies, and then its taxonomy updated following MMD and batnames. By doing this, we can ensure that the genetic sequences and identified clades have a clear taxonomy and there is no doubt about their identity. All sequences that raised doubts about their taxonomy but also about their origin, were excluded. Still, we recognize that our decision constitutes a working operational taxonomic unit for the purpose of our work. Finally, in the Discussion section, we explicitly caution readers that conservation actions stemming from our list should be accompanied by targeted, integrative taxonomic studies to confirm species boundaries.

4. **it is interesting that the authors chose the bPTP model, which assumes constant partition rates, over the more recent mPTP version that relaxes this assumption -- this is not justified in the paper.**

   Answer: We had initially selected bPTP under the assumption that coalescent rates would be sufficiently homogeneous across genera. However, to address the reviewer's concern, we also conducted analyses with mPTP on all phylogenies, to objectively compare both methods. For these analyses with mPTP, we applied the MCMC implementation (https://mcmc-mptp.h-its.org/) with two independent runs of 1,000,000 generations each, sampling every 1,000 generations with a burn-in of 10%. Both datasets were analyzed with and without outgroups. Our comparison found that there was 72% concordance between the two methods (45 of the 63 analyses were congruent among delimitations). Furthermore, in the cases that differed, mPTP had fewer species delimited than bPTP. Importantly, these differences did not affect our final results because our consensus approach (requiring agreement of the majority of methods) maintained the same species complex identifications regardless of whether bPTP or mPTP was used. Based on our empirical results and taking the suggestion from the reviewer, **we have now included mPTP** as another independent delimitation algorithm to our study, providing further consensus with 5 independent methods (ABGD, ASAP, GMYC, bPTP, and mPTP). This allows for even more confidence in identifying hidden species, while addressing the reviewer´s methodological concern.

5. **bPTP assumes that branching events are independent from one another. However, this assumption is violated given the sparse sampling of the genetic data.**

   Answer: The reviewer is correct that assumption of independent events in bPTP may not hold if sampling is sparse. As [kapli et al. (2017) *Bioinformatics*.] have acknowledged, uneven sampling affects delimitation accuracy because it can artificially increase genetic distances between samples, leading to a violation in the assumption of independence. That said, there are good reasons to believe this is not a major concern in our study:

First, our genus-level analyses reduce this problem. Within genera we have a reasonable sampling density (mean = 173) and thus would have a better representation of branching patterns. The independence assumption is more severely violated when single sequences represent entire lineages separated by long branches, a situation which we worked to avoid through our analytical design.

Second, the 72% concordance between methods and the fact that mPTP actually delimited fewer species in few discordant cases, suggests that independence violations are not driving oversplitting in our dataset. If sparse sampling were severely violating independence and inflating species counts in bPTP, we would expect mPTP to consistently delimit fewer species than bPTP across all phylogenies, which we did not observe.

Third, our multi-method consensus approach provides robustness against any single method's violations. Even if bPTP's independence assumption is violated for some complexes, this represents only one vote among five methods in our updated framework.

6. **ABGD strongly relies on large datasets to make delimitation inferences. For many of the datasets presented herein, potential or "hidden" species are represented by as few as 1 sample, as is the case of the top two bats in Table 1, which would produce unreliable estimates of barcode gaps.**

   Answer: We appreciate the reviewer's concern related to the implications of sampling limitations on the ABGD-based species delimitation. We agree that the presence of singletons can reduce confidence in the estimates of barcode gap. However, these were quite limited in our dataset and the vast majority of taxa were sufficiently sampled, allowing for a good inference. Also, in instances with few representatives, we incorporated these sequences to their closest phylogenetic genus to increase the density of sampling. All of our identification was also not based solely on ABGD and hidden species were recognized only when most delimitation algorithms were consistent. We think that these steps minimize the risk of artefactual species delimitation due to undersampling.

7. **ASAP uses pairwise genetic distances and does not rely on phylogenetic reconstruction. Uneven and spatially biased sampling poses a problem here by artificially increasing distances.**

   Answer: ASAP is not a simple clustering by some fixed threshold: it ranks partitions according to a composite score that combines (i) a Monte-Carlo-derived p-value testing panmixia for each group that was merged, and (ii) a relative barcode-gap width (W); the final asap-score is the mean rank of these two metrics [Puillandre et al. (2021) *Mol. Ecol. Resour.*]. Partitions that show inflated distances within a given group due to sampling artefacts, therefore, will yield low probabilistic support and lower ranks. In other words, they would not be considered.

8. **Mapping hidden species: The method developed here poses an interesting way to examine potential hidden diversity. While the authors clearly describe their HSR index, sampling still becomes an issue. For many bats examined here (including those species that are considered as "high priority") there are only one or a handful of sequences used as evidence for "hidden diversity". This clearly assumes that these bats are evenly distributed across provinces, which is not true. It is unclear how this assumption is treated in the geographic designation. This is important especially because sampling is uneven. For example, as described in the manuscript, habitats across the Neotropics are quite heterogeneous. Thus, a sampled bat in Guyana, where a disproportionately large sample of the COI sequences come from, is not indication that the species is evenly distributed through the Dry Diagonal or the Atlantic Forest, regardless of geographic proximity.**

   Answer: We agree that this is a critical point. Importantly, our approach does not assume that species are evenly distributed among provinces; rather, the ENMs explicitly identify areas of environmental suitability for each complex, thereby preventing the overestimation of uniform distributions and highlighting habitat heterogeneity across the Neotropics. To mitigate sampling bias, we (1) designed HSR explicitly to control three main sampling sources (sampling effort, accessibility, and province area) via logarithmic normalization and Z-score weightings, (2) mapped HSR at the biogeographic-province scale rather than extrapolating presence from single coordinates to large regions, and (3) developed ecological niche models (ENMs) for species complexes, not individual hidden lineages, since we are aware that the geographical boundaries of the undescribed lineages are unknown. This rationale is explicit in the methods section of the manuscript.

9. **Niche modeling: The niche modeling section is quite standard for what studies focused on spatio-temporal range changes typically do. There are several major issues with this as implemented in the manuscript to reach the overall goal: 1. there is no connection between the niche modeling data and the sequence data. Many species have only a few COI sequences available, yet the niche models are built with the entire observation dataset for each species, which goes against what the authors are trying to do with their estimation of hidden diversity.**

   Answer: In our framework, genetic data are used only to delimit a species complex that contains evidence of hidden diversity. Ecological Niche Models (ENMs) are then created for each of these complexes. We use all available verified occurrence records and employ those data to model their potential distributions in current and future climatic conditions. We do not model hidden lineages independently because the geographic limits of undescribed lineages are unknown. Thus, modeling at the complex level is the most conservative and biologically relevant way to approach this analysis. This allows us to examine which complexes that underlie hidden diversity may be more affected by climate change. In fact, if a currently recognized species faces a potential reduction in its distribution range, and that species represents a cryptic species complex, it is likely that each hidden lineage within the complex faces a similar, or even greater, individual threat. Therefore, while sequence data and ENMs may seem separate, this separation is not a methodological flaw, but rather a hierarchical approach: genetic data establish the presence

of hidden diversity, and the ENMs provide the geographic and environmental context that describes if that hidden diversity continues or disappears under alternative futures.

10. **the niche models were built for each species independently. This assumes that the species are immutable and not responding to any biotic interaction with sympatric/syntopic members of the bat community.**

    Answer: We agree with the reviewer that our ecological niche models (ENMs) were developed independently for each species complex and do not explicitly incorporate biotic interactions among sympatric or syntopic species. This approach is consistent with the nature and scope of our modeling framework. ENMs are inherently correlative models that relate known occurrences to scenopoetic variables (e.g. non-interactive environmental factors such as temperature and precipitation) that approximate the species' fundamental and existing niches [Soberon 2007 *Ecol. Lett.*). This allows us to identify areas of climatic suitability, rather than to predict realized distributions constrained by biotic processes. Our goal was thus to estimate the climatic suitability and potential vulnerability of complexes harboring hidden diversity under current and future scenarios, not to model community-level coexistence dynamics. In addition, modelling including biotic interactions require detailed biological and ecological data that are rarely available for broad-scale, multi-species analyses across the Neotropics, for bats or other taxonomic groups.

11. **each species niche model is independent and based on different factors selected by the Maxent algorithm, so a change in one species cannot be compared to a change in another species because the models were essentially built with a different set of variables. It is unclear how this affects the overall goal of identifying species conservation priorities.**

    Answer: While it is true that each species complex has a customized variable set selected through collinearity filtering ($r < 0.7$), this species-specific optimization is not only acceptable but methodologically preferable to forcing identical variable sets across ecologically diverse taxa [Feng et al. (2019) *Ecol. Evol.*; Guisan et al. (2017) Cambridge Univ. Press]. Each complex has different biological responses to variables, for example, high-Andean species cannot have the same set of variables for modeling as Amazonian species. Here, we would be assuming that all complex species respond equally to variables, which is not biologically true. The selection of variables was tailored to each complex according to their reproductive biology, ecology, and general knowledge. This tailored approach is fundamental because it is intended to maximize the predictive performance (AUC/TSS) of each individual Maxent model. By ensuring the highest possible accuracy for each species' current distribution and future projection, we increase the robustness and reliability of every species' estimated range change, which is the direct input for our final index.

    Crucially, our overall goal is not to compare the internal ecological drivers between species' models, but to use the predicted proportional change in suitable area (the output of the models) to calculate a composite conservation priority index. This index is inherently comparative as it places each species on a common scale of vulnerability. Since the model output for each species is normalized and combined with other independent, non-modeling

factors (Red List status, Human Modification Index), the differences in the initial variable sets become less critical for the final, comprehensive prioritization score.

12. **the issue of non-transferrability of the models into non-analog future environments is a big problem here because there is a strong reliance on the results of the niche models to estimate the "conservation priority" ranking.**

    Answer: We appreciate the reviewer's concern about model transferability to non-analog climates, a limitation discussed extensively in the ecological niche modeling literature for over two decades [Williams & Jackson (2007) *Front. Ecol. Environ*.; Fitzpatrick & Hargrove (2009) *Biodivers. Conserv.*]. However, this critique applies equally to virtually all climate-niche studies published in high-impact journals, including hundreds of papers in Nature, Science, and others papers that form the empirical foundation for IPCC assessments and global conservation policy [Thomas et al. (2004) *Nature*; Urban (2015) *Science*; Warren et al. (2013) *Nat. Clim. Chang*.]. The consensus in the field is not that such projections should be avoided, but rather that they should be conducted with appropriate methodological safeguards and interpreted with appropriate caveats, both of which we have implemented.

    Several factors demonstrate that non-analog climates do not undermine our conclusions. First, non-analog conditions in the Neotropics by 2070 under moderate scenarios (SSP245) are geographically limited, primarily affecting high-elevation tropical zones [Williams et al. (2007) *Proc. Natl. Acad. Sci*.]. Most lowland and mid-elevation regions experience warming that creates conditions analogous to current climates at lower latitudes or elevations, enabling reliable model transfer. Second, we employed conservative extrapolation protocols (restrictive calibration areas, rigorous cross-validation) that represent best practices specifically designed to minimize extrapolation artifacts [Elith et al. (2010) *Methods Ecol. Evol*.; Merow et al. (2013) *Ecography*]. Third, our ensemble approach using three contrasting GCMs (BCC-CSM2-MR, MRI-ESM2-0, UKESM1-0-LL) selected to maximize climate space coverage accounts for uncertainty in future climate trajectories [Esser et al. (2025) *Glob. Chang. Biol.*]. Most critically, our conservation prioritization framework was explicitly designed to be robust to uncertainties in any single component. Climate projections contribute only 25% to the Unified Score, with the remaining 75% derived from current data (IUCN status, human modification, range size) that involve no future extrapolation.

13. **Some specific line points:**

    L119: this argument is unsupported and misleading as sequencing is 1. uneven across ecoregions and 2. biased towards easily captured bats that are common across Neotropical communities. The assumption that these species are "dominant across Neotropical ecosystems" omits that many bat species are not as easily captured with the typical sampling methods.

    Answer: Indeed. We have removed the argument as suggested.

Figure1: Panel B in Figure 1 is clearly missing an axis label. Maybe a little bit too picky, but it is strange that the bat used in the center of panel D in Figure 1 clearly does not represent any bat species found in the Neotropics. Why not use the same as in Figure 2?
Answer: We thank the reviewer for noticing this lapsus. We have now added the X-axis label, and updated the bat silhouette to a neotropical one.

L146: What is the definition of an evolutionary unit used in this manuscript? Given the biased sampling of COI sequences, I would question the estimates presented in Figure 2.
Answer: In our manuscript, "evolutionary units" refers to independently evolving lineages identified through molecular species delimitation algorithms and represent potentially distinct species. This is now clarified in the manuscript.

L171: This result is greatly dependent on biased COI sampling.
Answer: Although we acknowledge sampling heterogeneity in our dataset, this critique needs to be nuanced. The biogeographic patterns we document (hidden diversity concentrated in northern South America, eastern Amazonia, and southeastern Brazil) are not merely artifacts of sampling intensity. This assertion is backed up with several lines of evidence: (1) our HSR Index controls for sampling bias via logarithmic normalization and accessibility weighting; (2) independent ecological studies document high species richness and endemism in these same regions, regardless of genetic sampling [Willig et al. (2003) *Annu. Rev. Ecol. Evol. Syst.*]; (3) provinces with high sampling effort but low HSR (e.g. western Ecuador, Caribbean islands) demonstrate that our method successfully distinguishes genuine diversity from sampling artifacts; and (4) biogeographic theory predicts high diversification in regions characterized by topographic complexity and stable climates, and the regions we formulate fit this description. The moderate correlation between HSR and sampling ($\rho = 0.584$) indicates some dependence (partial), but not complete, with large deviations from the correlation line demonstrating genuine biological signals. This is precisely the signal our statistical framework was developed to detect and isolate.

Figure3: Panel A in Figure 3 exemplifies the issue with the genetic data mining and biased sampling. Note how the "HSR" hotspots are centered only where a high number of available COI sequences are present.
Answer: This is the same concern as L171, addressed above. We have provided extensive justification in our response to Reviewer #3's general comment about HSR-sampling correlation, including quantitative evidence of provinces deviating from sampling patterns (8 high-HSR/low-sampling and 8 low-HSR/high-sampling provinces). The visual correlation that the reviewer notes reflects a historical reality: some specific regions have historically attracted more research. However, our statistical approach identifies meaningful deviations from this pattern that reveal undersampled diversity hotspots and over-sampled lower-diversity regions. Rather than undermining HSR validity, the moderate correlation

demonstrates that the index captures a mixture of genuine diversity and historical sampling, with our framework disentangling these effects. We have now clarified this in the Discussion section.

L228: This sentence makes no sense.
Answer: We have revised it for improved clarity and logical flow.

L262: This genetic dataset is arguably extensive given that the focus is unevenly sampled COI barcode sequences.
Answer: We appreciate the comment. Indeed, our 12,813 high-quality COI sequences represent the most comprehensive compilation of Neotropical bat genetic data assembled to date, covering 68% of genera and 44% of described species across all families. While sampling is geographically uneven (an unfortunate unavoidable reality), the dataset's taxonomic breadth and quality-control rigor make it uniquely suited for continental-scale analysis.

L263: "multi-disciplinary"
Answer: Corrected as indicated.

L266: This is a wild speculation not based on how evolving lineages work.
Answer: We agree that the sentence was speculative and have removed it from the manuscript.

L285: This is unsubstantiated.
Answer: We have revised this statement to be more cautious. The revised text now clarifies that our inference about potential hidden diversity is based on patterns observed in adjacent sampled regions, rather than direct evidence from these data-deficient areas.

L578: The model calibration here uses a 200 km buffer around the species observation areas. This seems to have been applied arbitrarily to all species modeled without considering the biology of the species or the potential for overfitting the models given the disparity between presences and background localities used to model.
Answer: To clarify, the 200 km buffer was not constructed randomly, but rather follows an established best practice in the field of ecological niche modeling in relation to continental-scale analyses [Barve et al. (2011) *Ecol. Modell.*]. Using this approach allows us to balance two relevant concerns: (1) calibration areas must be large enough to capture environmental variation accessible to species over ecological time, and (2) calibration areas must be small enough to minimize the likelihood of including areas with distinct biogeographical histories and/or potential dispersal barriers. For flying mammals with high dispersal capacity like bats, 200 km represents a biologically reasonable accessible area. Measuring species-specific calibration areas would be ideal, however most of the species in the complexes lack data on their movement ecology. Regarding potential overfitting arguments: We have also used a robust model framework that tested the species data against 10,000 background

points and found that the model output had good validation statistics suggesting the model is able to predict with good performance and without overfitting.

14. **Remarks on code availability: While I reviewed the code cited, I did not have time to do a full test to examine if the analyses are replicable because it entailed data downloads from multiple sources. As written, it is not possible to run the code on a subset of the species without spending additional time modifying it.**

    Answer: We show readers step by step how to implement our HSR index on GitHub (https://github.com/diegodaem/HSR-Index). As in any other potential use of pre-written scripts it may take some time to set up the data and to slightly adjust the code. We have made a test with other data and it takes about 20 minutes to set up and organize the desktop and folders properly before running the script.

Racing Against Time to Unveil Hidden Bat Diversity in the Neotropics

Diego A. Esquivel*, Angie P. Penagos, Anderson Feijó, Maria João Ramos Pereira

*Corresponding author. Email: diegodaem@gmail.com

We sincerely thank Reviewers 1–4 for their valuable and constructive suggestions and contributions, which have substantially improved the clarity, robustness, and overall impact of our manuscript. Their insightful comments were particularly helpful in refining key methodological aspects and strengthening our analytical framework. We greatly appreciate the time and effort invested in improving our work. Below, we provide detailed, point-by-point responses to the comments raised by Reviewer #5.

REVIEWER COMMENTS

Third Round of Revisions

Reviewer #5

1. **I find that some conclusions drawn from the results lack sufficient caution — specifically, the emphatic assertion regarding the existence of 101 potential undescribed species across 52 complexes. The tone of this conclusion should be more conservative.**
   Answer: We thank the reviewer for this observation. We have revised the relevant passages accordingly. Throughout the Results and Discussion, we have replaced uses of "undescribed species" or "new species" with "*candidate species*" or "*putative undescribed species*"; these explicitly conveys the preliminary status of these units pending formal integrative taxonomic validation. We have also replaced "waiting to be formally recognized" with "*pending formal taxonomic validation*".
   Regarding the Abstract, we restructured the key sentence as: "*we identified 95 Neotropical bat lineages that may represent potentially undescribed species within 45 complexes*", a formulation that explicitly separates the molecularly delimited units (lineages) from their provisional taxonomic interpretation (may represent potentially undescribed species).

2. **The discussion should more explicitly state that these findings are based on single-locus species delimitation approaches with heterogeneous geographic coverage, while also highlighting potential gaps in the data.**
   Answer: We have expanded the limitations paragraph to explicitly foreground these two fundamental constraints as a coherent block within the section that discusses the COI marker:

   "*…Third, our analyses were performed using a single mitochondrial marker (COI), which is abundant in public repositories but inevitably introduces some uncertainty into species delimitation (49-51). While our conservative approach relied on agreement among multiple delimitation algorithms, using nuclear markers or whole-genome data would offer greater resolution. However, this would restrict our continental assessment due to limited available data. Nevertheless, when comparing our phylogenies*

with published papers using multiple genes, we obtained similar results in most cases, suggesting that our single-gene-dependent phylogenies did not significantly affect our main findings. Fourth, the geographic coverage of available COI sequences is highly heterogeneous across the Neotropical realm. Well-sampled provinces are more likely to yield detectable candidate lineages, while poorly sampled regions yield no detectable signal due to insufficient data rather than true biological absence".

3. **Finally, I suggest increasing the transparency of certain results within the supplementary material, thereby allowing readers — particularly taxonomists — to draw their own informed conclusions. For several taxa, such as Myotis, Neoplatymops, and Nyctinomops, the phylogenetic pattern is very clear: species are represented by a large number of COI sequences forming paraphyletic assemblages, suggesting that deep taxonomic investigation is needed. However, for many other species, I wonder if the phylogenetic patterns shown in the supplementary data might: 1) represent phylogeographic structuring, as two sister-clades with no reported genetic divergence are presented; 2) be explained by stochastic processes in the mtDNA (e.g., heteroplasmy, introgression, gene tree discordance) or curation errors related to a single individual used in the analysis.**
   Answer: We thank the reviewer for this constructive suggestion. We have implemented several additions to the supplementary material in response.

   First, concerning phylogeographic structuring: we incorporated a new table associated with Figs. S1–S45 reporting mean intra- and interspecific genetic distances. When available, we also include supporting evidence from other studies. These additions allow readers to independently assess whether the genetic patterns support the delineated candidate lineages by molecular algorithms.

   Second, concerning stochastic mtDNA processes: our quality control pipeline explicitly filtered sequences with stop codons, frameshift mutations, and nonsense substitutions, removing the most common signatures of NUMTs and sequencing artifacts. We acknowledge that introgression and gene tree discordance cannot be fully excluded with a single mitochondrial marker, which is precisely why we retained only lineages supported by independent algorithms and explicitly frame all candidate lineages as molecular hypotheses requiring integrative validation.

   Third, concerning number of sequences by lineage: lineages represented by fewer than three sequences or exhibiting low genetic divergence are now explicitly flagged as requiring additional sampling prior to any taxonomic inference. This criterion is clearly indicated in the supplementary material to facilitate critical evaluation by readers.

4. **Histiotus is a pertinent example. In Table S1, I noted that 3 out of the 7 analyzed species of Histiotus were identified as complexes. Nevertheless, the delimitation methods do not point to a majority consensus, as argued by the authors in the rebuttal letter (page 22).**
   Answer: We appreciate the reviewer's comment and clarify how our consensus approach was implemented. In most cases, the delimitation algorithms converged on the same set of clades when identifying putative species complexes. This agreement made it straightforward to define both the number of complexes and their boundaries, and the consistency is reflected clearly in the table.

   In a few cases, however, the algorithms inferred different numbers of lineages, although most clades were shared among methods. This occurred, for example, in *Histiotus*. ASAP recovered seven lineages,

ABGD eight (splitting one additional lineage), bPTP three, mPTP two, and GMYC seven. Despite these differences, the same seven lineages were consistently delimited by three methods. We therefore considered these seven as the consensus set of species complexes. Please find below a description of how we defined putative undescribed lineages. Importantly, this situation applied to only a few genera, as the algorithms generally showed strong agreement or did not agree. The case of *Histiotus*, as well as several others discussed later, is illustrated in Example 4 (purple clade).

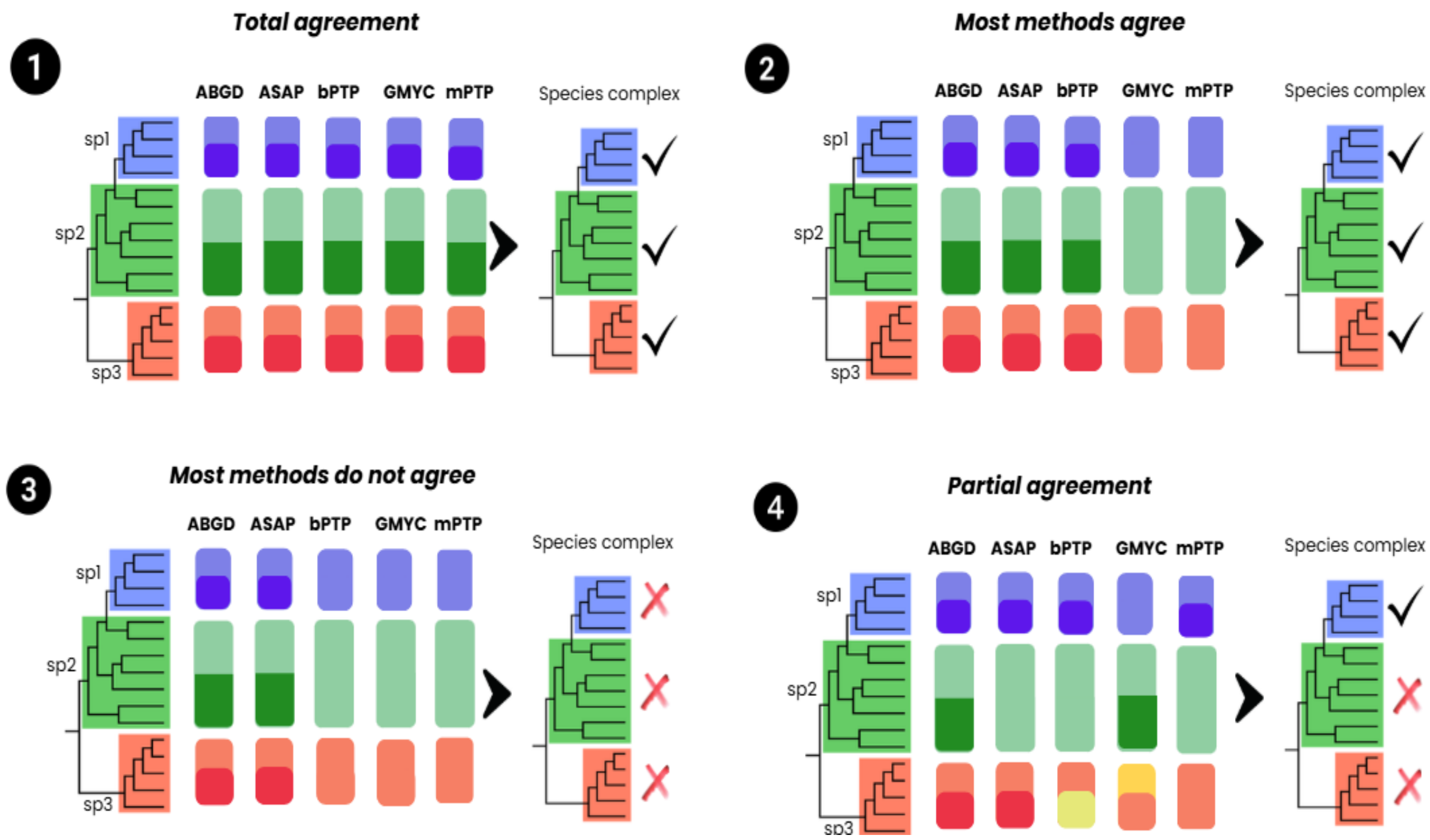

5. **While I understand that the authors adopted the most frequent result, I do not agree that this was a conservative choice. For example, the two lineages found in H. humboldti (page 42) are only 2% divergent. Moreover, for all three species, hidden lineages are inferred based on a single specimen, which violates the assumptions of the delimitation models used (as noted by Reviewer #4).**
Answer: We thank the reviewer for this important comment and have revised the manuscript accordingly, also considering the observations raised by Reviewer #4.

As justified in our previous response to Reviewer #4, we consider that our original approach did not severely violate the assumptions of the delimitation methods. However, to address the valid concerns raised by Reviewers #4 and #5, we have now adopted an even more conservative filtering of such cases, removing seven complexes (e.g., *Vampyrodes caraccioli*, *Platyrrhinus brachycephalus*, *Histiotus humboldti*, *Choeroniscus godmani*, *etc*.) that are poorly supported, either due to very low sample size (e.g., singletons) or minimal genetic divergence, reducing the total from 52 to 45.

In the revised version, we also explicitly flag those lineages that are represented by few sequences and are interpreted as putative undescribed species. These cases are clearly indicated in the supplementary material to inform the reader that caution and additional sampling is required.

We also clarify that genera lacking sufficient data to reliably run the algorithms had already been excluded prior to these analyses, ensuring that only phylogenies by genus with adequate sampling were

retained. As a result of these changes, our estimates of hidden diversity have been updated, decreasing from 52 to 45 species complexes.

Importantly, even after this more conservative assessment, the 45 complexes still represent 95 potentially undescribed species, reinforcing our finding that substantial overlooked diversity persists among Neotropical bats. All figures, tables, and supplementary materials have been updated accordingly to reflect these new estimates.

6. **This also appears to occur for Platyrrhinus brachycephalus, for which the divergence among the five COI sequences displayed in the tree is nearly 3% (a value potentially representing intraspecific variation), as well as for some Caribbean species, as also noticed by this same reviewer.**
   Answer: This is precisely one of the taxa that we have removed.

7. **I have highlighted some examples of lack of congruence in the species delimitation table that deserve further explanation or support. While many of them might have additional studies as evidence of hidden diversity, I found a lack of proper discussion in the text regarding these cases.**
   Answer: We have included, whenever available, the most up-to-date phylogeny for each group so that readers can compare our hypotheses with those currently accepted. Whenever possible, we prioritized multilocus phylogenies. This information is provided alongside each phylogeny; when it is not included, it is because no phylogenetic hypothesis is currently available for that group.

8. **I believe the results would be much clearer if the authors provided the mean divergence among COI lineages for each taxon in the supplementary material. I suggest including this information in the phylogeny/map figures. There is no need for two maps to show the geographic distribution of molecular clades and overall species sampling in Figures S1 to S52; instead, I suggest the authors use this space to show the divergence estimates among clades, displayed either as a table or within the phylogenetic scheme.**
   Answer: We have now added this information.

9. **The authors should draw attention to taxa with low genetic sampling, since downstream analyses using fewer than three sequences per COI lineage preclude geographic inferences and might bias the interpretation of results (perhaps by including a remark in Table S1, as they did for *insufficient data).**
   Answer: Thanks for the suggestion. We have also added this information in the Figs S1 – S45.

10. **I have additional observations regarding the supplementary data.**
    Figs. S1 to S52:

    Node Symbols: the circles representing node sizes/posterior probabilities are not clear; they should be replaced with more distinct symbols.
    Answer: We have revised Supplementary Figures S1–S45 by increasing the size of both the phylogenies and the maps. As a result, node symbols (posterior probabilities) are now clearly visible and easier to interpret.

Contrast: the light blue circles are difficult to see against the map background; I suggest a color change.
Answer: We have increased the size of both the phylogenies and the maps. As a result, the occurrence points are now clearly visible against the map background.

Taxonomic Context: in some trees, neighboring taxa were omitted (e.g., Phyllostomus hastatus, Platyrrhinus brachycephalus, Choeroniscus, Anoura). I suggest presenting the complete phylogenetic arrangement to assist taxonomists.
Answer: Thank you for this observation. We have now addressed this issue by including all relevant neighboring taxa in each phylogenetic tree.

Page 18: The symbol size is inconsistent (smaller) compared to other maps.
Answer: We have increased the size of the figure.

Page 35: The tree for P. brachycephalus includes a sequence for P. helleri. Please verify if this is a curation error.
Answer: This species is now excluded.

Page 43: Lineage colors are inverted on the map.
Answer: Thanks for catching that. We've fixed the lineage colors on the map

11. **I also have included a few comments in the rebuttal letter, main text and supplementary material documents.**

<u>**Main Text:**</u>

L31: I would say lineages, not species
Answer: Thank you for this suggestion. We have revised the text accordingly and now use "lineages": "*Here, we identified 95 Neotropical bat lineages that may represent potentially undescribed species within 45 complexes...*"

L32: may [referring to add the word "may" between "taxa" and "harbor…"]
Answer: The text has been revised to include "may" as suggested.

L37: same as above [referring change the word "species" by "lineages"]
Answer: The text has been revised to "putative species".

L534: "We conducted these analyses via respective web servers: the ABGD platform (https://bioinfo.mnhn.fr/abi/public/abgd/abgdweb.html) with settings for Simple Distance (p-distances) and a relative gap width of 1.5." **Was this the better-fitted model according to ModelFinder?**
Answer: ModelFinder was used exclusively for substitution model selection during phylogenetic tree inference, which serves as input for the tree-based methods (GMYC, bPTP, mPTP). For the distance-based methods (ABGD, ASAP), we deliberately used uncorrected p-distances, as empirical evidence consistently shows they perform equally well or better than model-corrected distances for barcode gap-based delimitation [Srivathsan & Meier (2012) *Cladistics]*.

<u>Rebuttal Letter:</u>

In Response to Reviewer #4, 1st comment about species delimitation:

In fact, Table S1 shows different choices for some taxa…example, Anoura, Choeroniscus, Desmodus

Answer: This reflects cases of partial agreement among methods, as explained above. Table S1 has been modified and, to avoid confusion, now shows the number of lineages identified by each algorithm only for detected species complexes. This makes interpretation easier, as the absence of these values indicates that no hidden lineages were detected.

When such divergence was not reached, which was your criteria then? This must be clear stated in your methods.

Answer: When such divergence was not reached, we did not consider those cases as evidence for hidden species complexes. Specifically, when fewer than three algorithms supported the same lineage delimitation, the result was treated as inconclusive and no hidden lineage was inferred. This conservative threshold is already stated in the Methods section: "*We used a conservative approach where hidden species complexes were defined when three or more algorithms identified the <u>same lineages</u> as a hidden species*".

In Response to Reviewer #4, 6th comment about species delimitation:

This remains as a point of concern to me. The recognition of hidden lineages (not species) did not follow a conservative approach in some cases (see the general comments)

Answer: We believe that the cases of concern arise from situations of partial agreement among methods. Delimitation approaches do not always recover perfectly matching sets of lineages; in some cases, they identify putative lineages only partially. We have now clarified how these situations were handled in our consensus framework to avoid overinterpretation.

<u>Supplementary material:</u>

Regarding TextS1:

Why w1 and w2 are not listed? Are these constant values? If so, this should be clearer in the text

Answer: This is correct, thank you for noting it. These values are not fixed and depend on each dataset. We have clarified this in the text by adding the following sentence: "These values must be calculated for each distinct dataset and are not universal".

Please discriminate in the formulae the values of w1 and z1

Answer: We have distinguished these values in the formulae and further clarified the examples provided.

Is this value correct? According to the hidden species description, it should be 100, right? Please correct the value reported for hidden species (it is now 100 instead of 10)
Answer: Thank you for noting this. We have corrected the value and made the example clearer and more intuitive.

Regarding Table S1

This is confusing… the value 0 does not make sense to me. If one species of Cyttarops has been evaluated, should not the delimitation approach recover at least one lineage?
Answer: That is correct, and we appreciate the reviewer for pointing out this source of confusion. We have corrected this by changing general presentation Table S1 and related inconsistencies in the revised version.

Substantial Neotropical bat diversity likely remains undescribed and vulnerable to extinction

Diego A. Esquivel*, Angie P. Penagos, Anderson Feijó, Maria João Ramos Pereira

*Corresponding author. Email: diegodaem@gmail.com

We sincerely thank Reviewer 5 for their valuable and constructive suggestions and contributions, which have substantially improved the clarity, robustness, and overall impact of our manuscript. Below, we provide detailed, point-by-point responses to the final comments raised by Reviewer 5.

REVIEWER COMMENTS

Fourth Round of Revisions

Reviewer #5

Supplementary material:

Regarding Figure S3: see also Pavan et al. 2012 (Evolutionary history of bulldog bats (genus Noctilio): recent diversification and the role of the Caribbean in Neotropical biogeography, Biol. J. Linn. Soc.) for additional evidence

Answer: Thank you for this suggestion. We have included Pavan et al. (2012) as recommended.

Regarding Figure S6, S32, S34: where is the graph? if not available, I suggest to remove number "4"

Answer: Thank you for the suggestion. Where this information was not available, we have removed the number as recommended.

Regarding Figure S41: see the study of Lim et al. 2017 (Phylogeography of Dominican Republic bats and implications for systematic relationships in the Neotropics. J. Mammal) for additional evidence.

Answer: We have included Lim et al. (2017) as recommended.

Regarding Table S1: I could not find these observations in the table

Answer: Thank you for noting this. We have ensured that the observations are included at the end of the table.
